# Evidence for Sequential and Increasing Activation of Replication Origins along Replication Timing Gradients in the Human Genome

**DOI:** 10.1371/journal.pcbi.1002322

**Published:** 2011-12-29

**Authors:** Guillaume Guilbaud, Aurélien Rappailles, Antoine Baker, Chun-Long Chen, Alain Arneodo, Arach Goldar, Yves d'Aubenton-Carafa, Claude Thermes, Benjamin Audit, Olivier Hyrien

**Affiliations:** 1 Institut de Biologie de l'Ecole Normale Supérieure (IBENS), CNRS UMR8197, Inserm U1024, Paris, France; 2 Université de Lyon, Lyon, France; 3 Laboratoire Joliot Curie et Laboratoire de Physique, Ecole Normale Supérieure de Lyon, CNRS, Lyon, France; 4 Centre de Génétique Moléculaire (CNRS UPR3404), Gif-sur-Yvette, France; 5 Commissariat à l'Energie Atomique (CEA), iBiTec-S, Gif-sur-Yvette, France; The Hospital for Sick Children and University of Toronto, Canada

## Abstract

Genome-wide replication timing studies have suggested that mammalian chromosomes consist of megabase-scale domains of coordinated origin firing separated by large originless transition regions. Here, we report a quantitative genome-wide analysis of DNA replication kinetics in several human cell types that contradicts this view. DNA combing in HeLa cells sorted into four temporal compartments of S phase shows that replication origins are spaced at 40 kb intervals and fire as small clusters whose synchrony increases during S phase and that replication fork velocity (mean 0.7 kb/min, maximum 2.0 kb/min) remains constant and narrowly distributed through S phase. However, multi-scale analysis of a genome-wide replication timing profile shows a broad distribution of replication timing gradients with practically no regions larger than 100 kb replicating at less than 2 kb/min. Therefore, HeLa cells lack large regions of unidirectional fork progression. Temporal transition regions are replicated by sequential activation of origins at a rate that increases during S phase and replication timing gradients are set by the delay and the spacing between successive origin firings rather than by the velocity of single forks. Activation of internal origins in a specific temporal transition region is directly demonstrated by DNA combing of the IGH locus in HeLa cells. Analysis of published origin maps in HeLa cells and published replication timing and DNA combing data in several other cell types corroborate these findings, with the interesting exception of embryonic stem cells where regions of unidirectional fork progression seem more abundant. These results can be explained if origins fire independently of each other but under the control of long-range chromatin structure, or if replication forks progressing from early origins stimulate initiation in nearby unreplicated DNA. These findings shed a new light on the replication timing program of mammalian genomes and provide a general model for their replication kinetics.

## Author Summary

Eukaryotic chromosomes replicate from multiple replication origins that fire at different times in S phase. The mechanisms that specify origin position and firing time and coordinate origins to ensure complete genome duplication are unclear. Previous studies proposed either that origins are arranged in temporally coordinated groups or fire independently of each other in a stochastic manner. Here, we have performed a quantitative analysis of human genome replication kinetics using a combination of DNA combing, which reveals local patterns of origin firing and replication fork progression on single DNA molecules, and massive sequencing of newly replicated DNA, which reveals the population-averaged replication timing profile of the entire genome. We show that origins are activated synchronously in large regions of uniform replication timing but more gradually in temporal transition regions and that the rate of origin firing increases as replication progresses. Large regions of unidirectional fork progression are abundant in embryonic stem cells but rare in differentiated cells. We propose a model in which replication forks progressing from early origins stimulate initiation in nearby unreplicated DNA in a manner that explains the shape of the replication timing profile. These results provide a fundamental insight into the temporal regulation of mammalian genome replication.

## Introduction

Eukaryotic chromosomes replicate from multiple replication origins that fire at different times in S phase [1]–[3]. In the yeast *S. cerevisiae*, microarray analysis of replicating DNA isolated from cells progressing synchronously through S phase first demonstrated that each region of the genome replicates at a reproducible mean time [4]. Similar findings have been reported for other eukaryotes including mammals [5]–[14]. The reproducible replication time might be interpreted to reflect a deterministic replication timing program, with replication origins located at specific positions firing at specific times in S phase. However, other methods had revealed that origins are often inefficient, firing in only a fraction of cells and being passively replicated by a fork emanating from another origin in other cells [15], [16]. Furthermore, single-molecule analyses of chromosomal replication intermediates showed that both time and order of origin firing are extremely variable so that no two cells use the same pattern of origin firing [17], [18]. These results favored a stochastic model for chromosomal replication where origins fire independently of each other and the mean replication time of each region is an ensemble average that only reflects the variable firing efficiencies of the surrounding origins [19]. Numerical simulations suggested that such models are compatible with the existing replication time course and origin efficiency data in yeast [20]–[22].

On the other hand, studies performed mostly in metazoan cells suggested that replicons are arranged in functional groups [23]. DNA fiber techniques revealed that adjacent origins are organized as clusters that often fire at similar times [24]–[30]. Intra-nuclear labeling of replication sites revealed discrete sites, or replication foci, that appear to contain multiple adjacent replicons and to correspond to stable structural units of both interphase and mitotic chromosomes [27], [31]–[34]. Furthermore, foci that replicate during consecutive time intervals are often spatially adjacent in nuclei and correspond to adjacent replicon clusters along chromosomes [35]–[40]. Therefore, origin clusters may correspond to stable structural entities that become available for efficient replication initiation at specific times in a sequence that depends on their order along the chromosomes. A study of the mouse immunoglobulin heavy chain region revealed a 0.4 Mb temporal transition region (TTR) that connects an early and a late replicating domain and is replicated by a single fork progressing in a unidirectional manner [41]–[43]. Studies of genome-wide replication profiles suggested that the dichotomy between 0.2–2 Mb domains containing multiple synchronous origins and 0.1–0.6 Mb originless TTRs that replicate in a unidirectional manner is a general feature of mammalian chromosome organization [8], [9], [11], [14], but the possibility that there is a gradual activation of origins in TTRs has also been considered [44].

Here we have performed a quantitative analysis of DNA replication kinetics using a combination of DNA combing data, genome-wide replication timing data and origin mapping data generated in this work or in previous studies in several human cell lines, as summarized in Table 1. We find that a large fraction of TTRs replicate at an apparent speed compatible with unidirectional progression of a single fork in embryonic stem cells. However, in differentiated cells or in cancer cells, most if not all TTRs replicate significantly faster than predicted by unidirectional progression of a single fork. Origins are activated synchronously in regions of uniform replication timing and more gradually in TTRs. We discuss how these findings may be reconciled with a stochastic model for replication timing. We propose an alternative domino model for origin activation in which replication forks progressing from early origins stimulate initiation in nearby unreplicated DNA and the space/time intervals between consecutive initiations explain the observed range of apparent replication speeds.

**Table 1:** Cells, DNA combing and replication timing datasets used in this study.

Cells	DNA combing (bulk genome)	DNA combing (specific loci)	Replication timing (genome-wide)	Origin mapping (ENCODE)
**Cancer cells**				
HeLa (adenocarcinoma)	This work.	*IGH* [this work]	[12], [this work]	Bubble trap [62] and λ-exo SNS [64]
K562 (erythroleukemia)	[50]		[13]	
**Embryonic stem cells**				
BG02			[13]	
H9	[61]			
H14	[61]			
**Fibroblasts**				
BJ			[13]	
MRC5	[58]	*FRA3B* [58]		
**Lymphoblasts**				
GM06990			[13]	
TL010			[13]	
H0287			[13]	
JEFF	[58]	*FRA3B* [58]		

## Results/Discussion

### DNA combing analysis of replication parameters in HeLa cells

**Figure 1:**
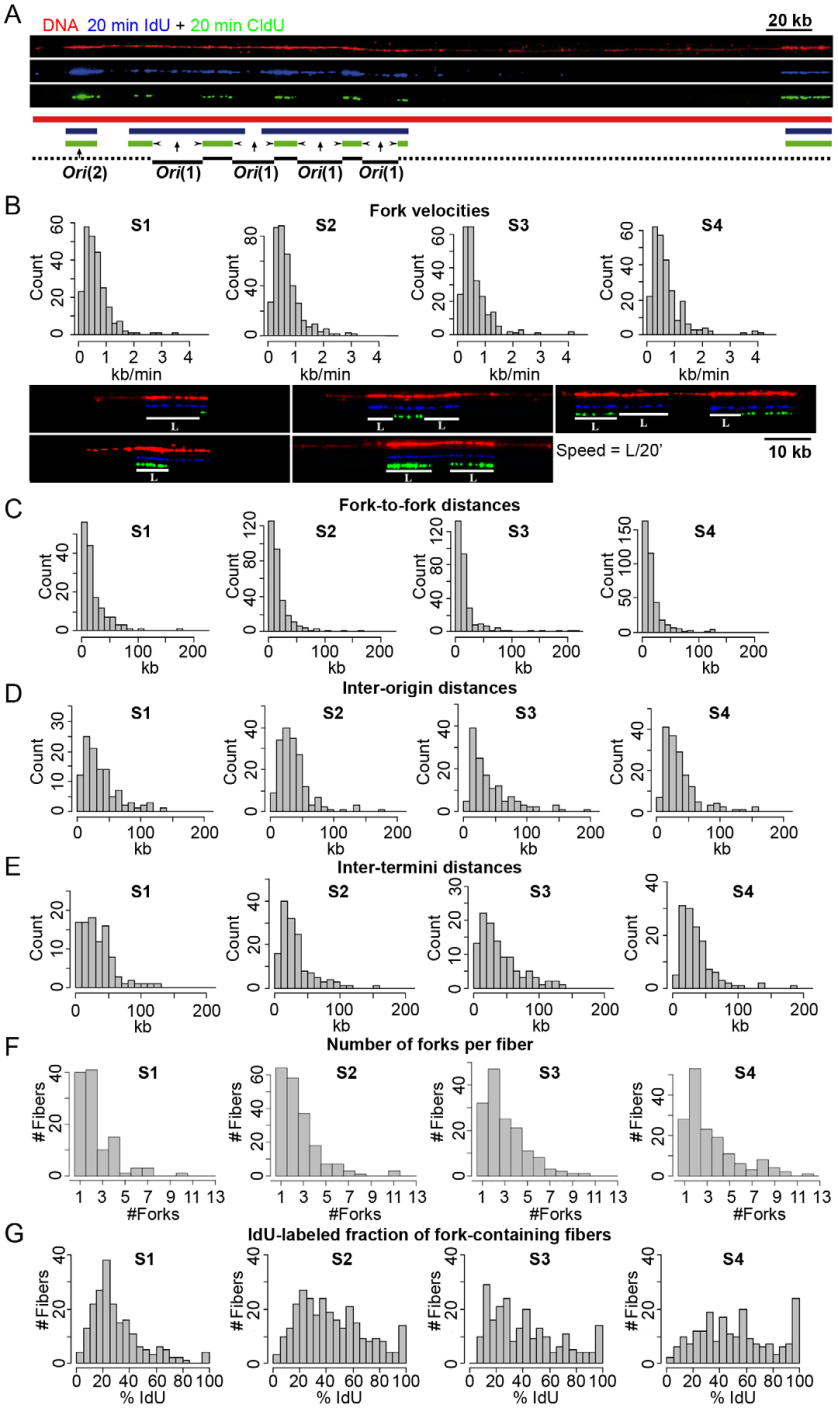
DNA combing analysis of DNA replication in HeLa cells. Cells were pulsed with IdU (20 min) followed by CldU (20 min) and sorted into four temporal compartments of S phase (S1, S2, S3 and S4). After DNA combing, DNA was stained in red, IdU in blue and CldU in green with fluorescent antibodies. (**A**) An exemplary DNA fiber and interpretative diagram. The blue-to-green transitions (indicated by arrows head) show the position and orientation of mobile forks at the time CldU was added (t = 20 min). This allows us to map origins that fired before (noted as *Ori*(1)) or after CldU addition (*Ori*(2)). Black solid lines show the intra-fiber distances between forks at the time of CldU addition. The dotted lines mark segments excluded from measurements of intra-fiber fork-to-fork distances. (**B**) Replication fork velocity analysis. Histograms of replication fork velocities in S1 to S4 fraction are shown. The five types of labeling patterns that could be unambiguously assigned to the progression of a single fork during 20 minutes labeling interval (white solid line) used to compute velocities are also presented. Each track length (L) was divided by the labeling time (20 min) to calculate the velocity of a single fork. (**C**) Distributions of local fork-to-fork distances in S1–S4. Only existing forks at the time of CldU addition were scored. For example, forks emanating from the leftmost origin in panel A, which fired after CldU addition, were not scored. Distribution of inter-origin (**D**) and inter-termini (**E**) distances in S1–S4. (**F**) Fibers containing ≥1 fork at the time of CldU addition were selected and the distribution of the number of forks per fiber was determined in S1–S4. (**G**) IdU-labeled fibers were selected and the distribution of the IdU-labeled length fraction of each fiber was determined in S1–S4.

We used DNA combing [45], [46] to measure replicon size and replication fork progression rate in HeLa cells at different stages of S phase (Figure 1). Asynchronously growing cells were pulsed with the halogenated nucleotide IdU for 20 min followed by CldU for another 20 min, fixed and sorted into four temporal compartments of S phase (S1, S2, S3 and S4) according to total DNA content. DNA was stretched on coverslips by combing and total DNA was stained in red with an anti-DNA antibody. The replicative labels were revealed in blue (IdU) and green (CldU) using appropriate antibodies. The blue-to-green transitions show the position and orientation of mobile forks at the time CldU was added (Figure 1A).

Replication fork velocities were determined by measuring the length of CldU or IdU tracts that could be unambiguously assigned to the progression of a single fork during an entire 20 min labeling interval. Fork velocities were narrowly distributed around a mean of 0.68 kb/min, with almost no values >2 kb/min, and did not change throughout S phase (Figure 1B).

The global density of replication forks (total number of forks divided by total length of DNA examined, corrected for contamination by non-replicating G1 or G2/M cells and for replicated genome fraction; see Material and Methods) increased through S phase (from 2.64 to 3.88, 4.55 and 5.4 forks per Mb in S1, S2, S3 and S4, respectively; Table 2). The measured replication fork densities and velocities were used to calculate the time required to duplicate the entire genome (see Material and Methods). The result (6 h 18 min) was consistent with the length of S phase independently measured from the cell doubling time and the fraction of the sorted cells in S phase (22 h×1/3 = 7 h 20 min), corroborating the fork density and velocity measurements.

**Table 2:** Statistical analysis of the parameters of DNA replication determined by DNA combing.

	S1	S2	S3	S4	ALL
**Fork velocities (kb/min)**					
Number of Values	238	376	250	258	1122
Median	0.53	0.56	0.48	0.55	0.54
Mean	0.64	0.68	0.67	0.72	0.68
SEM	0.03	0.02	0.04	0.04	0.02
**Global fork density (Forks/Mb)**					
Number of forks	82	152	190	182	606
DNA length Mb (corrected)	31.03	39.20	41.79	33.69	145.71
Forks/Mb	2.64	3.88	4.55	5.40	4.16
**Fork-to-fork distances (kb)**					
Number of Values	152	304	293	365	1114
Median	13	12	11	11	11
Mean	22	19	19	17	19
SEM	1.85	1.13	1.57	0.93	0.65
**Inter-origin distances (kb)**					
Number of Values	110	175	138	162	585
Median	28	31	30	28	30
Mean	35	36	42	36	37
SEM	2.68	1.90	2.9	2.13	1.20
**Inter-termini distances (kb)**					
Number of Values	98	146	108	130	482
Median	29	25	29	29	28
Mean	33	32	39	36	35
SEM	2.54	2.08	3.00	2.29	1.23
**Number of forks per fork-containing fiber**					
Number of forks	266	502	444	523	1735
Number of fibers	114	198	151	158	621
Median	2.0	2.0	2.0	2.0	2.0
Mean	2.33	2.54	2.94	3.31	2.79
SEM	0.10	0.08	0.09	0.10	0.05
**IdU-labeled fraction of fork-containing fibers (%)**					
Number of fibers	202	281	225	203	911
Median	24.2	39.5	37.8	48.4	36.9
Mean	30.1	44.3	41.9	52.0	42.3
SEM	1,38	1.48	1.79	1.95	0.86

The inverse of the global fork density is the global fork-to-fork distance (FTFD). The global FTFD decreased from 379 kb to 258, 220 and 185 kb in S1, S2, S3 and S4, respectively. However, the local FTFDs measured on single DNA fibers containing forks were much smaller (mean ∼19 kb) and did not decrease so much during S phase (from 22.0 kb in S1 to 17.0 kb in S4; Figure 1C; Table 2). Furthermore, the mean intra-fiber inter-origin distances and inter-termini distances were commensurate with the intra-fiber FTFDs, i.e. both were in the 35–42 kb range at all stages of S phase (Figure 1D, E; Table 2). Thus, replicons were much shorter than global FTFDs would suggest. The discrepancy between local and global FTFDs might be attributed to the finite fiber size, which prevents measurement of large FTFDs, but actually results from the fact that origins are activated as clusters that fire at different times in S phase. Thus, only 10–20% of all fibers showed replication forks at any stage of S phase but among these many showed several forks (Figure 1F). To assess the clustering of replication forks, we compared the distribution of the number of forks per fiber with that generated in a simulation that assumed random initiation and a fiber size distribution and global fork density identical to the experimental samples. The observed distributions were significantly (*P*<10^−4^) different from the simulation, with a lack of fibers with one fork (whole S-phase average, 6.3% vs. 10.6%) and an excess of fibers with ≥2 forks (8.5% vs. 2.2%). This demonstrates a clustering of origin firing.

We next examined whether the global fork density increased because more origin clusters fired or because more origins per cluster fired during S phase. We found that the number of forks per fork-containing fiber (2.33, 2.54, 2.94 and 3.31 forks per fiber in S1, S2, S3, and S4, respectively; Figure 1F; Table 2), and the IdU-labeled fraction of fork-containing fibers (30.1% , 44.3%, 41.9% and 52.0%; Figure 1G; Table 2) increased throughout S phase. Thus, more origins per cluster fired as S phase progressed. The distances between origin clusters are generally too large to be measured, because they exceed the mean fiber size. Although such distances cannot be individually measured, their mean can be computed from the statistics of fibers with and without forks (by dividing the total length of DNA minus the sum of intra-fiber FTFDs by the number of fork-containing fibers, assuming at most one cluster per fiber). Note that intercluster segments mainly consist in unreplicated DNA in early S phase and already replicated DNA in late S phase and that the total DNA length used in our calculations is corrected for the extent of DNA replication (see Material and Methods). The mean intercluster distance decreased from 772 kb in S1 to 484, 465 and 501 kb in S2, S3 and S4. Thus, inter-cluster distances were reduced as S phase progressed from S1 to S2 but did not change thereafter. This reduction was too large to be explained by the increase in cluster size. Therefore, the number of active clusters increased from S1 to S2.

To further evaluate the tightness of origin synchrony we reasoned that the consecutive IdU/CldU labeling scheme allows us to distinguish origins that fired before (type 1) or after (type 2) CldU addition. Type 1 origins are flanked by two divergent blue-to-green transitions whereas type 2 origins give rise to doubly-labeled, isolated tracks. For example, most origins shown on Figure 1A fired before CldU addition but the leftmost one fired after CldU addition. We first noticed that when inter-origin distances were plotted separately for type 1 and type 2 origins (not shown), their distributions were not markedly different from those shown on Figure 1D, where all origins were taken into account. This suggested that type 1 and type 2 origins were not frequently interspersed with each other. We then selected fibers containing more than one origin and found that adjacent origins were significantly more frequently of the same type than if randomly interspersed (254 type1/type1; 60 type2/type2; 99 type1/type2; *P*<10^−4^, chi-square test of homogeneity). Thus, adjacent origins tended to fire within 20 min of each other. Together these observations suggested that a wave of initiations propagates on the DNA molecule.

In conclusion, DNA combing showed that in HeLa cells i) replication origins are spaced at mean ∼40 kb intervals; ii) adjacent origins fire within 20 min of each other, resulting in a spatial clustering of replication forks; iii) replication fork velocity (∼0.68 kb/min) does not change during S phase; iv) the global fork density increases during S phase, because more replicon clusters and more origins within clusters become active as S phase progresses. Therefore, the global rate of DNA replication increases during S phase due to increasing origin synchrony.

### Comparison with replication parameters found in other studies

Our conclusion that fork speed is constant through S phase contrasts with earlier reports of changes in fork speed during S phase [47], [48]. However, in these studies, chemicals or serum starvation were used to synchronize cells, which may affect nucleotide pools and replication fork progression, whereas the retroactive (FACS) synchronization we used does not perturb the cell cycle. Furthermore, these studies used less precise techniques than DNA combing to spread DNA fibers, and some of the track length changes interpreted as changes in fork progression may in fact have resulted from changes in the synchrony of adjacent origins and consequent merging of forks. We have minimized such potential artifacts thanks to the use of two short, consecutive labeling pulses and the better resolution of DNA combing, which allowed us to demonstrate an increase in adjacent origin synchrony during S phase.

The fork speed (∼0.7 kb/min) and interorigin distance (∼40 kb) we found are somewhat, though not much, lower than usually reported in other human cell lines (typically 1.0–2.0 kb/min and 100–200 kb) [49]. Small interorigin distances (57 kb) and slow forks (0.37 kb/min) have also been found by DNA combing in K562 leukemic cells [50]. Small interorigin distances were also reported using another DNA fiber technique both in U2OS osteocarcinoma cells (50 kb) and in nontransformed MRC5 cells (42.5 kb) [51]. More intriguingly, our estimates also differ from those reported by other investigators in HeLa cells (fork rates of 0.59–1.37 kb/min [52] and 1.7±0.3 kb/min [27] and interorigin distances of 144±66 kb [27]). In yet another HeLa clone (data not shown) we observed slightly larger replicons (50 kb) and faster forks (1.0 kb /min) than in this work. Thus, clonal variation as well as differences in labeling scheme, DNA fiber technique and track choice probably explain these differences.

Such clonal variation is consistent with the possibility that the cancerous nature and genetic or epigenetic instability of HeLa cells influence origin activity and fork progression and their response to a number of physiological and pathological stimuli [53]–[55]. Indeed, recent work showed that forced expression of oncogenes in primary keratinocytes can slow down replication fork progression and trigger activation of dormant origins due to decreased nucleotide pools [56]. However, in another study, no change in origin spacing and fork velocity could be observed between primary keratinocytes and a keratinocyte-derived tumour cell line [57]. Thus, it remains possible that the fork speed and origin spacing observed in HeLa cells just reflect some physiological tissue variation range.

### Whole-genome replication timing profile of HeLa cells

We generated a high-resolution, genome-wide replication timing profile in HeLa cells as described previously [12] with minor modifications detailed in the Material and Methods. Briefly, HeLa cells were pulsed with BrdU, sorted into four temporal compartments of the S phase and nascent DNA was immunoprecipitated with anti-BrdU antibodies and sequenced using the Illumina technology to yield a total of 50 million reads that mapped uniquely to the human genome sequence. The abundance of sequence reads along the genome was computed every 10 kb in a 100 kb sliding window in each S phase compartment allowing to cover 90% of the genome. The resulting profile was used to compute in each window the fraction of the S phase at which 50% of the DNA was replicated (S_50_, [12]). Using the FACS DNA fluorescence histogram to extract the proportion of cells at different stages in S phase and the temporal profile of the rate of DNA synthesis, we calculated the profile of DNA content as a function of the time spent by a cell in S phase. The S_50_ values were then used to deduce the time (TR_50_) at which a defined genome region had replicated in 50% of the cells (see Material and Methods). A biological replicate showed excellent reproducibility of the TR_50_ (Pearson *R* = 0.97, *P*<10^−16^). The average of the two TR_50_ determinations was used for subsequent analyses.

**Figure 2:**
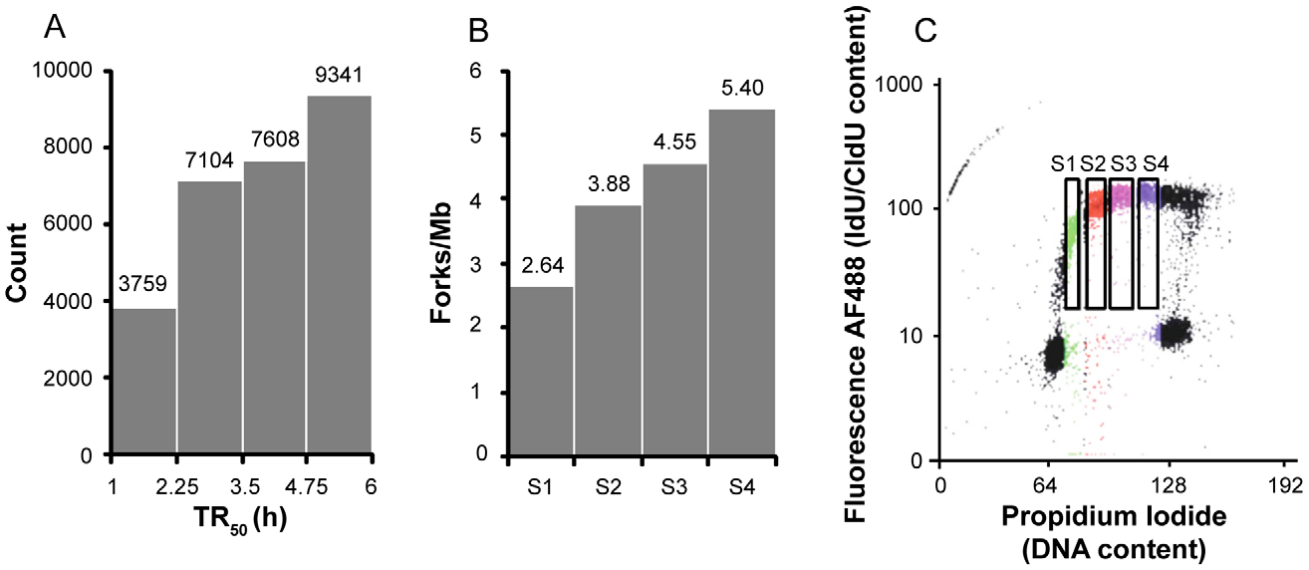
The global rate of DNA replication increases during S phase in HeLa cells. (**A**) Histogram of replication timing values (TR_50_, hours) in the whole genome. (**B**) Histogram of global fork densities in S1, S2, S3 and S4 as determined by DNA combing. (**C**) Flow cytometry profile of cells pulsed labeled with 25 µM IdU/CldU for 20/20 min. IdU/CldU was stained with fluorescent antibodies. Fluorescence was plotted against total DNA content. Cells in S1, S2, S3 and S4 appear respectively in green, red, purple and blue. Four windows indicate cells in S1–S4 and labeled with IdU/CldU.

The genome-wide TR_50_ histogram (Figure 2A) showed a continuum of replication times with no dearth of replicating regions in mid-S phase and an increasing number of replicating regions during S phase. This is consistent with the increase in global fork density observed by DNA combing (Figure 2B) and the one of global rate DNA replication observed by flow cytometry analysis (Figure 2C). This is also consistent with the observed dip in the FACS histogram of DNA content from S1 to S3, due to cells moving faster through this DNA content (Figure S1A). The expected dip in S4 was not observed but this was due to the spreading of the adjacent G2 peak. The continuous dip from S1 to S4 was indeed visible in the post sort control, where the DNA content of sorted S1 to S4 cells was reexamined in a second round of sorting (Figure S1B).

### Multiscale analysis of apparent replication speeds in HeLa cells

**Figure 3:**
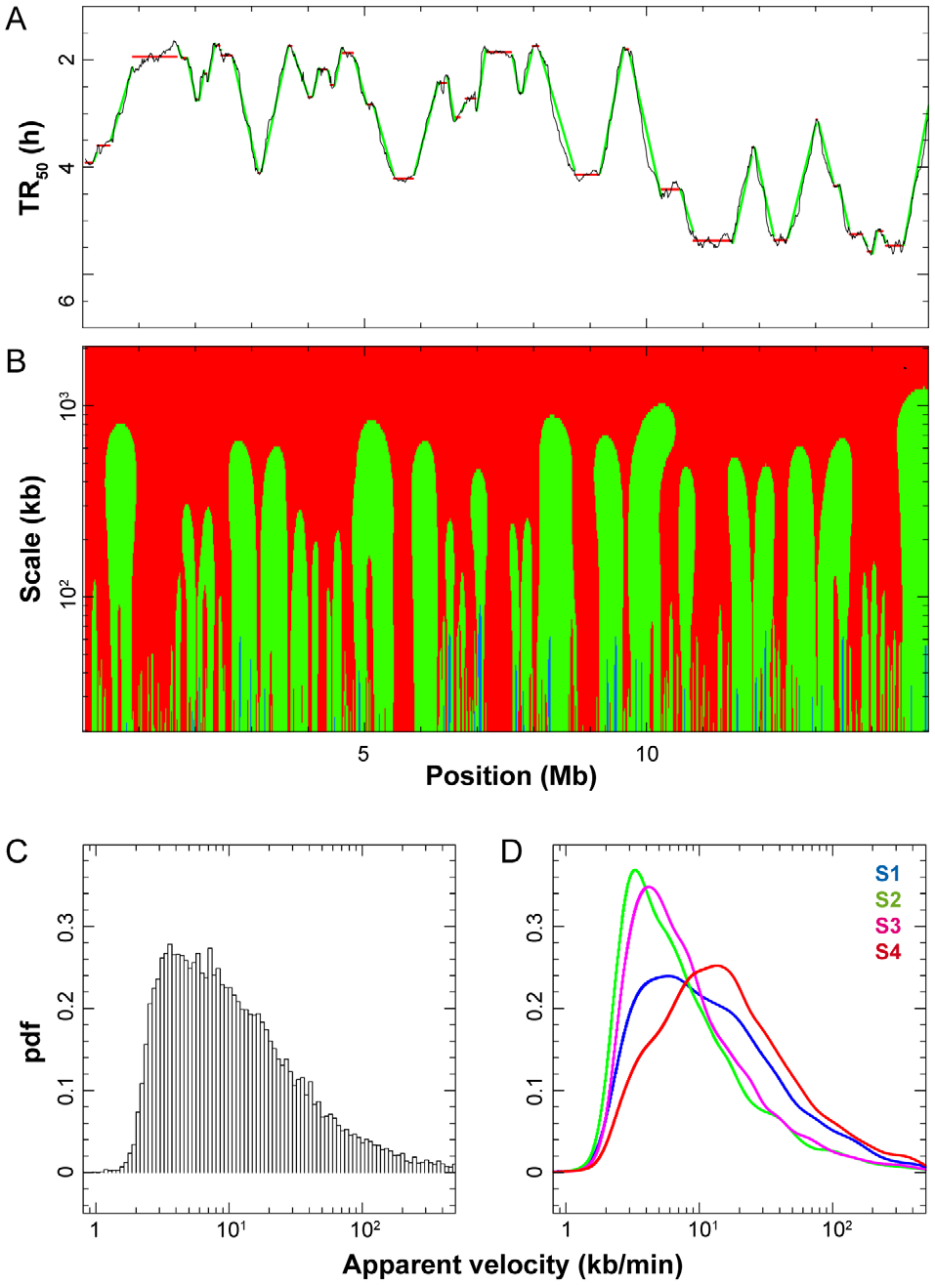
Replication timing profiles segmented in CTRs/TTRs and multiscale analysis of apparent replication speeds in HeLa cells. (**A**) Profile of replication timing (TR_50_, hours) along a 15 Mb segment of chromosome 17. Small TR_50_ values correspond to early replicating regions; large TR_50_ values correspond to late replicating regions. The replication timing profile was segmented into regions that replicate at apparent speed >10 kb/min (CTRs: Constant Timing Regions, red horizontal lines) and <10 kb/min (TTRs: Timing Transition Regions, green oblique lines) at scale 100 kb. (**B**) Multiscale analysis of apparent replication speeds along the same chromosome segment. Replication speeds determined by wavelet transform analysis (see Material and Methods) at scales indicated on the y-axis are shown in three colors (blue, <2 kb/min; green, from 2 to 10 kb/min; red, >10 kb/min). (**C**) Distribution of apparent replication speed at the 100 kb scale in the whole genome (pdf: probability density function). (**D**) Distribution of apparent replication speeds in the four temporal compartments of S phase: S1, S2, S3 and S4 (respectively: blue, green, pink, and red curves).

The TR_50_ profile along the genome showed a landscape of peaks and valleys interspersed with flat domains of uniform replication time (Figure 3A shows an exemplary 15 Mb chromosomal segment; see Figure S2 for a whole-genome profile). The slope of replication timing profiles has often been taken as a measure of replication fork velocity. However, since replication timing profiles are population averages, this is only true for regions in which forks progress in the same direction in all cells. Here we demonstrate (see Material and Methods), as first proposed by de Moura et al [22] in a recent analysis of yeast replication timing profiles, that the derivative of the replication timing, *dt/dx*, depends not only on the fork speed, *v*, but also on the local proportion of rightward (*R*) and leftward (*L*) moving forks in the cell population, such that *dt/dx* = *(R−L)/v*. The apparent replication speed is defined here as the inverse of this derivative, *dx/dt*. Note that the equality *dx/dt = v/(R−L)* implies that the sign of the apparent replication speed indicates the predominant direction of replication progression and that in flat domains of uniform replication time (infinite apparent replication speed), forks move equally in both directions.

We performed a multiscale analysis of the apparent replication speed genome wide, using the continuous wavelet transform, a robust method to obtain a well defined and numerically stable measurement of the local slope of the timing profile at any scale of observation (Figure 3B; Figure S2). The replication speed modulus, |*dx/dt*|, critically depended on the measured segment scale *dx*. At very large scales (>2 Mb), the entirety of the genome appeared to replicate at >10 kb/min. At smaller scales, a differentiation of the genome into smaller and slower replicating segments was observed, revealing finer details of the replication profile. The landscape of replication speeds stabilized below the 100 kb scale, as expected from the spatial resolution of the profile. At this scale, a broad distribution of replication speeds was observed in the HeLa cell genome (Figure 3C), with 1% of 100 kb segments replicating at an apparent speed ≤2 kb/min, 53% in the 2–10 kb/min range, and 46% at >10 kb/min. We noted that the speed distribution was shifted toward higher speeds for S1 and S4 compared to S2 and S3 fractions (Figure 3D).

The observed range of apparent replication speeds cannot be explained by the range of single fork velocities measured by DNA combing in the same cells. The mean and maximum fork velocities are 0.68 kb/min and 2.0 kb/min, whereas 99% of the genome replicates at an apparent speed >2 kb/min. The possibility that regions with the slowest apparent replication speed are specifically replicated by the fastest forks seems unlikely since fork velocities at single loci usually show the same degree of heterogeneity as in the bulk genome (e.g. [58], and see below our data on the IGH locus). These results imply that in HeLa cells, |*R−L*|<1, i.e. replication forks move in both directions, in most of the genome and that the proportion of right and left forks varies widely along the genome. There is a complete gradation between regions where forks progress predominantly (if not exclusively) in one direction (steep timing gradient, apparent speed ≤*v_max_*), and regions where they progress equally in both directions (flat timing gradient, high apparent speed). The apparent speeds must therefore reflect the statistics of origin activation around and within the timing gradients. Essentially similar results were obtained for several other cell lines (see below).

### Segmentation of the genome into CTRs and TTRs

**Figure 4:**
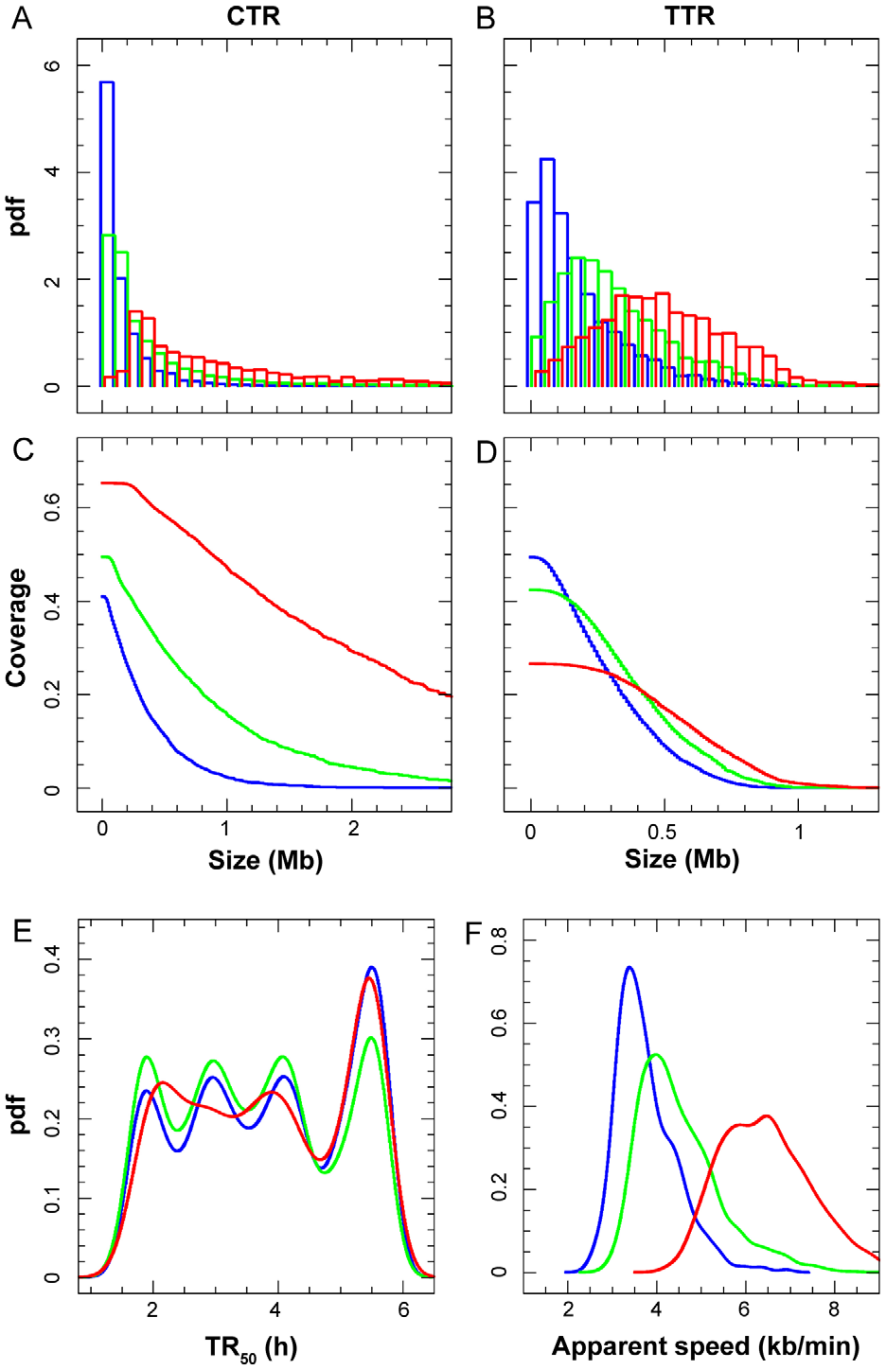
Characteristics of CTRs and TTRs. Blue, green and red curves respectively depict parameters defined at scales 100, 200 and 500 kb. (**A, B**) Size distribution of CTRs and TTRs (pdf: probability density function). (**C, D**) Genome coverage of CTRs and TTRs of length>Size (in Mb). (**E**) Mean TR_50_ of CTRs. (**F**) Apparent replication speeds of TTRs (pdf: probability density function). Note that different scales are used on the X-axis for CTRs and TTRs because their sizes are different.

To address the mechanism by which different proportions of rightward and leftward moving forks are established in different parts of the genome in HeLa cells, we segmented the whole genome into constant timing regions (CTRs) replicating at >10 kb/min and timing transition regions (TTRs) replicating at ≤10 kb/min and analyzed them separately. Figure 4A–F shows the size distribution, genome coverage, TR_50_ and apparent replication speed of CTRs and TTRs defined at 100 kb (blue), 200 kb (green) and 500 kb (red) scales. At the 100 kb scale, the whole genome was segmented into 7548 CTRs and 7504 TTRs (Figure 3A; Figure S2). All CTRs were ≤2 Mb and 53.8%<100 kb (Figure 4A), with CTRs>100 kb covering 34.2% of the genome (Figure 4C). All TTRs were ≤900 kb and 64.4%<200 kb (Figure 4B), with TTRs>200 kb covering 32.4% of the genome (Figure 4D). At larger scales, as expected, the mean size of both CTRs and TTRs increased and the genome fraction covered by CTRs increased at the expense of TTRs. The TR_50_ distribution of CTRs was relatively insensitive to scale (Figure 4E) and was similar to that of the whole genome, but the apparent replication speed of TTRs increased with scale (Figure 4F). The small oscillations in the TR_50_ distribution of CTRs are an artifact of the finite number of S phase fractions, which we have not attempted to correct. The proportion of CTRs was higher in S1 (48%) and S4 (56%) than in S2 (28%) and S3 (32%), consistent with the fastest distribution of speeds in these two S phase compartments (Figure 3D).

### Replication mode of Constant Timing Regions

One possible mechanism for explaining why an equal proportion of rightward and leftward moving forks replicate a CTR is that it does not contain origins and is passively replicated from an outside origin that is activated equally often on its right or its left side (Figure S3A). Given a mean fork velocity of 0.68 kb/min (40 kb/h) this mechanism could only apply to short enough CTRs (<300 kb) to replicate within a 7–8 h S phase in HeLa cells. At the 100 kb scale, CTRs<300 kb and >300 kb cover 19.7% and 21.2% of the genome, respectively (Figure 4C). This mechanism predicts that i) the edges of small CTRs would replicate asynchronously in non-adjacent S-phase compartments whereas their centers would replicate synchronously in mid-S phase; ii) that small CTRs lying between 150 and 300 kb would replicate rather in mid-S phase. A previous study of Hela cells replication timing determined that about 20% of the ENCODE regions present a pan-S replication profile [59]. However, we reported that in HeLa cells only 7.4% of all genomic sequences replicate with such a pan-S profile [12]. Although a significant correlation was observed between these two studies (Pearson, *R* = 0.77, *P*<10^−15^), the differences may result from the use of microarray hybridization and cell synchronisation by drug treatment in the first study vs. massive sequencing and no drug treatment in our study. Furthermore, we have found that the TR_50_ distribution of CTRs spans the entire S phase whatever their size (data not shown), inconsistent with the mechanism proposed above.

Alternatively, CTRs might consist of regions in which multiple origins are synchronously activated (Figure S3B). This mechanism would result in an equal number of forks moving in both directions whatever the size and the replication time of the CTR. The fact that the TR_50_ distribution of CTRs spans the entire S phase whatever their size suggests that all long CTRs and most small CTRs replicate during defined intervals of S phase by synchronous firing of multiple replication origins. The small-scale changes in fork polarity around individual origins are not seen due to the small replicon size and/or to the use of different potential origins in different cells, which effectively smooth replication timing gradients across multiple replicons.

### Replication mode of Timing Transition Regions

**Figure 5:**
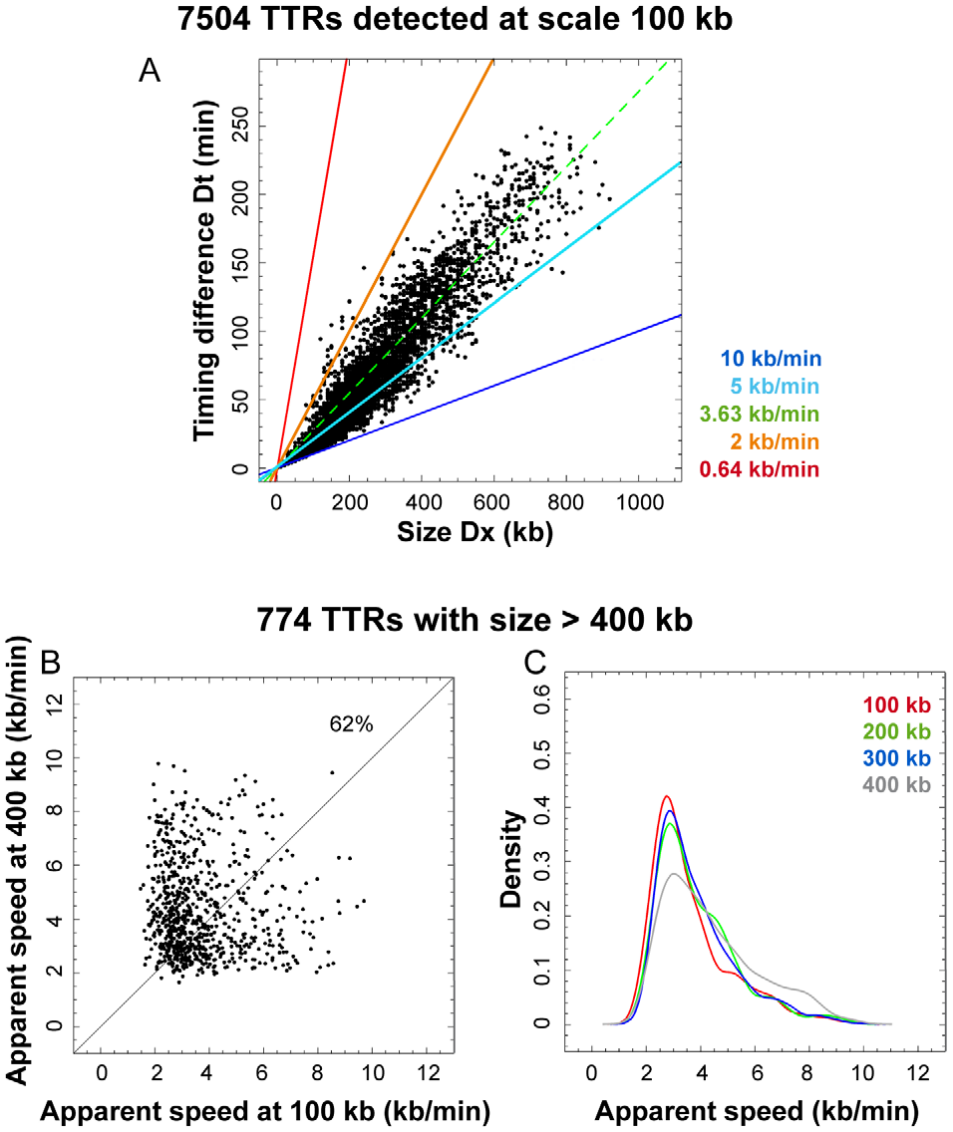
Replication Speeds along TTRs. (**A**) Size and replication time of individual TTR. The time difference, Dt, between the early and the late side of each TTR is plotted along its length, Dx, for each of the 7504 TTRs (open circles). By definition, the maximum replication speed of TTRs is 10 kb/min (dark blue line). The mean apparent replication speed, Dx/Dt, is 3.63 kb/min (green dashed line). The mean (v = 0.64 kb/min, red line) and maximum (v = 2 kb/min, orange line) velocities of single forks measured by DNA combing are indicated. Only 24 TTRs lie between the orange line and the vertical axis. (**B**) Evolution of apparent replication speed along the 774 TTRs>400 kb. The apparent speed measured at the distance D4 = 400 kb from early edge of each TTR is plotted against the apparent speed measured at the distance D1 = 100 kb. Replication accelerates for 62% of TTRs. (**C**) Distribution of apparent replication speeds along the 774 TTRs>400 kb. The apparent speed has been measured at different distances D1–D4 from early edge of each TTR: D1 = 100 kb, red curve; D2 = 200 kb, green curve; D3 = 300 kb, blue curve; D4 = 400 kb, grey curve.

Our demonstration that the apparent replication speed is equal to *v/(R−L)* (assuming that *v* is locally constant), implies that in TTRs replication forks move predominantly but perhaps not exclusively in one direction. To further investigate this we analyzed TTRs individually. We found that the temporal transitions were directly proportional to the length of the TTRs (Figure 5A). Even at the smallest scale analyzed (100 kb), only 24 out of these 7504 transitions were compatible with the progression of a single fork even at maximum rate (*v_max_* = 2 kb/min) and together they only covered 0.13% of the genome. None of them was >250 kb, as expected from the maximum distance that a single fork can travel during S phase. Therefore, systematic unidirectional replication of large regions is not observed in HeLa cells. Replication forks instead appear to move in both a major and a minor direction in most TTRs. One potential explanation is that some TTRs support no internal initiation and are replicated from alternative origins located on either side of the TTR and used in unequal fractions of the cells (Figure S3C). As discussed for CTRs, this mechanism could only apply to TTRs<300 kb and would predict asynchronous replication of their edges, for which we did not find convincing evidence.

Alternatively, multiple origins could fire in a progressive manner along the TTRs (Figure S3D). The mean replication progression rate along TTRs was 3.63 kb/min, 5 times the mean progression rate of single forks (Figure 5A). This suggests that on average 2–3 adjacent replicons simultaneously operated along the gradient or, in other words, that on average adjacent origins spaced at ∼36 kb intervals were consecutively activated at ∼10 min intervals. Faster (slower) apparent speeds may result from shorter (larger) space and/or time intervals between adjacent initiations. This mechanism not only explains why replication progresses faster than single forks in TTRs but also why a higher proportion of forks move downstream than upstream the gradient, because when a new origin fires, the upstream moving fork will rapidly merge with the converging fork emanating from the upstream origin, whereas the downstream moving fork will progress for some distance before the next downstream origin fires. According to this mechanism, the faster distribution of speeds in late S phase is due to an increased synchrony of origin firings, consistent with the DNA combing results.

### Replication accelerates along the TTRs

A visual inspection of the replication timing profile suggested that the slope of a large fraction of the TTRs tended to flatten with distance from their early edge. To asses this point, we selected TTRs>400 kb and measured the apparent replication speed at different positions along the slope. It was found that the apparent replication speed increased for about two thirds of the TTRs (Figure 5B). Furthermore, the distribution of apparent replication speeds along the TTRs was shifted to higher values at increasing distances from the early edge of the TTR (Figure 5C). These results suggest that forks move more and more in both directions along the TTRs as S phase progresses. These results are consistent with the DNA combing data showing that origins fire in an increasingly synchronous manner as S phase progresses.

### Multiscale analysis of apparent replication speeds in other cell lines

**Figure 6:**
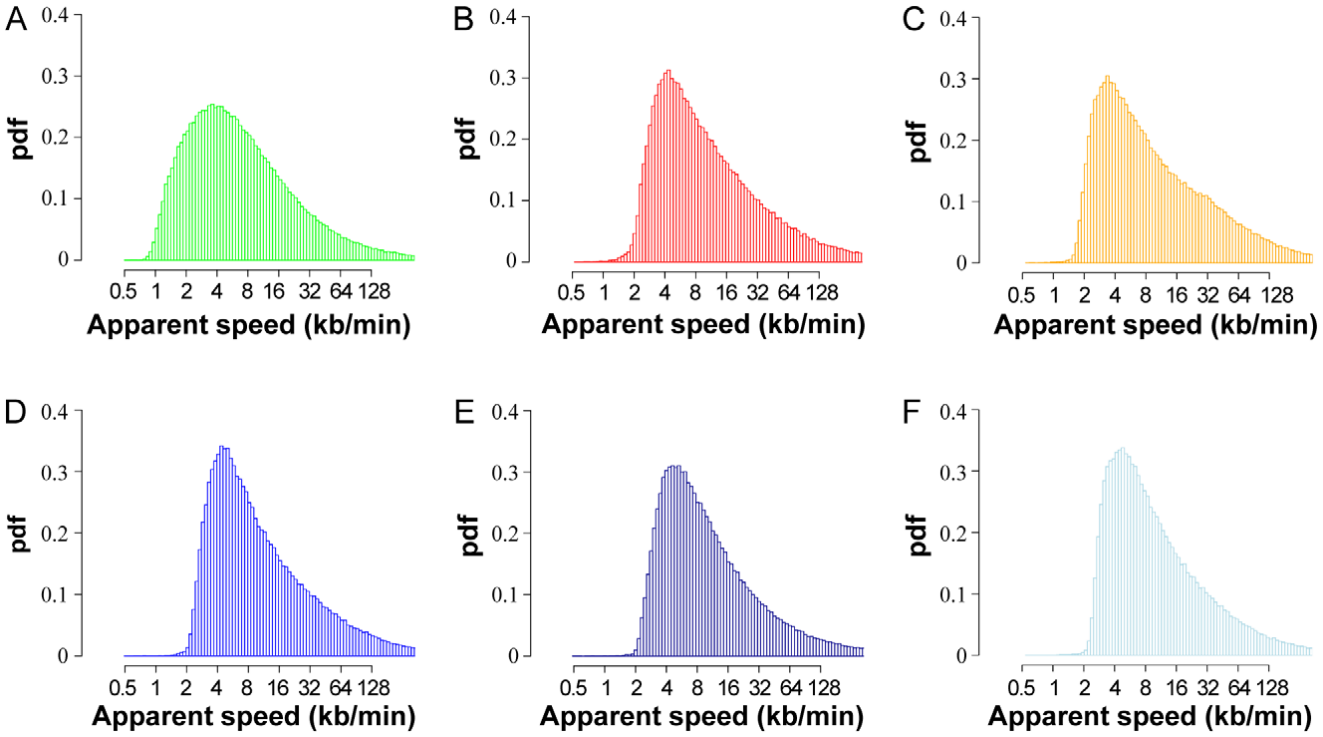
Analysis of apparent replication speeds in multiple cell types. The distribution of apparent replication speeds at the 100 kb scale in the whole genome was determined for (**A**) BG02, a human embryonic stem cell line; (**B**) K562, a chronic myelogenous leukemia cell line; (**C**) BJ, normal fibroblasts; (**D**) GM06990, (**E**) TL010, and (**F**) H0287, lymphoblastoid cell lines, as described for HeLa cells in Figure 3 legend.

The recent availability of high-resolution replication timing data in six other human cell lines (BG02, a human embryonic stem cell line; K562, a chronic myelogenous leukemia cell line; BJ, normal fibroblasts; GM06990, TL010, and H0287, lymphoblastoid cell lines) [13] prompted us to carry out a similar multiscale analysis of their apparent replication speeds. As shown in Figure 6, the distributions of replication speeds at the 100 kb scale were quite similar to HeLa cells with 3% (BJ) and <1% (other cells) of apparent speeds ≤2 kb/min, except for BG02 cells where a higher proportion of speeds ≤2 kb/min was observed (14.3%). Note that in the absence of published measurements of S phase length in these cell lines we have assumed a uniform S phase length of 8 h, typical of most mammalian cell lines. These distributions would be shifted toward proportionately faster (slower) speeds if S phase turned out to be shorter (longer). It is interesting to note that apparent speed distributions were much more similar among cell lines than single fork speeds and, by inference, origin activation patterns. This is consistent with a number of observations suggesting that replication timing is a more conserved feature among cell types than replication origin distribution [60]. The difference between BG02 and the other cell lines presumably reflects the previously described smaller replication domain size and higher density of timing transition regions in embryonic stem cells than in differentiated cells [8], [14].

As in HeLa cells, the observed ranges of apparent replication speeds in these cells cannot be explained by the range of single fork velocities measured by DNA combing in identical or comparable cells. In K562 cells, mean and max fork velocities are 0.37 kb/min and 1.0 kb/min [50] whereas >99.9% of the genome replicates at apparent speed >1.0 kb/min. To our knowledge, replication fork velocities have not been measured in the five other cell lines. However, mean and max fork velocities have been estimated to 1.73 and 2.9 kb/min in MRC5 fibroblasts (M. Debatisse, pers. comm.) and to 2.06 and 4.4 kb/min in JEFF lymphoblastoid cells [58]. Taking these values as reasonable estimates for BJ fibroblasts and for GM06990, TL010, and H0287 lymphoblastoid cells, respectively, it appears that 99.5–99.8% and 76–85% of the genome replicate faster than the mean and max fork velocity, respectively, in all those cell lines. Furthermore, mean fork velocities of 1.53–2.49 kb/min have been found in H9 and H14 embryonic stem cells [61]. Assuming that mean and max velocity in BG02 embryonic stem cells are 2.0 kb/min and 4.0 kb/min, respectively, we find that 85.7% and 61.9% of the genome replicate faster than these respective speeds. Thus, a higher proportion of the genome replicates at an apparent speed compatible with unidirectional progression of a single fork in BG02 cells.

**Figure 7:**
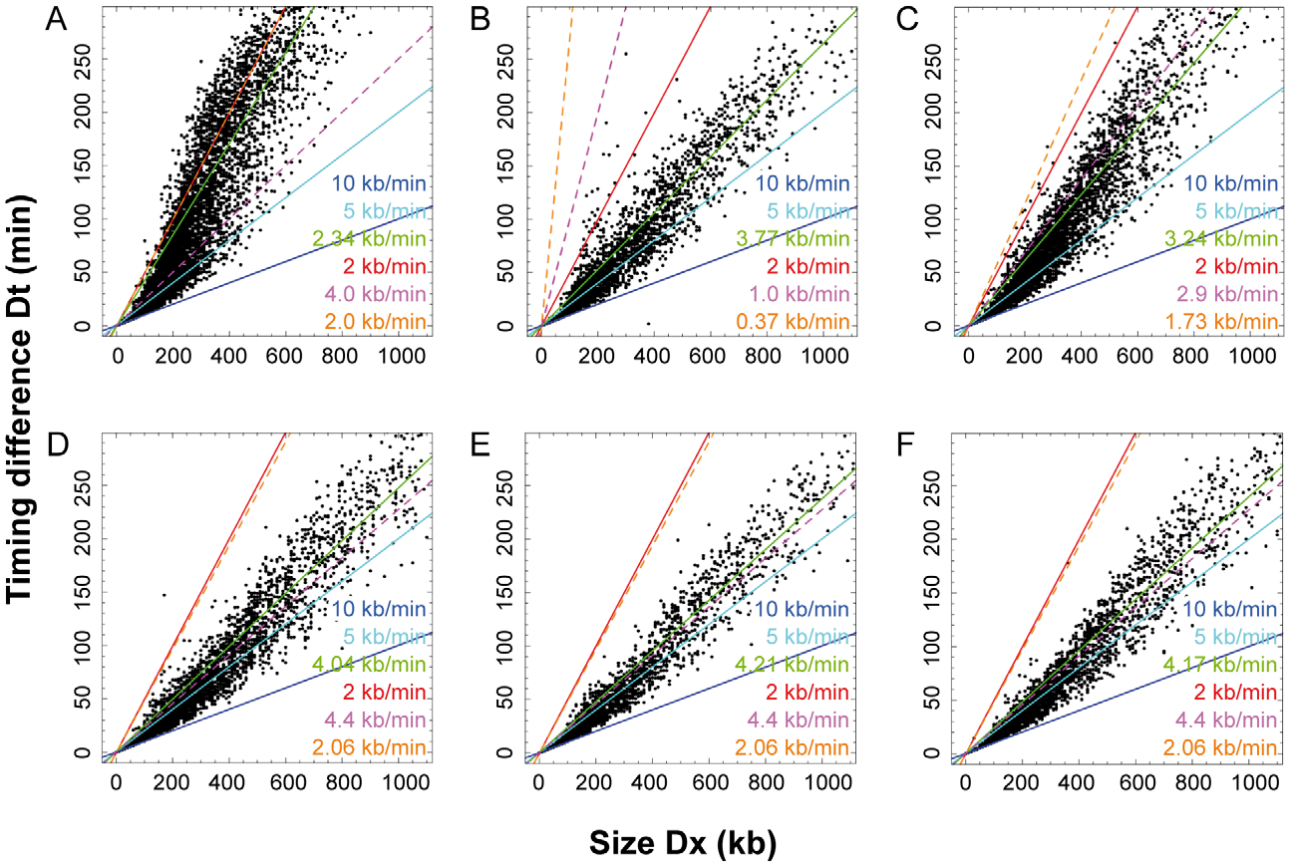
Size and replication time of individual TTRs in multiple cell types. The time difference, Dt, between the early and the late side of each TTR detected at scale 100 kb is plotted along its length, Dx, for each of the TTRs (dots) for (**A**) BG02, a human embryonic stem cell line (7657 TTRs); (**B**) K562, a chronic myelogenous leukemia cell line (3638 TTRs); (**C**) BJ, normal fibroblasts (5266 TTRs); (**D**) GM06990 (4017 TTRs), (**E**) TL010, (2492 TTRs) and (**F**) H0287 (3237 TTRs), lymphoblastoid cell lines. By definition, the maximum replication speed of TTRs is 10 kb/min (dark blue line). Lines corresponding to 5 kb/min (turquoise) and 2 kb/min are also provided as a guide to the eye. The mean apparent replication speed, Dx/Dt, is indicated by a green line (BG02, 2.34 kb/min; K562, 3.77 kb/min; BJ, 3.24 kb/min; GM06990, 4.04 kb/min; TL010, 4.21 kb/min; H0287, 4.17 kb/min). The mean (orange dashed line) and maximum (purple dashed line) velocities of single forks measured by DNA combing in identical or comparable cell lines (see text) are indicated. Except for BG02, practically no TTR is found between the orange dashed line and the vertical axis.

To further investigate this we analyzed the TTRs of these six cell lines individually (Figure 7). The number of TTRs is about 2-fold higher in BG02 embryonic stem cells than in the differentiated cells (numbers in Figure 7 legend). Interestingly, a large fraction of the BG02 TTRs replicated at an apparent speed compatible with unidirectional progression of a single fork (Figure 7 A; average apparent speed 2.34 kb/min, mean fork velocity 2.0 kb/min). In all the other cell lines (Figure 7 B–F), however, the TTRs replicated faster than in BG02 (average apparent speed ranging from 3.24 kb/min to 4.21 kb/min, green lines), and faster than the mean fork velocity (compare dots with orange dashed lines). The discrepancy was most pronounced in K562 cells (Figure 7 B), where no TTR replicated slower than the fastest single forks (*v_max_* = 1.0 kb/min, purple dashed line). In BJ fibroblasts (Figure 7 C) and in the three lymphoblastoïd cell lines (Figure 7 D–F), however, many TTRs replicated at an intermediate speed between the mean and max fork velocity (orange and purple dashed lines, respectively). The possibility that the slowest TTRs are specifically replicated by the fastest forks cannot be formally discounted but seems unlikely, as explained above. Therefore, most of the TTRs in these five cell lines replicate faster than by a single unidirectional fork. In other words, internal initiation in TTRs is more frequent in differentiated cells than in BG02 stem cells. Furthermore, in cancerous cells K562 and HeLa, replication forks progress more slowly and this likely triggers additional origin activation in TTRs.

Importantly, if domains of constant replication time were separated by timing transition regions of uniform and slow replication speed [8], [14], a biphasic distribution of apparent replication speeds should have been observed. This was not the case in any of the cell lines investigated. We found instead that the apparent replication speed, *dx/dt*, has a continuous and wide-range distribution significantly faster than the known range of fork velocities, *v*, in the vast majority of the genome. This implies that in all these cell lines, the statistics of origin activation creates throughout the genome a complete gradation in the predominance with which forks move in a preferred direction.

### Comparison with previous genome-wide replication timing studies

Our findings appear to contradict earlier views of genome-wide replication timing in human and mouse cells, which proposed a strict dichotomy between large (0.2–2.0 Mb) CTRs containing multiple synchronous origins and smaller (0.1–0.6 Mb) TTRs with slopes consistent with unidirectional replication fork progression [8], [9], [11], [14].

In the study by Desprat et al [9], the profiles were generated from the <2-fold copy number difference between S and G1 cells, which resulted in a low signal-to-noise ratio, and TTRs were defined as regions >250 kb in which the slope did not differ by more than 0.1 kb/min over their entire lengths. Such TTRs had slopes consistent with unidirectional fork progression (0.8–3.5 kb/min) but they only encompassed 5–8% of the genome. In three other studies [8], [11], [14], the profiles were generated from the abundance ratio of newly replicated DNA in different fractions of S phase and were segmented into CTRs and TTRs using a clustering algorithm. In all three cases, the resulting TTRs again only encompassed a small fraction of the genome (<10%). Hiratani et al [8] and Ryba et al [14], who used only two fractions of S phase, found slopes consistent with unidirectional fork progression (0.8–3.5 kb/min), but Farkash-Amar et al [11], who used up to seven fractions of S phase, found faster slopes (1.5–6.5 kb/min). As can be seen in Figure S3 in Hiratani et al [8], having only two S phase fractions creates an essentially biphasic distribution of replication times, an artifact that is much attenuated by the use of four to six S phase fractions (Figure 4E).

The profiles we analysed in this work were generated from four [12] or six [13] fractions of S phase, allowing us to discern replication timing differences within regions that were merged as a single replication timing domain in previous studies. Furthermore, we determined the full distributions of apparent speeds before any segmentation of the genome. These distributions were continuous, not biphasic, which implies that any segmentation in CTRs and TTRs entails a degree of arbitrariness. When we delineated CTRs and TTRs as contiguous regions which replicate faster (resp. slower) than 10 kb/min at a 100 kb scale, the genome was partitioned in two nearly equal halves. However, to obtain a set of TTRs that encompass <10% of the genome, we would need to set the threshold at ∼3 kb/min. Interestingly, the size range (0.1–0.5 Mb) and mean replication speed (2.3 kb/min) of such TTRs would be similar to those reported in the other studies, yet mostly incompatible with unidirectional fork progression given the fork speed measured by DNA combing in HeLa cells (Figure 5A, and data not shown). In none of the previous studies was the speed of replication forks directly measured on single DNA molecules in the same cells. We therefore believe that the rigid dichotomy reported in these studies overlooked the existence of a broad range of timing transition slopes, due to insufficient temporal resolution and/or to the use of a segmentation algorithm, and needs to be replaced with a more nuanced picture of DNA replication kinetics.

Although we do not exclude passive (but bidirectional) replication of TTRs<300 kb in HeLa cells, our data show that the mean replication progression rate along most of the genome is remarkably high, meaning that most TTRs are preferentially replicated by the progressive firing of multiple origins in most cells of a population. This is also the case for K562 cells. Nevertheless, we observed a higher proportion of apparent replication speeds consistent with unidirectional progression of a single fork in BG02 stem cells, and, to a smaller extent, in fibroblasts and lymphoblastoid cell lines in which replicons are longer and replication forks move faster than in HeLa cells.

### DNA combing analysis of the IGH TTR

In the study by Desprat et al [9], the notion that TTRs are originless regions that replicate by unidirectional fork progression was strongly supported by a single molecule analysis of the human IGH locus. This experiment unambiguously demonstrated that most forks progress unidirectionally in this transition region in human ES cells, in agreement with ample evidence for unidirectional replication of the homologous locus in mouse ES cells and T lymphocytes [41], [42]. This behavior is cell-type dependent, however, since abundant initiation events were detected in the same region during early and late stages of mouse B cell development [42].

**Figure 8:**
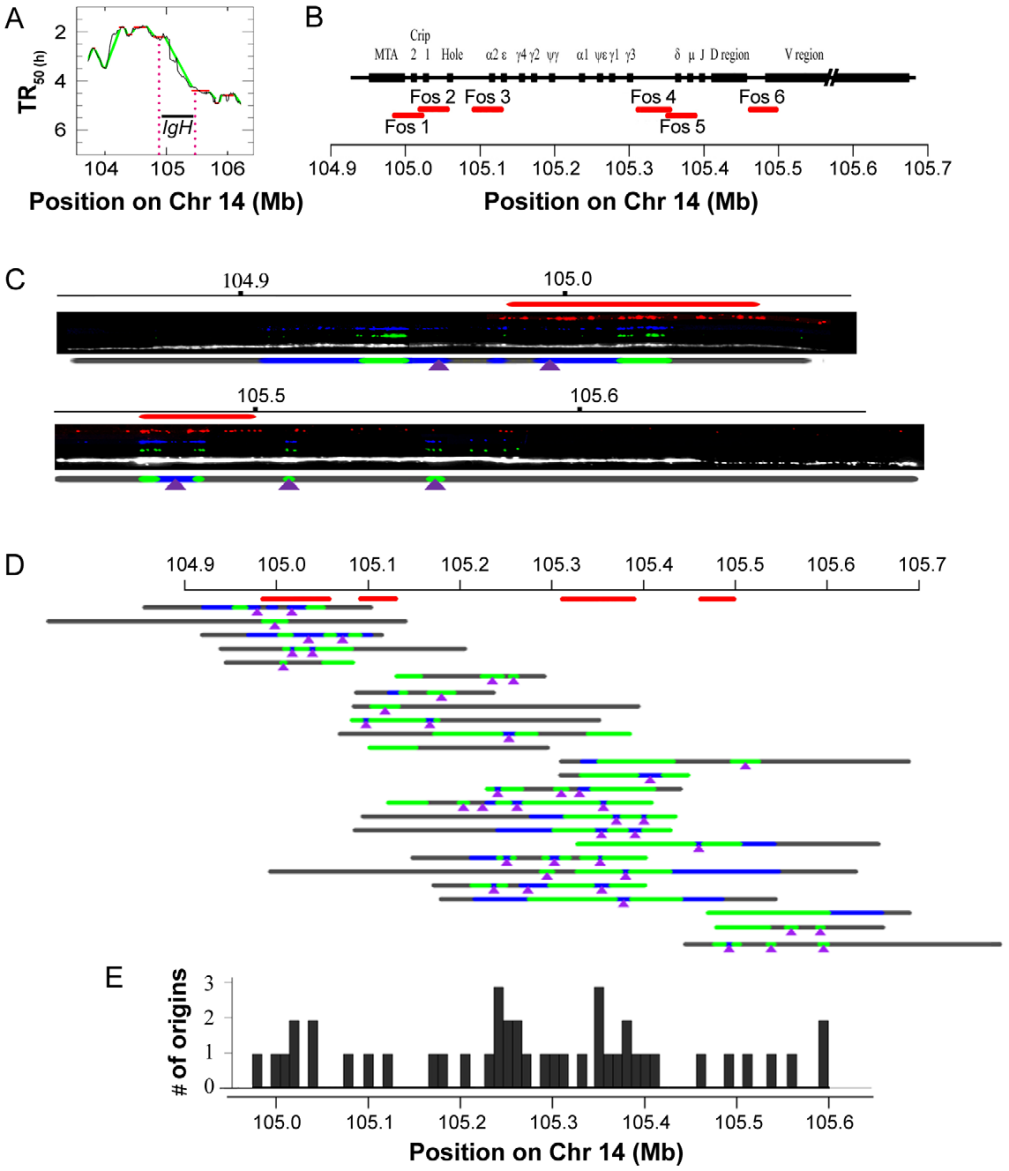
DNA combing analysis of the IGH TTR. (**A**) Replication timing profile of the IGH region and its surroundings in HeLa cells. (**B**) Map of the IGH region, position of the fosmid probes (red lines) and chromosome coordinates. Combed DNA molecules were hybridized either with Fos1-2-3 or with Fos4-5-6, allowing reliable detection and orientation of the combed IGH molecules. (**C**) Exemplary DNA molecules and interpretative diagrams showing probe hybridization (red), IdU (blue) and CldU (green) tracks and total DNA (white) and deduced origin locations (purple arrowheads). The complete set of analyzed molecules is shown on Figure S4. (**D**) Schematic representation of all replicative DNA molecules analyzed aligned along the locus using the detected hybridization patterns. Note that for one of these molecules (13^th^ line) the orientation could not be unambiguously determined and one of the two possible orientations was arbitrarily chosen. (**E**) Distribution of detected origins along the locus.

We found that in HeLa cells the IGH locus is included in a 440 kb TTR whose apparent replication speed is 3.77 kb/min, inconsistent with unidirectional replication and significantly faster than reported by Desprat et al [9] in other cells (Figure 8A). We used DNA combing to determine the replication mode of this region (Figure 8 and Figure S4). We observed 43 initiation events on 25 DNA fibers evenly spread over a >700 kb region including the three restriction fragments studied by Desprat et al [9]. Only two out of these 26 fibers were found to contain a single fork. We also found that replication fork velocities (1.48±0.21 kb/min, N = 38) and inter-origin distances (46.0±5.1 kb, N = 20) in this region were approximately similar to that of the bulk genome. These results unambiguously demonstrate that this TTR replicates by progressive activation of multiple replication origins in HeLa cells and confirm the validity of our multiscale analysis of apparent replication speeds in predicting regions that cannot replicate by unidirectional progression of a single fork. Together with the results of Desprat et al [9], they also confirm that the replication mode of the human IGH locus can change according to cell type, as previously reported for the mouse IgH locus [42]. Since HeLa cells are derived from an adenocarcinoma, they show that replication origins in this region can be activated in non-B cells, although it is not clear if this results from a normal developmental program or from the tumoral nature of HeLa cells.

### Mustiscale analysis of apparent replication speeds in the *FRA3B* fragile site

To further check our predictions of bidirectionally replicating CTRs and TTRs, we took advantage of the recent work of Letessier et al [58], who used DNA combing in fibroblasts and lymphoblastoid cells to reveal cell-type specific replication initiation programs at the *FRA3B* chromosome fragile site.

**Figure 9:**
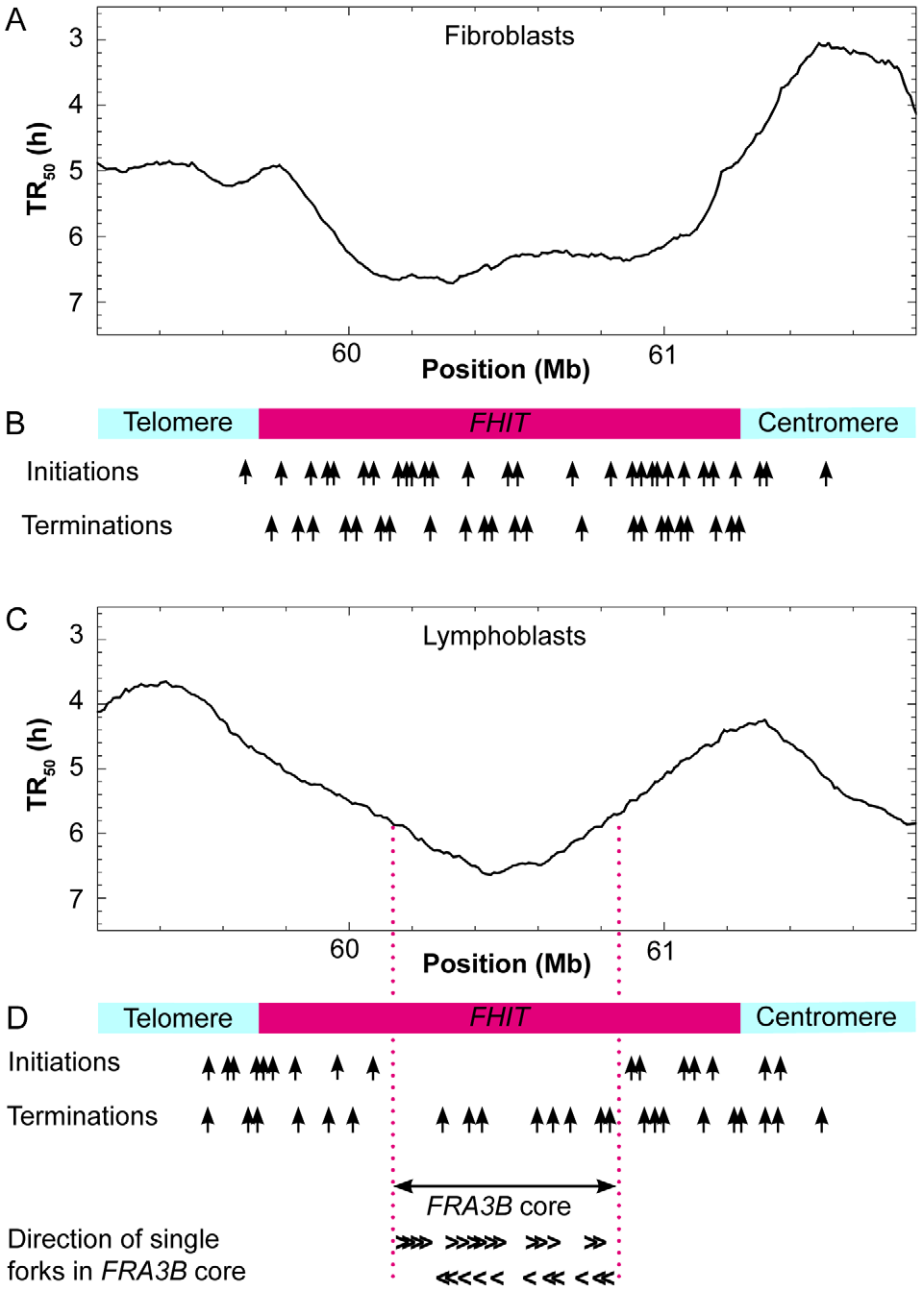
Replication mode of the *FHIT* locus in fibroblasts and lymphoid cells. (**A**) Replication timing profile of the locus in BJ fibroblasts and (**B**) summary of initiation and termination events mapped in MRC5 fibroblasts (data from Figure 2c and Figure S7 in [58]). The *FRA3B* region is embedded into a 1.2 Mb CTR that replicates by evenly spread initiations. (**C**) Replication timing profile of the locus in GM06990 lymphoblasts and (**D**) summary of initiation and termination events and direction of single forks mapped in JEFF lymphoblastoid cells (data from Figure 2a and Figure S3 in [58]). The *FRA3B* region maps at the bottom of two converging TTRs where forks move in both directions.

Analysis of the replication timing data of Hansen et al [13] shows that in BJ fibroblasts, the *FRA3B* region is embedded into a late-replicating 0.9 Mb CTR that is predicted to replicate by synchronous initiations (Figure 9A). The DNA combing results of Letessier et al. [58] entirely confirm this prediction, showing initiation and termination events evenly distributed all along this locus in MRC5 fibroblasts (Figure 9B).

In GM06990 lymphoblastoid cells, the *FRA3B* region lies within a V-shaped replication timing trough formed by two converging TTRs, each about 1 Mb in length (Figure 9C). Both TTRs replicate at an apparent speed of 6–8 kb/min, inconsistent with the mean (1.87 kb/min) and max (3.2 kb/min) velocity of single forks measured within this locus by DNA combing in JEFF lymphoblastoid cells [58]. Each of these two TTRs is therefore predicted to contain forks moving in both directions. A single fork moving at 2 kb/min could replicate up to 1 Mb of DNA within an 8 h S phase. Therefore, in principle, each TTR could be replicated without internal initiation if it is traversed by a single fork that is initiated two-thirds of the time on its early edge and one-third of the time on its late edge, since the resulting apparent speed would be *v*/|*R−L*| = 2/0.33 = 6 kb/min (Figure S3C). However, this scenario would predict that the edges of these TTRs would replicate either very early or very late in S phase, which is not supported by the timing data of Hansen et al [13] (see Figure 3 in Letessier et al [58]). The alternative hypothesis is that these TTRs replicate by internal initiations (Figure S3D). The data of Letessier et al [58] in JEFF cells indeed show initiations over the early and middle parts of each TTR, although initiations are excluded from a 700 kb region that corresponds to the late edges of both TTRs. Forks nevertheless are found to move in both directions in this 700 kb originless region [58], consistent with our predictions (Figure 9D). Another interpretation of these data would be that the edge of the early CTR is different in individual cells but the TTR is unidirectional in all cells, thus termination occurs at different points in the TTR. However, in order to quantitatively explain the discrepancy between the TTR slope and fork velocity, the position of this edge should differ by up to 1–2 Mb in different cells, which is again not supported by the timing data [13] [58]. Therefore, these results again confirm the validity of our analysis of apparent replication speeds in predicting regions that cannot replicate by unidirectional progression of a single fork.

### Comparison with replication origin maps in ENCODE regions

**Figure 10:**
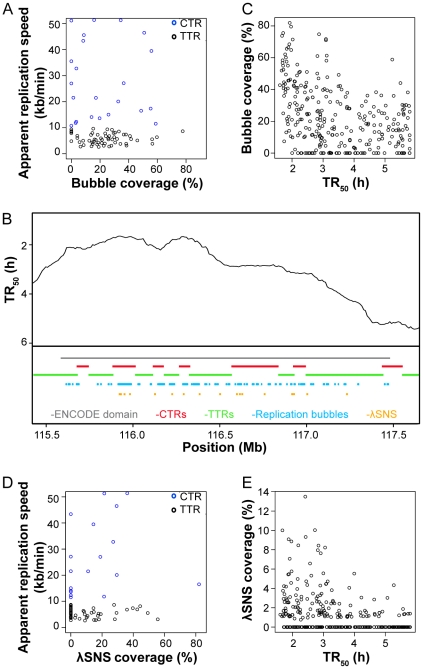
Analysis of replication bubble and λ-SNS coverage in ENCODE CTRs and TTRs. Replication bubble data are from log-phase HeLa Rep4 library [62] and λ-SNS data are from [64]. (**A**) Replication bubble coverage is plotted against apparent replication speed of CTRs (blue circles) and TTRs (dark circles). (**B**) An exemplary ENCODE region. Replication timing profile (dark wavy line) of region ENm001 (grey line) and its surroundings with replication bubbles (cyan) and λ-SNS (orange) in CTRs (red) and TTRs (green) are shown. (**C**) Replication bubble coverage computed by 100 kb adjacent windows along ENCODE regions is plotted against replication time. (**D**) λ-SNS coverage is plotted against apparent replication speed of CTRs (blue circles) and TTRs (dark circles). (**E**) λ-SNS coverage computed by 100 kb adjacent windows along ENCODE regions is plotted against replication time.

Mesner et al [62] have recently provided a reliable map of replication origins in 1% of the human genome in HeLa and GM06990 cells, using a novel replication-bubble trapping procedure to prepare nearly pure origin libraries that were hybridized to encyclopedia of DNA elements [ENCODE] microarrays [63]. We compared the coverage of CTRs and TTRs by replication bubbles within these regions in HeLa cells (Figure 10A, and Figure S5). Most CTRs and TTRs contained replication bubbles, consistent with our proposal that most of the genome replicates by internal initiations (see e.g. region ENm001, Figure 10B), and we found no major difference in replication bubble coverage in TTRs (22%) vs. CTRs (29%). We noted however a higher bubble coverage in early replicating regions (Figure 10C). This was surprising because we found by DNA combing that interorigin distances did not change during S phase. Similar results were obtained with bubbles mapped in GM06990 cells (data not shown). A potential explanation for this discrepancy is that early bubbles are more efficiently trapped, perhaps because they are more efficient and less delocalised than late ones. Early bubbles may also have a longer dwell time than late ones because they are less synchronous and slower to merge with neighboring bubbles. This interpretation would imply that an even larger fraction of the genome than found by Mesner et al [62] can support a significant level of delocalised, replication initiation of low efficiency.

We also compared the coverage of CTRs and TTRs by short RNA-primed, nascent DNA strands purified by λ exonuclease digestion (λ-SNS) by Cadoret et al [64]. Although there is only modest concordance between bubble and λ-SNS maps [62], we again found no major difference in λ-SNS coverage (Figure 10D and Figure S5) in TTRs (1.05%) vs. CTRs (1.71%) and a higher λ-SNS coverage in early replicating regions (Figure 10E). It is expected that λ-SNS peaks are less efficiently detected if initiation is more random in late replicating regions.

### A gradient of chromatin openness along TTRs

**Figure 11:**
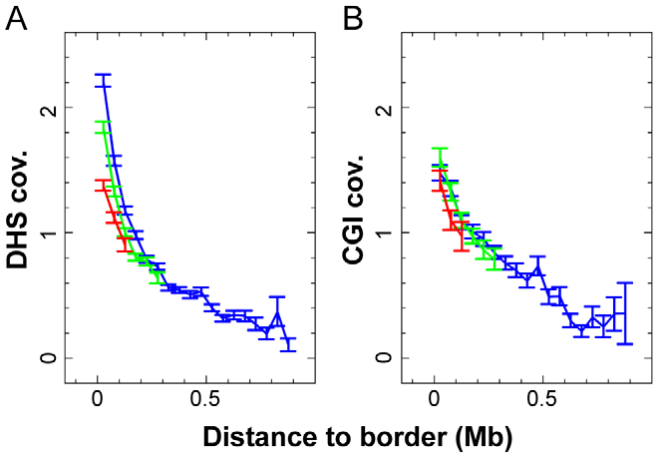
Representation of open chromatin markers along all TTRs relative to the corresponding genome-wide average value. (**A**) Mean coverage by DNase I hypersensitive zones, as a function of the distance to the earliest TTRs border. TTRs have been detected at 200 kb scale and classified by size: in red TTRs<200 kb; in green 200 kb<TTRs<360 kb; in blue TTRs>360 kb. (**B**) Mean coverage by 1 kb-enlarged CpG islands as a function of the distance to the earliest TTRs border. Three size categories have been defined: in red TTRs<200 kb; in green 200 kb<TTRs<360 kb; in blue TTRs>360 kb.

A general correlation between replication timing, chromatin openness and transcriptional activity has been reported [12], [65]–[67]. To examine this in further detail, we analyzed the distribution along TTRs of an experimental marker (DNase I hypersensitive sites determined in HeLa S3 cells) and a DNA sequence marker (CpG islands) of open and transcriptionally active chromatin available genome-wide. We observed that the average coverages are maximum at TTRs early border and steadily decrease when going towards the late border (Figure 11A, B). These gradients of open chromatin marker distribution are unchanged when considering small, intermediate and large TTR size classes, suggesting there exists a characteristic scale for the change of chromatin state along TTRs. These results raise the possibility that there is a direct link between the gradients of origin firing time and a gradient in chromatin openness along the TTRs.

### Temporal control of origin firing

**Figure 12:**
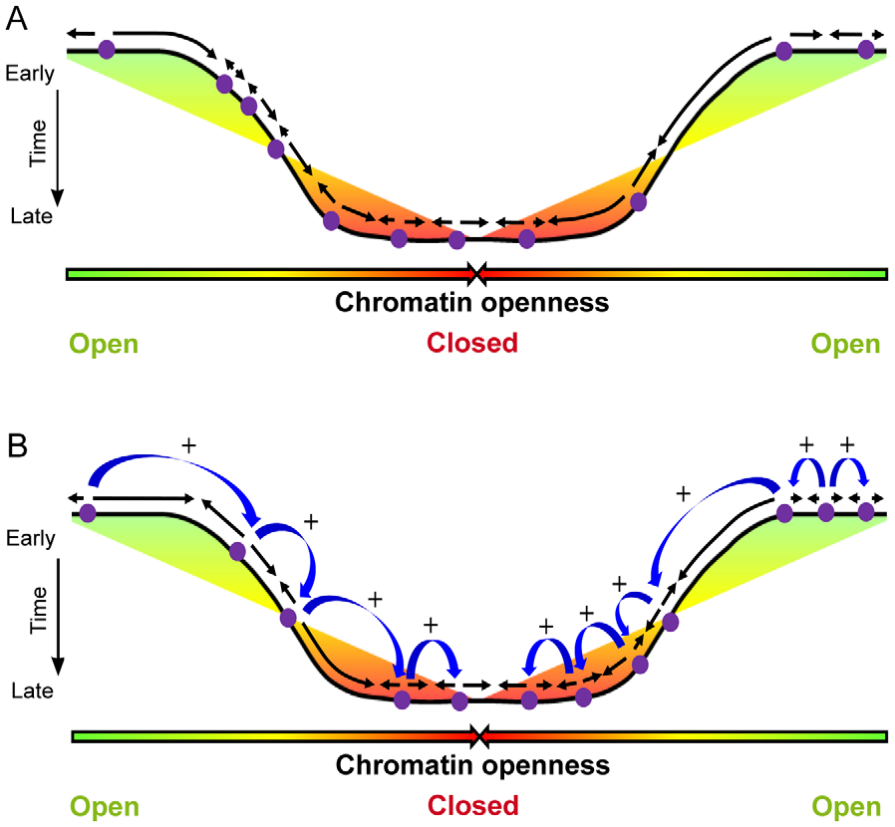
Two alternative models for origins activation along TTRs. (**A**) Replication first initiates at early firing origins. Origins fire independently of each other and are specified by an open chromatin structure. (**B**) Domino model in which replication initiates at early efficient origins. Activation of later origins in less open chromatin is stimulated by approaching replication forks from upstream origins. In both cases (A and B), the rate of origin firing increases during S phase resulting in a U-shaped replication timing profile. Origins of replication are marked by purple circles and black arrows show the direction of replication forks. Color variation (green to red) depicts the chromatin openness. Blue arrows indicate origin stimulation by replication forks from upstream origins.

The mechanisms that regulate the timing of replication are unknown. A simple model to account for our data is that origins have different relative firing probabilities and fire stochastically, and that the firing probability of all origins increases during S phase. Thus, efficient origins are likely to fire during early S phase and weak origins are unlikely to fire early but become more likely to fire during late S phase [19]. The firing probability of origins may be specified by chromatin structure, since there is a general correlation between replication timing and chromatin openness [12], [65]–[67] (Figure 12A). Consistent with this model, we show here that markers of open chromatin are correlated with early replication throughout TTRs (Figure 11), and we have previously reported that origin firing probability increases during S phase in a wide range of eukaryotes including human [21], [68]. Furthermore, both the combing data and the distributions of apparent replication velocities at different stages of S phase provide evidences for increasing origin firing during S phase. One observation, however, argues against a purely uniform and uncorrelated stochastic model: origin firings are temporally and/or spatially correlated. It is possible that neighbor origins fire independently of each other but are nevertheless temporally correlated because their timing is set by some underlying chromatin features that change over a characteristic distance longer than individual replicons.

An attractive alternative mechanism to explain the progressive activation of neighboring origins along the TTRs is that active forks stimulate nearby initiation in unreplicated DNA. As discussed elsewhere [21], [69], [70], forks may stimulate initiation due to changes in DNA supercoiling in front of the fork or to association of chromatin remodellers or origin triggering factors with replication fork proteins. Early studies of replication foci labelled by two consecutive pulses showed that the intranuclear distance between consecutively replicated domains increased linearly with the time interval between the labels [36]. Studies on the dynamics of PCNA assembly at replication foci indicated that once replication is completed at a given site, a new replication focus assembles de novo at a neighboring site, consistent with a domino effect in activation of neighboring origins [38], [39]. A more recent study of S phase progression in HeLa cells suggested that replication foci that lie side-by-side in the nuclei are replicated in consecutive intervals of S phase because of their genetic continuity along the chromosomal fiber and that a “next-in-line” principle defines the efficiency with which origins are activated once S phase has begun [40]. In this work, we have quantitatively analyzed the speed of the replication wave progression and shown that it is consistent with a cascade of origin activation along TTRs as predicted by a domino model for origin activation. Thus, replication would first initiate in efficient zones of variable size specified by an open chromatin structure [67], followed by progressive activation of flanking origins in less open chromatin due to the approach of an incoming fork (Figure 12B). This model explains why adjacent origins tend to fire synchronously, why replication progresses faster than a single fork and why origins embedded in closed chromatin do not fire in early S phase but fire efficiently when the replication wave reaches them. With an increasing rate of origin firing during S phase [21], [68], this domino model can further explain why the apparent speed of replication increases along replication timing gradients, and predicts a progressive change in replication fork polarity along these gradients.

Works from several groups suggest that activation of one origin within a potential initiation zone suppresses rather than activates the activation of immediately surrounding origins [71]–[73]. However, the range of this negative origin interference is limited to distances smaller than the typical interorigin distance and is not incompatible with positive origin interference acting over larger distances [71], [74]. Data on origin spacing and synchrony in Xenopus egg extracts are indeed consistent with a mechanism whereby loop formation between a potential origin and an approaching fork suppresses initiation at very close spacing and enhances initiation at a larger, characteristic distance [29], [71], [74], [75].

In favor of a role of fork progression in controlling sequential origin activation, a recent study in yeast has shown that mutants deficient in chromatin remodeling activities located at replication forks specifically delay the replication of late replicating domains [76]. On the other hand, a study with mammalian cells has shown that exposure of aphidicolin-arrested cells to checkpoint inhibitors results in initiation of replication at successively later-replicating domains in the absence of detectable elongation of replication forks [77]. This suggests that fork elongation is not strictly required for at least the global aspect of temporal origin activation, but does not prove that it has no role in this process. Furthermore, it is possible that only the earliest origins are activated in successive large-scale replication domains, and that secondary origins within a domain require activation by replication forks.

### Conclusion

In this work, we have performed a quantitative analysis of human genome replication in cells sorted into four or six stages of S phase, using DNA combing, mathematical analysis of replication timing profiles generated by massive sequencing of newly replicated DNA, and bioinformatic analysis of replication origin maps and chromatin structure data. The results show that i) replication origins fire in a correlated manner and at an increasing rate during S phase, ii) the apparent speed of replication progression throughout the genome depends on both the velocity of single forks and the proportion of rightward and leftward moving forks in the cell population, and ultimately reflects the pattern of origin firings along replication timing gradients rather than the unidirectional progression of a single fork. The correlation between adjacent origin firings may be due to their common chromatin environment or to a stimulation of origin firing by approaching forks. Further analyses and mathematical modelling of replication timing profiles are underway to explore these issues.

## Materials and Methods

### Molecular combing of DNA from sorted cells in four S-phase compartments

Asynchronously growing HeLa cells were labeled for 20 min with 25 µM IdU, washed with 1× PBS, and labeled for another 20 min with 25 µM CIdU. At the end of the labeling period, cells were harvested by trypsinisation, centrifuged at 500 g for 10 min at 4°C, washed in ice-cold 1× PBS, centrifuged again and fixed in 80% ethanol in 1× PBS. The fixed cells were centrifuged at 500 g for 5 min and resuspended in 1× PBS, 0.2 mg/mL RNaseA, 67 µg/mL propidium iodide at a final concentration of 2.10^6^ cells/mL. Cells were sorted in four replication temporal compartments S1, S2, S3, and S4 based on their DNA content. DNA was extracted after encapsulation of cells in low-melting point agarose blocks at 60.000 genome equivalents per block (e.g. 60.000 cells for S1 and 30.000 for S4) and combed on silanised coverslips as described [78]. To detect the DNA molecules and the IdU and CldU labels, combed DNA was denatured in 50% formamide, 2× SSC for 10 min at 80°C. Coverslips were blocked in a humid chamber for 30 min at 37°C in antibody dilution buffer (1.5% blocking reagent (Roche), 0.05% Tween 20 in 1× PBS). The following sequential incubations were performed: (1) CldU detection: 1/20 rat anti-BrdU (Abcys) 1 hour, 1/25 chicken anti-rat Alexa Fluor 488 20 min, 1/25 goat anti-chicken Alexa Fluor 488 20 min. (2) IdU detection: 1/5 mouse anti-BrdU (Becton Dickinson) 1 hour, 1/200 rabbit anti-mouse Alexa Fluor 350 20 min, 1/25 goat anti-rabbit Alexa Fluor 350 20 min. (3) Total DNA detection : 1/25 mouse anti-human DNA (Millipore) 1 h, 1/25 goat anti-mouse Alexa Fluor 594 1 h. Coverslips were mounted in phenylenediamine and stored at −20°C before analysis. Incubations were at 37°C (except for the first step of incubations 1 and 2, at room temperature) in a humid chamber and washes between successive antibodies were three times in 1× PBS for a total of 15 min (anti-BrdU and anti-DNA antibodies) or 9 min (secondary antibodies). Coverslips were scanned using an Olympus IX81 or a Nikon Ti inverted microscope with a 100× objective, both connected to a CoolSNAP HQ CCD camera (Photometrics) run by MetaMorph version 6.3r7 (Molecular Devices). Fluorescent signals were analyzed with ImageJ software (Rasband, W.S., ImageJ, U. S. National Institutes of Health, Bethesda, Maryland, USA, http://rsb.info.nih.gov/ij/, 1997–2009.) and Adobe Photoshop 9.0.2 software. Data were inserted in an Excel® (Microsoft®) spread sheet and analyzed using R (http://www.r-project.org).

Several arguments suggest that IdU/CldU labeling had minimal effect on the rate of replication. First, it has been shown previously that the range of BrdU concentrations used for DNA combing does not affect the growth of yeast cells [79]. Second, the concentrations of IdU and CldU we used (25 µM) are among the lowest employed in numerous comparable studies (25–100 µM). Third, the rate of fork progression calculated from the IdU or IdU+CldU tracks was the same (data not shown), suggesting that a doubling of the total analog concentration did not affect fork progression.

### DNA combing analysis of the IgH TTR

Asynchronously growing HeLa cells were labeled with IdU and CldU and sorted and DNA was combed as described above except that cells were sorted in a single S phase compartment. Two sets of 3 fosmids (G248P83284G6, G248P8783H11, G248P81611C11) and (G248P86652F6, G248P87335H11, G248P81864F6) were biotinylated by random priming (Bioprime labeling system, Invitrogen) and were independently hybridized to the combed DNA as described [72]. Fosmids were selected on http://genome.ucsc.edu and distributed by http://bacpac.chori.org. Antibody incubation, washes and slide mounting for IdU/CldU and DNA detection were performed as described above with the following changes: DNA detection was coupled to FISH detection by sequential incubation (for 20 min each) with: 1/25 mouse anti-human DNA and 1/25 Alexa Fluor 594 conjugated Streptavidin, 1/50 biotinylated anti-streptavidin and 1/25 mouse anti-human DNA, 1/25 Alexa Fluor 594 conjugated Streptavidin and 1/25 goat anti-mouse Alexa Fluor 647, 1/50 biotinylated anti-streptavidin and 1/25 goat anti-mouse Alexa Fluor 647, 1/25 Alexa Fluor 594 conjugated Streptavidin and 1/25 chicken anti-goat Alexa Fluor 647.

### Flow cytometry

We studied S-phase progression by flow cytometry analysis based on DNA content and IdU/CldU incorporation as described previously [80] with minor modifications. Here asynchronous HeLa cells were pulse-labeled with 25 µM IdU for 20 min and 25 µM CldU for another 20 min. DNA was stained with 1/5 mouse anti-BrdU antibody (Becton Dickinson) then with 1/25 rabbit anti-mouse Alexa Fluor 488 (invitrogen). Samples were analyzed on a CyAn ADP LX (Beckman Coulter).

### DNA content correction

To correct DNA content observed in DNA combing, we used flow cytometry analysis to estimate the percentage of IdU/CldU negative cells (i.e. fluorescence≤15) in S1–S4 fractions (Figure 2C). We measured at 31.5%, 9.05%, 4.65% and 15.1% the non replicative cells for S1–S4 fractions. Amount of DNA from these fractions have been subtracted from the total DNA length measured by combing. A second correction has been applied in order to remove previously synthesized DNA for each fraction. To this end, the mean DNA content for each fraction was estimated using FACS profiles (1, 1.15, 1.34, 1.57, 1.82 and 2 respectively for G1, S1 to S4 and G2) and then used to divide the previously corrected total DNA length.

### Determination of time required to duplicate the entire genome

The time required to duplicate the entire genome was estimated as the sum of times spent in S1, S2, S3 and S4 phases and corrected by the proportion of the genome that is replicated during these phases. S1–S4 lengths were individually calculated using the following equation: T_Si_ = Q_Si_/V_GSi_ where Q_Si_ is the quantity of DNA synthesised, V_GSi_ is the global progression of DNA replication. Q_Si_ was calculated as: Q_Si_ = 3.10^9^×P_Si_ with P_Si_ determined by FACS profiles and corresponding to the proportion of the genome that is replicated in each S1–S4 compartment (respectively 15, 19, 22 and 20%). V_GSi_ was calculated using the following equation V_GSi_ = N_FSi_×V_Si_ where N_FSi_ is the quantity of forks in S1–S4 phases (i.e. forks density×genome length) and V_Si_ is the replication forks velocity. The time required to duplicate the entire genome was computed as T = ΣTsi/ΣQsi.

### The apparent replication speed is estimated by *v/(R−L)*

In one cell cycle, the timing profile around an active origin of replication has a typical inverted V shape corresponding to a local minimum (timing increases when going downward). Downstream of the origin of replication, loci are replicated by forks coming from their left, therefore *R* = 1 and *L* = 0, and in that region the timing profile has a positive derivative dt/dx = 1/*v* where *v* is the replication fork velocity. Respectively, upstream of the origin of replication, *L* = 1 and *R* = 0 and the timing profile has a negative derivative dt/dx = −1/*v*. Therefore, the derivative of the timing profile of a single cell is given by dt/dx = (*R−L)*/*v*. It can be shown that this result still holds when considering the average over a cell population: the average fork polarity is provided by the derivative of the average timing profile given a constant fork velocity. Given the finite resolution of the experimental average timing profile, we defined the apparent speed at scale X kb as the inverse of the slope of the timing profile computed at that scale. This apparent velocity is equal to the fork velocity divided by the average fork polarity over that scale.

### Determination of the replication timing profiles

For HeLa cells, we previously generated a profile of S_50_, the fraction of S phase at which 50% of the DNA is replicated in a defined genome region, using massively parallel sequencing of BrdU-labeled nascent DNA from sorted cells in S1, S2, S3, S4 [12]. To verify the DNA content of sorted cells for DNA combing and replication timing experiments, 10^5^ BrdU-labelled, sorted cells in S1, S2, S3, S4 and 10^5^ sorted cells with a DNA content ranging from G1 to G2 were re-stained with propidium iodide and their DNA content examined by FACS (Figure S1). This control showed that the sorted cells had the expected DNA content.

The enrichment of sequence read densities relatively to background was computed along the genome for each of the four compartments of the S phase and S_50_ values were computed by linear interpolation of enrichment values [12]. TR50, the time at which a defined genome region had replicated in 50% of the cells was then deduced from S50 values as follows.

The FACS DNA fluorescence histogram was analysed using a modified version of the method developed by Bertuzzi et al. [81]. We assumed that in average all cells whose DNA content at time *t* is *x*, synthesize their DNA with the same rate *φ(x)* that we approximated with the sum of six Gaussian functions. The fraction of cells in S phase (equation (16) in [81]) with a DNA content *x* is given by:
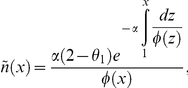
where *θ_1_* is the fraction of cells in G1 phase measured by integration of the peak of the FACS fluorescence histogram corresponding to the cells in G1; the term 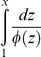 represents the time spent by a cell in S phase (equation (7) in [81]). Using the fundamental equation of cytofluorimetry [82] and the expression for *ñ*(x) we fitted the fluorescence histogram utilising a simplex algorithm and extracted the profile of *φ(x)*. Using the expression for time as a function of DNA content, 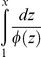, we obtained the overall fraction of the genome that has replicated at time *t* in S phase. This was used to convert S_50_ into TR_50_.

For the other cell lines, we determined a profile of S_50_ using Repli-Seq tags for 6 FACS fractions that were obtained from the authors [13]. For a given cell line and for each S-phase fraction, we computed the tag densities in 100 Kb windows, and following the authors [13] the tag densities were normalized to the same genome-wide sequence tag counts for each fraction. We performed a second normalization so that at each genomic position, the sum over S-phase fractions be one. To filter out the noise which could critically bias mean timing profile estimate, we proceeded as follow. We noticed that the genome-wide distribution of the normalized tag density presents a mode at 0.01<m<0.08 (mainly noise) and a long tail up to 1 (mainly corresponding to the replication signal). For each S-phase fraction we set to 0 the normalized tag density <4 m, and re-normalized at each genomic position by the sum over S-phase fractions. The mean replication timing profile computed on these denoised tag densities superimposed on the original one, but was much less noisy. We directly converted S_50_ into TR_50_ assuming an S phase length of 8 h and a linear mapping between DNA content and S phase progression.

### Multiscale analysis of apparent replication speeds and identification of CTRs and TTRs

The apparent replication speed of a locus intuitively corresponds to the inverse of the slope of the replication timing profile. In fact the timing profile is noisy so that its derivative is strictly speaking not defined. We used the continuous wavelet transform (WT), a powerful framework for the robust estimation of signal variations over any length scales [83], [84], to obtain a well defined and numerically stable measurement of the local slope of the timing profile at any scale of observation. This allowed us to construct the space-scale map of apparent replication speeds (Figure 3B and Figure S2B). Using this map, CTRs and TTRs were delineated as the contiguous regions where the speed is above (resp. below) a constant threshold (10 kb/min) at a given scale of observation (100, 200 and 500 kb) (Figure 4).

### Sequence and annotation data

Sequence and annotation data were retrieved from the Genome Browsers of the University of California Santa Cruz (UCSC) [85]. Analyses were performed using the human genome assembly of March 2006 (NCBI36 or hg18). We used CpG islands (CGIs) annotation provided in UCSC table “cpgIslandExt”. As previously done, we computed 1 kb-enlarged CGI coverage as an hypomethylation marker [67].

### DNase I hypersensitive site data

We used the DNaseI sensitivity measured genome-wide in HeLa S3 cell line using the Digital DNase I methodology [44], [45]. Data corresponding to Release 3 (Jan 2010) of the ENCODE UW DNaseI HS track, were downloaded from the UCSC FTP site: ftp://hgdownload.cse.ucsc.edu/goldenPath/hg18/encodeDCC/wgEncodeUwDnaseSeq/. We plotted the coverage by DNase Hypersentive Sites (DHSs) identified as signal peaks at a false discovery rate threshold of 0.5% within hypersensitive zones delineated using the HotSpot algorithm (“wgEncodeUwDnaseSeqPeaks” tables).

### ENCODE data

The coordinates of replication-bubble trapping fragments of HeLa and GM06990 cells within ENCODE regions were obtained from the authors [62].

## Supporting Information

Figure S1 **Post-sort control.** (**A**) FACS profile and windows used to sort S1, S2, S3, S4 cells and cells with a DNA content ranging from G1 to G2. (**B**) FACS profiles of the resorted cell populations: S1 (blue), S2 (red), S3 (green), S4 (cyan) and whole cycle population (dark). (**C**) Quantitative analysis of the resorted cells in S1, S2, S3, S4 fractions.
(TIF)

Figure S2 **Replication timing profiles segmented in CTRs/TTRs and multiscale analysis of apparent replication speeds.** (**A**) Profile of replication timing (TR_50_ in hours) along the genome. Small TR_50_ values correspond to early replicating regions; large TR_50_ values correspond to late replicating regions. The replication timing profile was segmented into regions that replicate at apparent speed >10 kb/min (CTRs: Constant Timing Regions, red horizontal lines) and <10 kb/min (TTRs: Timing Transition Regions, green oblique lines) at scale 100 kb. (**B**) Multiscale analysis of apparent replication speeds along the genome. Replication speeds determined by wavelet transform analysis (see Material and Methods) at scales indicated on the y-axis are shown in three colors (blue, <2 kb/min; green, from 2 to 10 kb/min; red, >10 kb/min).
(PDF)

Figure S3 **Models for replication fork progression in Constant Timing Regions (CTRs) and Timing Transition Region (TTRs).** (**A**) A CTR is passively replicated from left to right in one half of the cells and from right to left in the other half. The average replication time is in mid-S phase for all sequences. (**B**) A CTR is replicated from multiple, synchronous internal initiations. The average replication time can be any time in S phase. This time is constant all along the CTR. (**C**) A TTR is passively replicated from left to right in two-thirds of the cells and from right to left in the other third. The average replication time changes from early to late S phase from left to right and the apparent replication speed = 3*v*, where *v* is the speed of a single fork. (**D**) A TTR replicates from multiple, consecutive initiations. The apparent speed is the mean replicon size divided by the mean time interval between successive initiations.
(TIF)

Figure S4 **Complete set of all molecules of the IGH TTR analyzed by DNA combing.** The top diagram shows a map of the IGH region, the position of the fosmid probes (red lines) and chromosome coordinates. The bottom panel shows the complete set of molecules schematized in Figure 8D.
(PDF)

Figure S5 **Comparison of replication timing data with replication bubble data in ENCODE regions.** Each page shows: (**top**) the extent of each ENCODE region (dark line), the segmentation into CTRs (blue) and TTRs (red), the mapping of replication bubbles in log-phase HeLa library Rep3 (orange) and Rep4 (purple) and when the two libraries were combined (pale blue); (**middle**) the replication timing profile of the considered region and its immediate surroundings; (**bottom**) the signed apparent replication speed at scale 100 kb.
(PDF)
