# Supplementary figures and images for: Evidence for Sequential and Increasing Activation of Replication Origins along Replication Timing Gradients in the Human Genome
Source: PLoS Comput Biol. 2011 Dec 29;7(12):e1002322. doi: 10.1371/journal.pcbi.1002322 (PMC3248390; doi:10.1371/journal.pcbi.1002322)

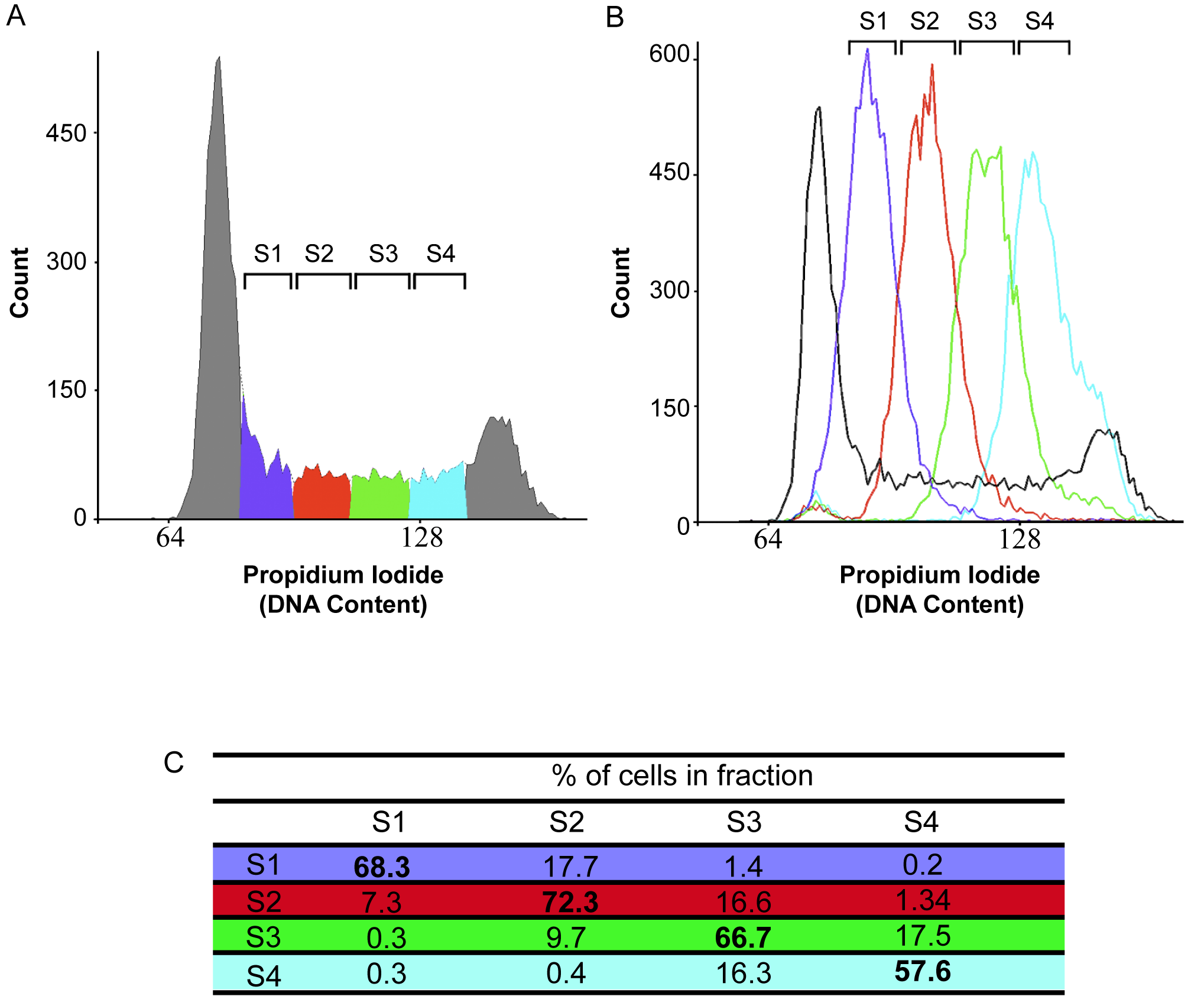

Supplement: Figure S1 — Post-sort control. (A) FACS profile and windows used to sort S1, S2, S3, S4 cells and cells with a DNA content ranging from G1 to G2. (B) FACS profiles of the resorted cell populations: S1 (blue), S2 (red), S3 (green), S4 (cyan) and whole cycle population (dark). (C) Quantitative analysis of the resorted cells in S1, S2, S3, S4 fractions. (TIF) [file pcbi.1002322.s001.tif]

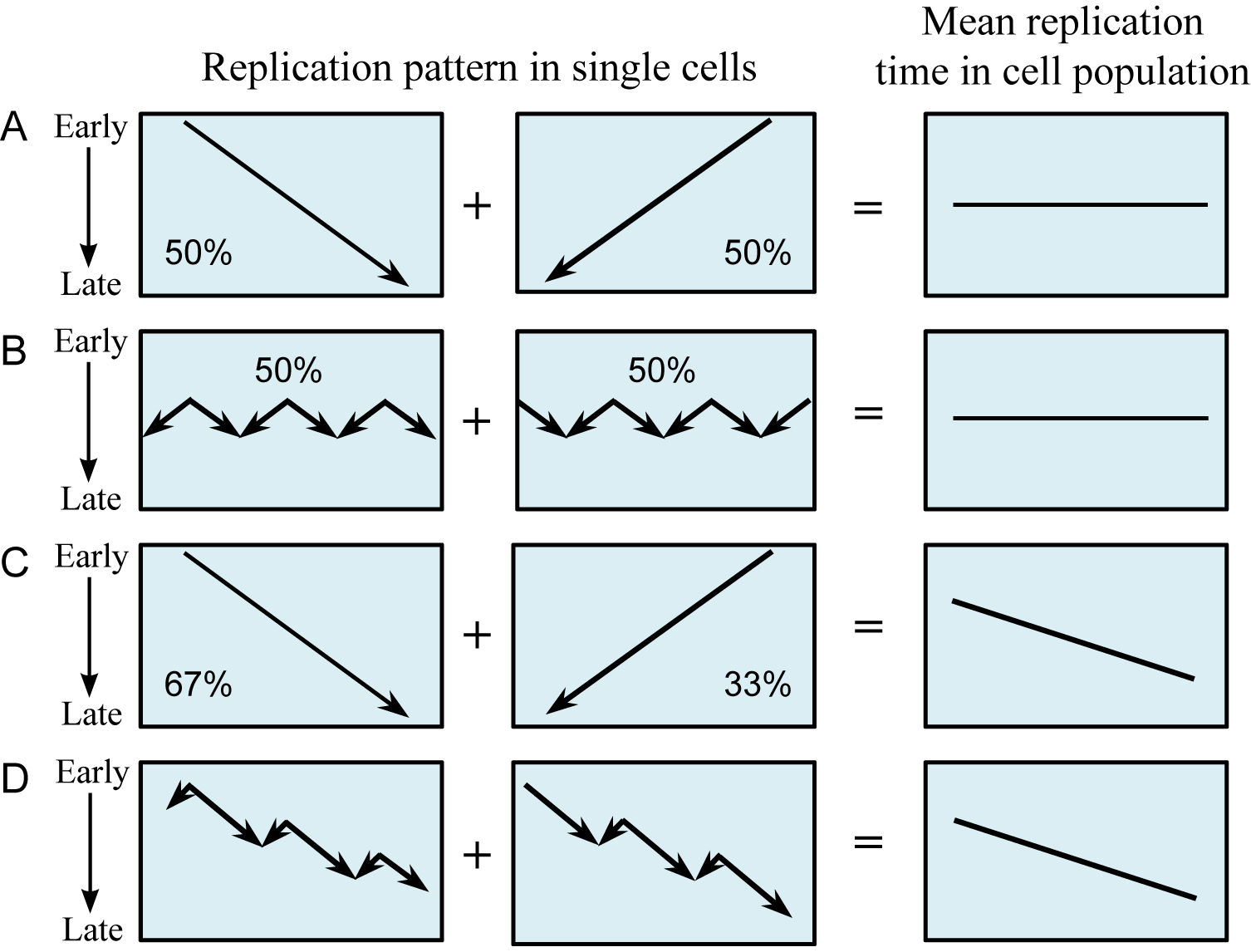

Supplement: Figure S3 — Models for replication fork progression in Constant Timing Regions (CTRs) and Timing Transition Region (TTRs). (A) A CTR is passively replicated from left to right in one half of the cells and from right to left in the other half. The average replication time is in mid-S phase for all sequences. (B) A CTR is replicated from multiple, synchronous internal initiations. The average replication time can be any time in S phase. This time is constant all along the CTR. (C) A TTR is passively replicated from left to right in two-thirds of the cells and from right to left in the other third. The average replication time changes from early to late S phase from left to right and the apparent replication speed = 3v, where v is the speed of a single fork. (D) A TTR replicates from multiple, consecutive initiations. The apparent speed is the mean replicon size divided by the mean time interval between successive initiations. (TIF) [file pcbi.1002322.s003.tif]
