# Supplementary material for: Evidence for Sequential and Increasing Activation of Replication Origins along Replication Timing Gradients in the Human Genome
Source: PLoS Comput Biol. 2011 Dec 29;7(12):e1002322. doi: 10.1371/journal.pcbi.1002322 (PMC3248390; doi:10.1371/journal.pcbi.1002322)
Supplement: Figure S2 — Replication timing profiles segmented in CTRs/TTRs and multiscale analysis of apparent replication speeds. (A) Profile of replication timing (TR50 in hours) along the genome. Small TR50 values correspond to early replicating regions; large TR50 values correspond to late replicating regions. The replication timing profile was segmented into regions that replicate at apparent speed >10 kb/min (CTRs: Constant Timing Regions, red horizontal lines) and <10 kb/min (TTRs: Timing Transition Regions, green oblique lines) at scale 100 kb. (B) Multiscale analysis of apparent replication speeds along the genome. Replication speeds determined by wavelet transform analysis (see Material and Methods) at scales indicated on the y-axis are shown in three colors (blue, <2 kb/min; green, from 2 to 10 kb/min; red, >10 kb/min). (PDF) [file pcbi.1002322.s002.pdf]

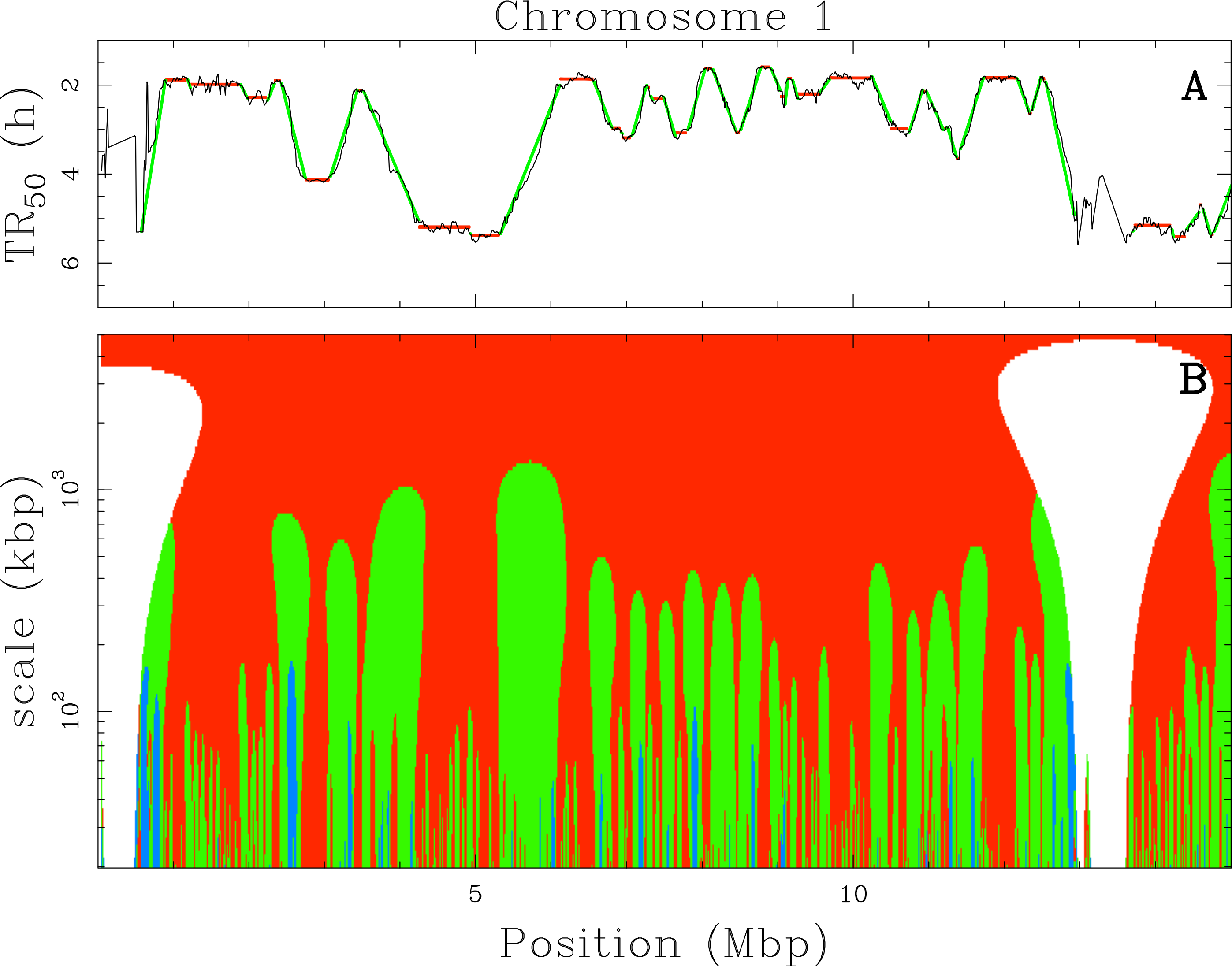

Chromosome 1
A
TR50 (h)
2
4
6
B
scale (kbp)
10^3
10^2
5
10
Position (Mbp)

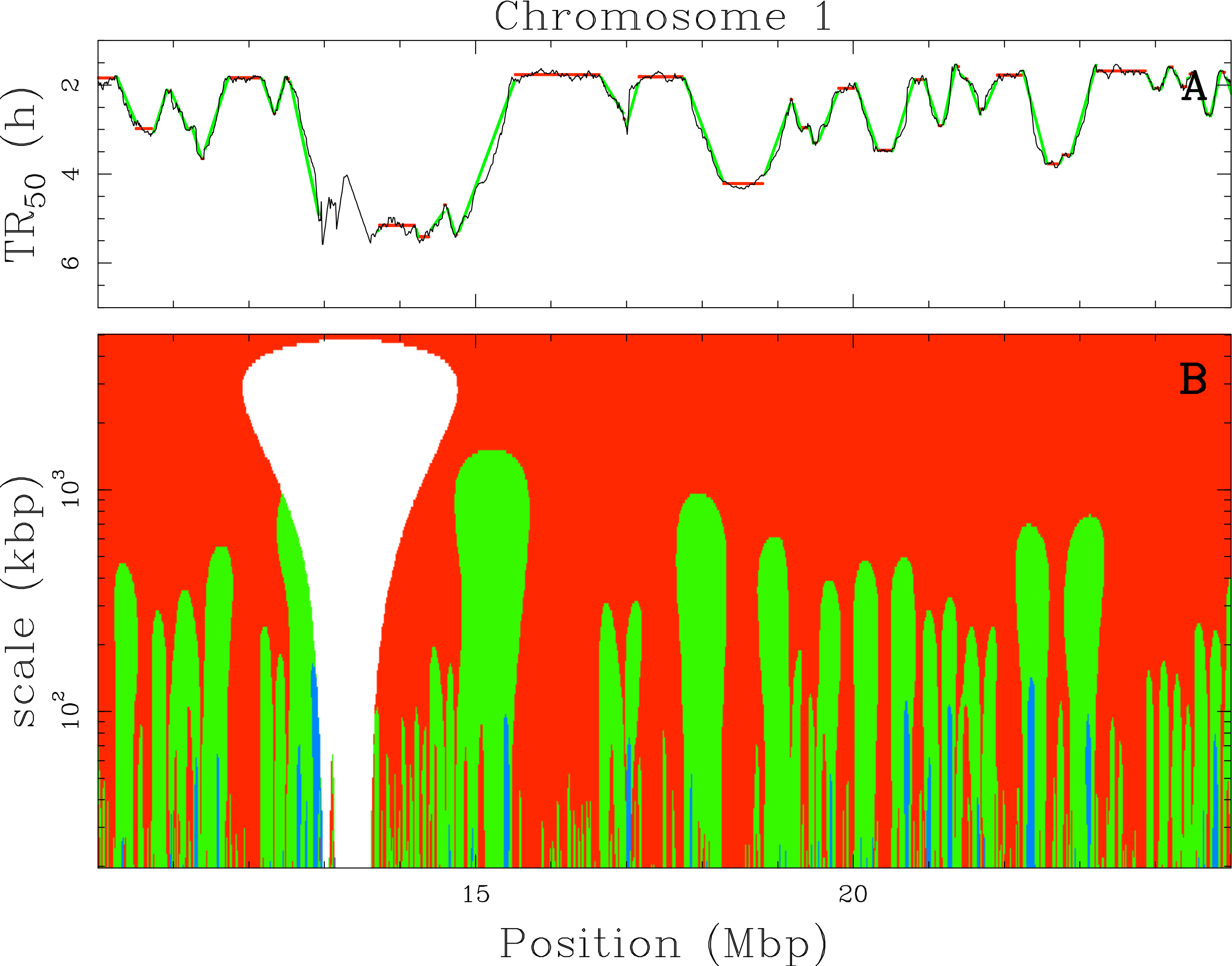

Chromosome 1
TR50 (h)
2
4
6
A
B
scale (kbp)
10^3
10^2
15
20
Position (Mbp)

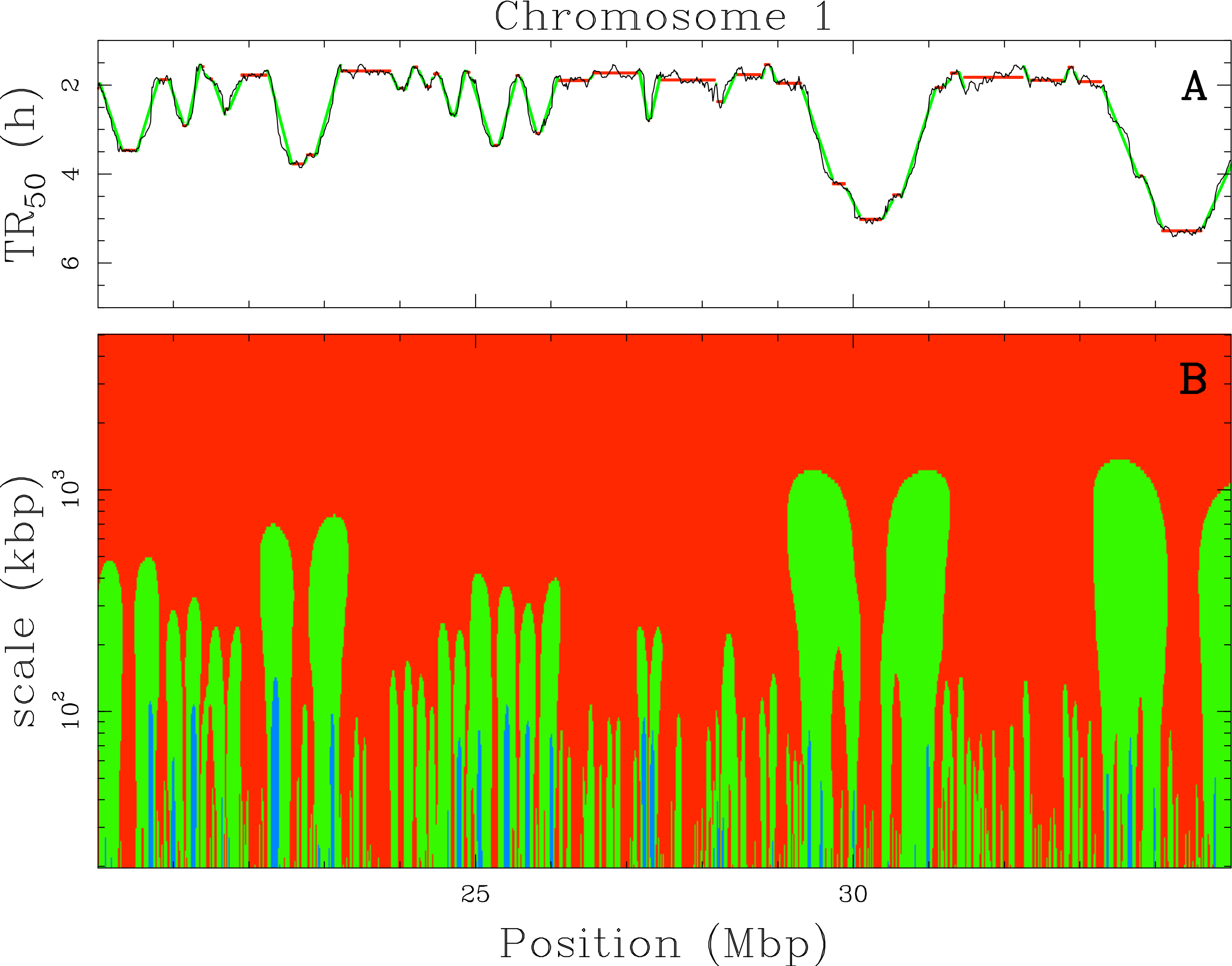
Chromosome 1
TR50 (h)
2
4
6
A
scale (kbp)
10^3
10^2
B
25
30
Position (Mbp)

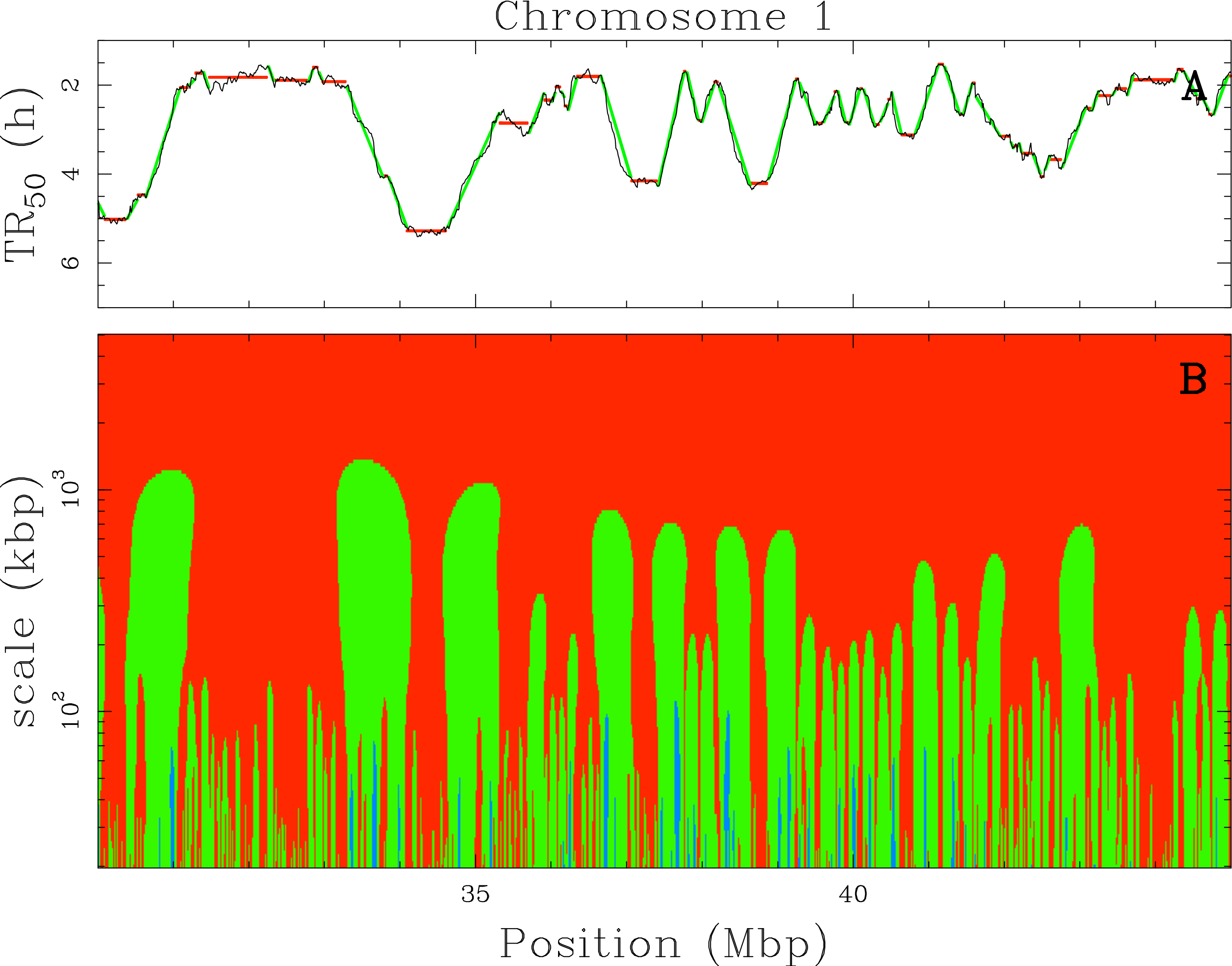
Chromosome 1
TR50 (h)
2
4
6
A
B
scale (kbp)
10^3
10^2
35
40
Position (Mbp)

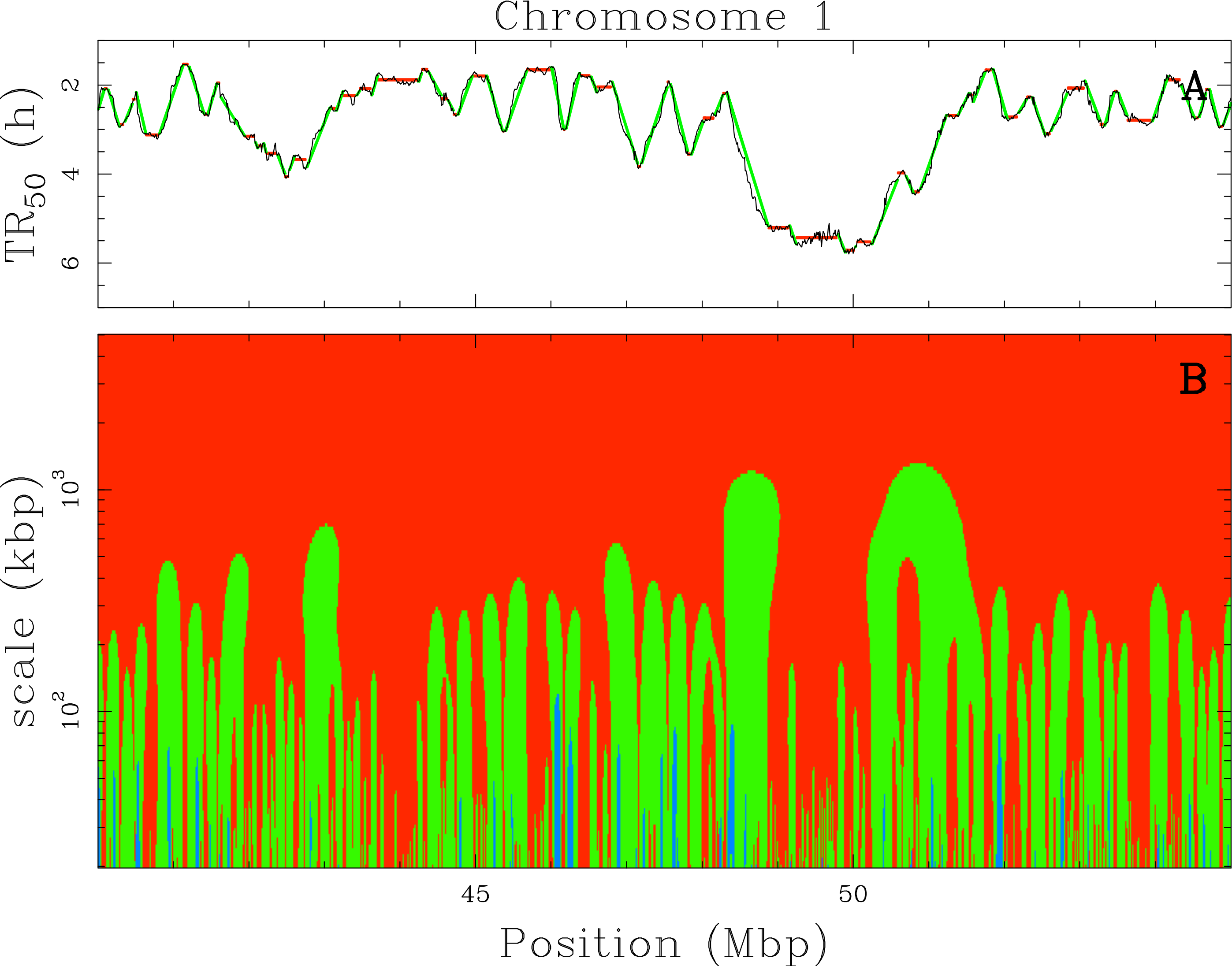

Chromosome 1
TR50 (h)
2
4
6
A
B
scale (kbp)
10^3
10^2
45
50
Position (Mbp)

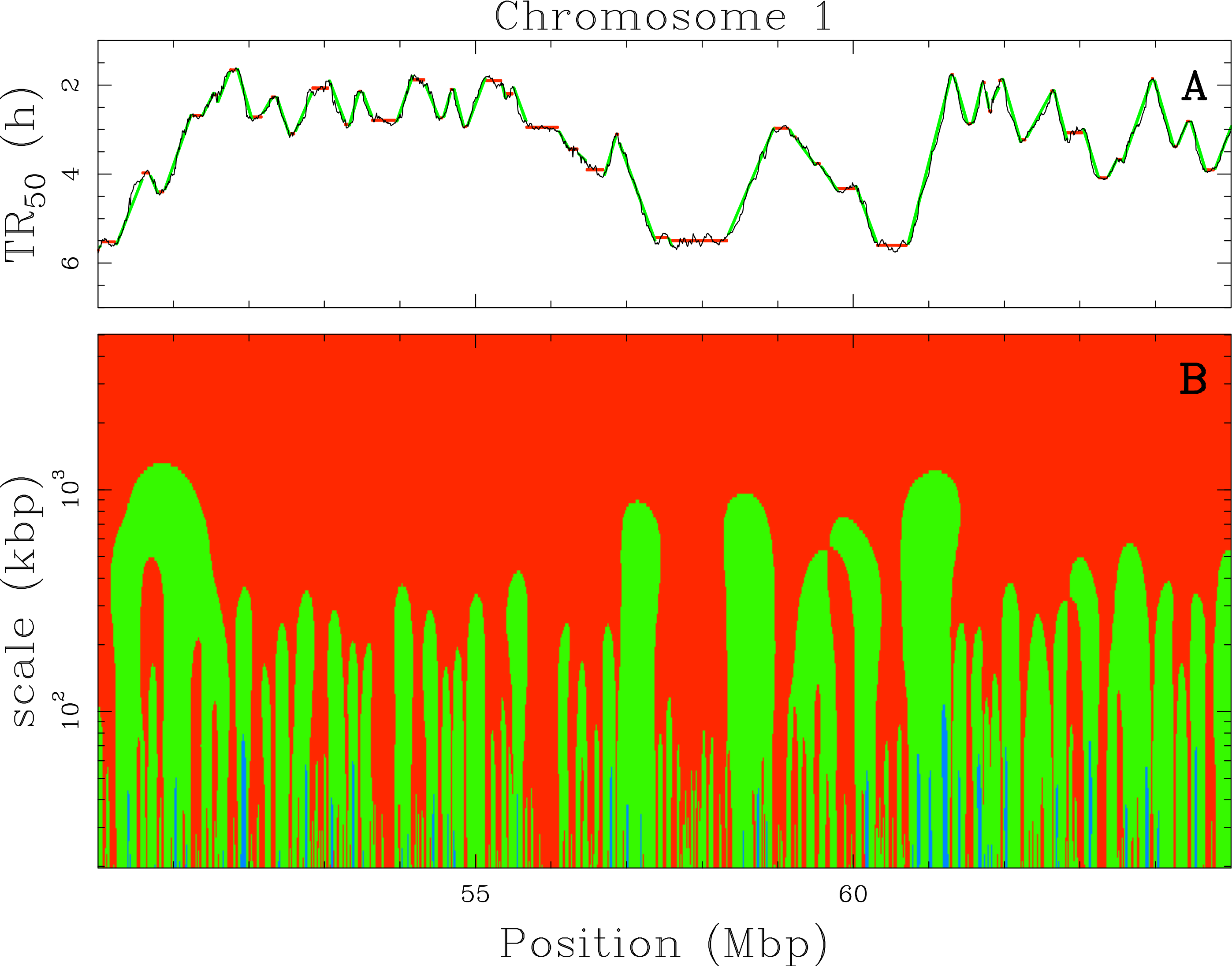

Chromosome 1
A
B
TR50 (h)
2
4
6
scale (kbp)
10^3
10^2
55
60
Position (Mbp)

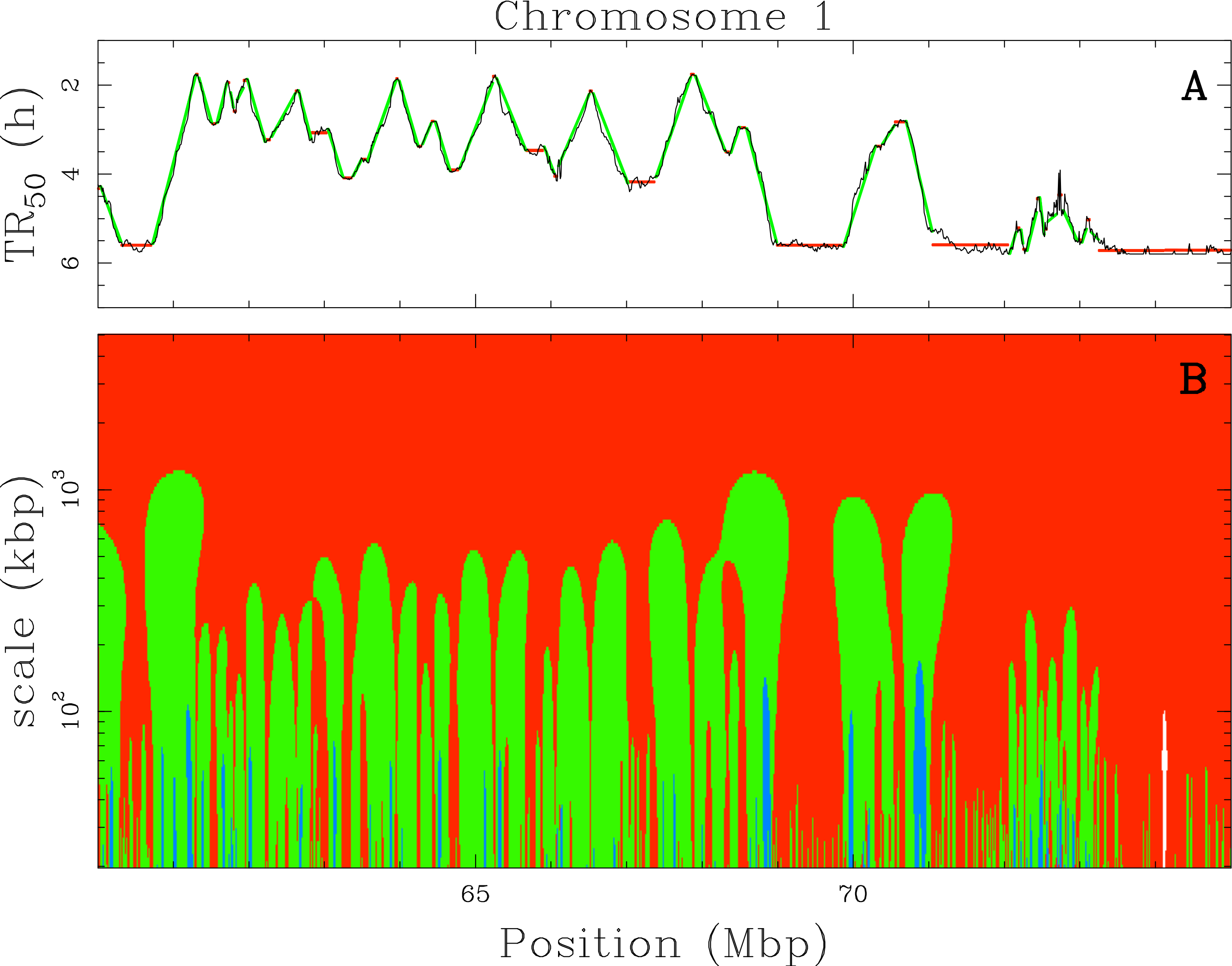

Chromosome 1
A
B
TR50 (h)
2
4
6
scale (kbp)
10^3
10^2
65
70
Position (Mbp)

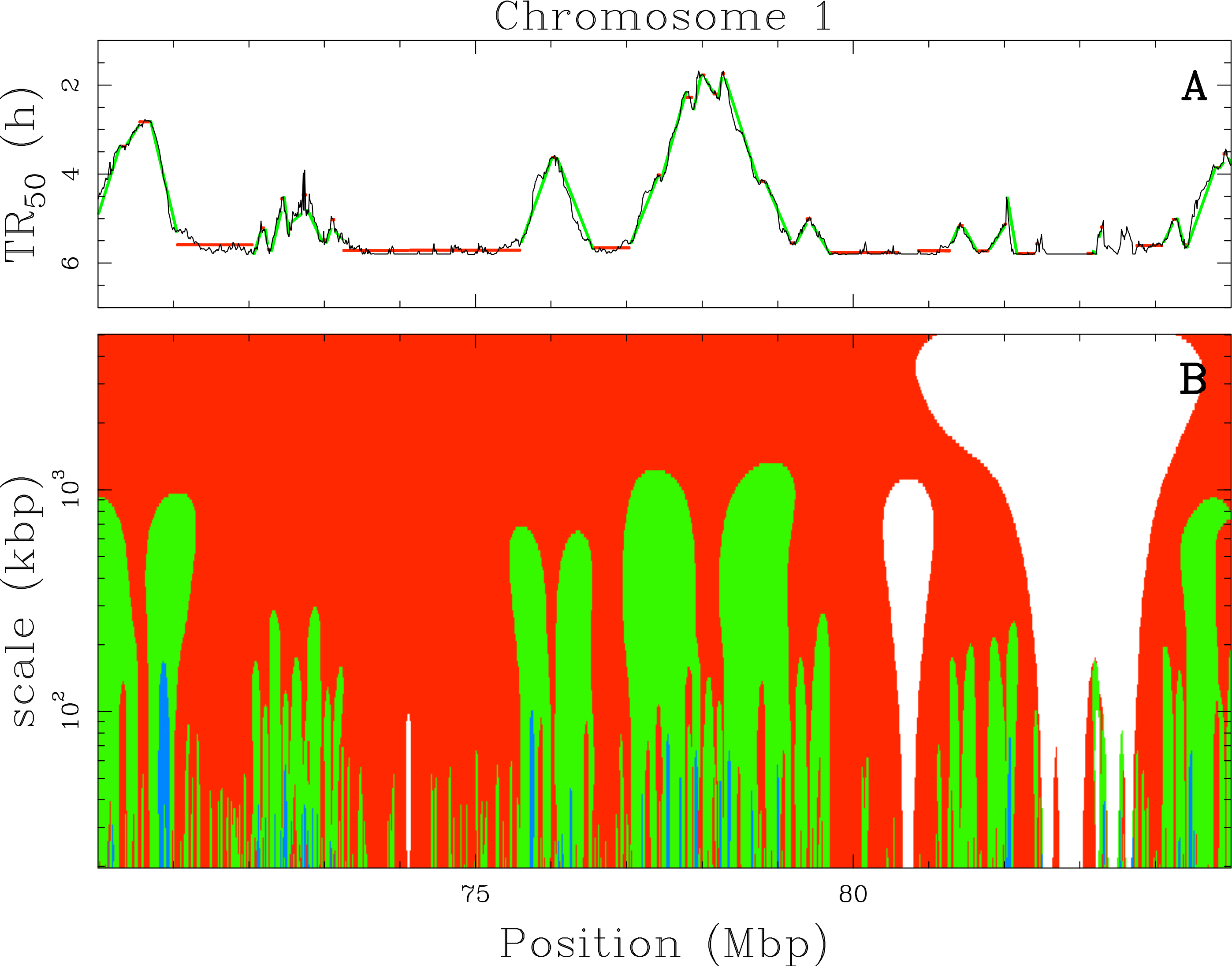

Chromosome 1
A
B
TR50 (h)
2
4
6
scale (kbp)
10^3
10^2
75
80
Position (Mbp)

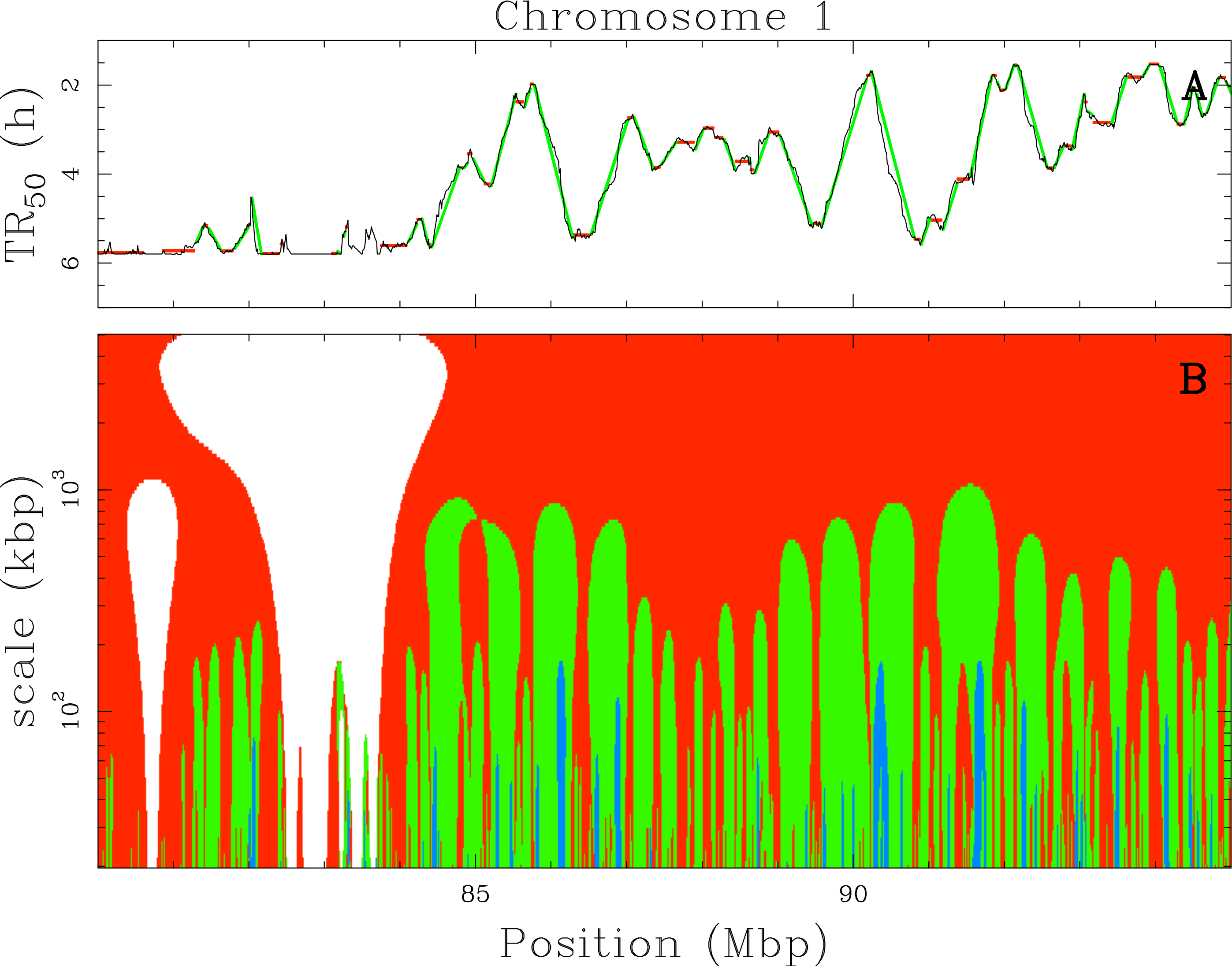

Chromosome 1
TR50 (h)
2
4
6
A
B
scale (kbp)
10^3
10^2
85
90
Position (Mbp)

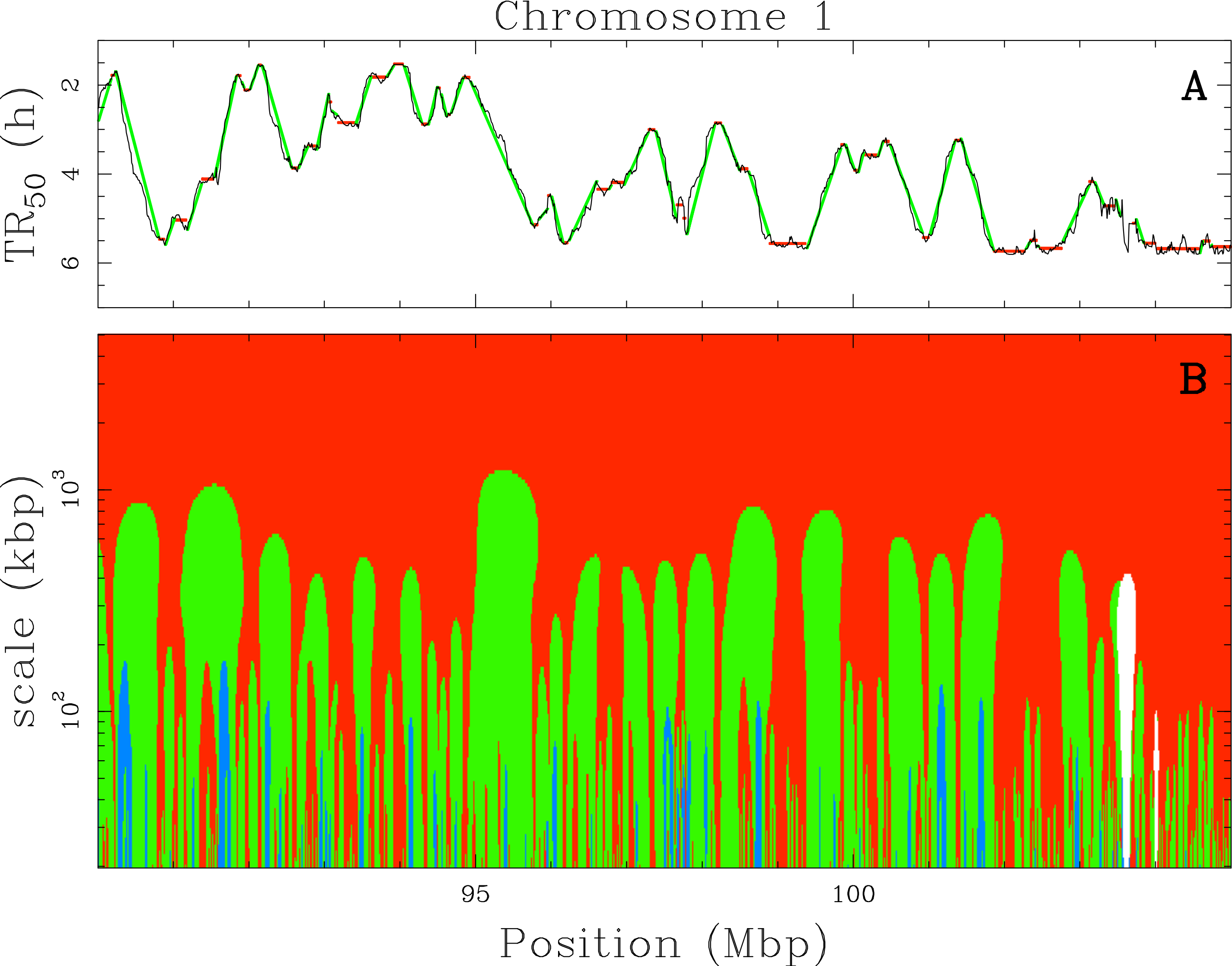

Chromosome 1
TR$_{50}$ (h)
2
4
6
A
B
scale (kbp)
10^3
10^2
95
100
Position (Mbp)

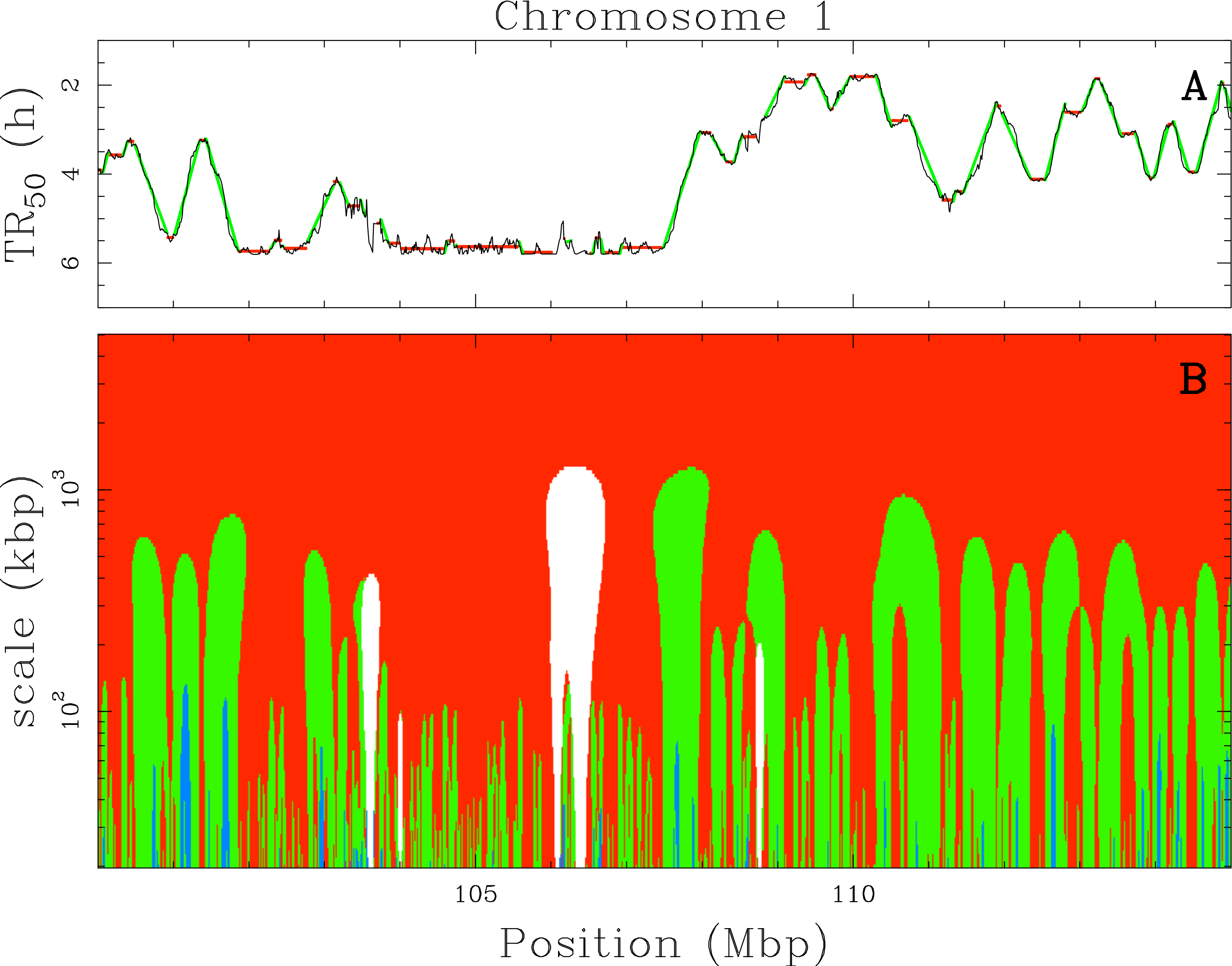

Chromosome 1
A
TR50 (h)
2
4
6
B
scale (kbp)
10^3
10^2
105
110
Position (Mbp)

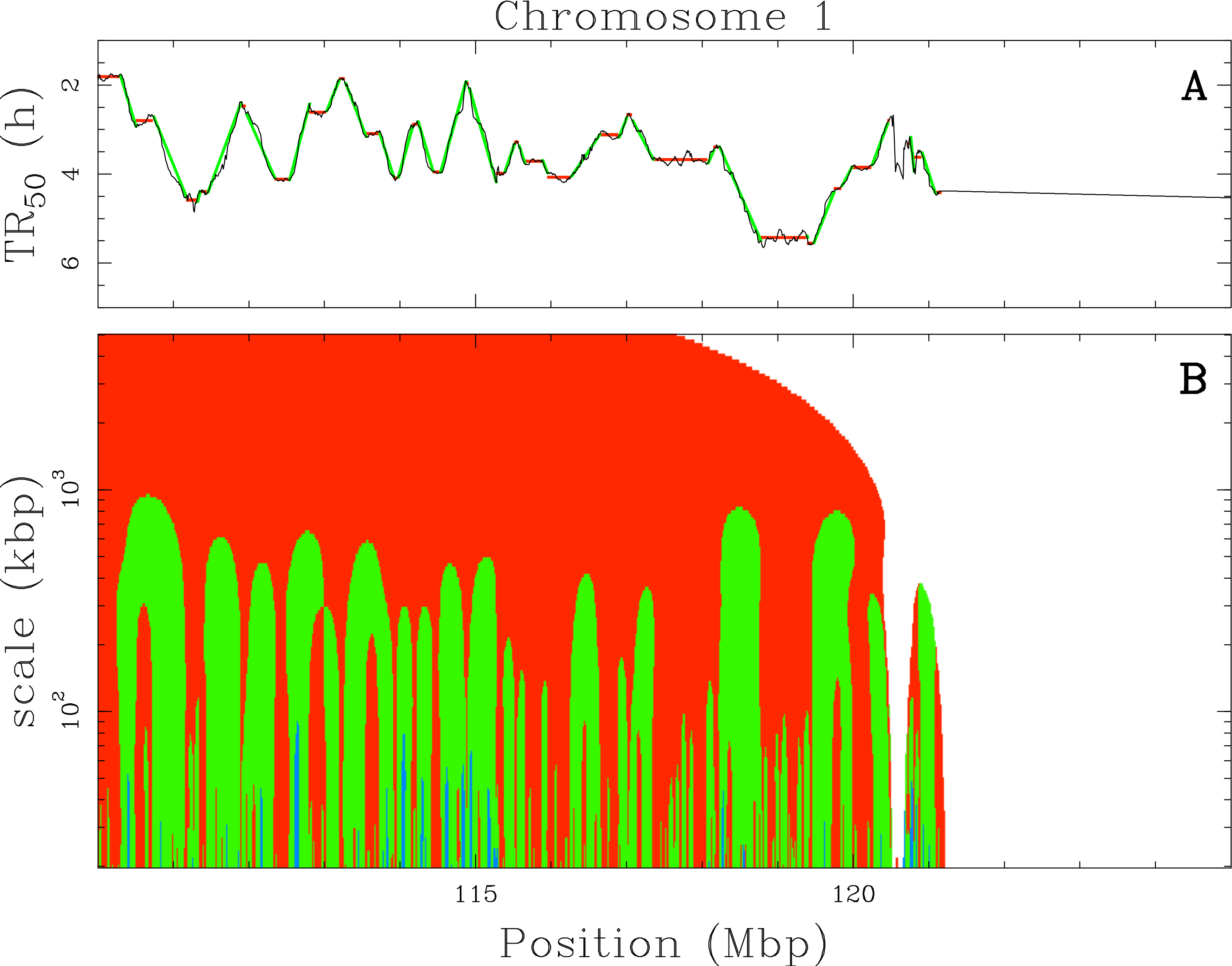
Chromosome 1
A
B
TR50 (h)
2
4
6
scale (kbp)
10^3
10^2
115
120
Position (Mbp)

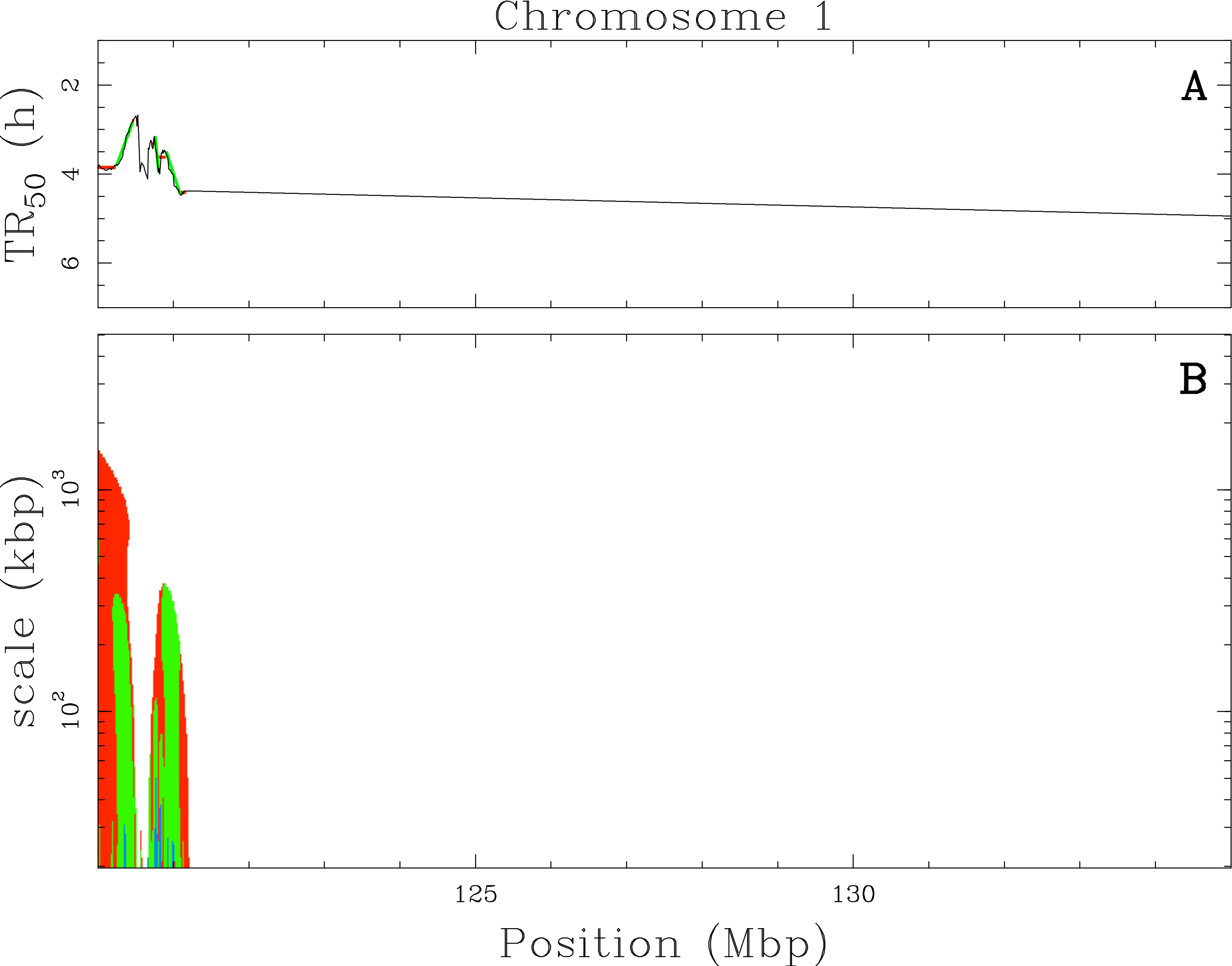
Chromosome 1
A
B
TR50 (h)
2
4
6
scale (kbp)
10^3
10^2
125
130
Position (Mbp)

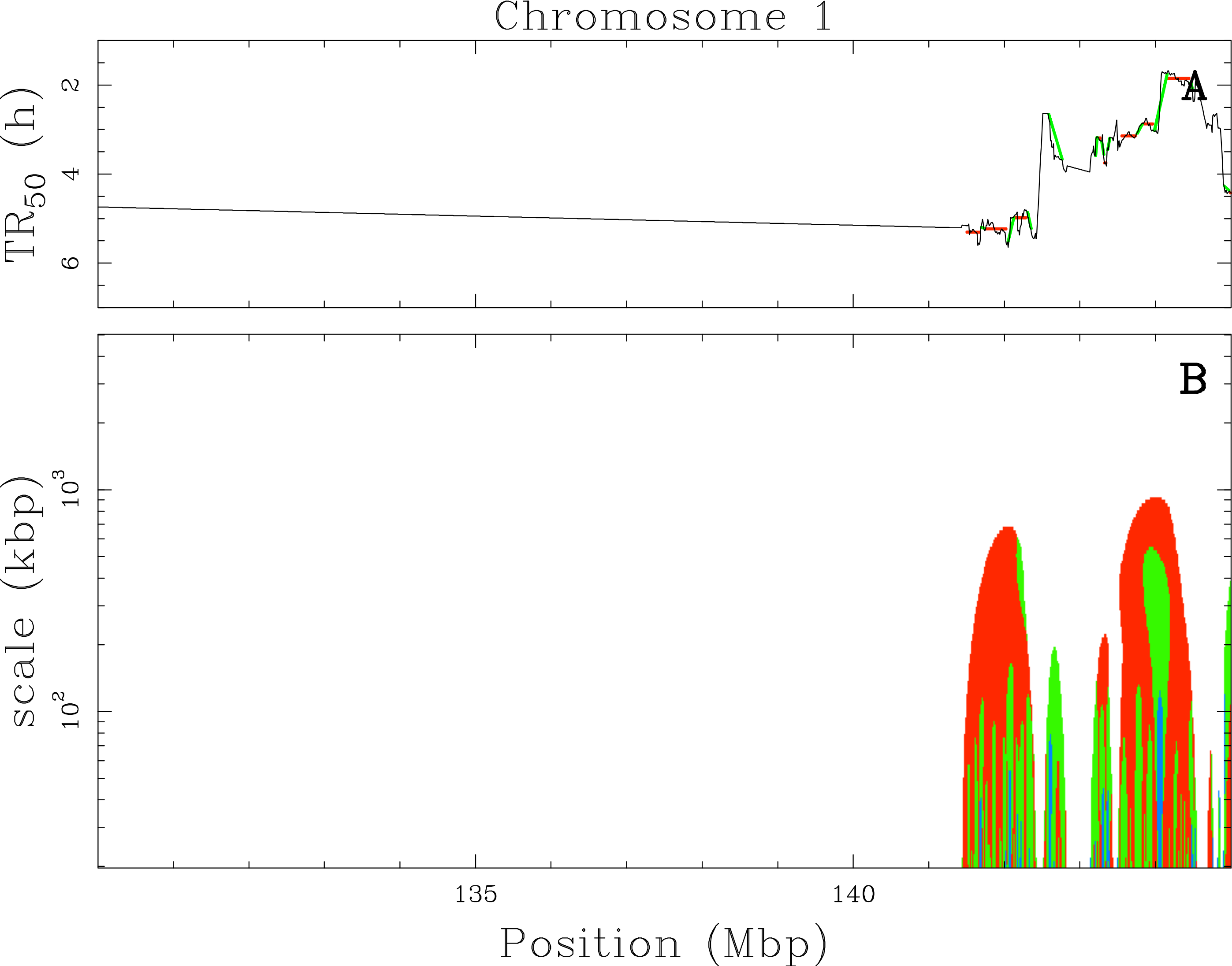
Chromosome 1
TR$_{50}$ (h)
2
4
6
A
B
scale (kbp)
10^3
10^2
135
140
Position (Mbp)

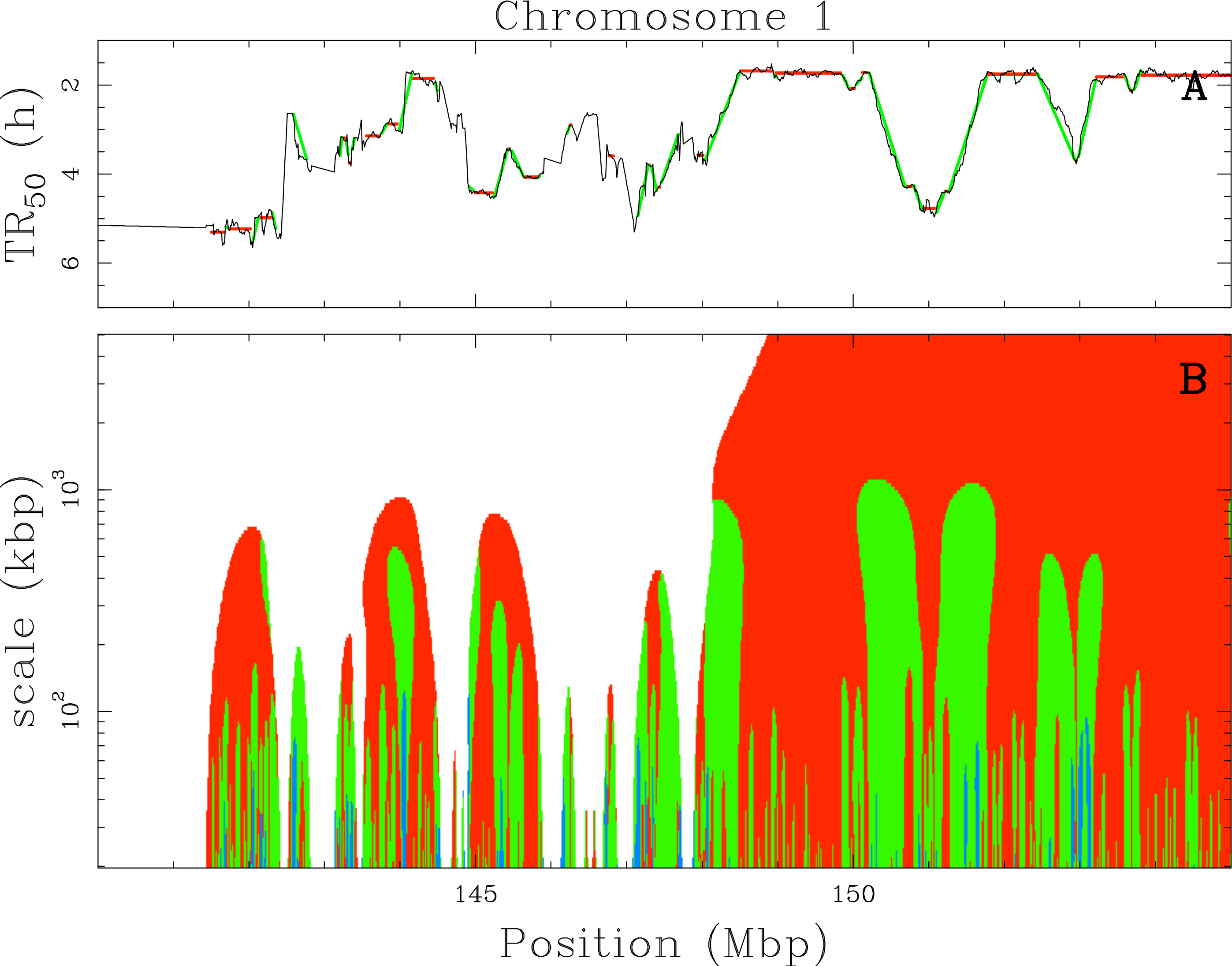

Chromosome 1
A
B
TR50 (h)
2
4
6
scale (kbp)
10^3
10^2
145
150
Position (Mbp)

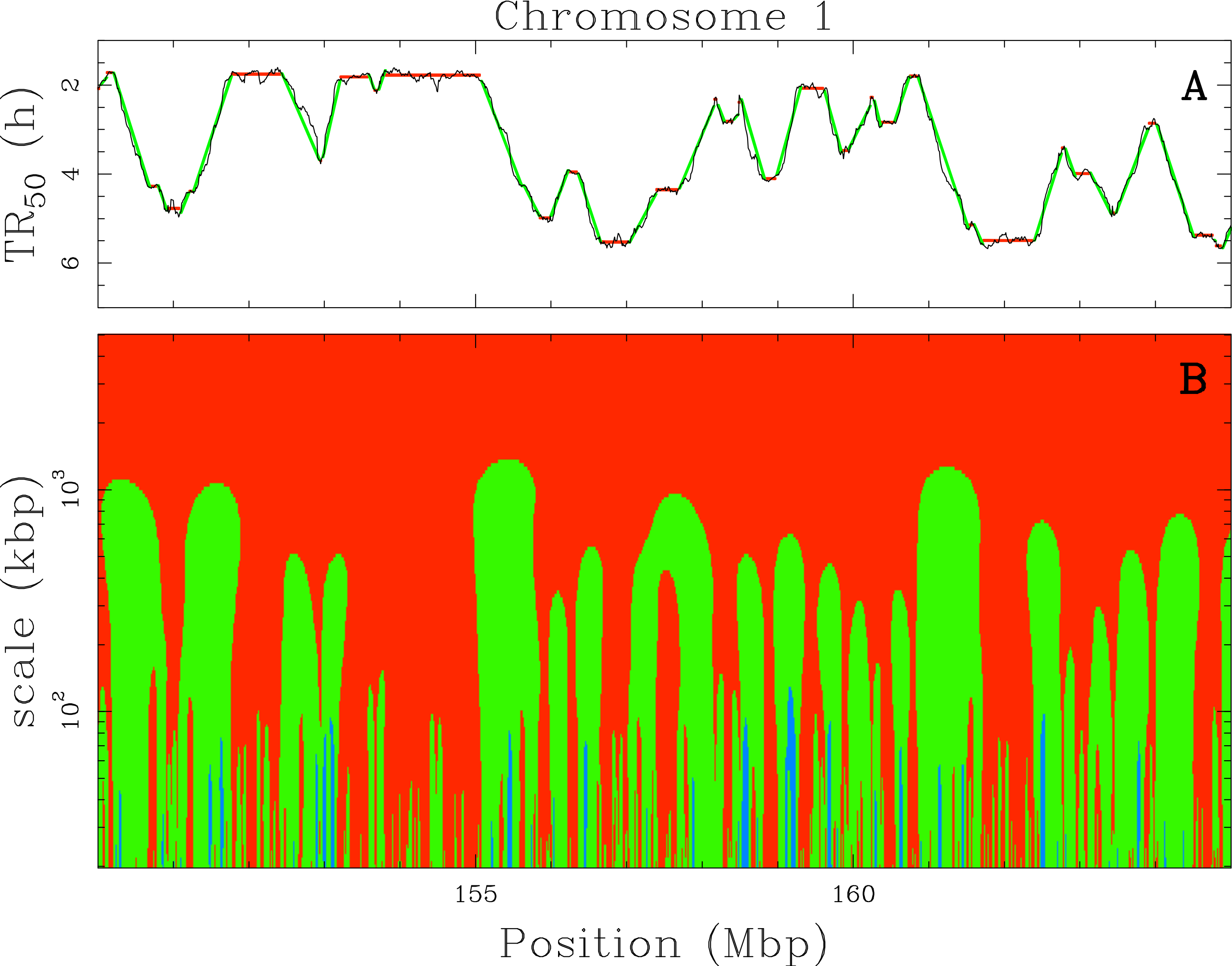

Chromosome 1
TR50 (h)
2
4
6
A
B
scale (kbp)
10^3
10^2
155
160
Position (Mbp)

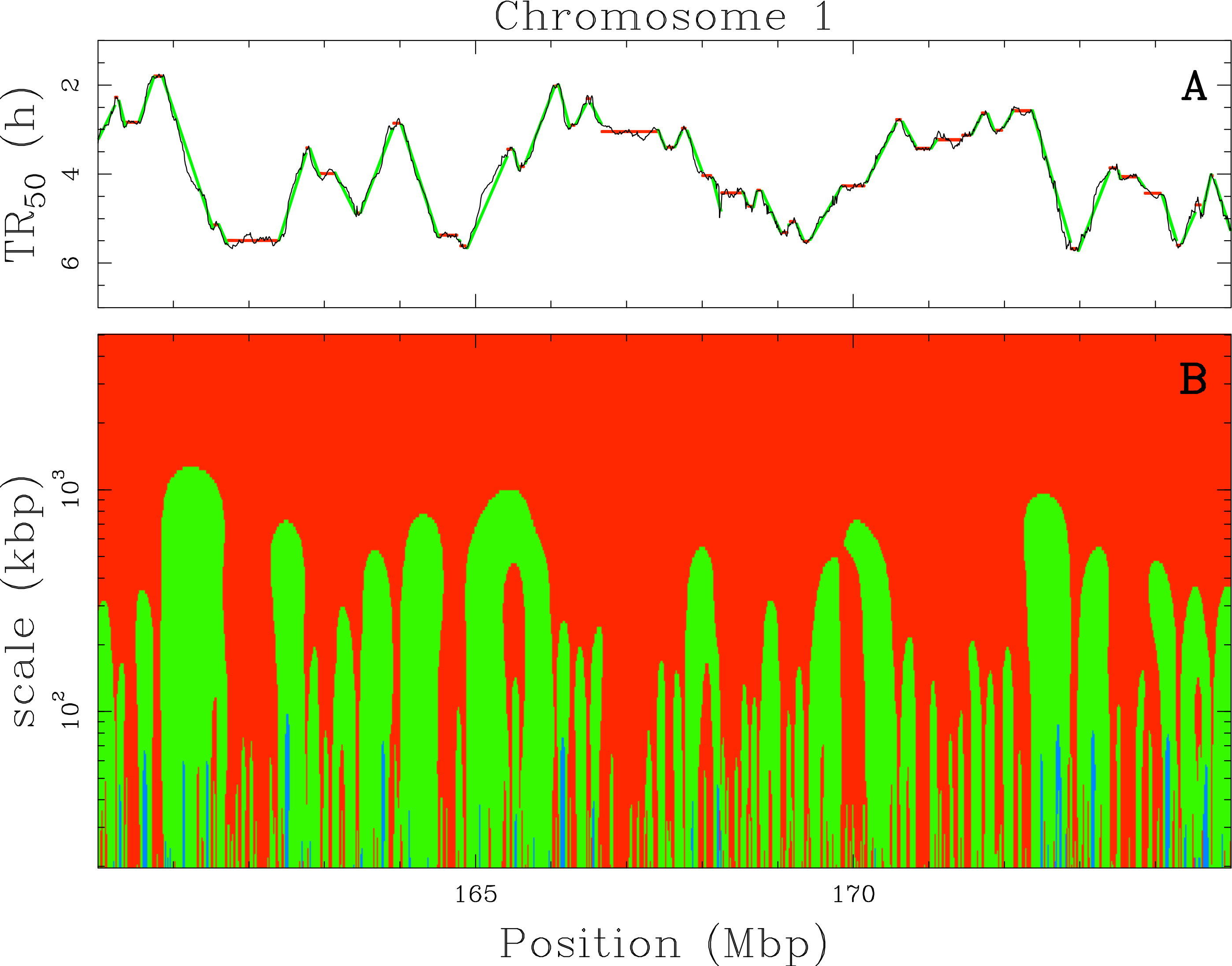

Chromosome 1
A
TR50 (h)
2
4
6
B
scale (kbp)
10^3
10^2
165
170
Position (Mbp)

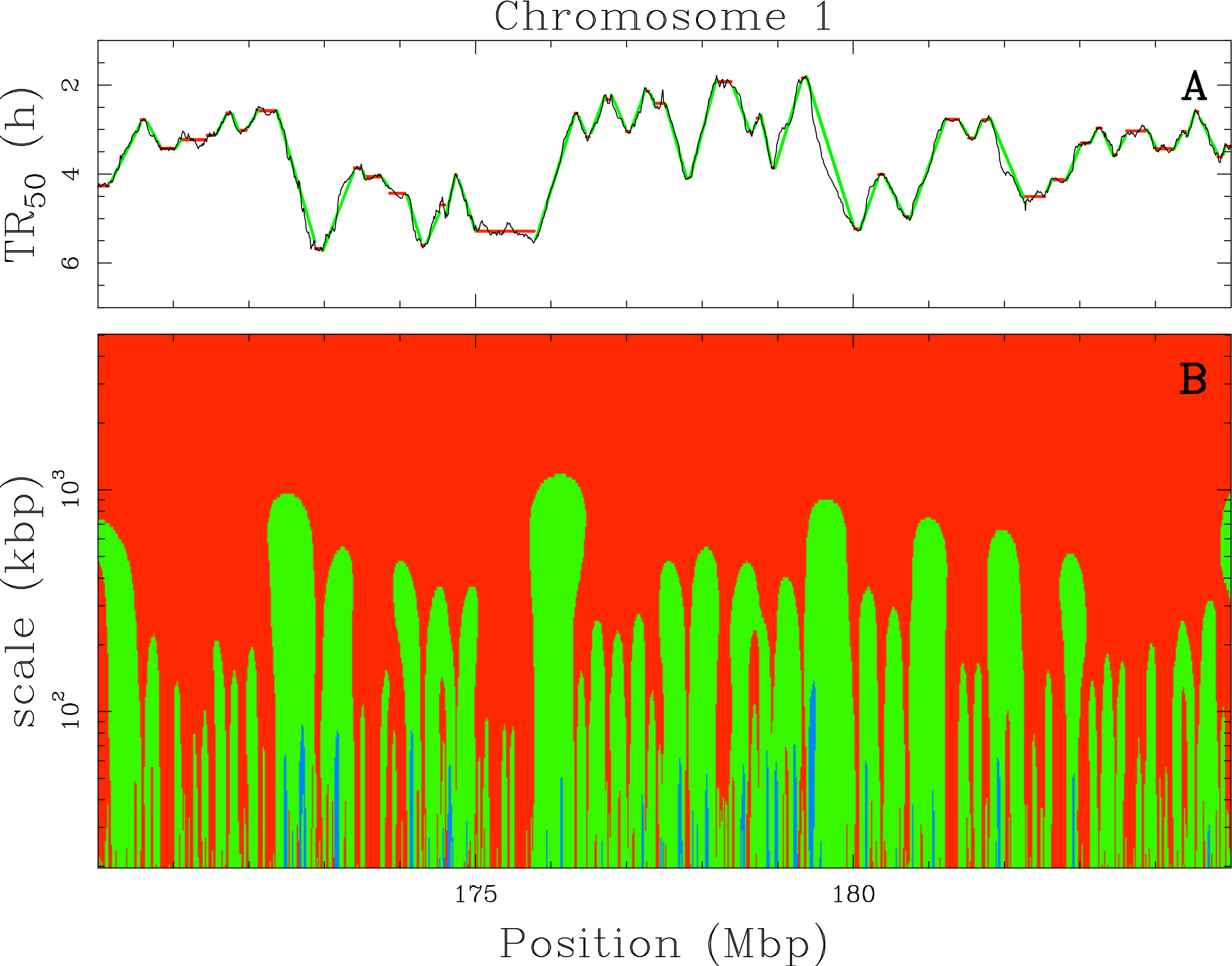

Chromosome 1
A
B
TR_{50} (h)
2
4
6
scale (kbp)
10^3
10^2
175
180
Position (Mbp)

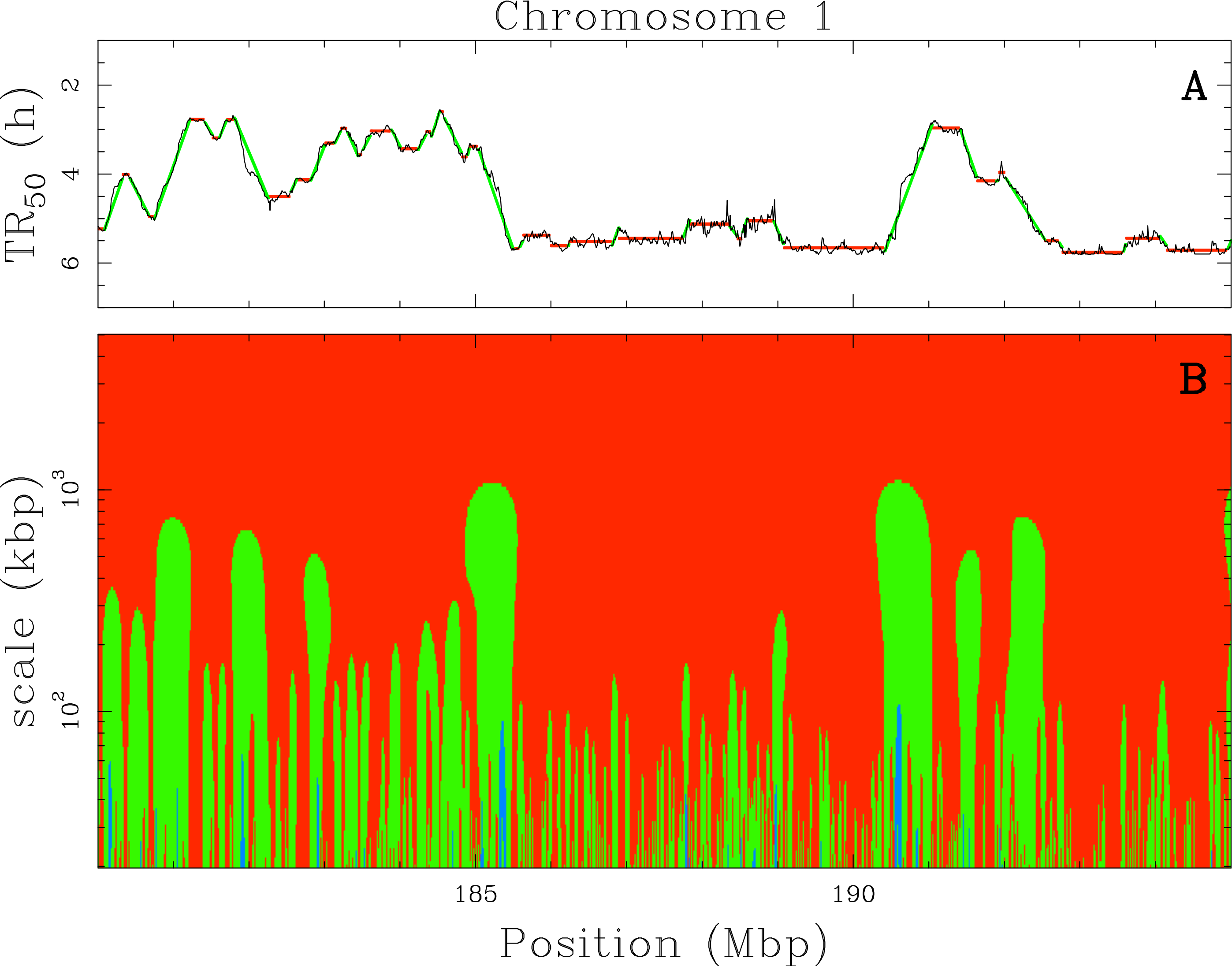

Chromosome 1
A
TR50 (h)
2
4
6
B
scale (kbp)
10^3
10^2
185
190
Position (Mbp)

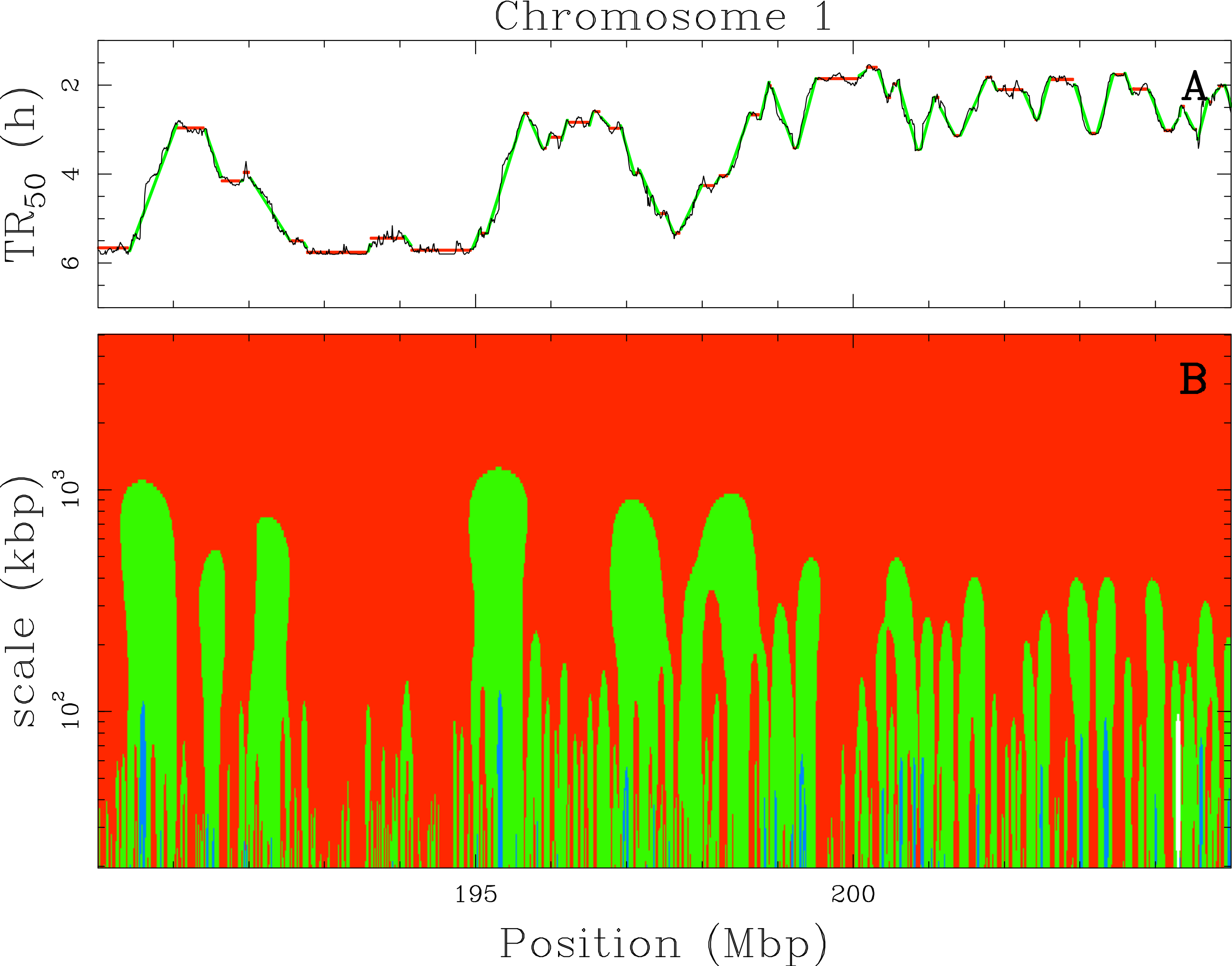

Chromosome 1
TR50 (h)
2
4
6
A
B
scale (kbp)
10^3
10^2
195
200
Position (Mbp)

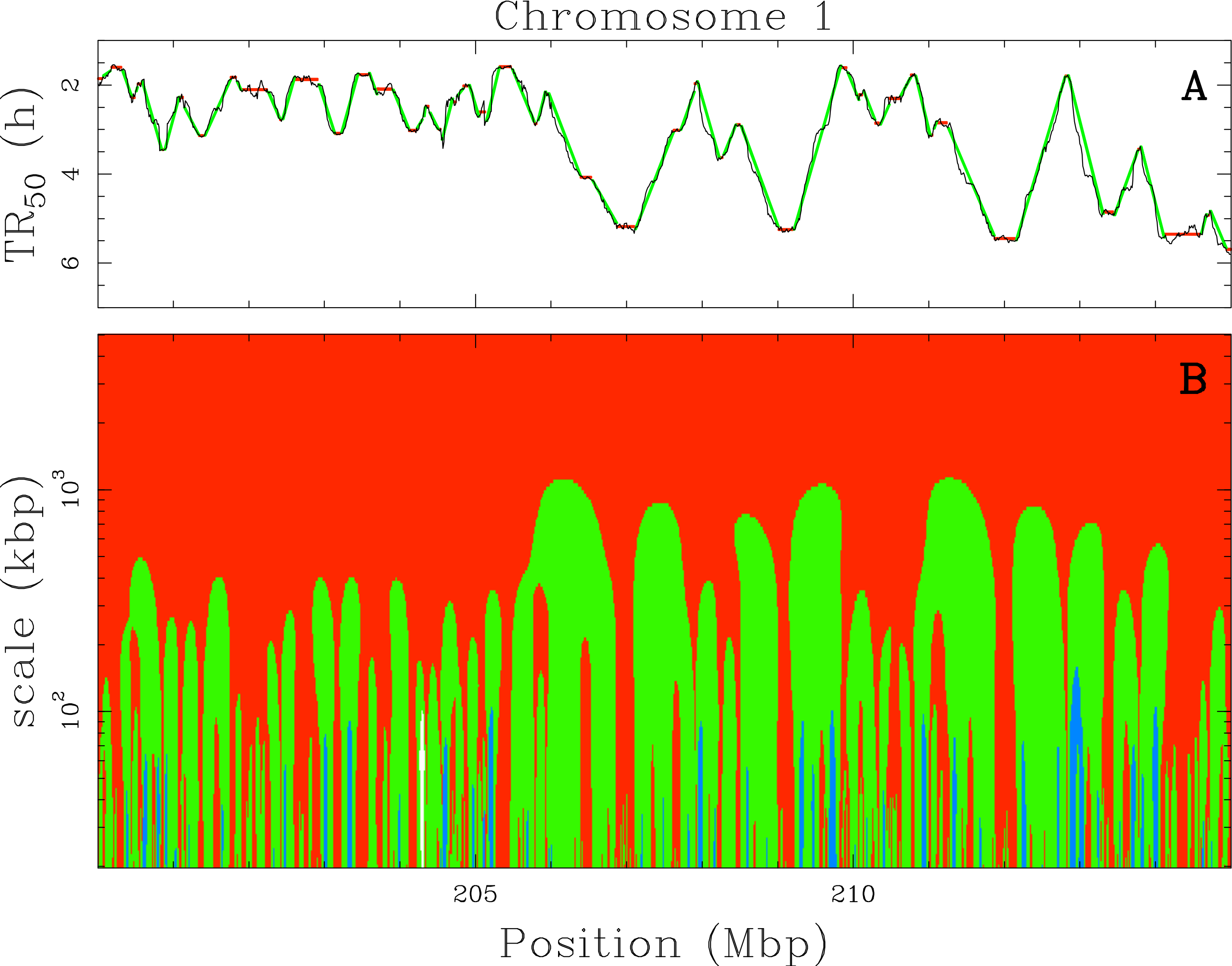

Chromosome 1
A
B
TR50 (h)
2
4
6
scale (kbp)
10^3
10^2
205
210
Position (Mbp)

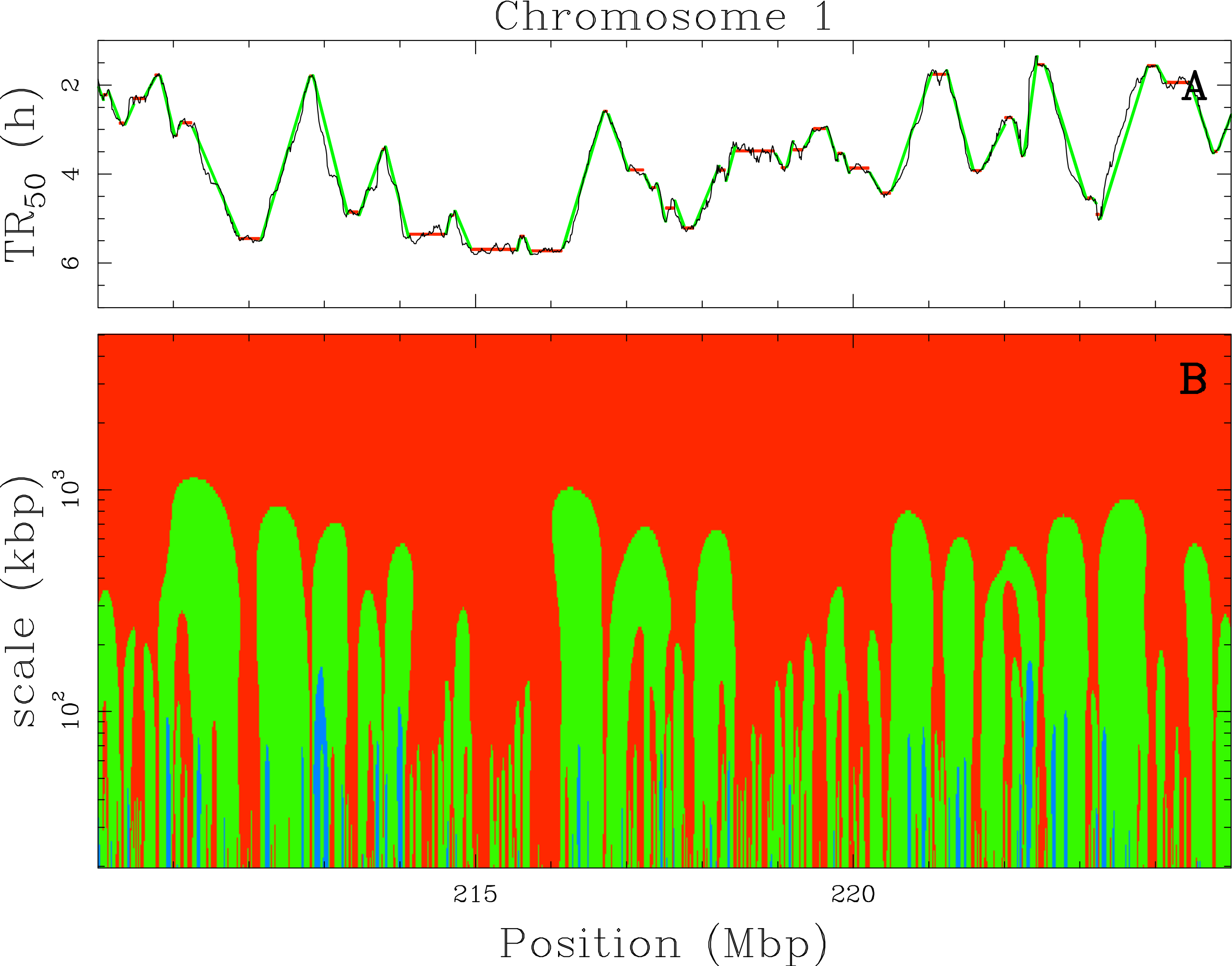

Chromosome 1
A
B
TR50 (h)
2
4
6
scale (kbp)
10^2
10^3
215
220
Position (Mbp)

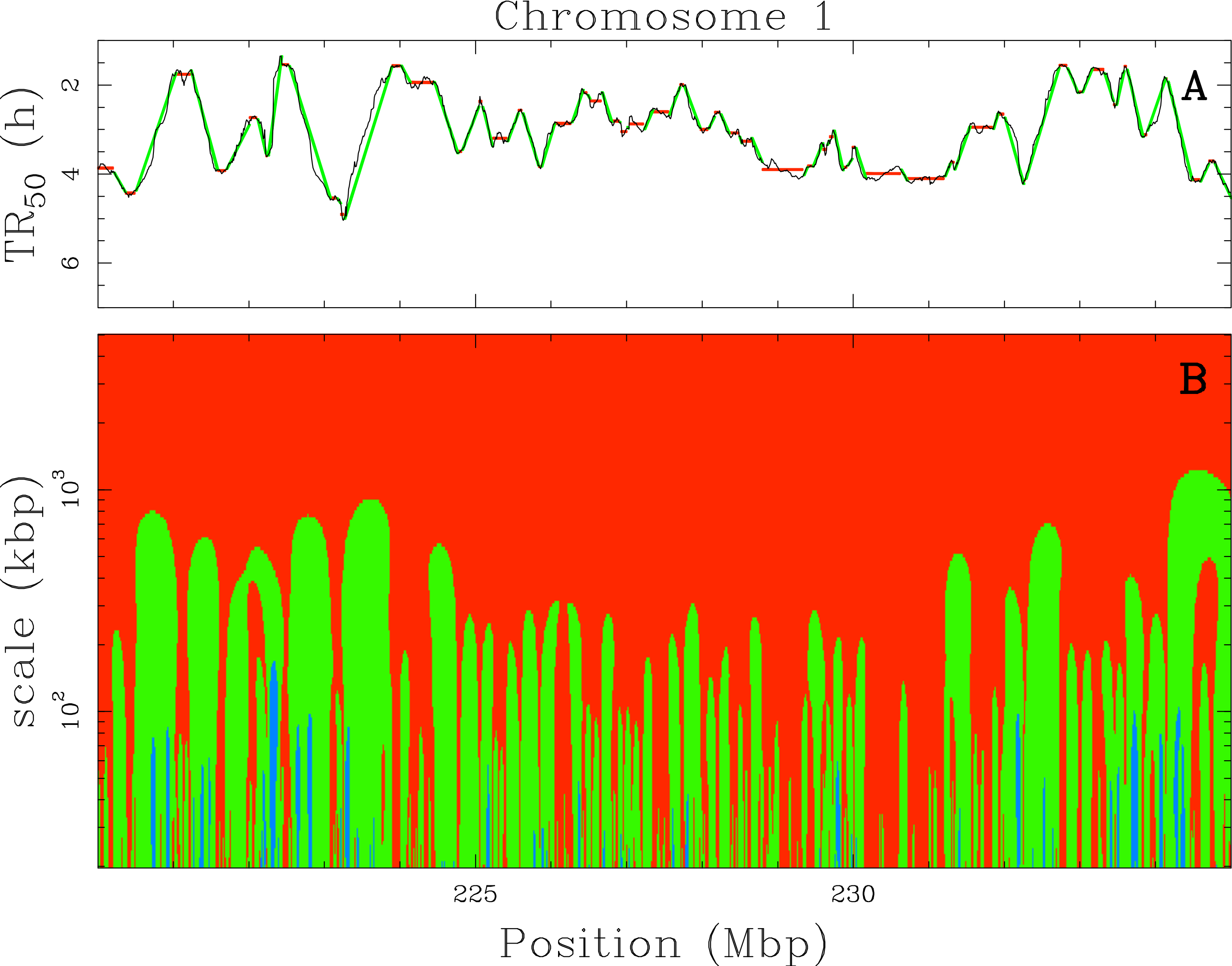

Chromosome 1
A
B
TR50 (h)
2
4
6
scale (kbp)
10^3
10^2
225
230
Position (Mbp)

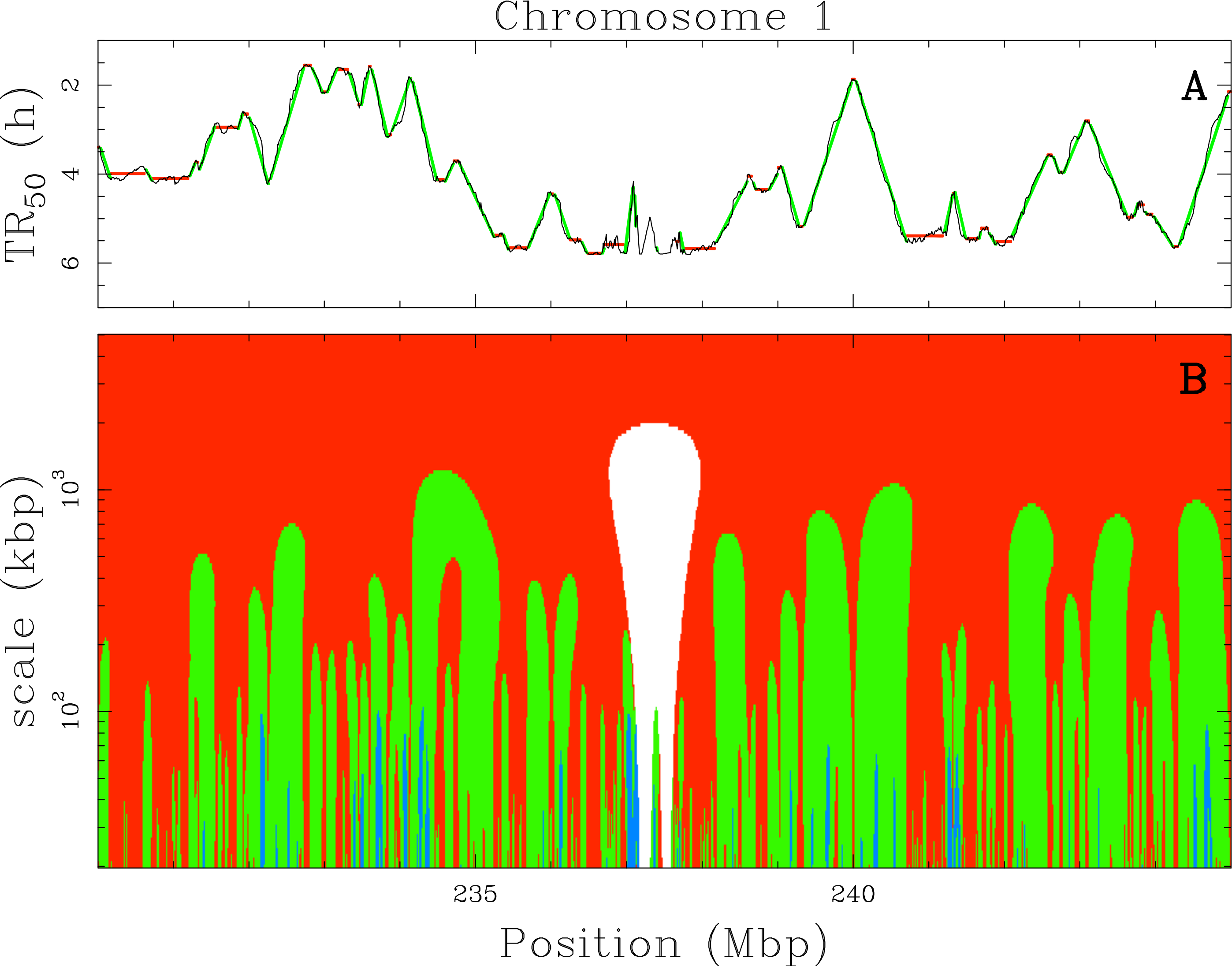
Chromosome 1
A
B
TR_{50} (h)
2
4
6
scale (kbp)
10^3
10^2
235
240
Position (Mbp)

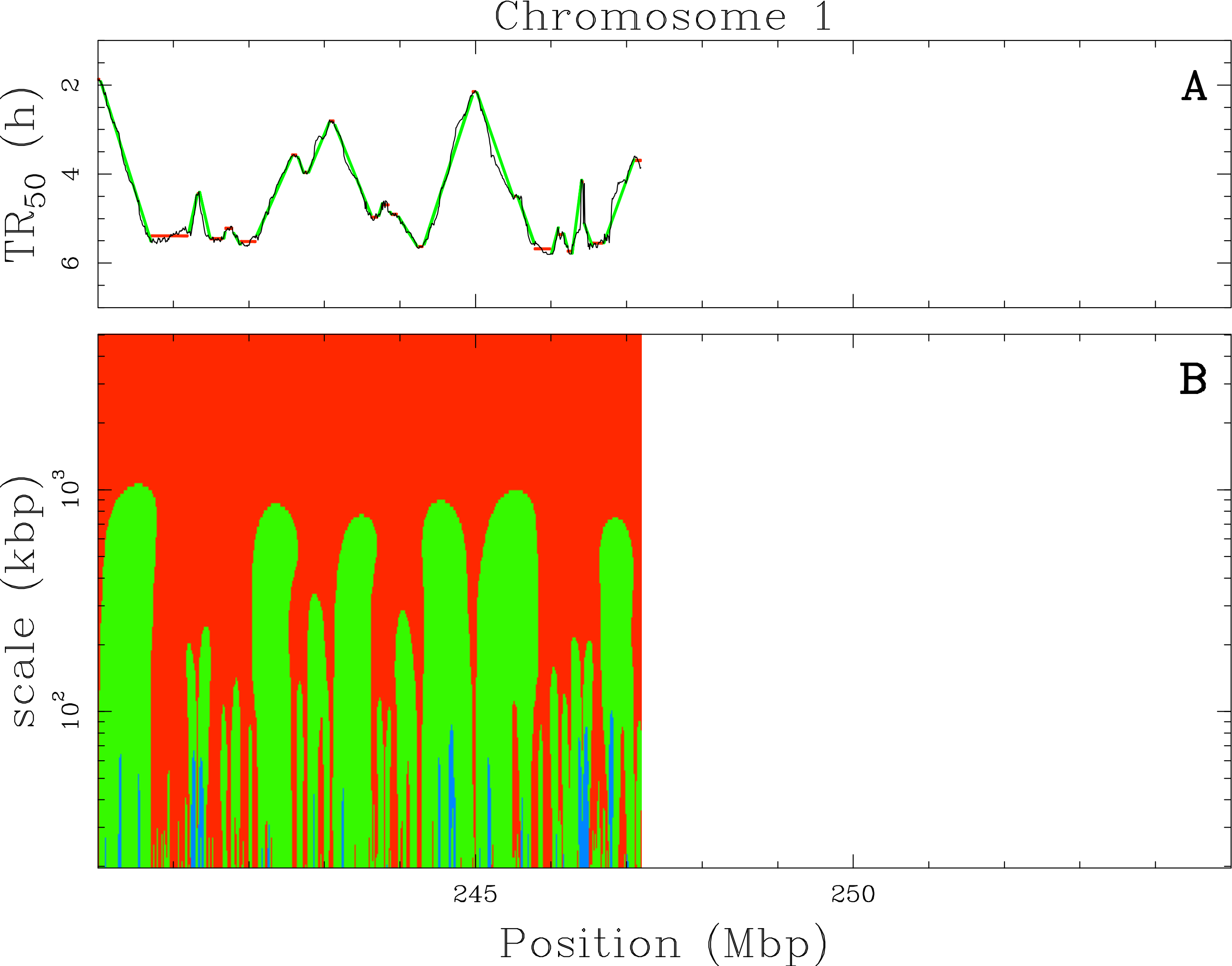

Chromosome 1
TR50 (h)
2
4
6
A
scale (kbp)
10^3
10^2
B
245
250
Position (Mbp)

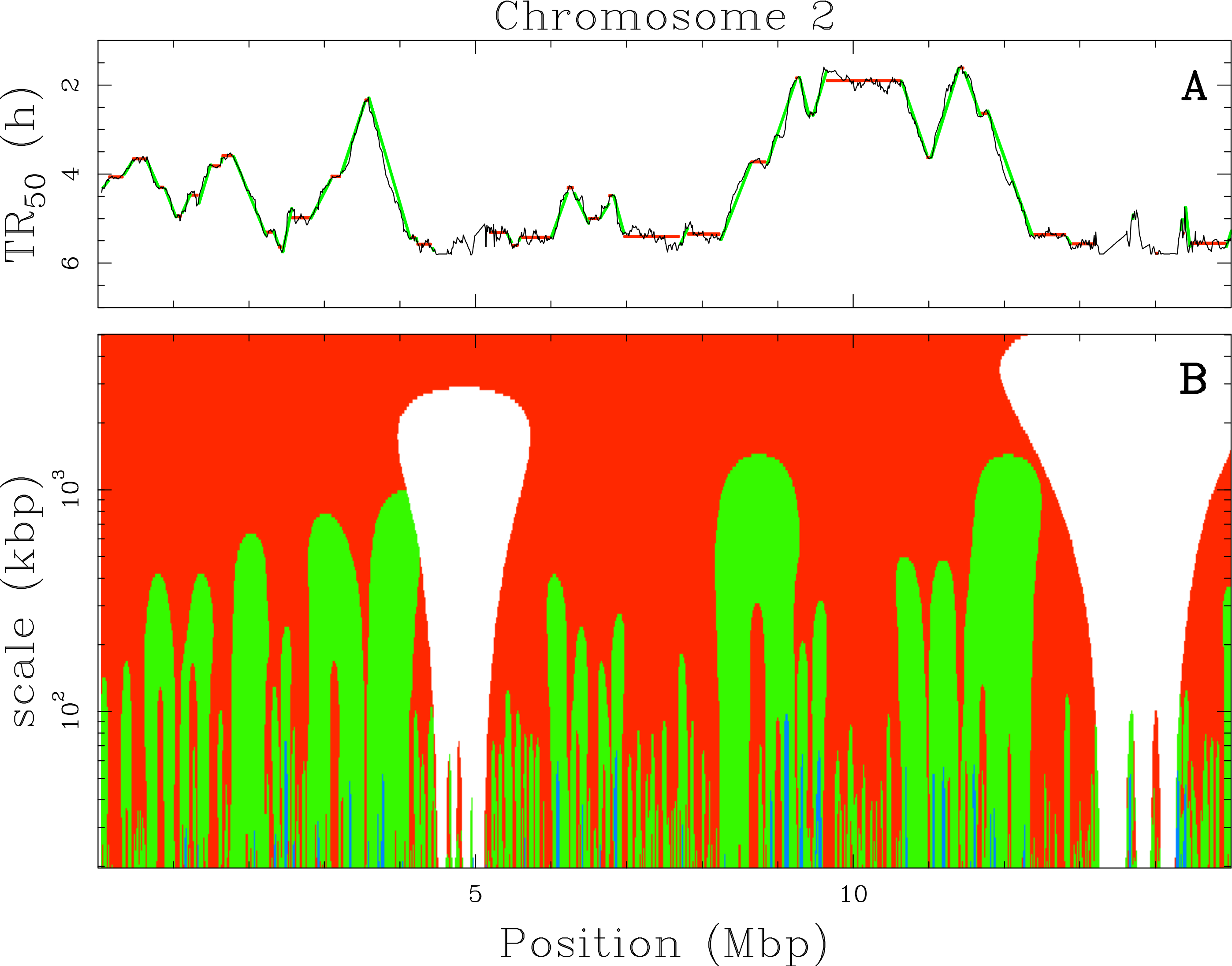
Chromosome 2
TR50 (h)
2
4
6
A
B
scale (kbp)
10^3
10^2
5
10
Position (Mbp)

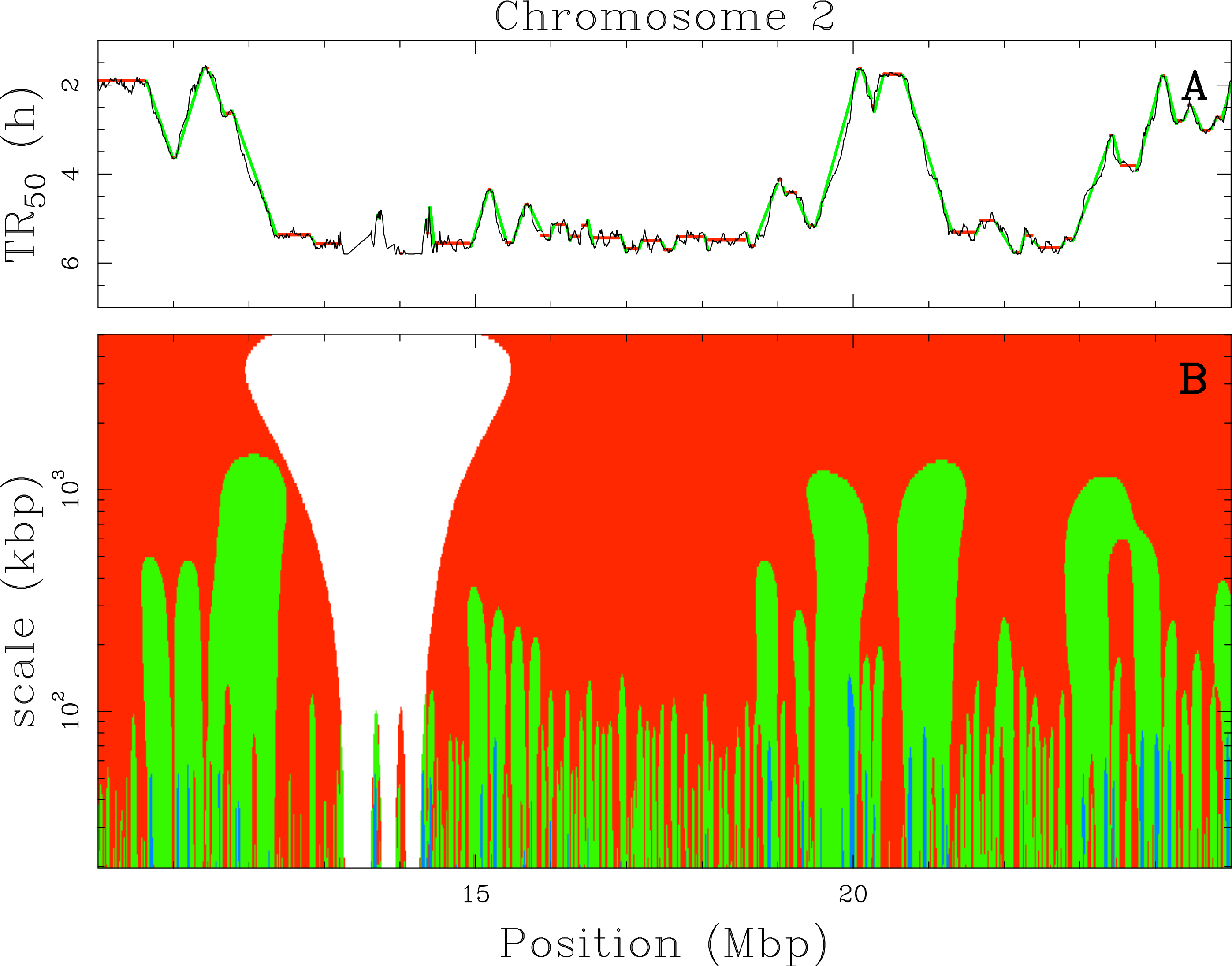

Chromosome 2
A
B
TR50 (h)
2
4
6
scale (kbp)
10^3
10^2
15
20
Position (Mbp)

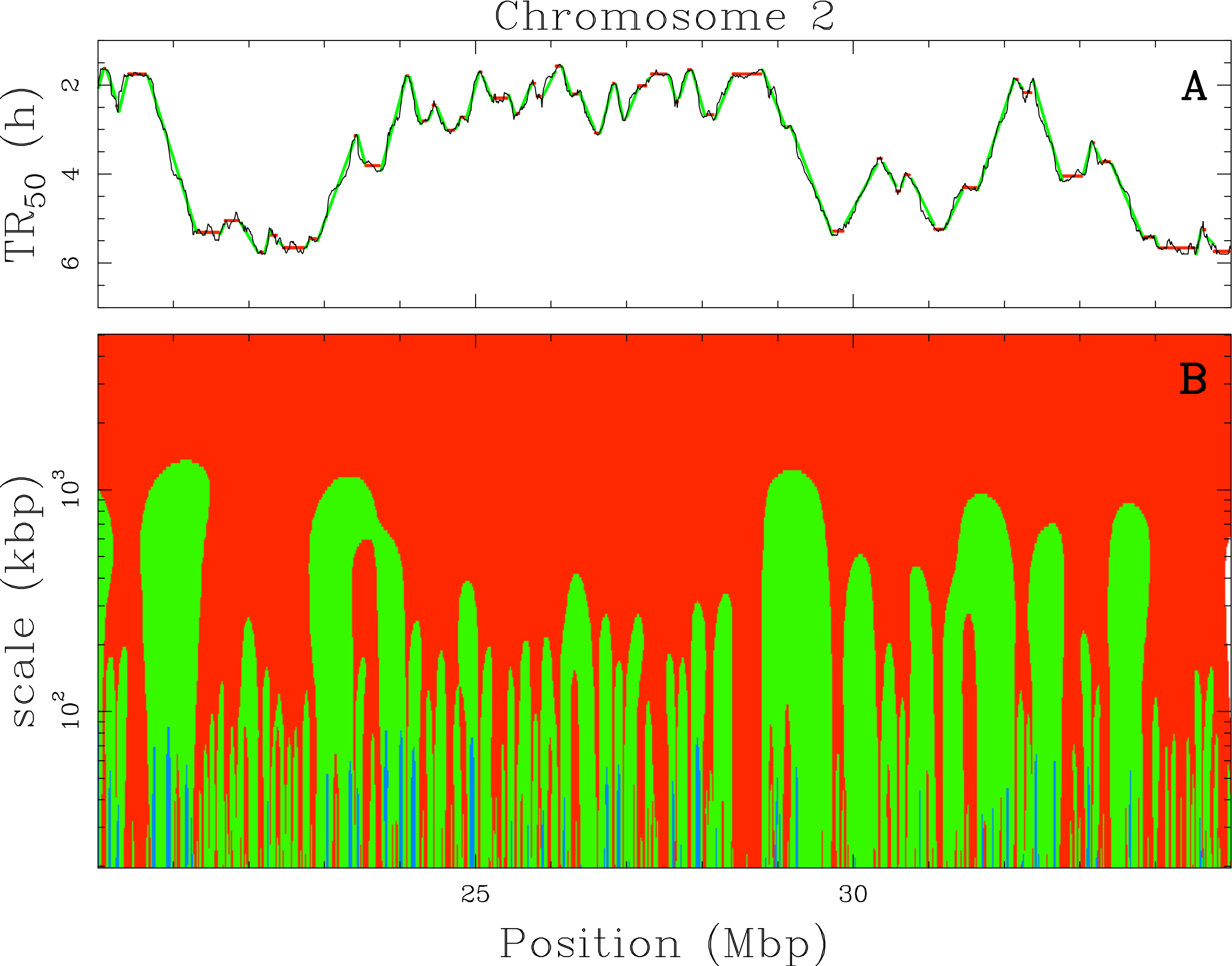

Chromosome 2
A
B
TR50 (h)
2
4
6
scale (kbp)
10^3
10^2
25
30
Position (Mbp)

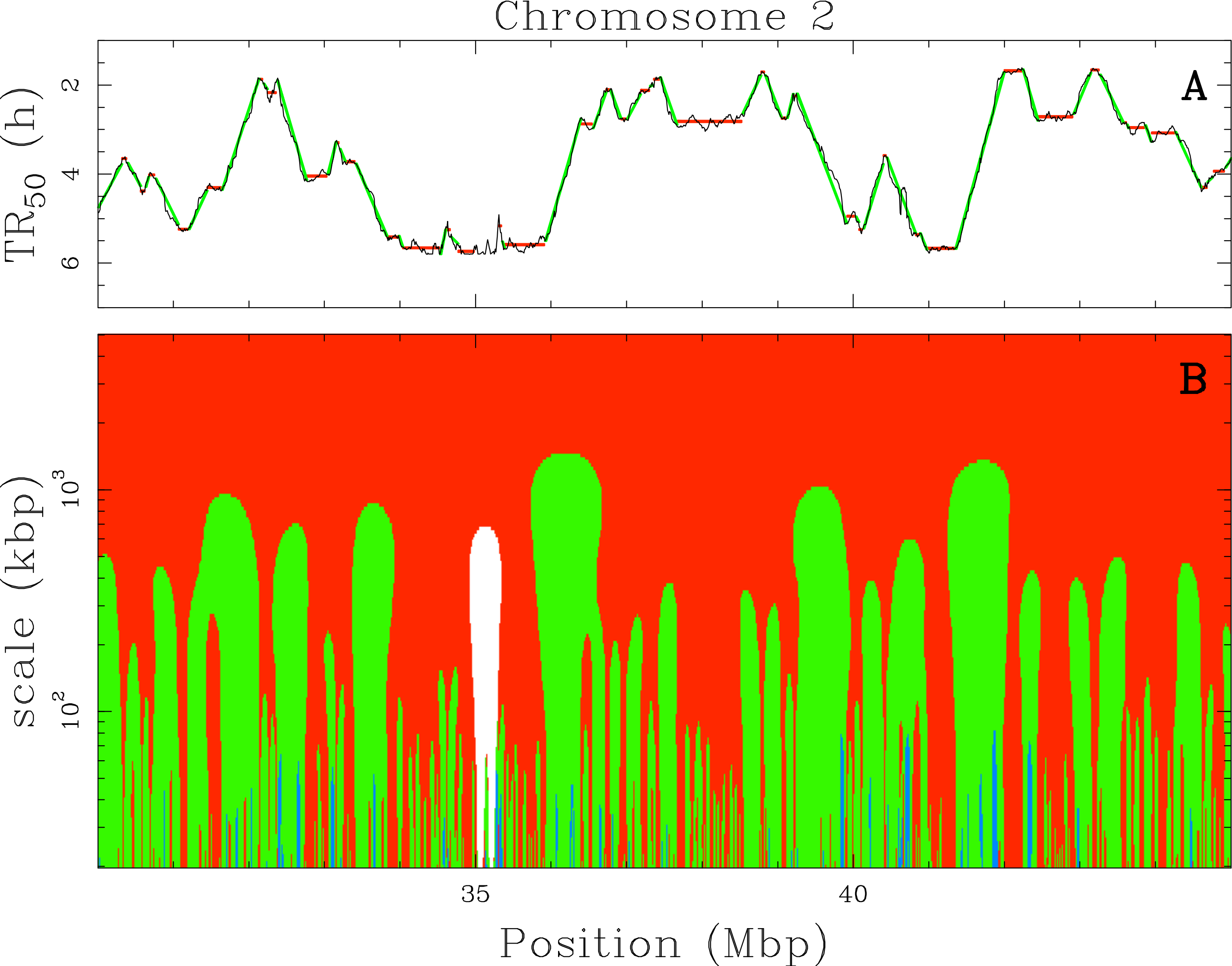

Chromosome 2
A
B
TR50 (h)
2
4
6
scale (kbp)
10^3
10^2
35
40
Position (Mbp)

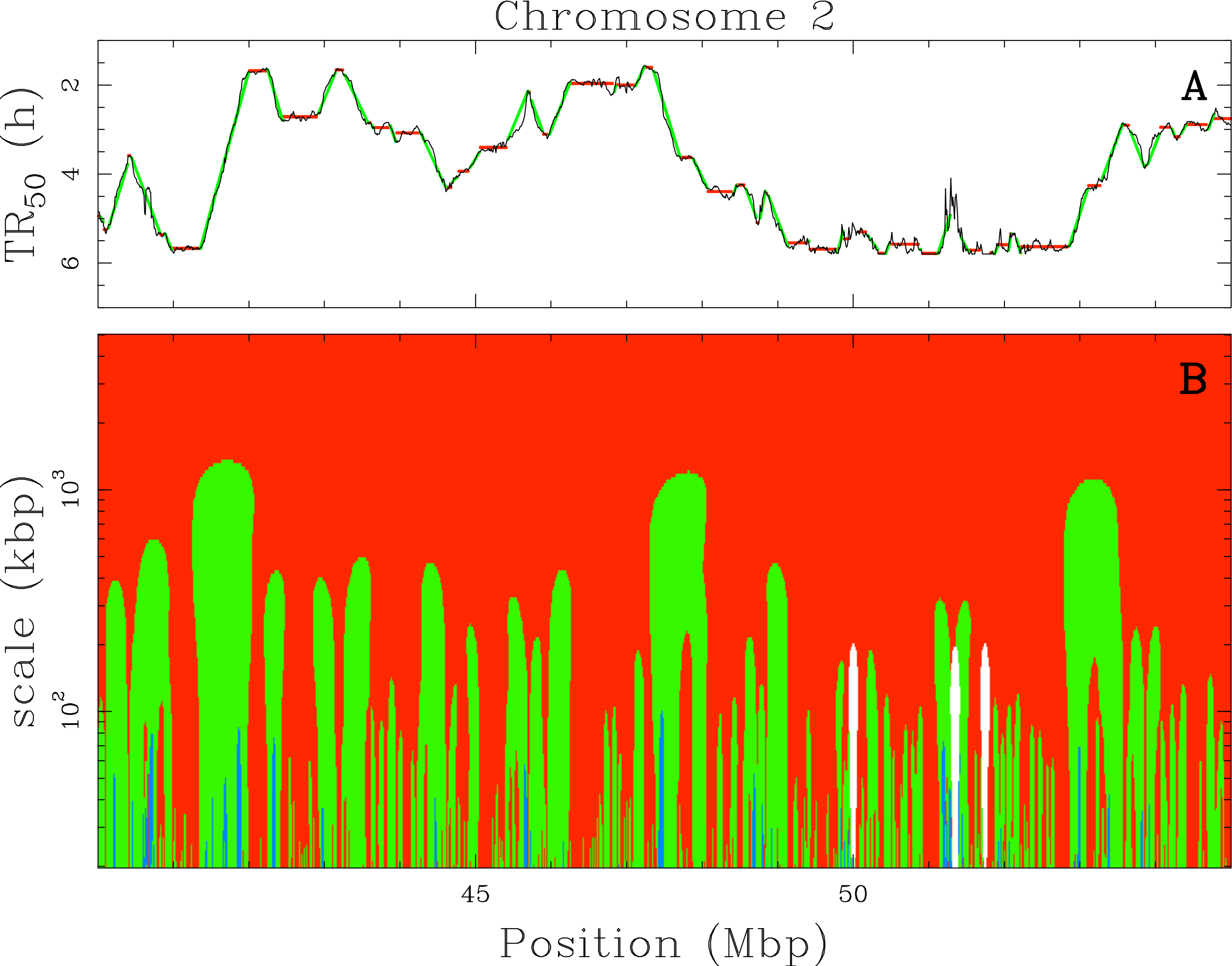

Chromosome 2
A
B
TR50 (h)
2
4
6
scale (kbp)
10^3
10^2
45
50
Position (Mbp)

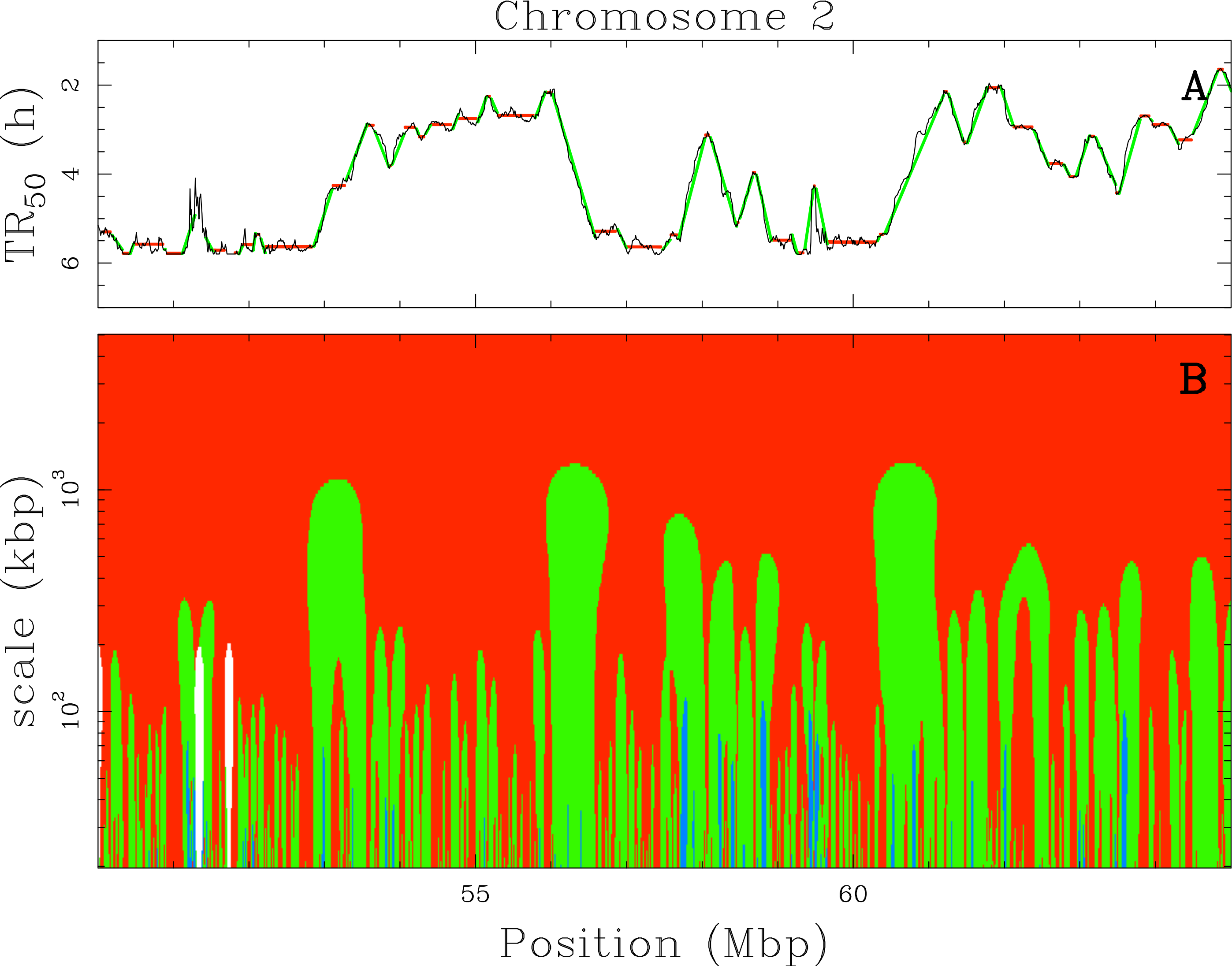

Chromosome 2
A
TR_{50} (h)
2
4
6
B
scale (kbp)
10^3
10^2
55
60
Position (Mbp)

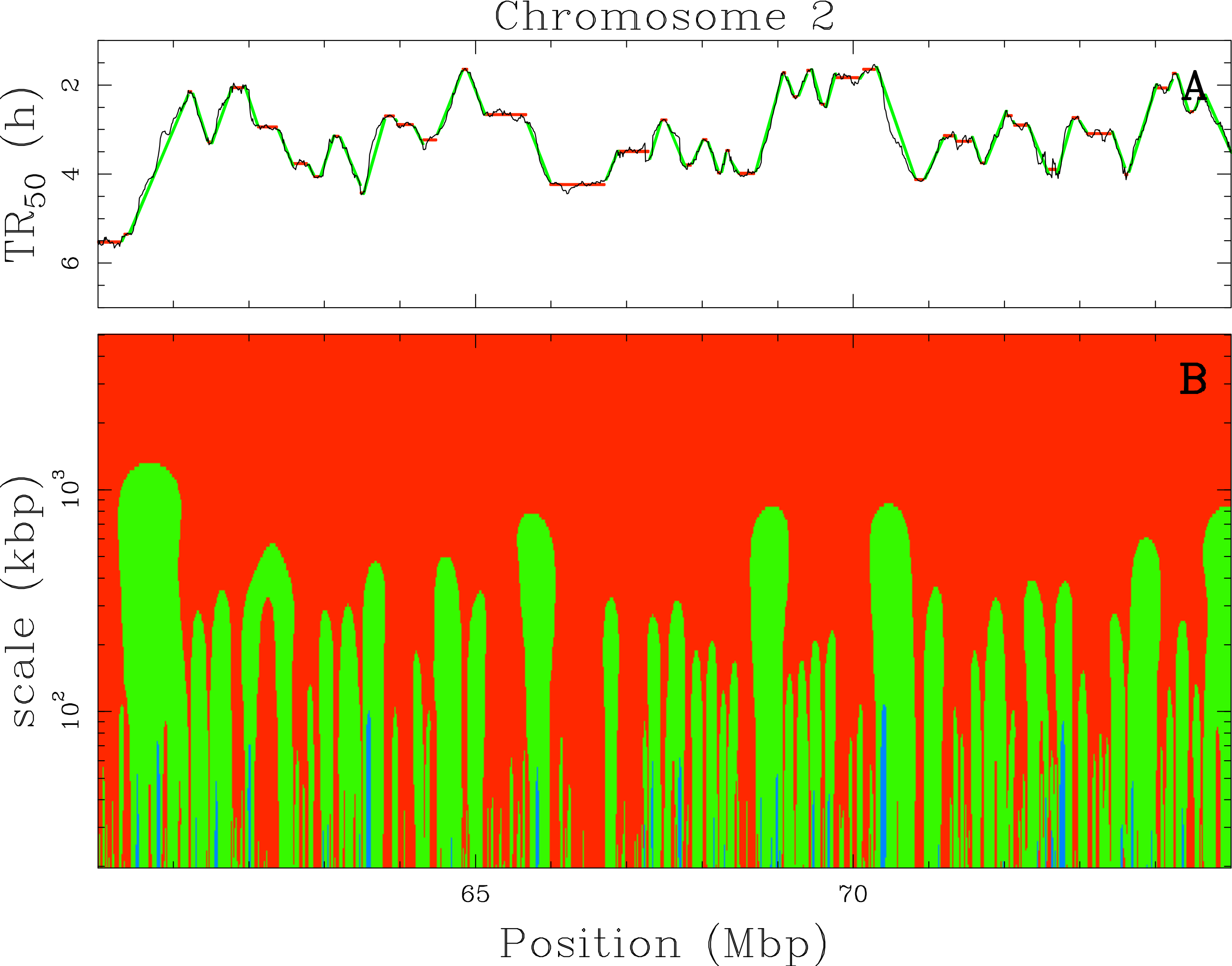

Chromosome 2
A
B
TR50 (h)
2
4
6
scale (kbp)
10^3
10^2
65
70
Position (Mbp)

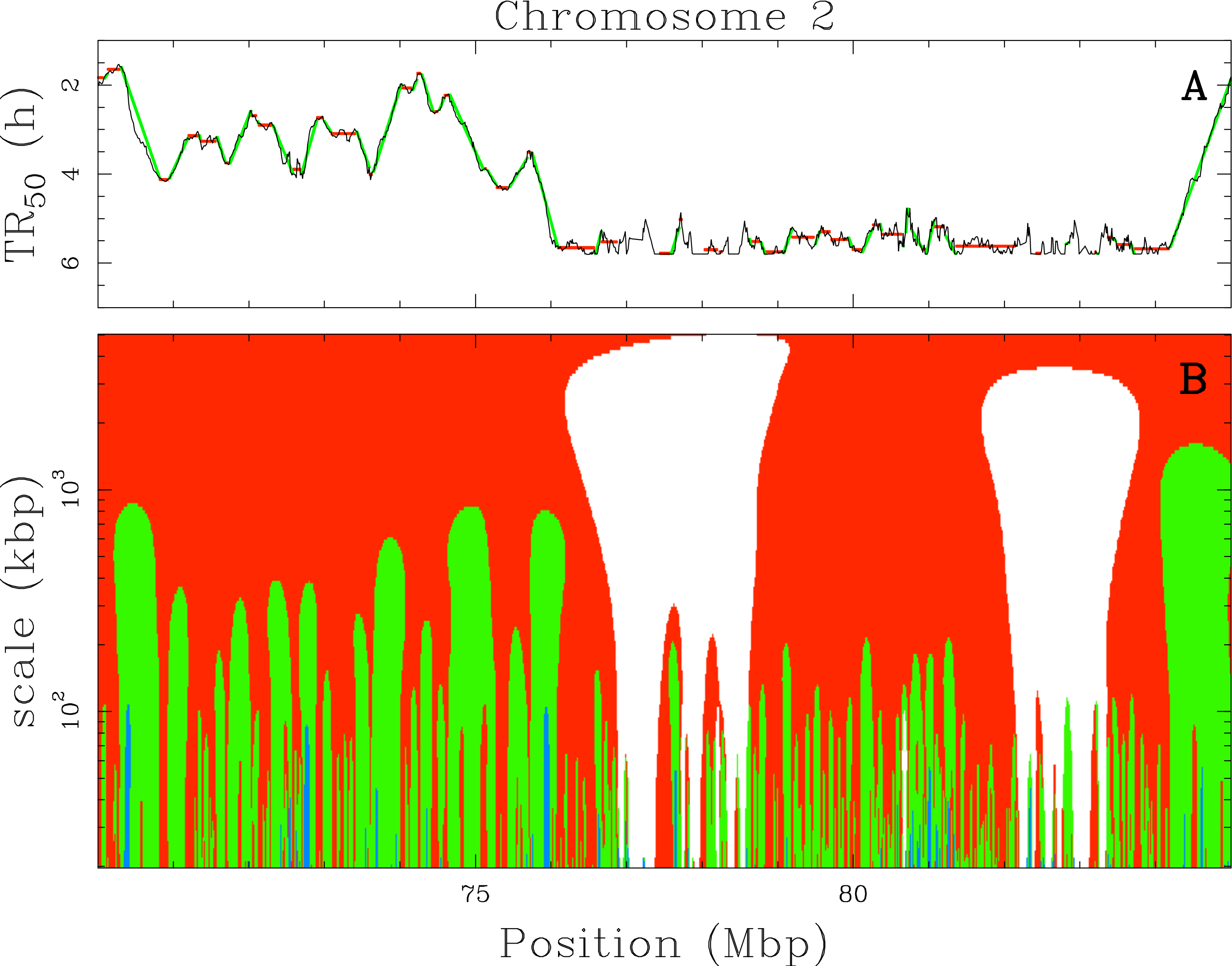
Chromosome 2
A
TR_{50} (h)
2
4
6
B
scale (kbp)
10^3
10^2
75
80
Position (Mbp)

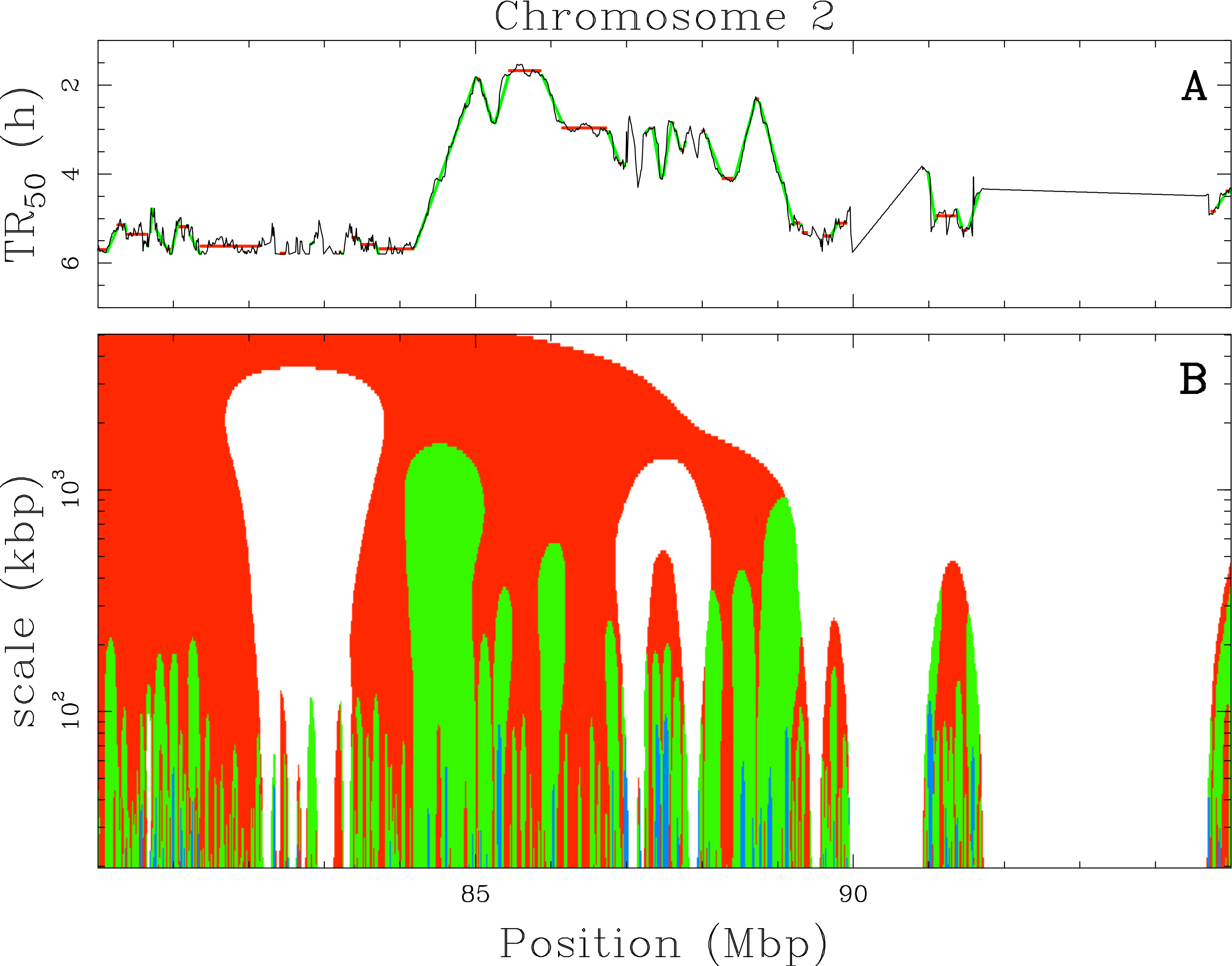
Chromosome 2
A
B
TR50 (h)
2
4
6
scale (kbp)
10^3
10^2
85
90
Position (Mbp)

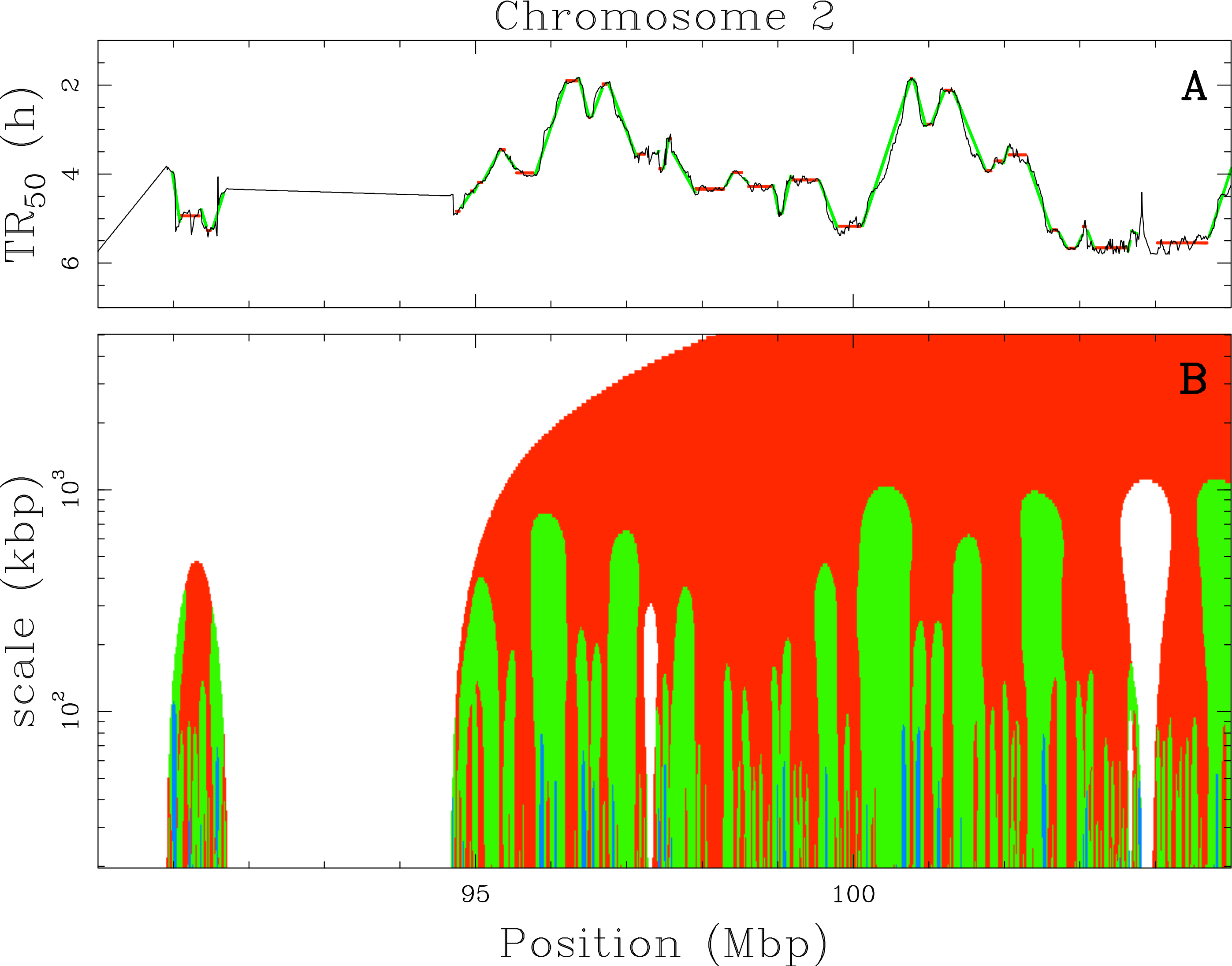

Chromosome 2
A
B
TR_{50} (h)
2
4
6
scale (kbp)
10^3
10^2
95
100
Position (Mbp)

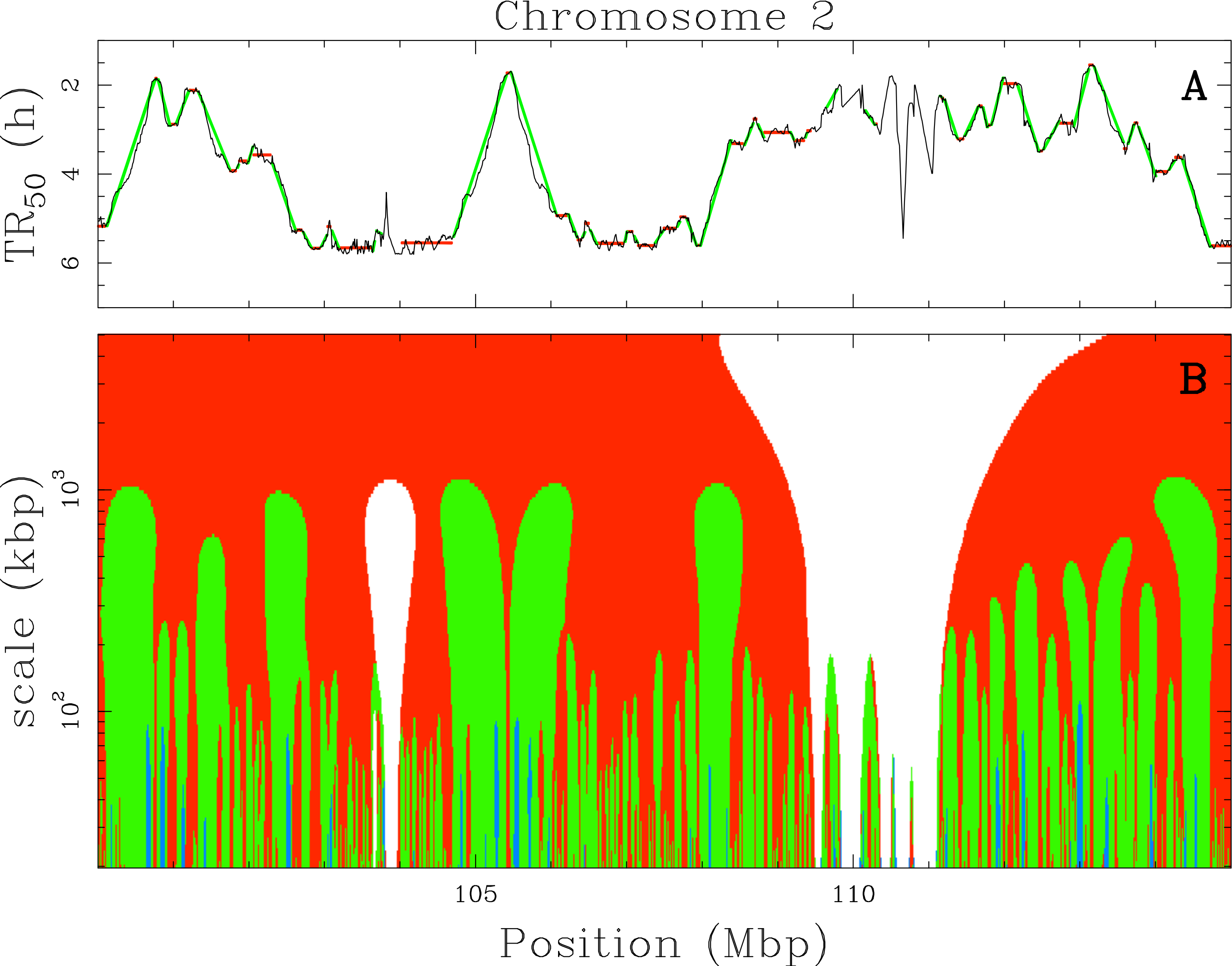

Chromosome 2
A
TR_{50} (h)
2
4
6
B
scale (kbp)
10^3
10^2
105
110
Position (Mbp)

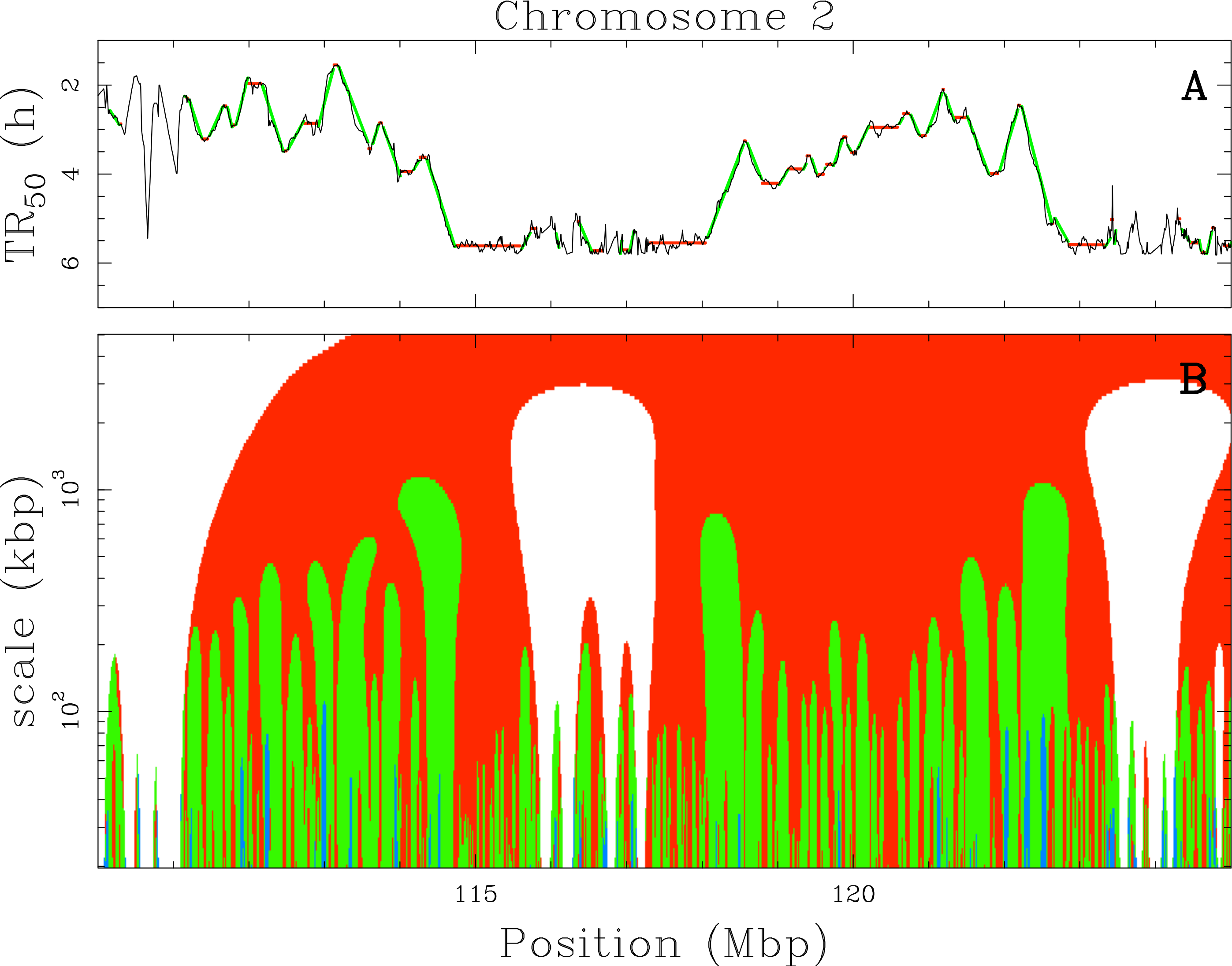

Chromosome 2
A
TR50 (h)
2
4
6
B
scale (kbp)
10^3
10^2
115
120
Position (Mbp)

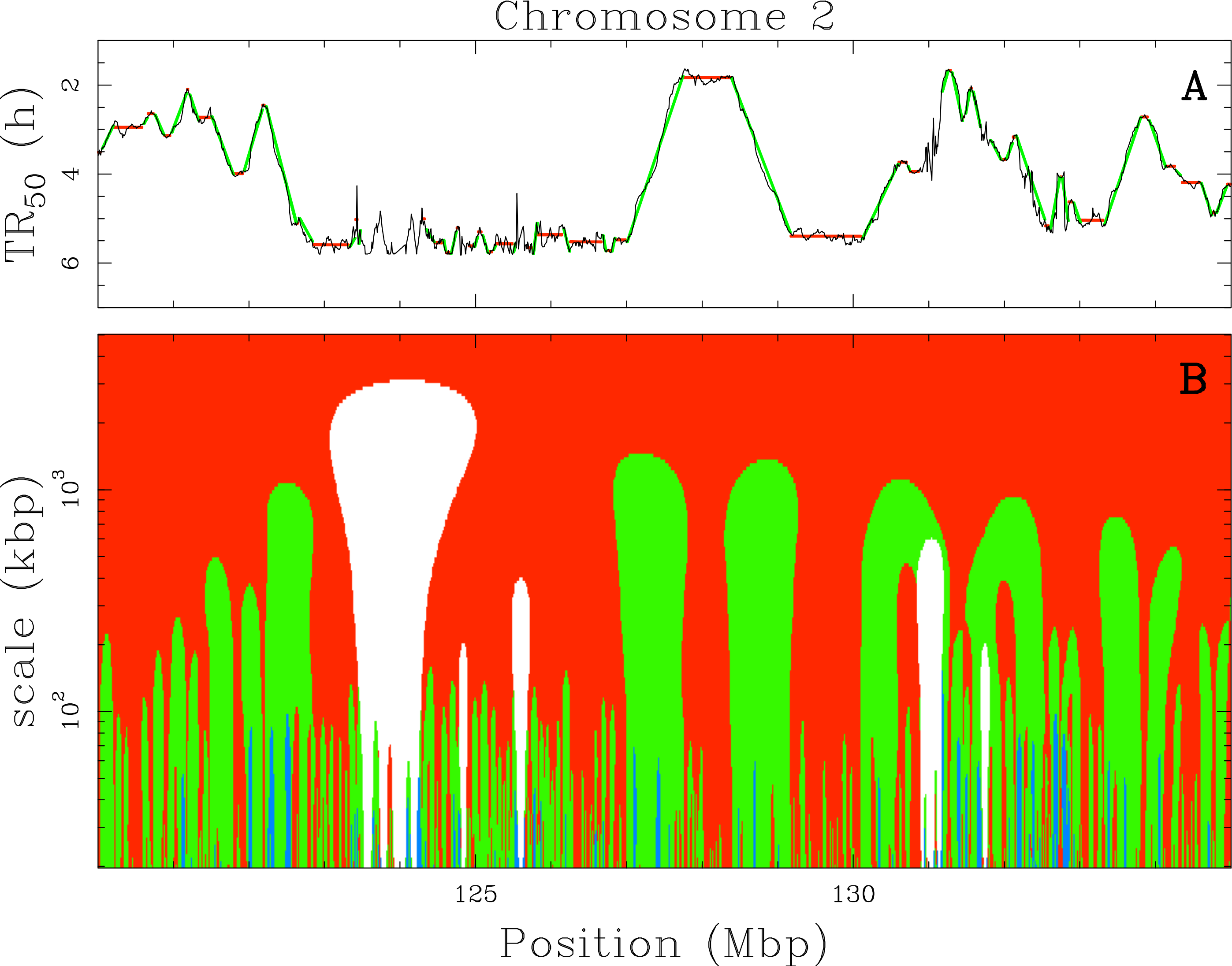

Chromosome 2
TR$_{50}$ (h)
2
4
6
A
B
scale (kbp)
10^3
10^2
125
130
Position (Mbp)

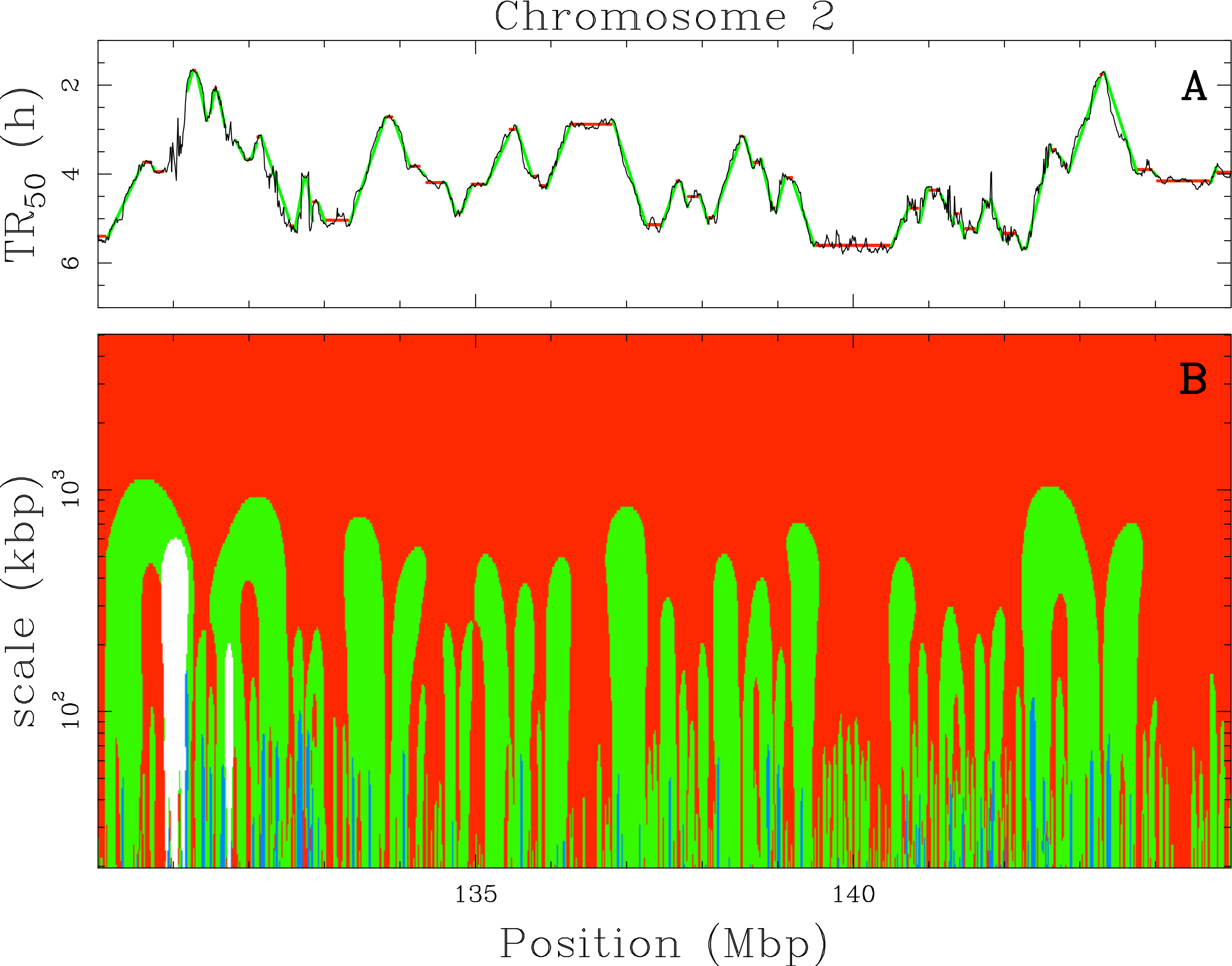
Chromosome 2
A
B
TR50 (h)
2
4
6
scale (kbp)
10^3
10^2
135
140
Position (Mbp)

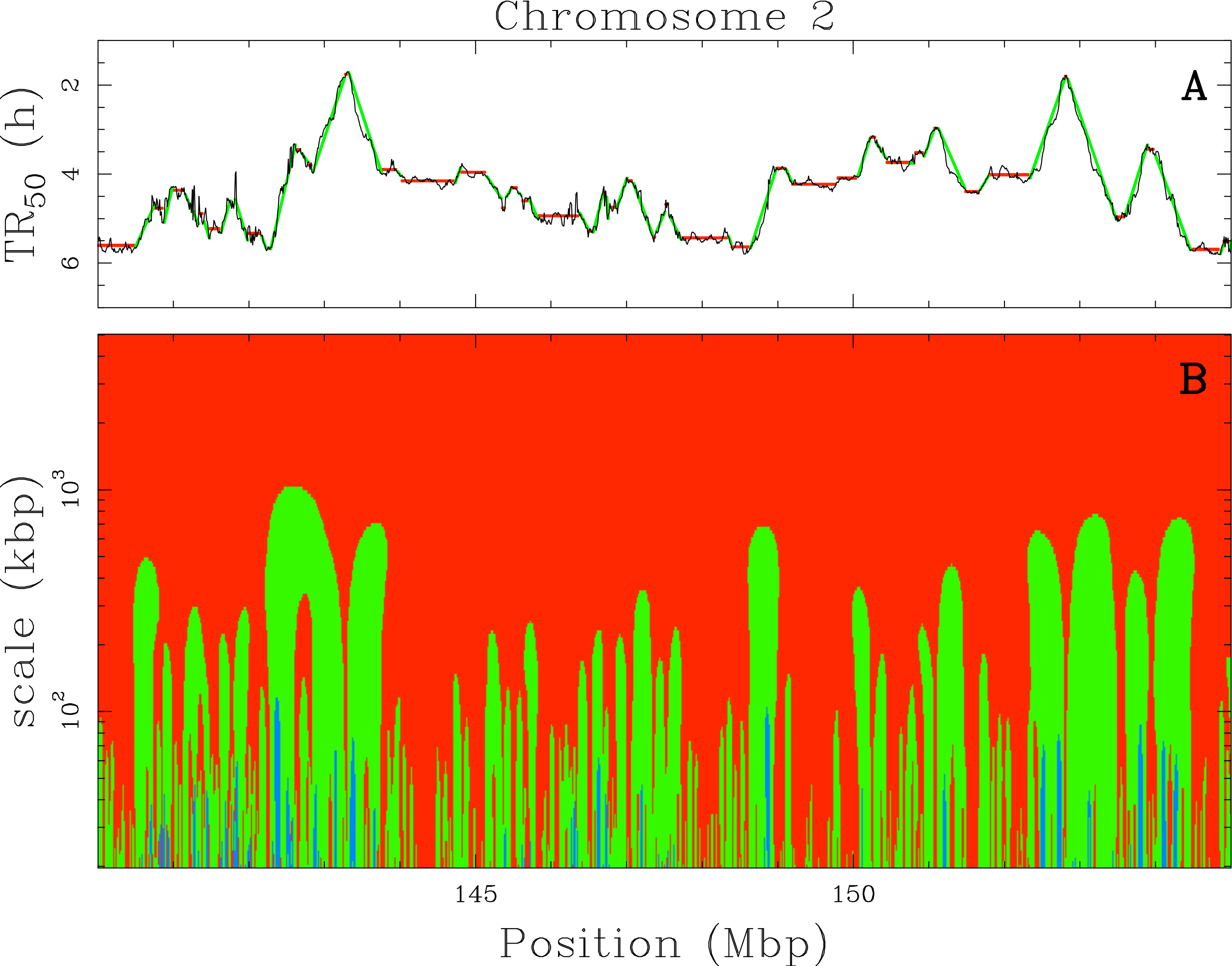

Chromosome 2
A
TR50 (h)
2
4
6
B
scale (kbp)
10^3
10^2
145
150
Position (Mbp)

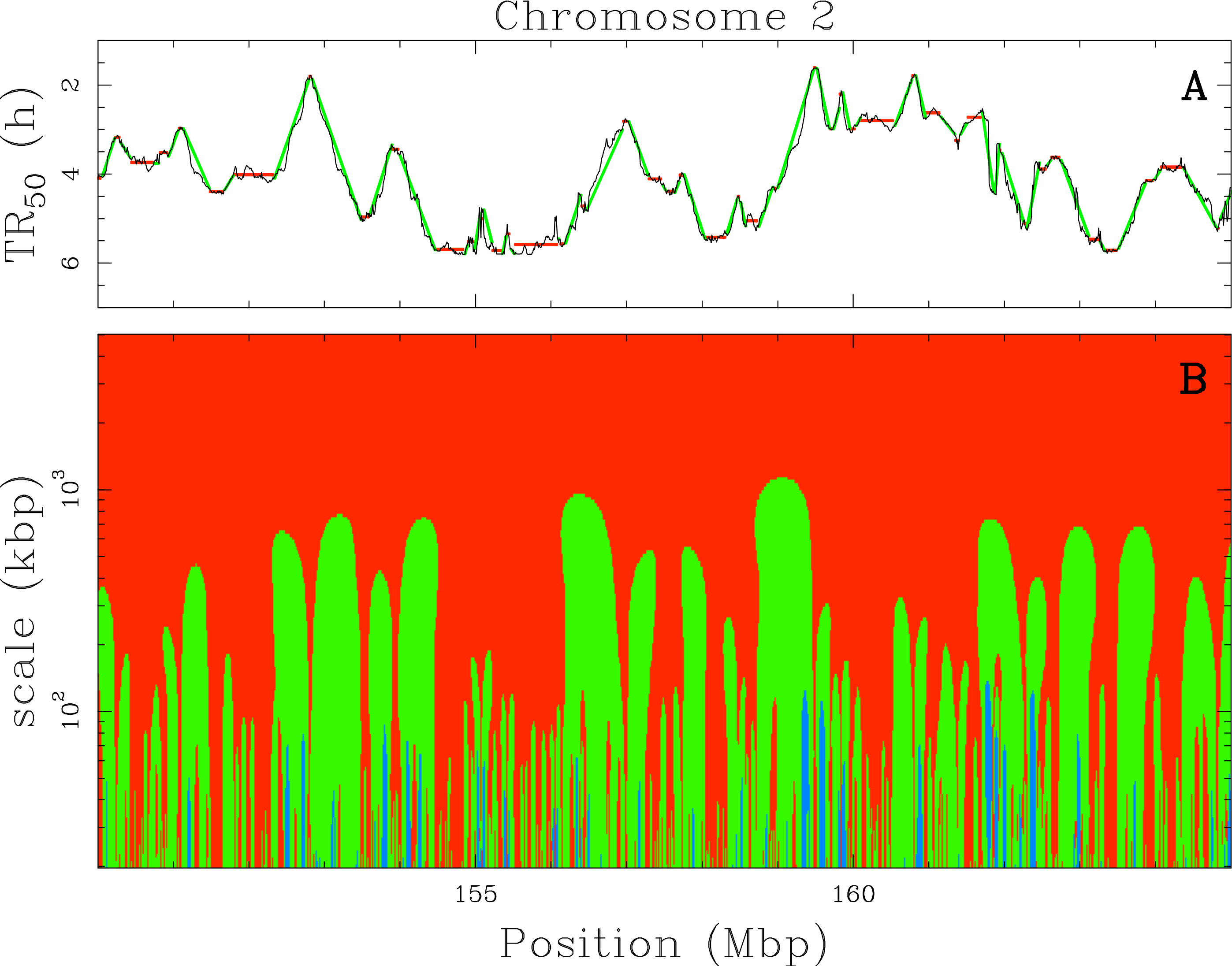

Chromosome 2
A
B
TR50 (h)
2
4
6
scale (kbp)
10^3
10^2
155
160
Position (Mbp)

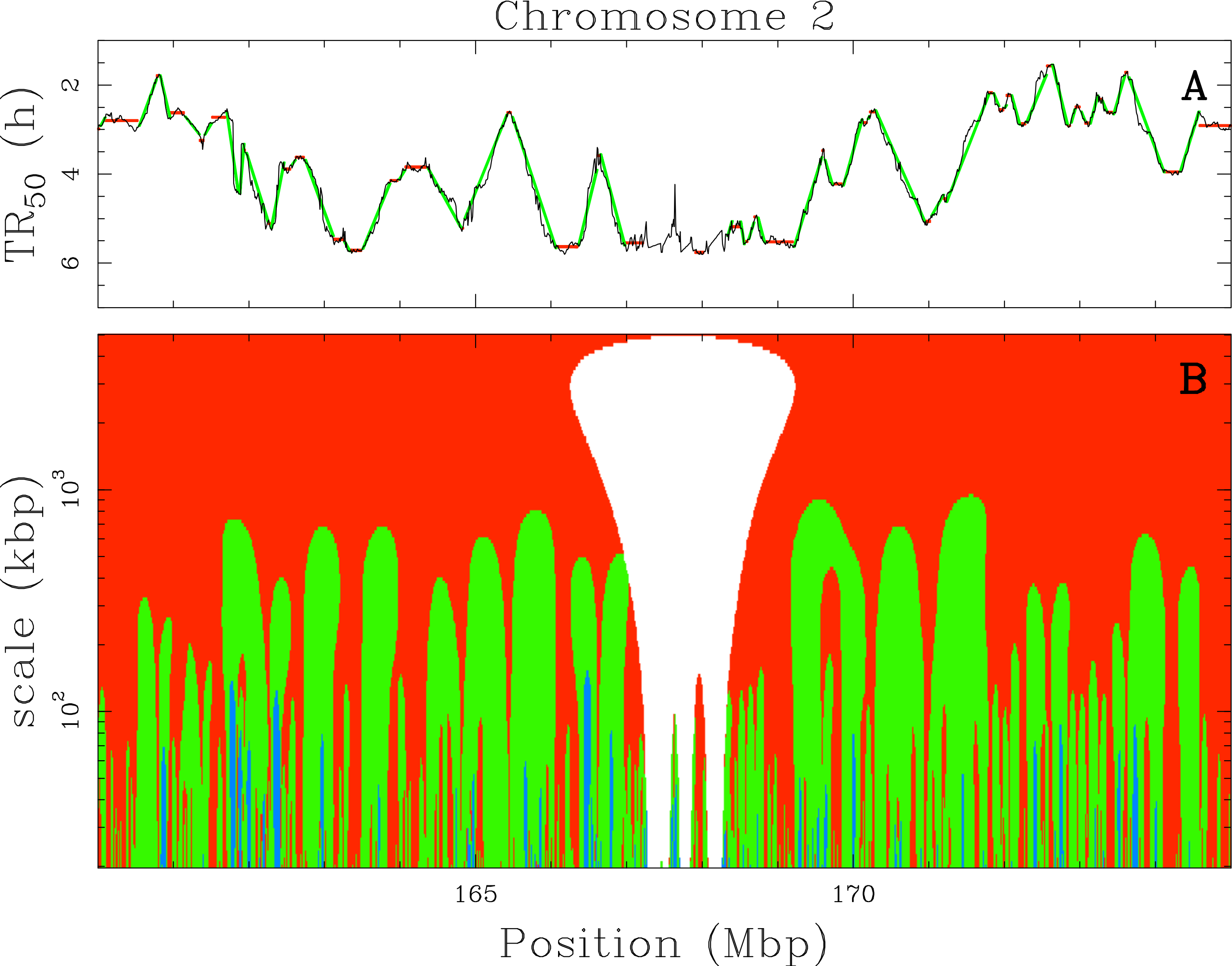

Chromosome 2
A
TR50 (h)
2
4
6
B
scale (kbp)
10^3
10^2
165
170
Position (Mbp)

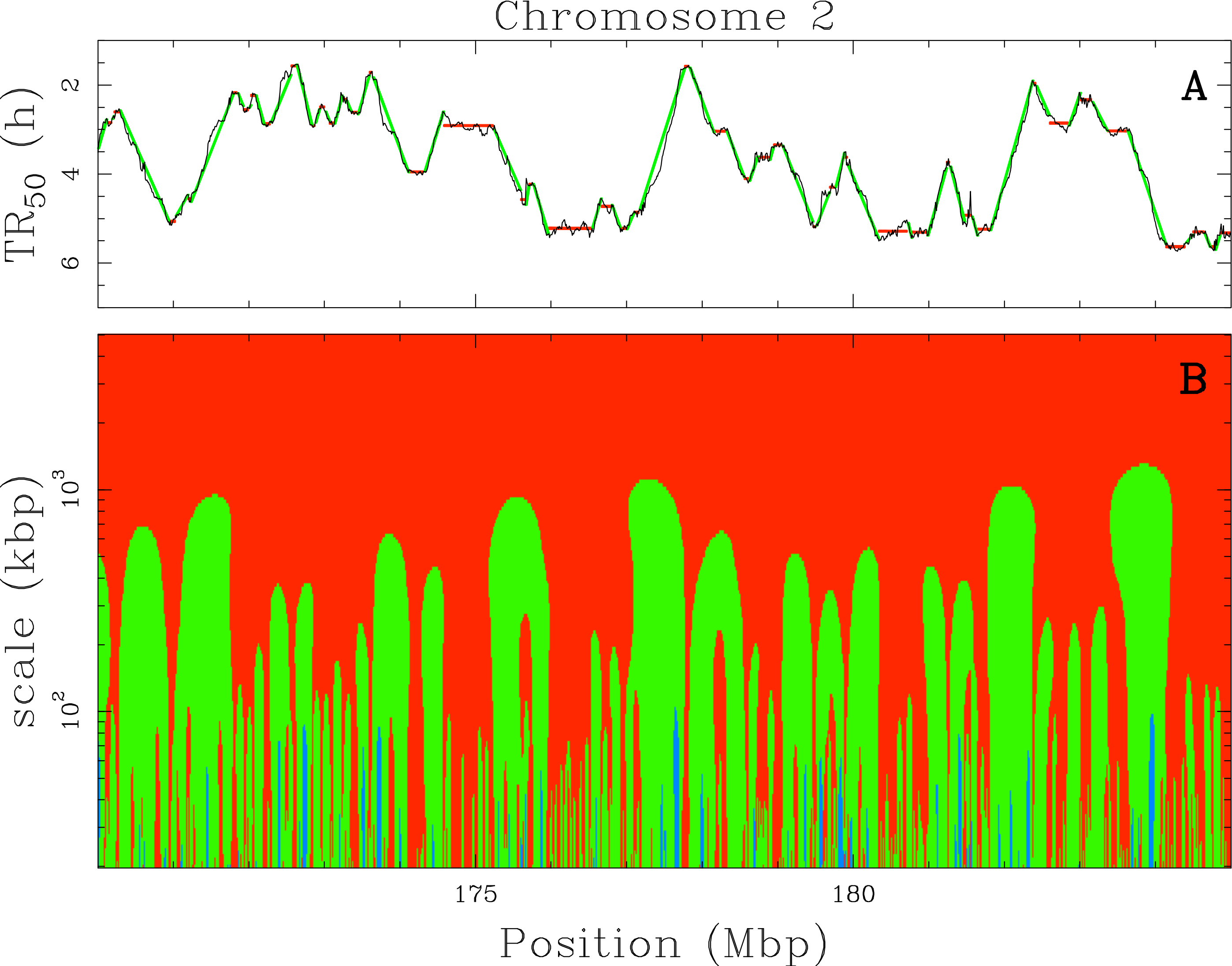

Chromosome 2
A
TR50 (h)
2
4
6
B
scale (kbp)
10^3
10^2
175
180
Position (Mbp)

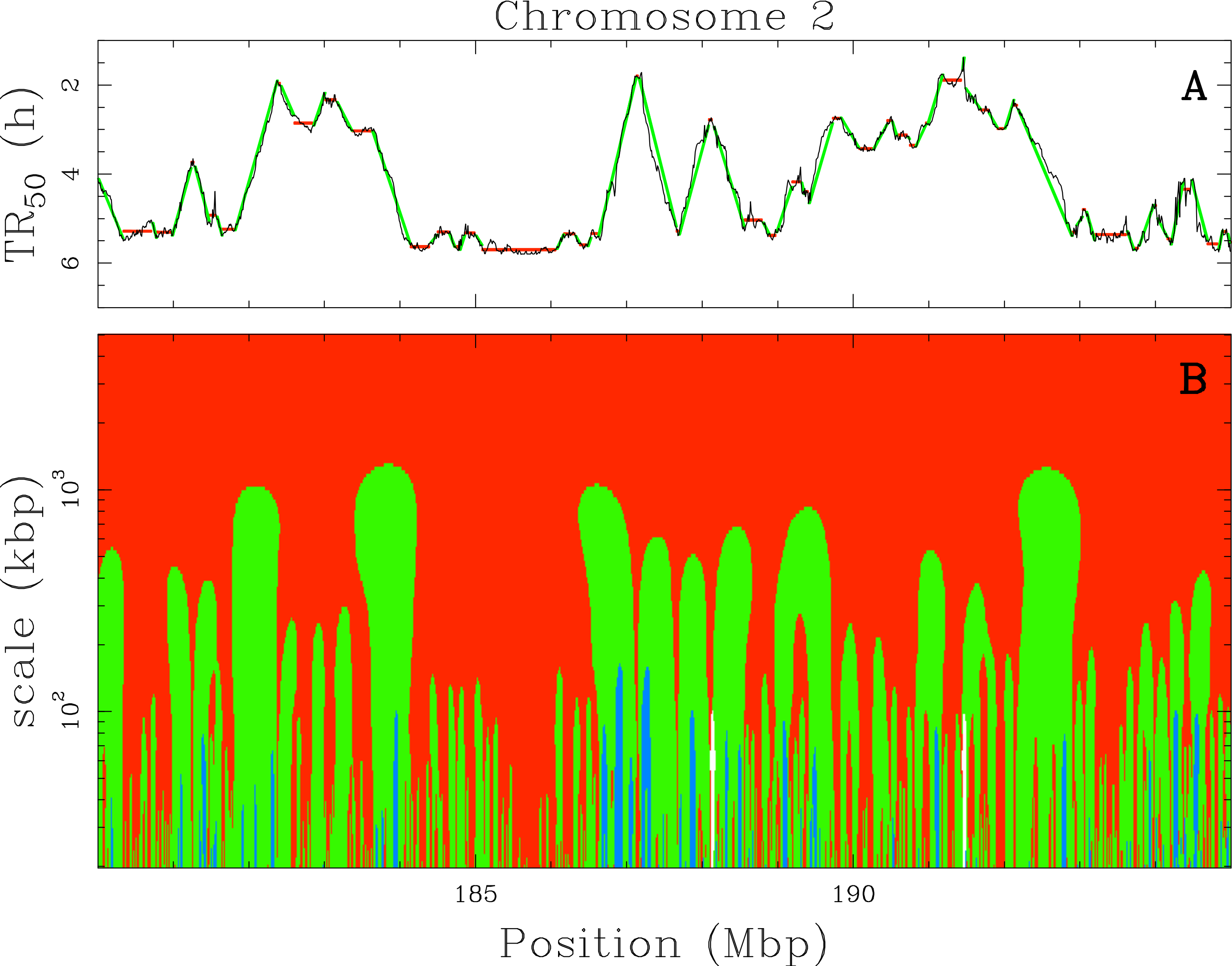

Chromosome 2
TR50 (h)
2
4
6
A
B
scale (kbp)
10^3
10^2
185
190
Position (Mbp)

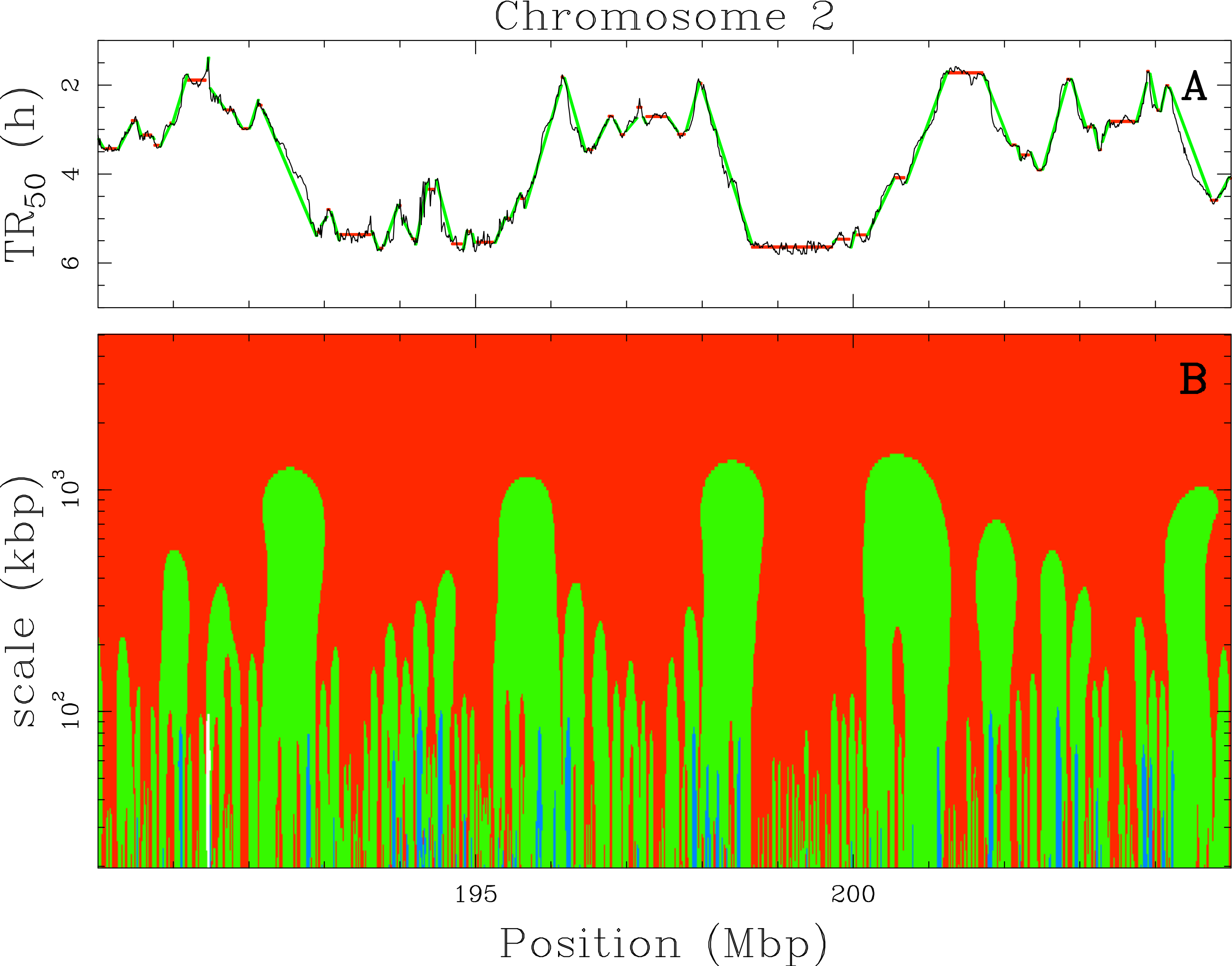

Chromosome 2
A
B
TR50 (h)
2
4
6
scale (kbp)
10^3
10^2
195
200
Position (Mbp)

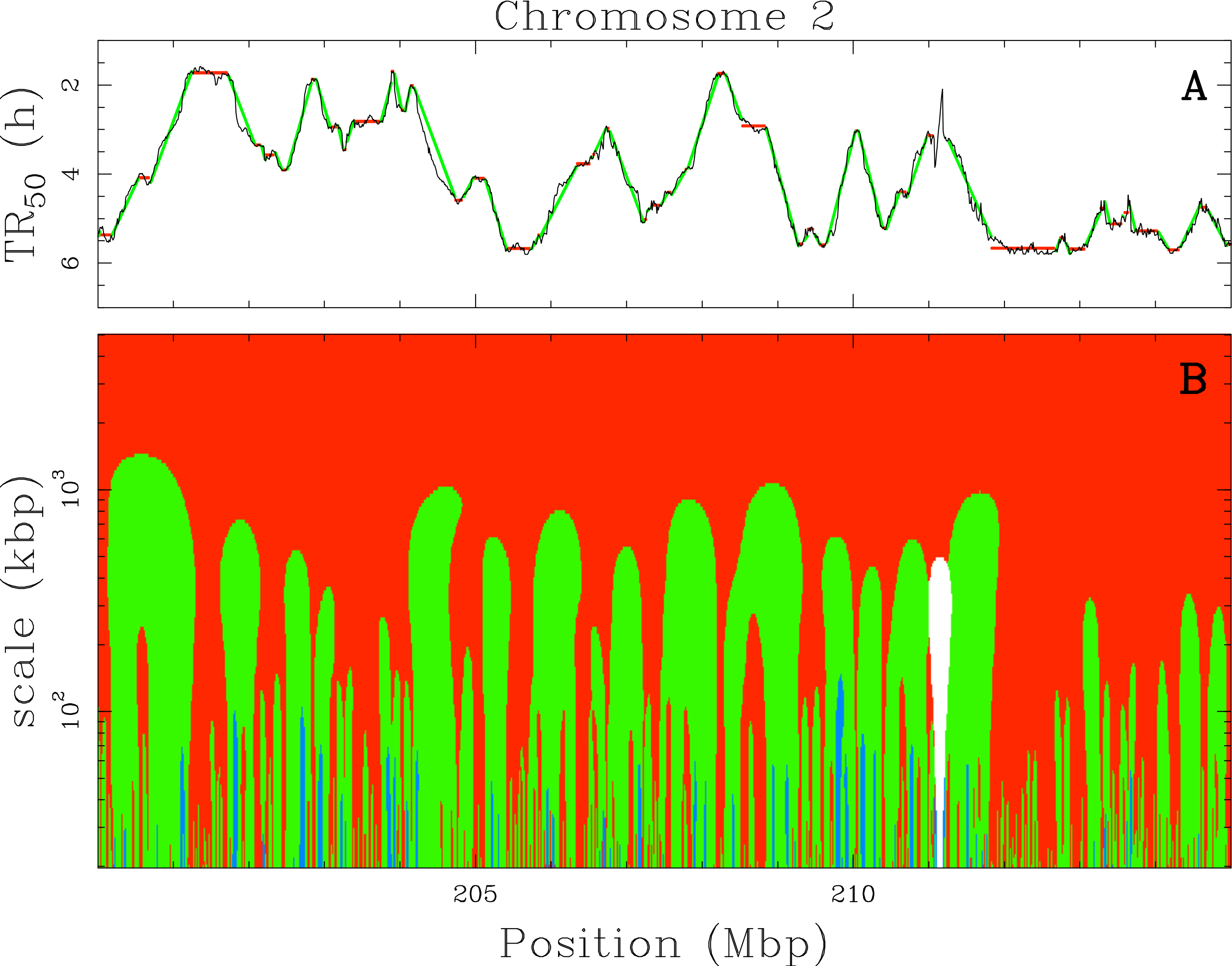

Chromosome 2
A
B
TR$_{50}$ (h)
2
4
6
scale (kbp)
10^3
10^2
205
210
Position (Mbp)

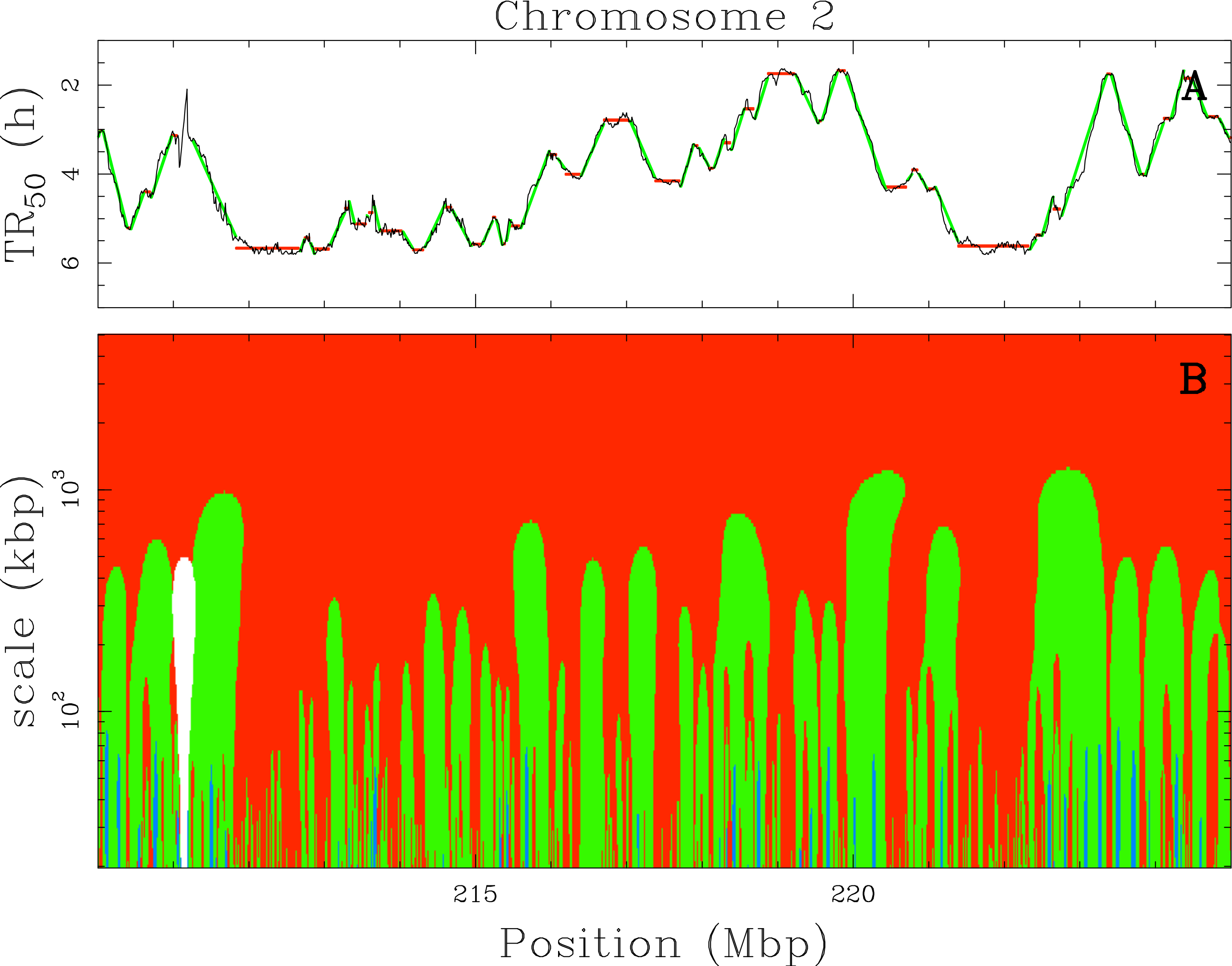

Chromosome 2
A
B
TR50 (h)
2
4
6
scale (kbp)
10^3
10^2
215
220
Position (Mbp)

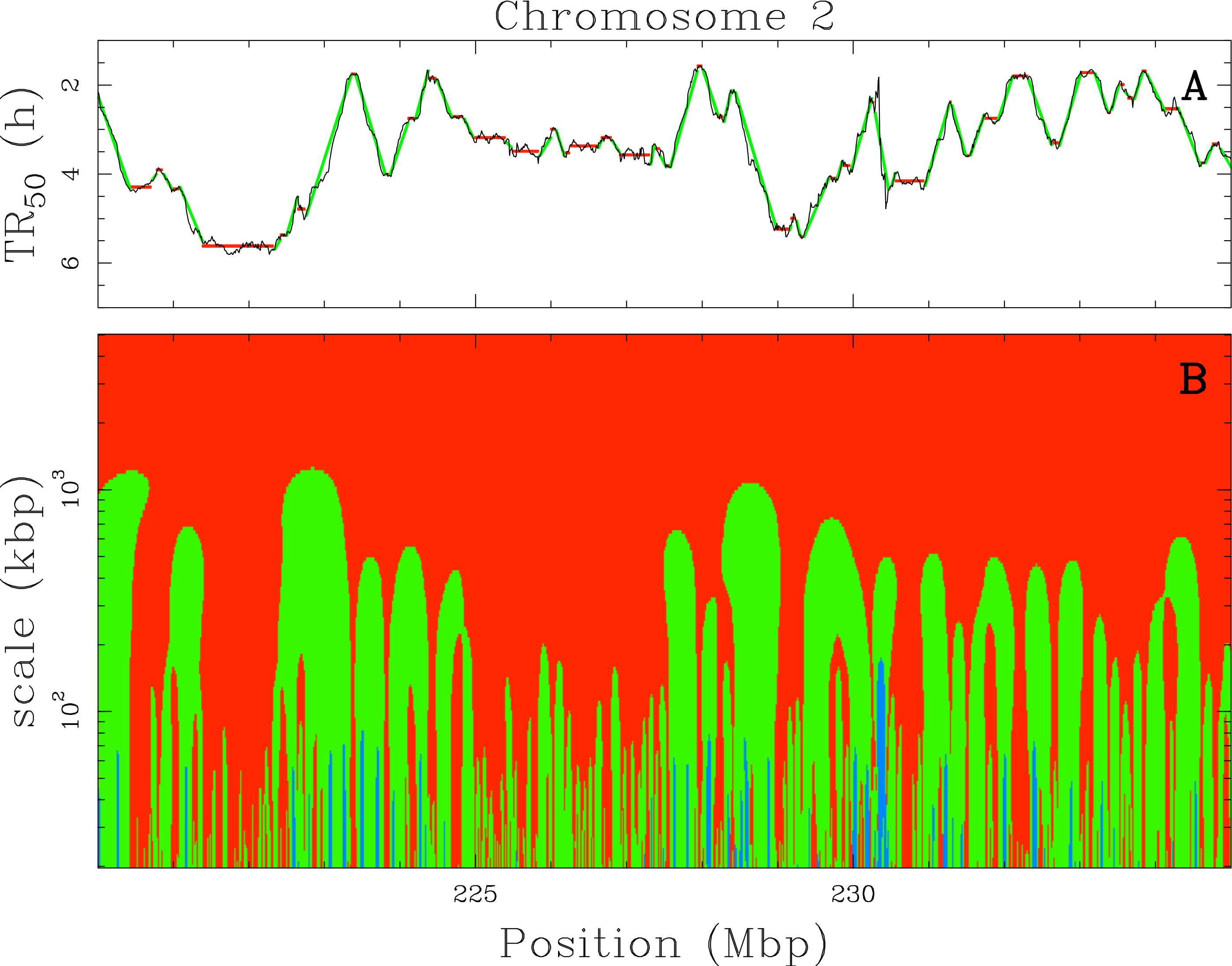

Chromosome 2
A
B
TR50 (h)
2
4
6
scale (kbp)
10^3
10^2
225
230
Position (Mbp)

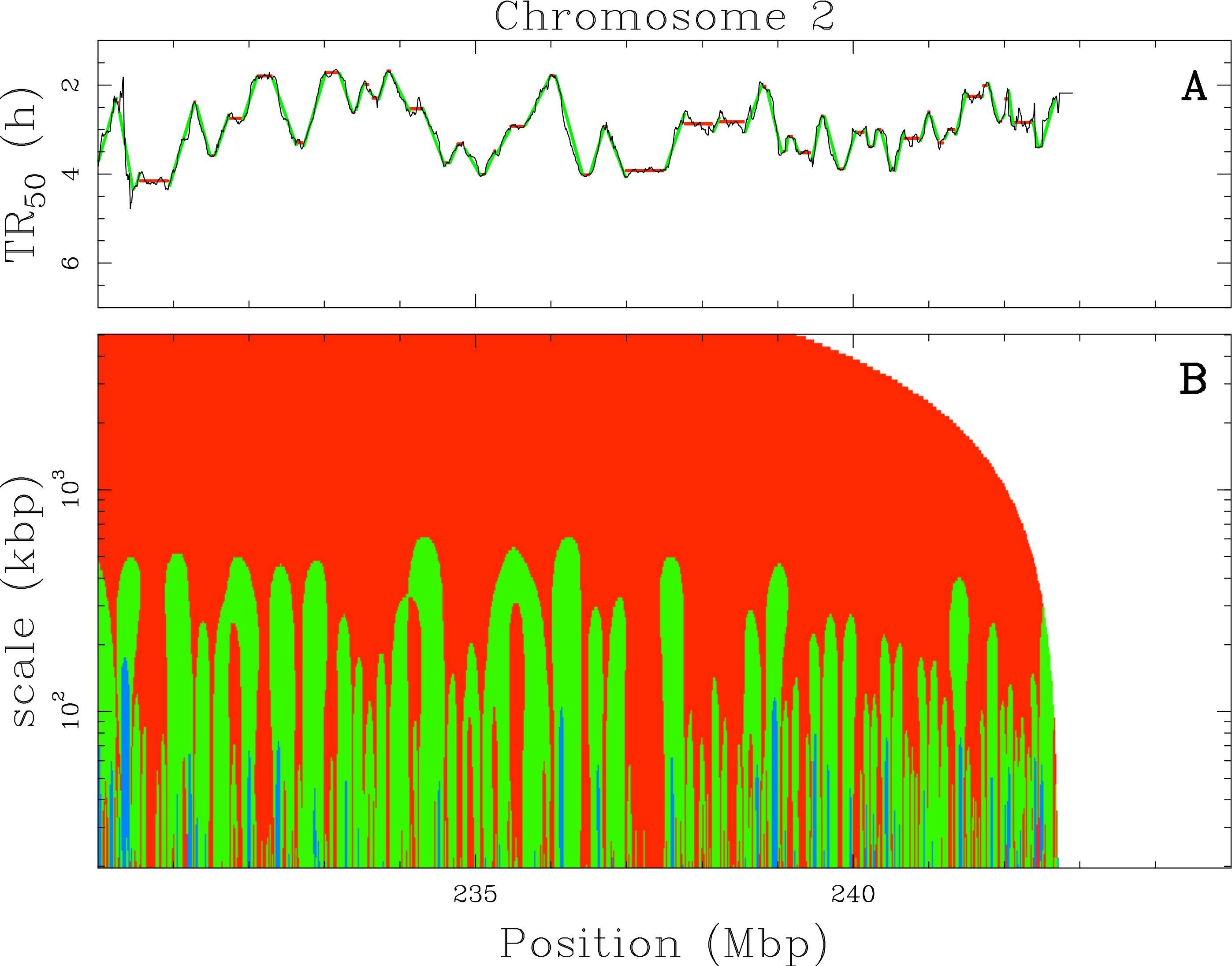

Chromosome 2
A
B
TR50 (h)
2
4
6
scale (kbp)
10^3
10^2
235
240
Position (Mbp)

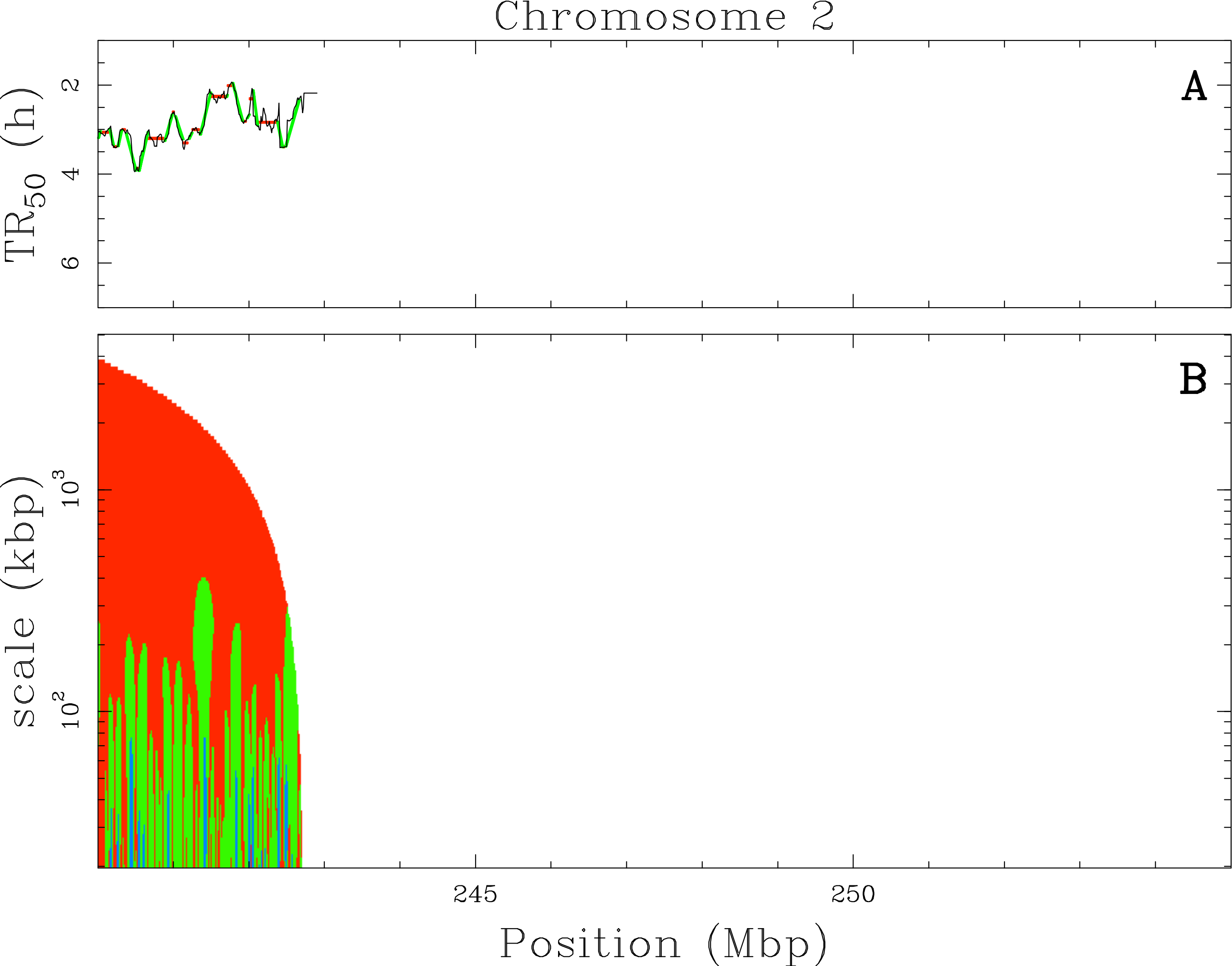

Chromosome 2
TR50 (h)
2
4
6
A
B
scale (kbp)
10^3
10^2
245
250
Position (Mbp)

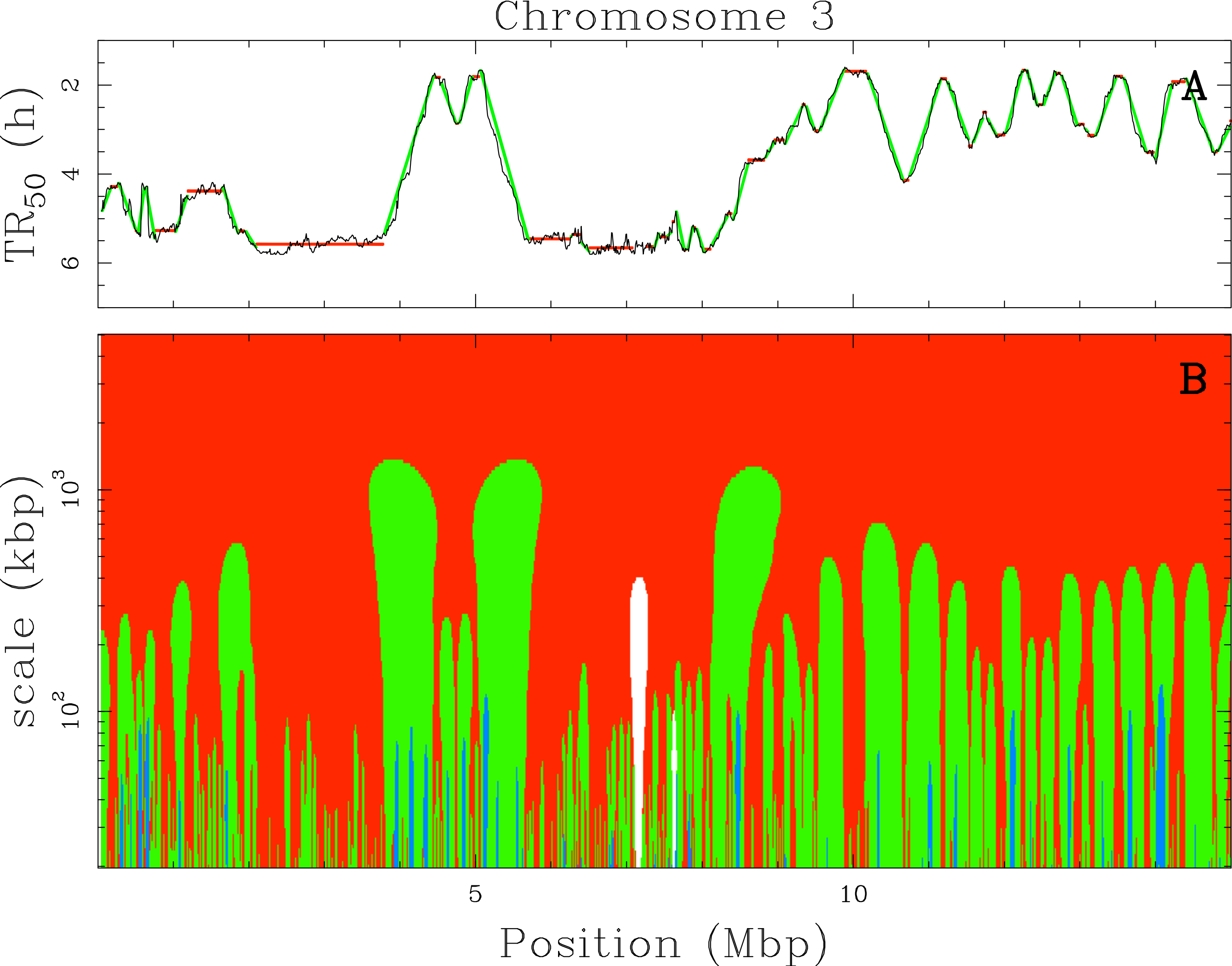
Chromosome 3
A
B
TR50 (h)
2
4
6
scale (kbp)
10^3
10^2
5
10
Position (Mbp)

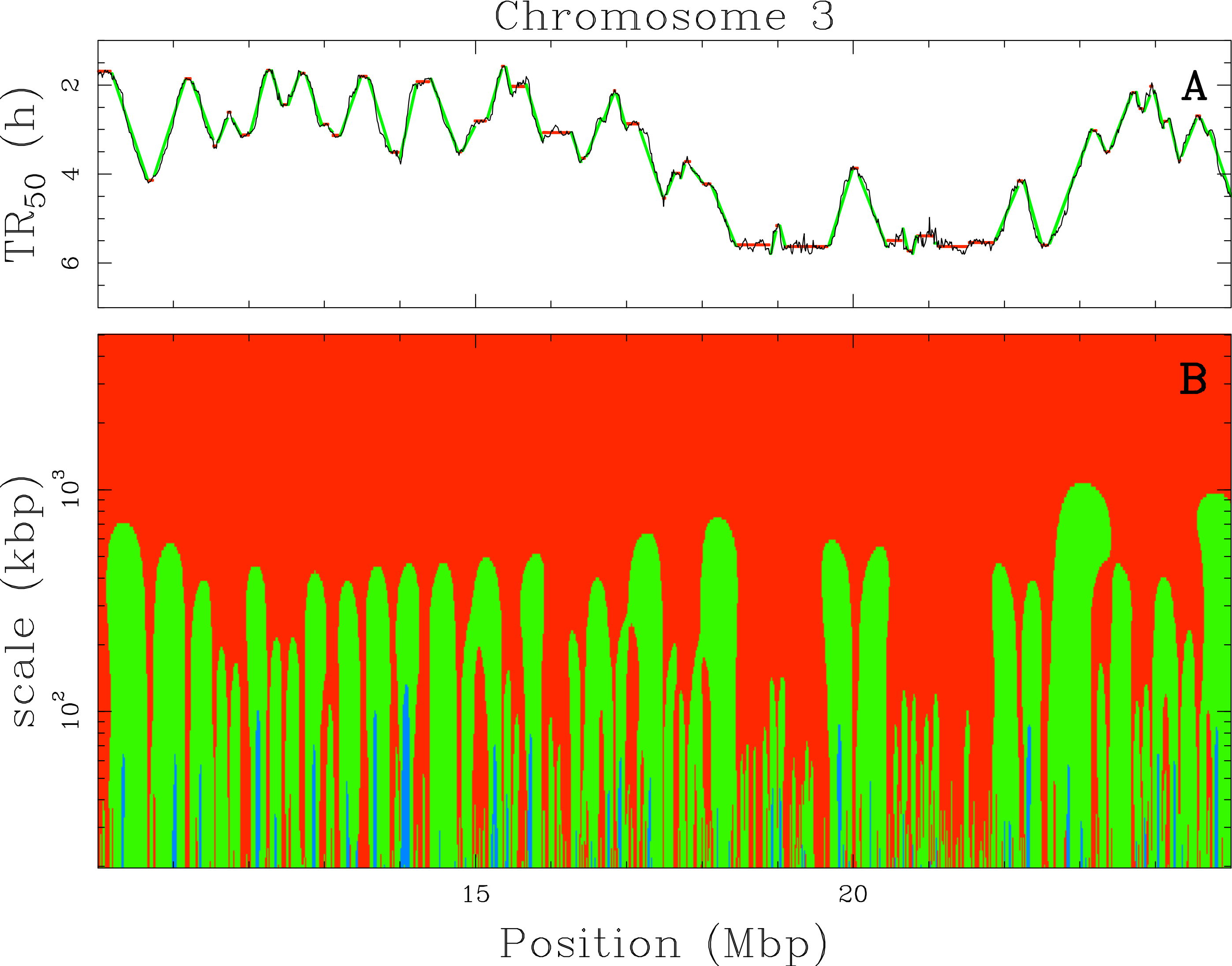

Chromosome 3
A
B
TR50 (h)
2
4
6
scale (kbp)
10^3
10^2
15
20
Position (Mbp)

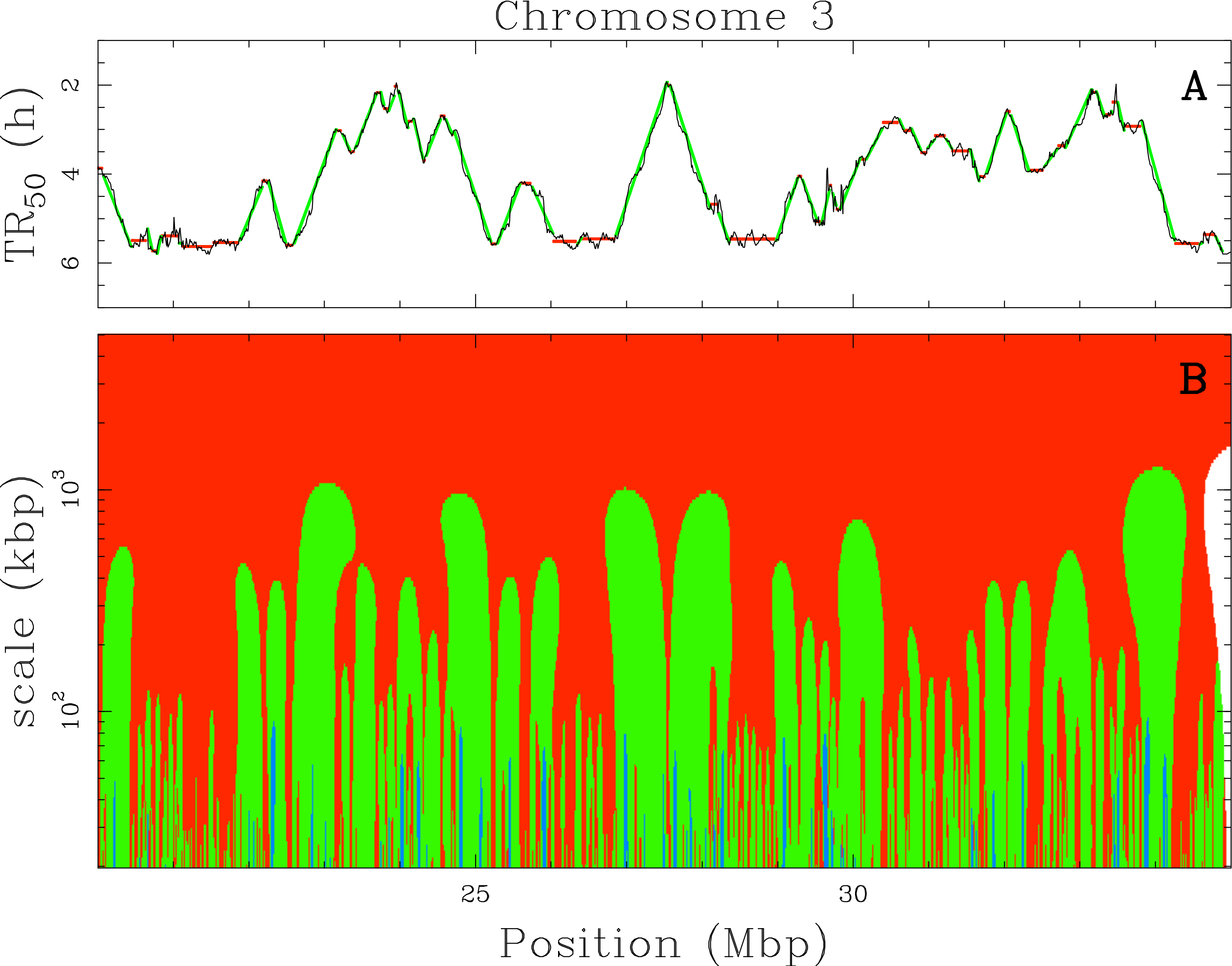

Chromosome 3
A
B
TR50 (h)
2
4
6
scale (kbp)
10^3
10^2
25
30
Position (Mbp)

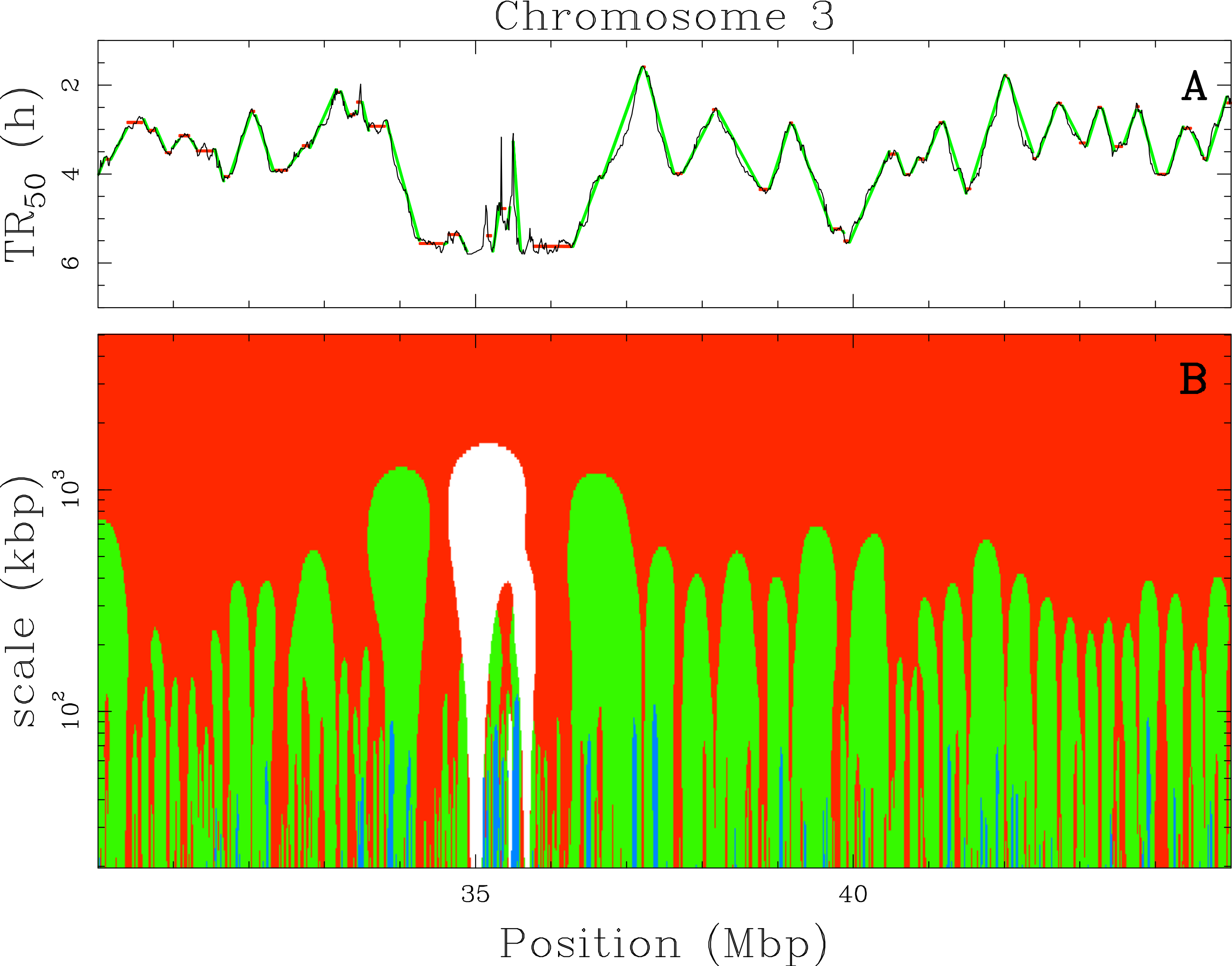
Chromosome 3
A
TR_{50} (h)
2
4
6
B
scale (kbp)
10^3
10^2
35
40
Position (Mbp)

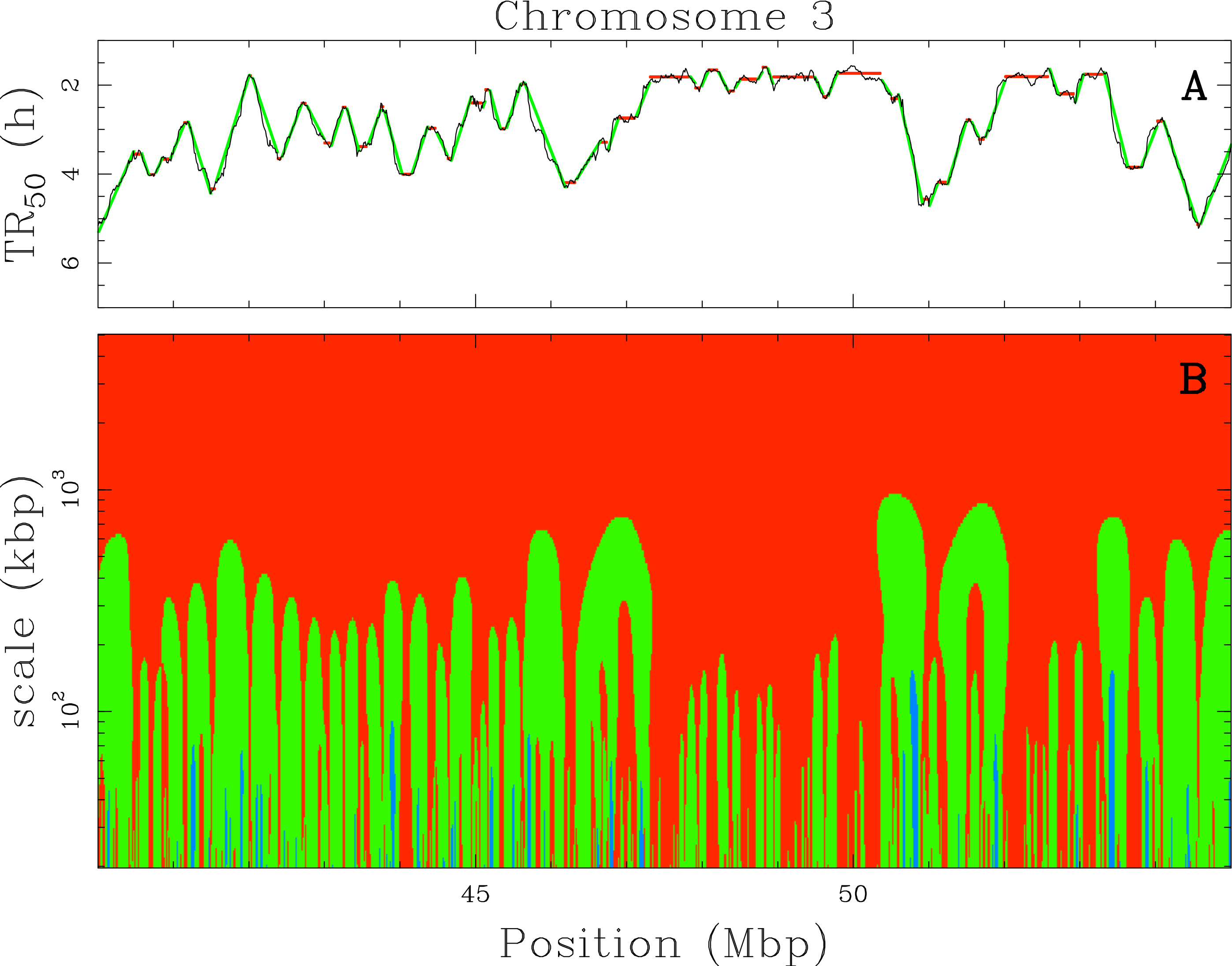
Chromosome 3
TR_{50} (h)
2
4
6
A
B
scale (kbp)
10^3
10^2
45
50
Position (Mbp)

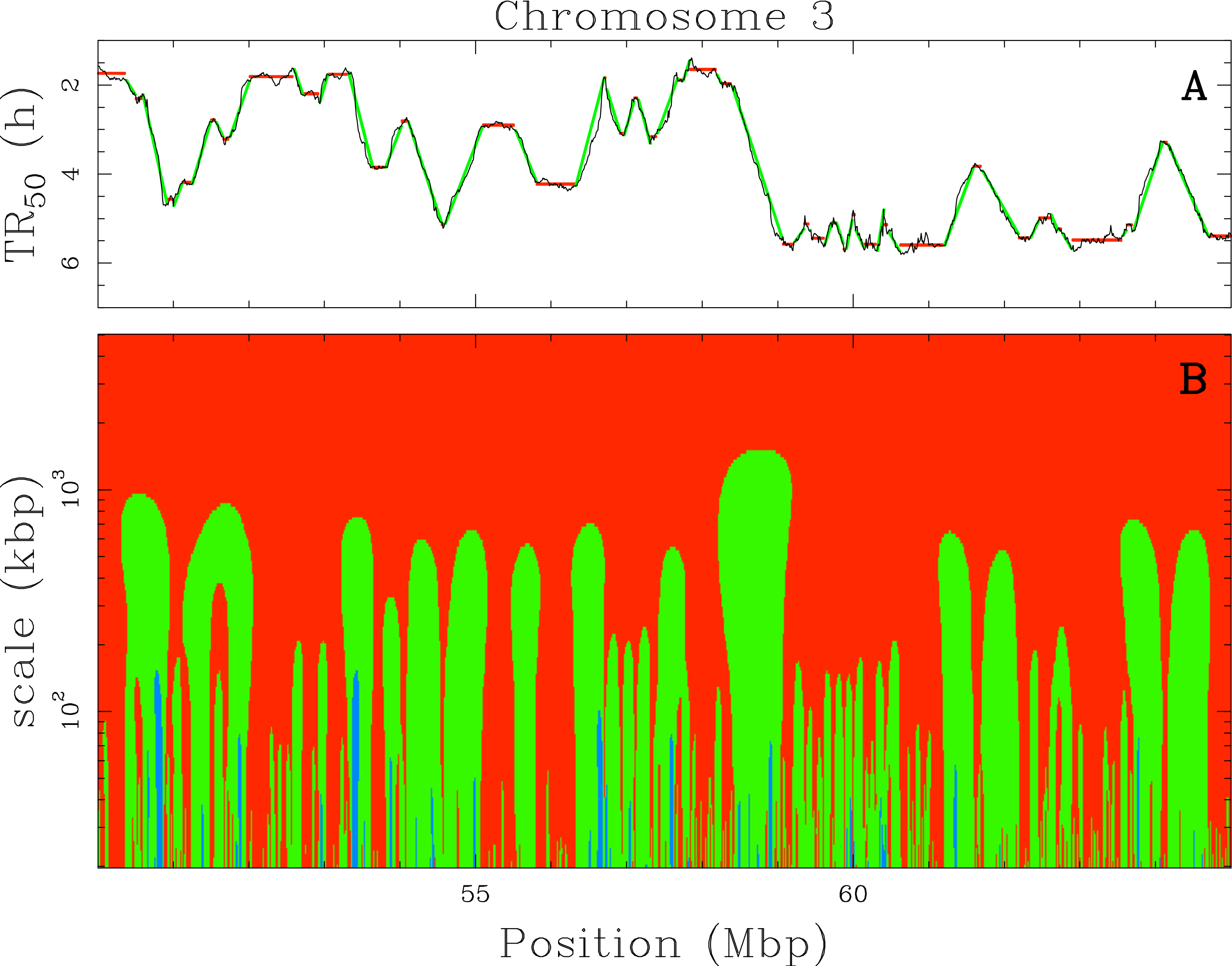

Chromosome 3
A
B
TR50 (h)
2
4
6
scale (kbp)
10^3
10^2
55
60
Position (Mbp)

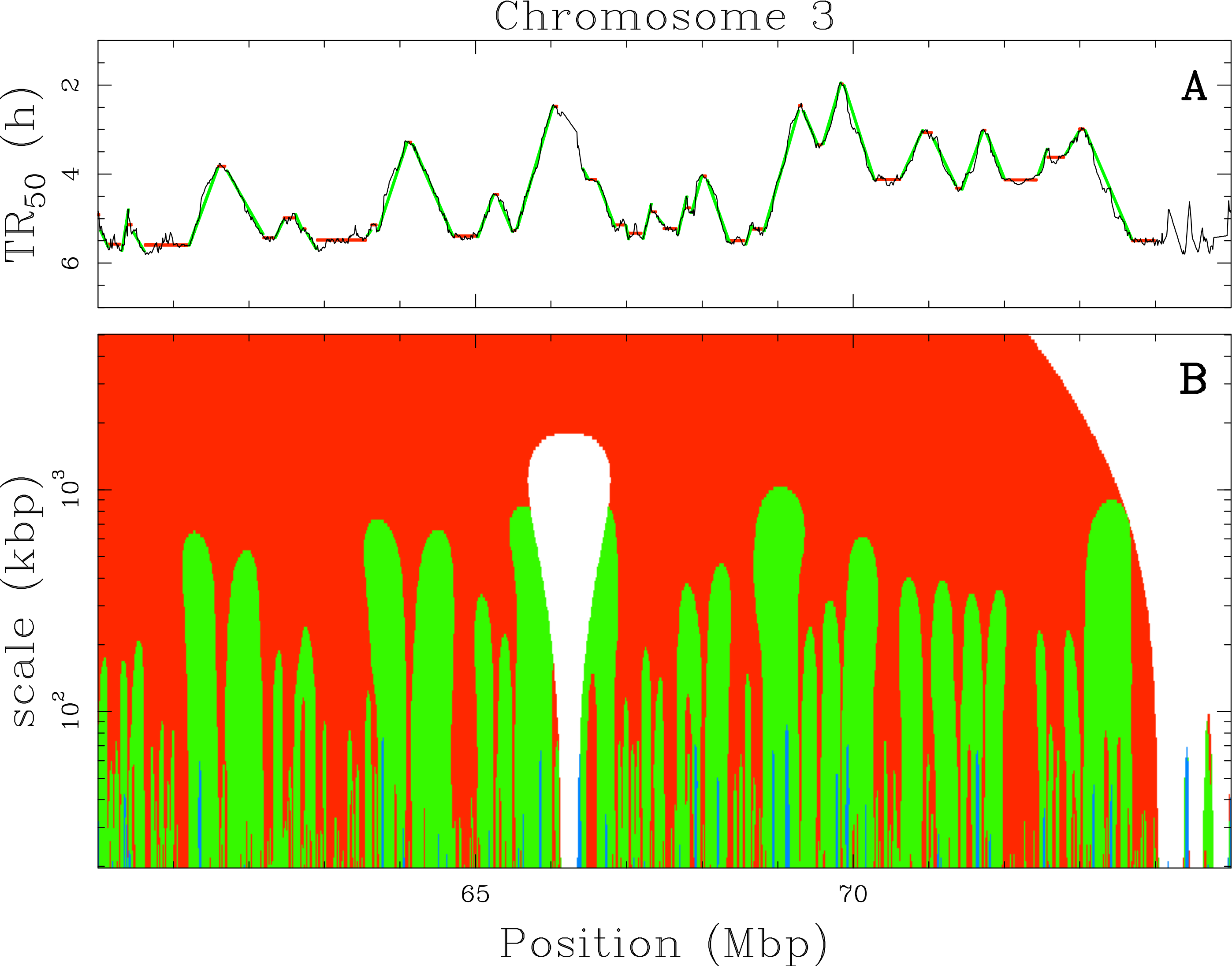

Chromosome 3
A
TR50 (h)
2
4
6
B
scale (kbp)
10^3
10^2
65
70
Position (Mbp)

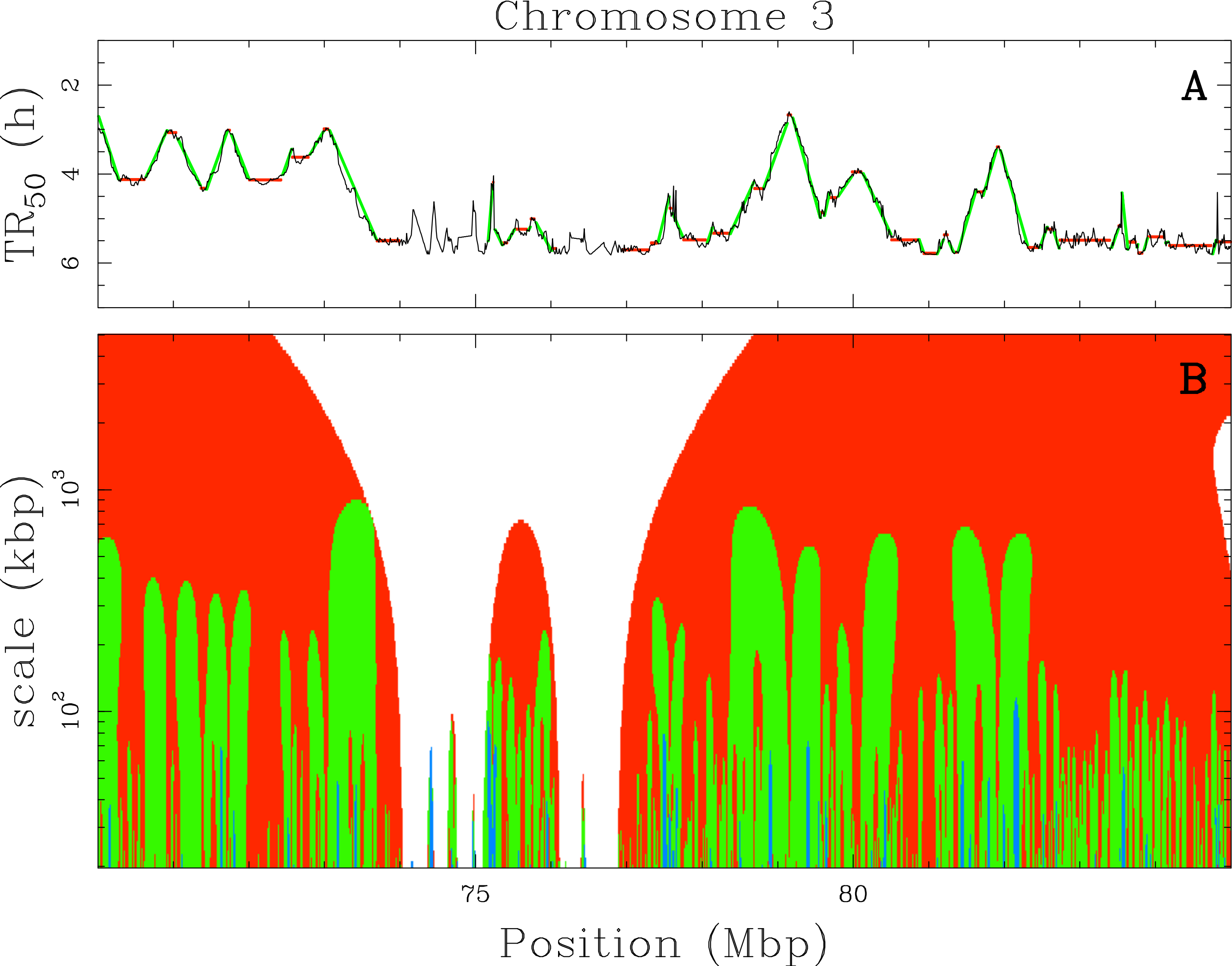
Chromosome 3
A
B
TR50 (h)
2
4
6
scale (kbp)
10^3
10^2
75
80
Position (Mbp)

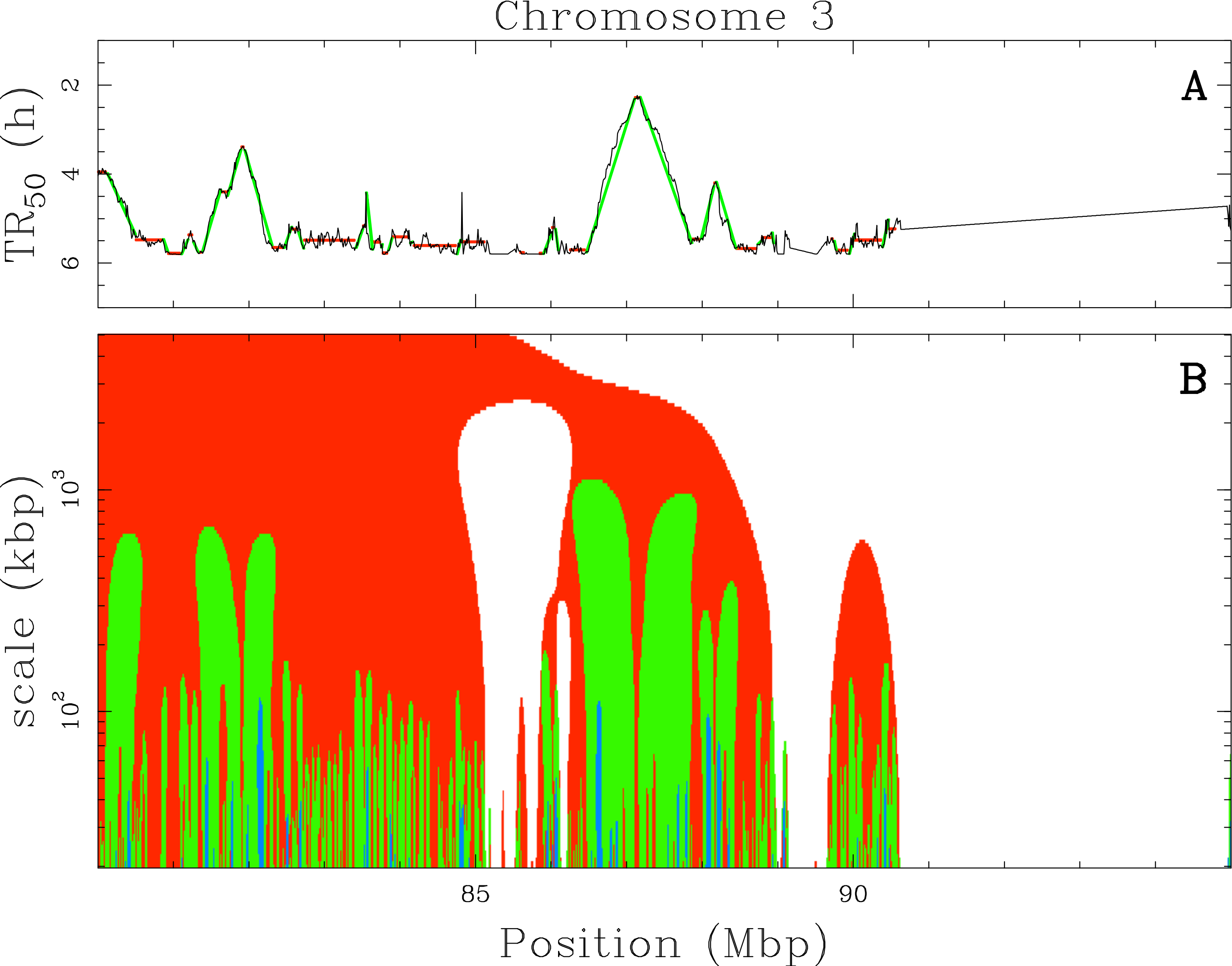

Chromosome 3
A
B
TR50 (h)
2
4
6
scale (kbp)
10^3
10^2
85
90
Position (Mbp)

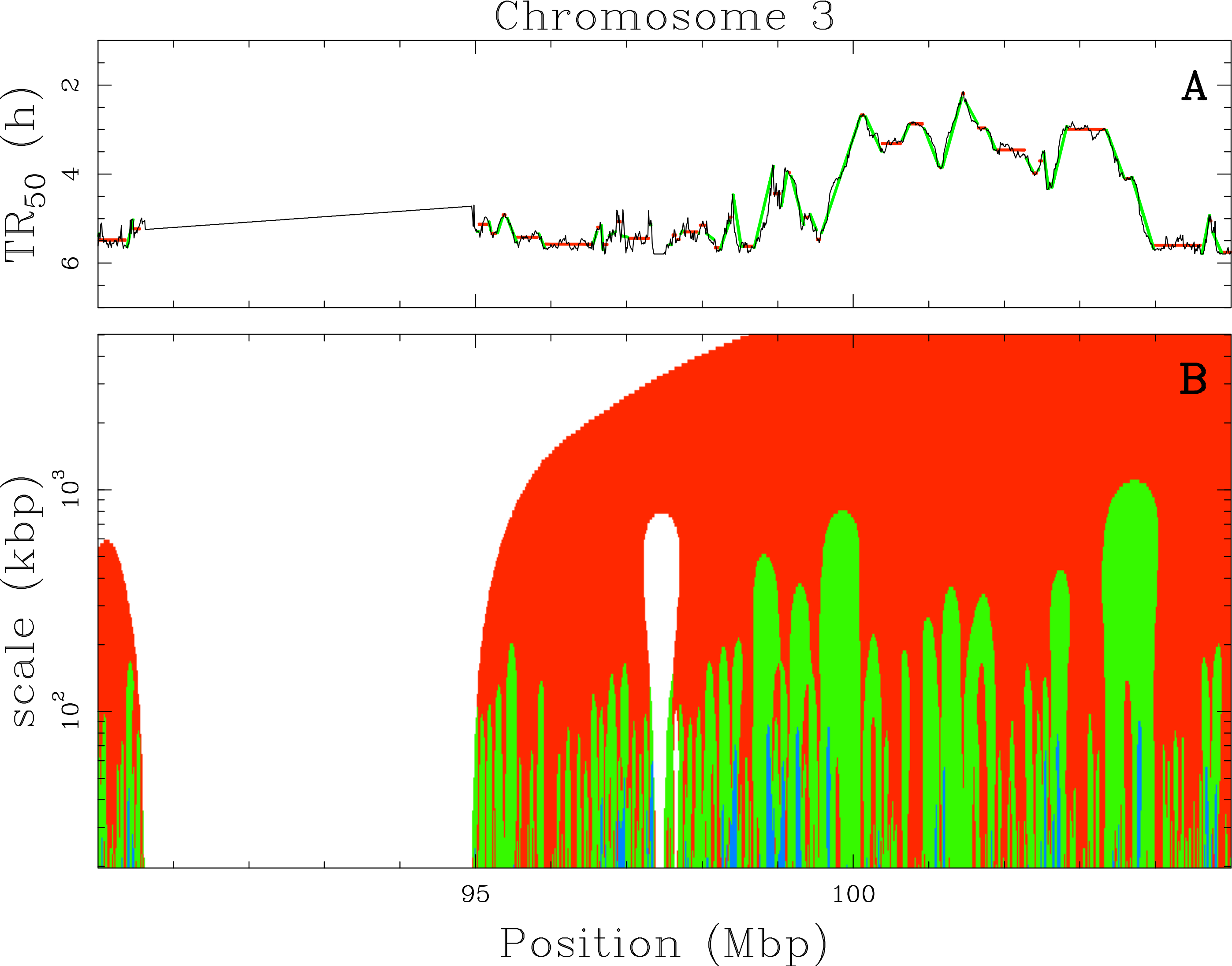
Chromosome 3
A
B
TR_{50} (h)
2
4
6
scale (kbp)
10^3
10^2
95
100
Position (Mbp)

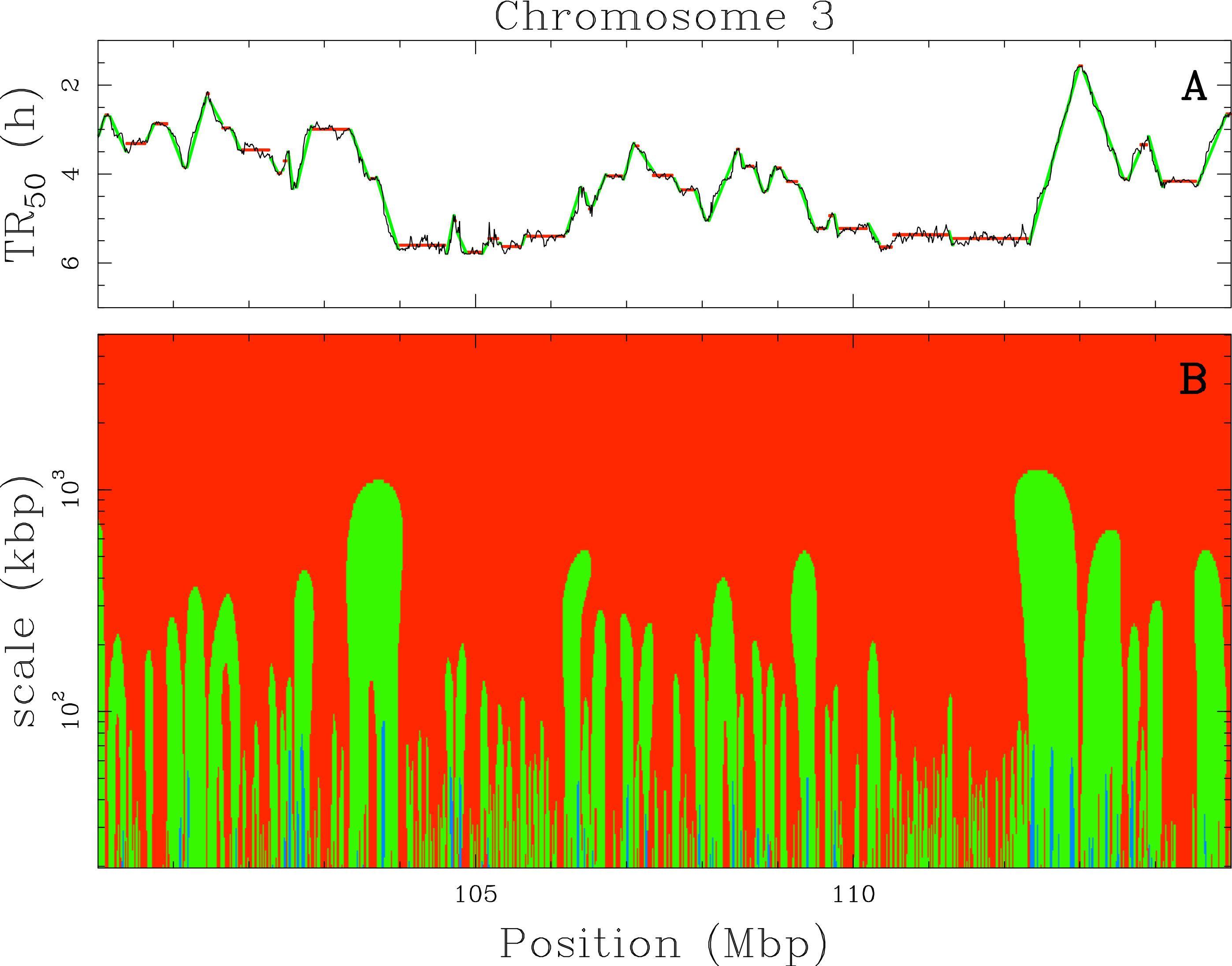
Chromosome 3
A
B
TR50 (h)
2
4
6
scale (kbp)
10^3
10^2
105
110
Position (Mbp)

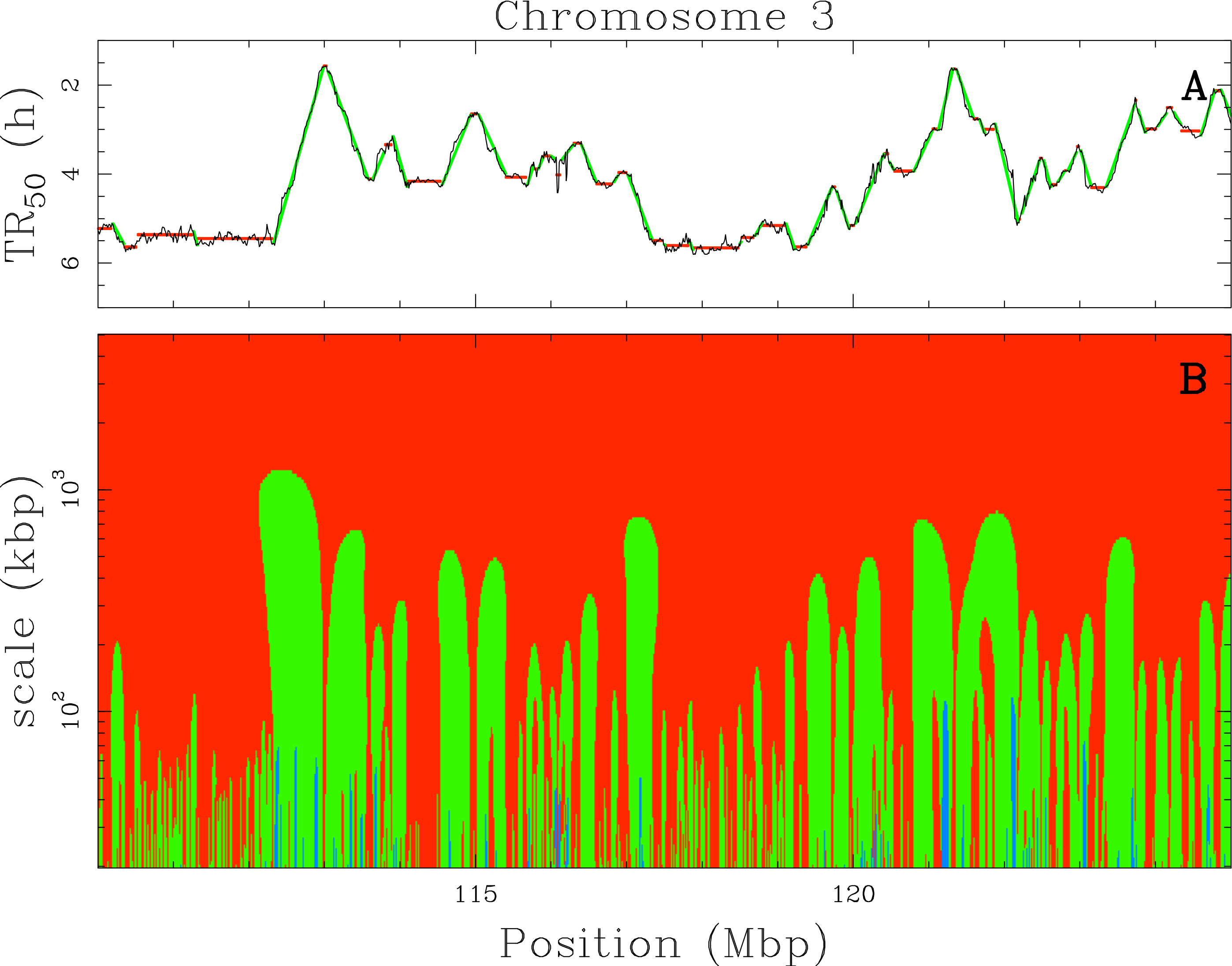

Chromosome 3
A
B
TR50 (h)
2
4
6
scale (kbp)
10^3
10^2
115
120
Position (Mbp)

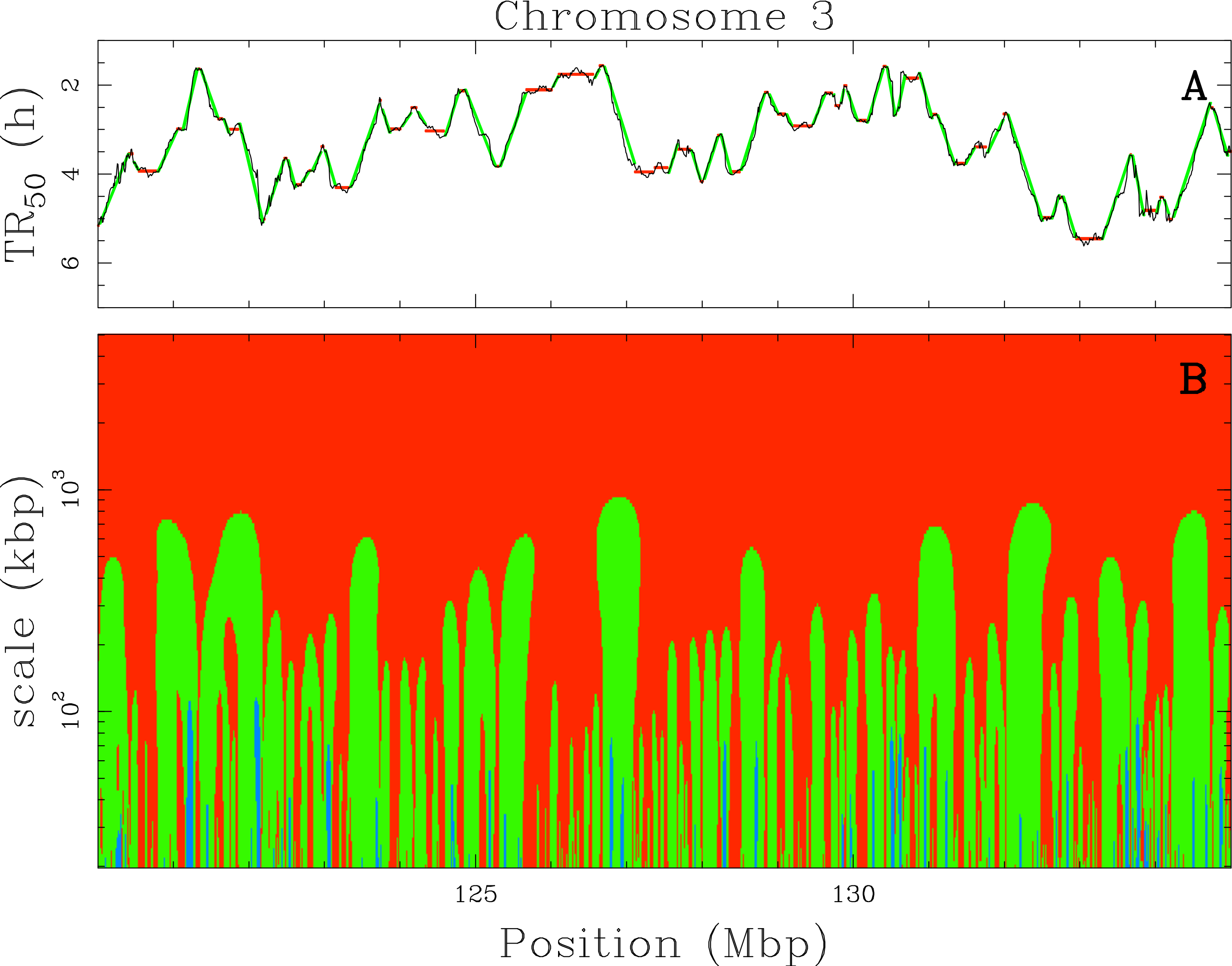

Chromosome 3
A
TR50 (h)
2
4
6
B
scale (kbp)
10^3
10^2
125
130
Position (Mbp)

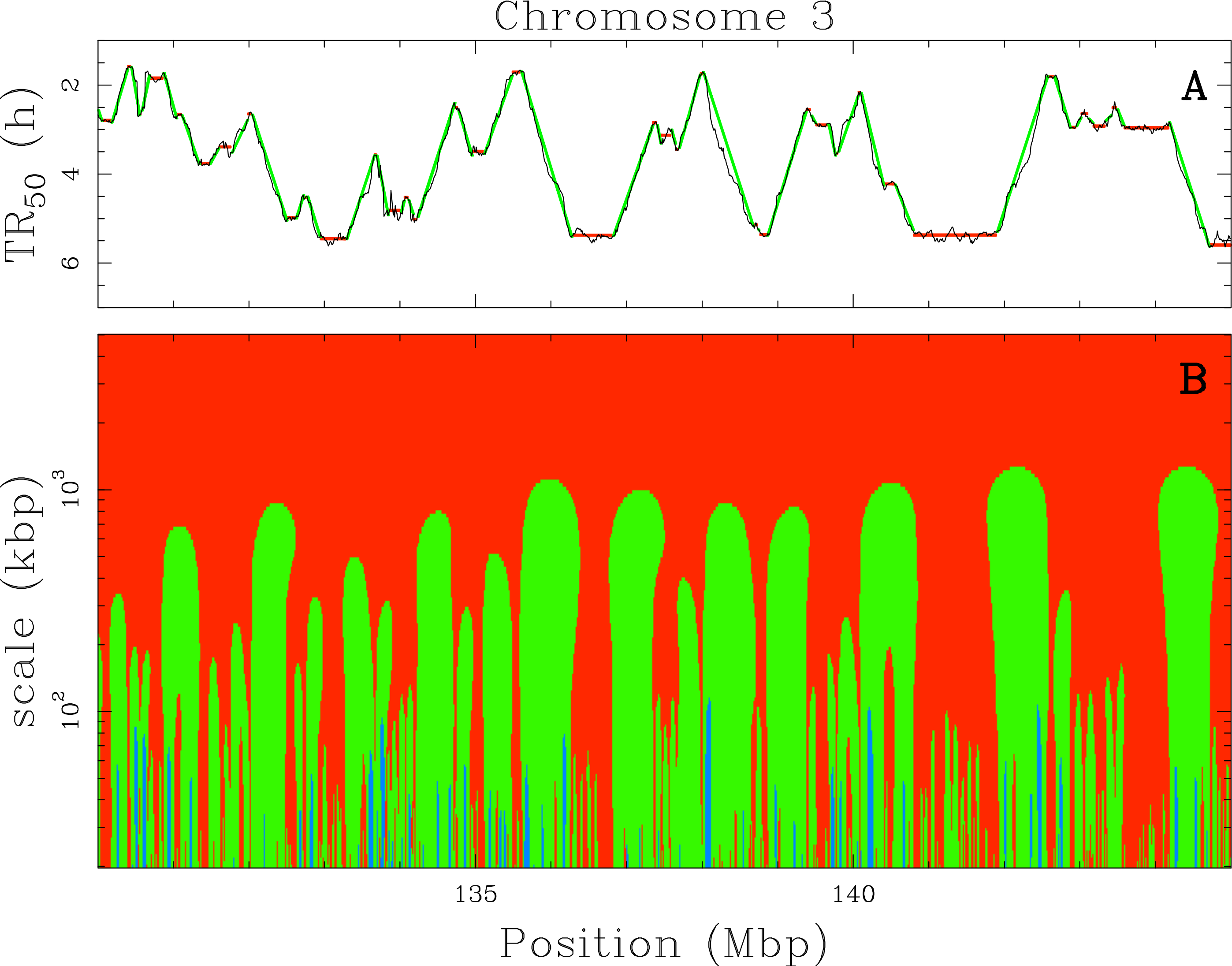

Chromosome 3
A
TR50 (h)
2
4
6
B
scale (kbp)
10^3
10^2
135
140
Position (Mbp)

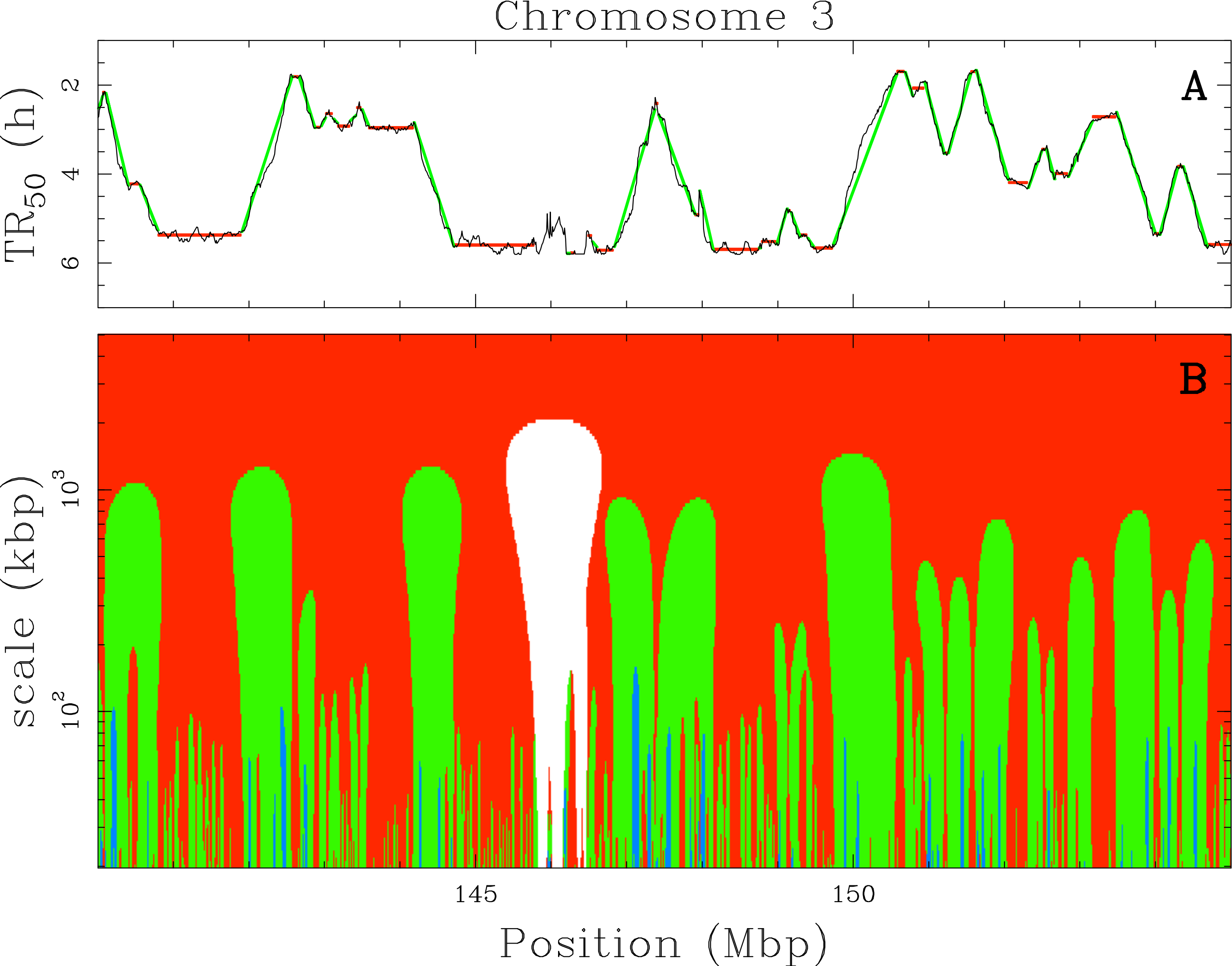

Chromosome 3
A
B
TR50 (h)
2
4
6
scale (kbp)
10^3
10^2
145
150
Position (Mbp)

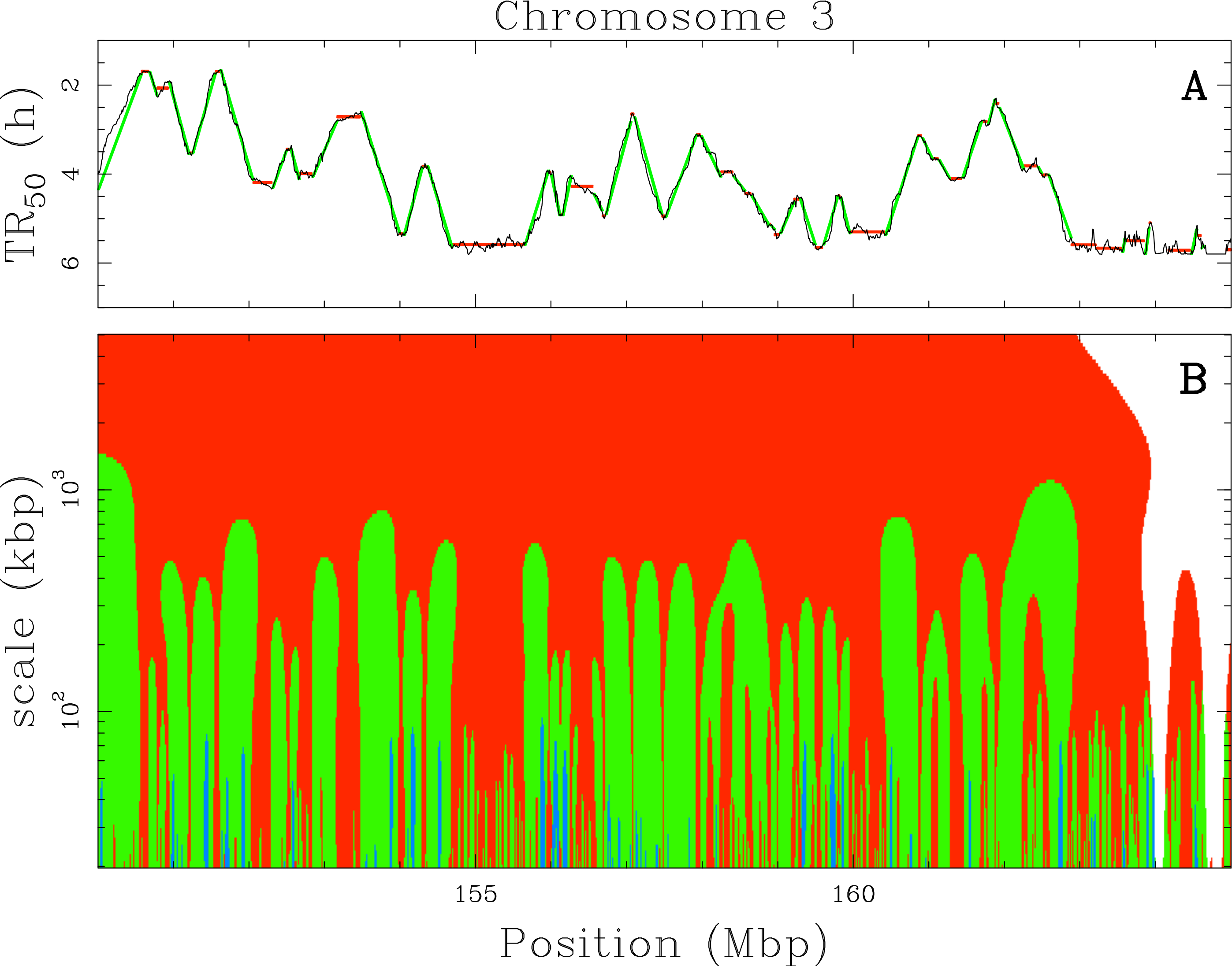

Chromosome 3
A
B
TR50 (h)
2
4
6
scale (kbp)
10^3
10^2
155
160
Position (Mbp)

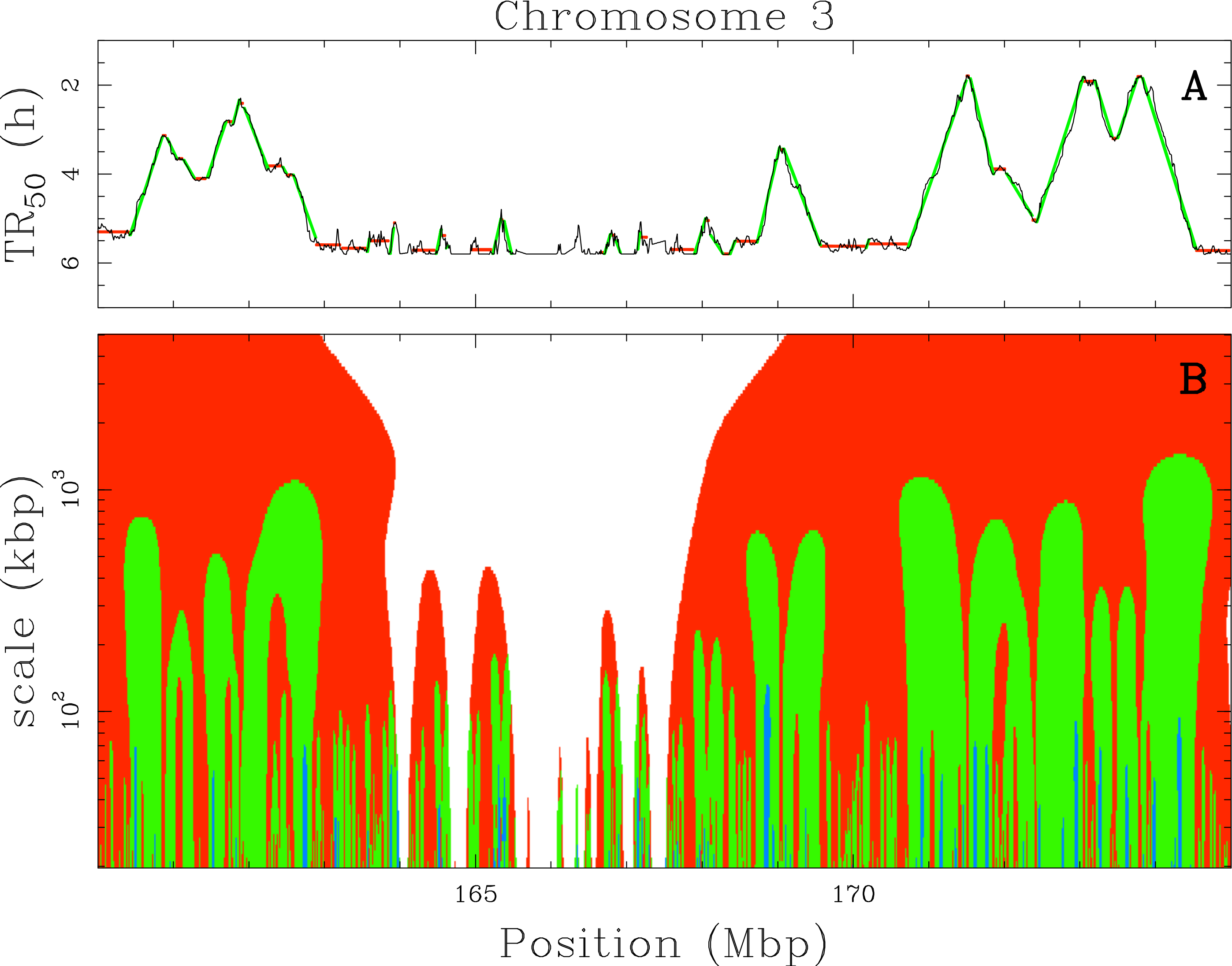
Chromosome 3
A
B
TR50 (h)
2
4
6
scale (kbp)
10^3
10^2
165
170
Position (Mbp)

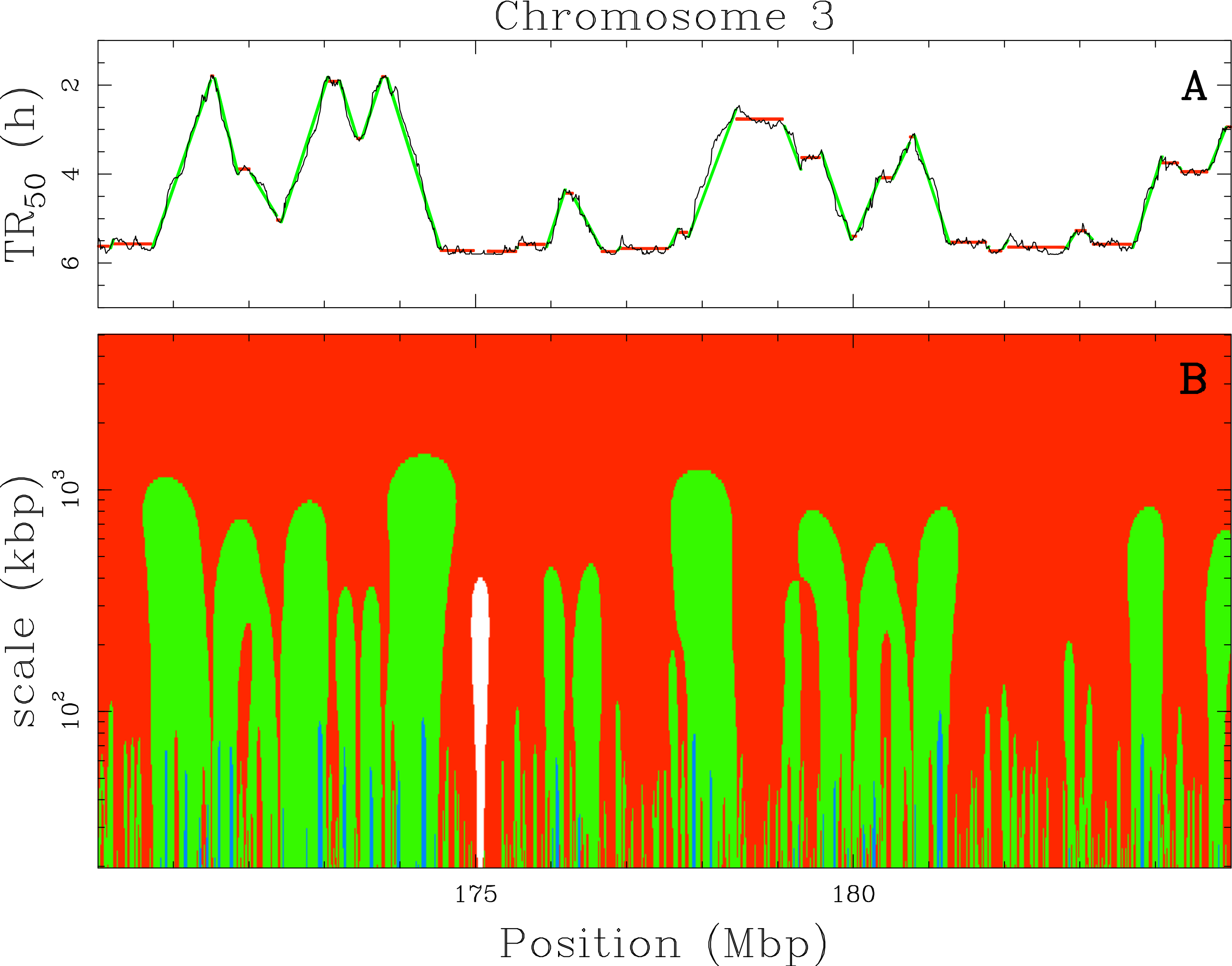

Chromosome 3
A
TR50 (h)
2
4
6
B
scale (kbp)
10^3
10^2
175
180
Position (Mbp)

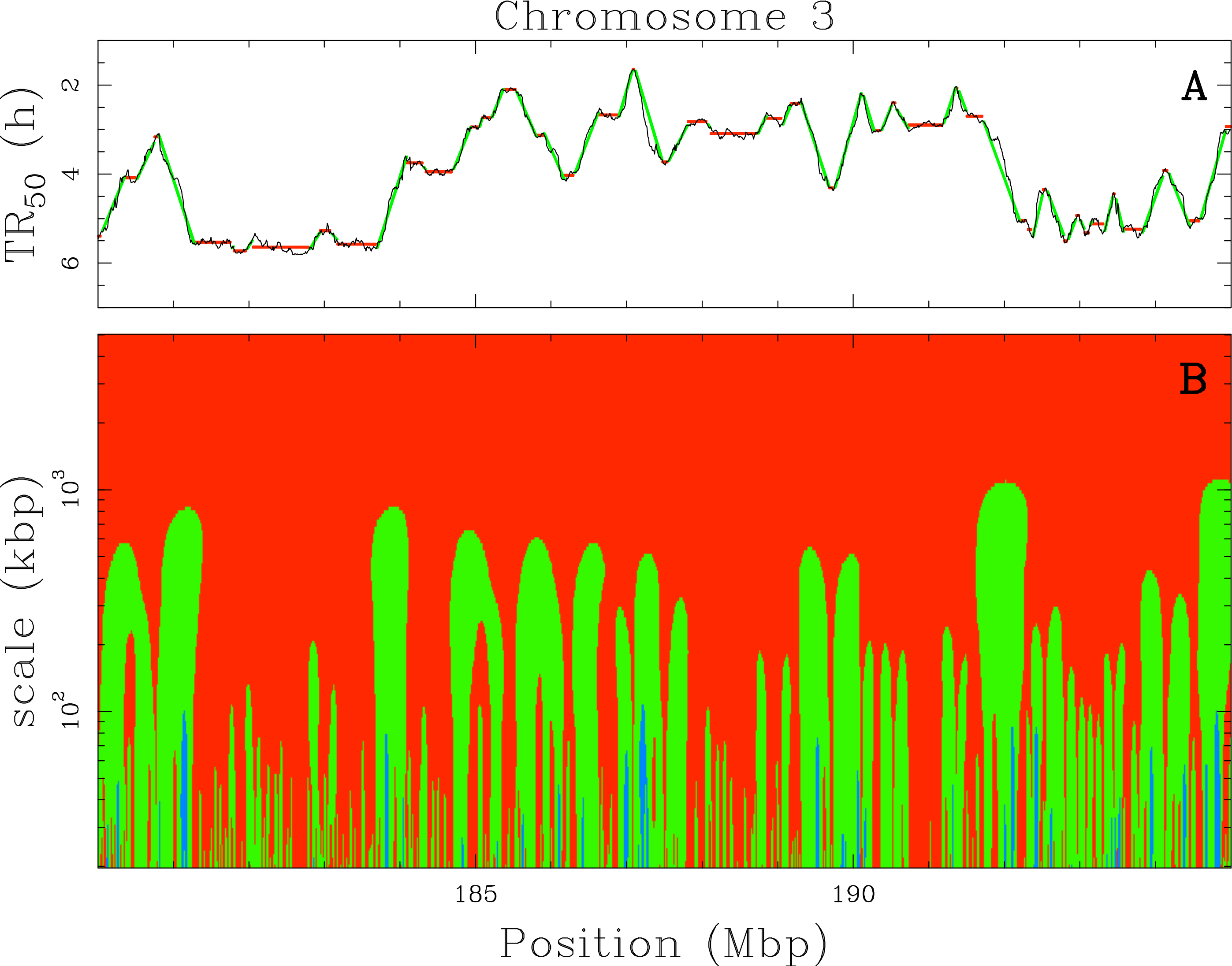

Chromosome 3
A
B
TR_{50} (h)
2
4
6
scale (kbp)
10^3
10^2
185
190
Position (Mbp)

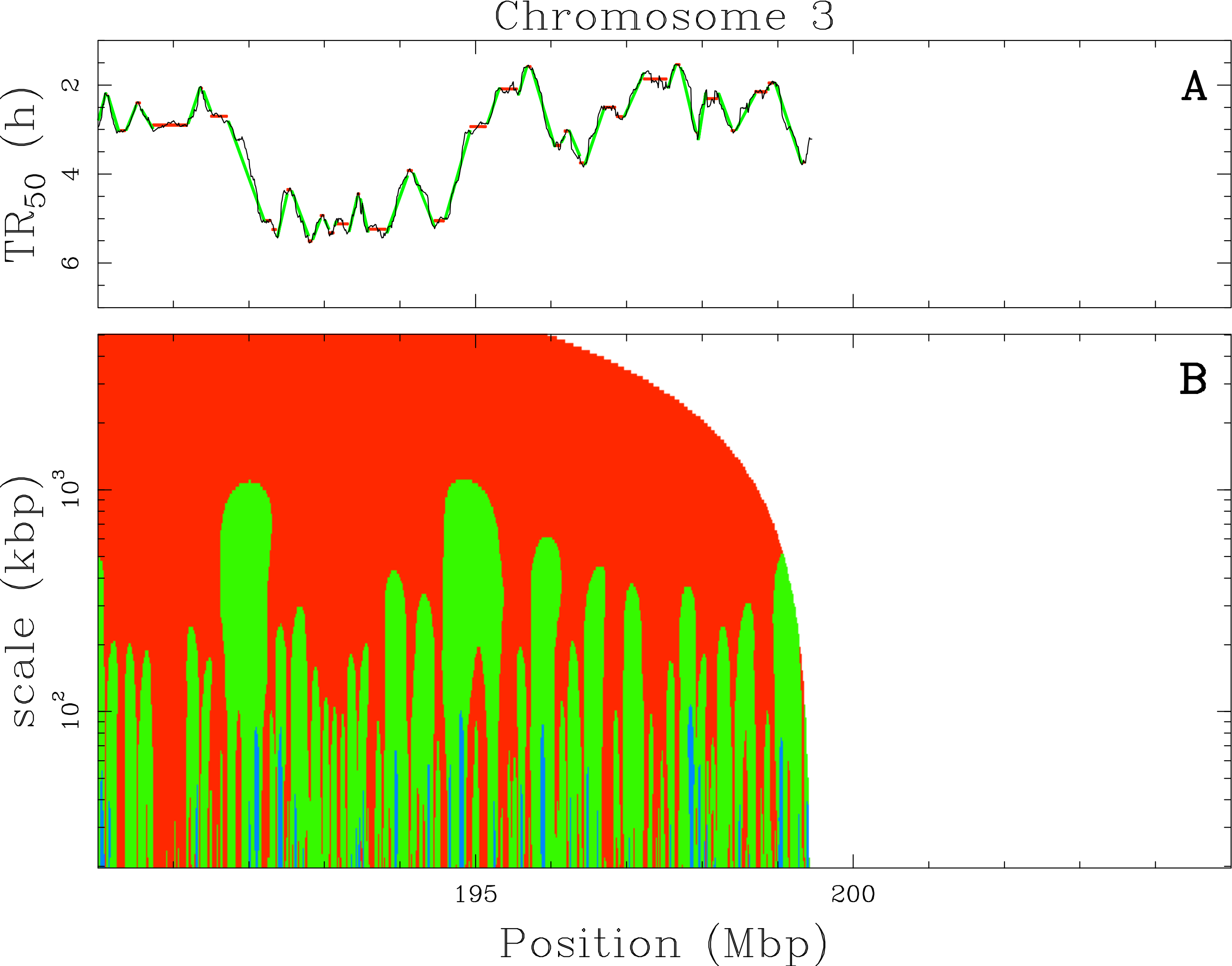

Chromosome 3
TR_{50} (h)
2
4
6
A
B
scale (kbp)
10^3
10^2
195
200
Position (Mbp)

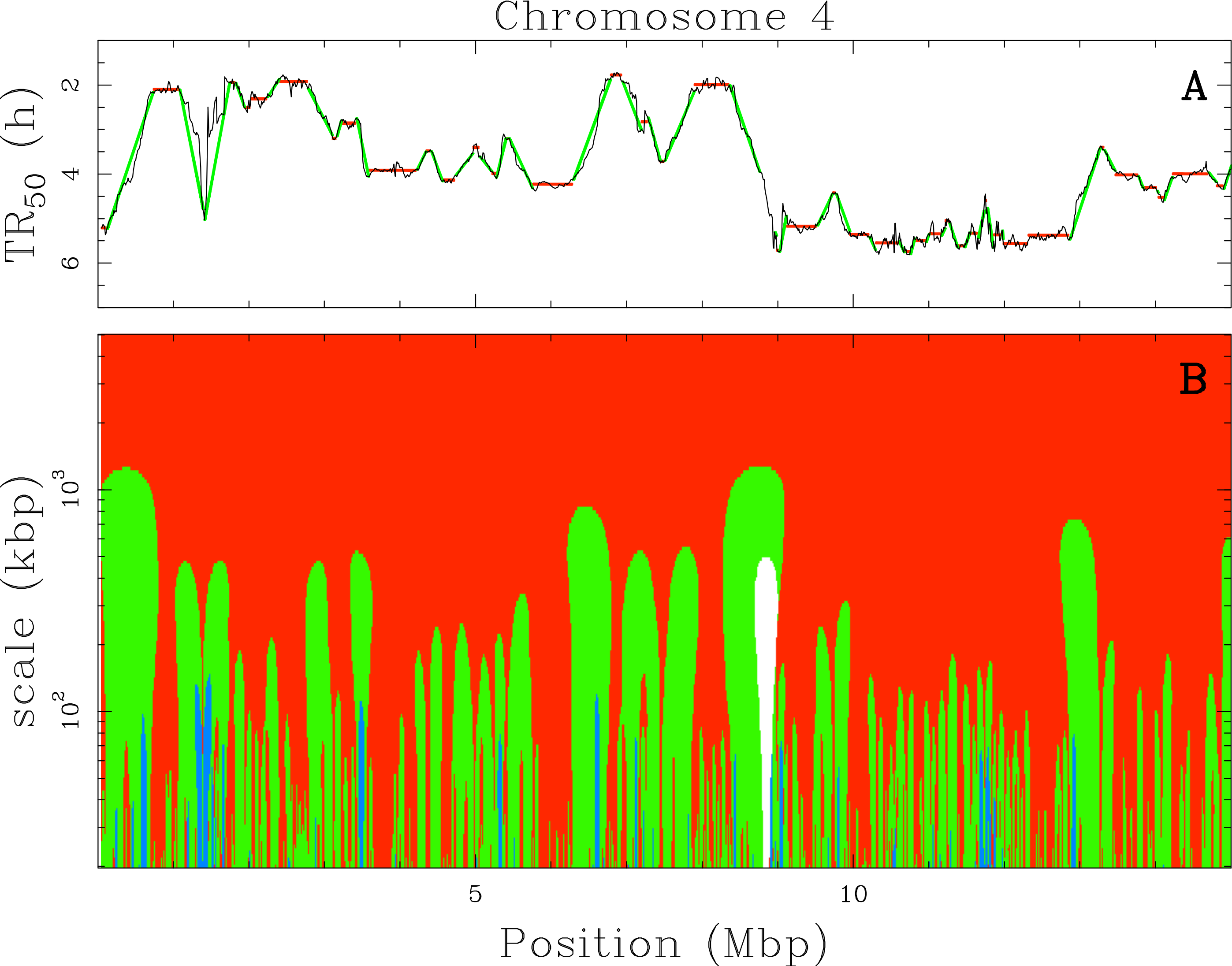

Chromosome 4
A
B
TR50 (h)
2
4
6
scale (kbp)
10^3
10^2
5
10
Position (Mbp)

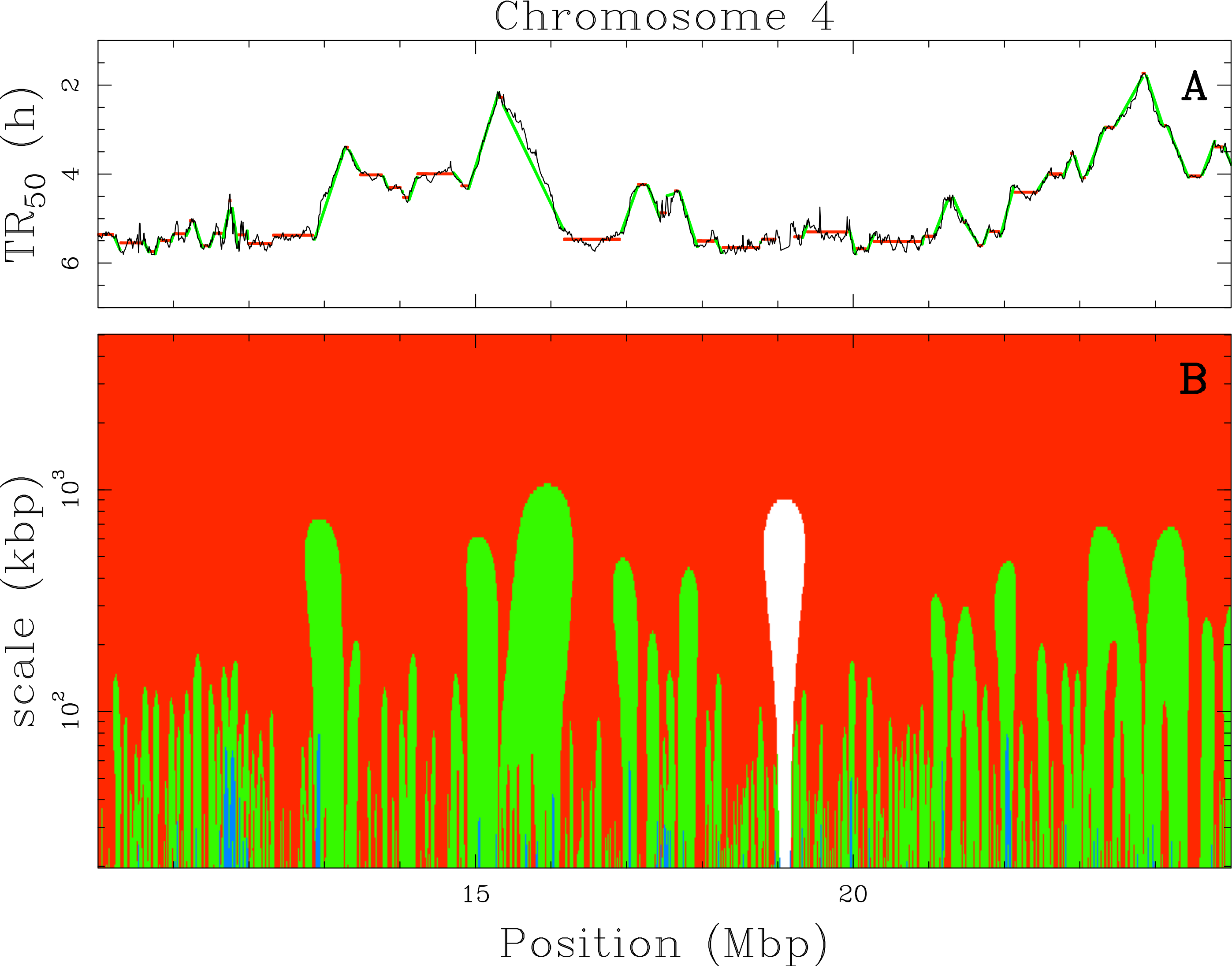

Chromosome 4
A
B
TR_{50} (h)
2
4
6
scale (kbp)
10^3
10^2
15
20
Position (Mbp)

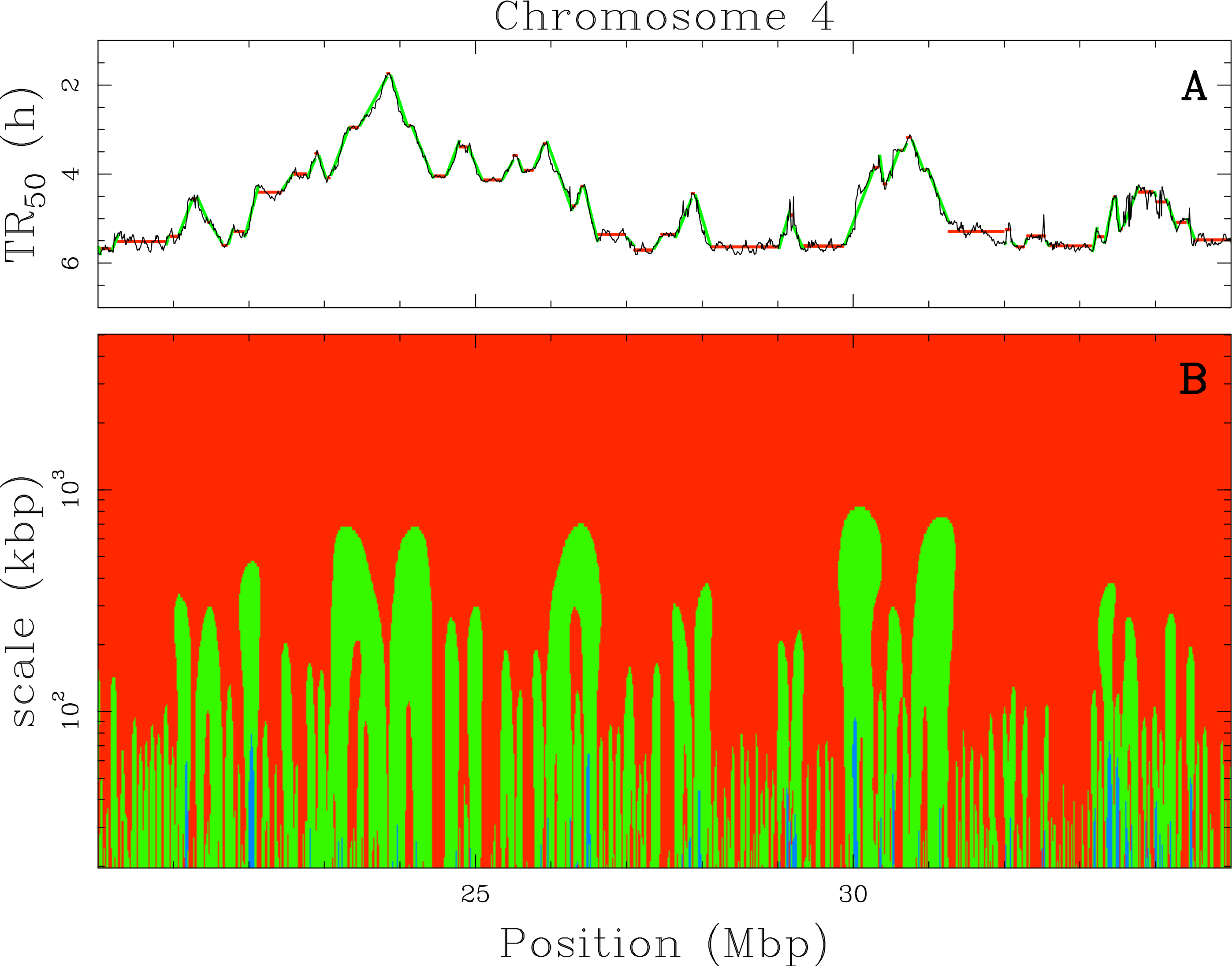
Chromosome 4
TR50 (h)
2
4
6
A
B
scale (kbp)
10^3
10^2
25
30
Position (Mbp)

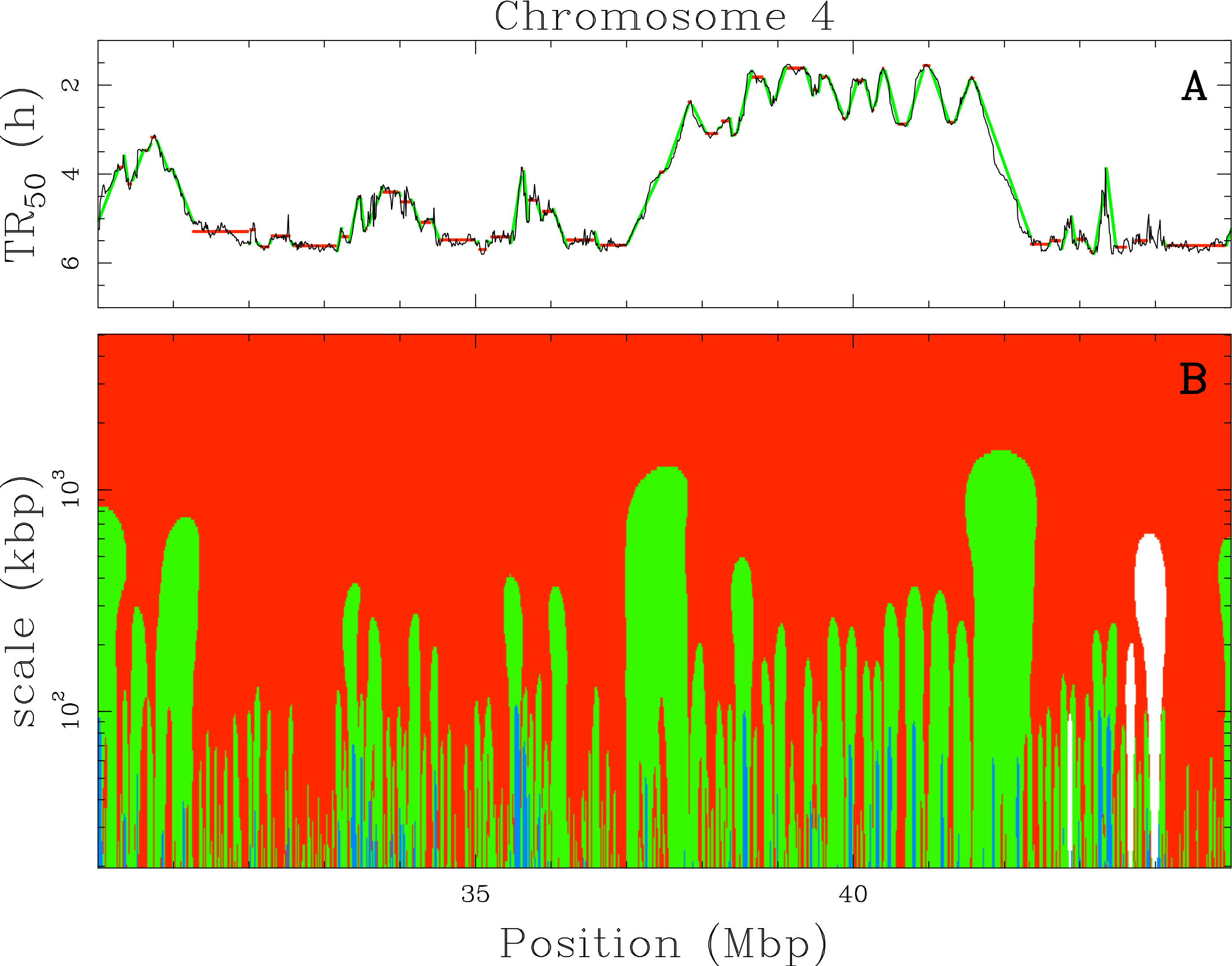

Chromosome 4
A
B
TR50 (h)
2
4
6
scale (kbp)
10^3
10^2
35
40
Position (Mbp)

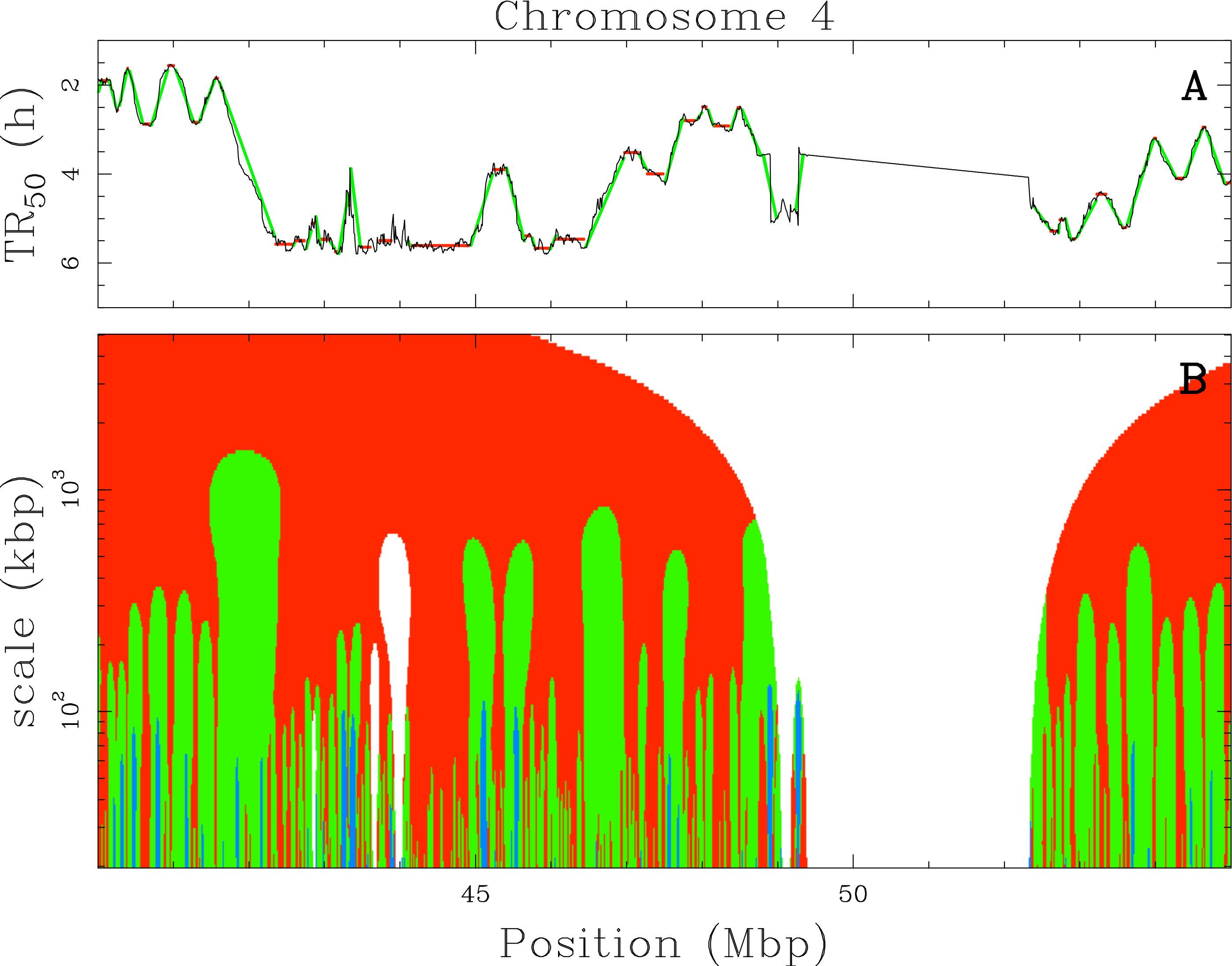

Chromosome 4
A
B
TR50 (h)
2
4
6
scale (kbp)
10^3
10^2
45
50
Position (Mbp)

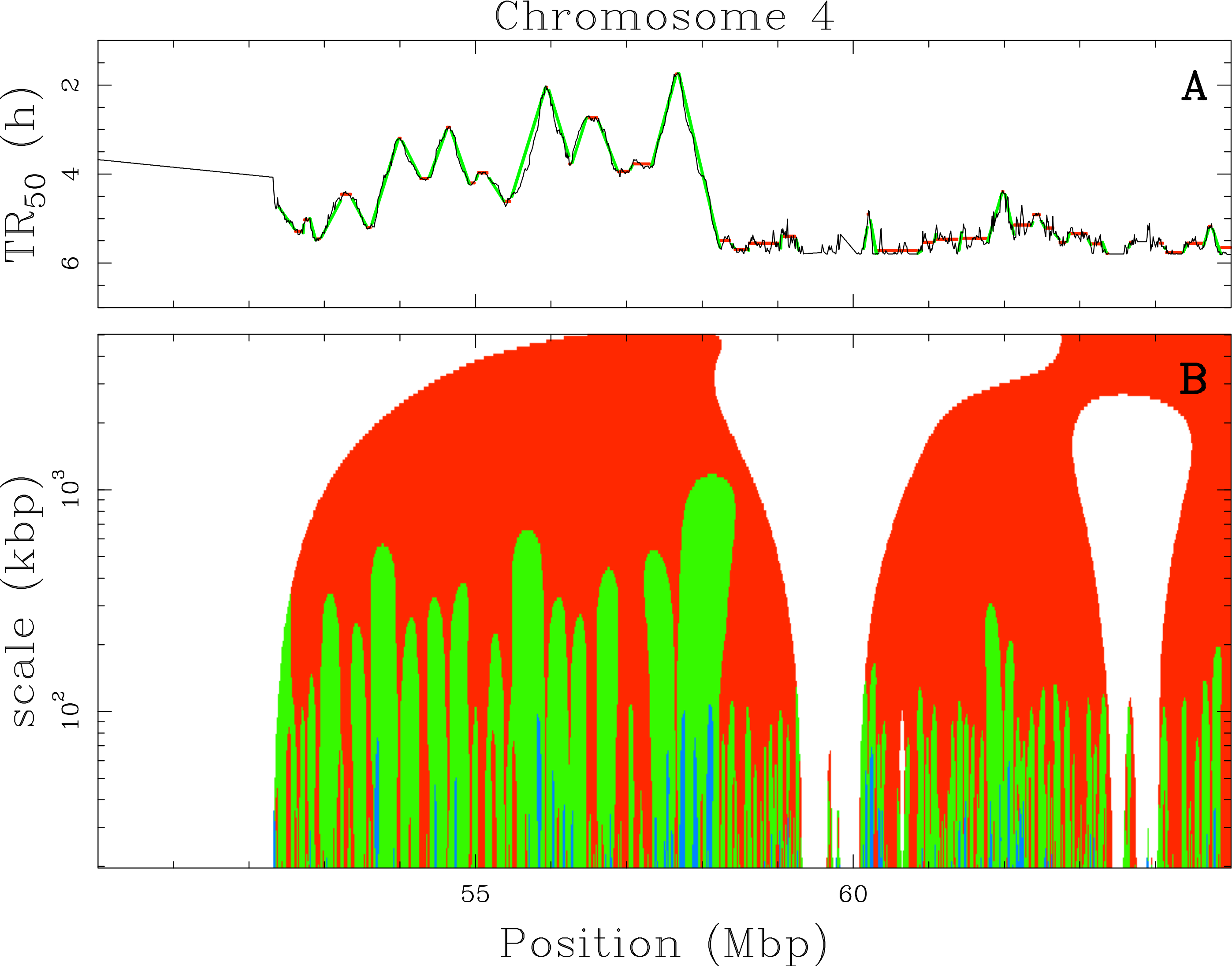

Chromosome 4
A
B
TR_{50} (h)
2
4
6
scale (kbp)
10^3
10^2
55
60
Position (Mbp)

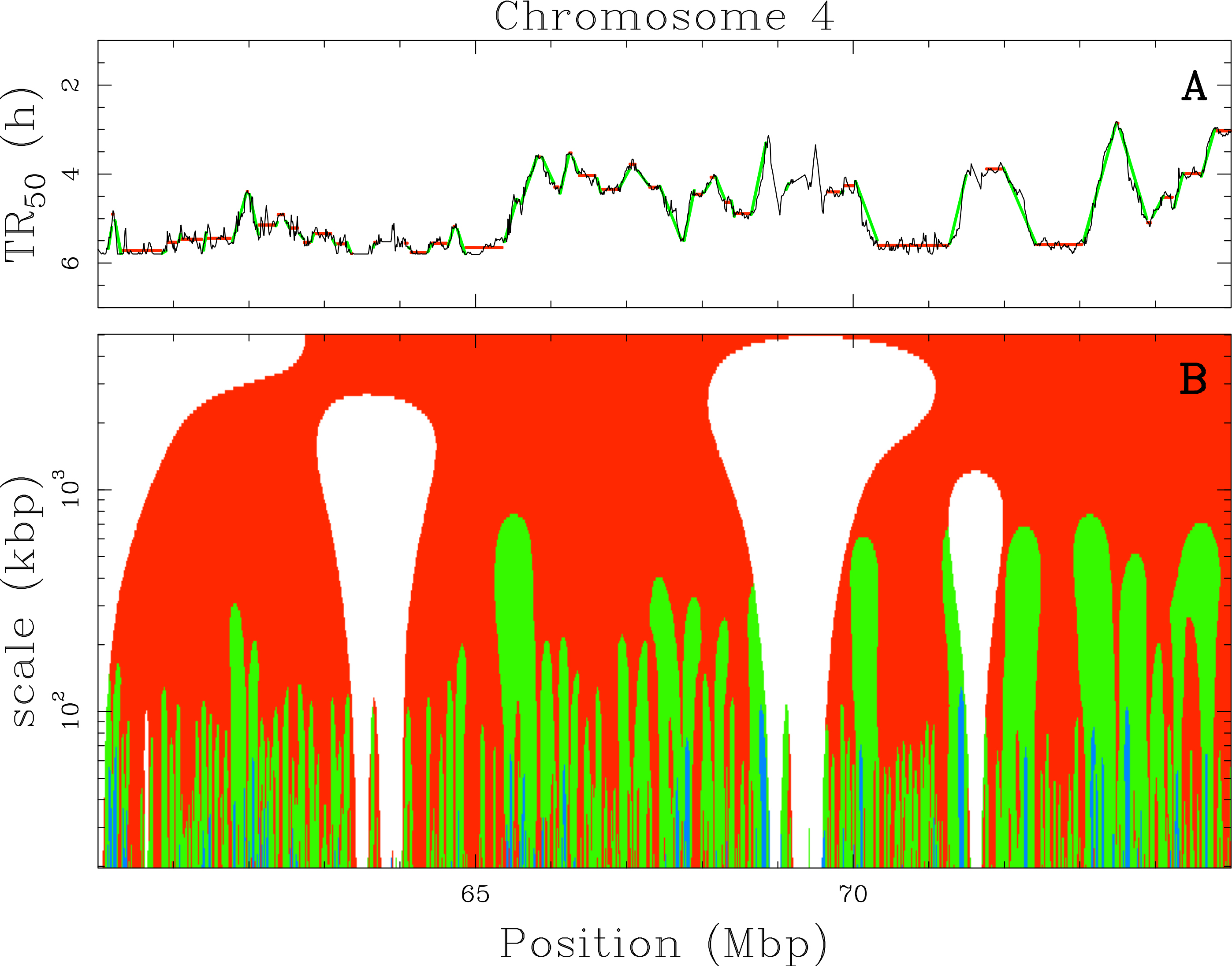

Chromosome 4
A
TR50 (h)
2
4
6
B
scale (kbp)
10^3
10^2
65
70
Position (Mbp)

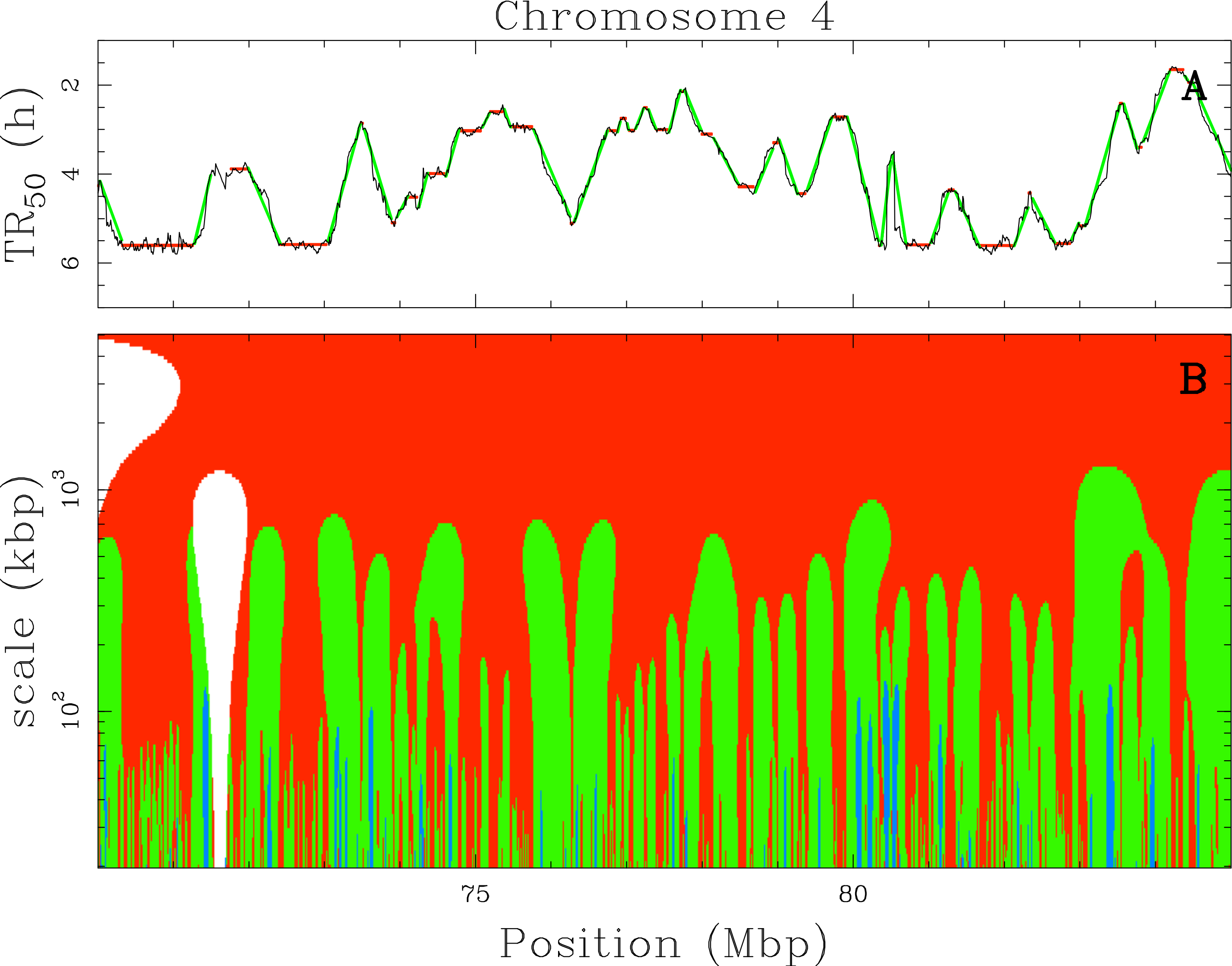

Chromosome 4
TR50 (h)
2
4
6
A
B
scale (kbp)
10^3
10^2
75
80
Position (Mbp)

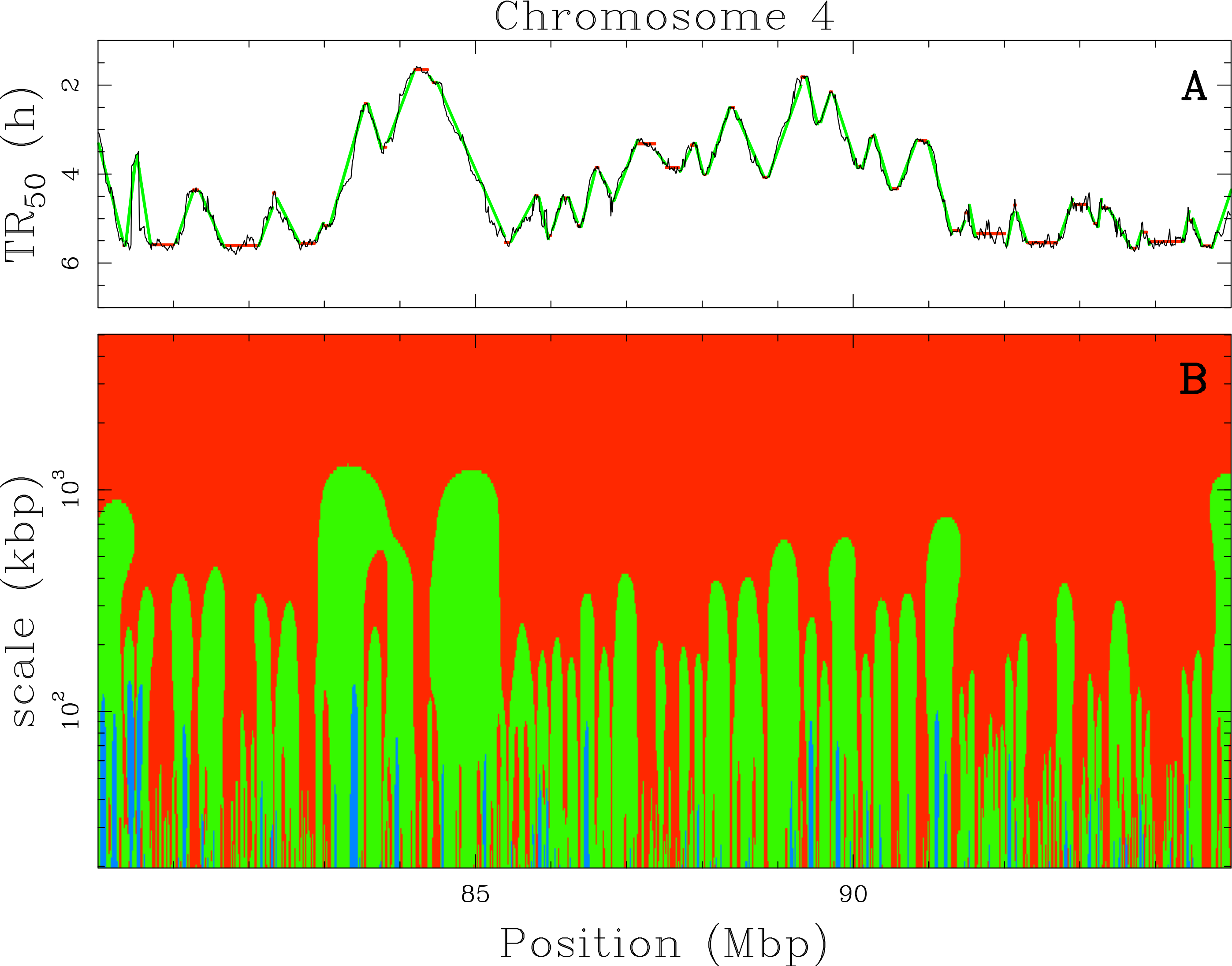

Chromosome 4
A
B
TR50 (h)
2
4
6
scale (kbp)
10^3
10^2
85
90
Position (Mbp)

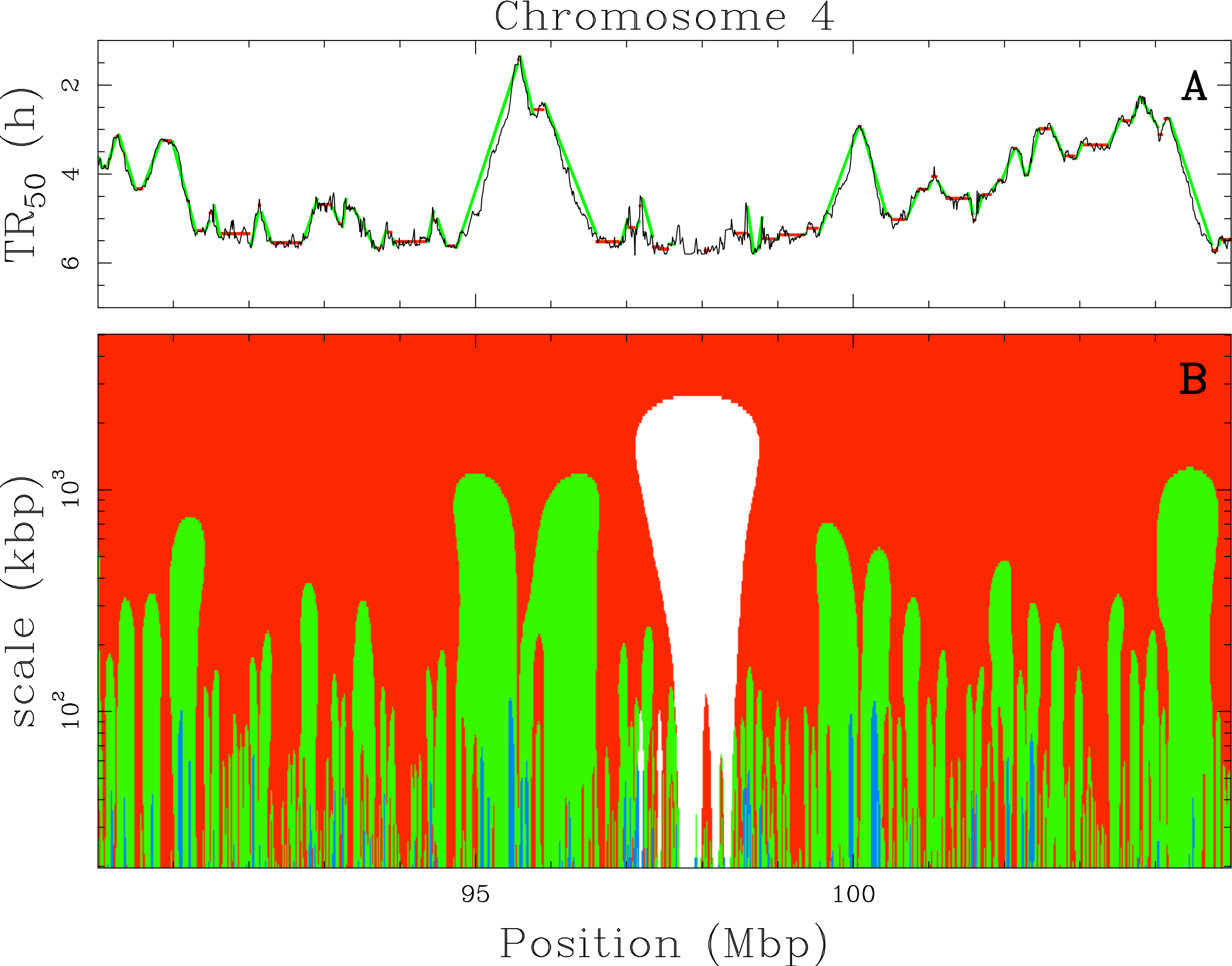
Chromosome 4
A
B
TR50 (h)
2
4
6
scale (kbp)
10^3
10^2
95
100
Position (Mbp)

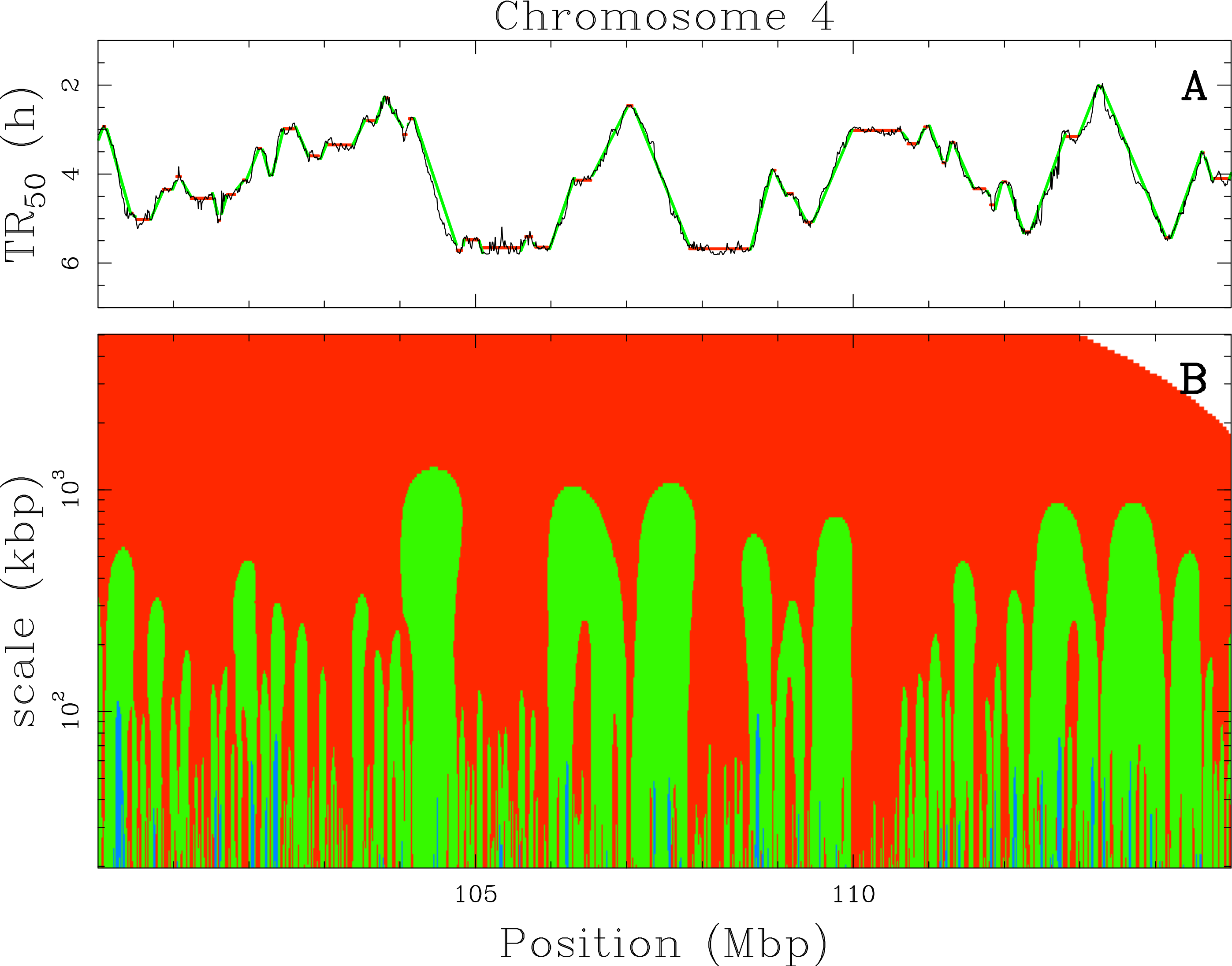
Chromosome 4
TR50 (h)
2
4
6
A
B
scale (kbp)
10^3
10^2
105
110
Position (Mbp)

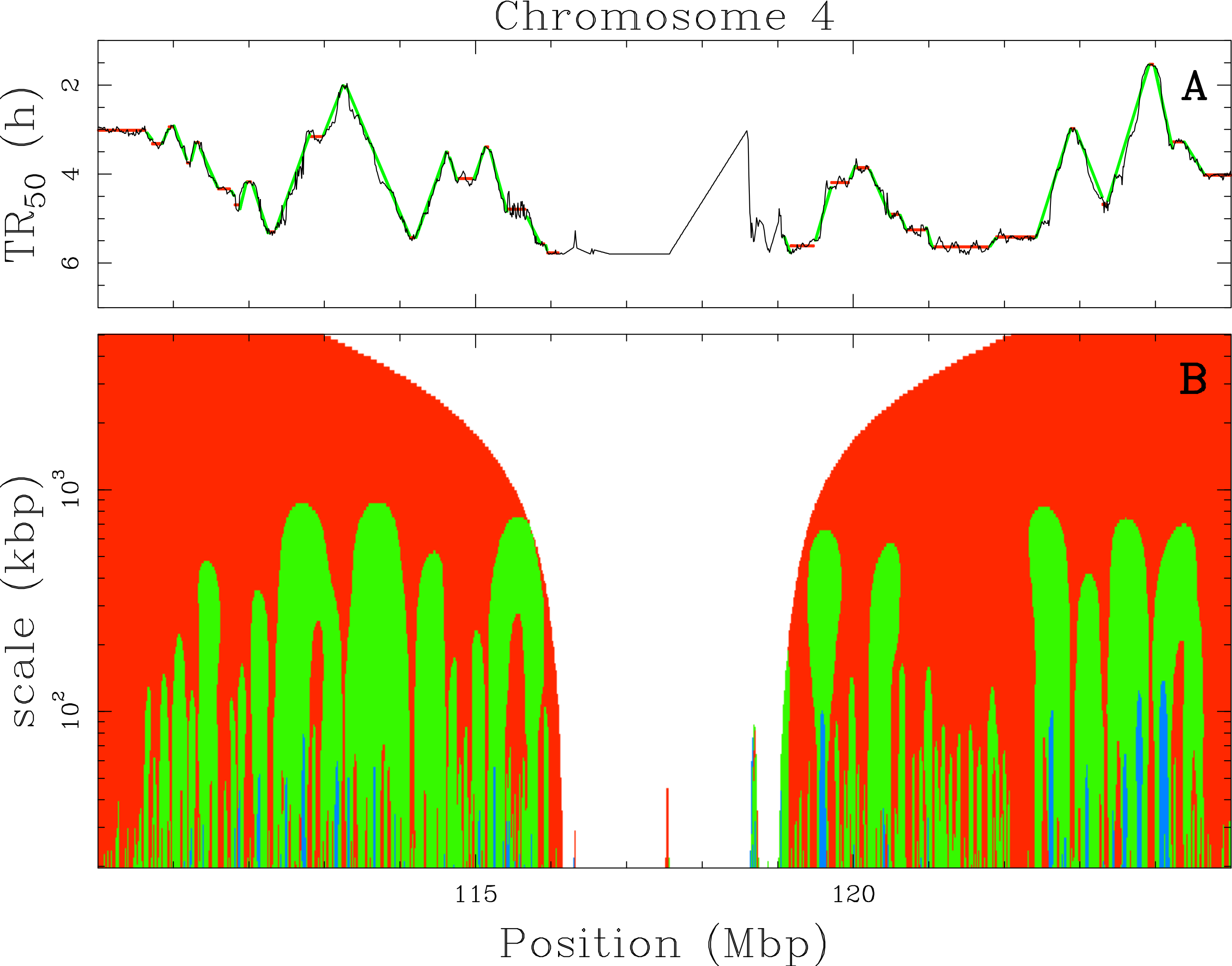

Chromosome 4
A
TR50 (h)
2
4
6
B
scale (kbp)
10^3
10^2
115
120
Position (Mbp)

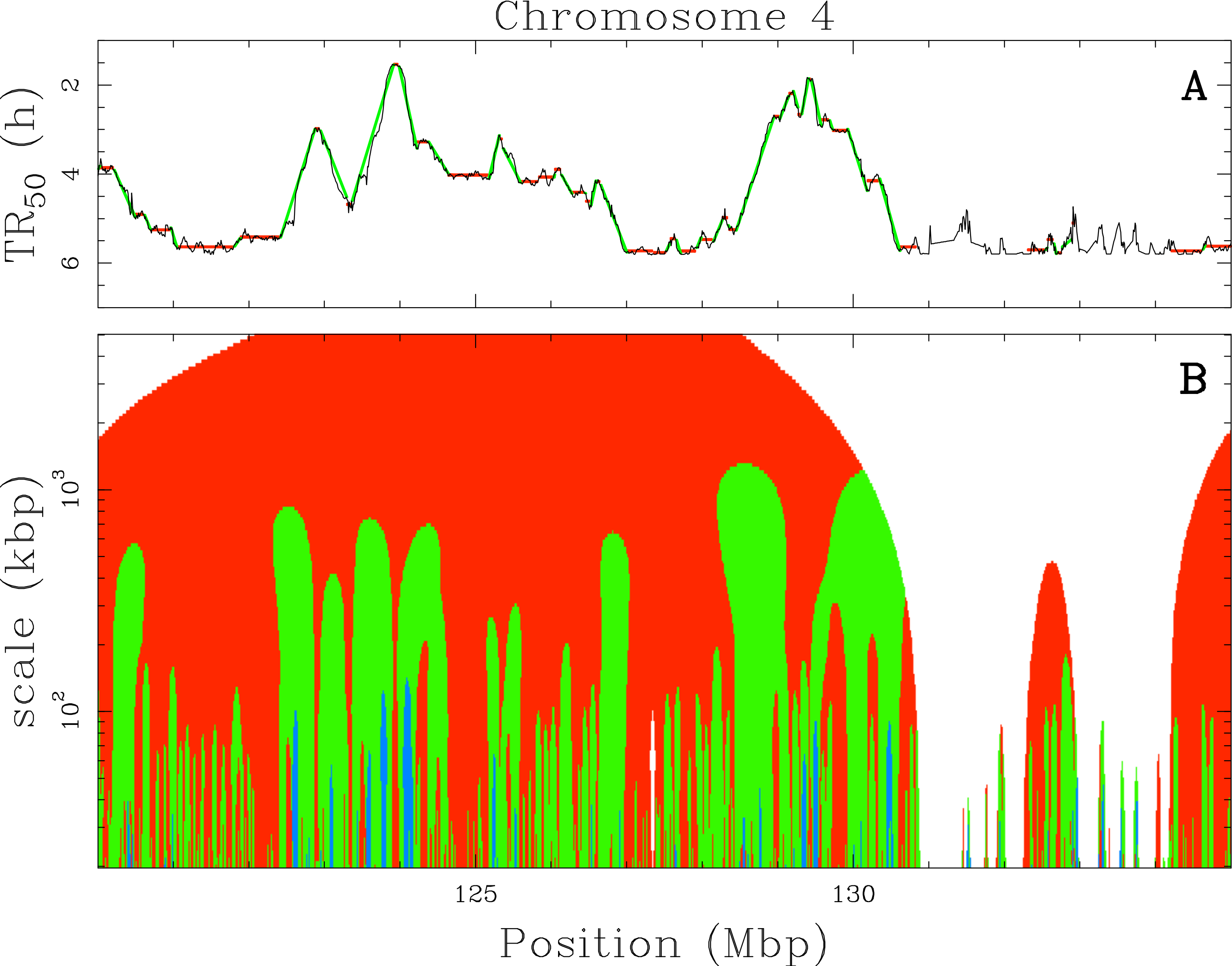
Chromosome 4
A
B
TR_{50} (h)
2
4
6
scale (kbp)
10^3
10^2
125
130
Position (Mbp)

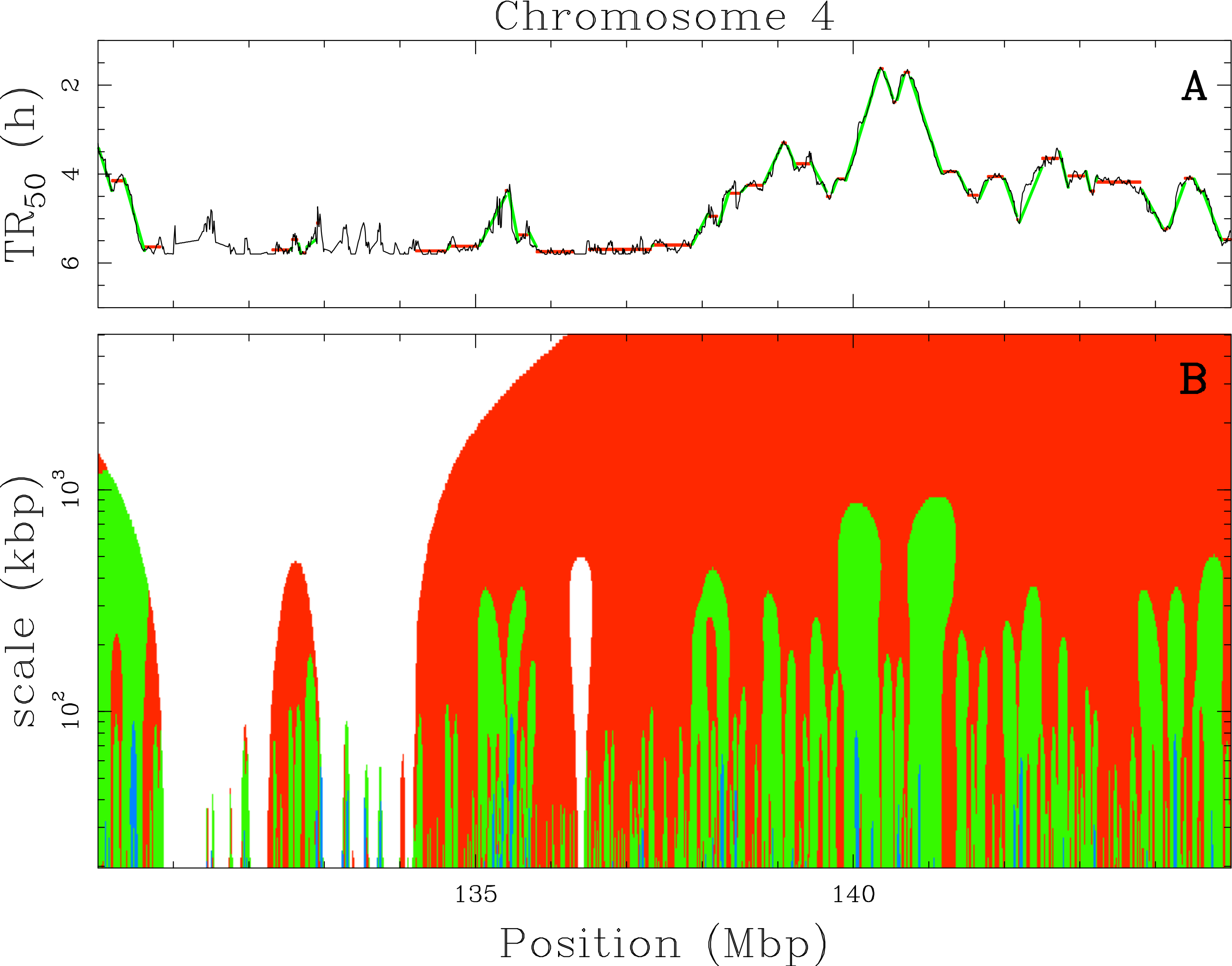

Chromosome 4
A
B
TR_{50} (h)
2
4
6
scale (kbp)
10^3
10^2
135
140
Position (Mbp)

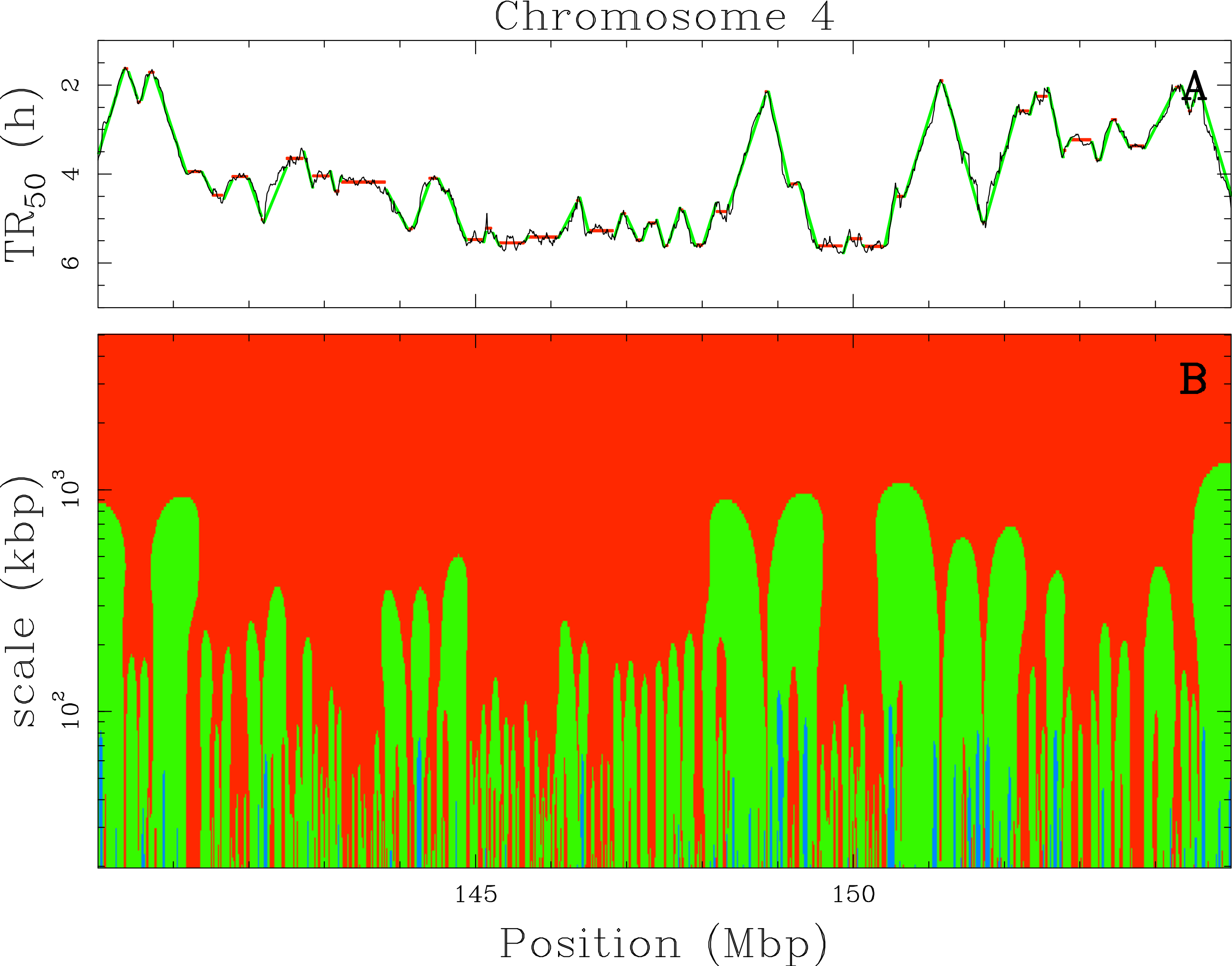

Chromosome 4
A
B
TR_{50} (h)
2
4
6
scale (kbp)
10^3
10^2
145
150
Position (Mbp)

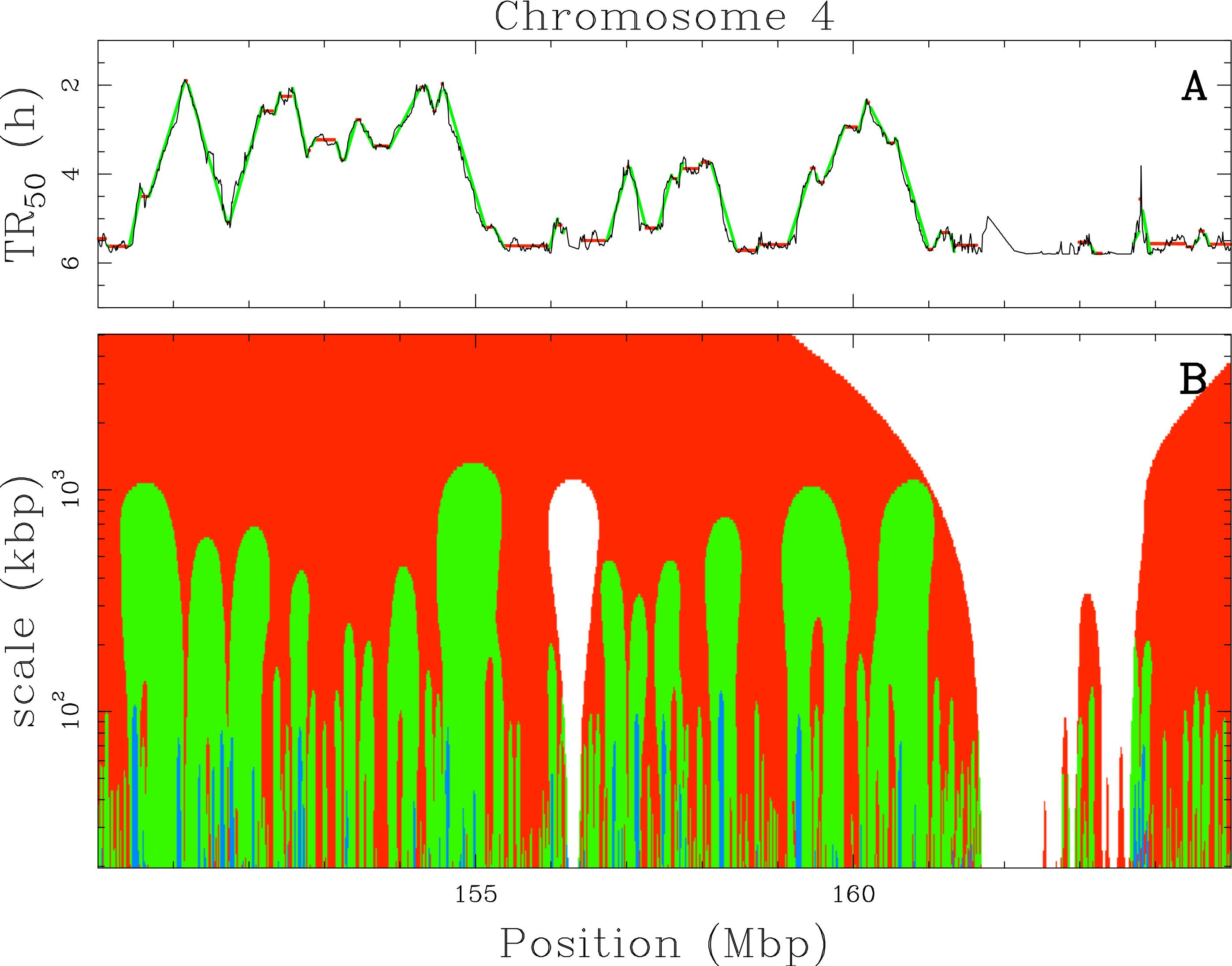

Chromosome 4
A
B
TR_{50} (h)
2
4
6
scale (kbp)
10^3
10^2
155
160
Position (Mbp)

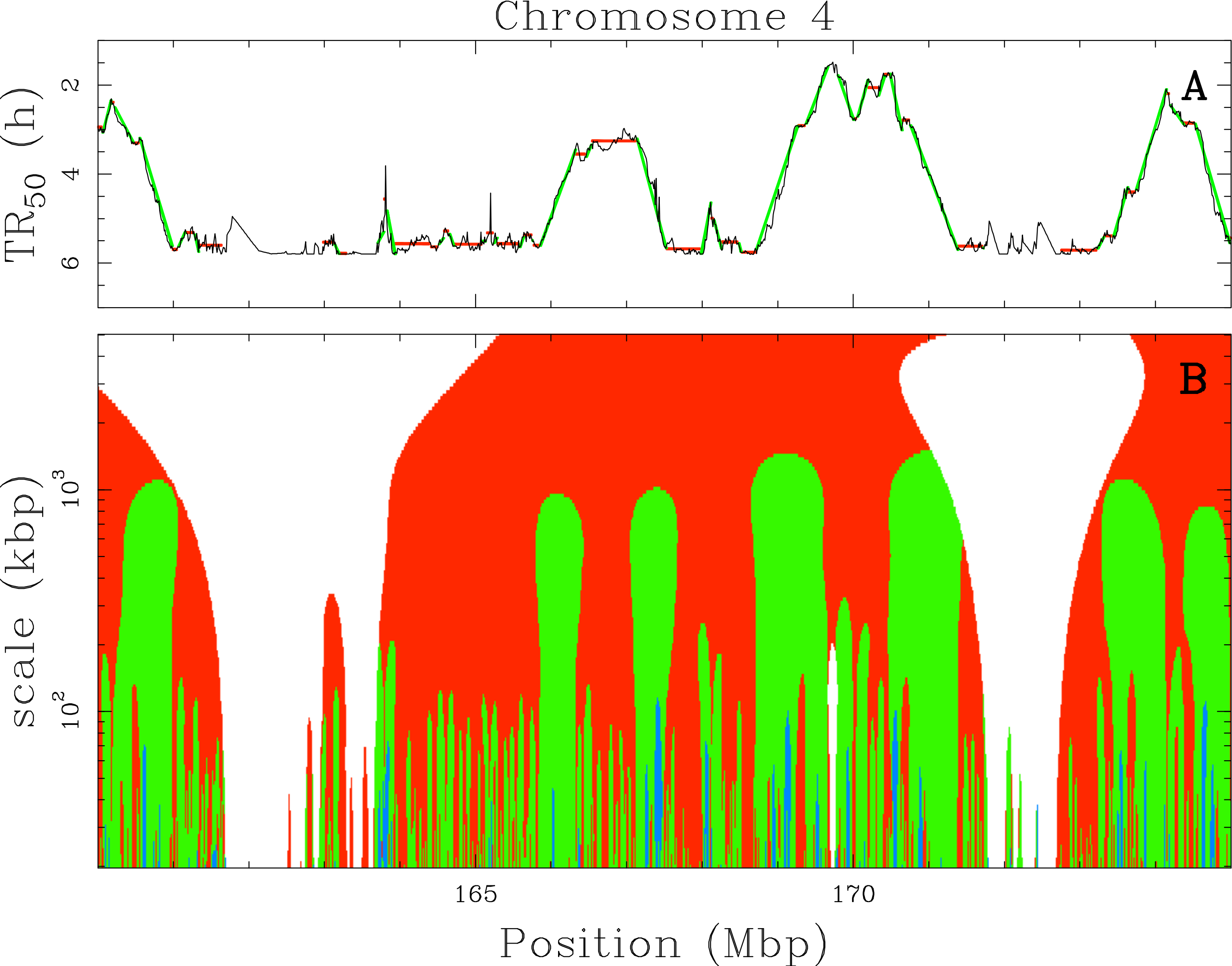
Chromosome 4
A
B
TR50 (h)
2
4
6
scale (kbp)
10^3
10^2
165
170
Position (Mbp)

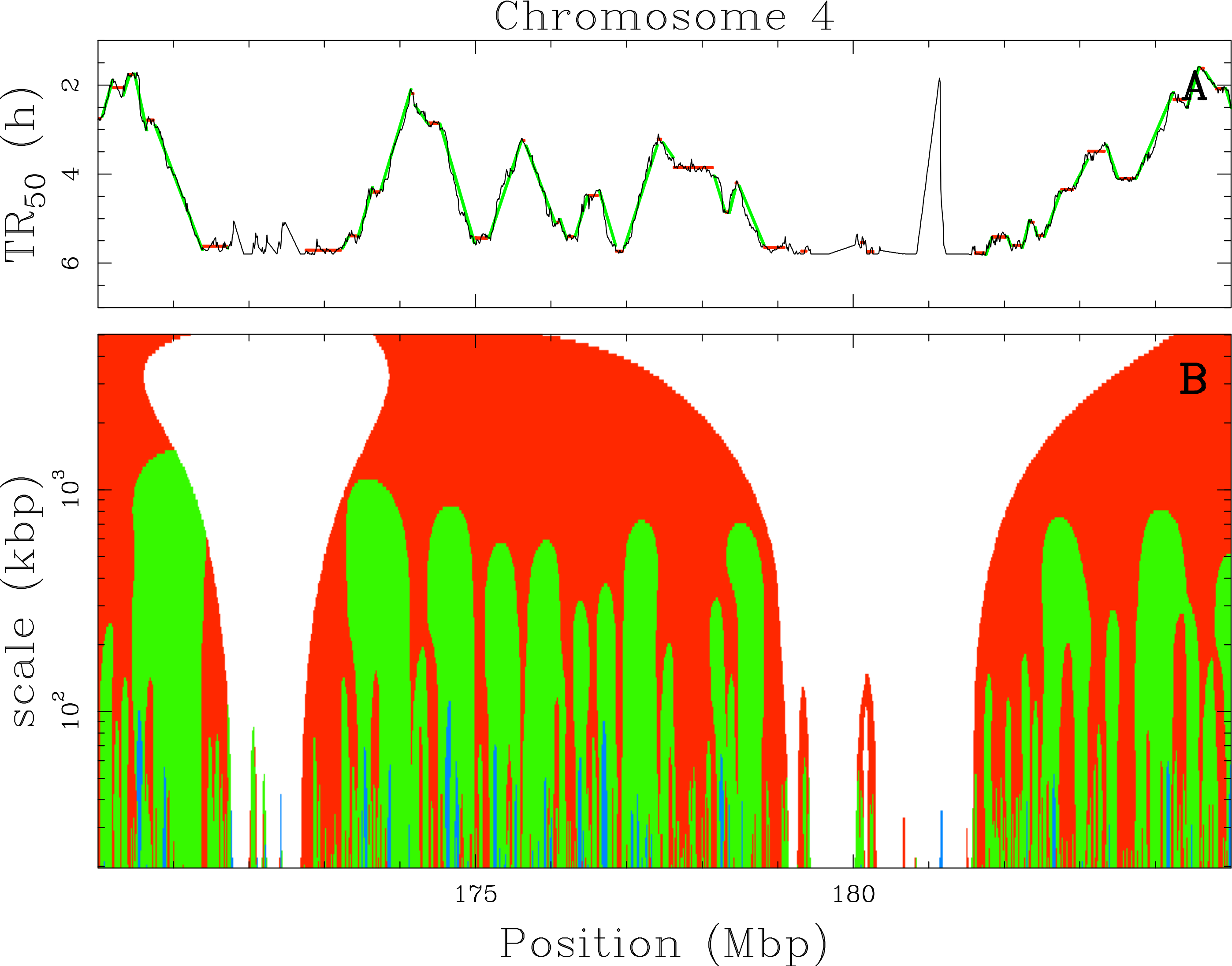
Chromosome 4
A
B
TR_{50} (h)
2
4
6
scale (kbp)
10^3
10^2
175
180
Position (Mbp)

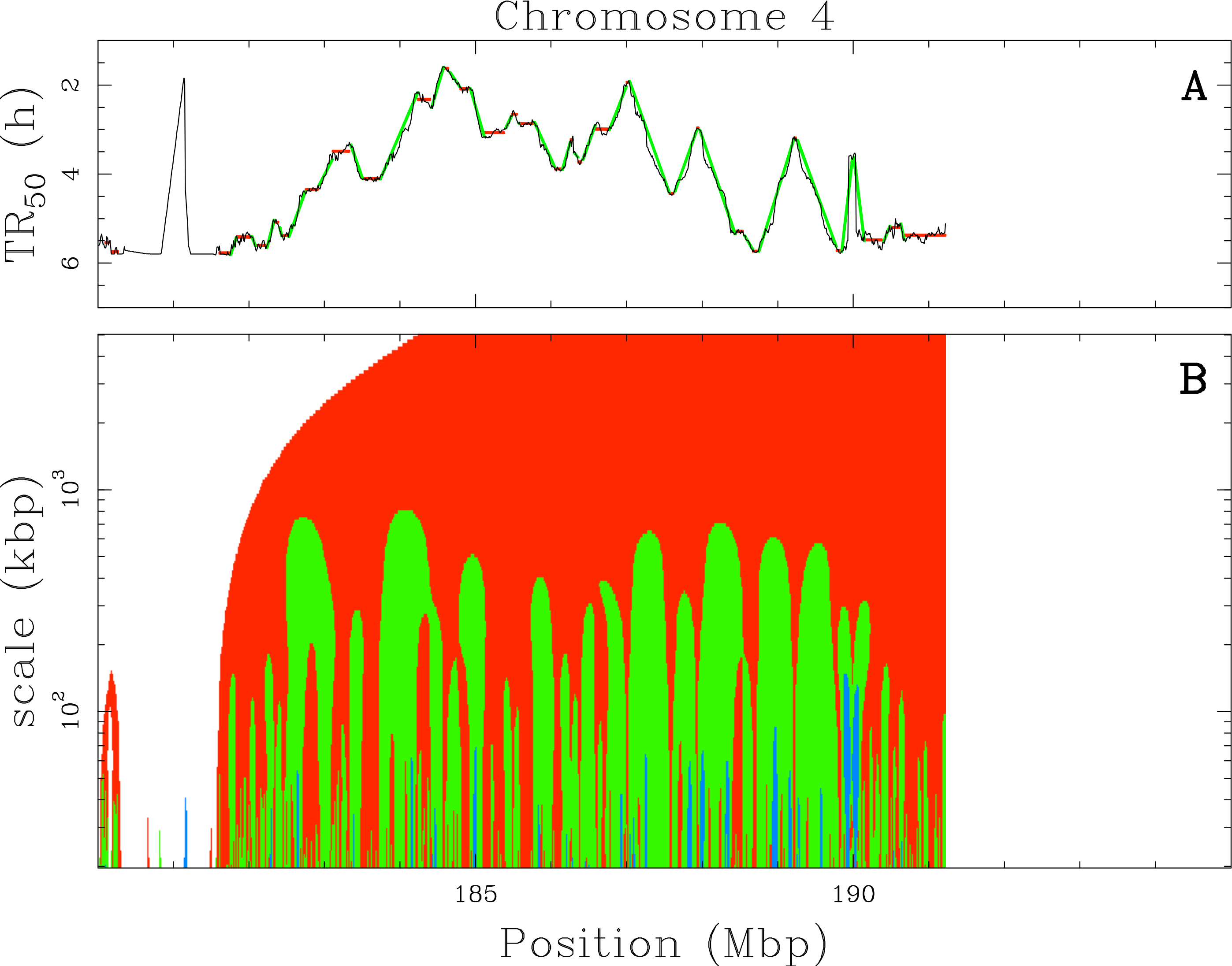

Chromosome 4
A
B
TR_{50} (h)
2
4
6
scale (kbp)
10^3
10^2
185
190
Position (Mbp)

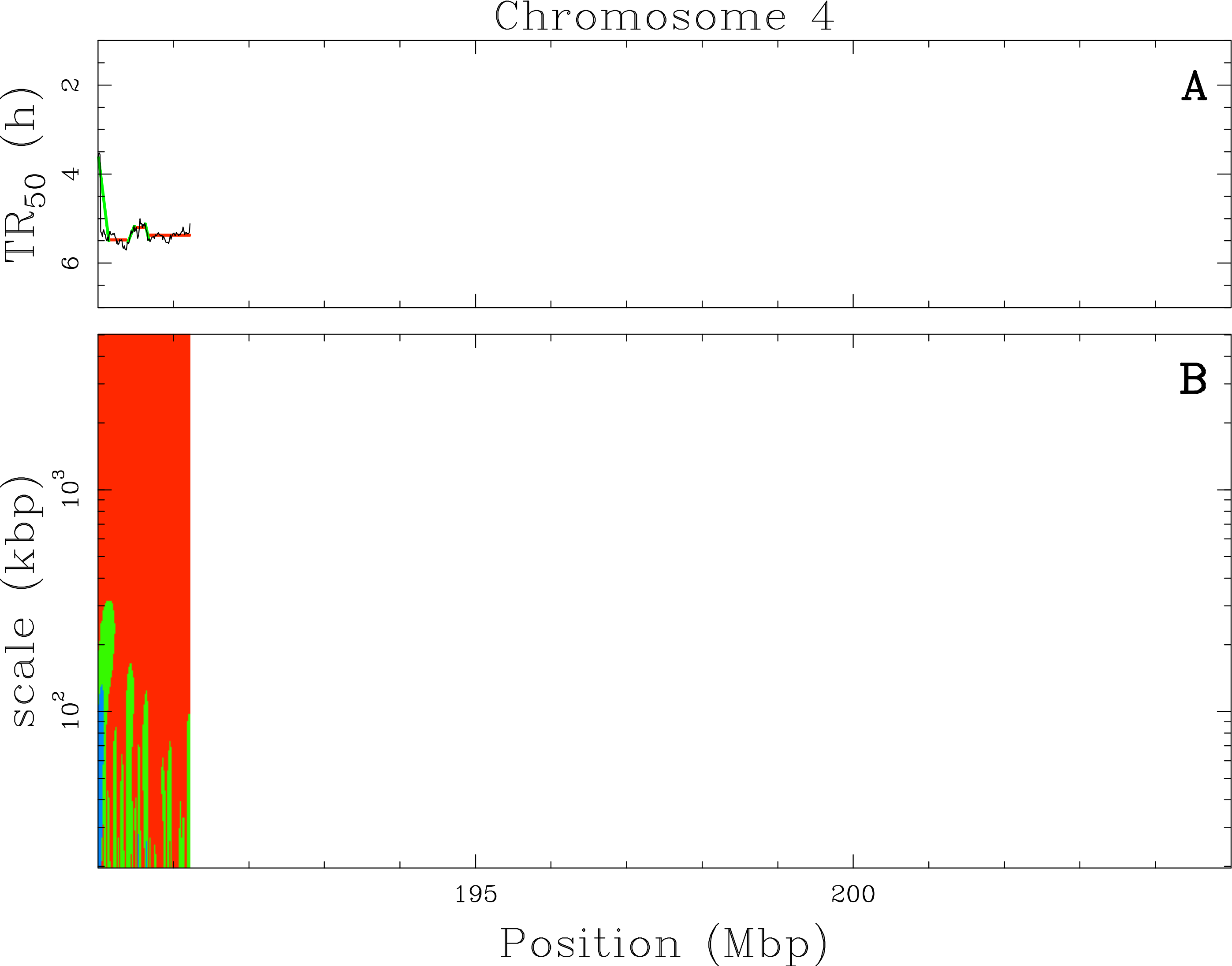

Chromosome 4
A
B
TR50 (h)
2
4
6
scale (kbp)
10^3
10^2
195
200
Position (Mbp)

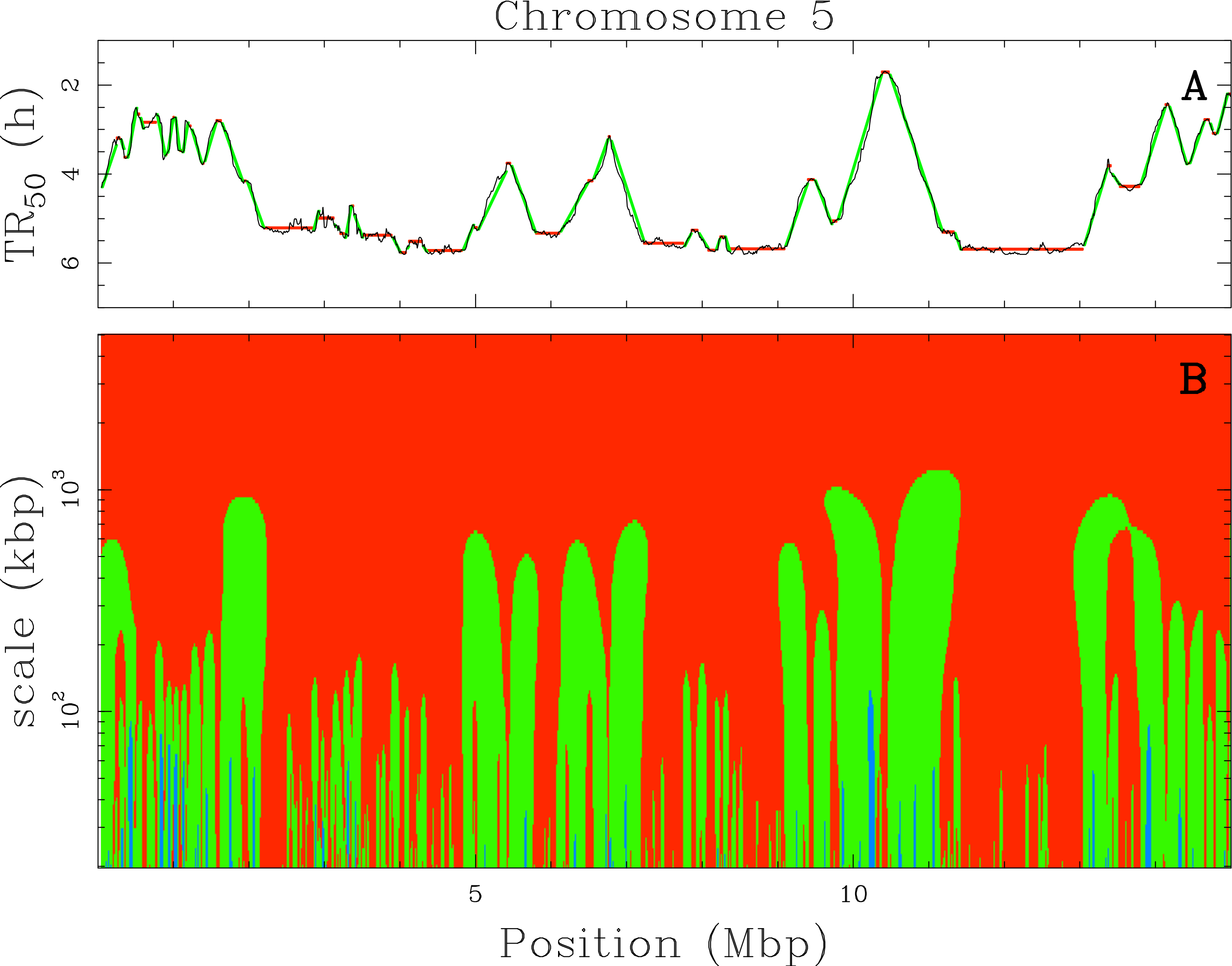

Chromosome 5
A
B
TR50 (h)
2
4
6
scale (kbp)
10^3
10^2
5
10
Position (Mbp)

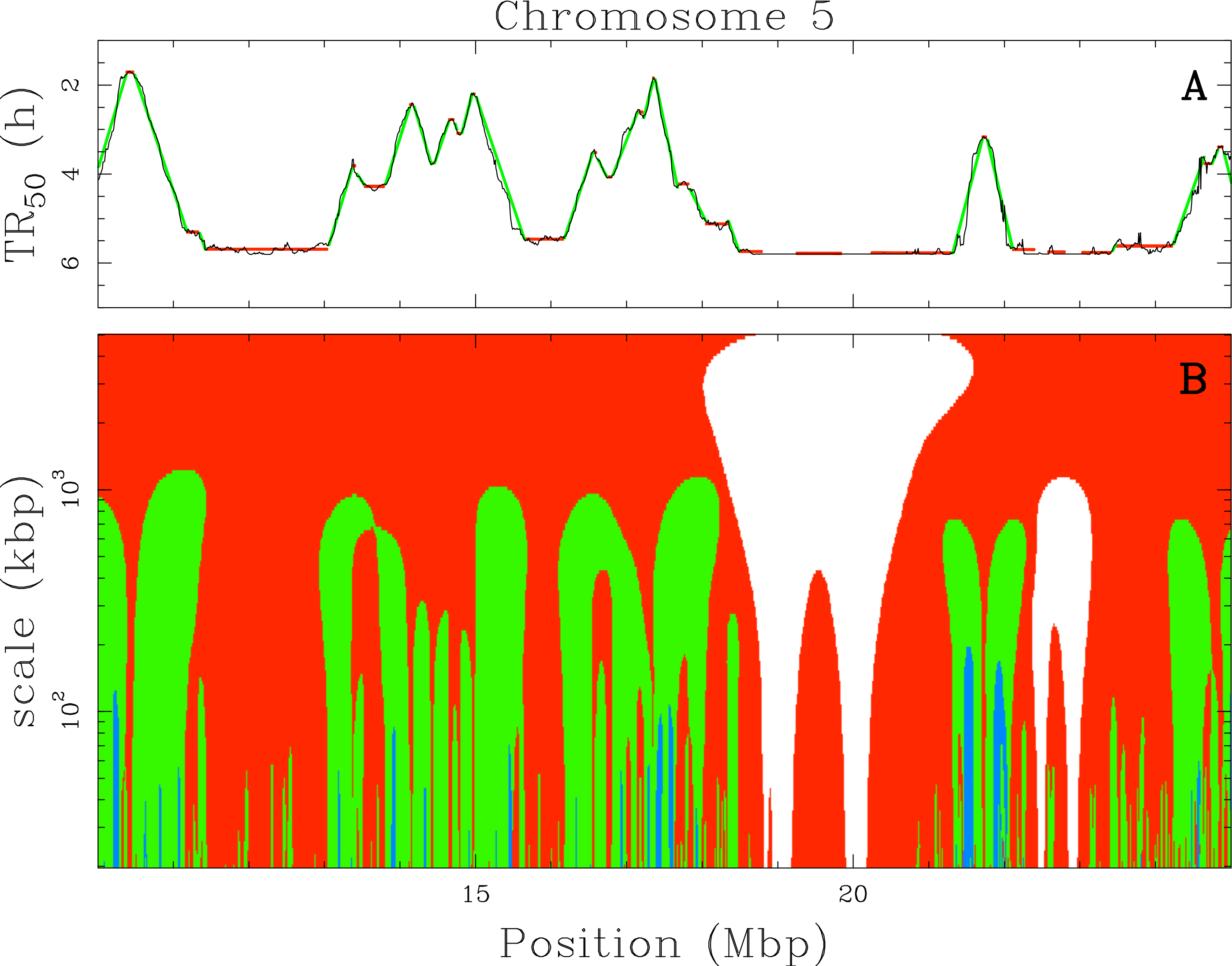

Chromosome 5
A
B
TR_{50} (h)
2
4
6
scale (kbp)
10^3
10^2
15
20
Position (Mbp)

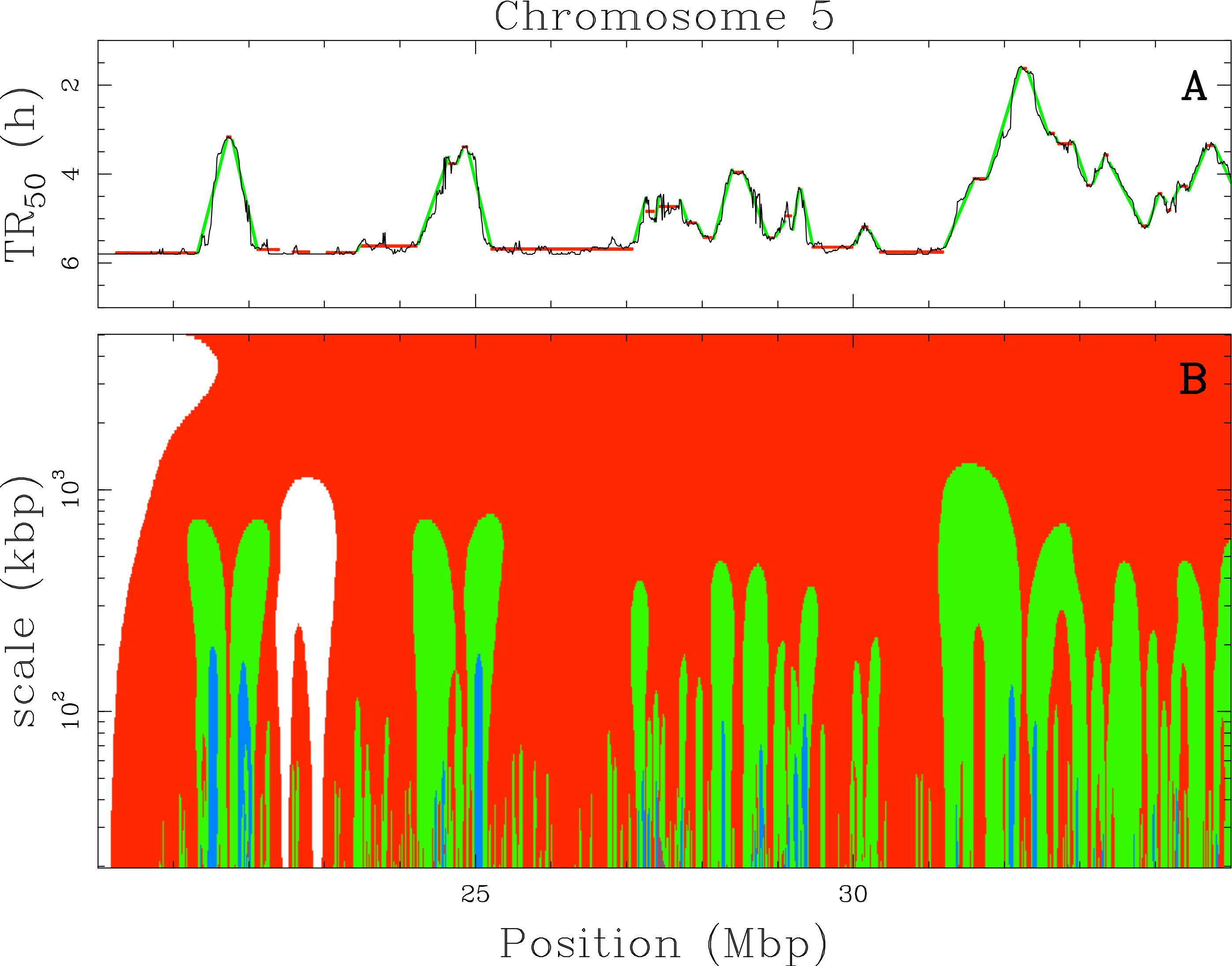

Chromosome 5
A
B
TR_{50} (h)
2
4
6
scale (kbp)
10^3
10^2
25
30
Position (Mbp)

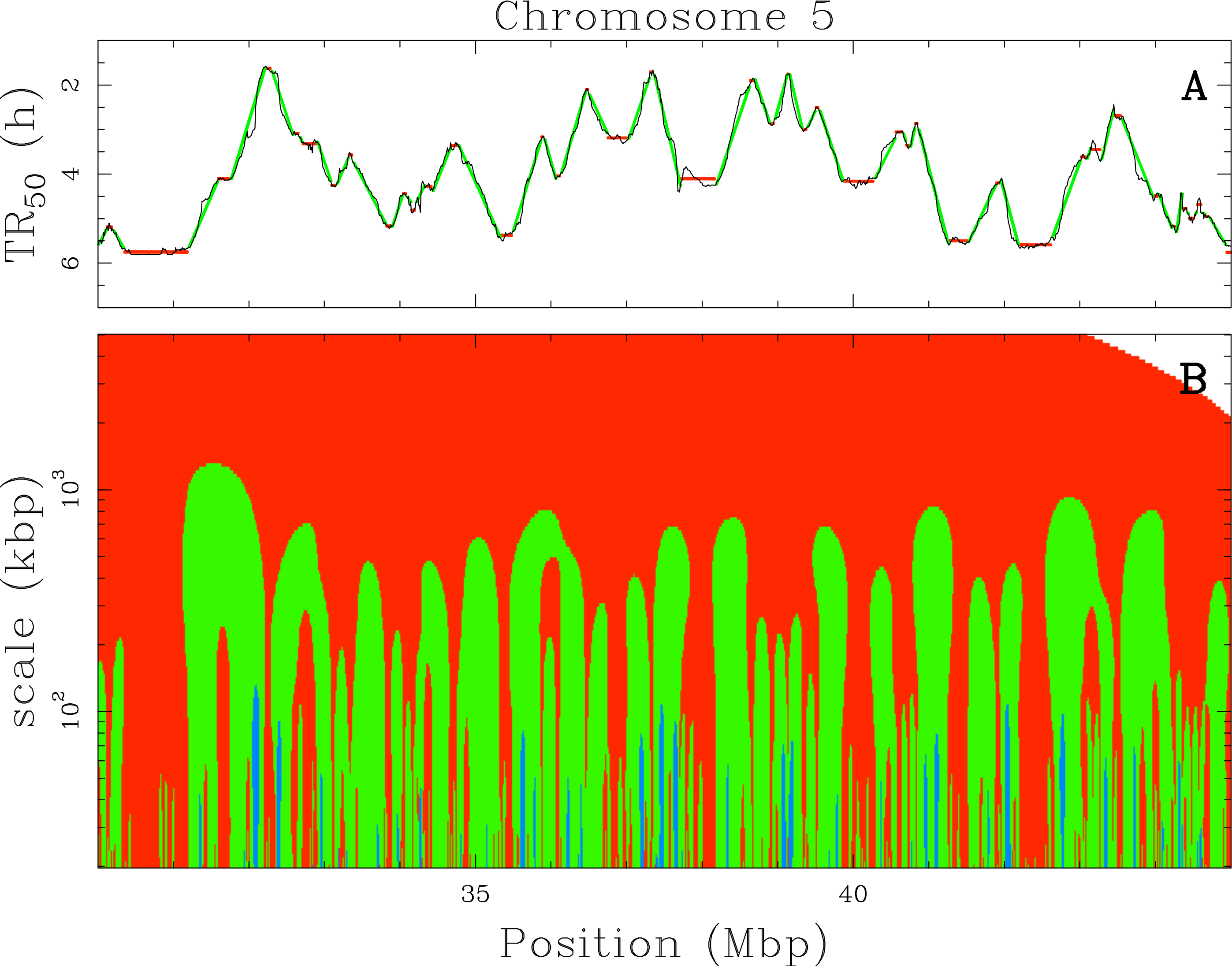

Chromosome 5
A
B
TR_{50} (h)
2
4
6
scale (kbp)
10^3
10^2
35
40
Position (Mbp)

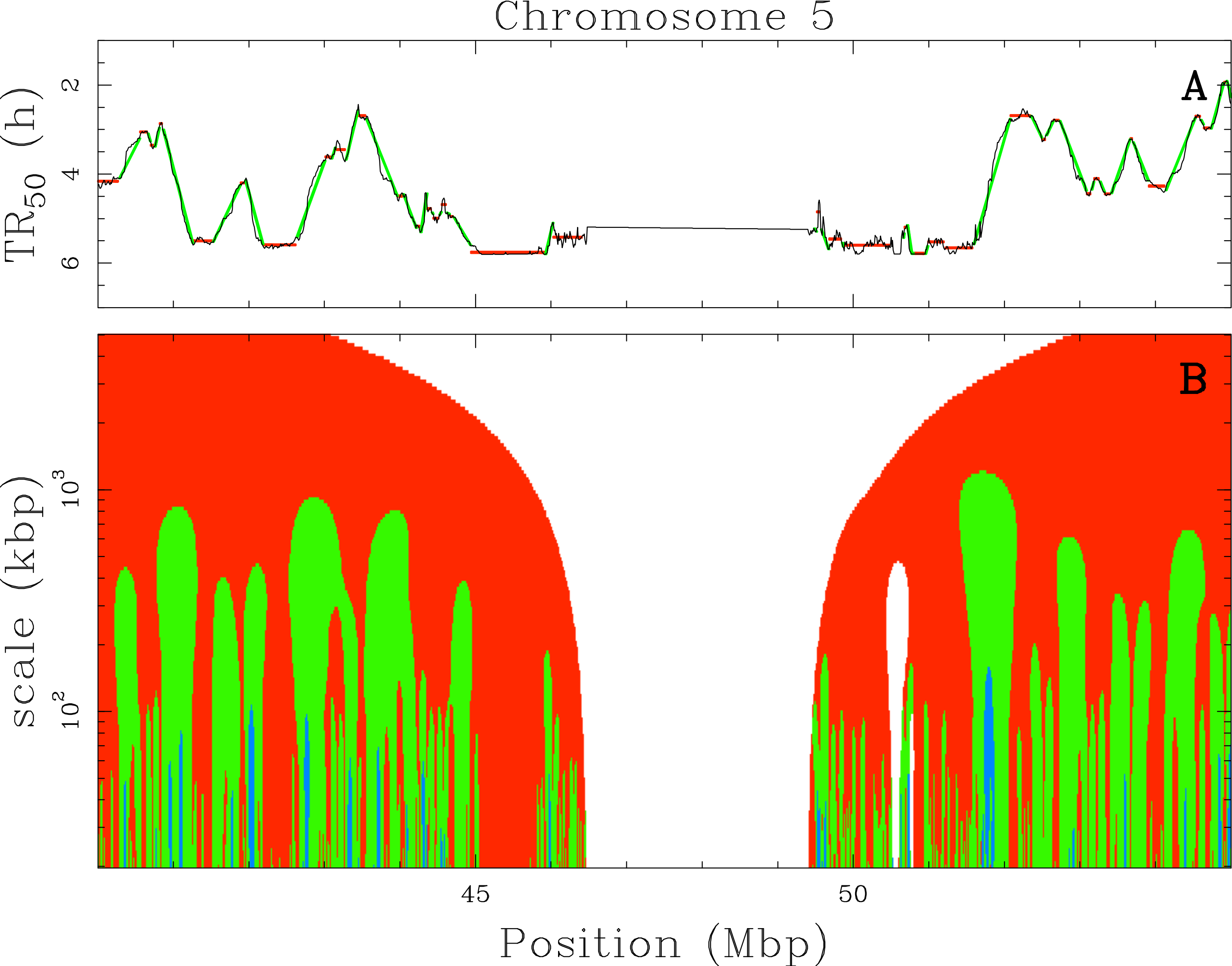

Chromosome 5
A
TR50 (h)
2
4
6
B
scale (kbp)
10^3
10^2
45
50
Position (Mbp)

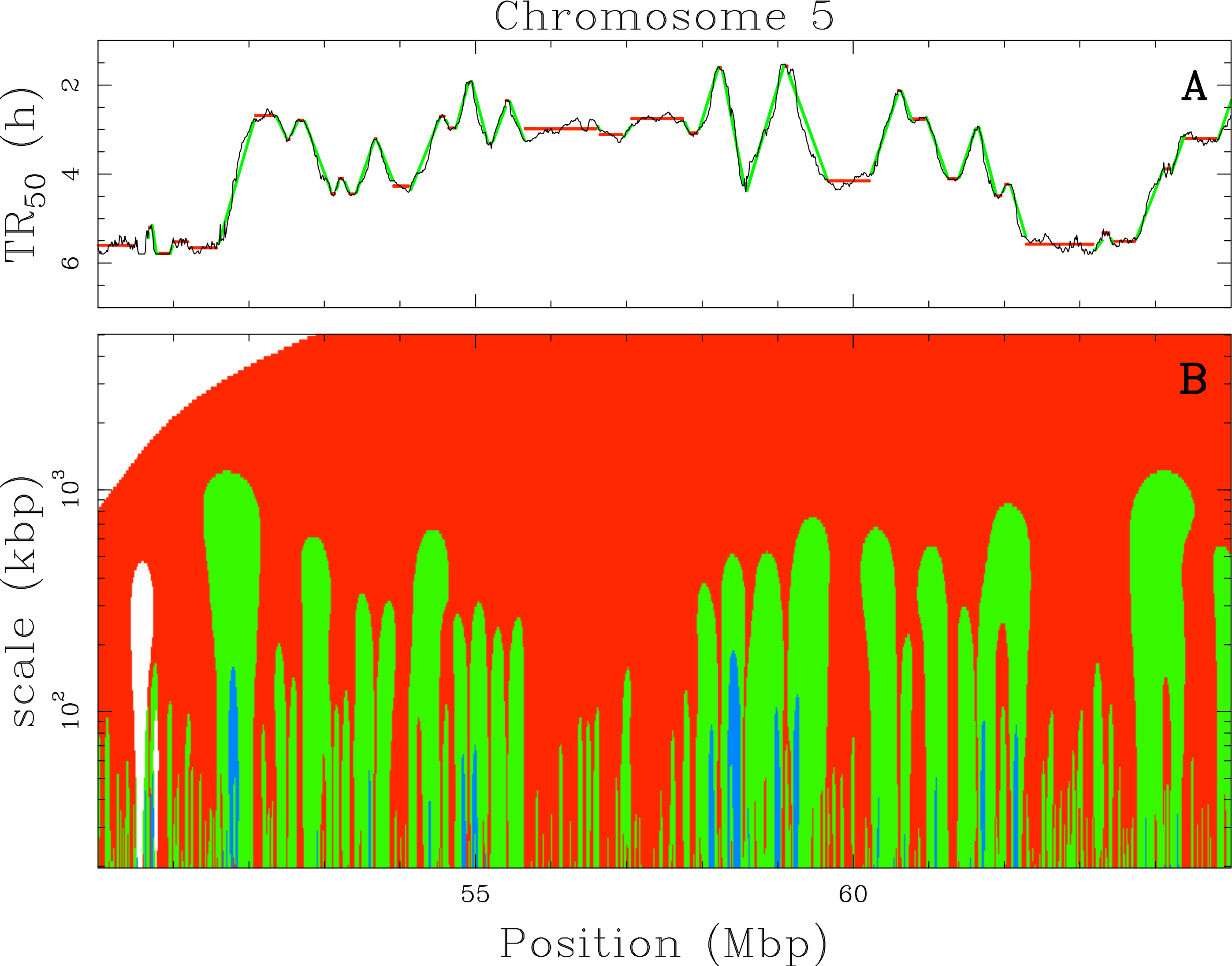

Chromosome 5
A
B
TR50 (h)
2
4
6
scale (kbp)
10^3
10^2
55
60
Position (Mbp)

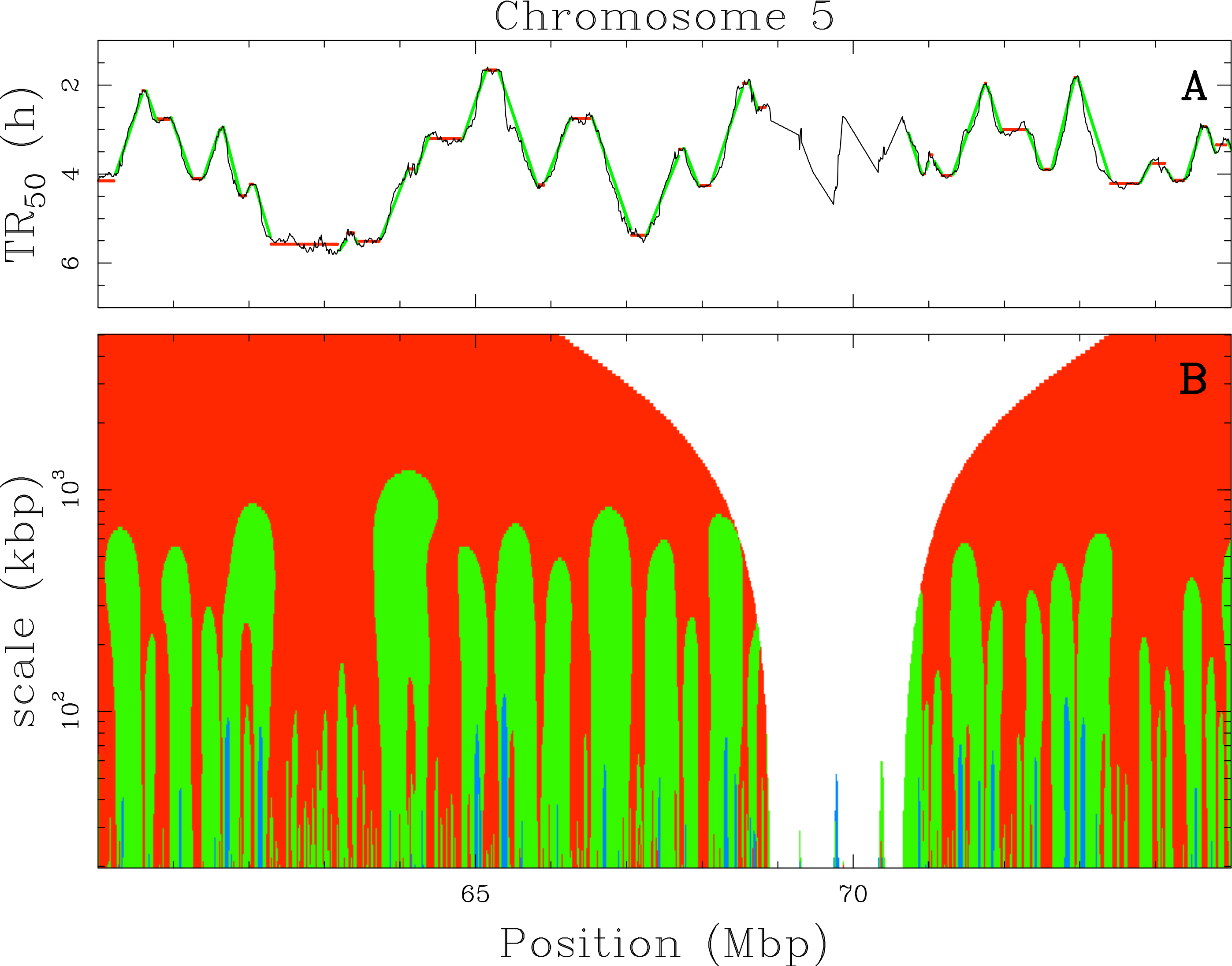
Chromosome 5
A
B
TR50 (h)
2
4
6
scale (kbp)
10^3
10^2
65
70
Position (Mbp)

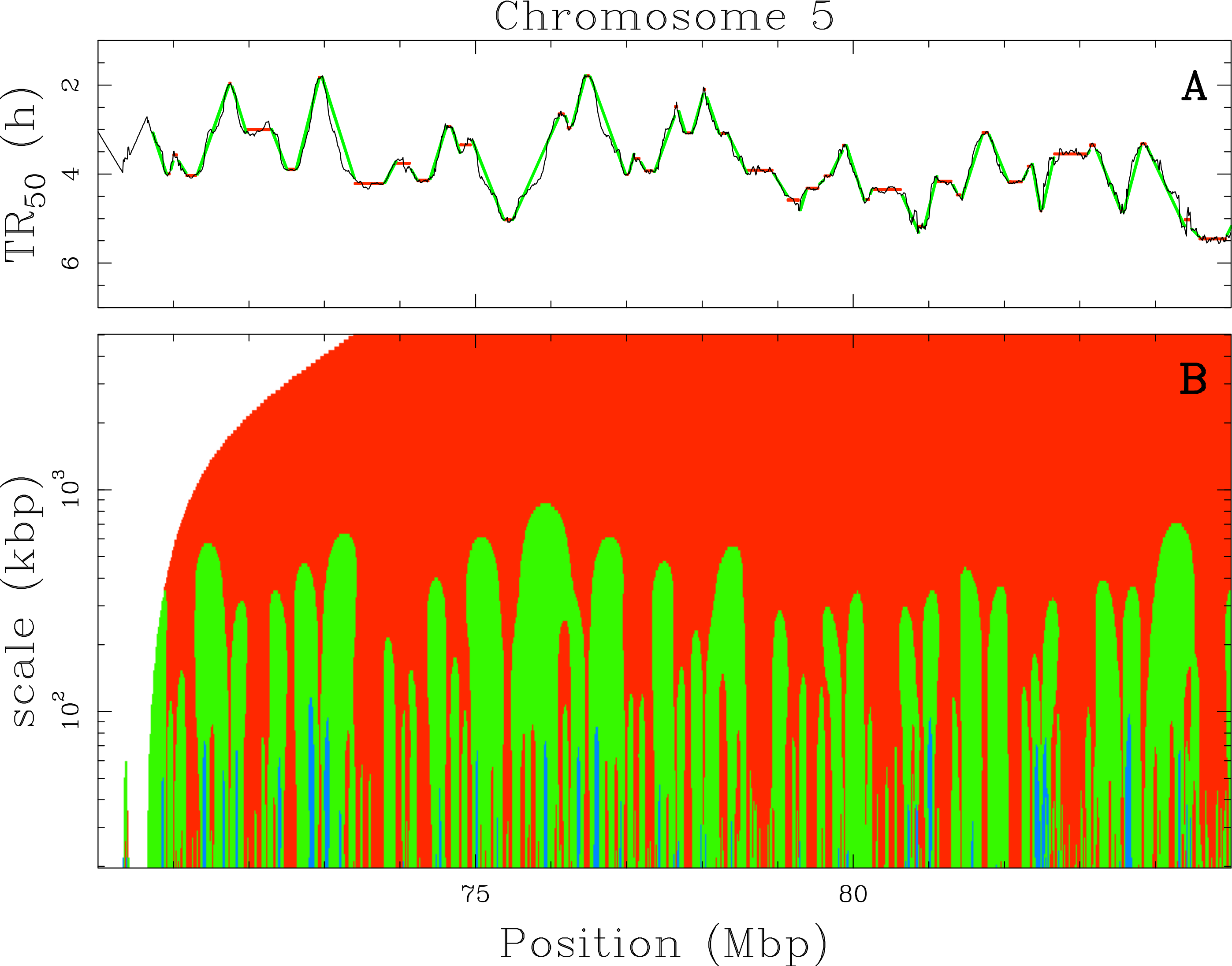

Chromosome 5
A
TR$_{50}$ (h)
2
4
6
B
scale (kbp)
10^3
10^2
75
80
Position (Mbp)

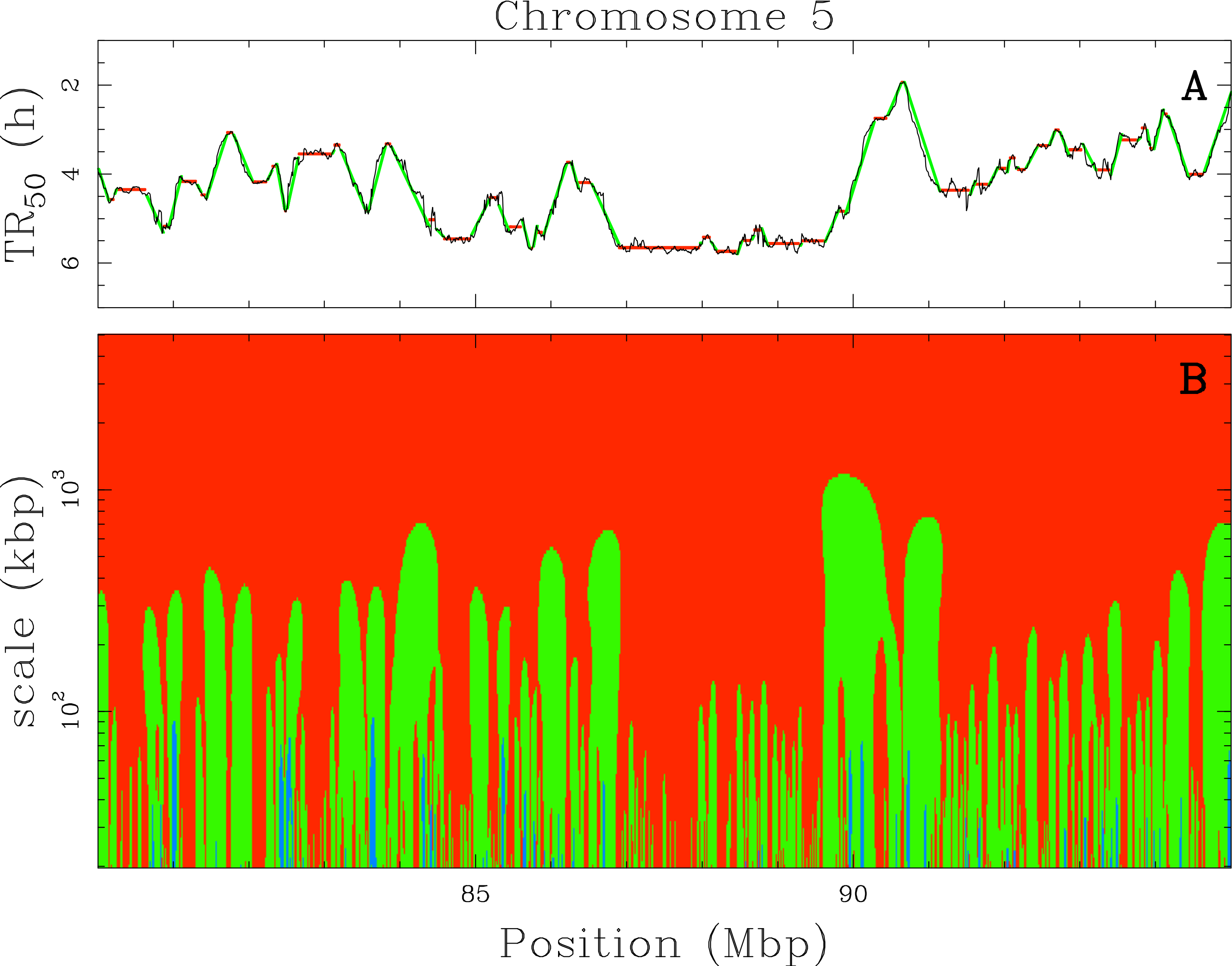

Chromosome 5
A
B
TR50 (h)
2
4
6
scale (kbp)
10^3
10^2
85
90
Position (Mbp)

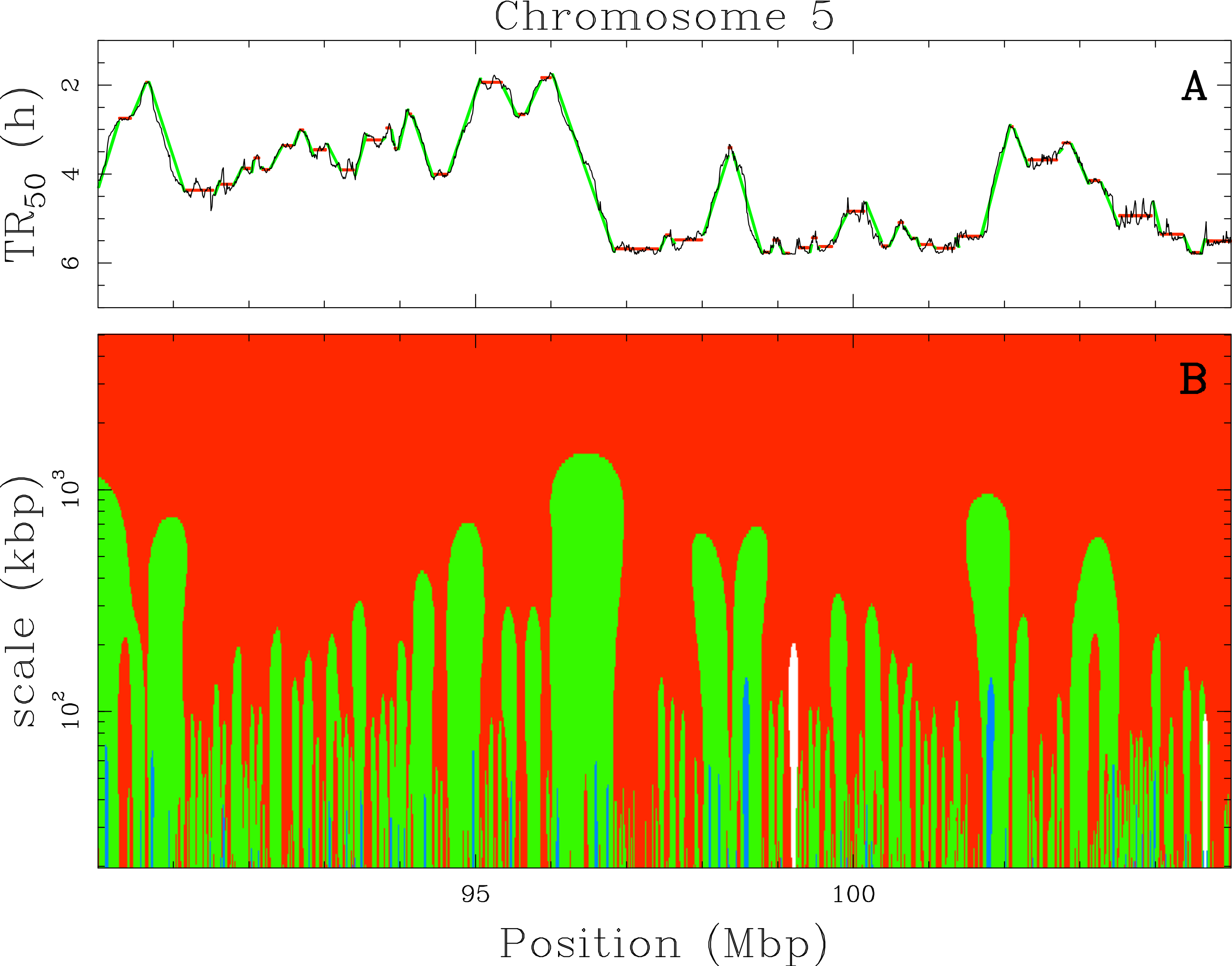

Chromosome 5
A
TR50 (h)
2
4
6
B
scale (kbp)
10^3
10^2
95
100
Position (Mbp)

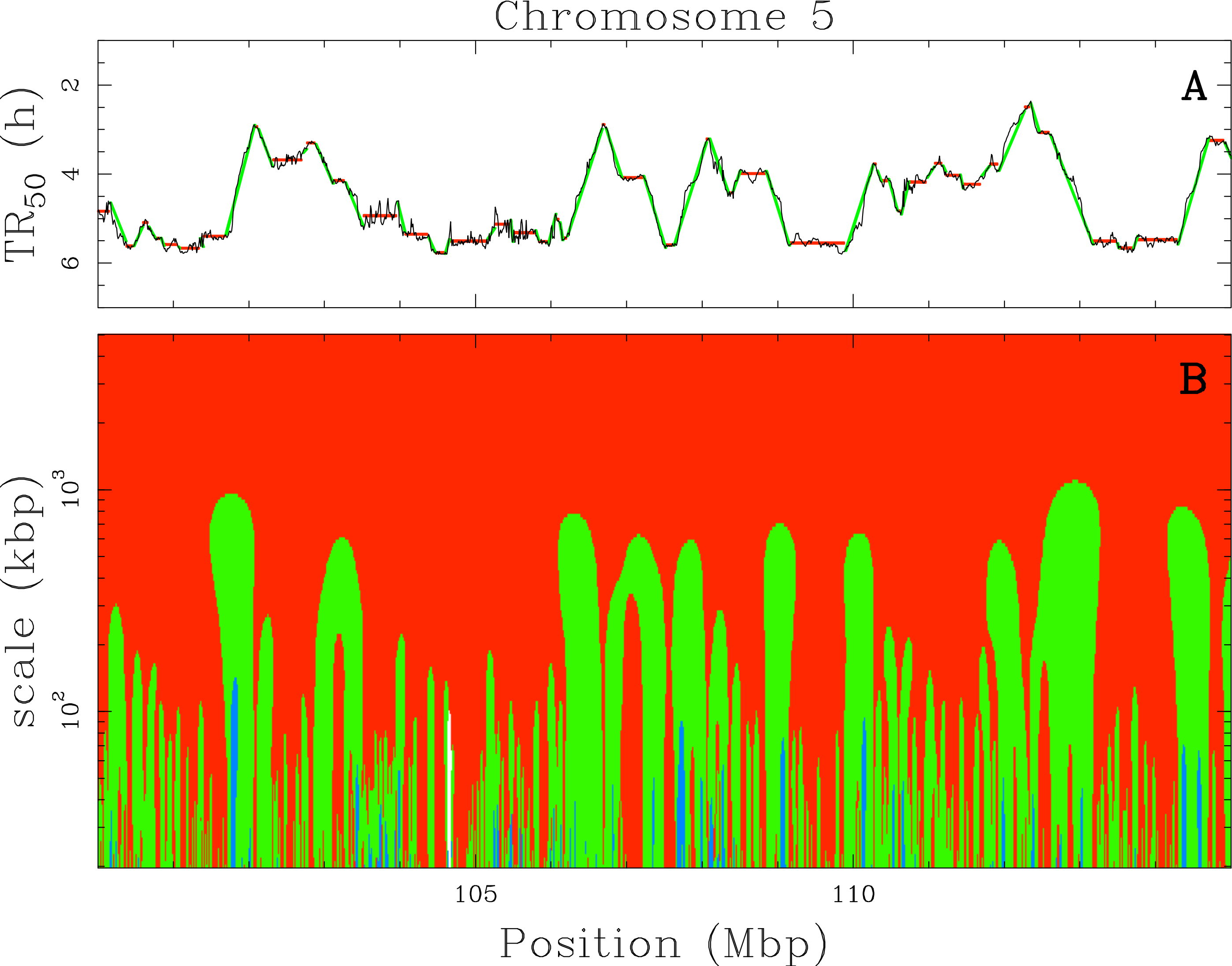
Chromosome 5
A
TR50 (h)
2
4
6
B
scale (kbp)
10^3
10^2
105
110
Position (Mbp)

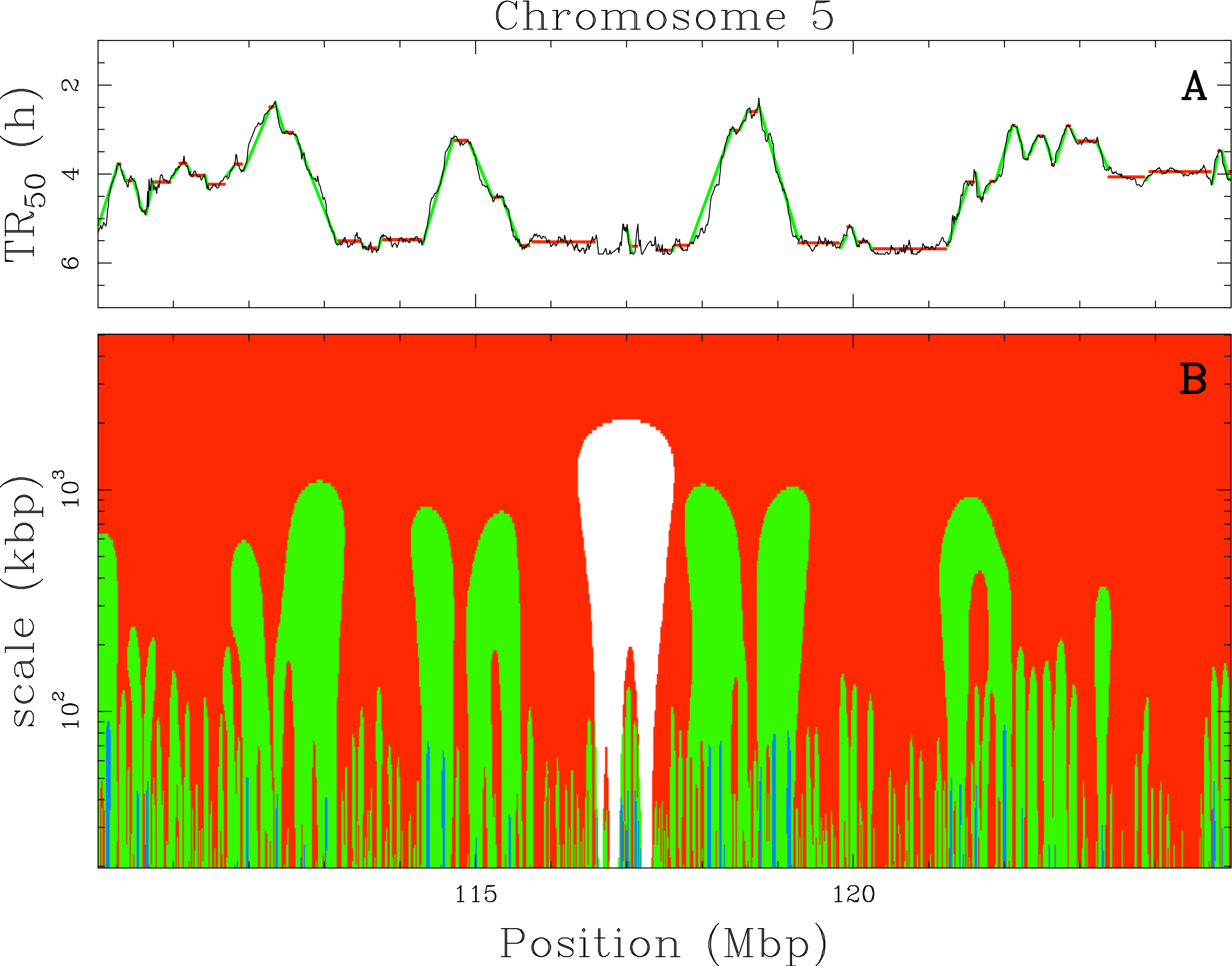

Chromosome 5
A
TR50 (h)
2
4
6
B
scale (kbp)
10^3
10^2
115
120
Position (Mbp)

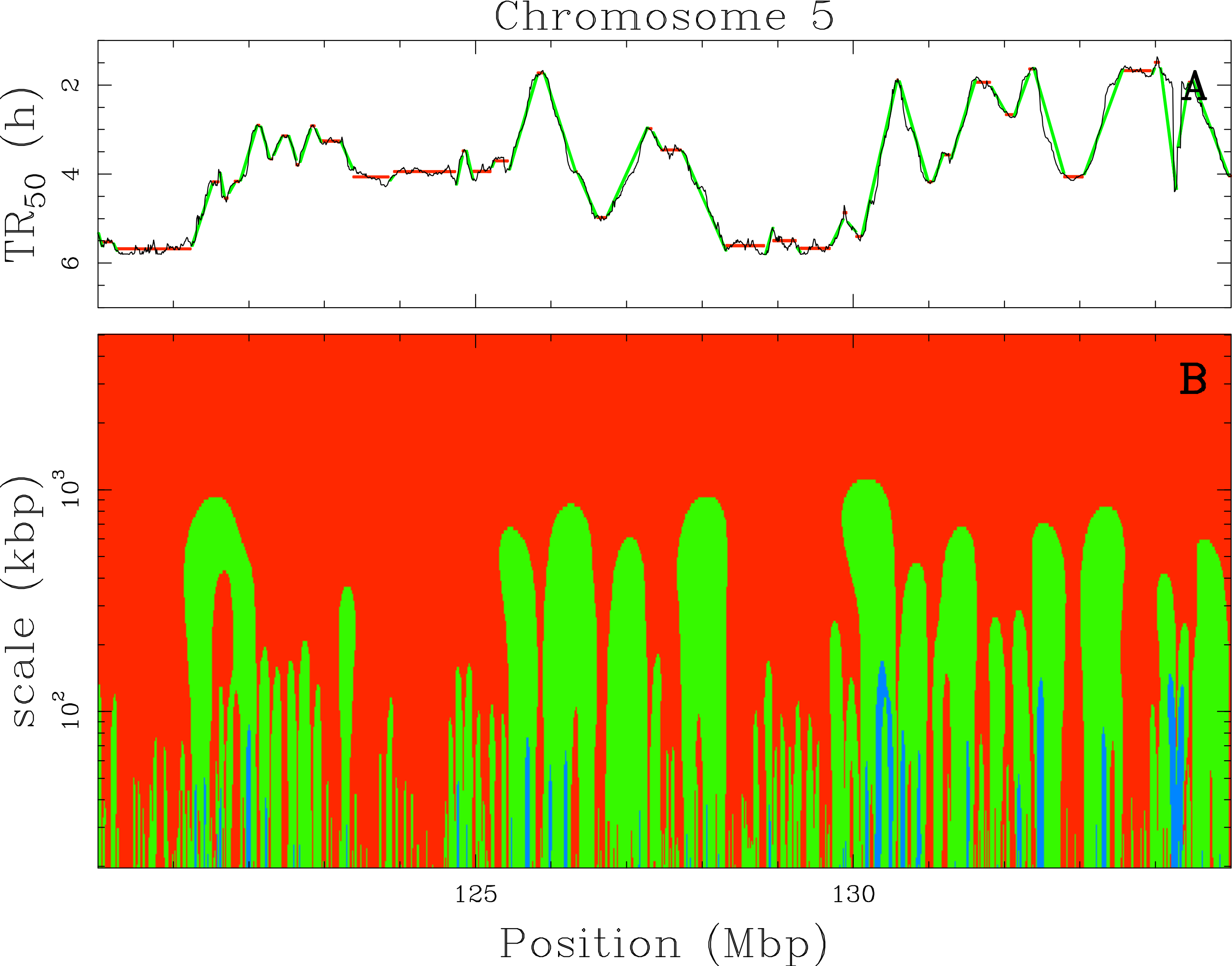

Chromosome 5
TR50 (h)
2
4
6
A
B
scale (kbp)
10^3
10^2
125
130
Position (Mbp)

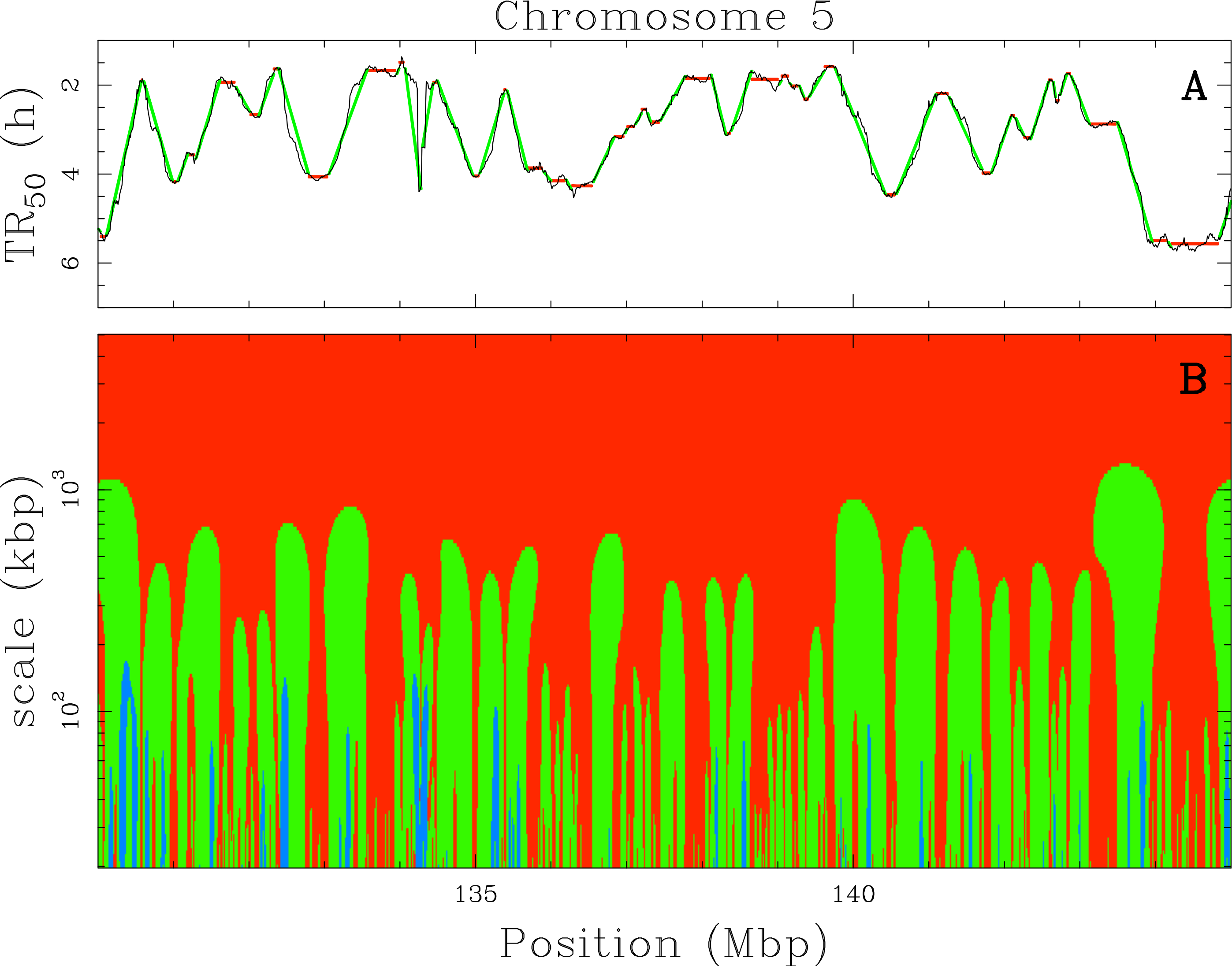

Chromosome 5
A
TR50 (h)
2
4
6
B
scale (kbp)
10^3
10^2
135
140
Position (Mbp)

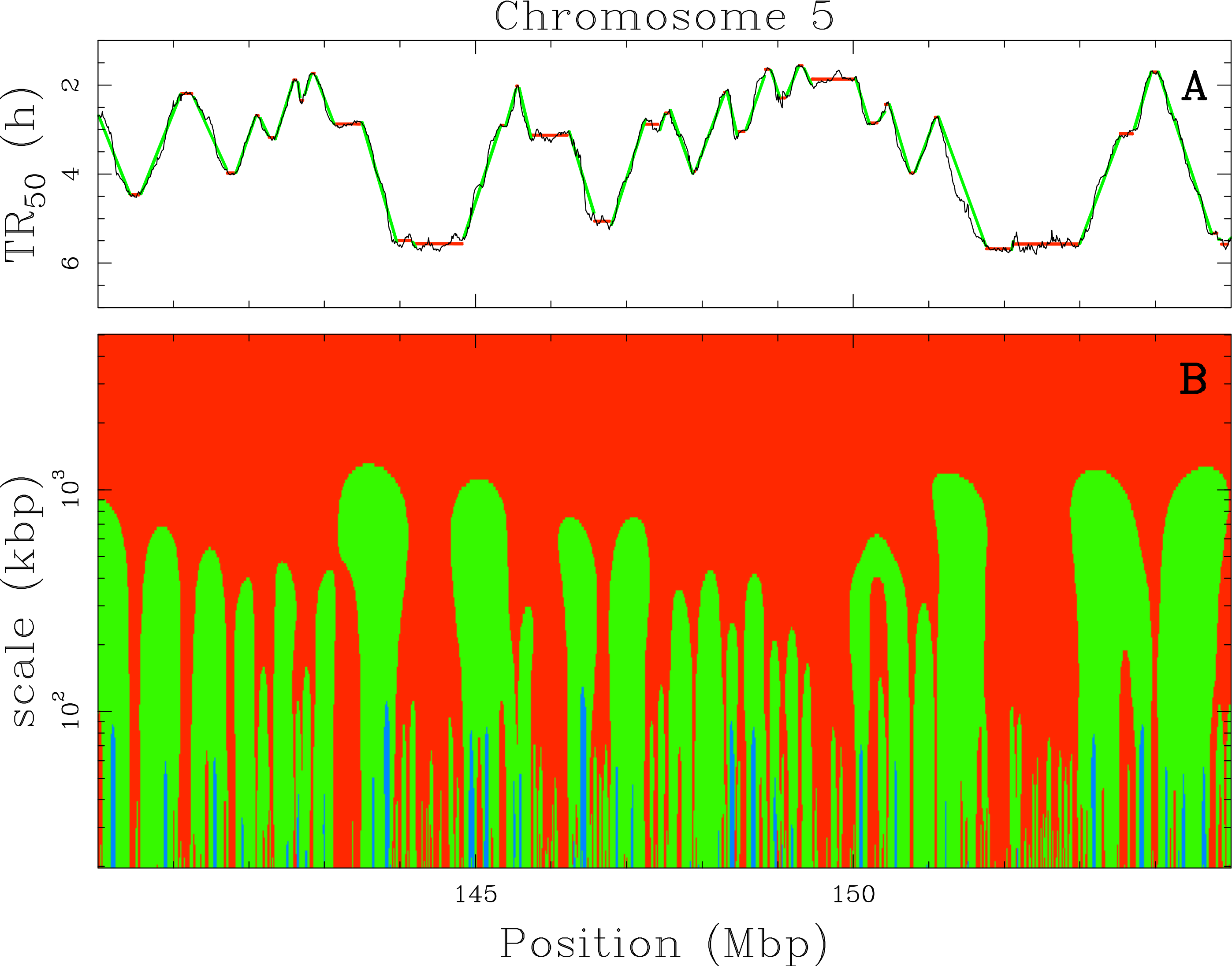

Chromosome 5
A
B
TR50 (h)
2
4
6
scale (kbp)
10^3
10^2
145
150
Position (Mbp)

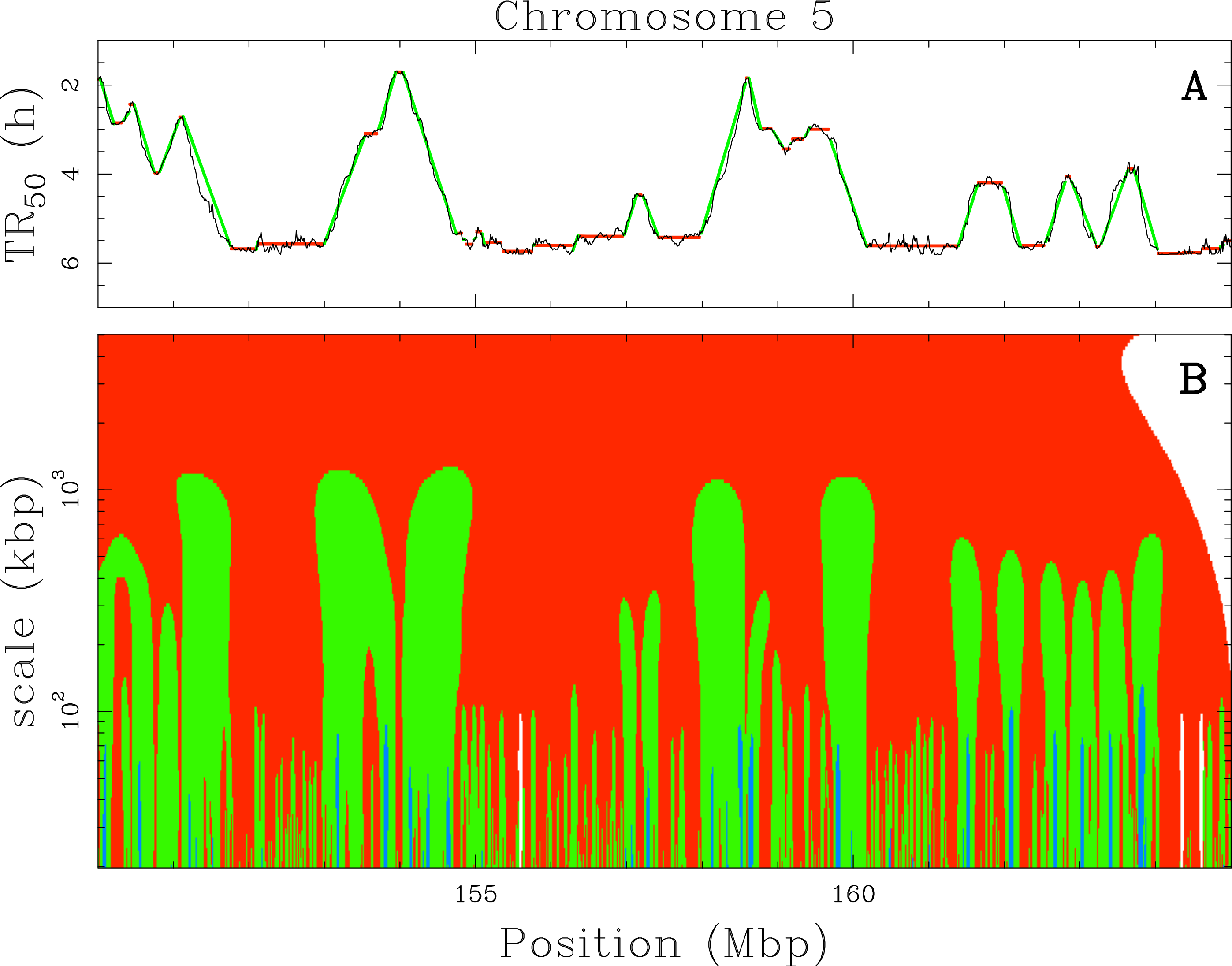
Chromosome 5
A
B
TR_{50} (h)
2
4
6
scale (kbp)
10^3
10^2
155
160
Position (Mbp)

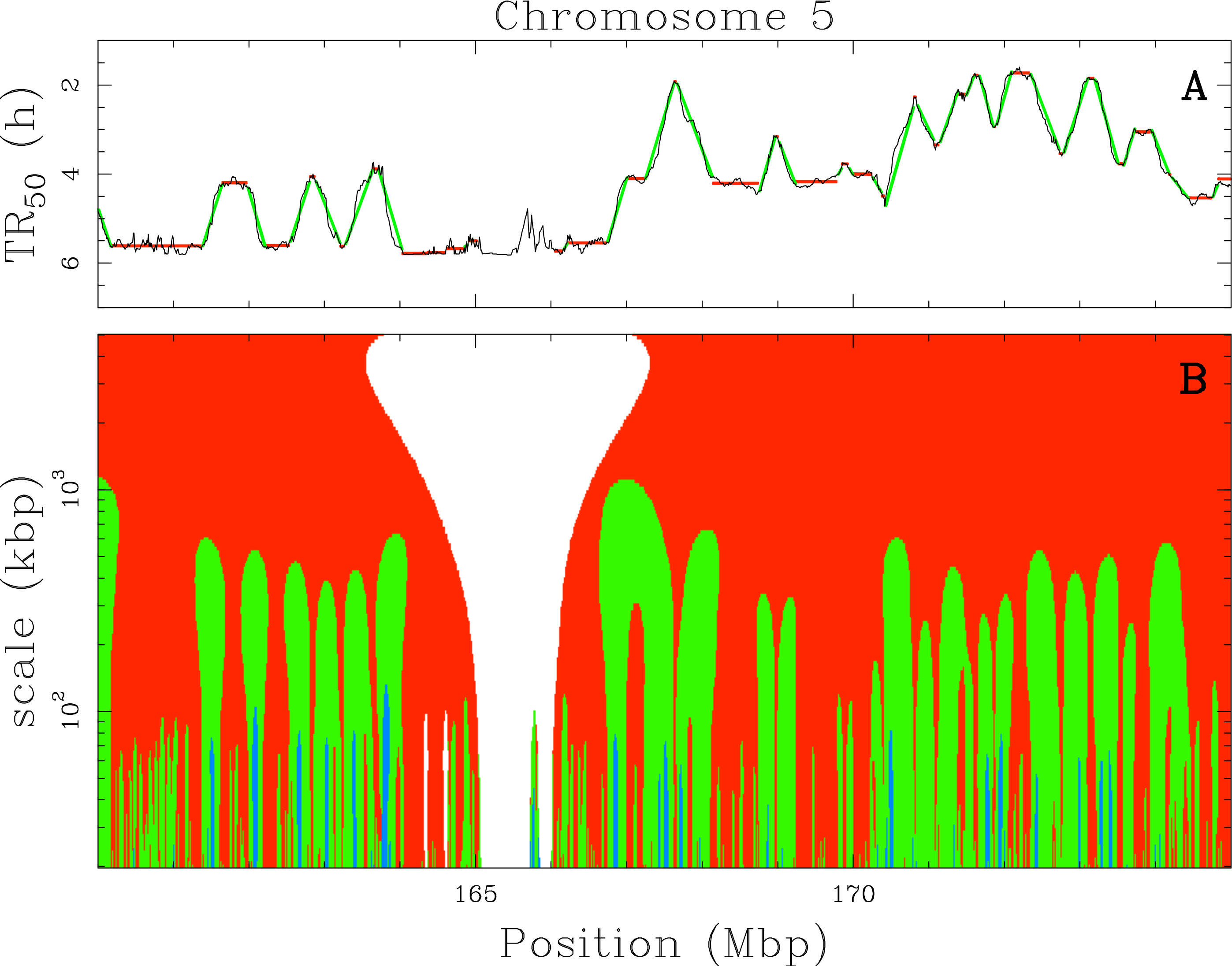

Chromosome 5
A
B
TR50 (h)
2
4
6
scale (kbp)
10^3
10^2
165
170
Position (Mbp)

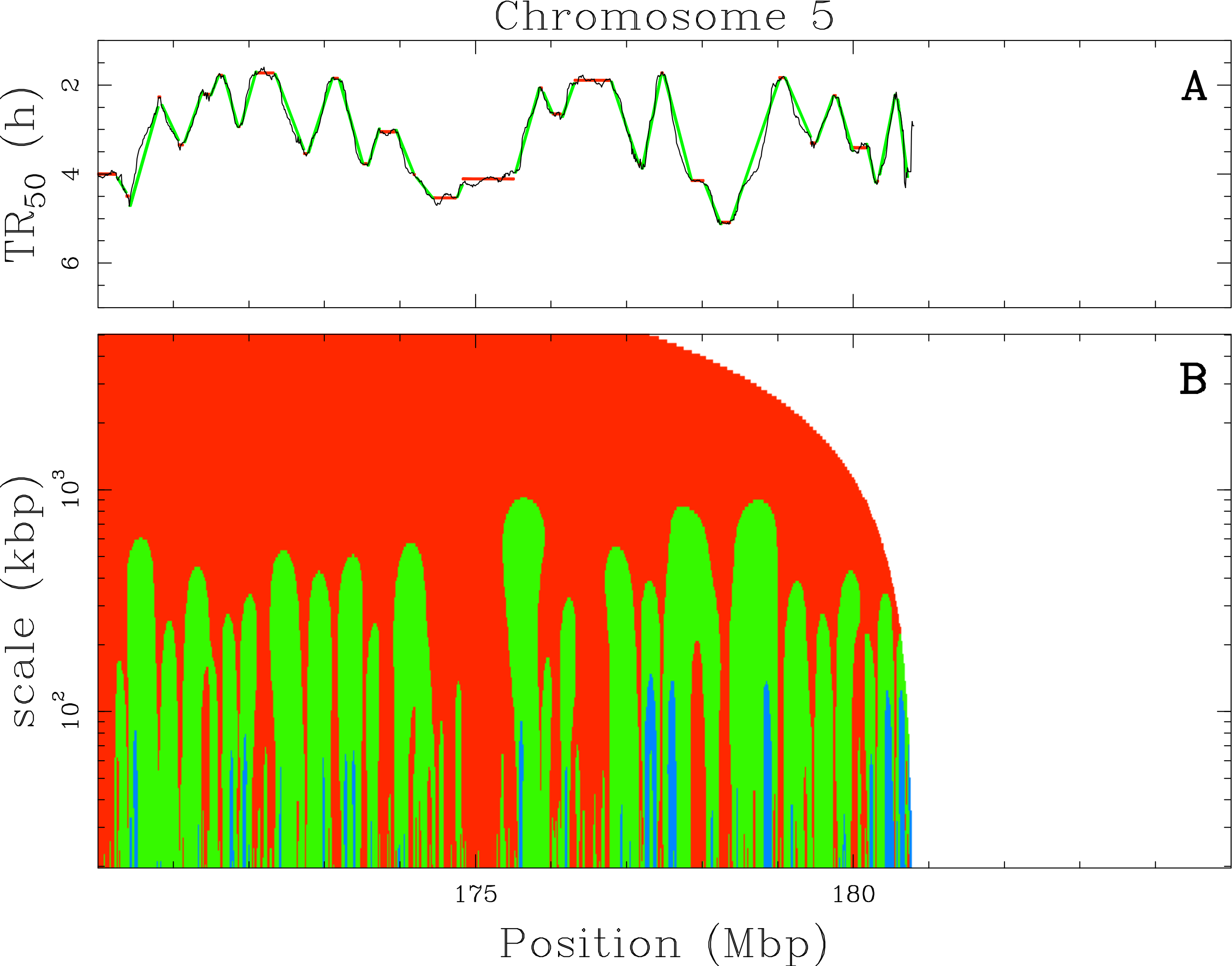

Chromosome 5
A
TR50 (h)
2
4
6
B
scale (kbp)
10^3
10^2
175
180
Position (Mbp)

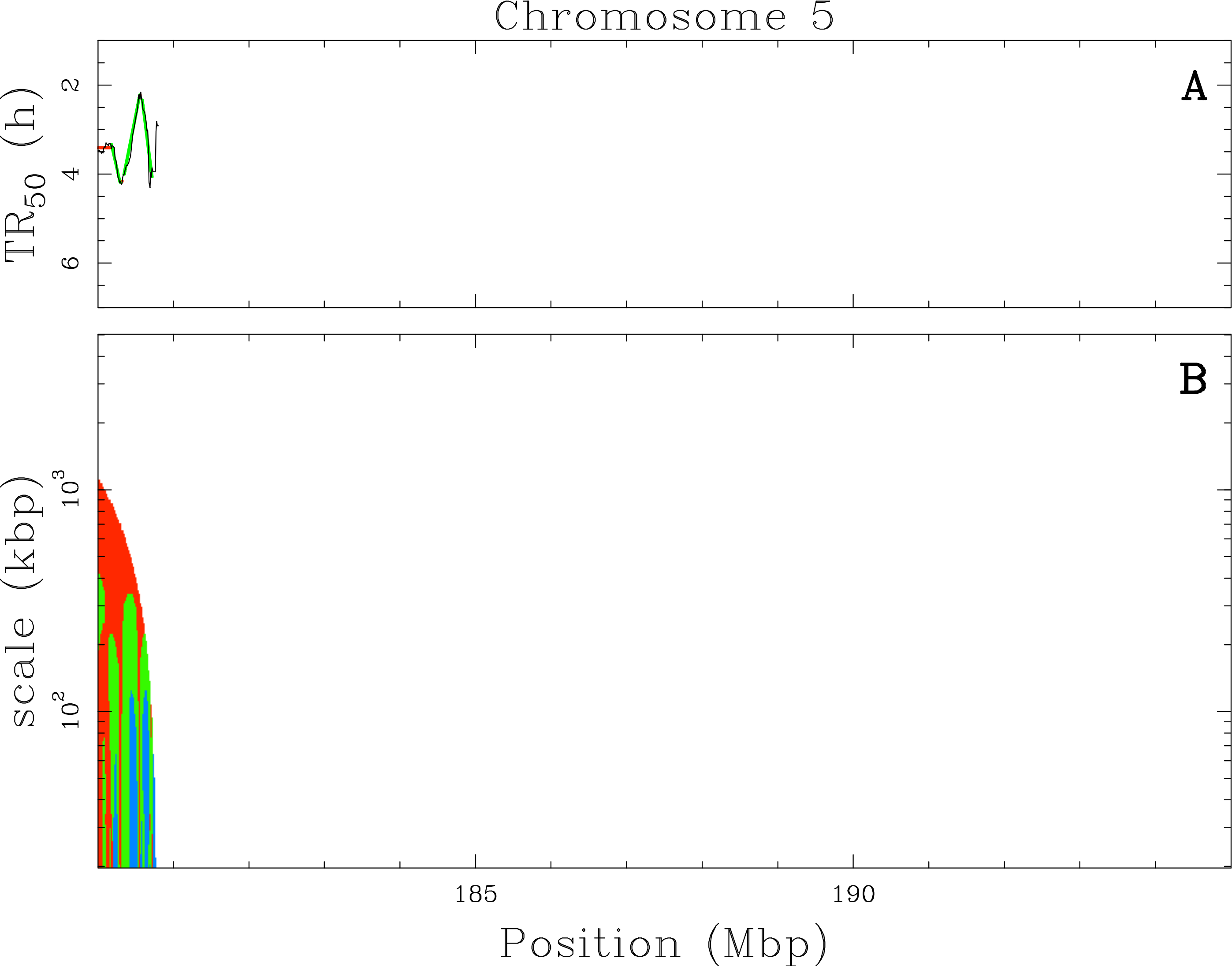

Chromosome 5
A
B
TR50 (h)
2
4
6
scale (kbp)
10^3
10^2
185
190
Position (Mbp)

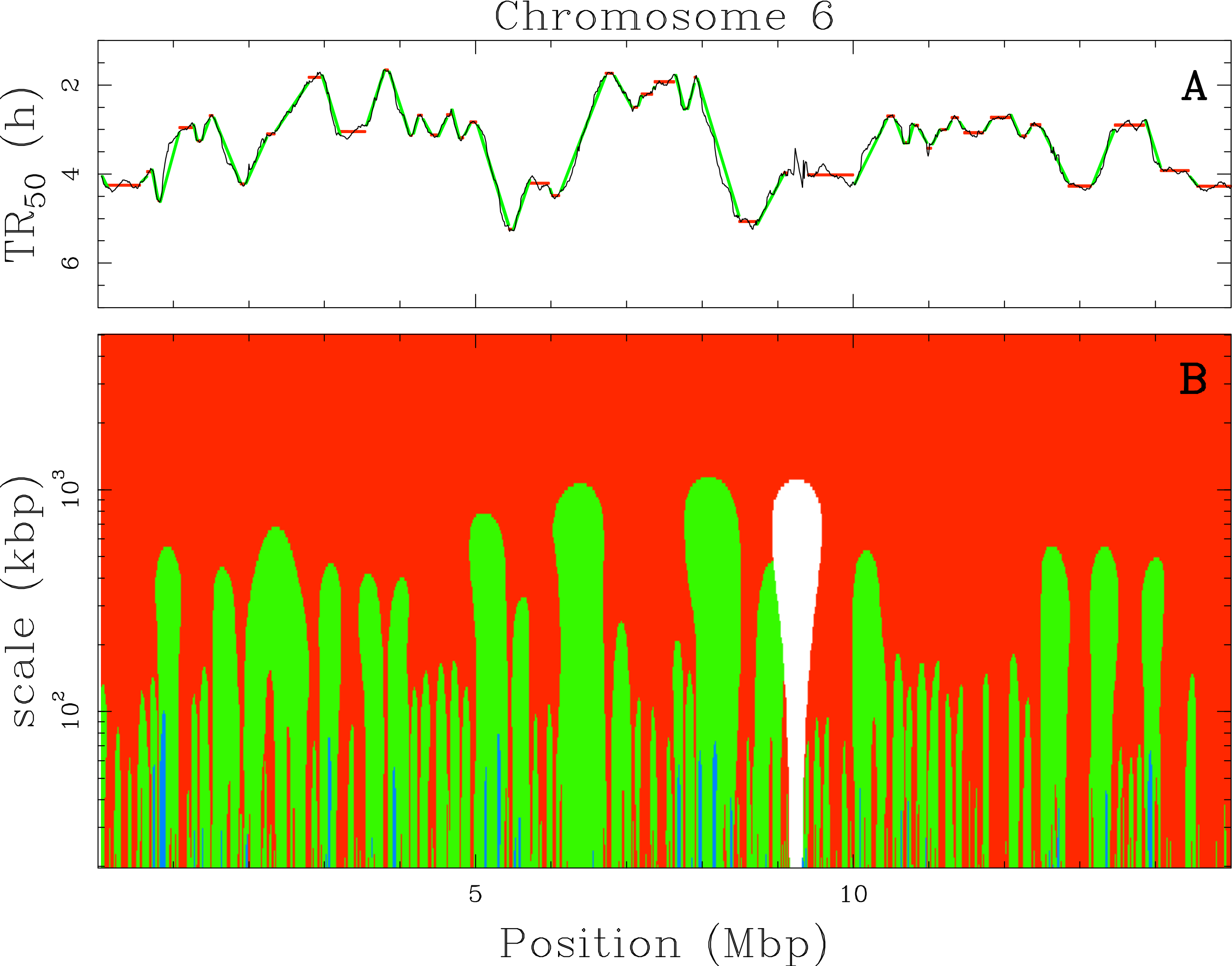

Chromosome 6
A
B
TR_{50} (h)
2
4
6
scale (kbp)
10^3
10^2
5
10
Position (Mbp)

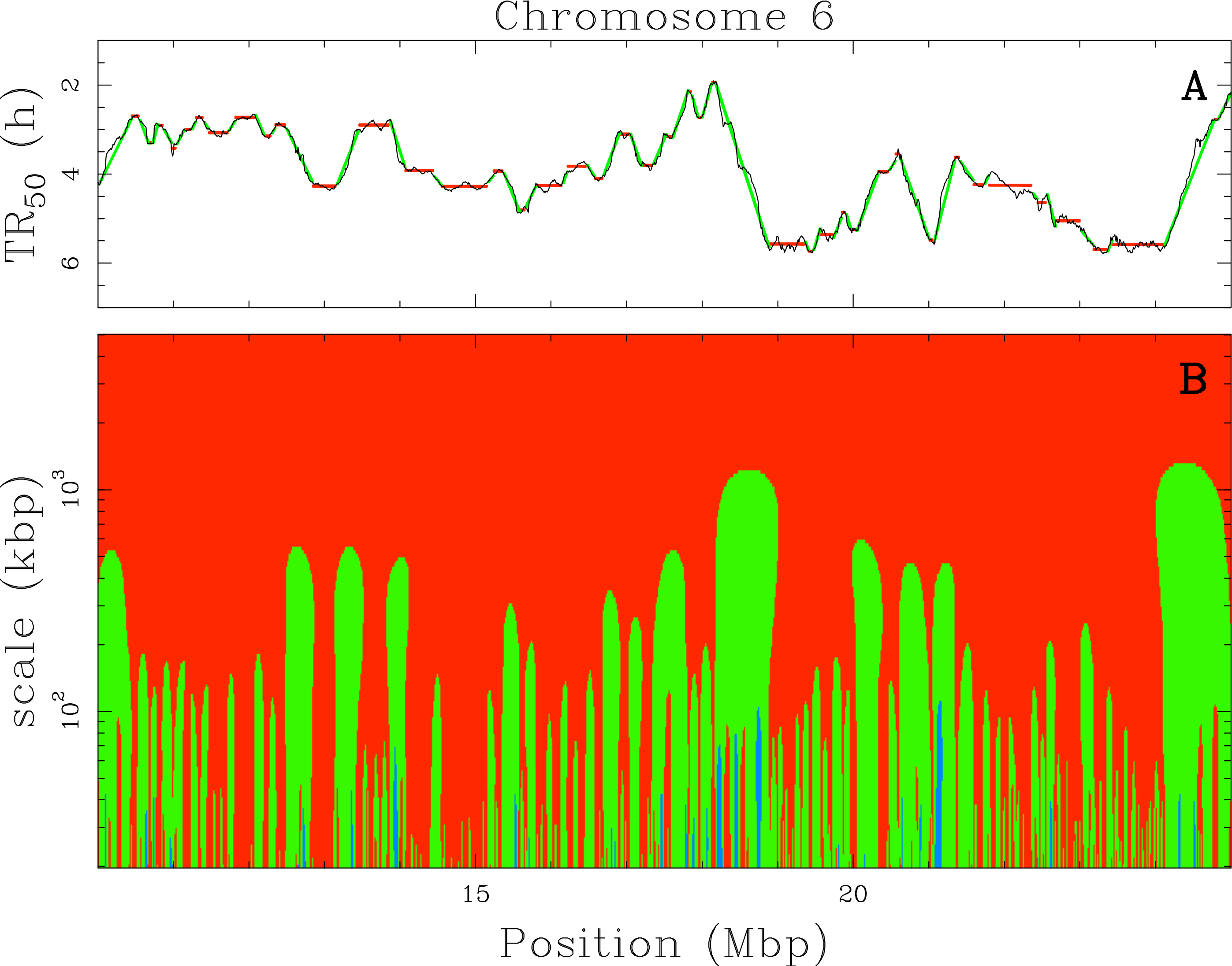

Chromosome 6
A
B
TR_{50} (h)
2
4
6
scale (kbp)
10^3
10^2
15
20
Position (Mbp)

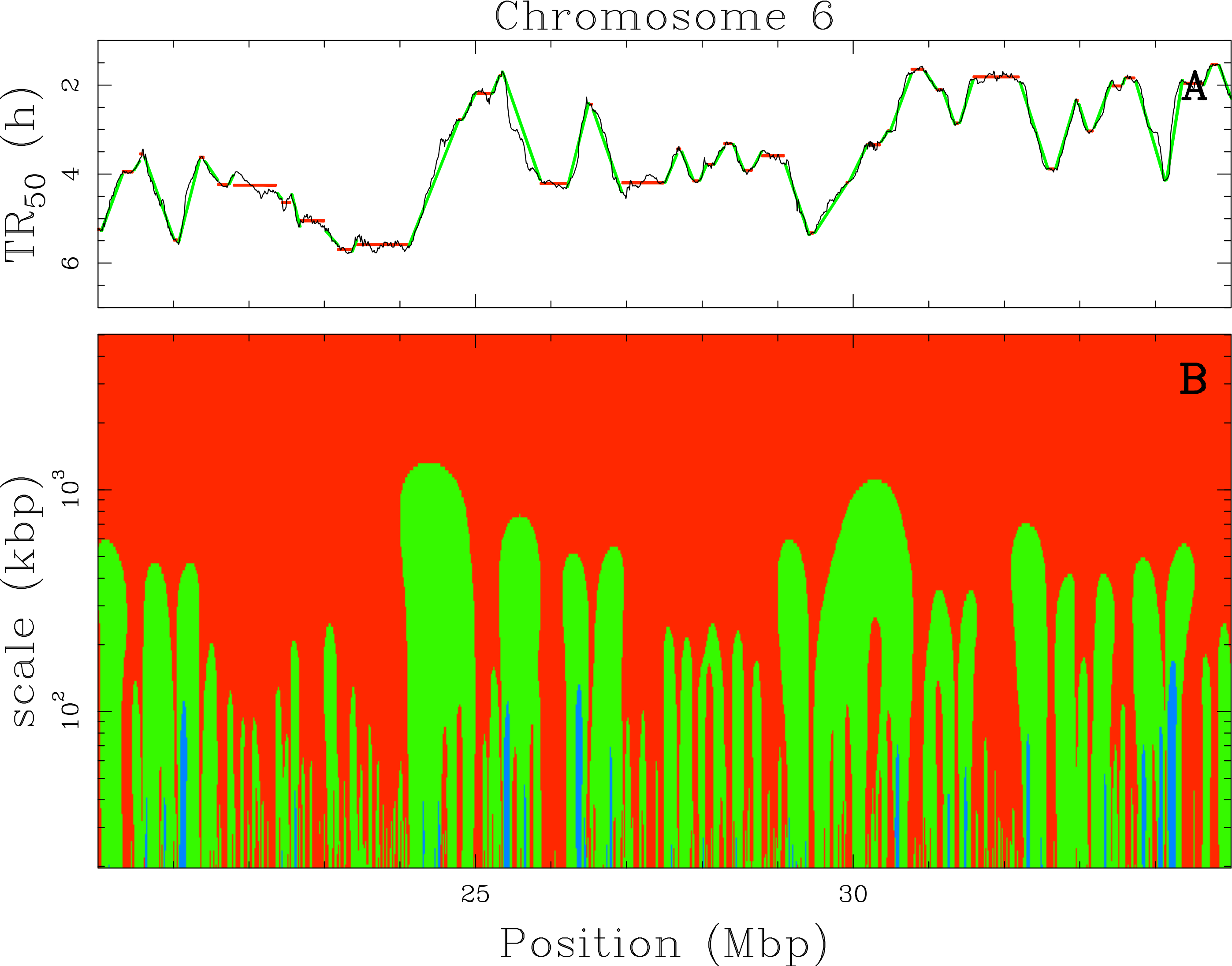

Chromosome 6
TR$_{50}$ (h)
2
4
6
A
B
scale (kbp)
10^3
10^2
25
30
Position (Mbp)

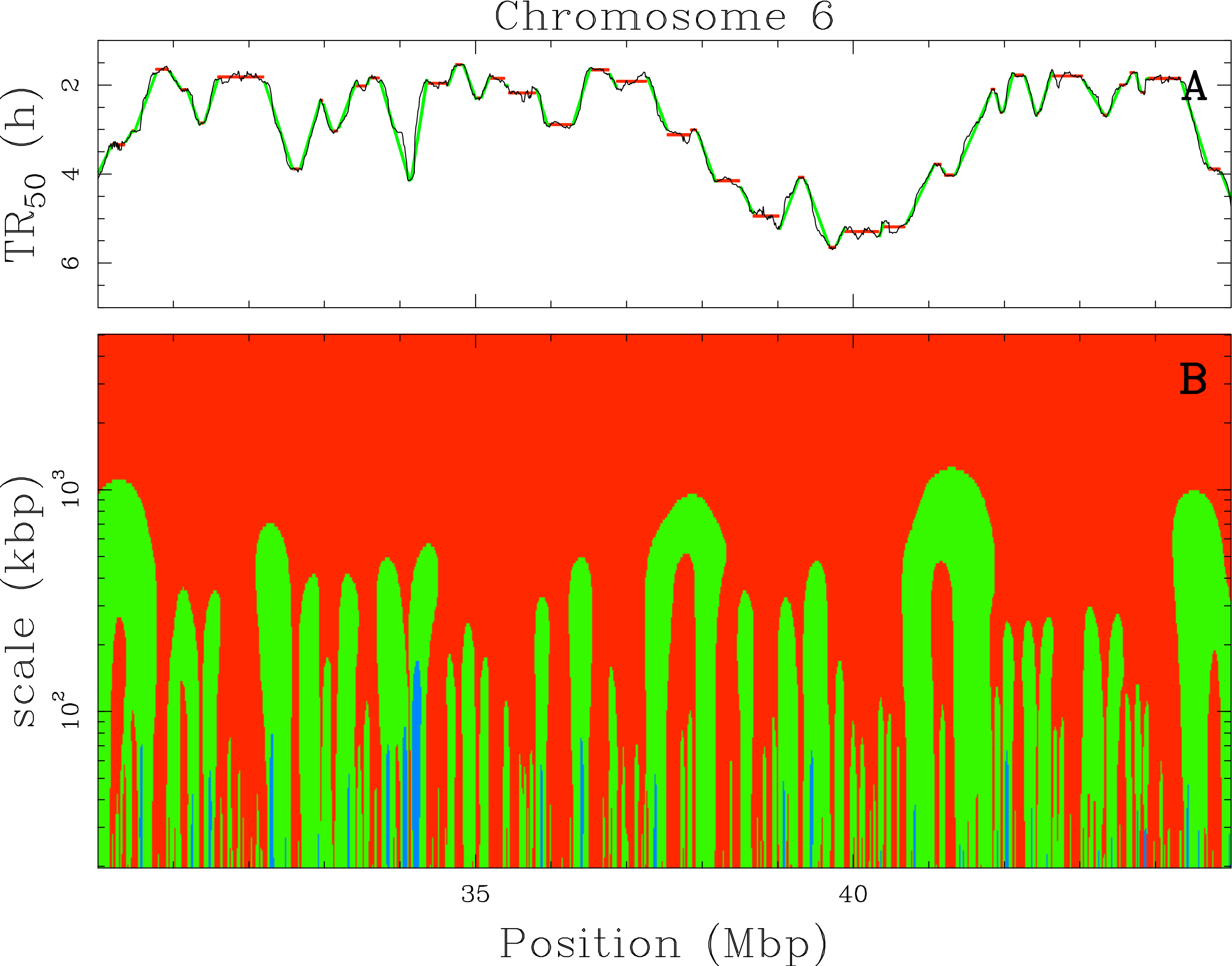

Chromosome 6
TR$_{50}$ (h)
2
4
6
A
B
scale (kbp)
10^3
10^2
35
40
Position (Mbp)

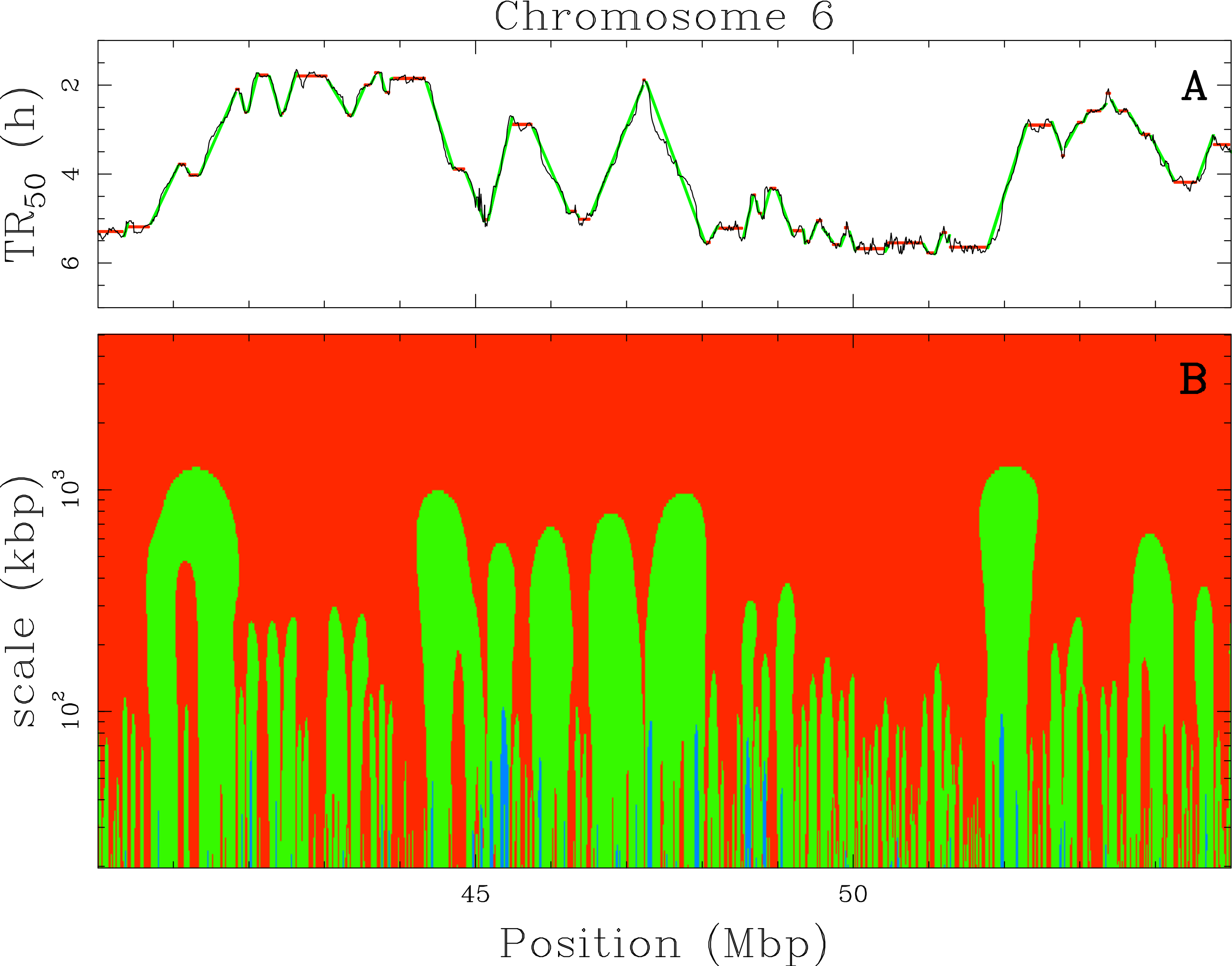

Chromosome 6
A
TR$_{50}$ (h)
2
4
6
B
scale (kbp)
10^3
10^2
45
50
Position (Mbp)

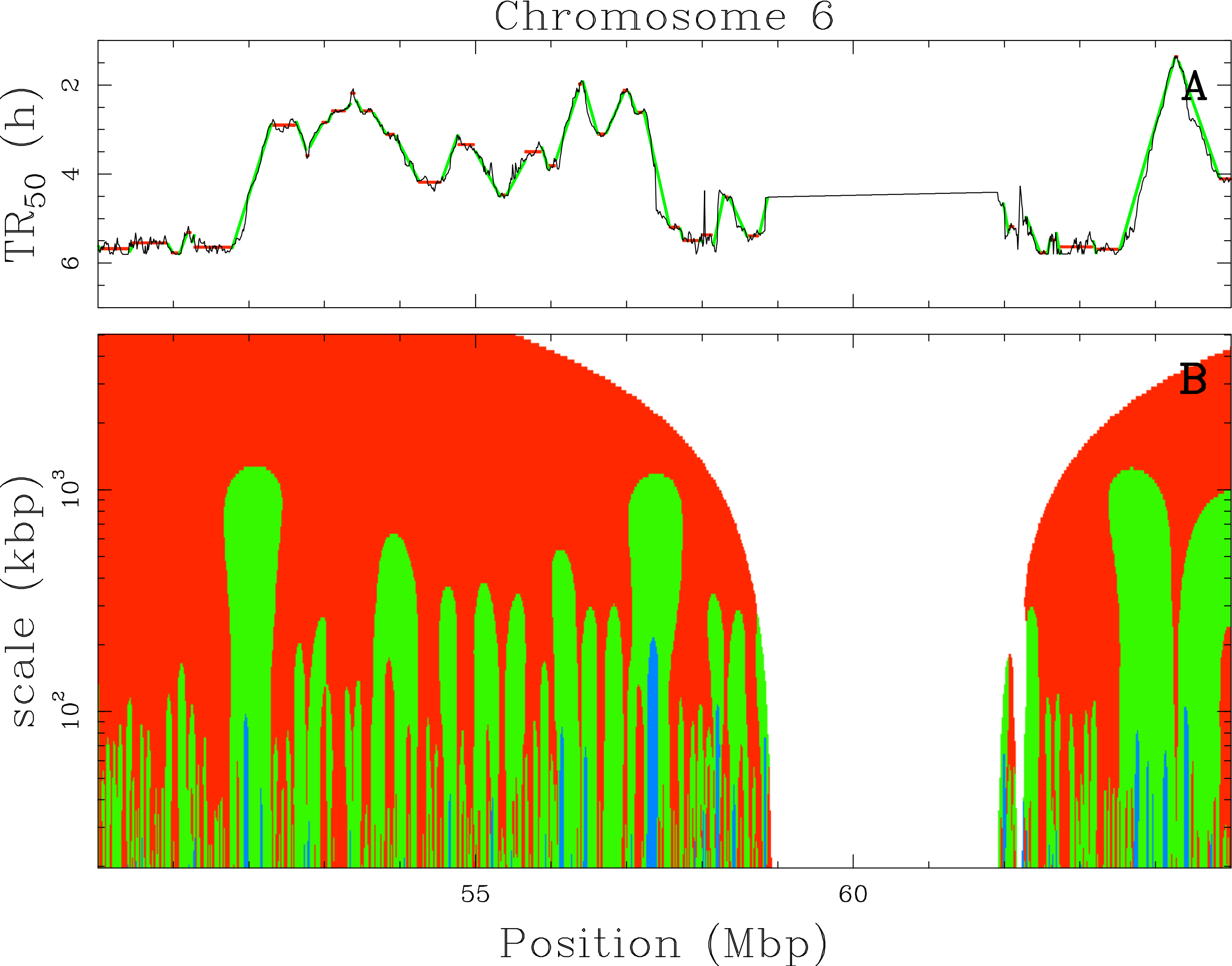

Chromosome 6
A
TR$_{50}$ (h)
2
4
6
B
scale (kbp)
10^3
10^2
55
60
Position (Mbp)

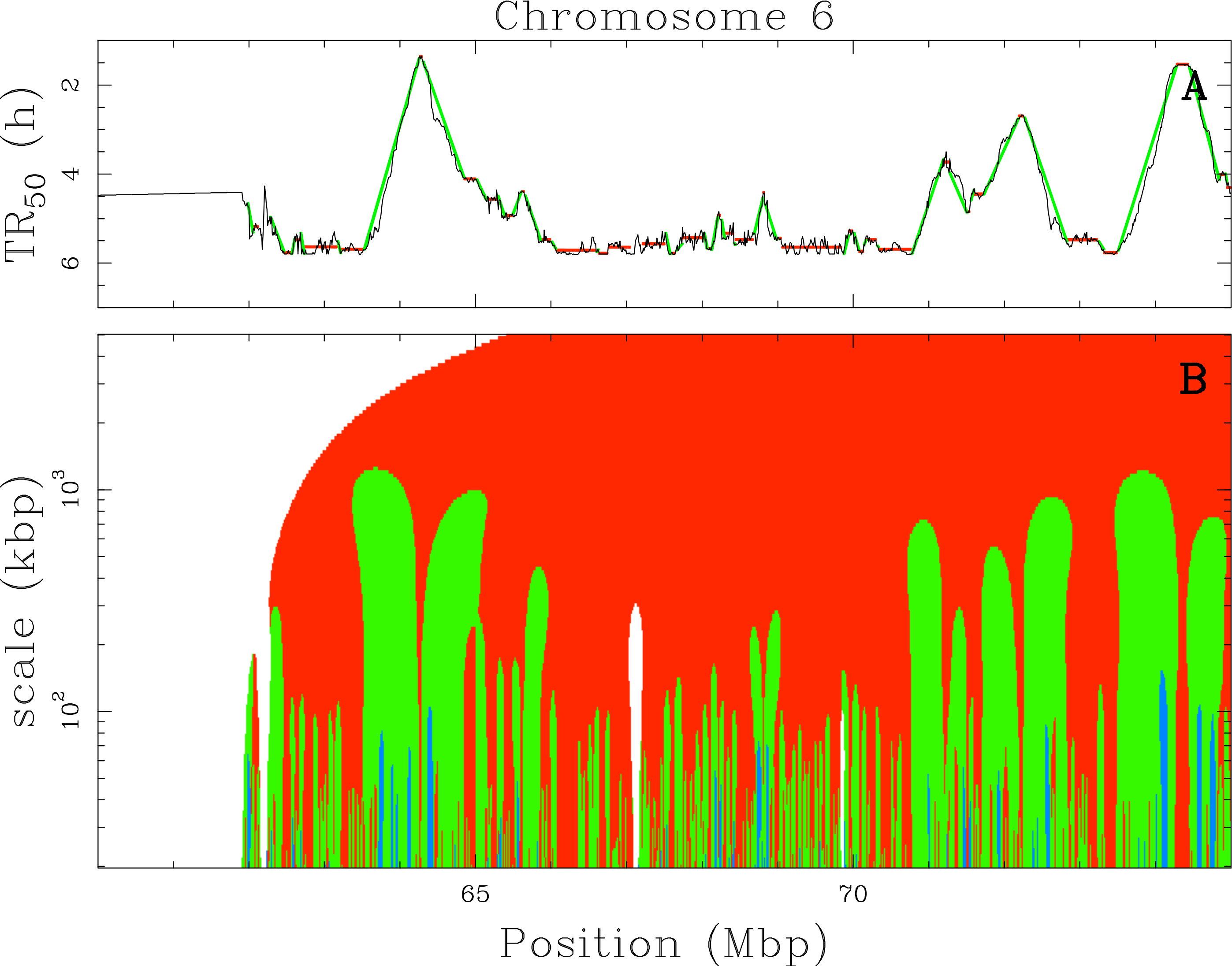

Chromosome 6
A
B
TR_{50} (h)
2
4
6
scale (kbp)
10^3
10^2
65
70
Position (Mbp)

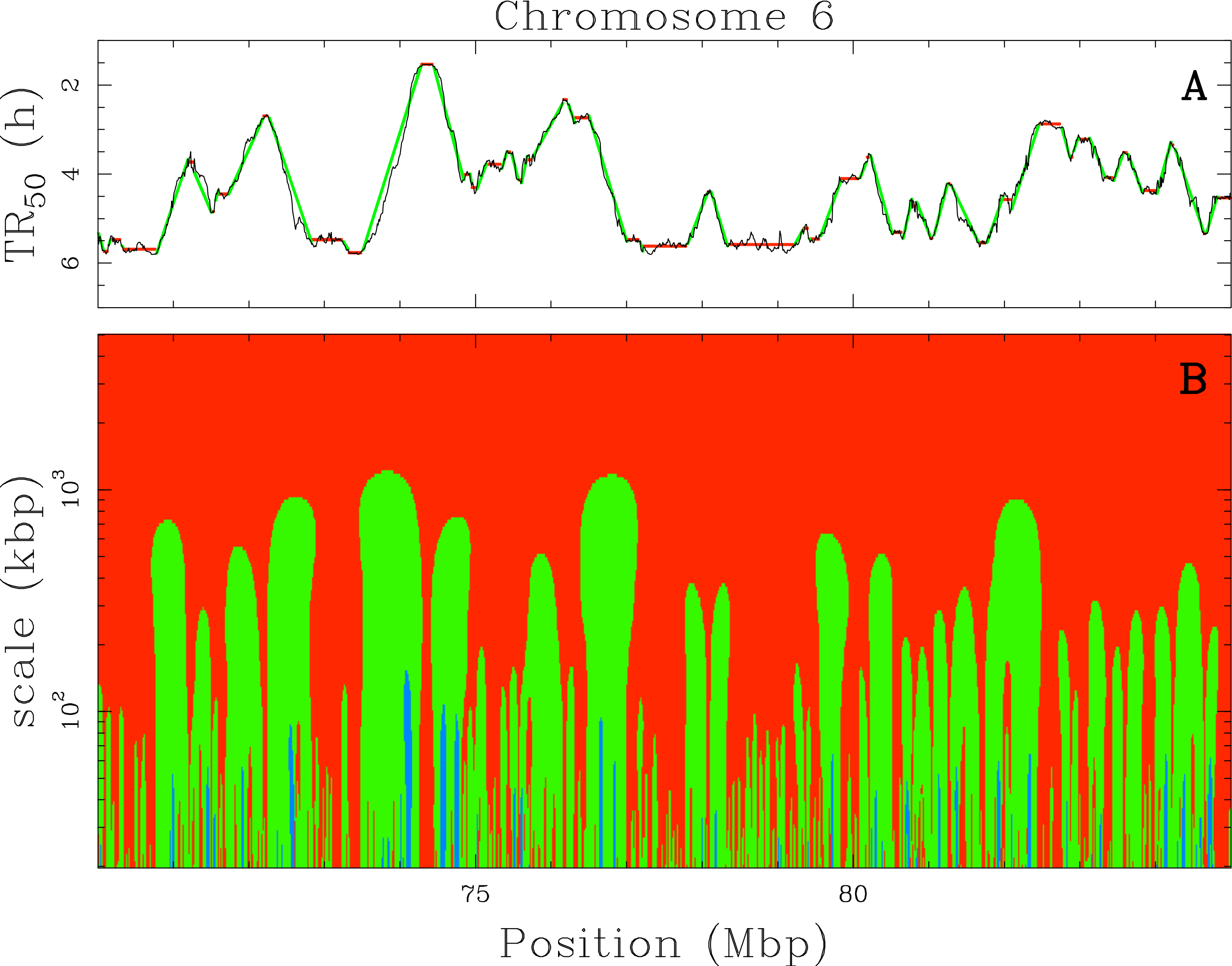

Chromosome 6
TR$_{50}$ (h)
2
4
6
A
B
scale (kbp)
10^3
10^2
75
80
Position (Mbp)

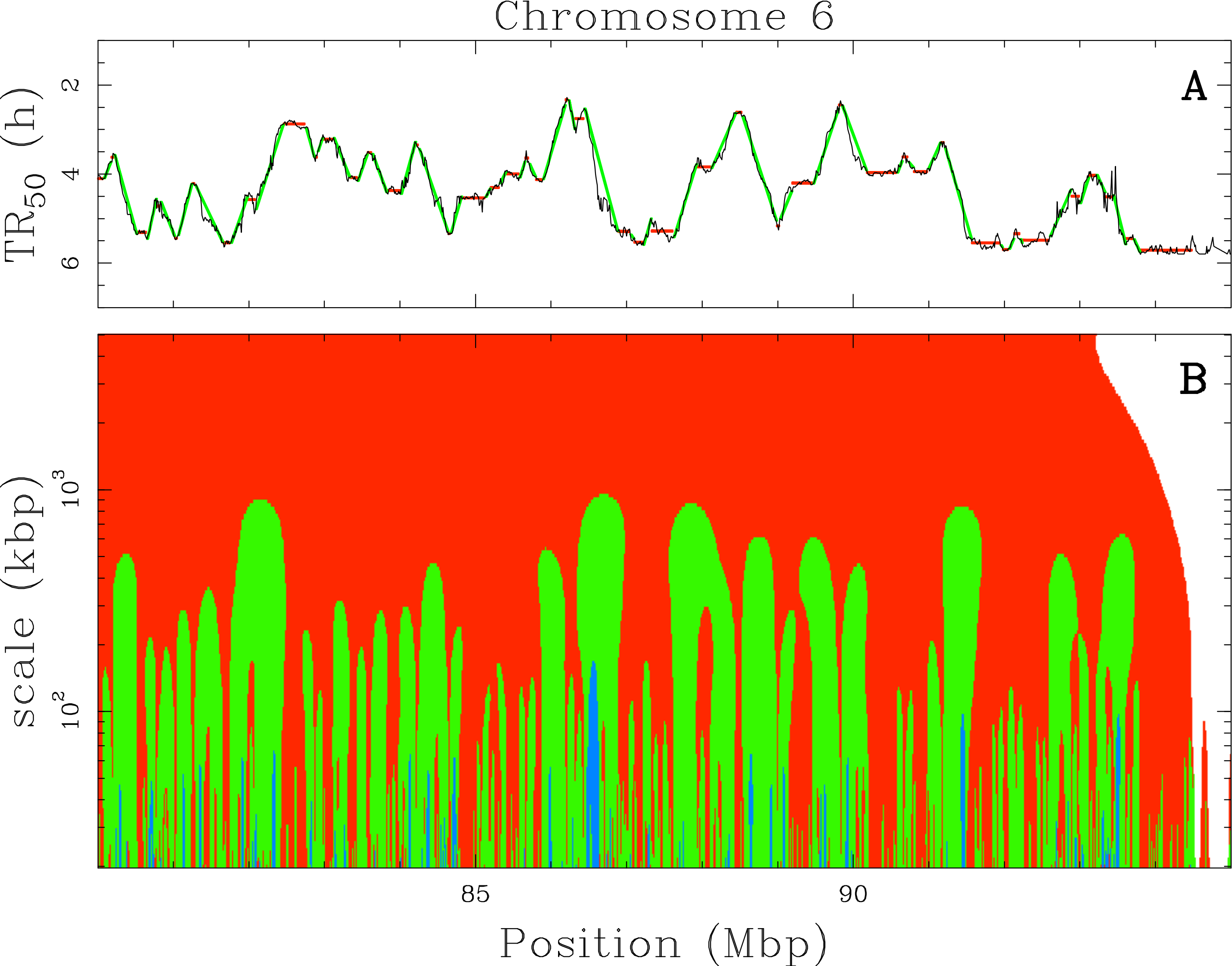

Chromosome 6
A
B
TR50 (h)
2
4
6
scale (kbp)
10^3
10^2
85
90
Position (Mbp)

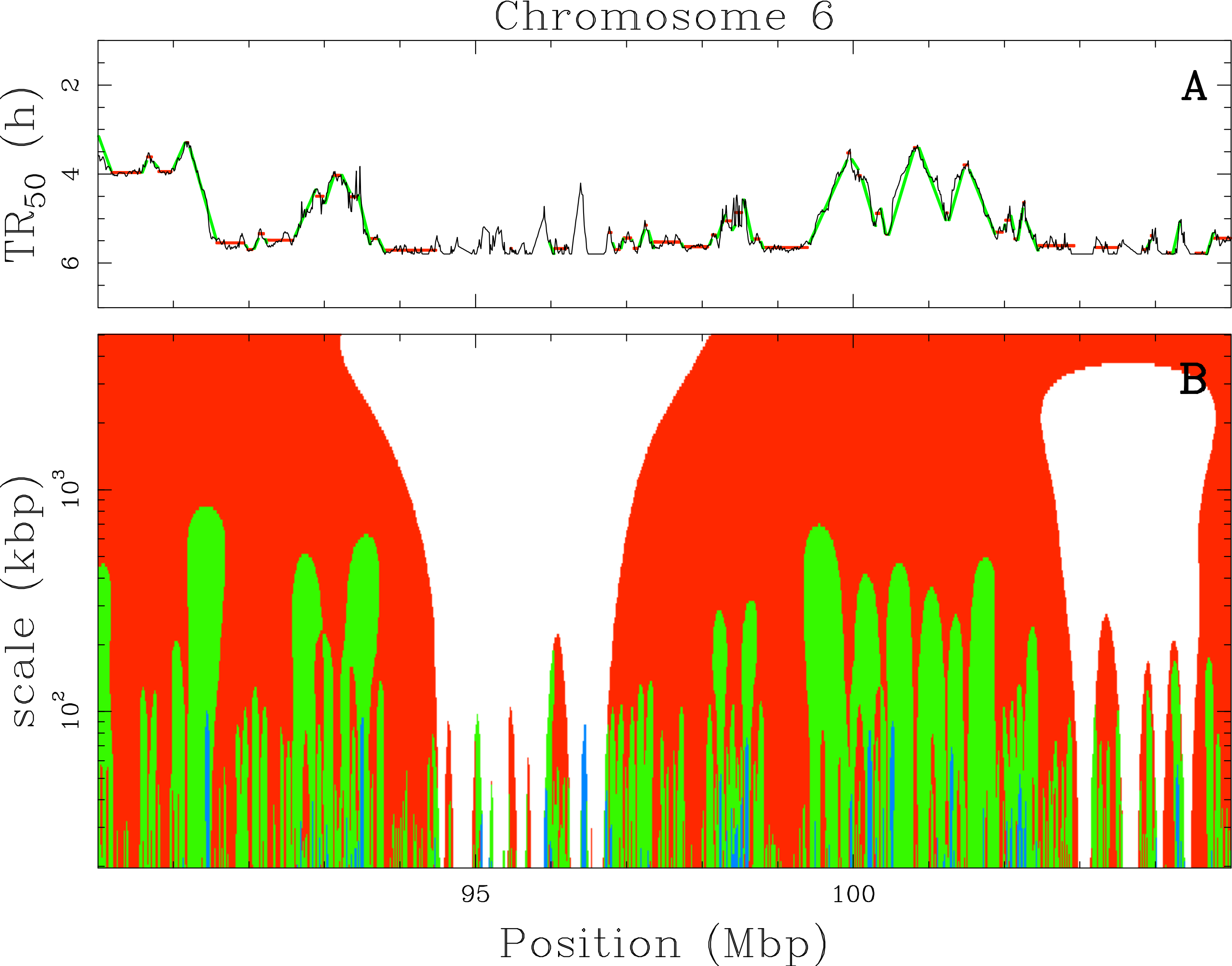

Chromosome 6
TR50 (h)
2
4
6
A
B
scale (kbp)
10^3
10^2
95
100
Position (Mbp)

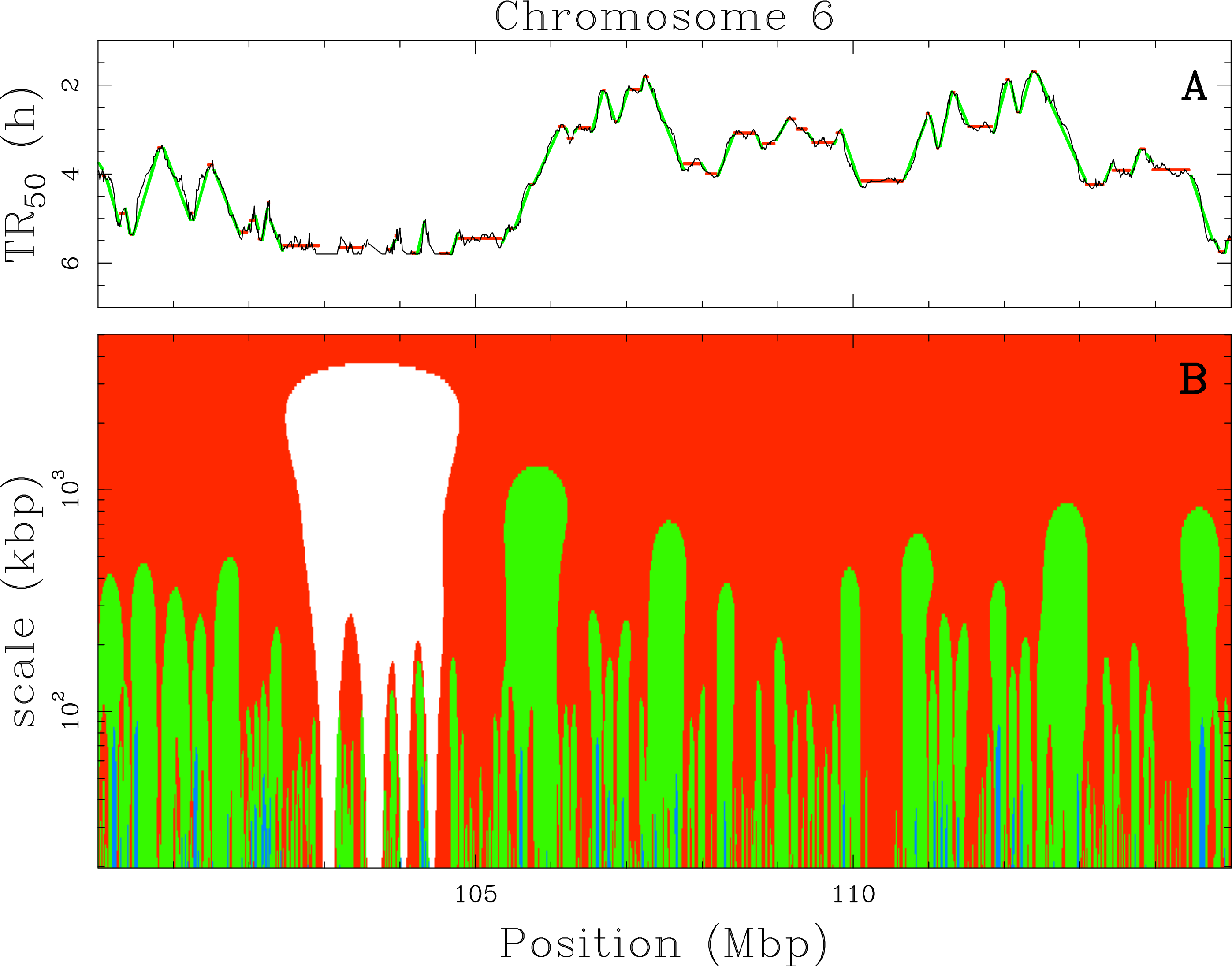
Chromosome 6
A
B
TR_{50} (h)
2
4
6
scale (kbp)
10^3
10^2
105
110
Position (Mbp)

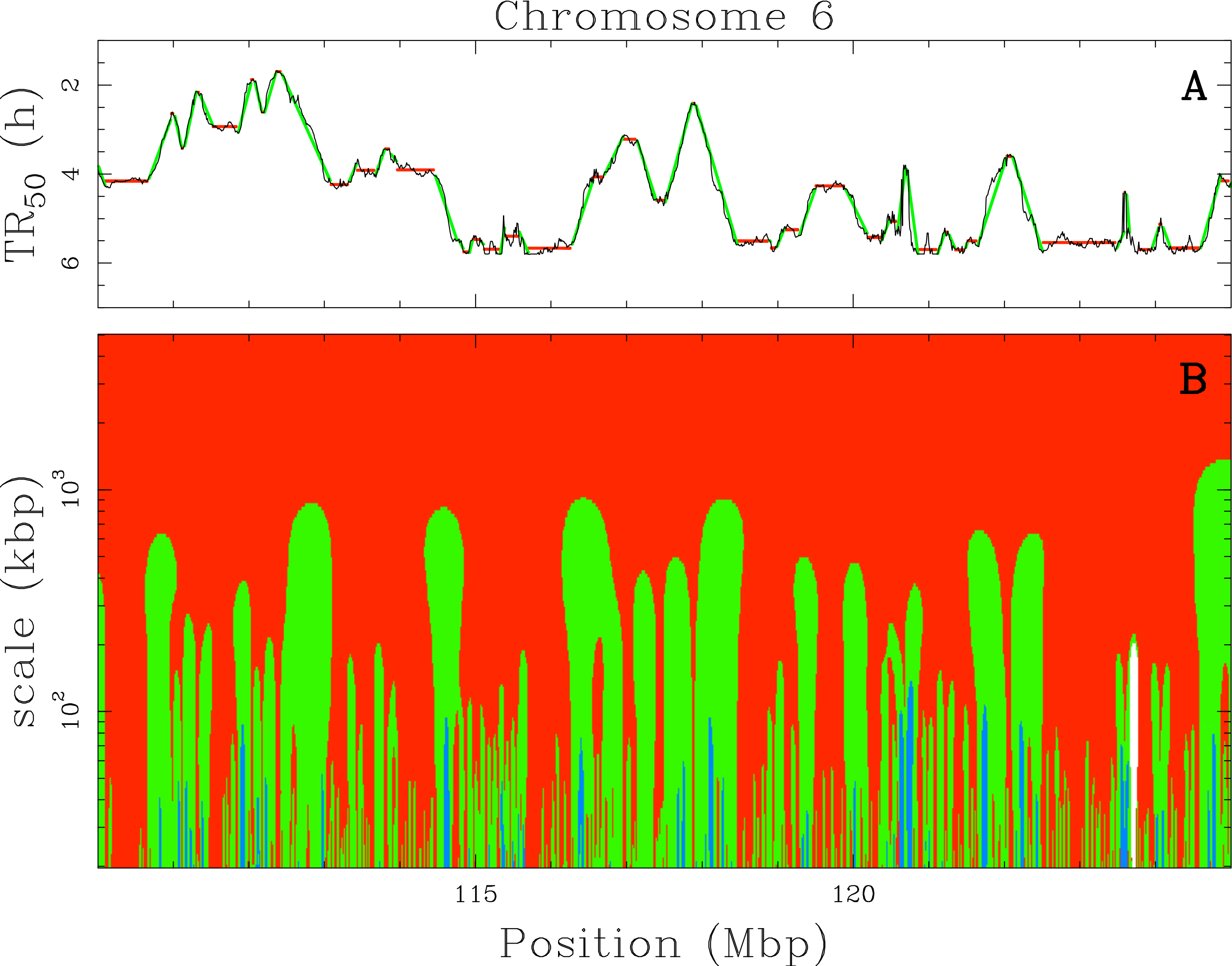

Chromosome 6
A
B
TR50 (h)
2
4
6
scale (kbp)
10^3
10^2
115
120
Position (Mbp)

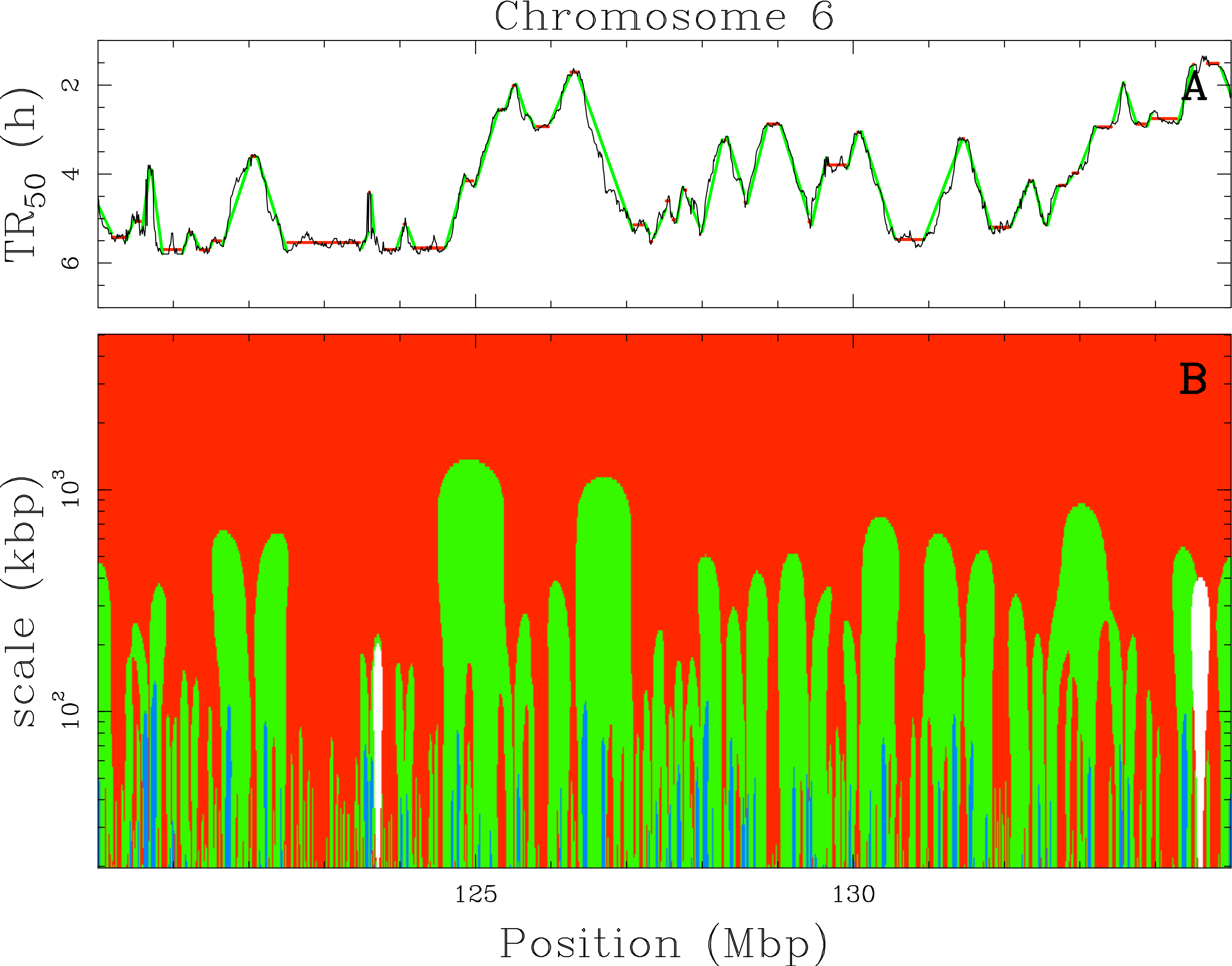

Chromosome 6
A
B
TR50 (h)
2
4
6
scale (kbp)
10^3
10^2
125
130
Position (Mbp)

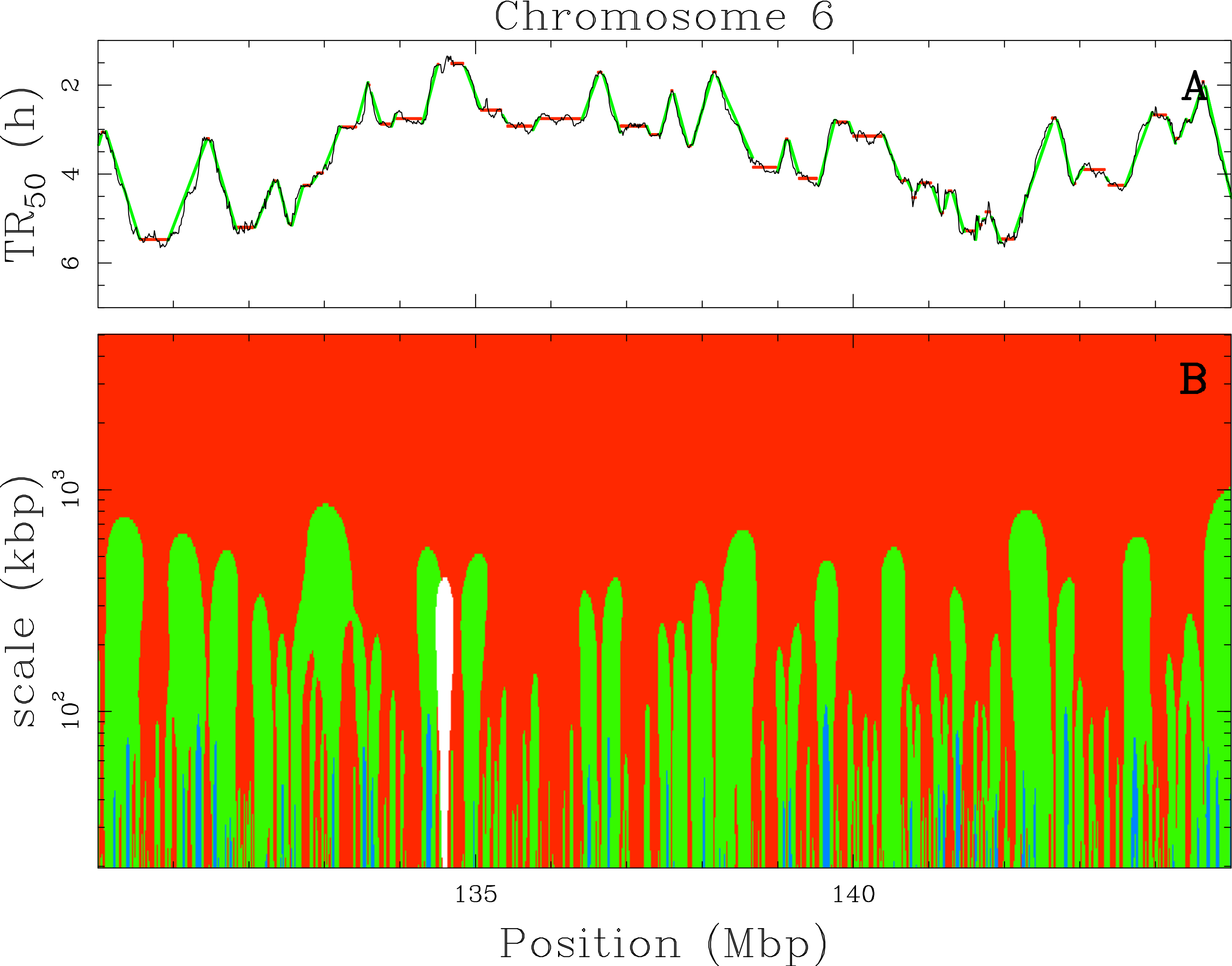

Chromosome 6
A
TR_{50} (h)
2
4
6
B
scale (kbp)
10^3
10^2
135
140
Position (Mbp)

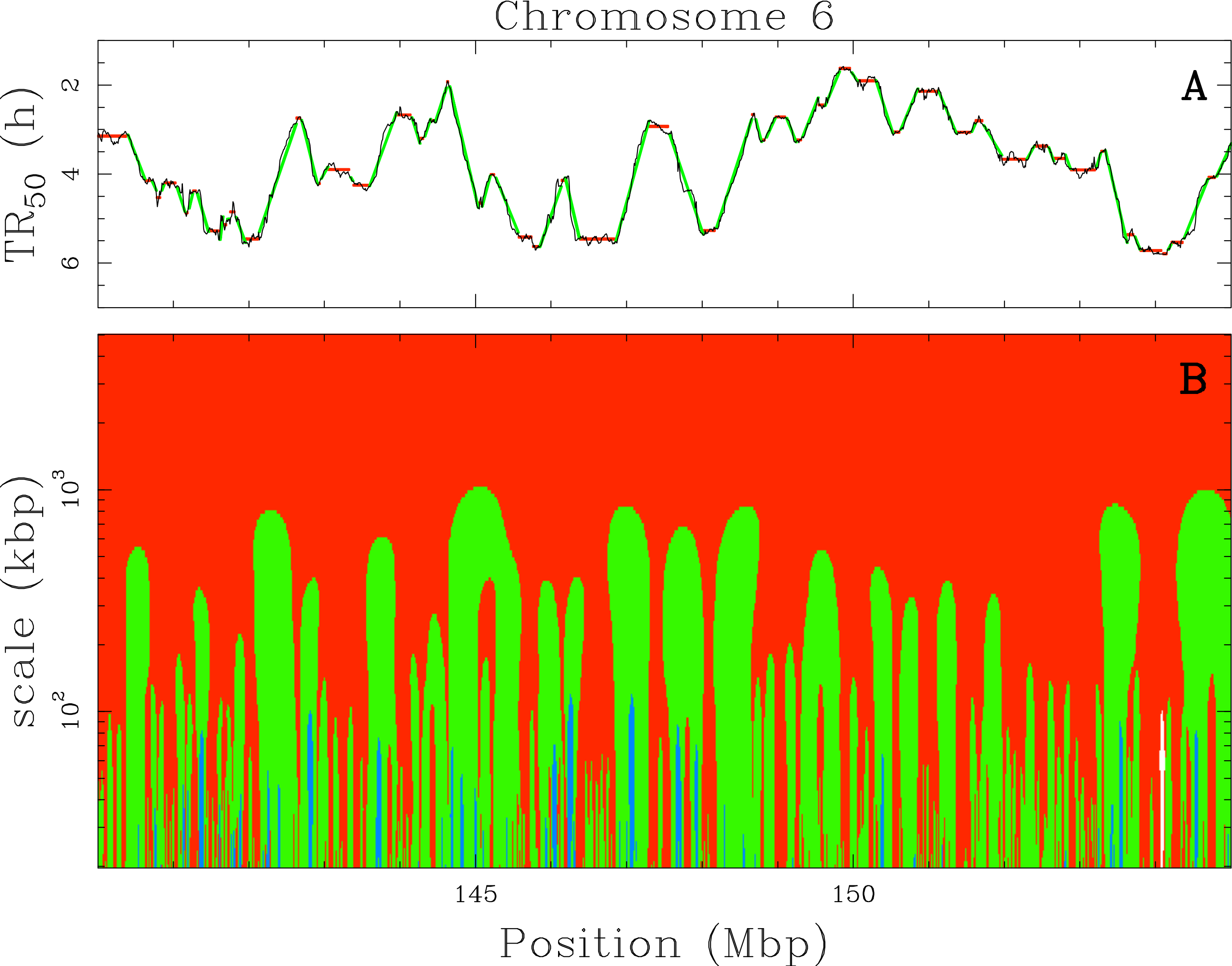

Chromosome 6
A
B
TR50 (h)
2
4
6
scale (kbp)
10^3
10^2
145
150
Position (Mbp)

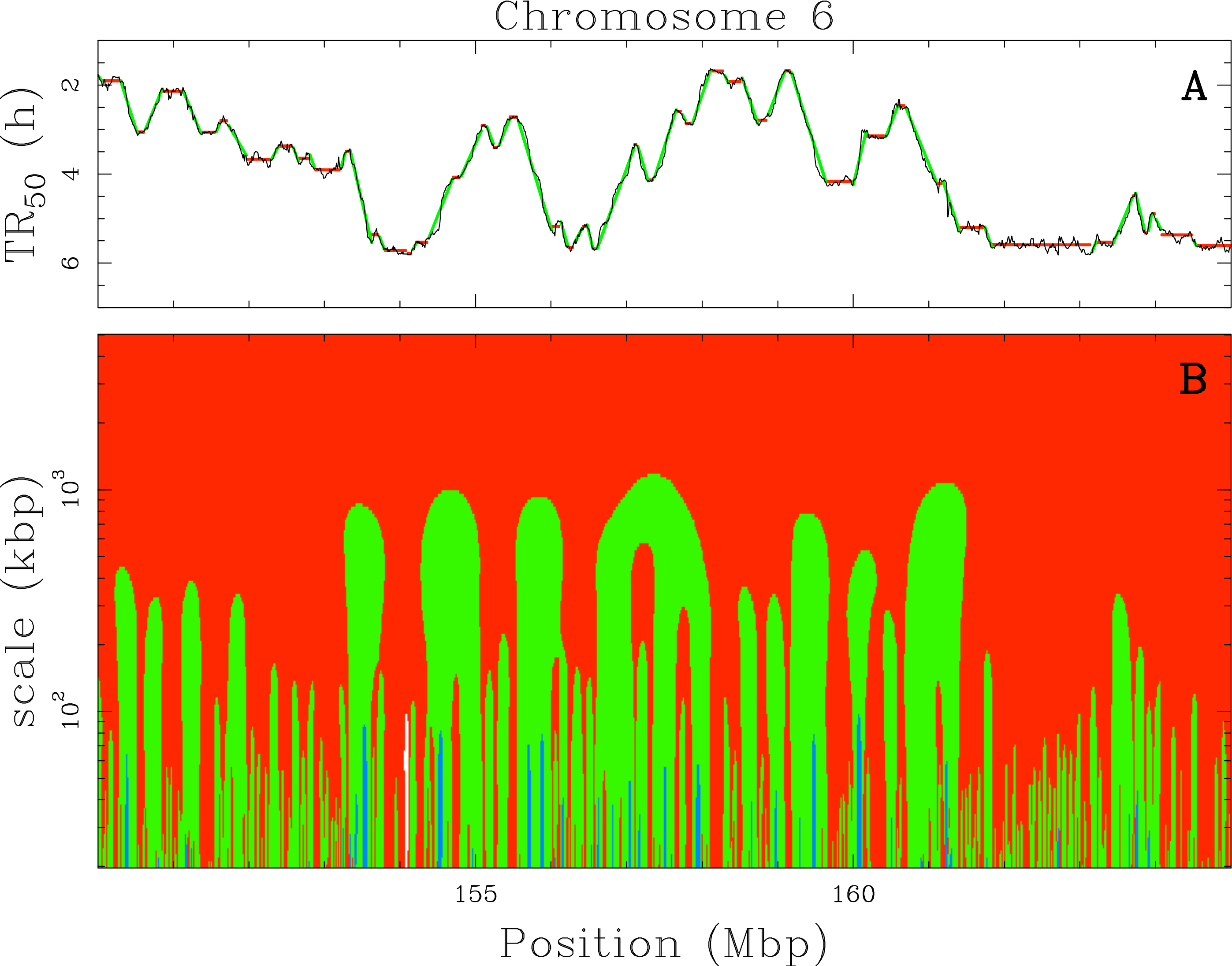

Chromosome 6
A
B
TR50 (h)
2
4
6
scale (kbp)
10^3
10^2
155
160
Position (Mbp)

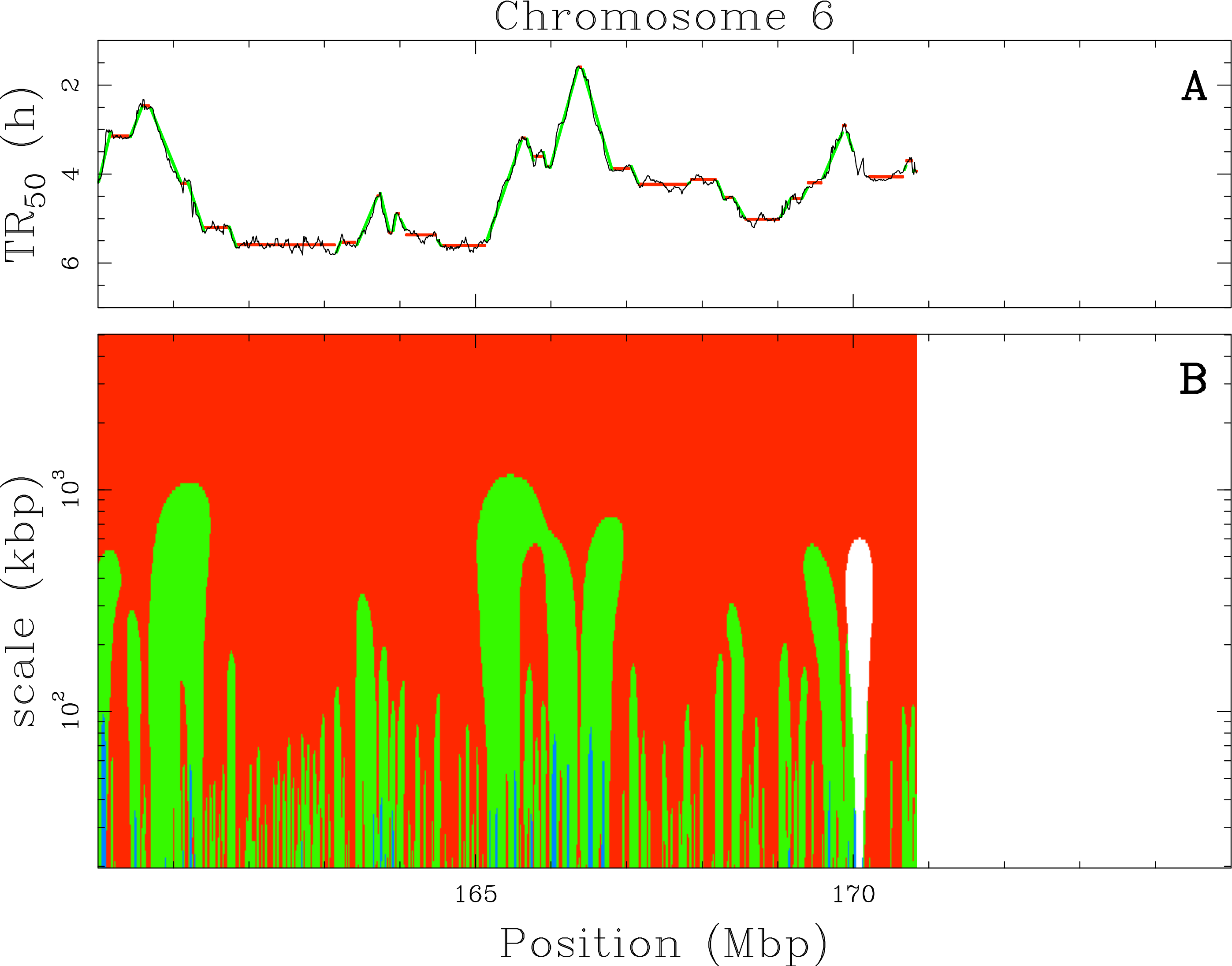

Chromosome 6
TR50 (h)
2
4
6
A
scale (kbp)
10^3
10^2
B
165
170
Position (Mbp)

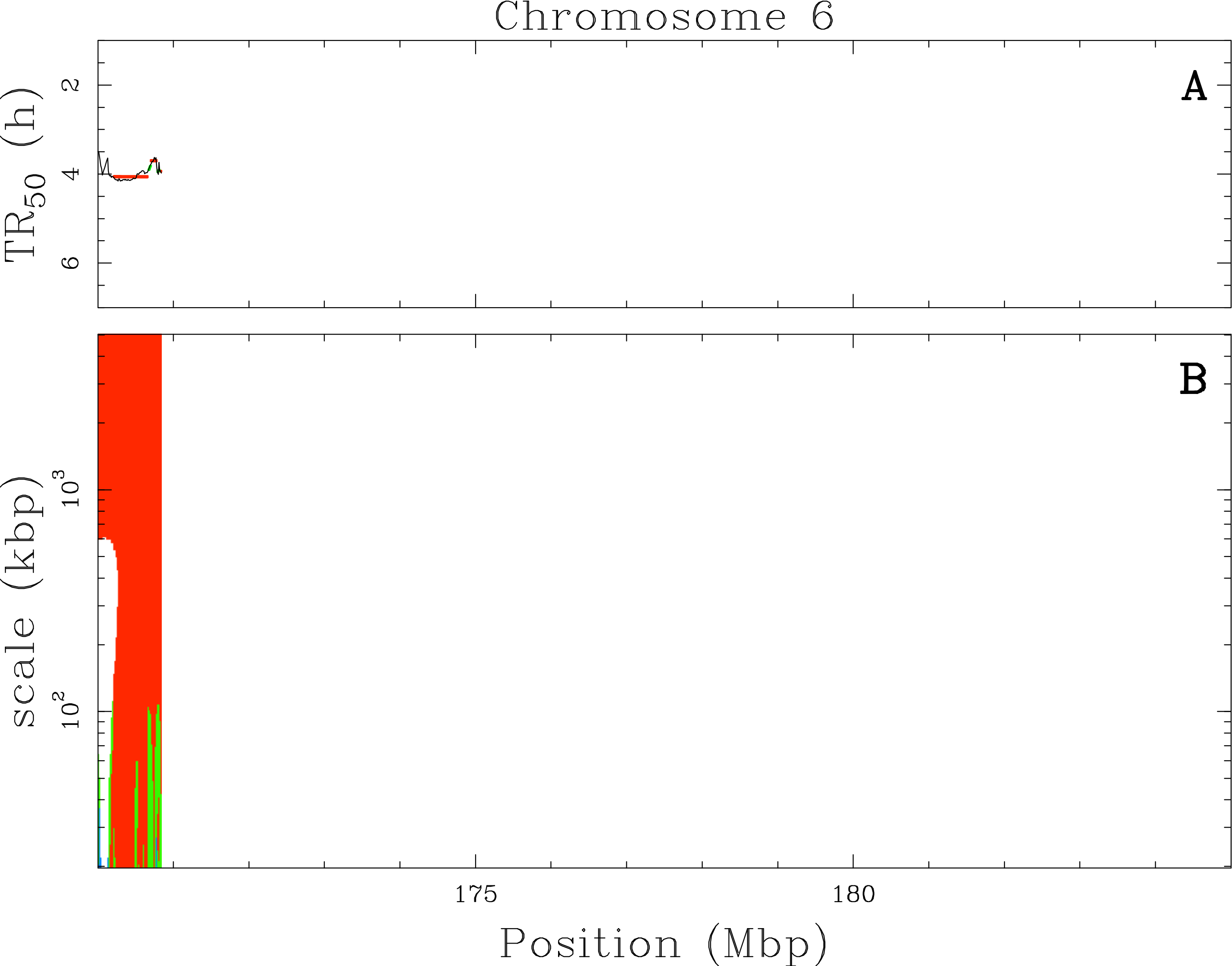
Chromosome 6
TR50 (h)
2
4
6
A
scale (kbp)
10^3
10^2
B
175
180
Position (Mbp)

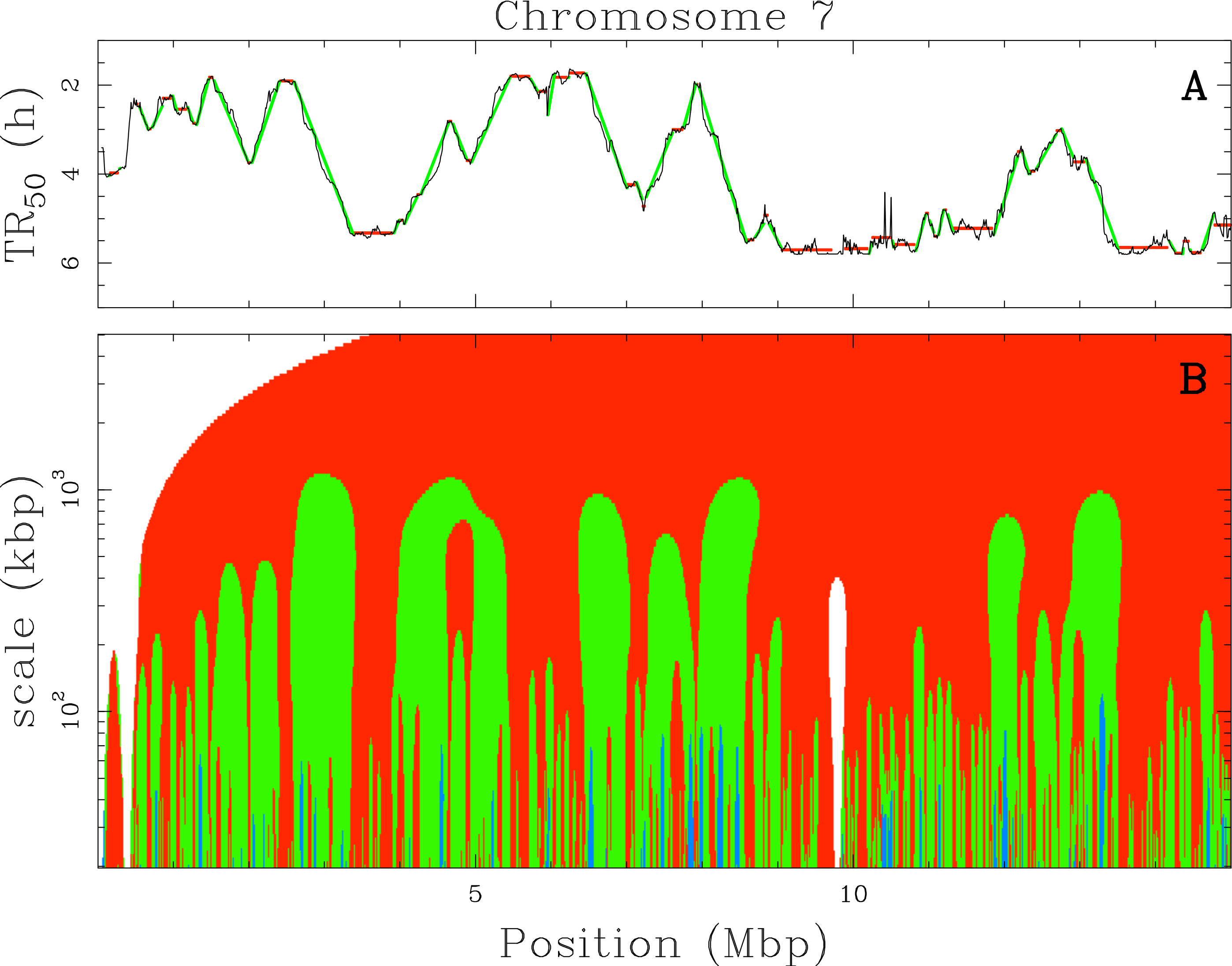

Chromosome 7
A
B
TR_{50} (h)
2
4
6
scale (kbp)
10^3
10^2
5
10
Position (Mbp)

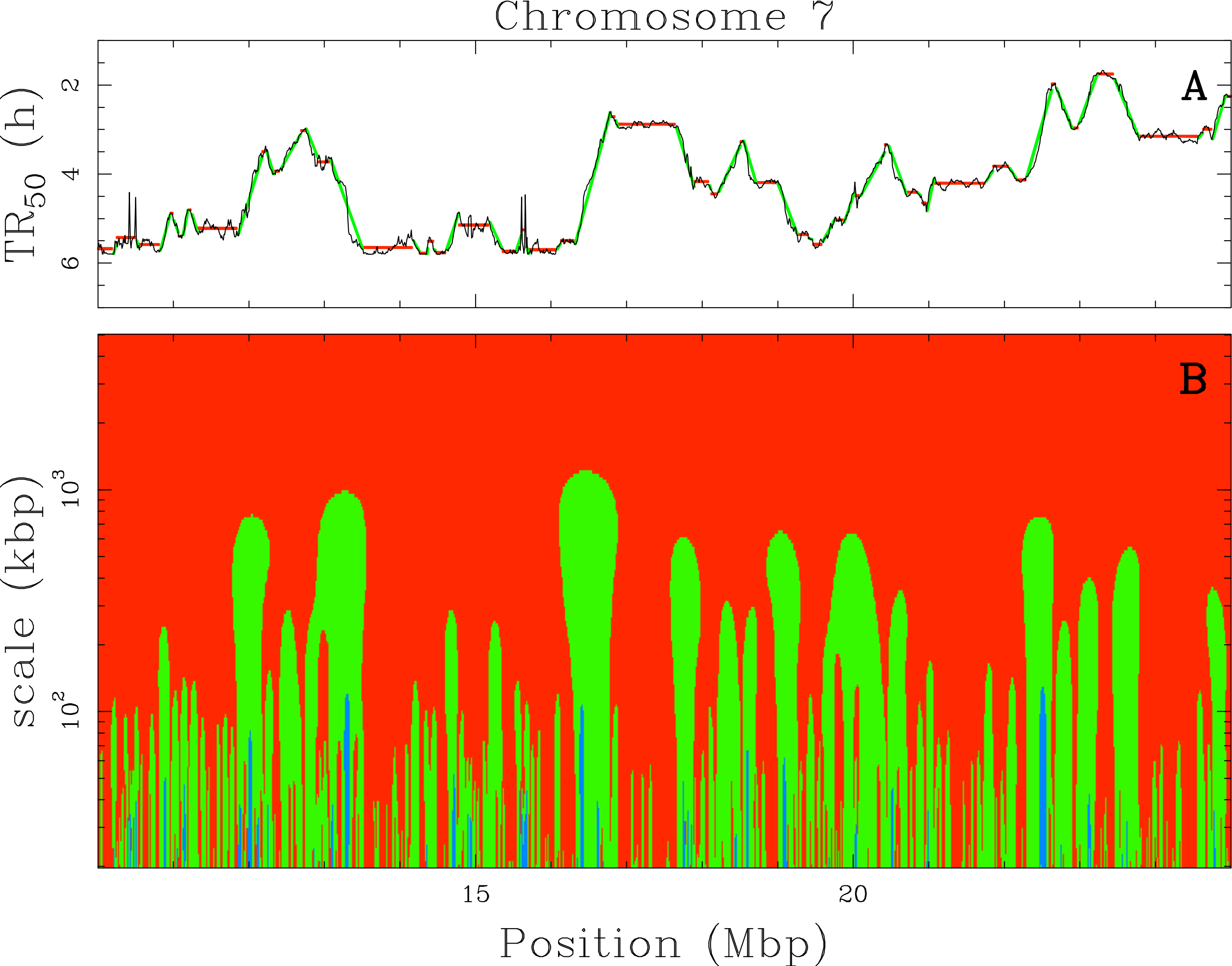

Chromosome 7
A
B
TR_{50} (h)
2
4
6
scale (kbp)
10^3
10^2
15
20
Position (Mbp)

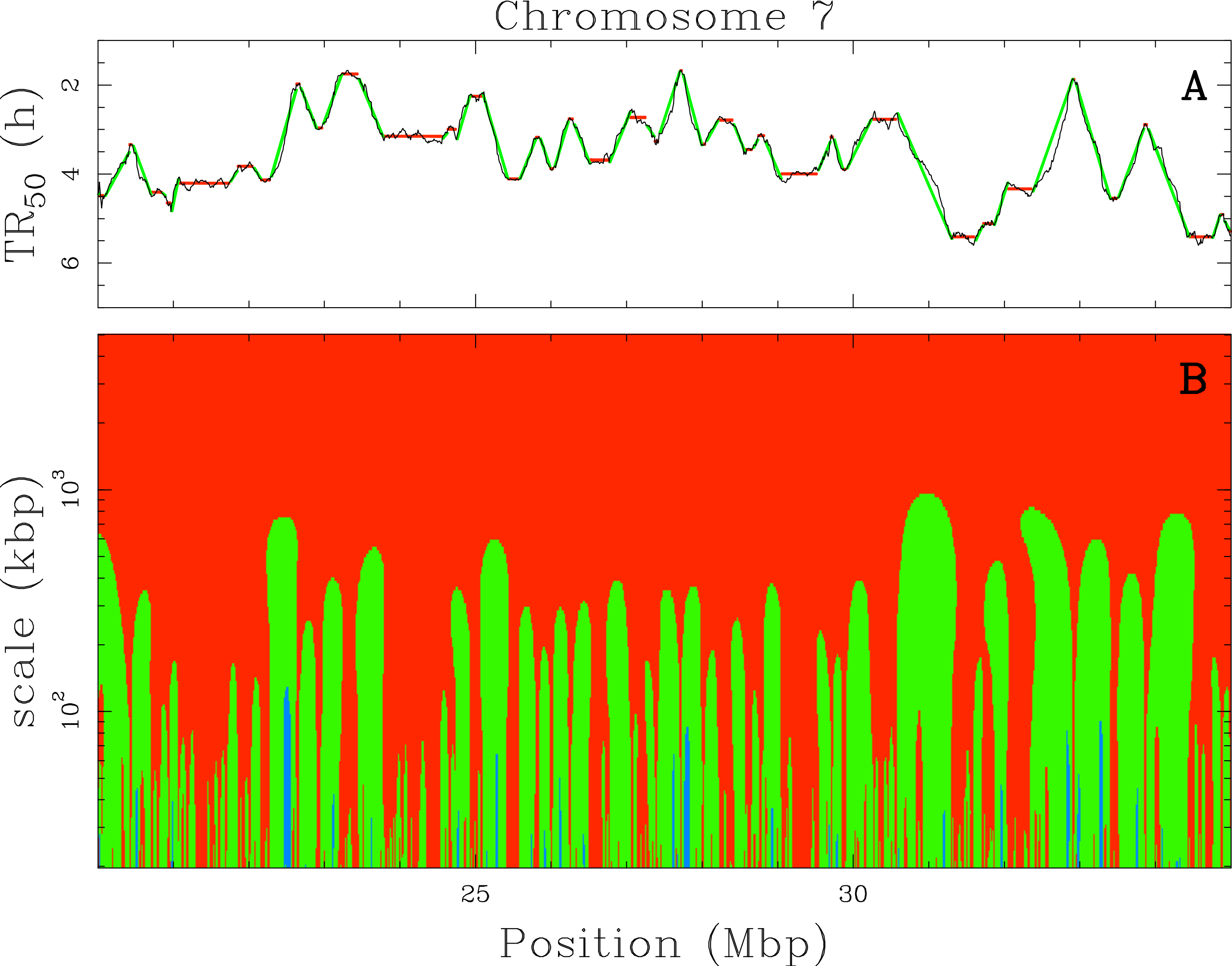

Chromosome 7
A
B
TR_{50} (h)
2
4
6
scale (kbp)
10^3
10^2
25
30
Position (Mbp)

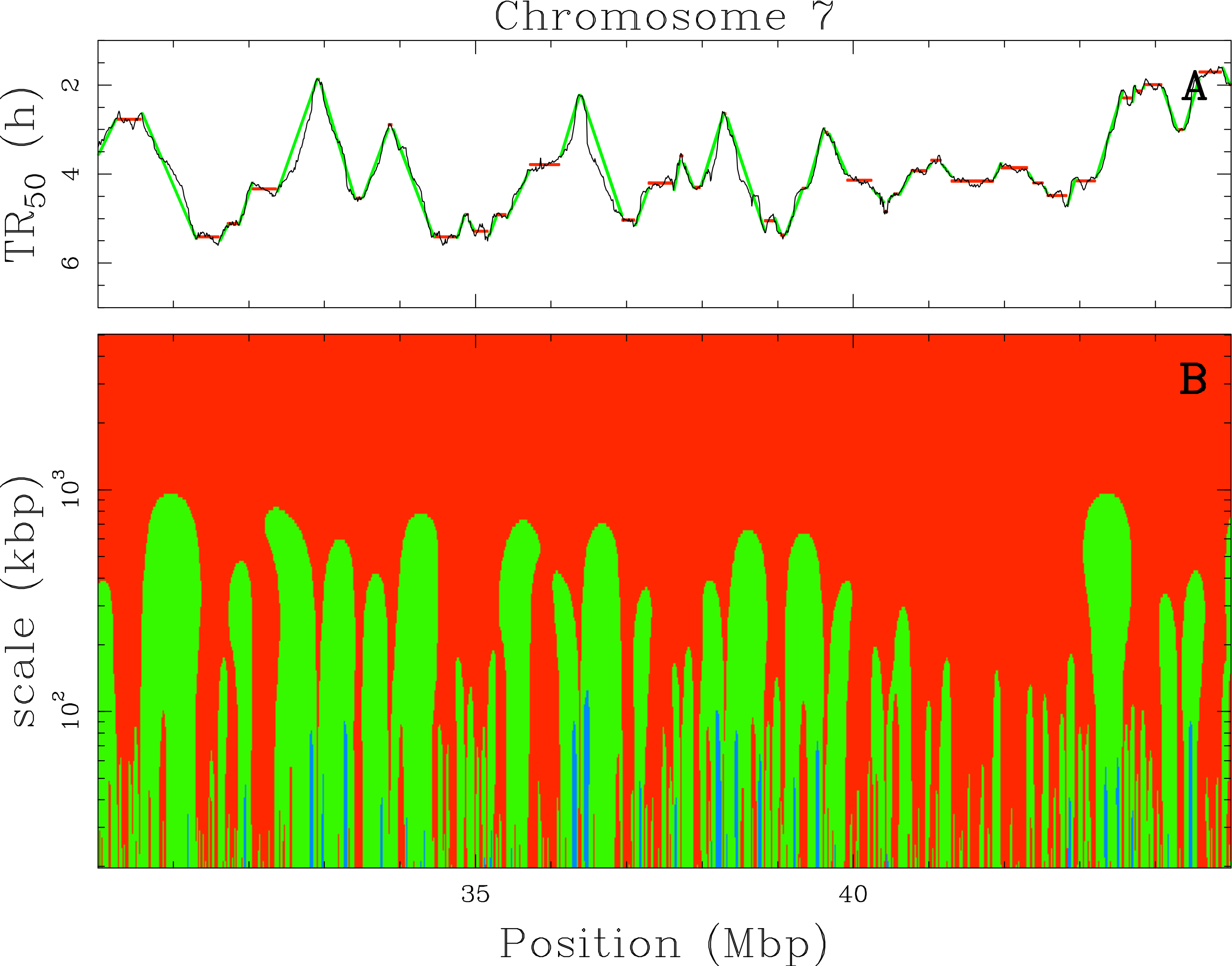

Chromosome 7
A
TR50 (h)
2
4
6
B
scale (kbp)
10^3
10^2
35
40
Position (Mbp)

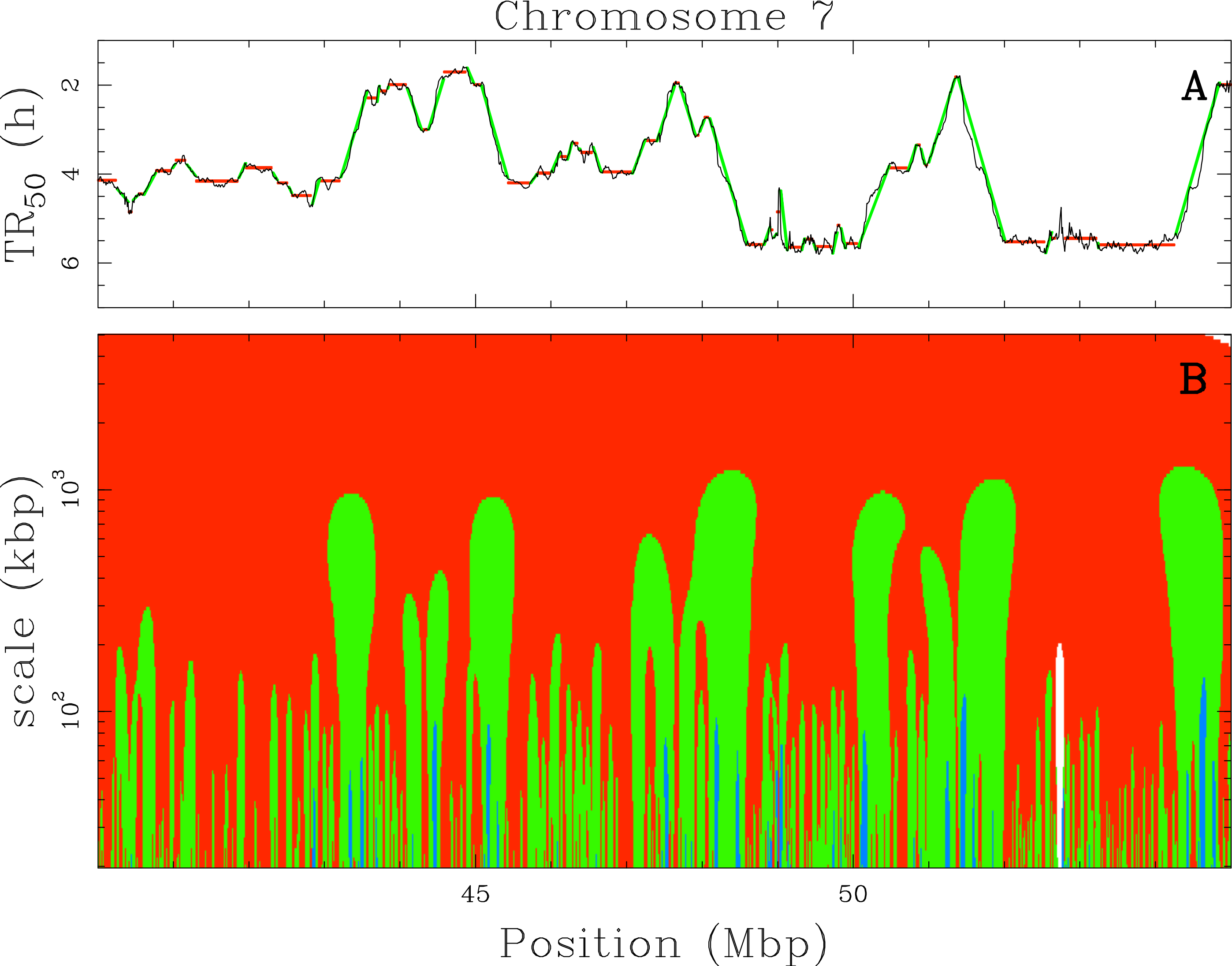

Chromosome 7
A
B
TR_{50} (h)
2
4
6
scale (kbp)
10^3
10^2
45
50
Position (Mbp)

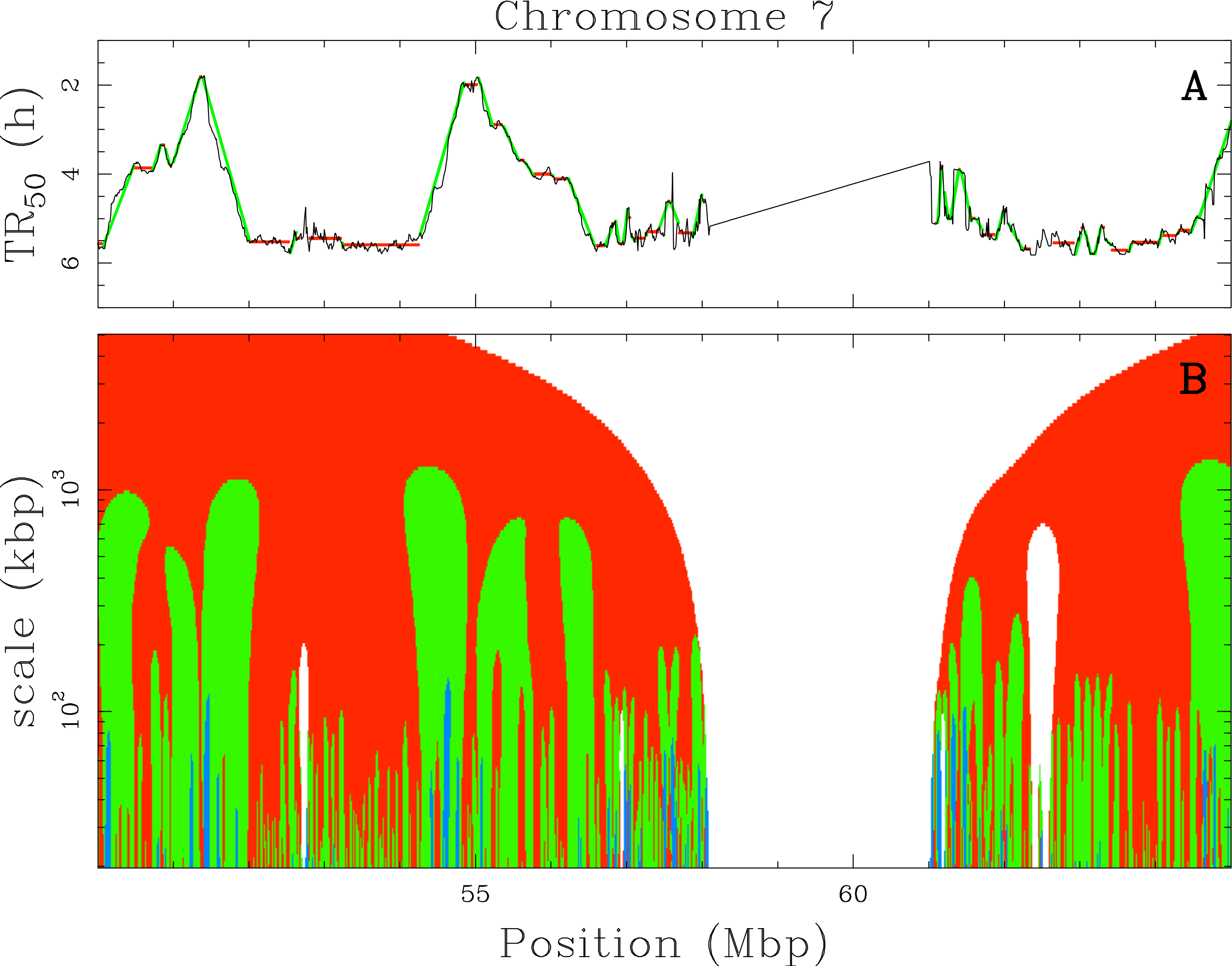

Chromosome 7
TR$_{50}$ (h)
2
4
6
A
B
scale (kbp)
10^3
10^2
55
60
Position (Mbp)

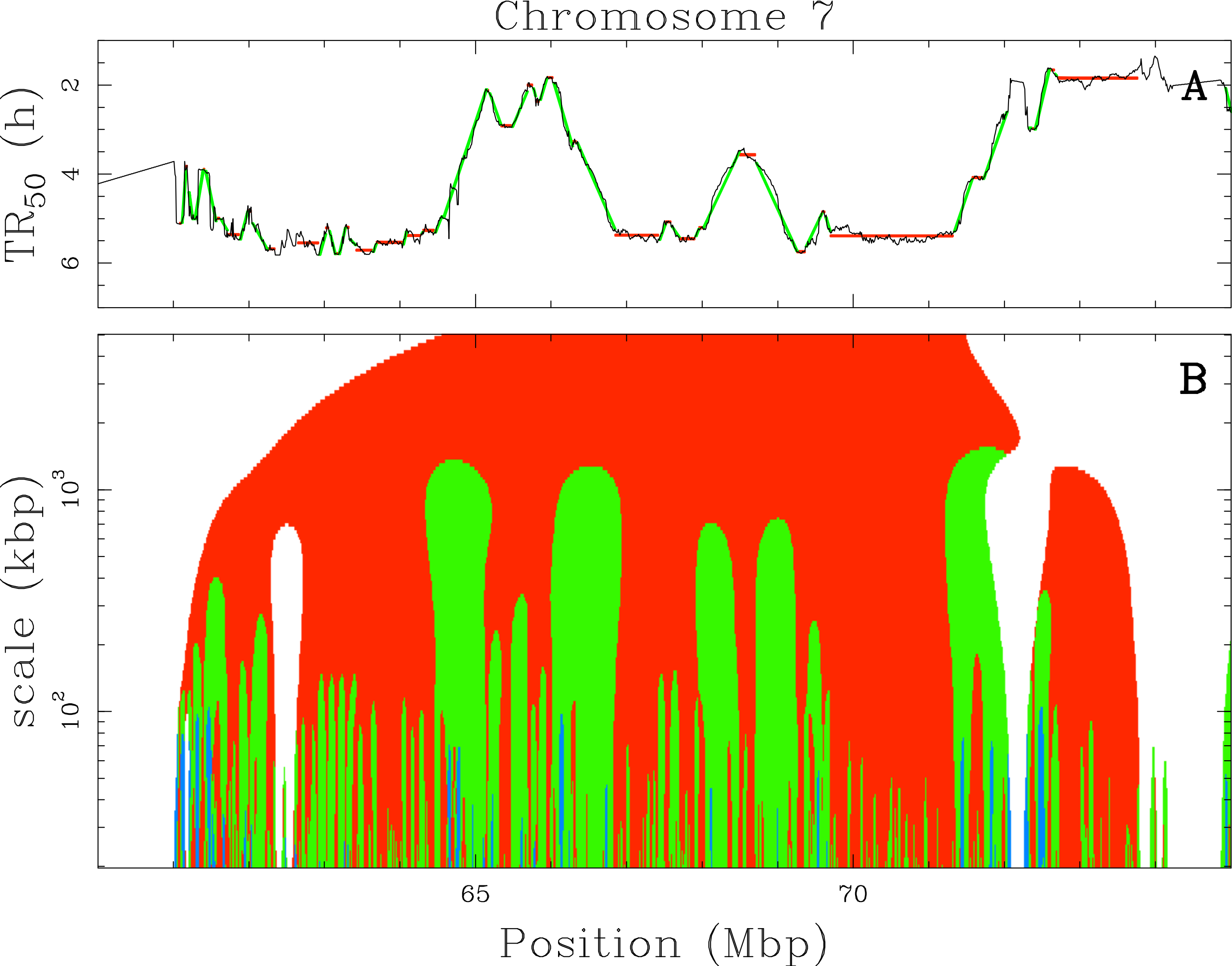

Chromosome 7
A
B
TR$_{50}$ (h)
2
4
6
scale (kbp)
10^3
10^2
65
70
Position (Mbp)

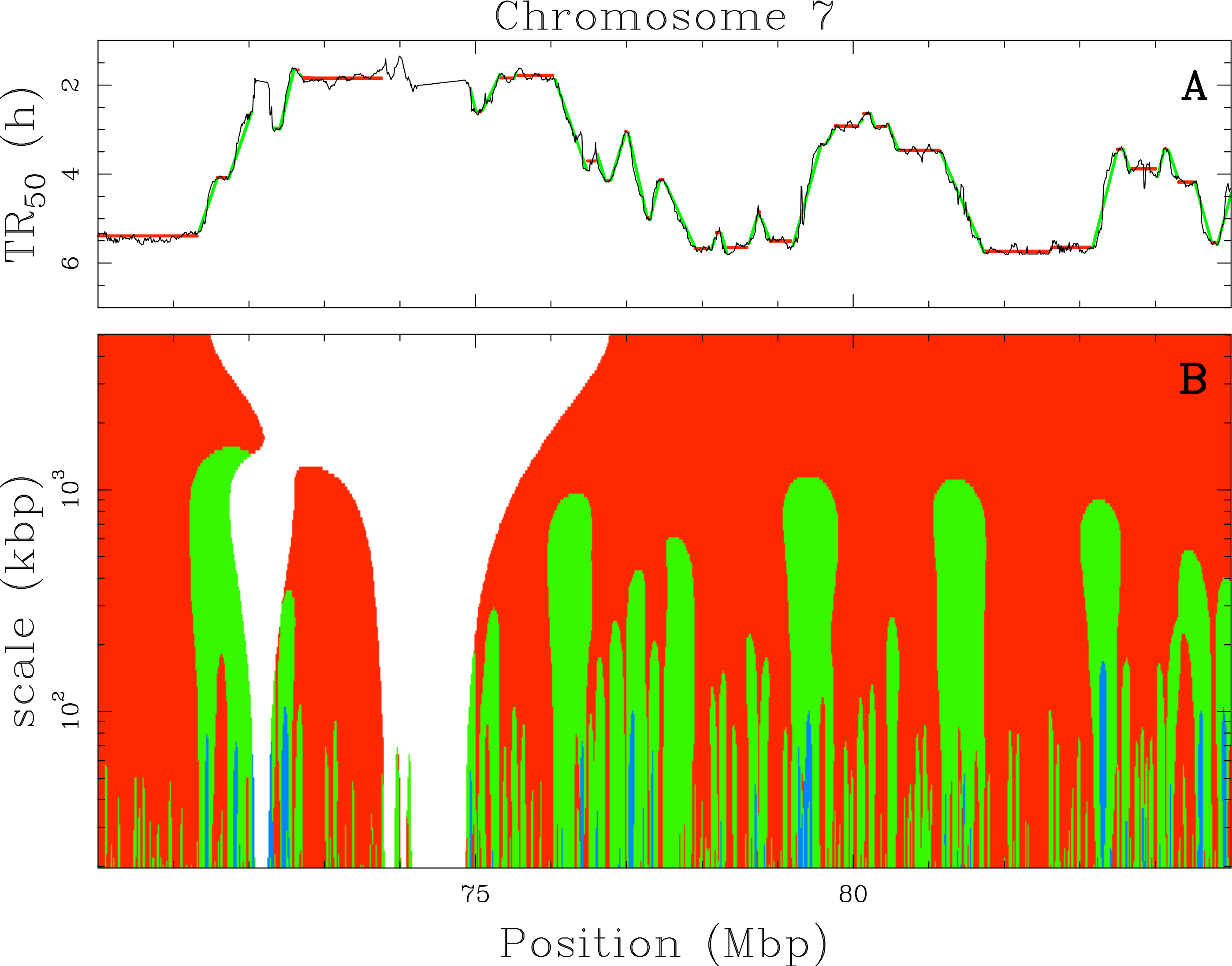

Chromosome 7
A
TR50 (h)
2
4
6
B
scale (kbp)
10^3
10^2
75
80
Position (Mbp)

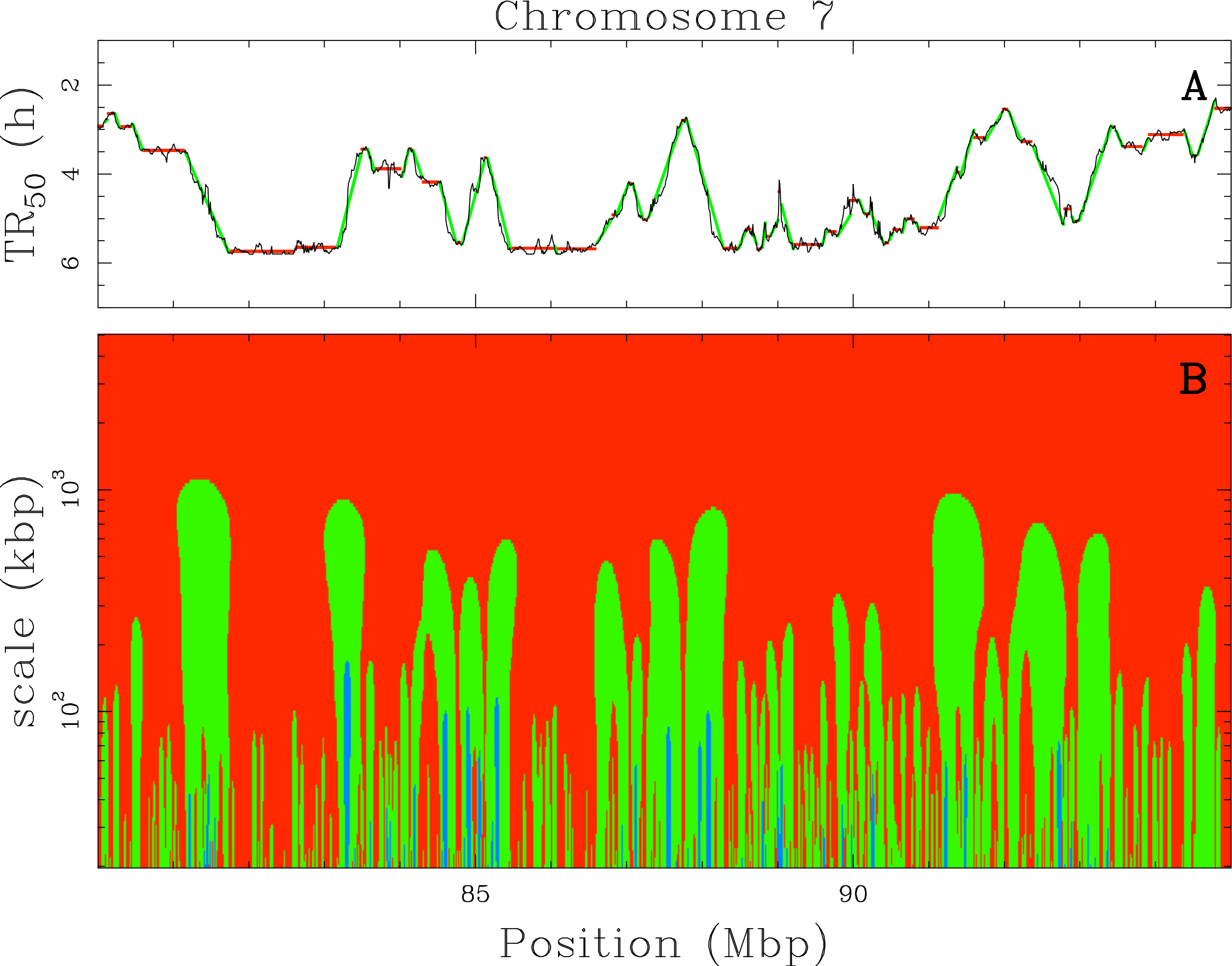

Chromosome 7
A
TR_{50} (h)
2
4
6
B
scale (kbp)
10^3
10^2
85
90
Position (Mbp)

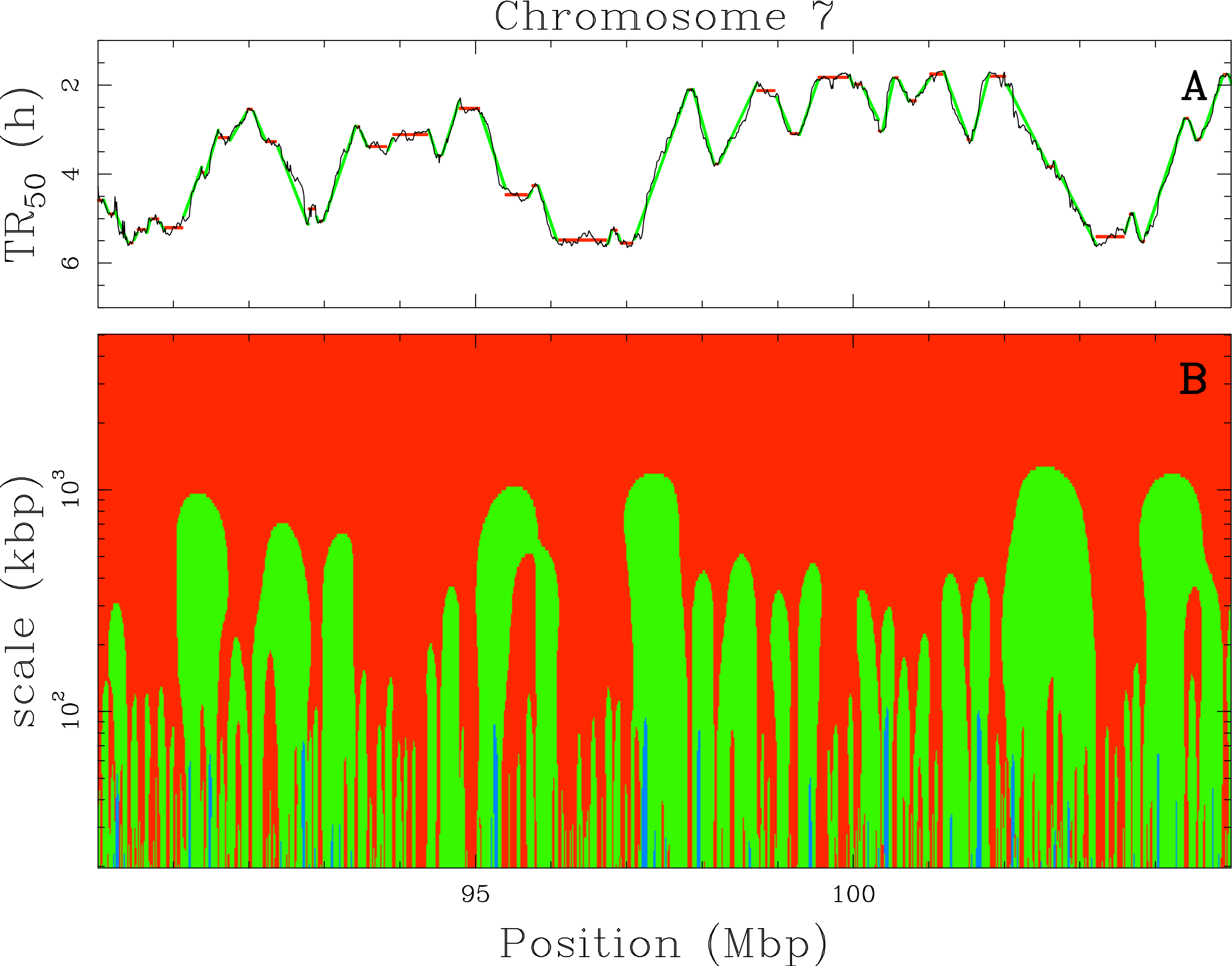

Chromosome 7
A
B
TR50 (h)
2
4
6
scale (kbp)
10^3
10^2
95
100
Position (Mbp)

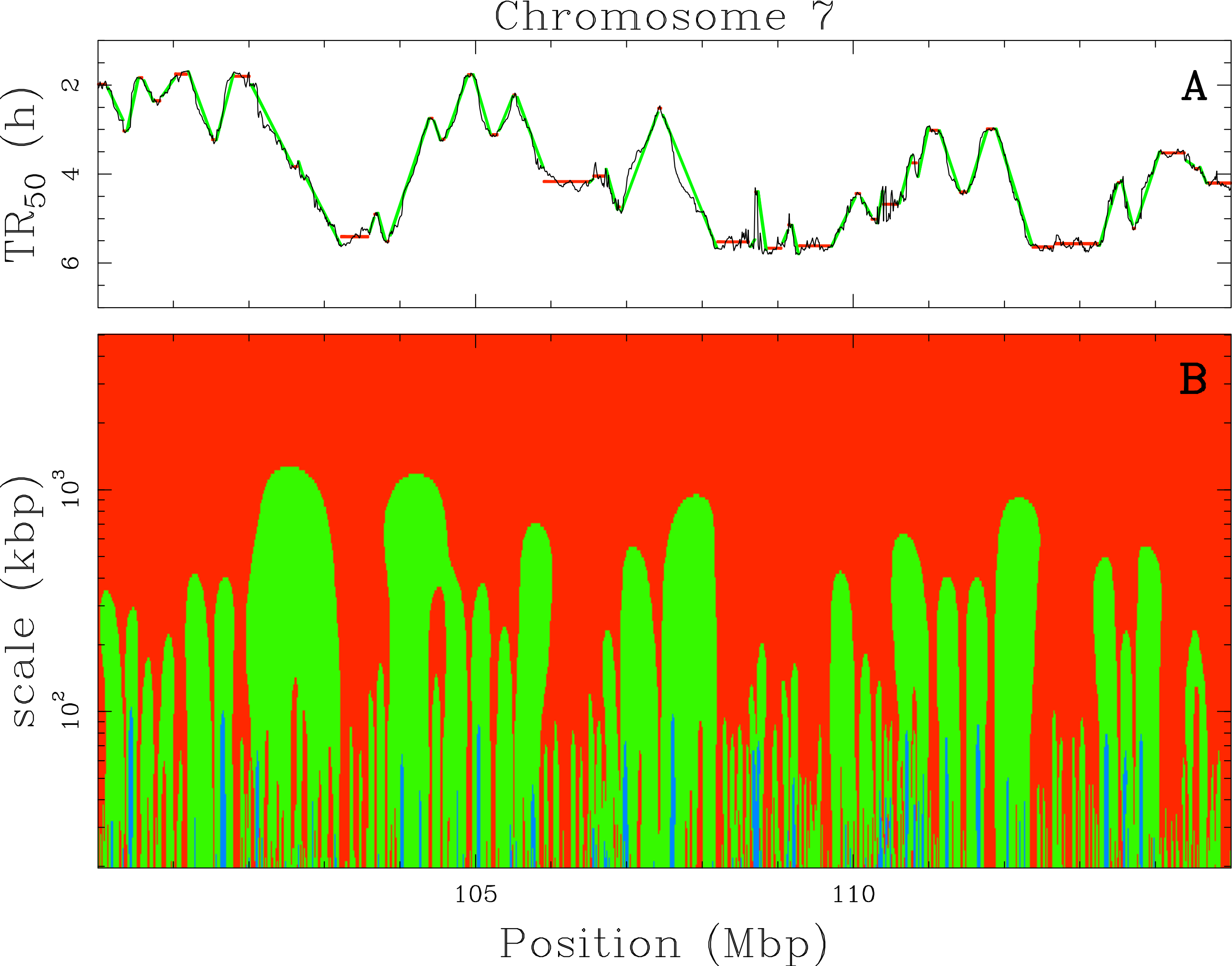
Chromosome 7
A
B
TR50 (h)
2
4
6
scale (kbp)
10^3
10^2
105
110
Position (Mbp)

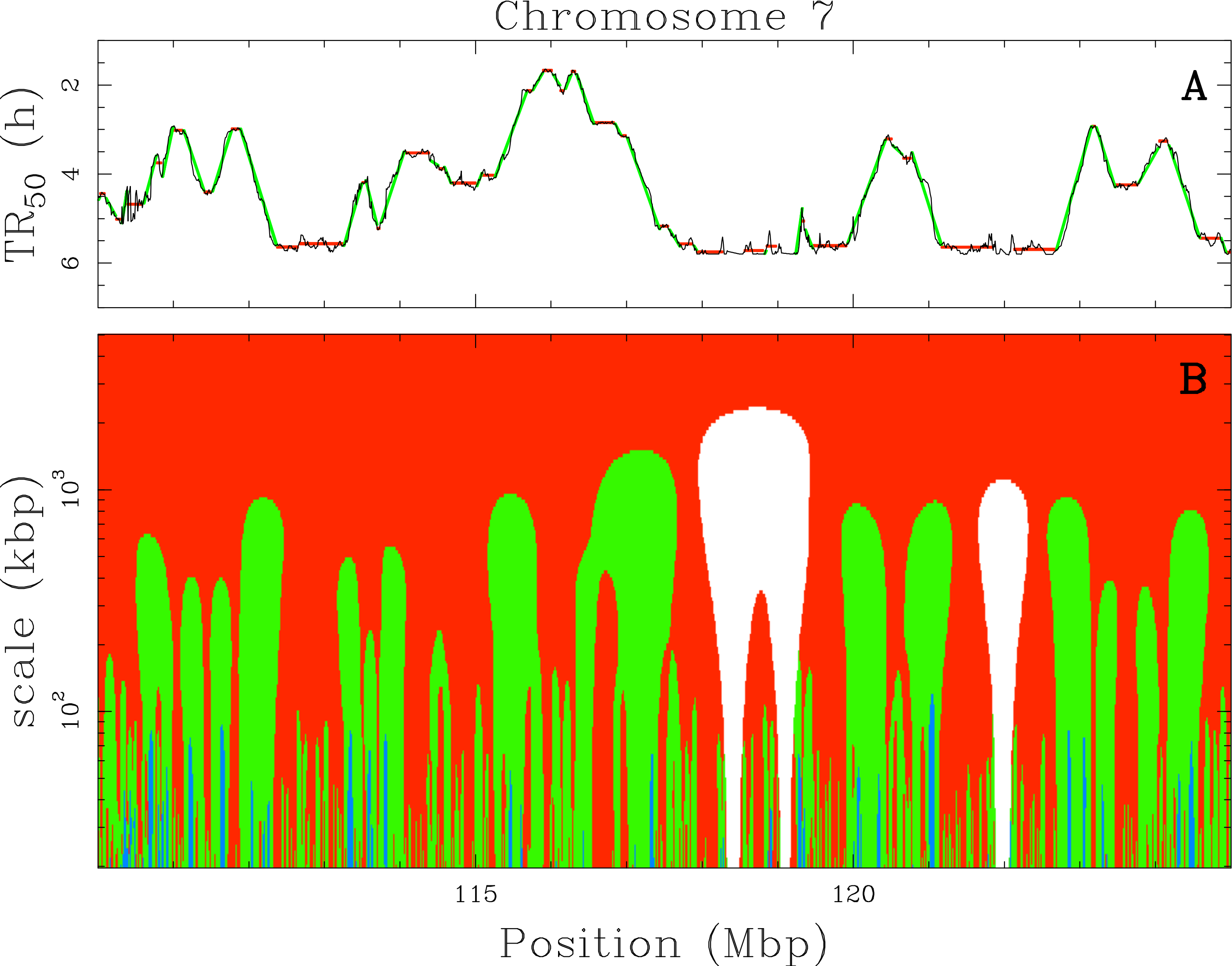

Chromosome 7
A
B
TR50 (h)
2
4
6
scale (kbp)
10^3
10^2
115
120
Position (Mbp)

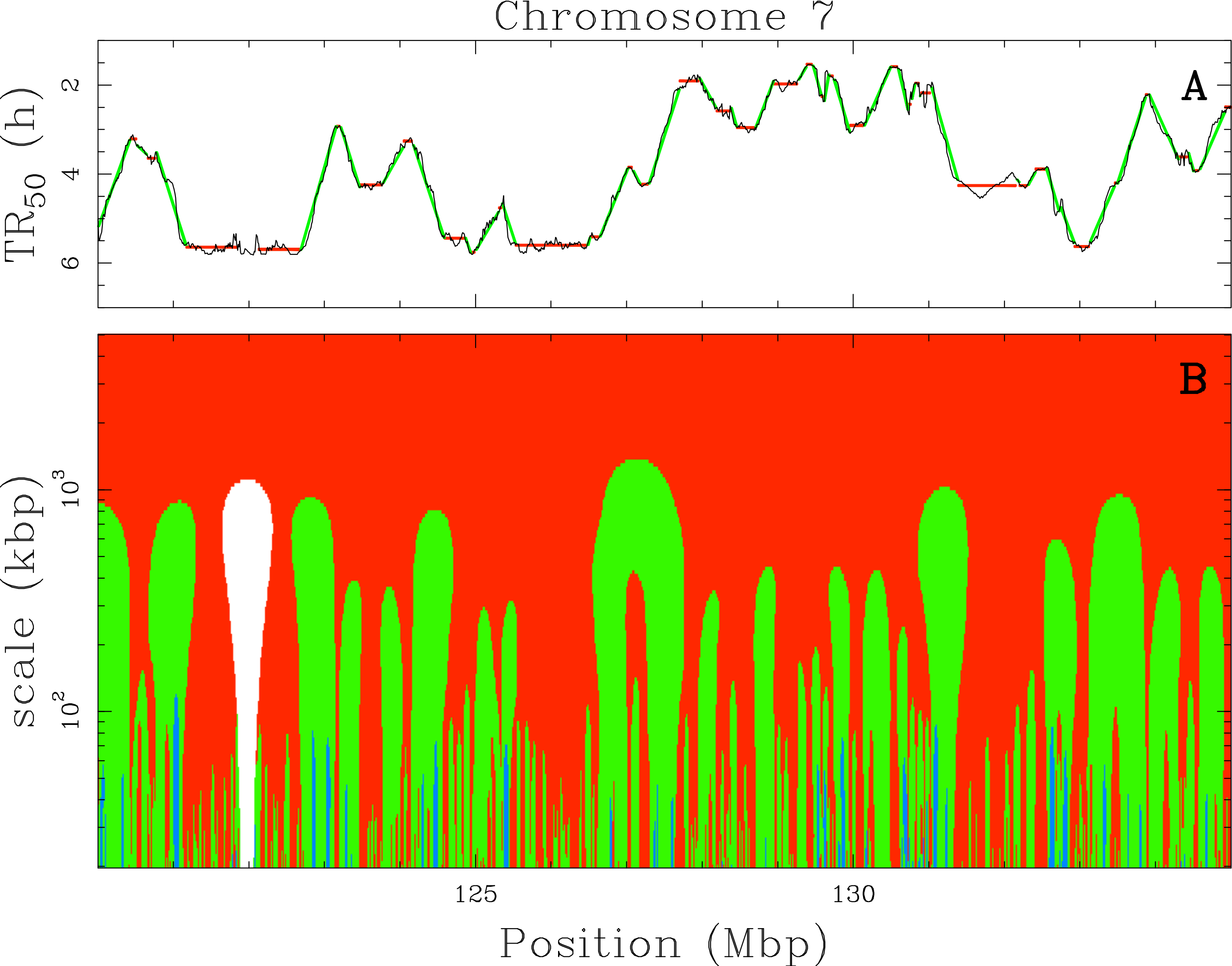

Chromosome 7
A
B
TR_{50} (h)
2
4
6
scale (kbp)
10^3
10^2
125
130
Position (Mbp)

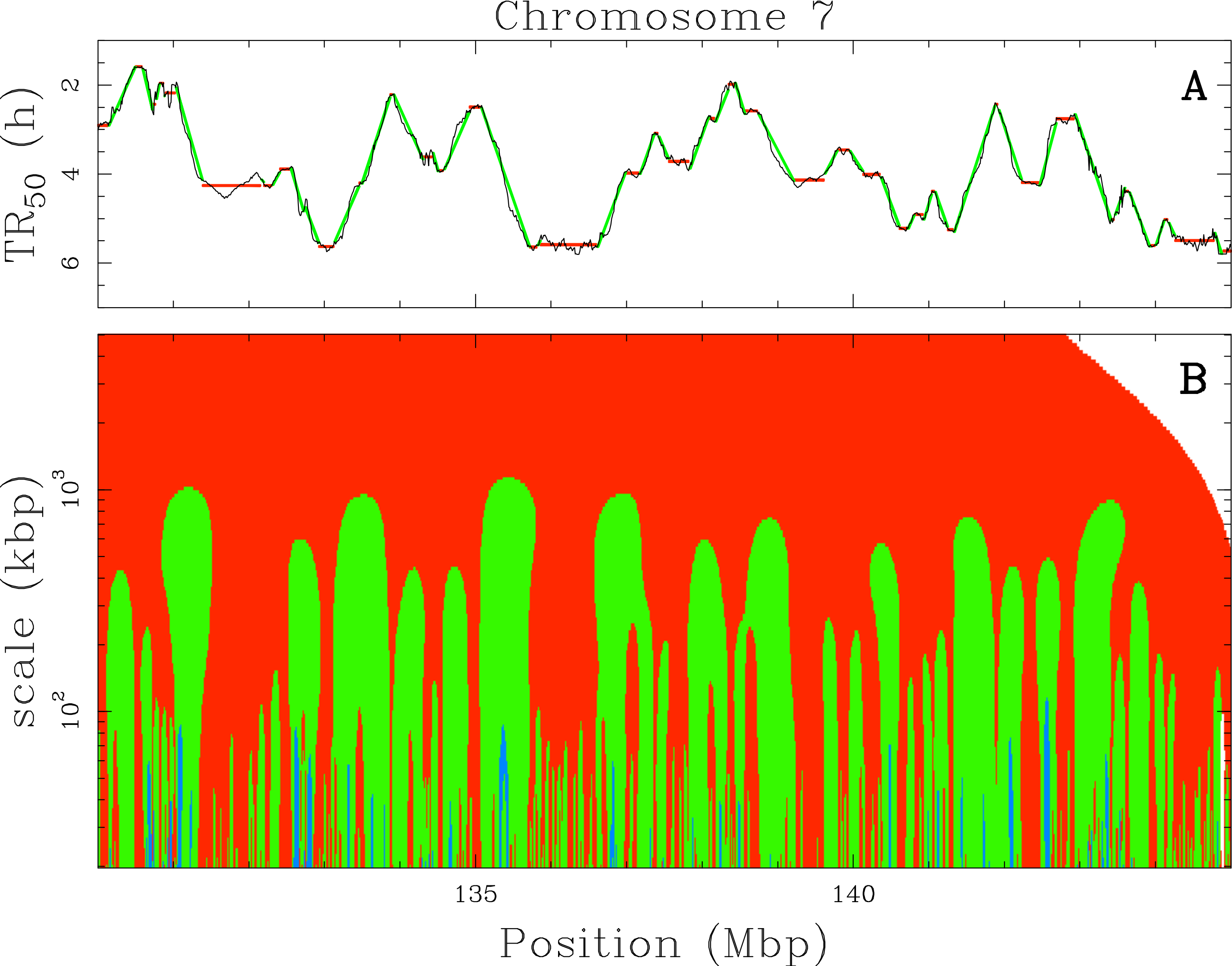

Chromosome 7
A
B
TR50 (h)
2
4
6
scale (kbp)
10^3
10^2
135
140
Position (Mbp)

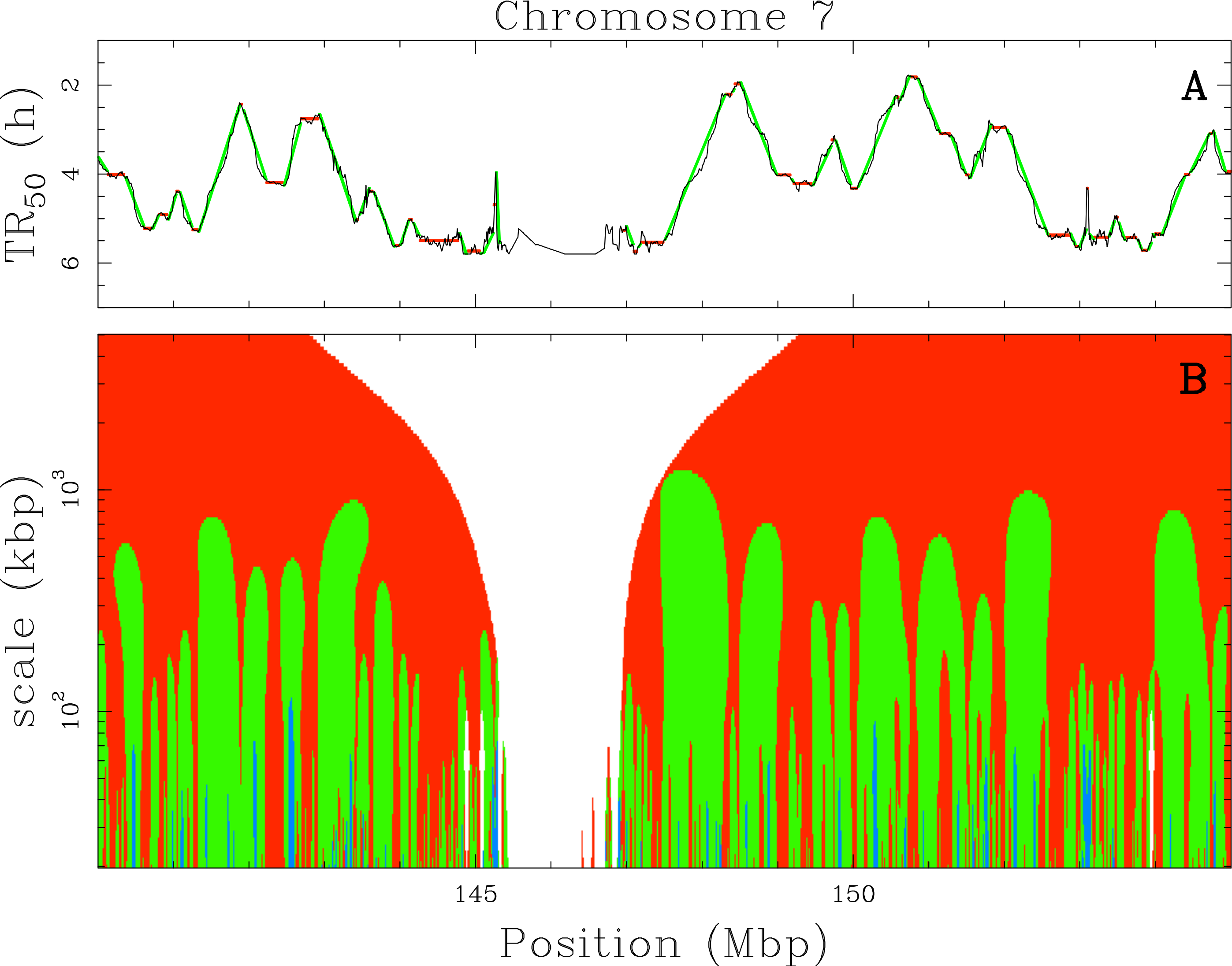

Chromosome 7
A
B
TR50 (h)
2
4
6
scale (kbp)
10^3
10^2
145
150
Position (Mbp)

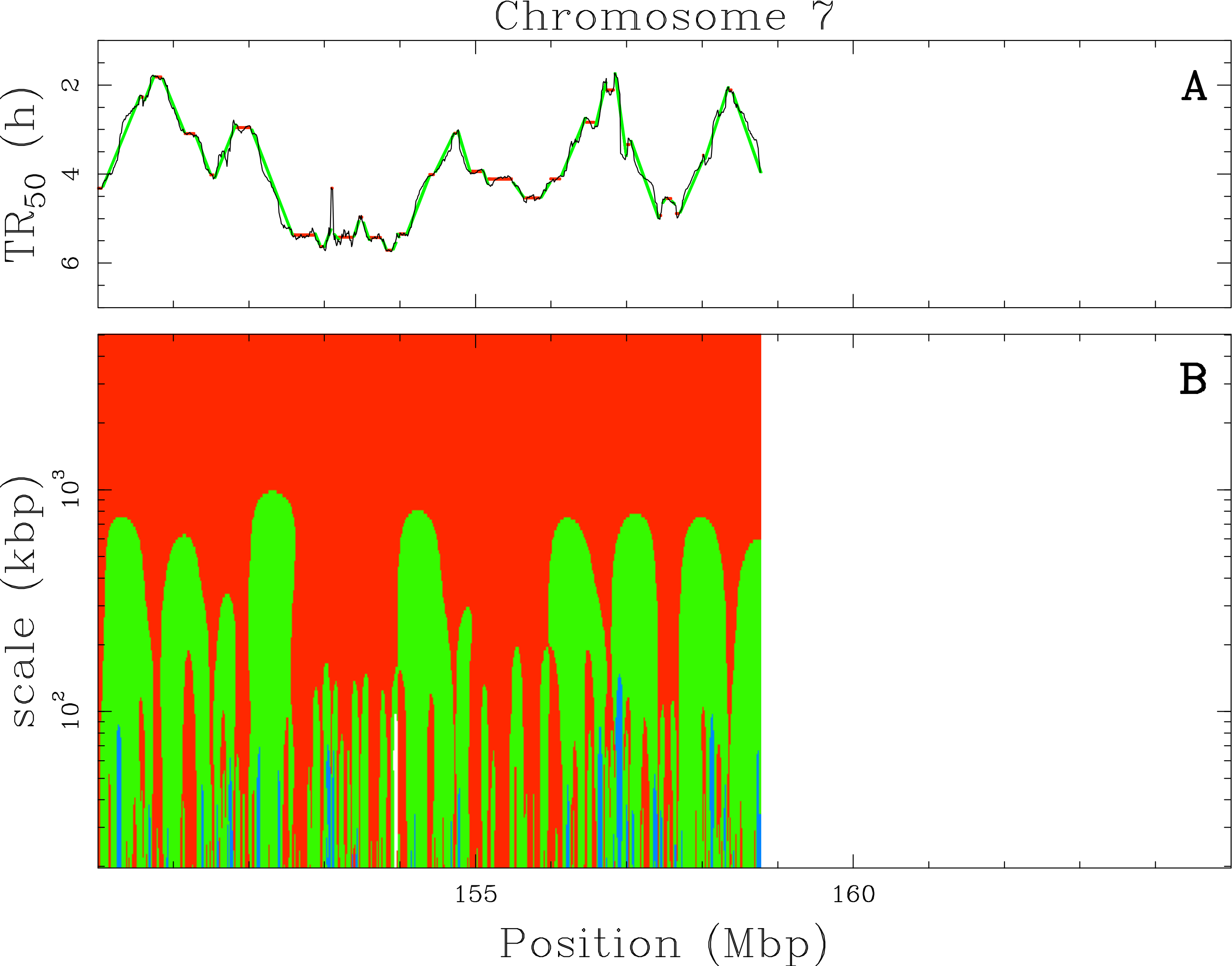
Chromosome 7
TR50 (h)
2
4
6
A
scale (kbp)
10^3
10^2
B
155
160
Position (Mbp)

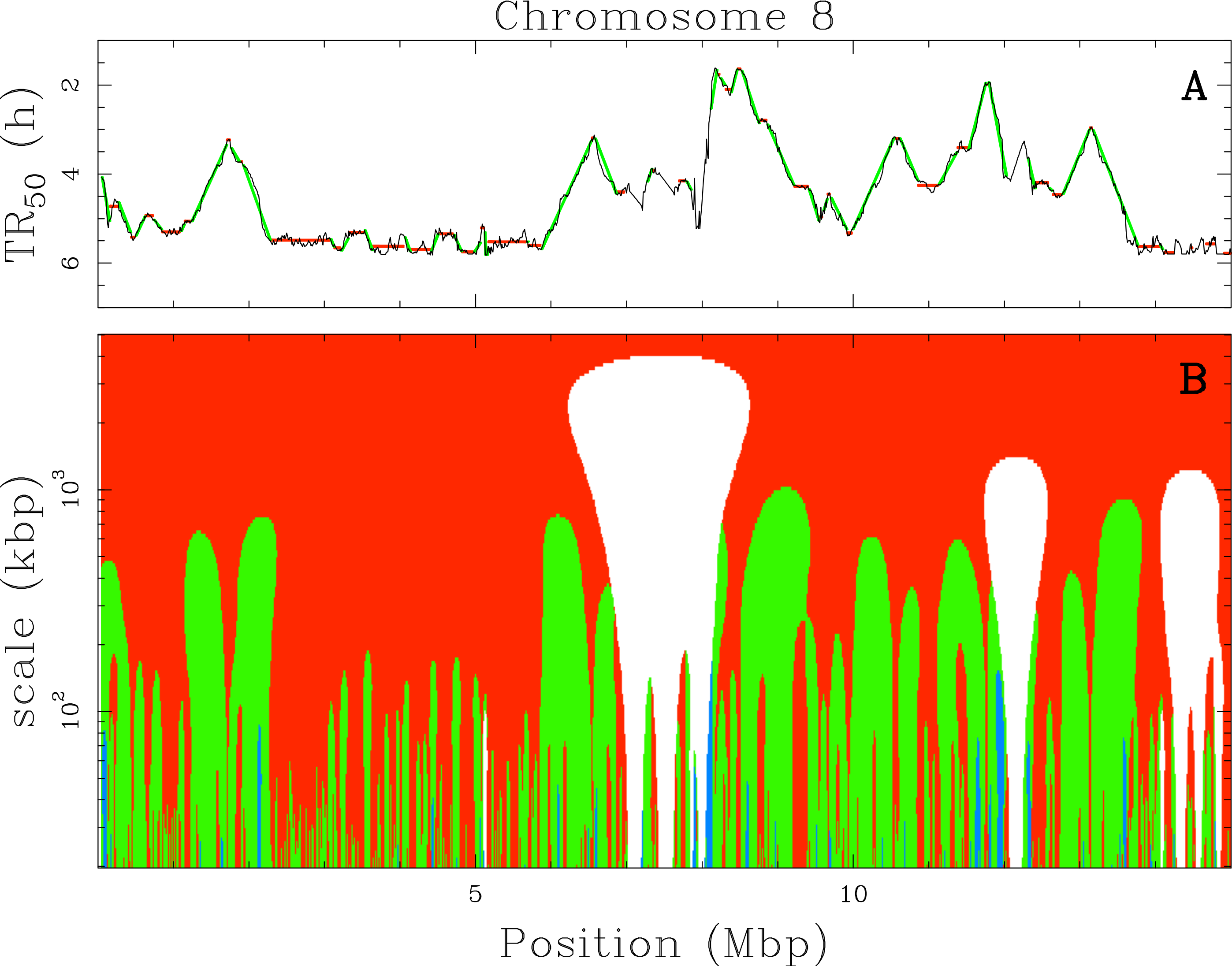

Chromosome 8
TR50 (h)
2
4
6
A
B
scale (kbp)
10^3
10^2
5
10
Position (Mbp)

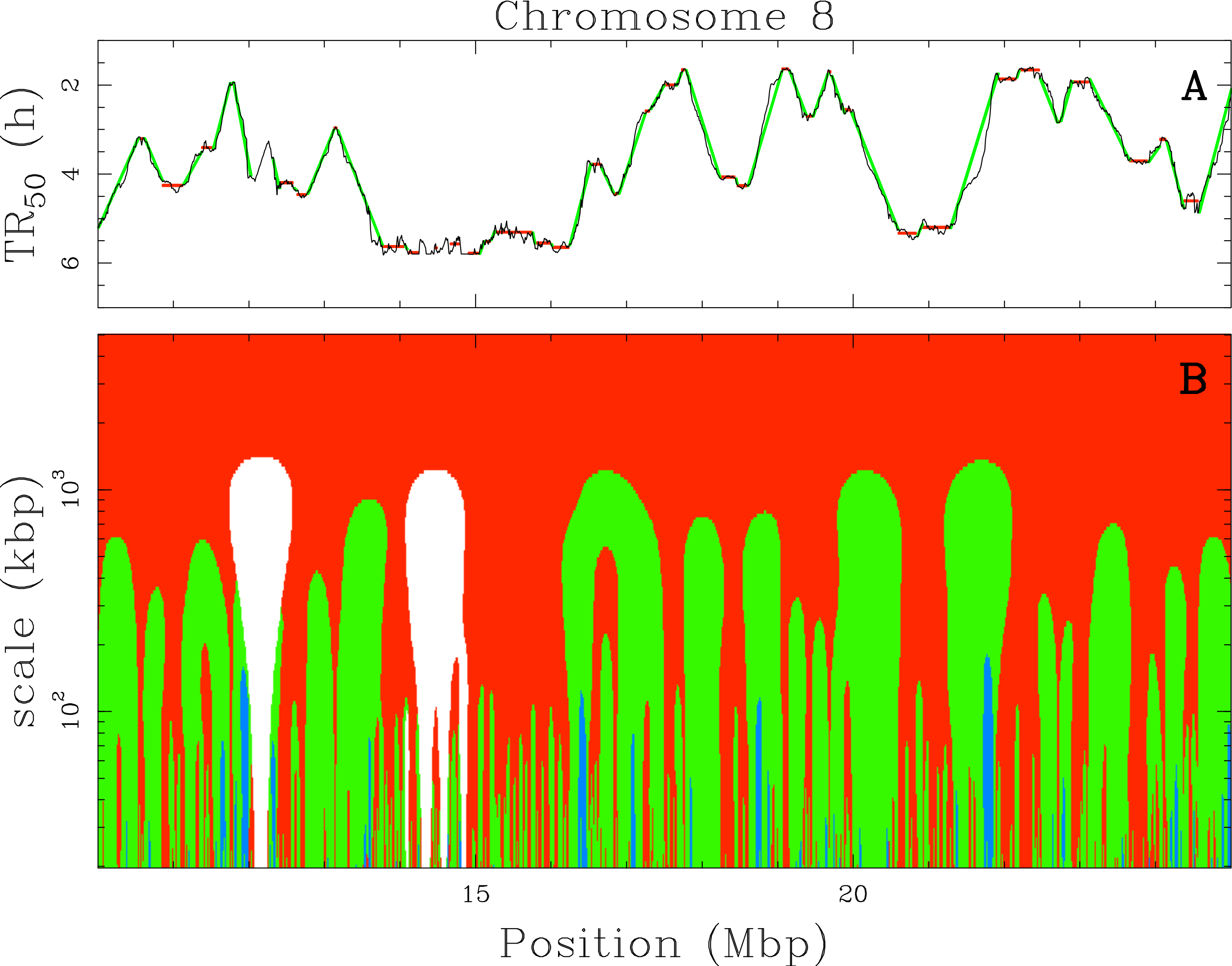

Chromosome 8
A
B
TR50 (h)
2
4
6
scale (kbp)
10^3
10^2
15
20
Position (Mbp)

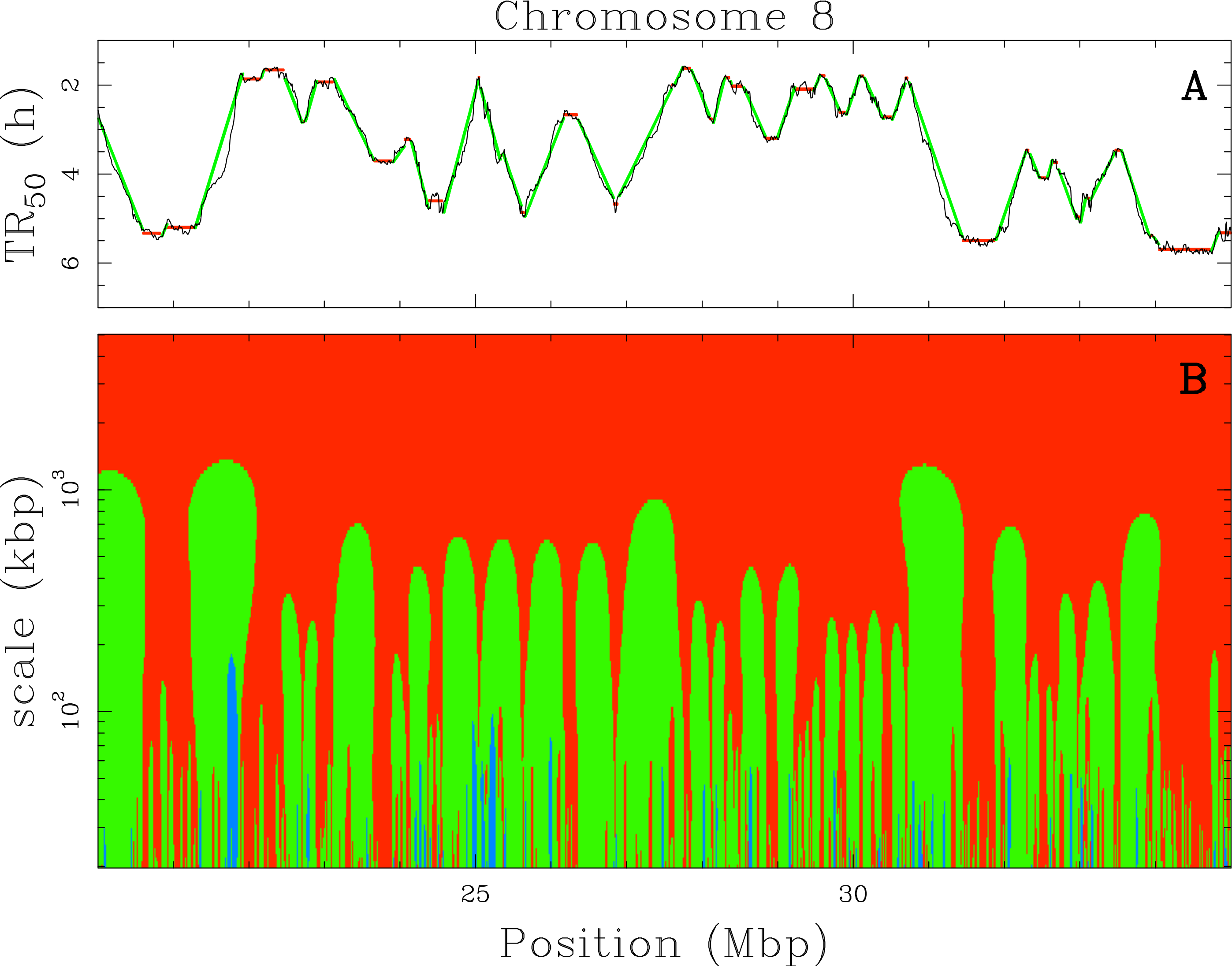
Chromosome 8
A
B
TR50 (h)
2
4
6
scale (kbp)
10^3
10^2
25
30
Position (Mbp)

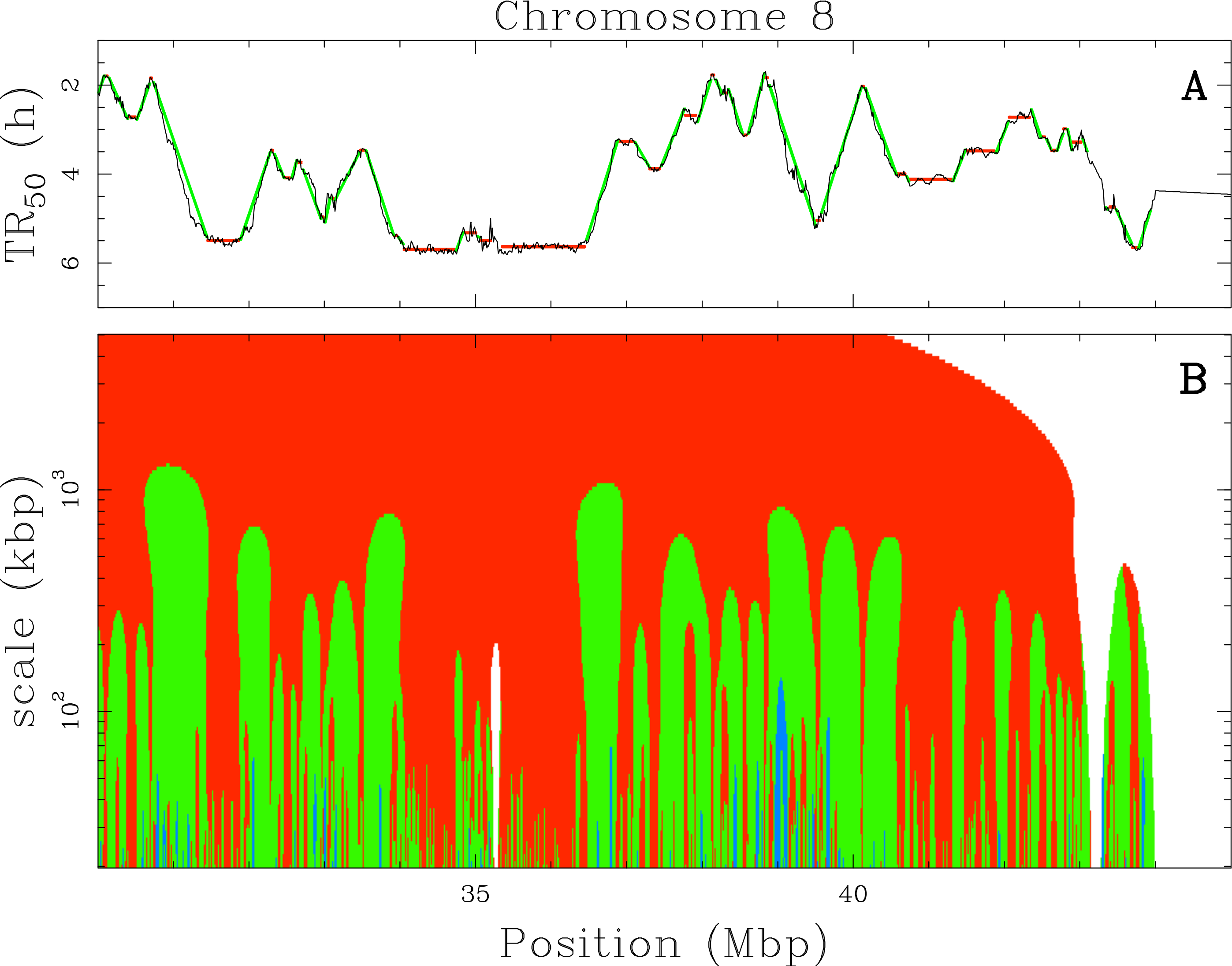

Chromosome 8
A
B
TR50 (h)
2
4
6
scale (kbp)
10^3
10^2
35
40
Position (Mbp)

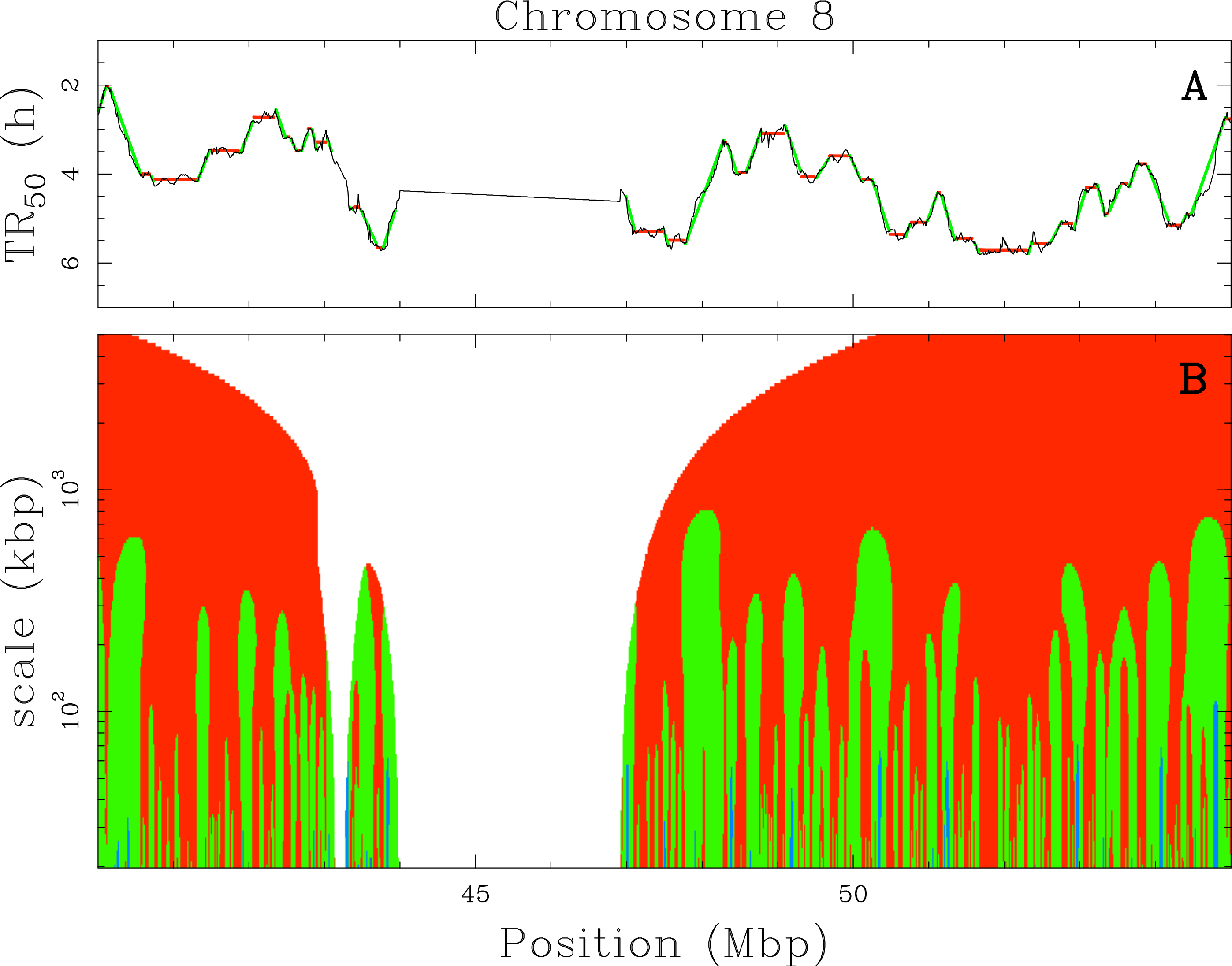

Chromosome 8
A
B
TR$_{50}$ (h)
2
4
6
scale (kbp)
10^3
10^2
45
50
Position (Mbp)

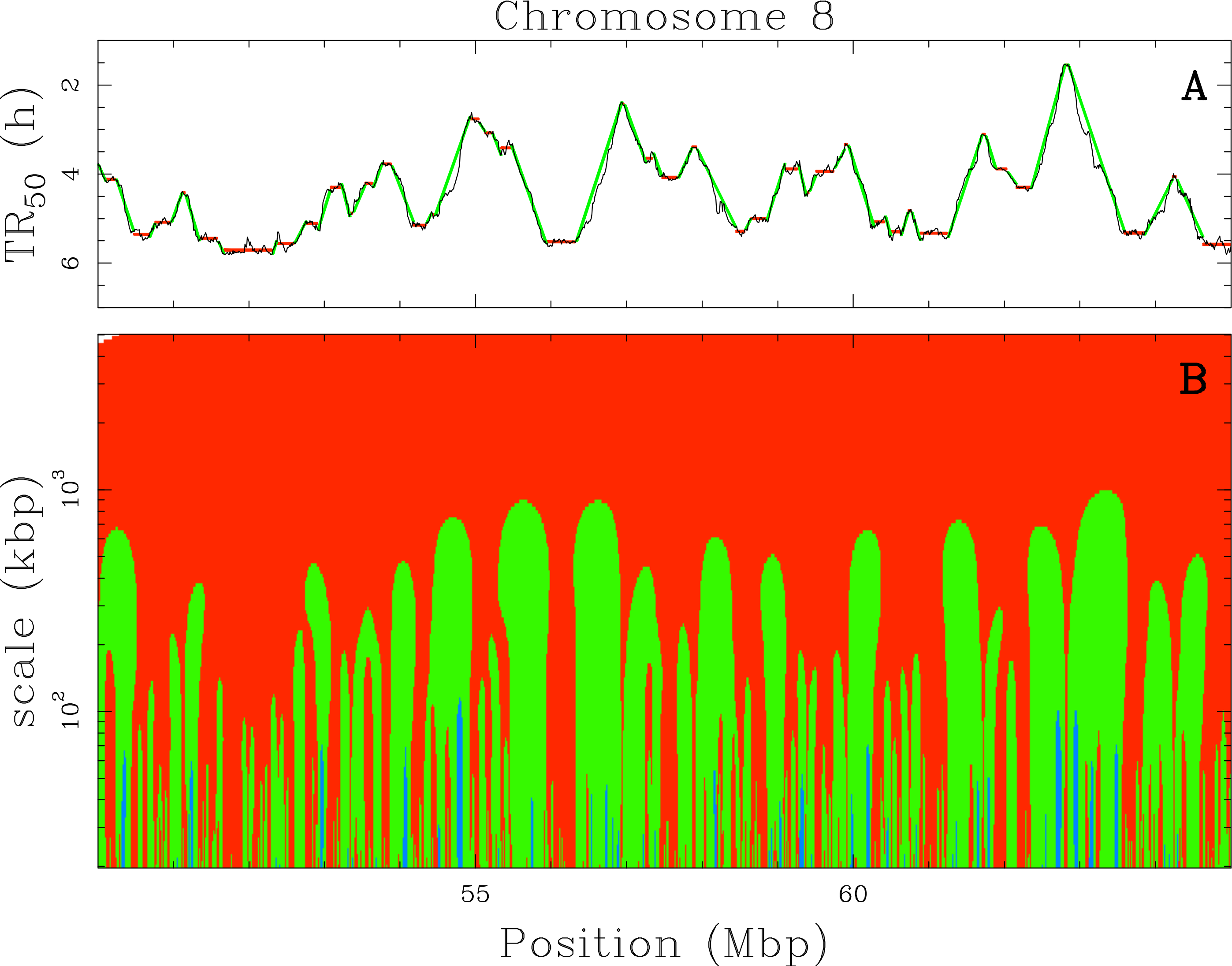

Chromosome 8
A
B
TR50 (h)
2
4
6
scale (kbp)
10^3
10^2
55
60
Position (Mbp)

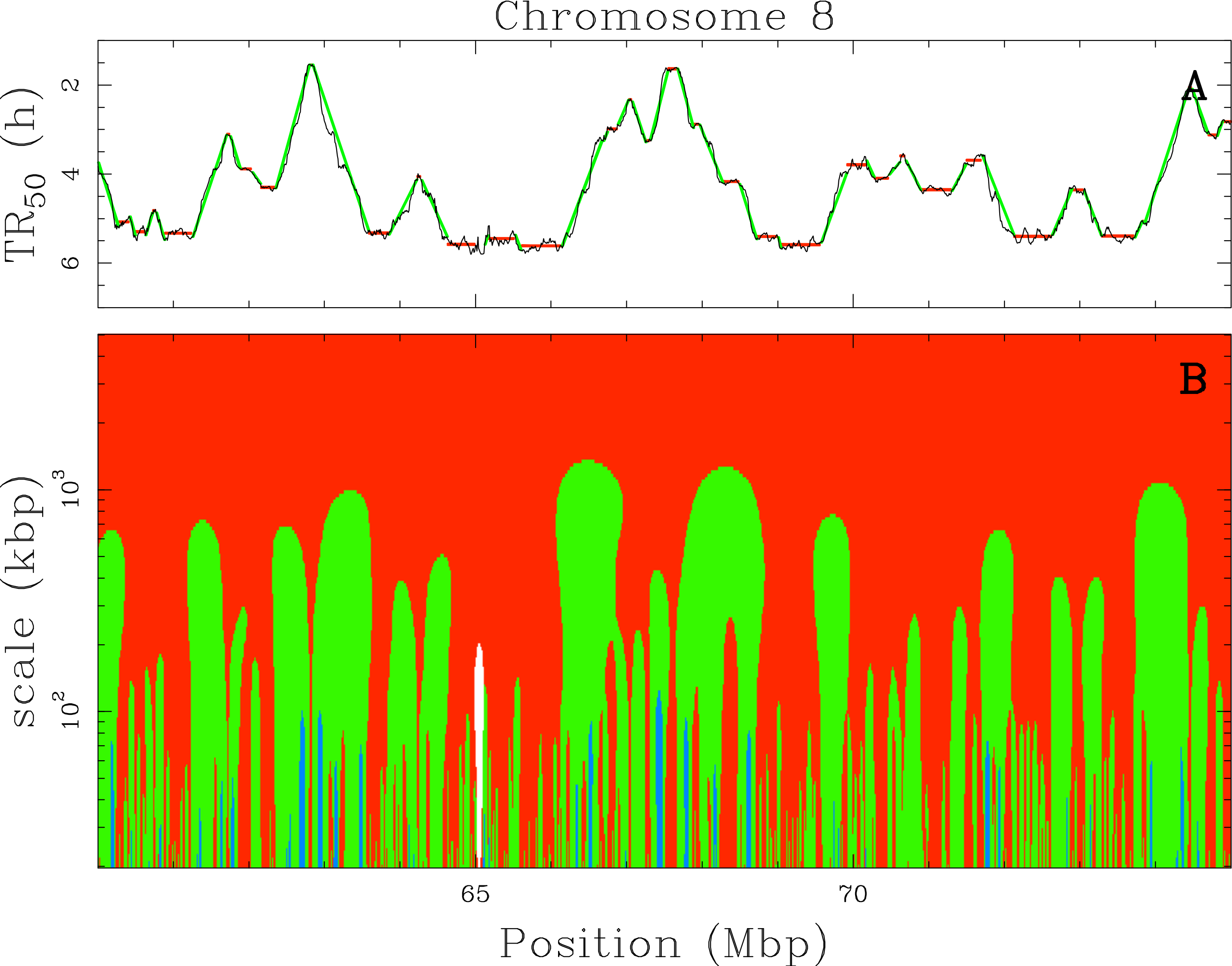

Chromosome 8
A
B
TR_{50} (h)
2
4
6
scale (kbp)
10^3
10^2
65
70
Position (Mbp)

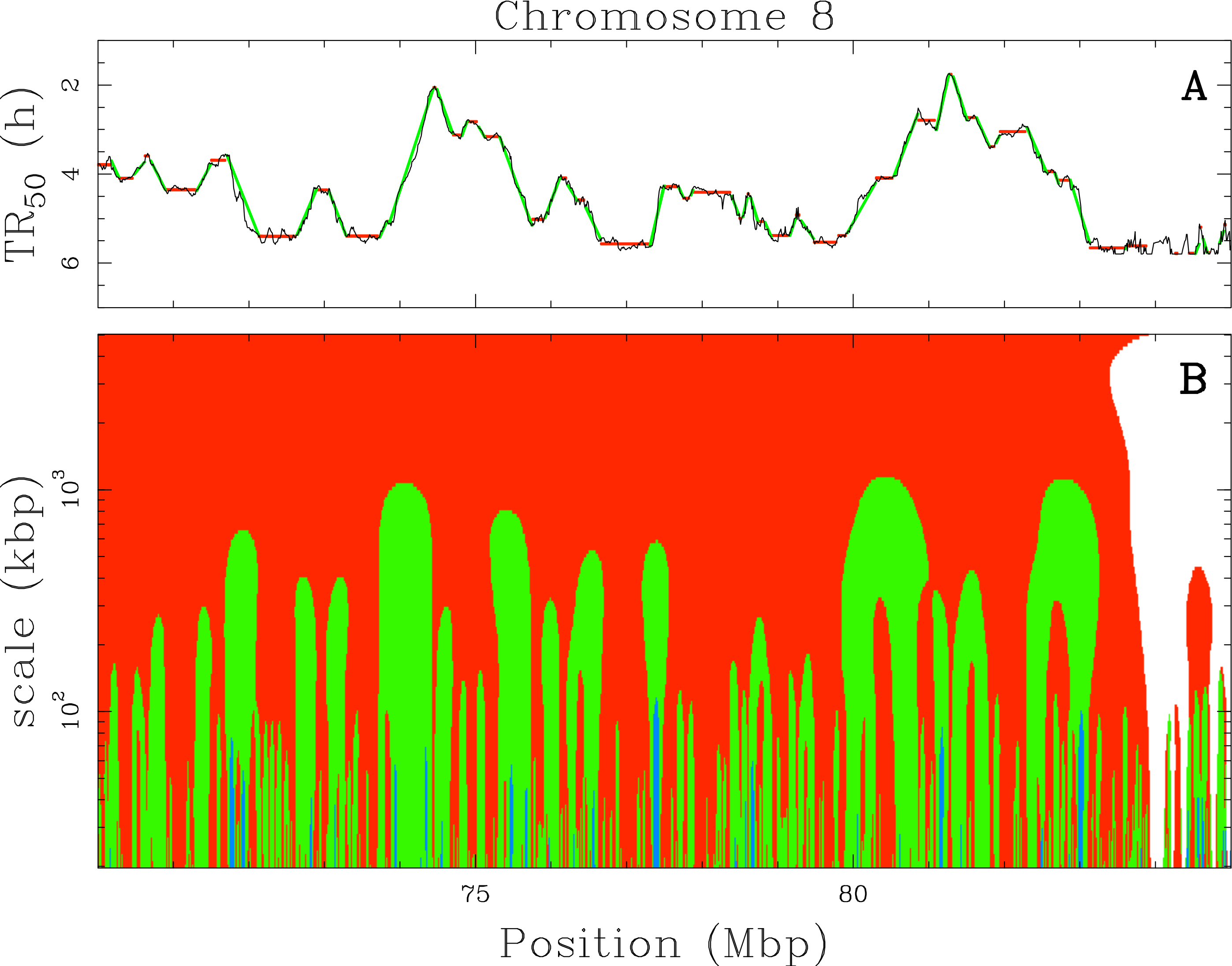

Chromosome 8
A
B
TR_{50} (h)
2
4
6
scale (kbp)
10^3
10^2
75
80
Position (Mbp)

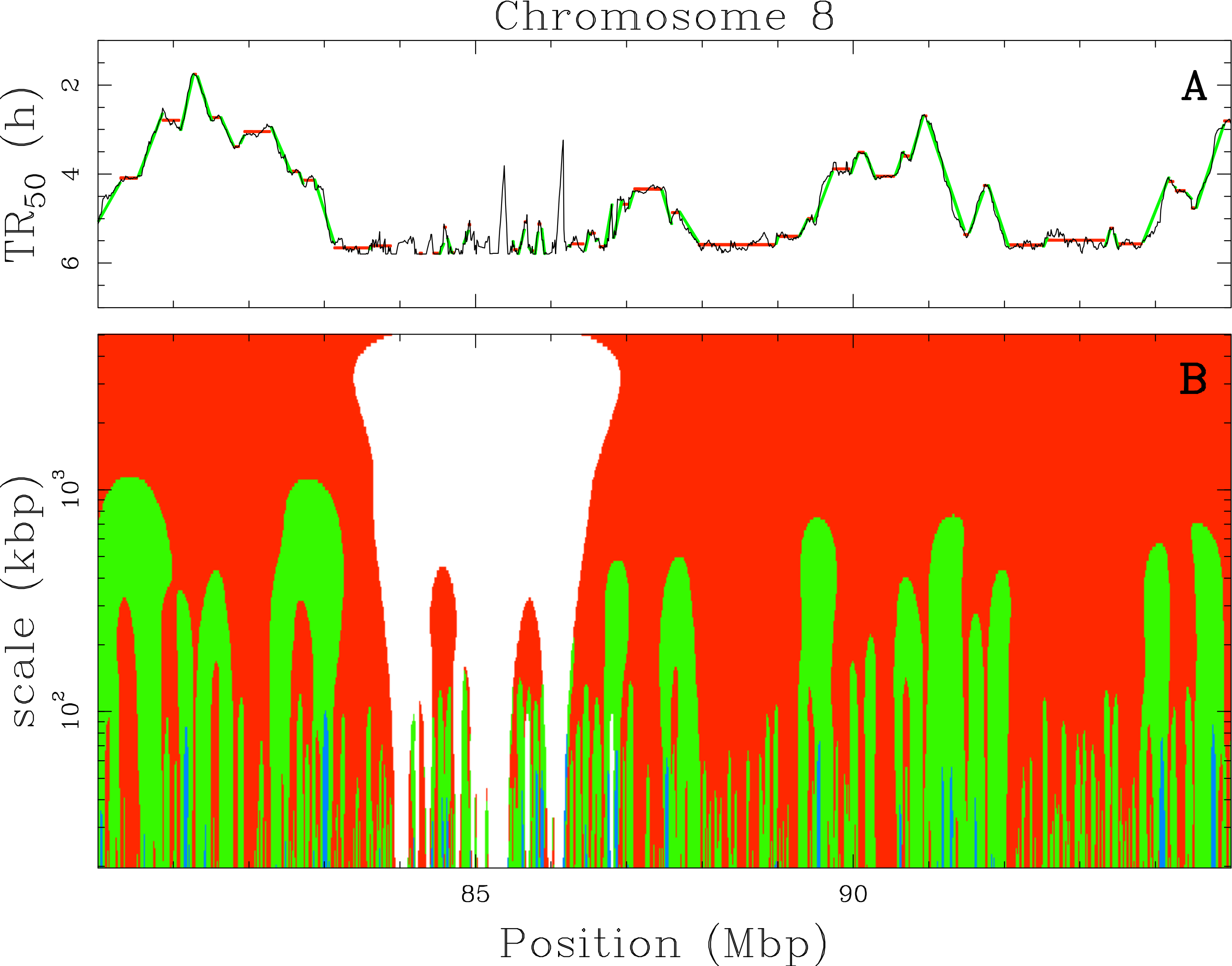

Chromosome 8
A
B
TR50 (h)
2
4
6
scale (kbp)
10^3
10^2
85
90
Position (Mbp)

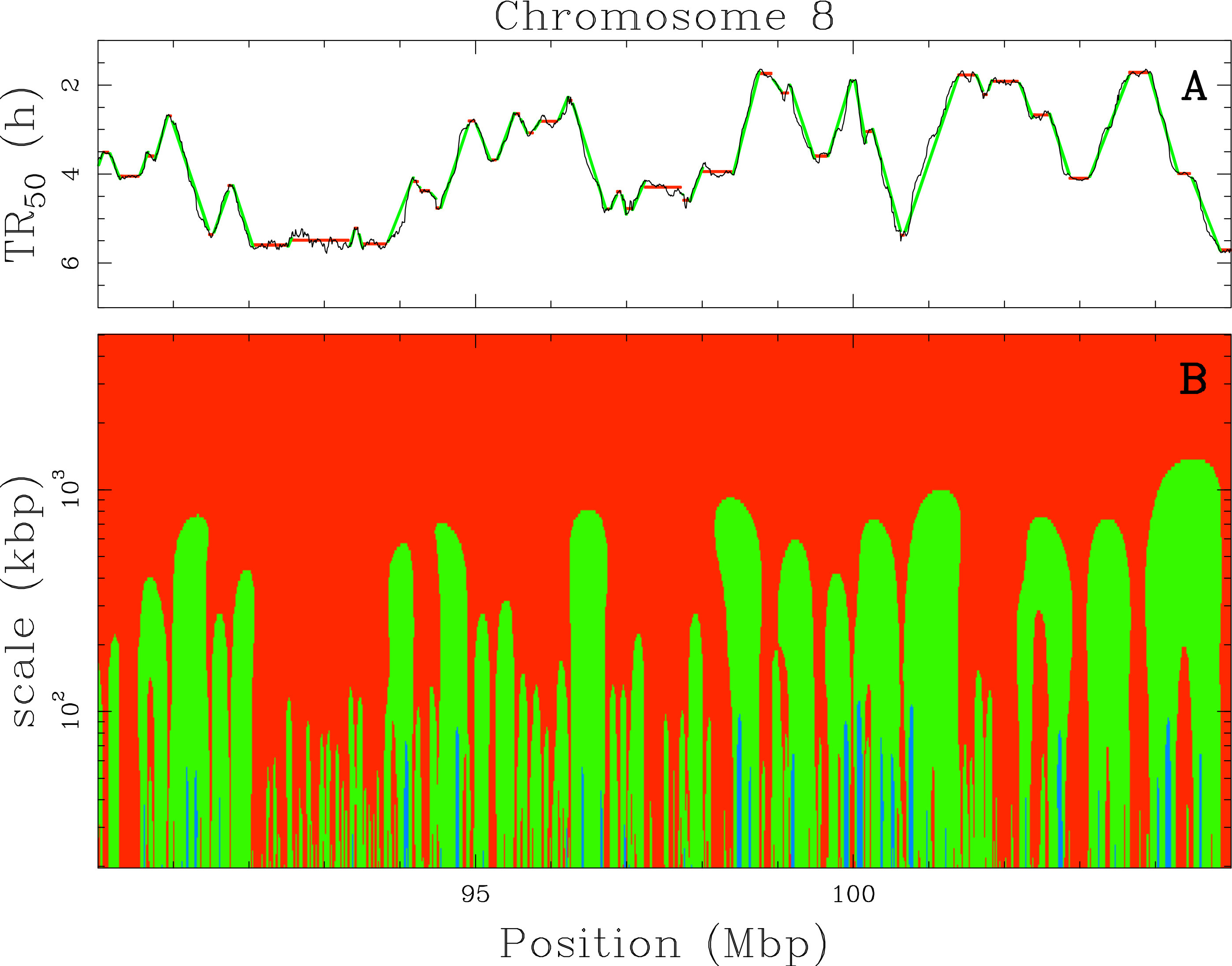
Chromosome 8
A
B
TR$_{50}$ (h)
2
4
6
scale (kbp)
10^3
10^2
95
100
Position (Mbp)

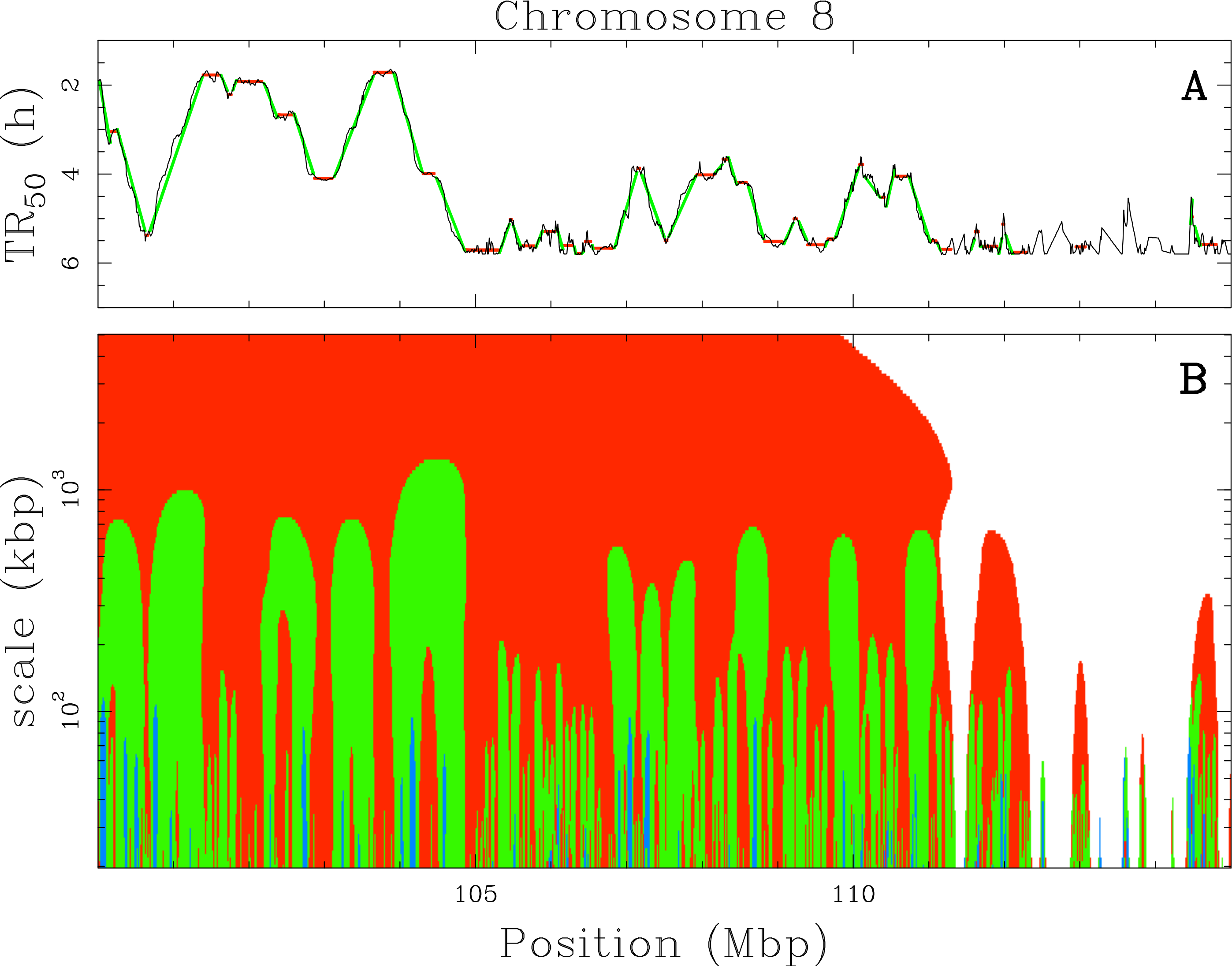

Chromosome 8
A
B
TR50 (h)
2
4
6
scale (kbp)
10^3
10^2
105
110
Position (Mbp)

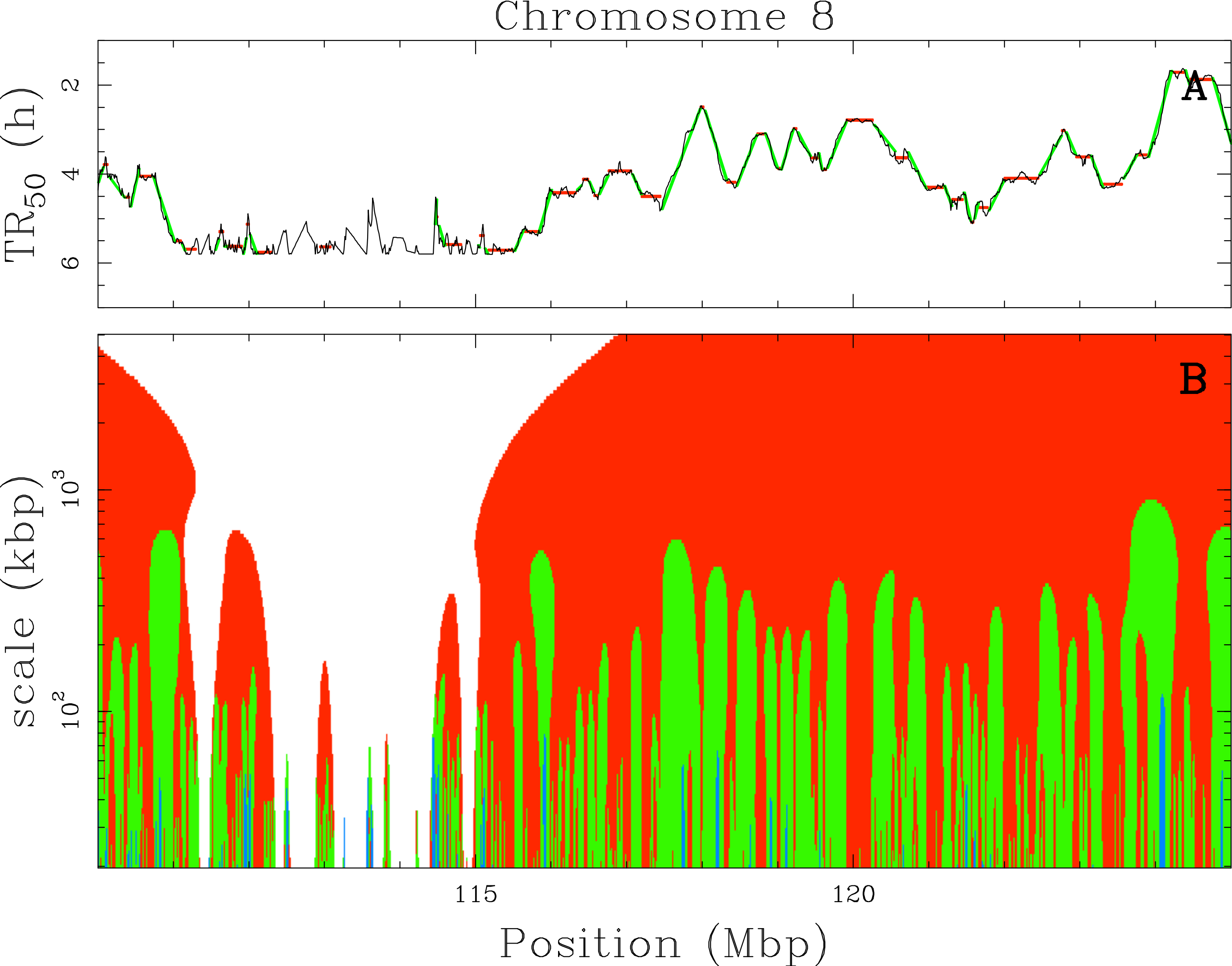

Chromosome 8
TR_{50} (h)
2
4
6
A
B
scale (kbp)
10^3
10^2
115
120
Position (Mbp)

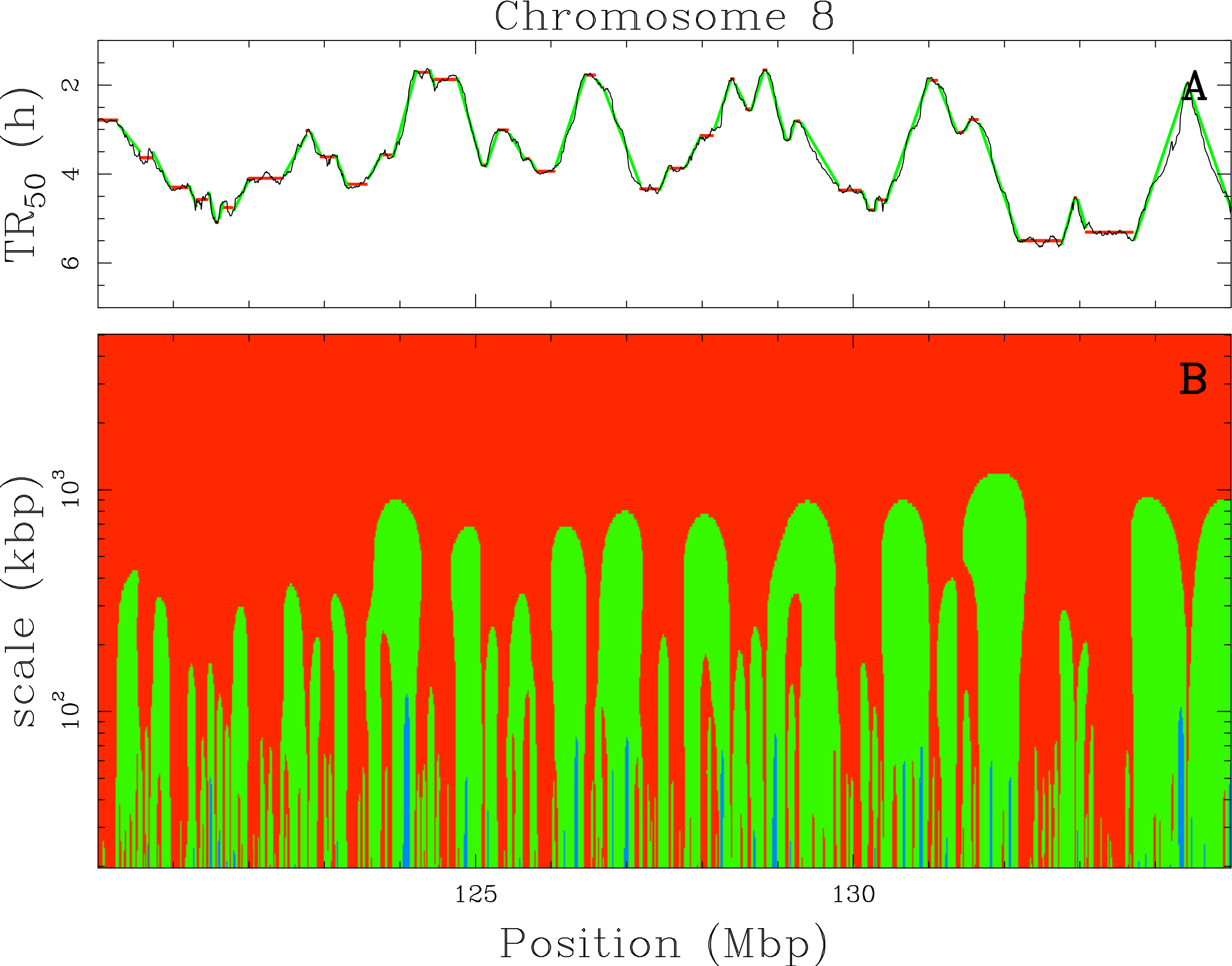

Chromosome 8
A
B
TR50 (h)
2
4
6
scale (kbp)
10^3
10^2
125
130
Position (Mbp)

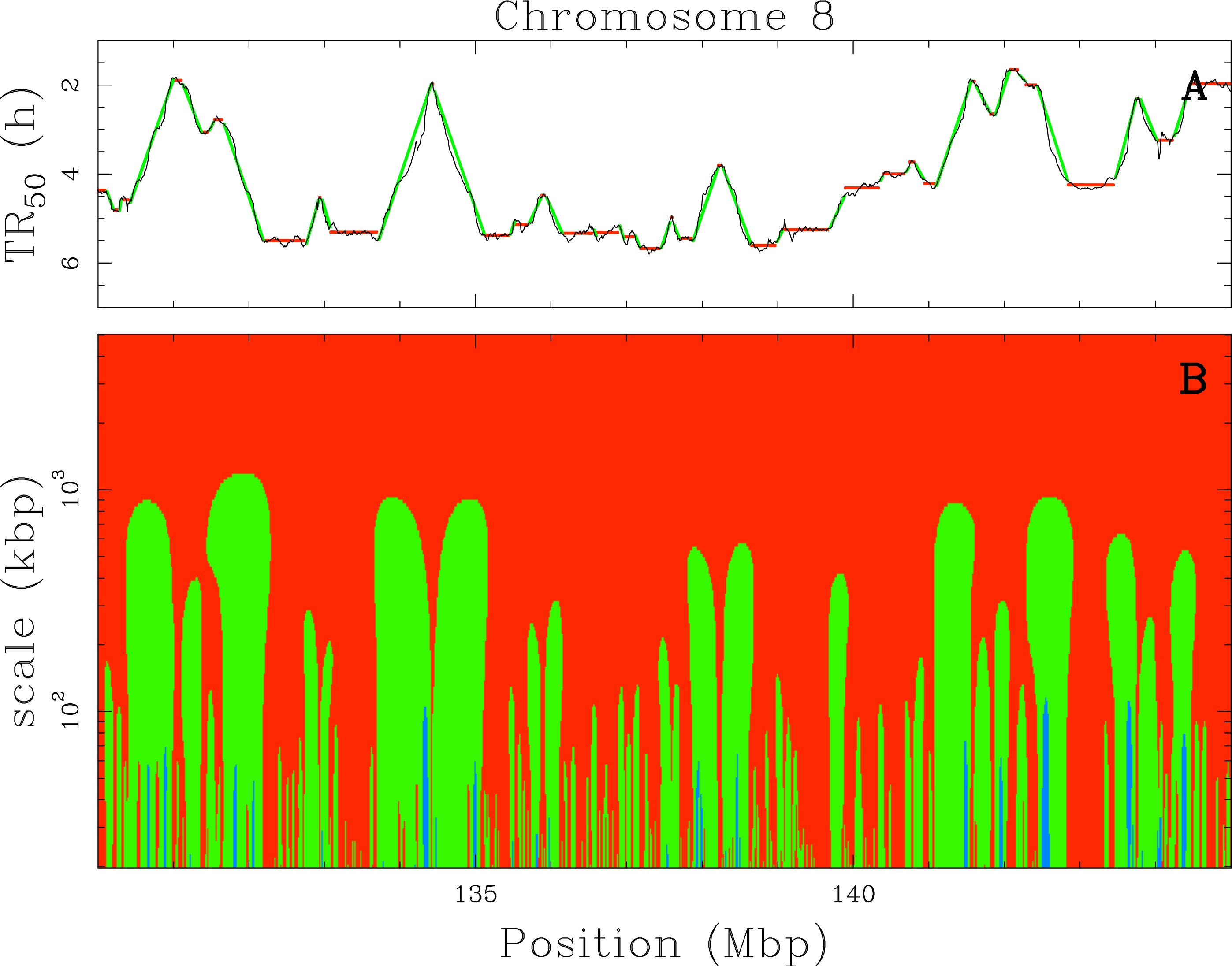

Chromosome 8
A
B
TR50 (h)
2
4
6
scale (kbp)
10^3
10^2
135
140
Position (Mbp)

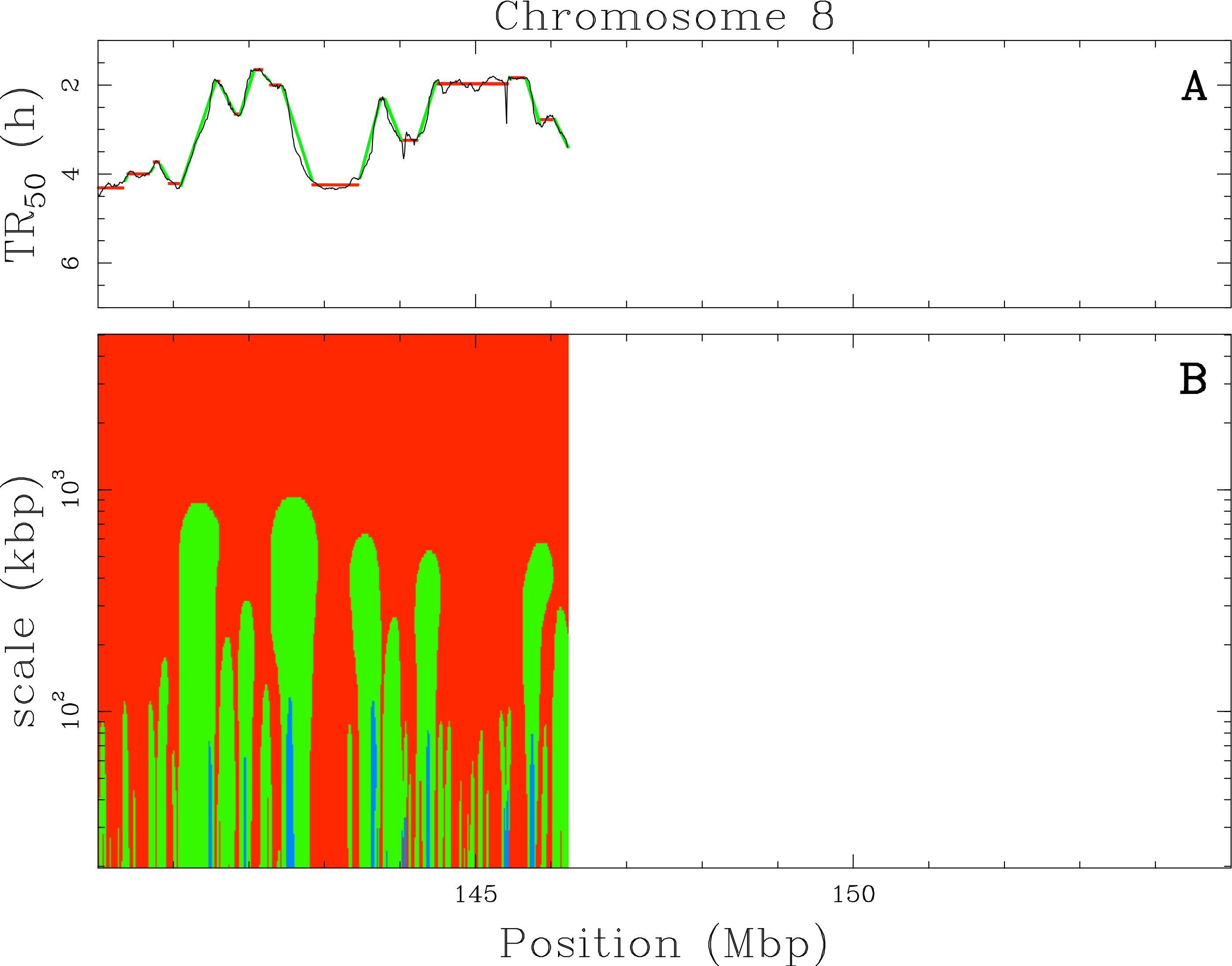

Chromosome 8
A
B
TR50 (h)
2
4
6
scale (kbp)
10^3
10^2
145
150
Position (Mbp)

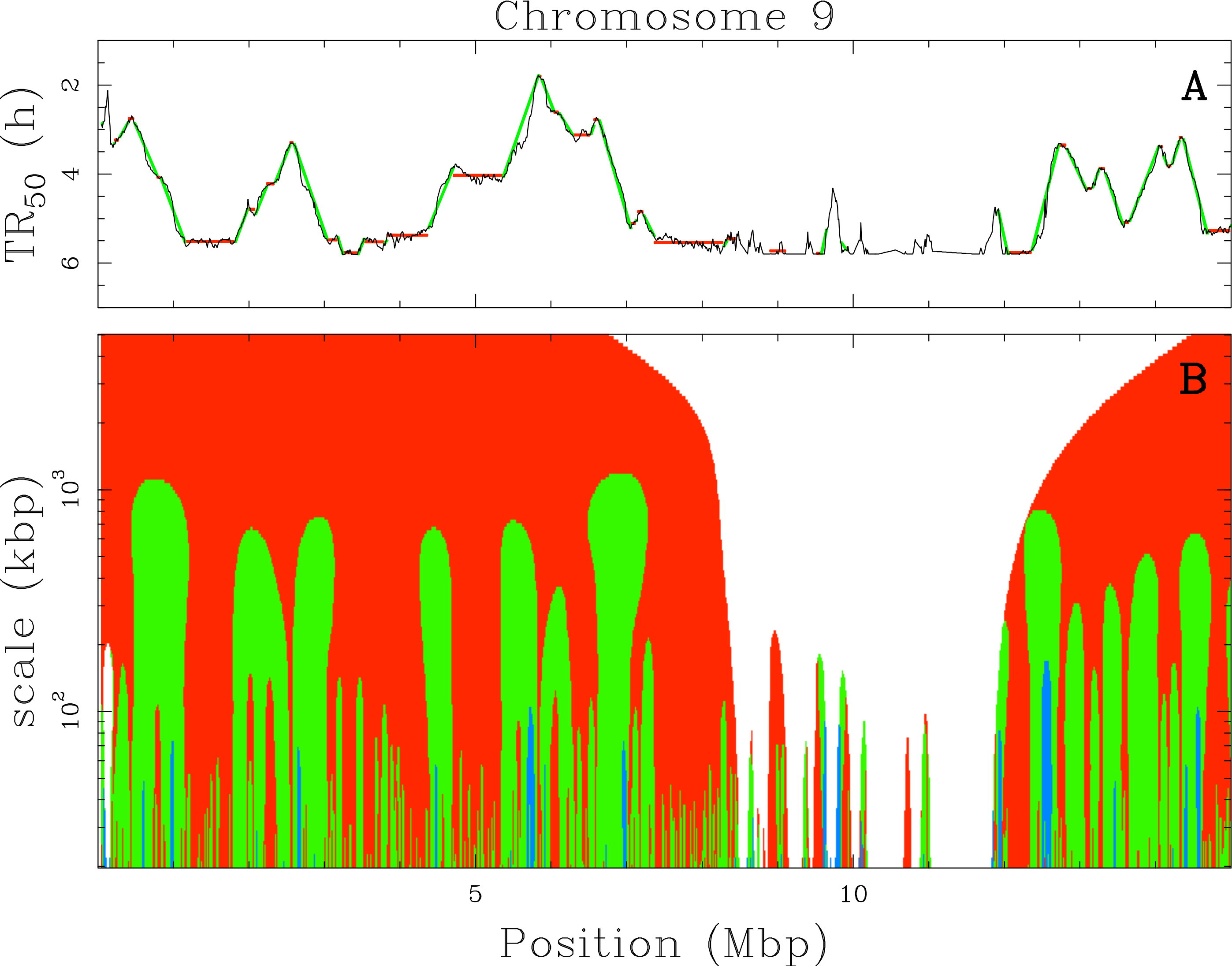

Chromosome 9
A
B
TR_{50} (h)
2
4
6
scale (kbp)
10^3
10^2
5
10
Position (Mbp)

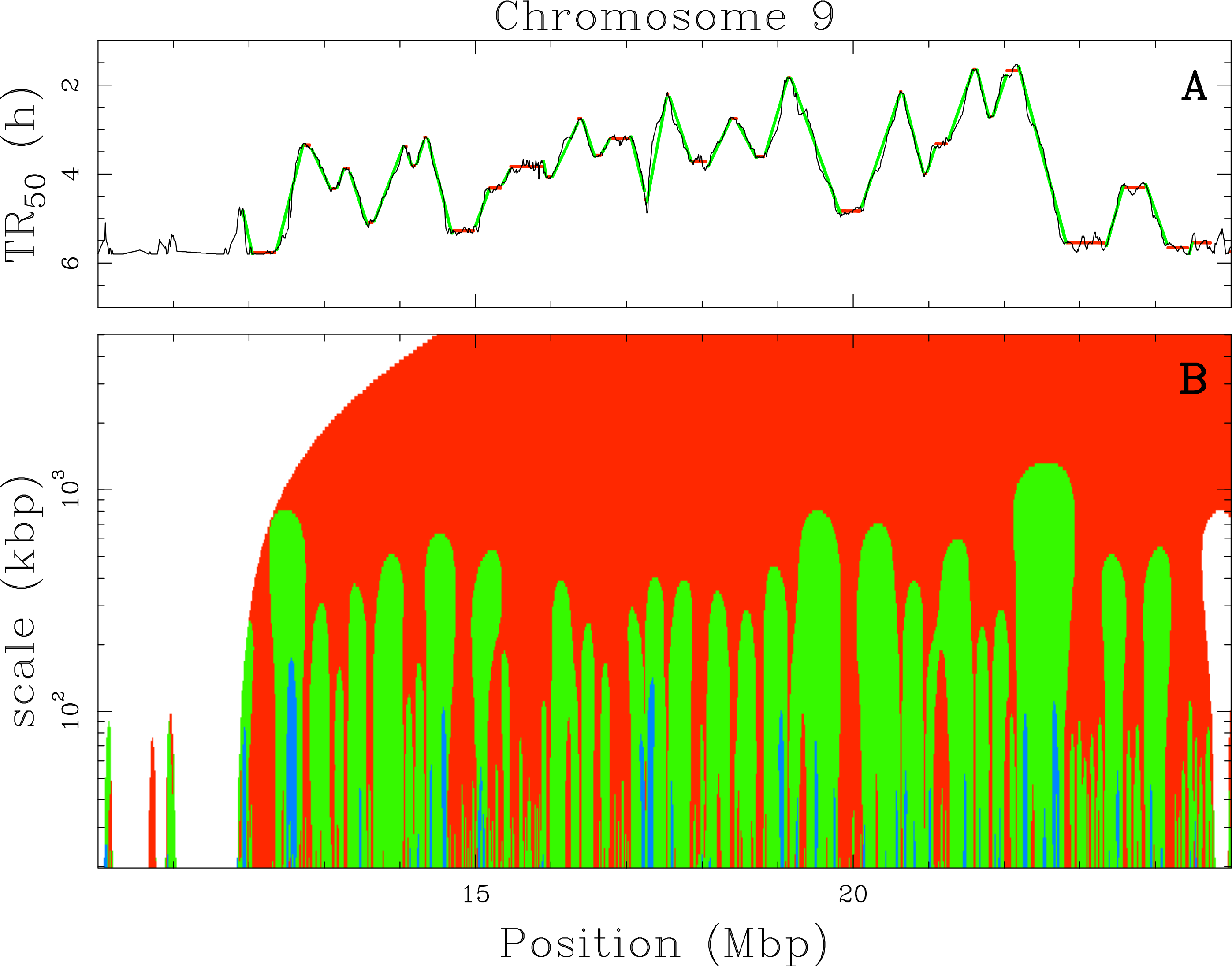

Chromosome 9
A
TR_{50} (h)
2
4
6
B
scale (kbp)
10^3
10^2
15
20
Position (Mbp)

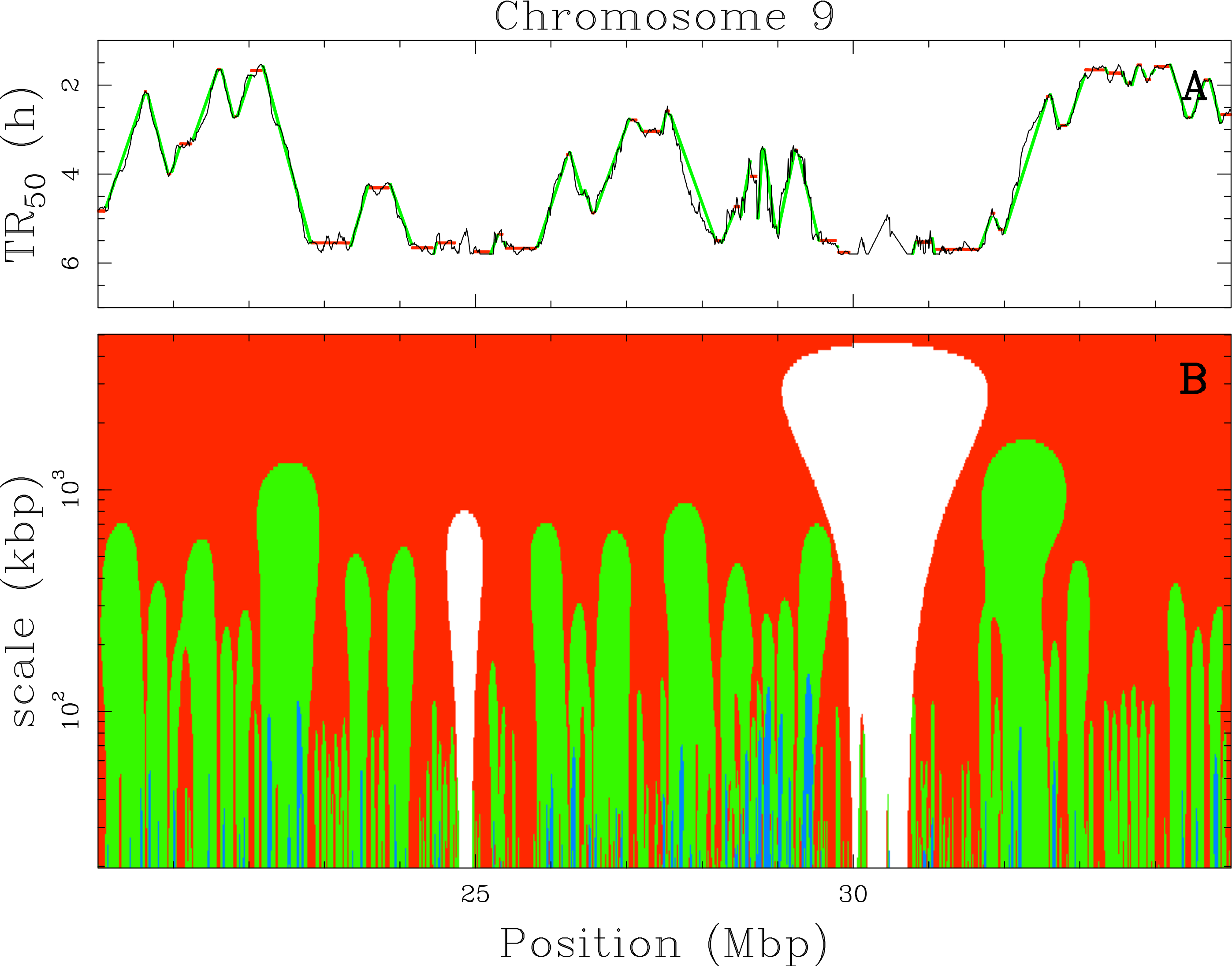

Chromosome 9
A
B
TR50 (h)
2
4
6
scale (kbp)
10^3
10^2
25
30
Position (Mbp)

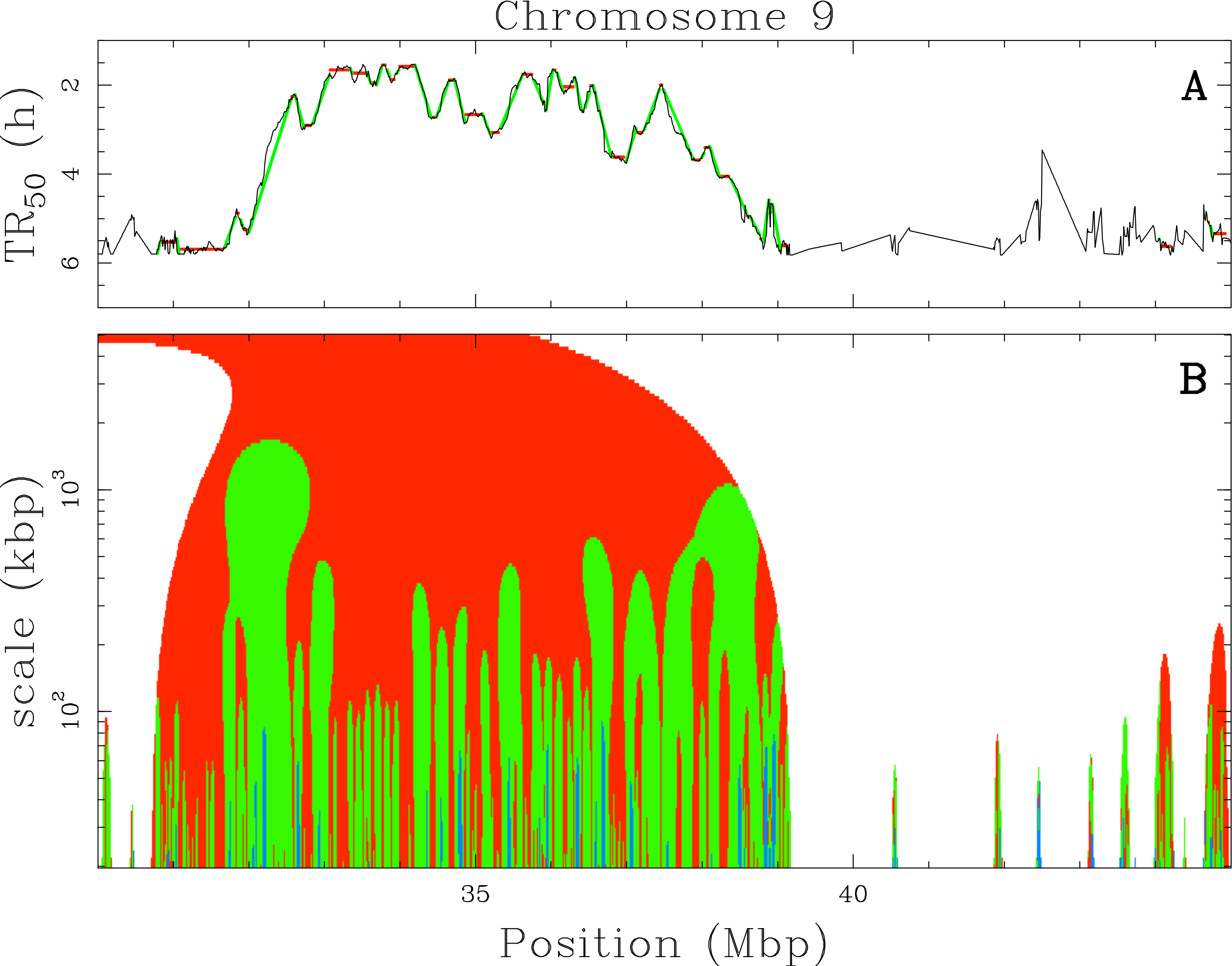
Chromosome 9
A
B
TR$_{50}$ (h)
2
4
6
scale (kbp)
10^3
10^2
35
40
Position (Mbp)

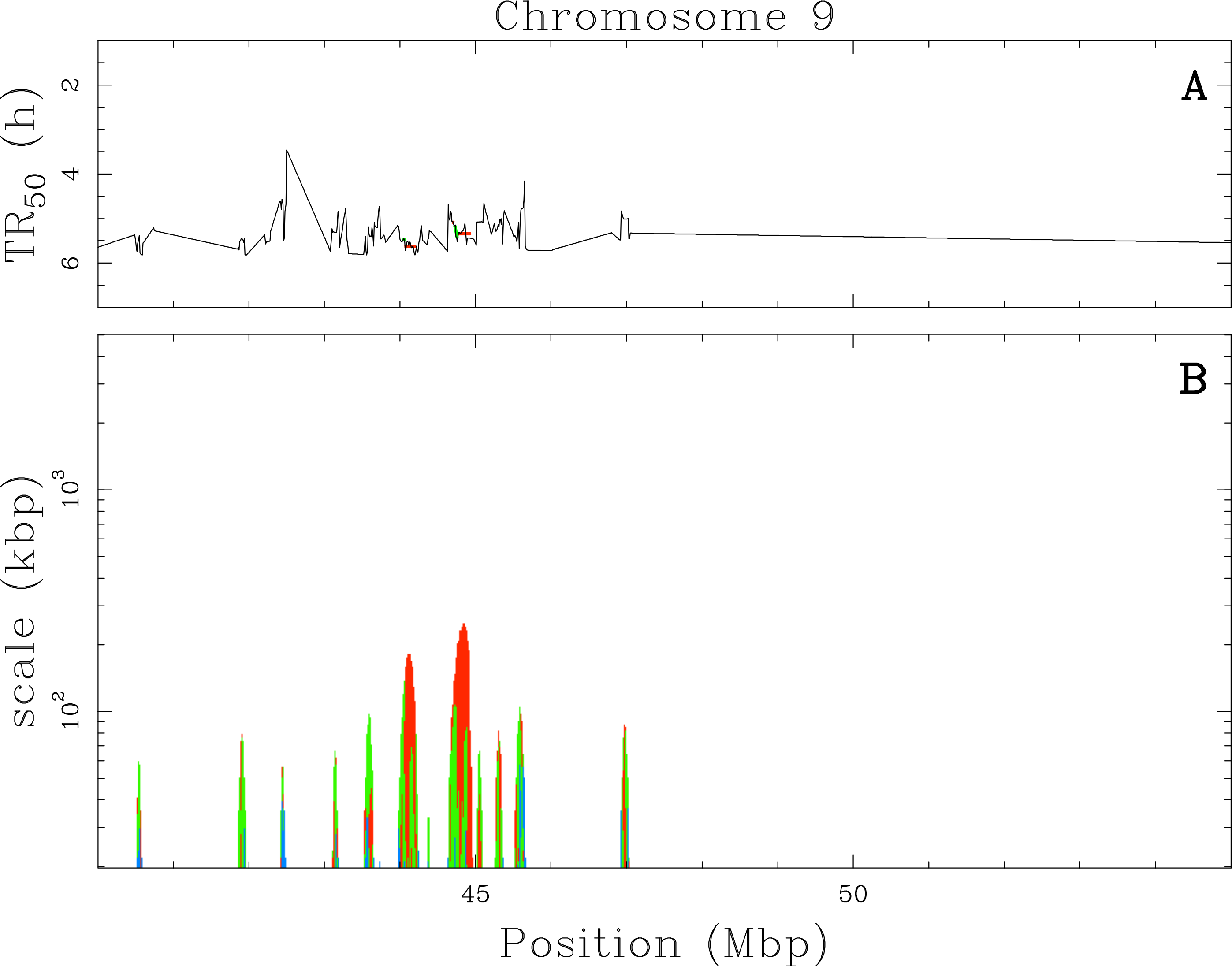

Chromosome 9
A
B
TR50 (h)
2
4
6
scale (kbp)
10^3
10^2
45
50
Position (Mbp)

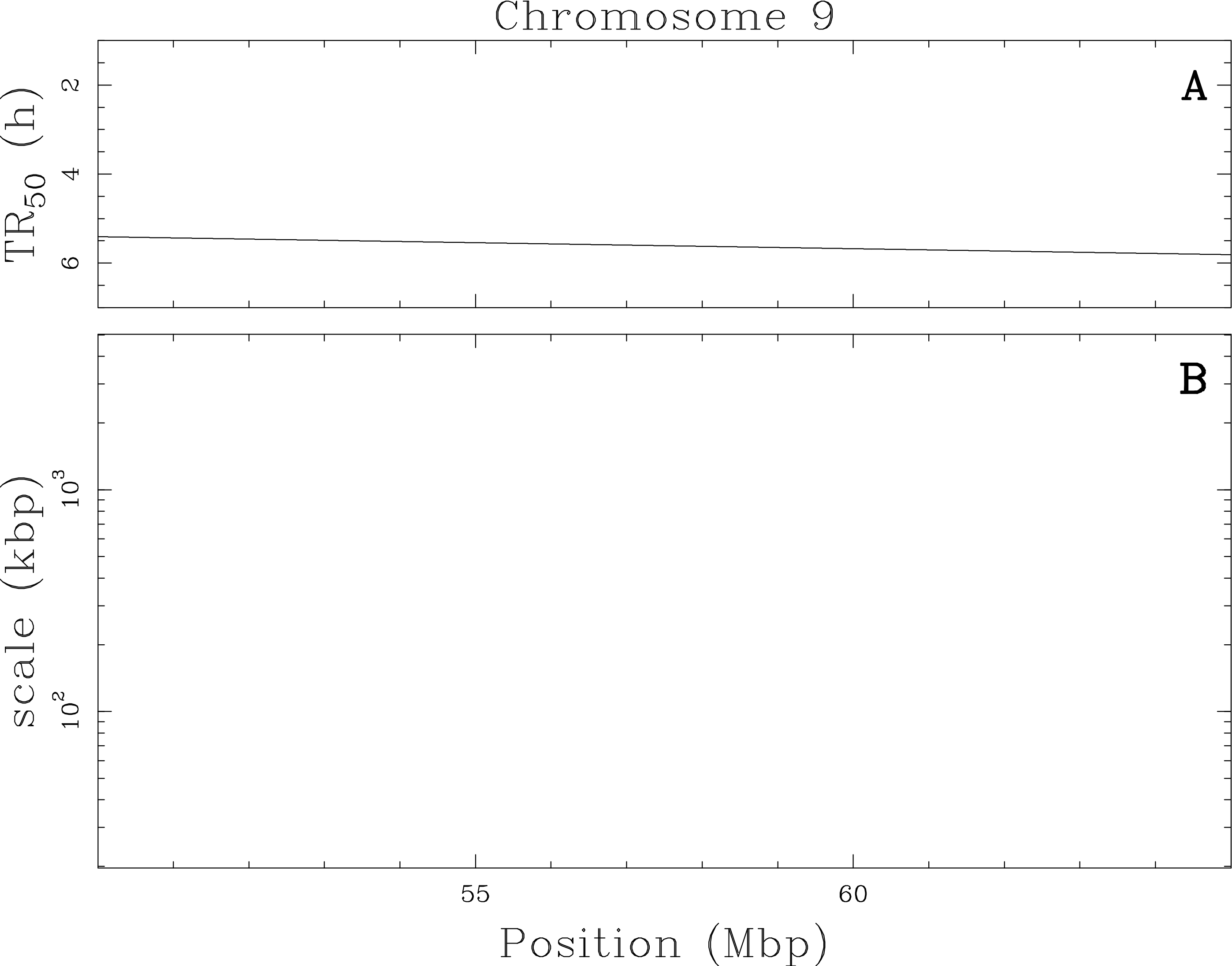

Chromosome 9
TR50 (h)
2
4
6
A
B
scale (kbp)
10^3
10^2
55
60
Position (Mbp)

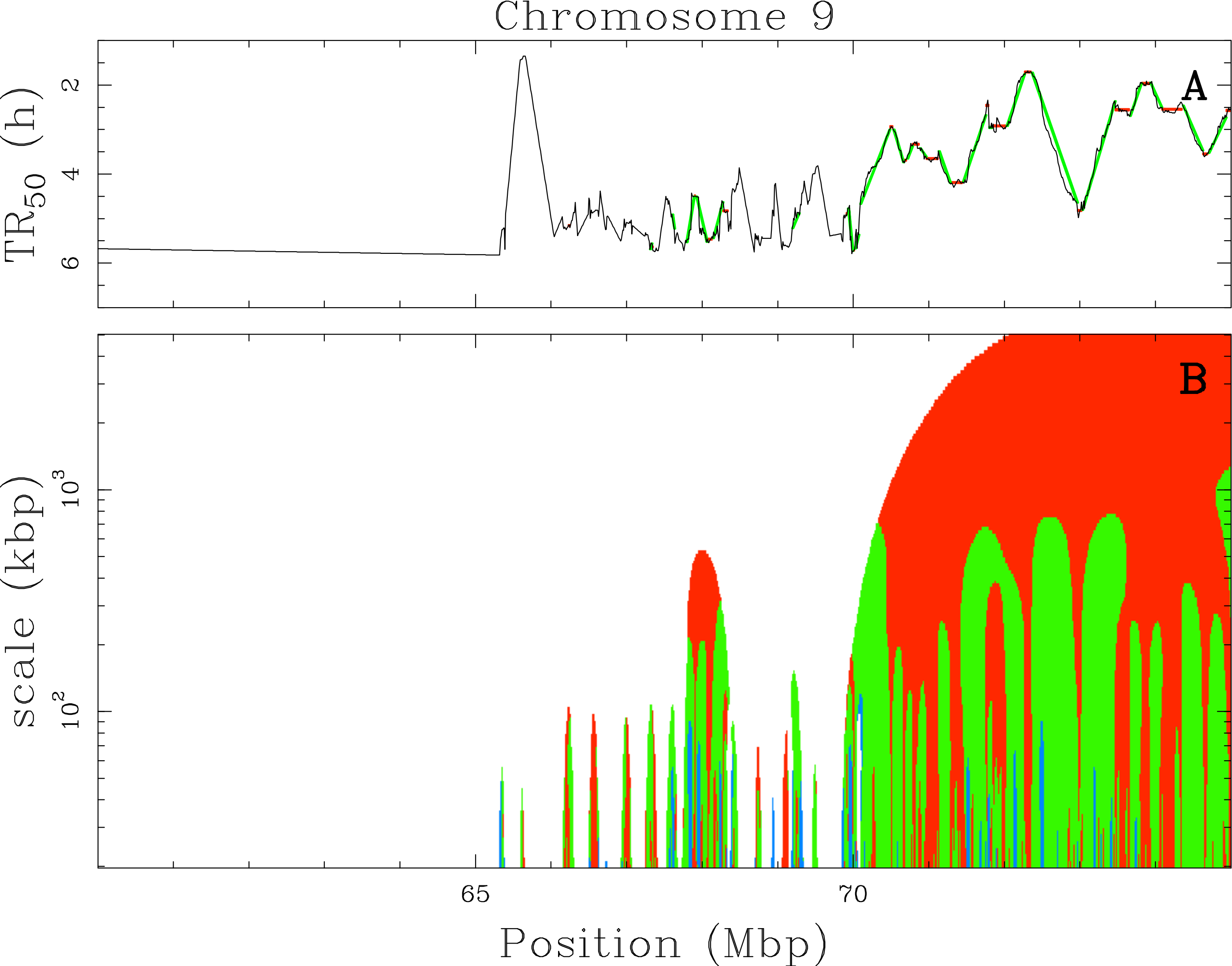

Chromosome 9
A
B
TR50 (h)
2
4
6
scale (kbp)
10^3
10^2
65
70
Position (Mbp)

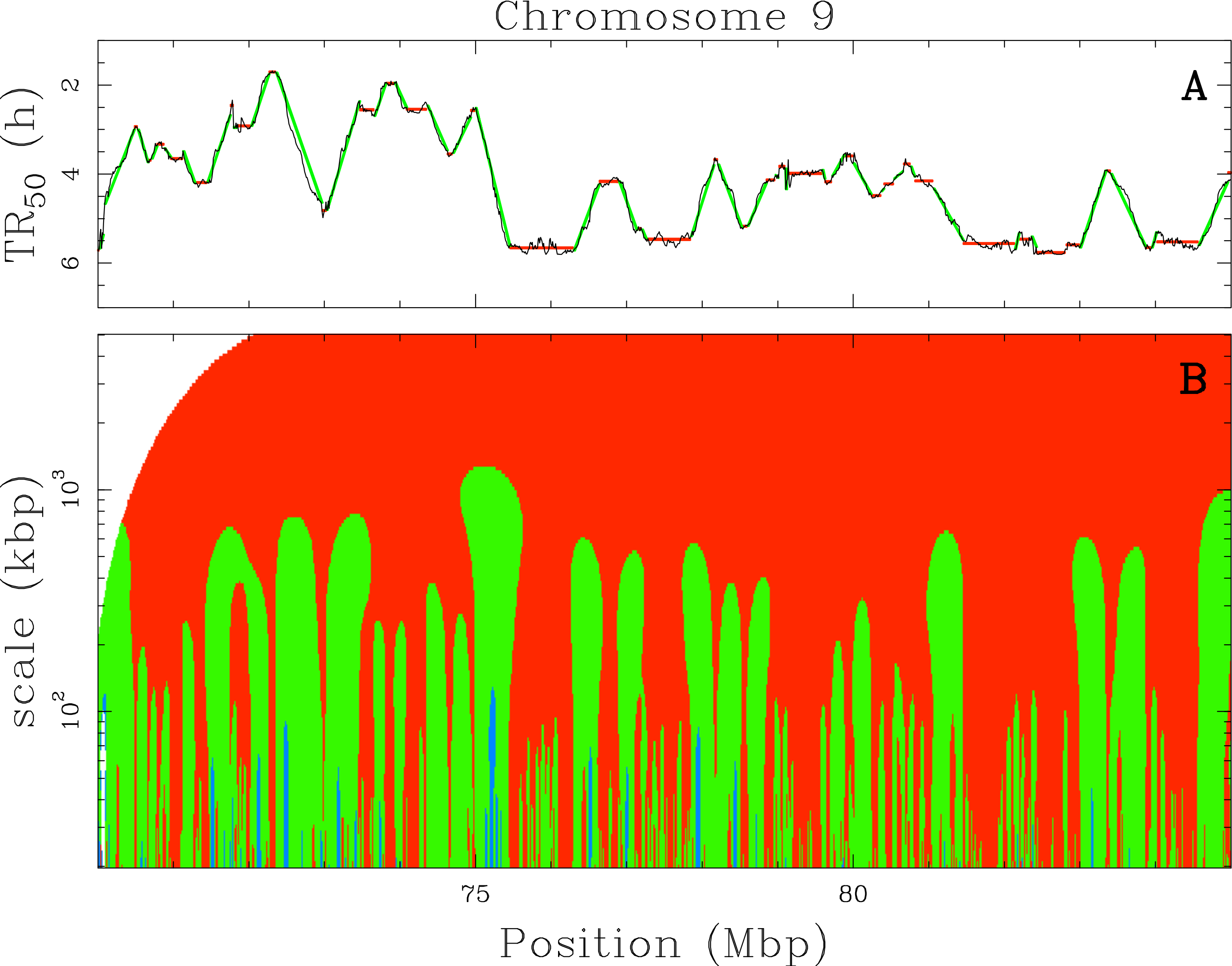

Chromosome 9
TR50 (h)
2
4
6
A
B
scale (kbp)
10^3
10^2
75
80
Position (Mbp)

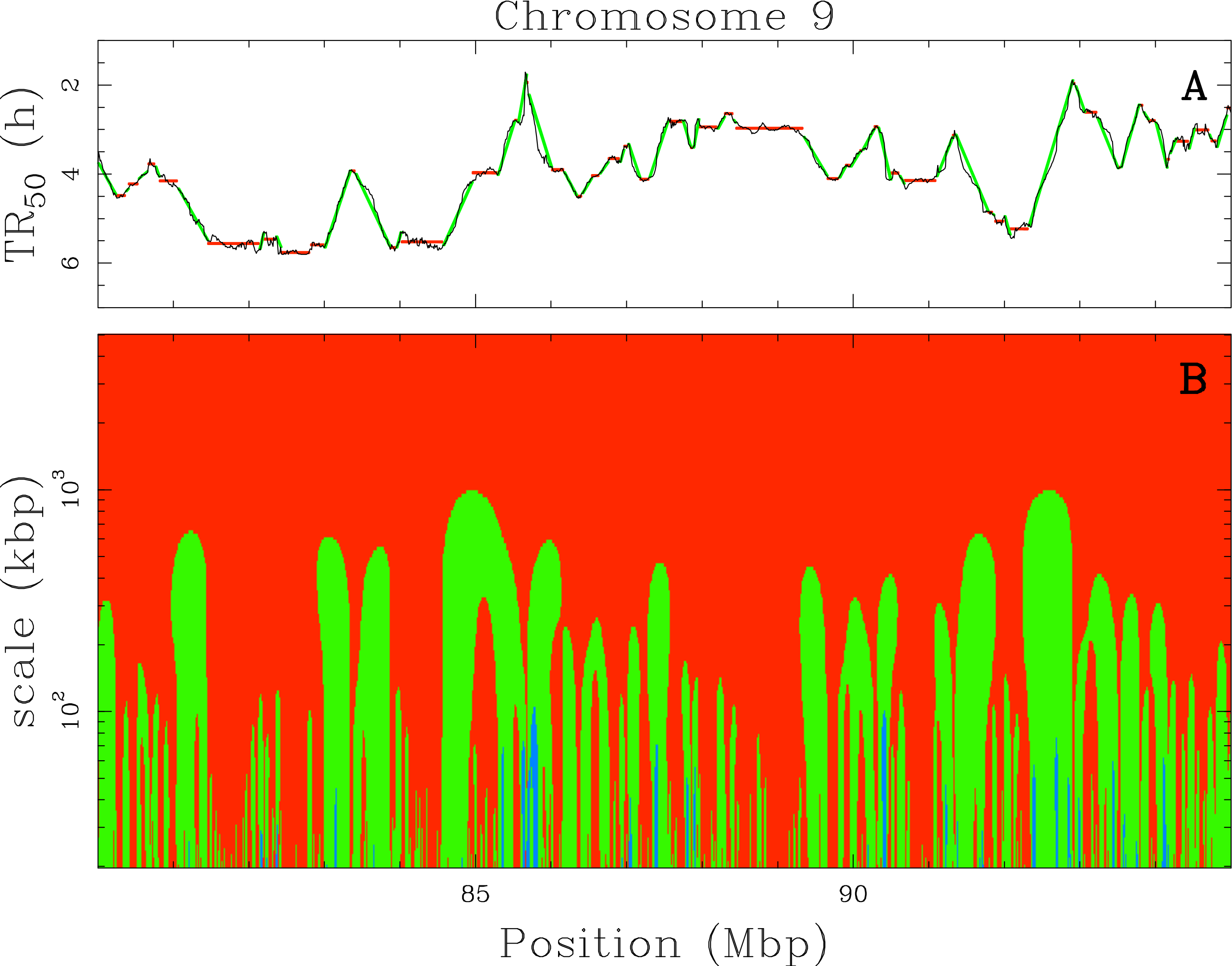
Chromosome 9
A
B
TR_{50} (h)
2
4
6
scale (kbp)
10^3
10^2
85
90
Position (Mbp)

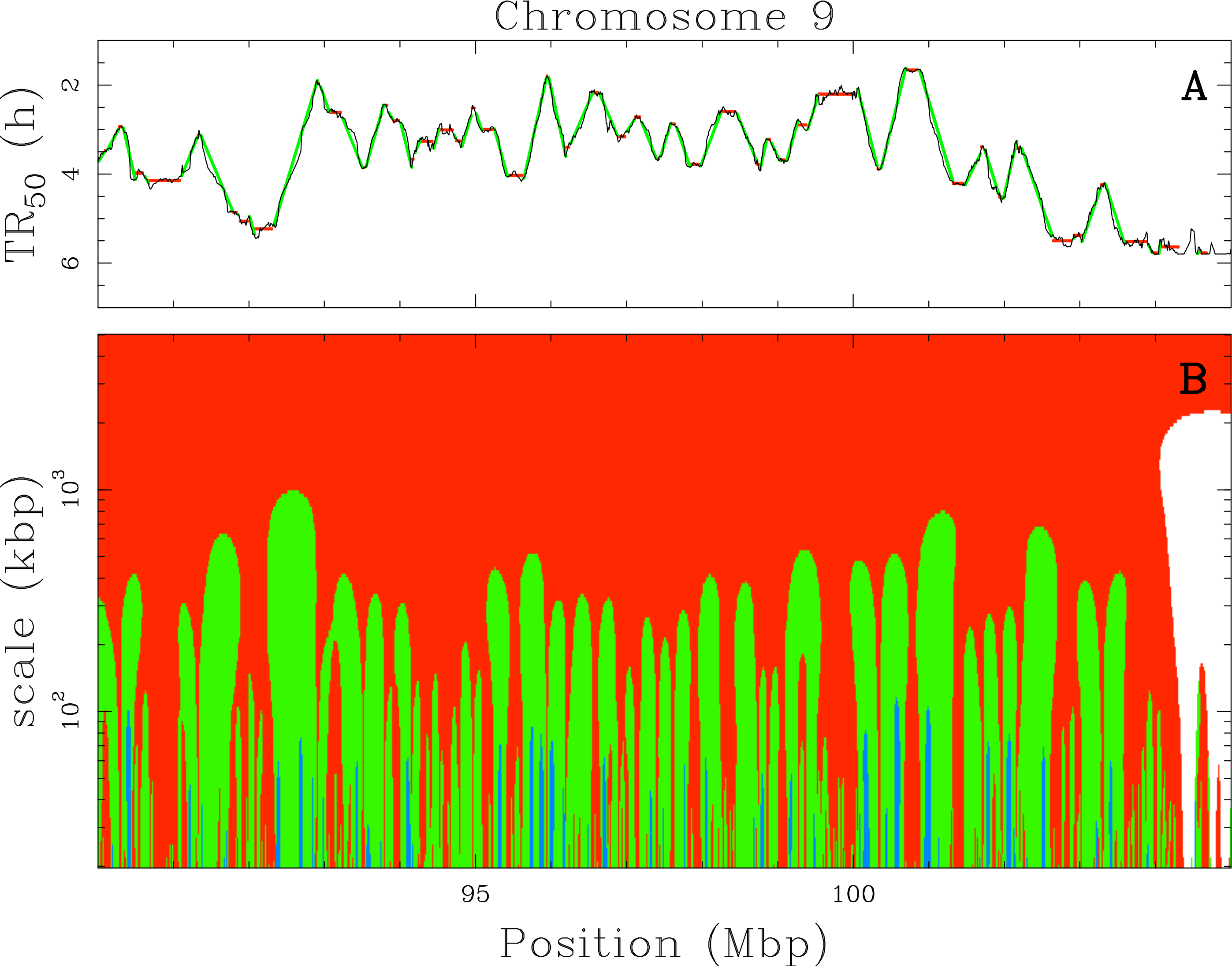

Chromosome 9
A
TR_{50} (h)
2
4
6
B
scale (kbp)
10^3
10^2
95
100
Position (Mbp)

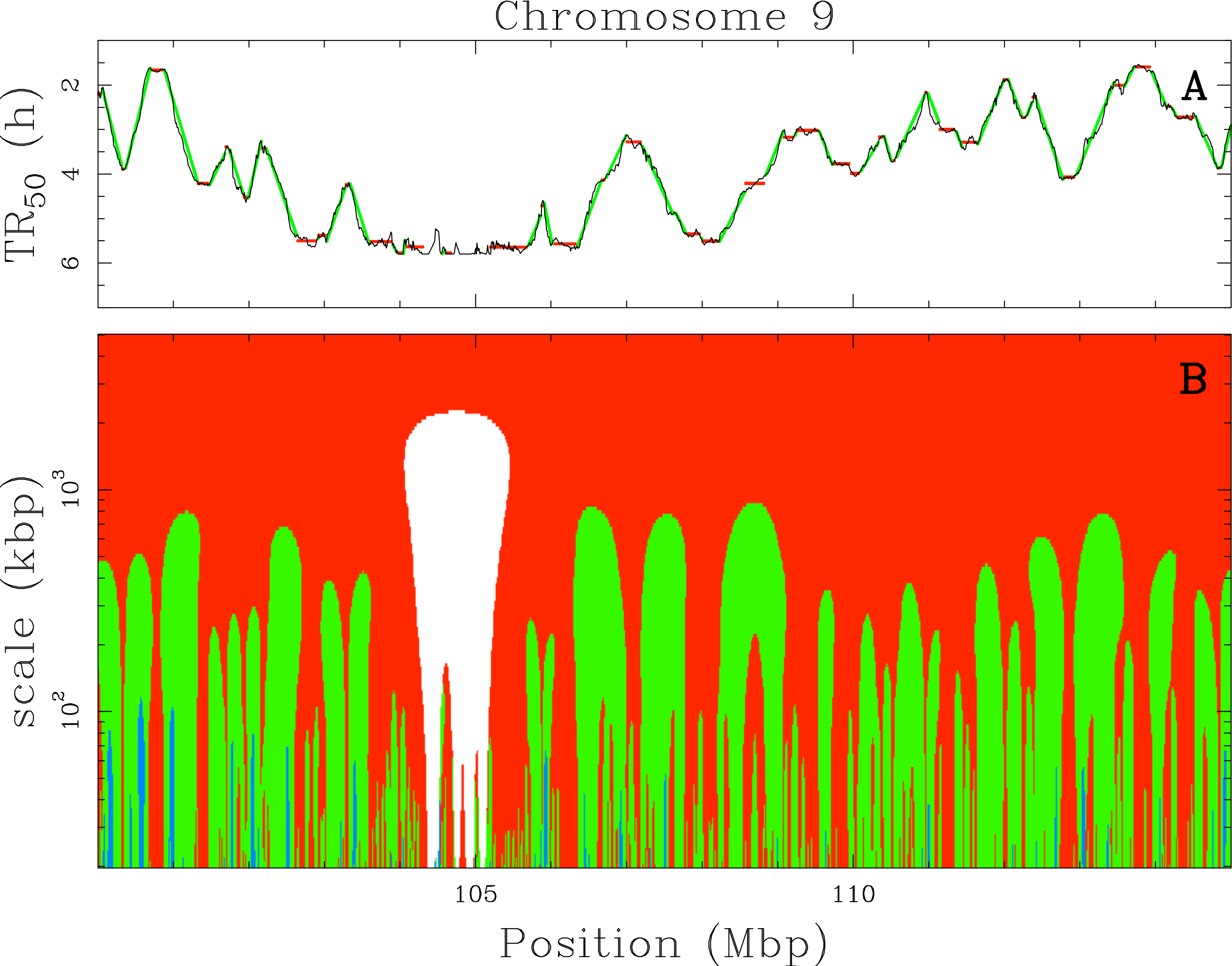

Chromosome 9
A
TR_{50} (h)
2
4
6
B
scale (kbp)
10^3
10^2
105
110
Position (Mbp)

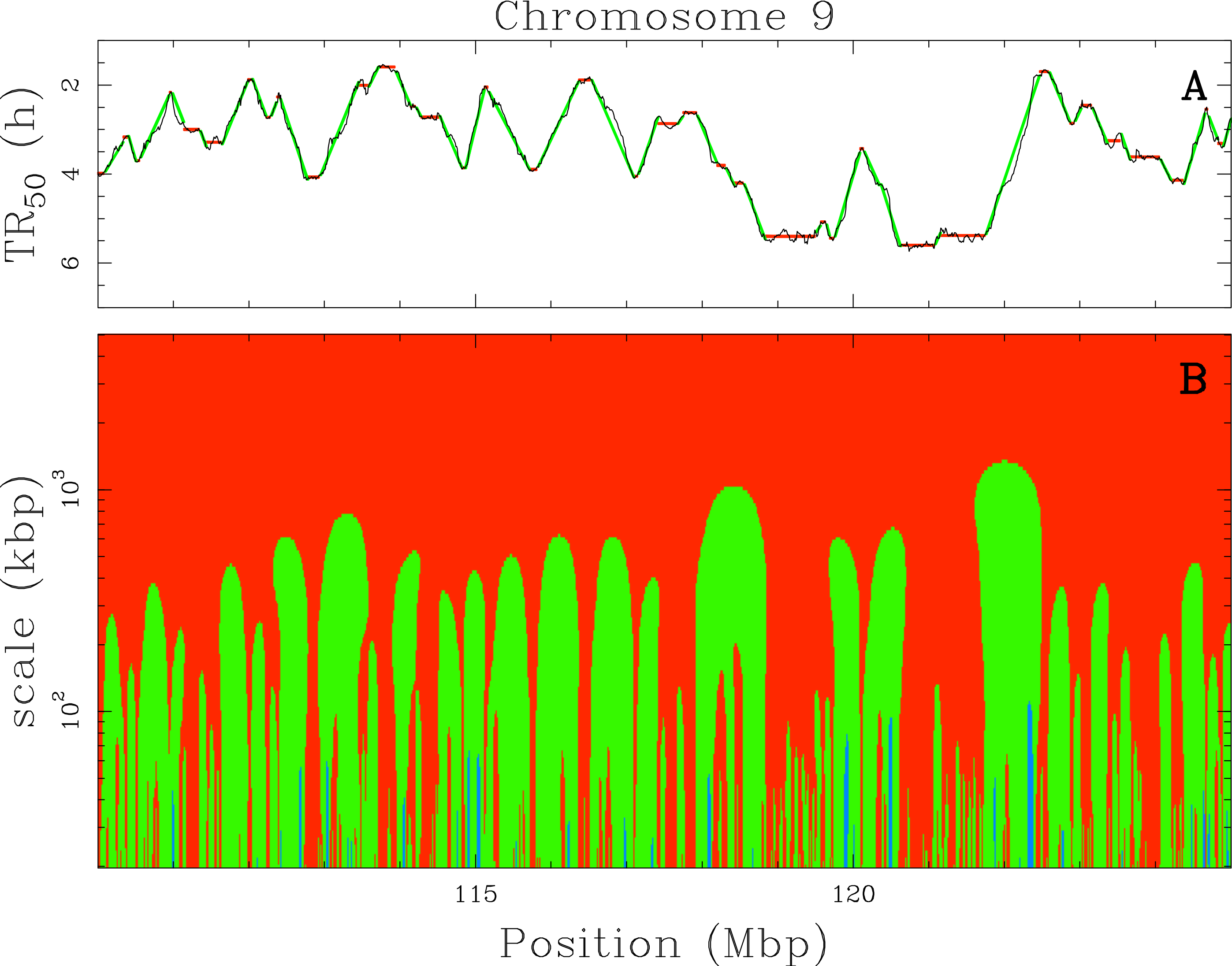

Chromosome 9
A
TR50 (h)
2
4
6
B
scale (kbp)
10^3
10^2
115
120
Position (Mbp)

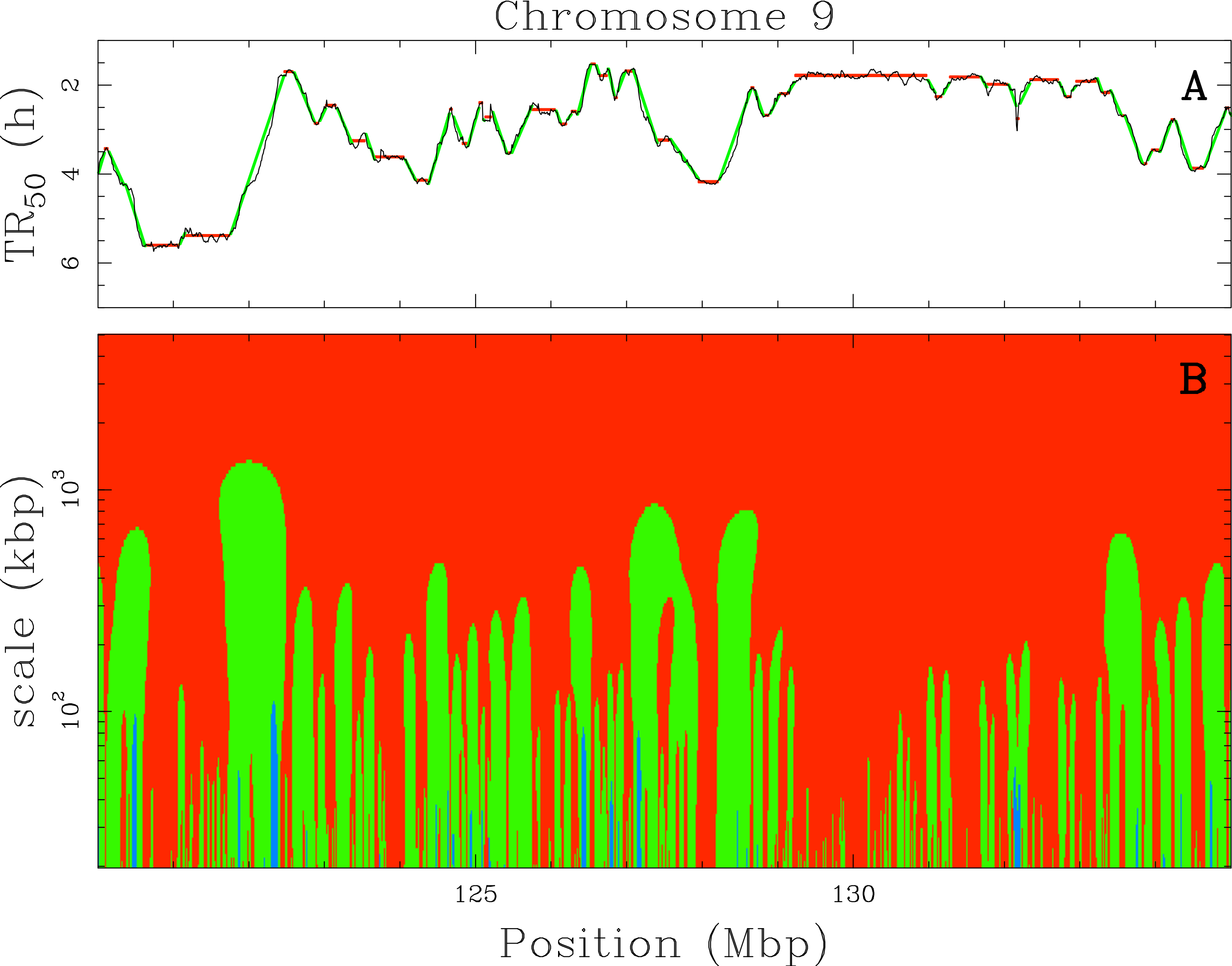
Chromosome 9
TR50 (h)
2
4
6
A
B
scale (kbp)
10^3
10^2
125
130
Position (Mbp)

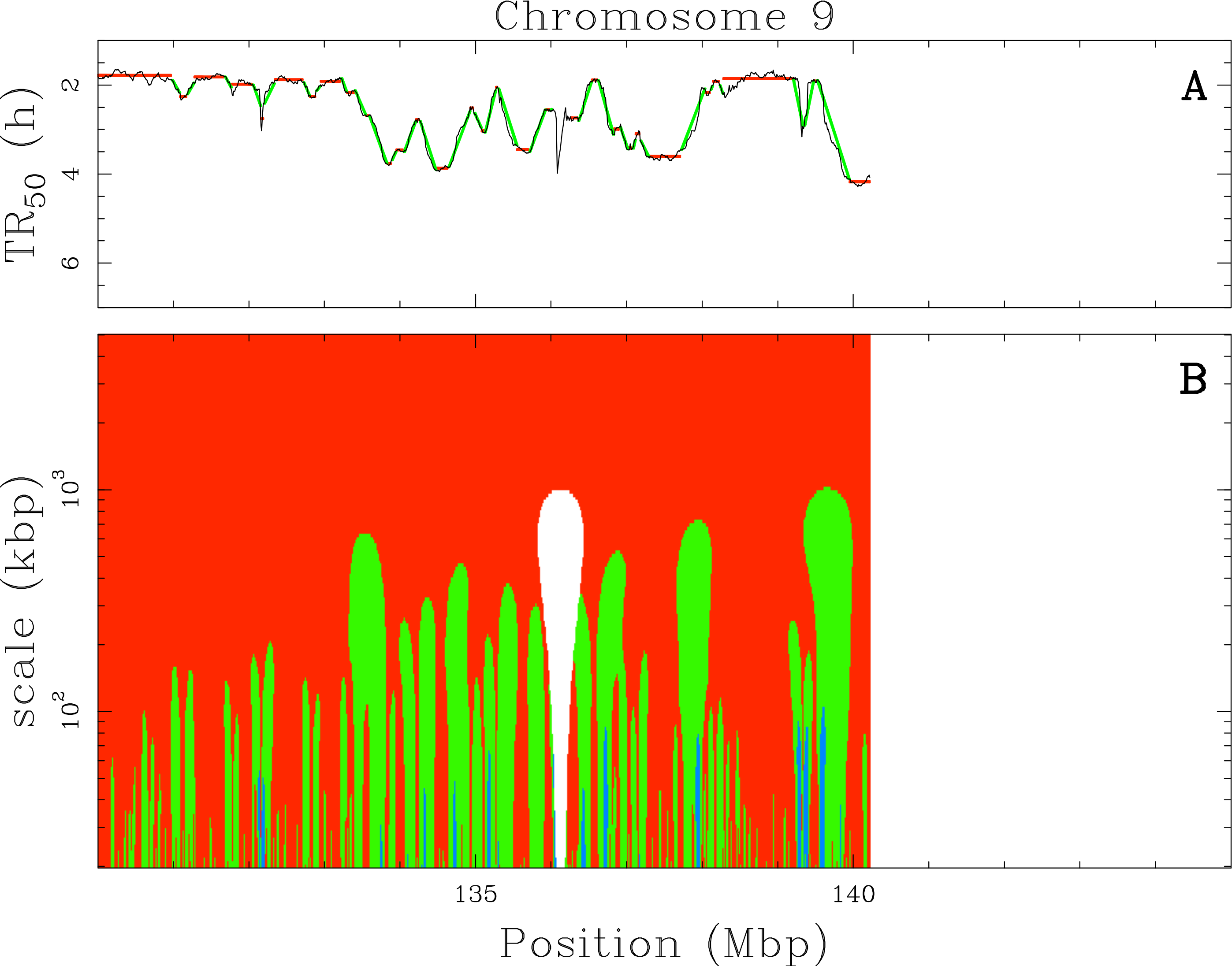

Chromosome 9
TR50 (h)
2
4
6
A
B
scale (kbp)
10^3
10^2
135
140
Position (Mbp)

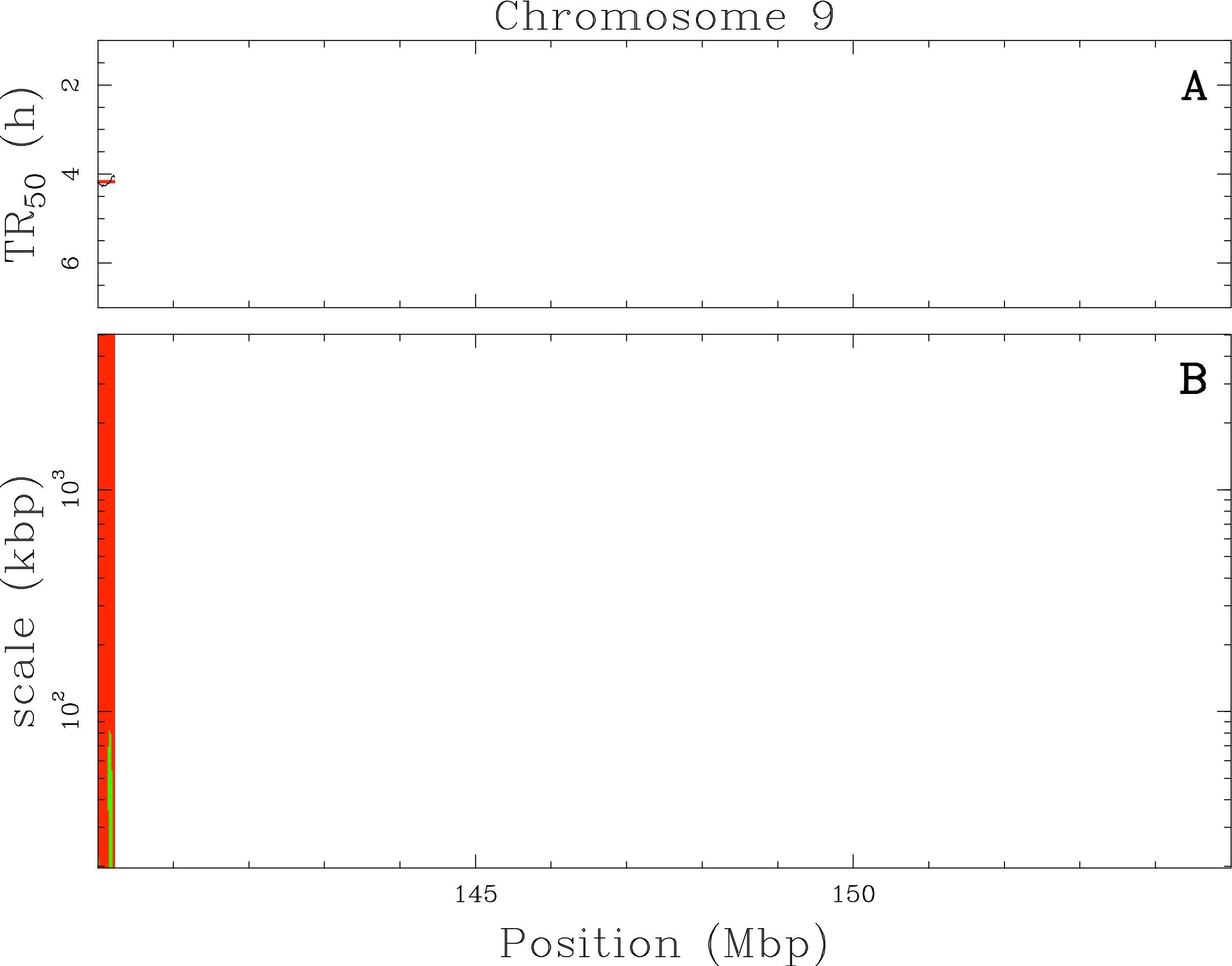

Chromosome 9
A
B
TR50 (h)
2
4
6
scale (kbp)
10^3
10^2
145
150
Position (Mbp)

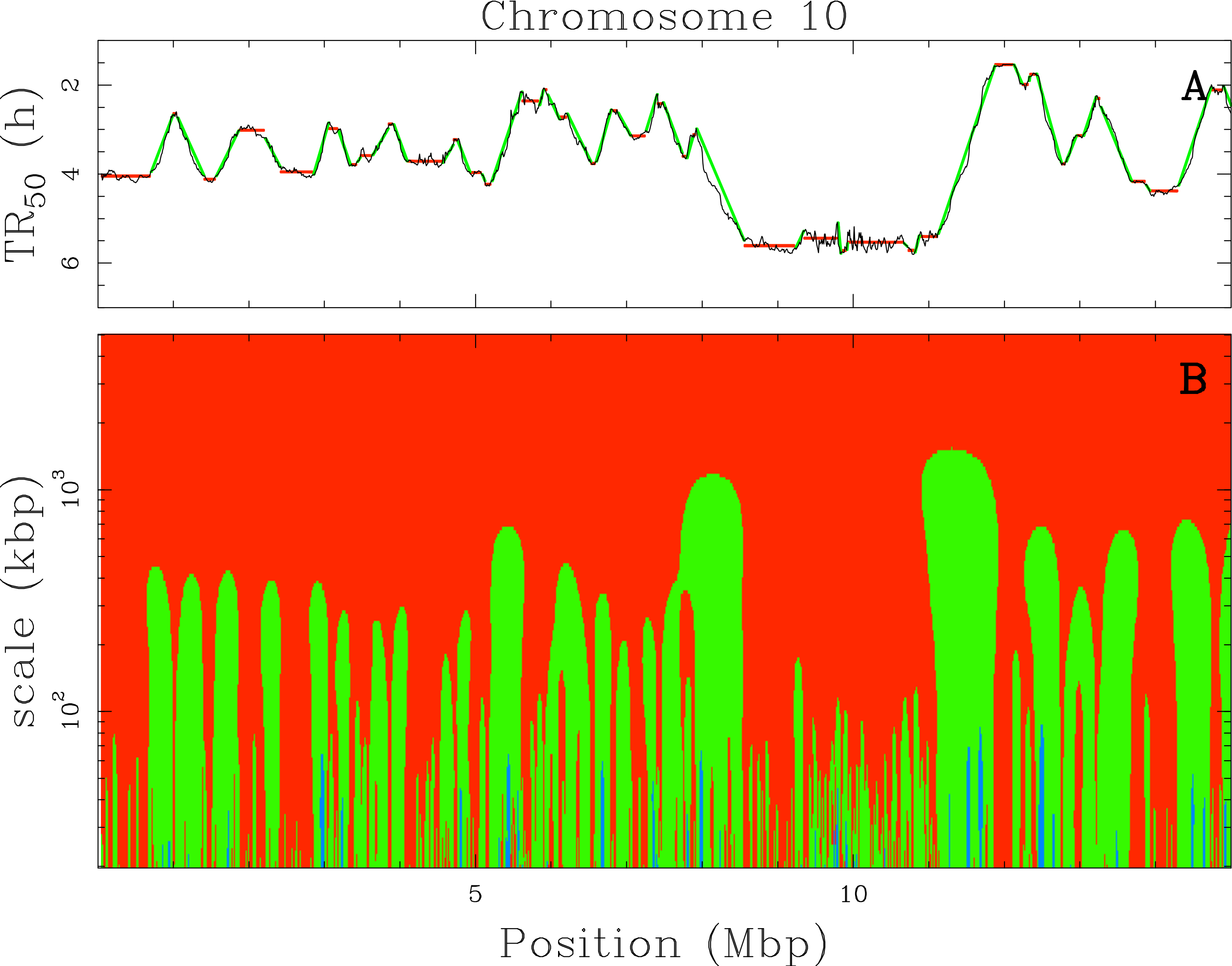

Chromosome 10
A
B
TR50 (h)
2
4
6
scale (kbp)
10^3
10^2
5
10
Position (Mbp)

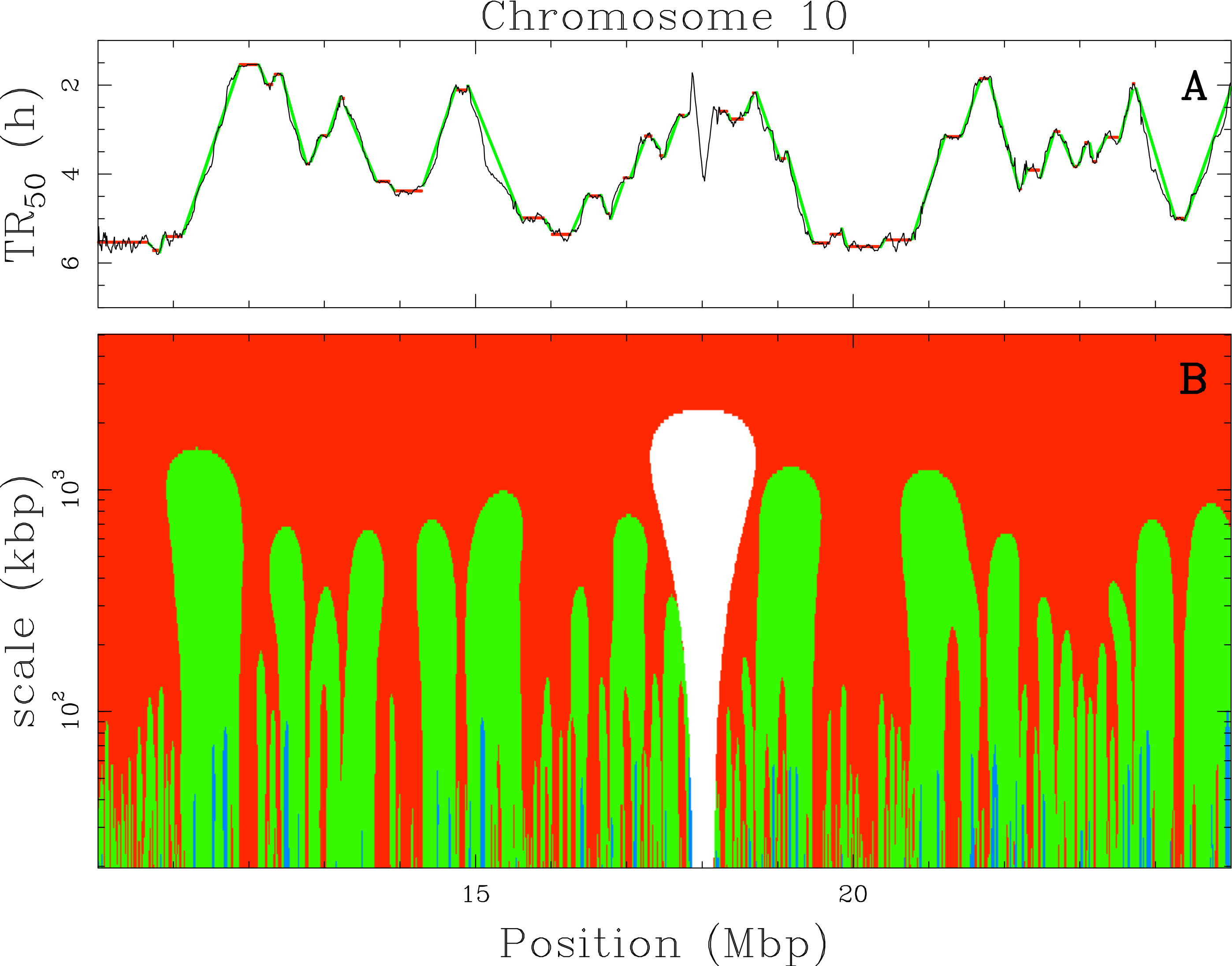

Chromosome 10
TR$_{50}$ (h)
2
4
6
A
B
scale (kbp)
10^3
10^2
15
20
Position (Mbp)

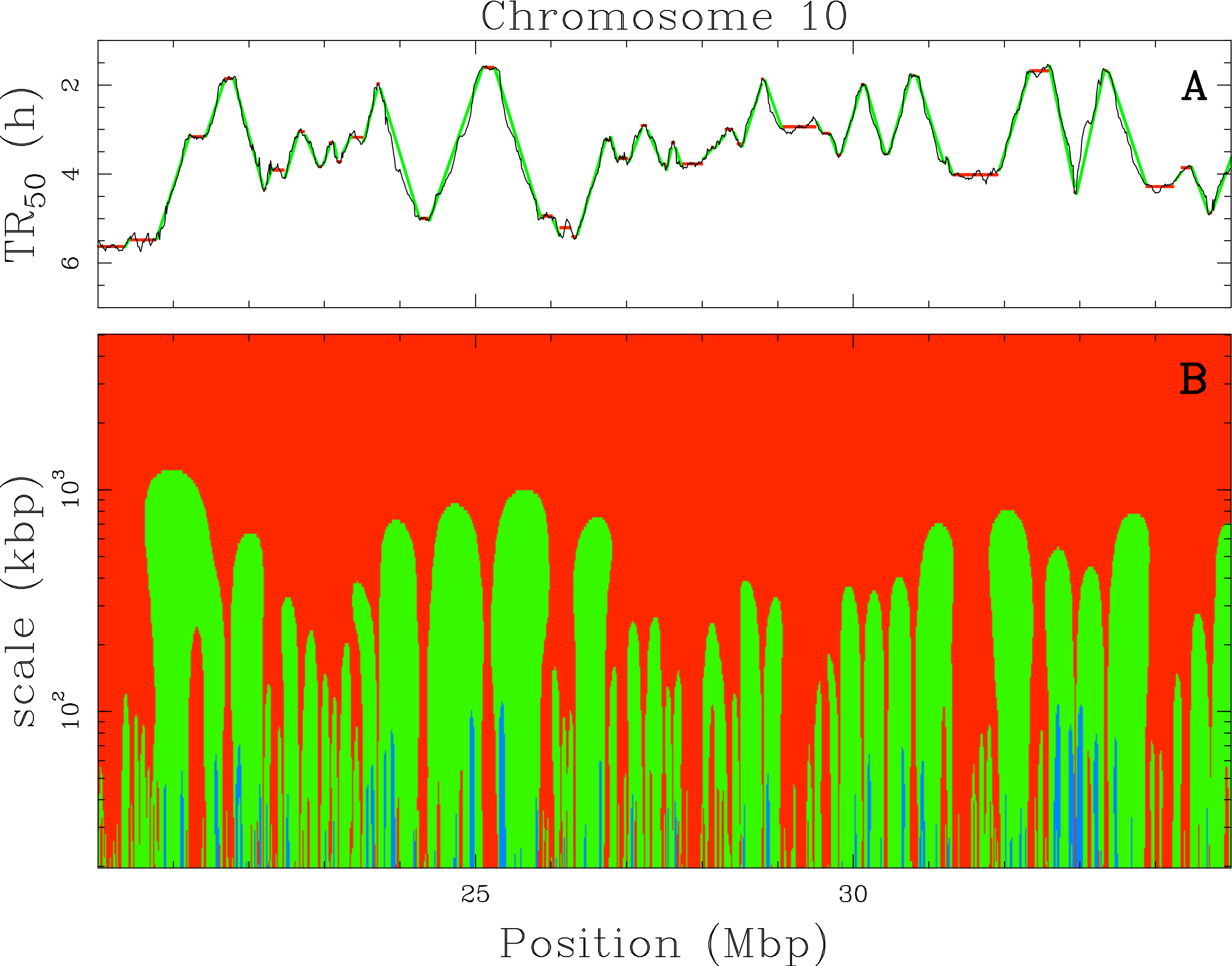

Chromosome 10
A
TR50 (h)
2
4
6
B
scale (kbp)
10^3
10^2
25
30
Position (Mbp)

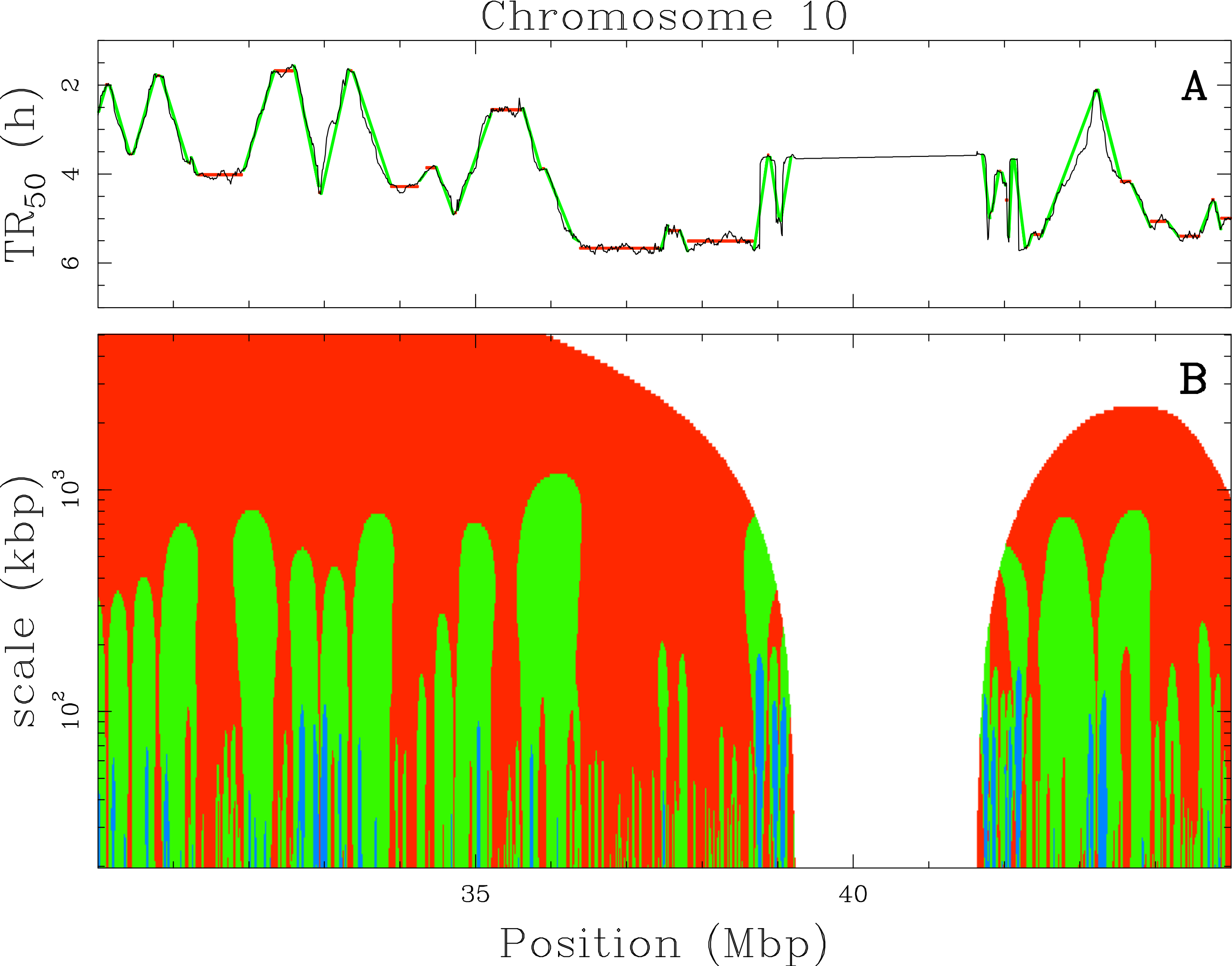

Chromosome 10
A
B
TR50 (h)
2
4
6
scale (kbp)
10^3
10^2
35
40
Position (Mbp)

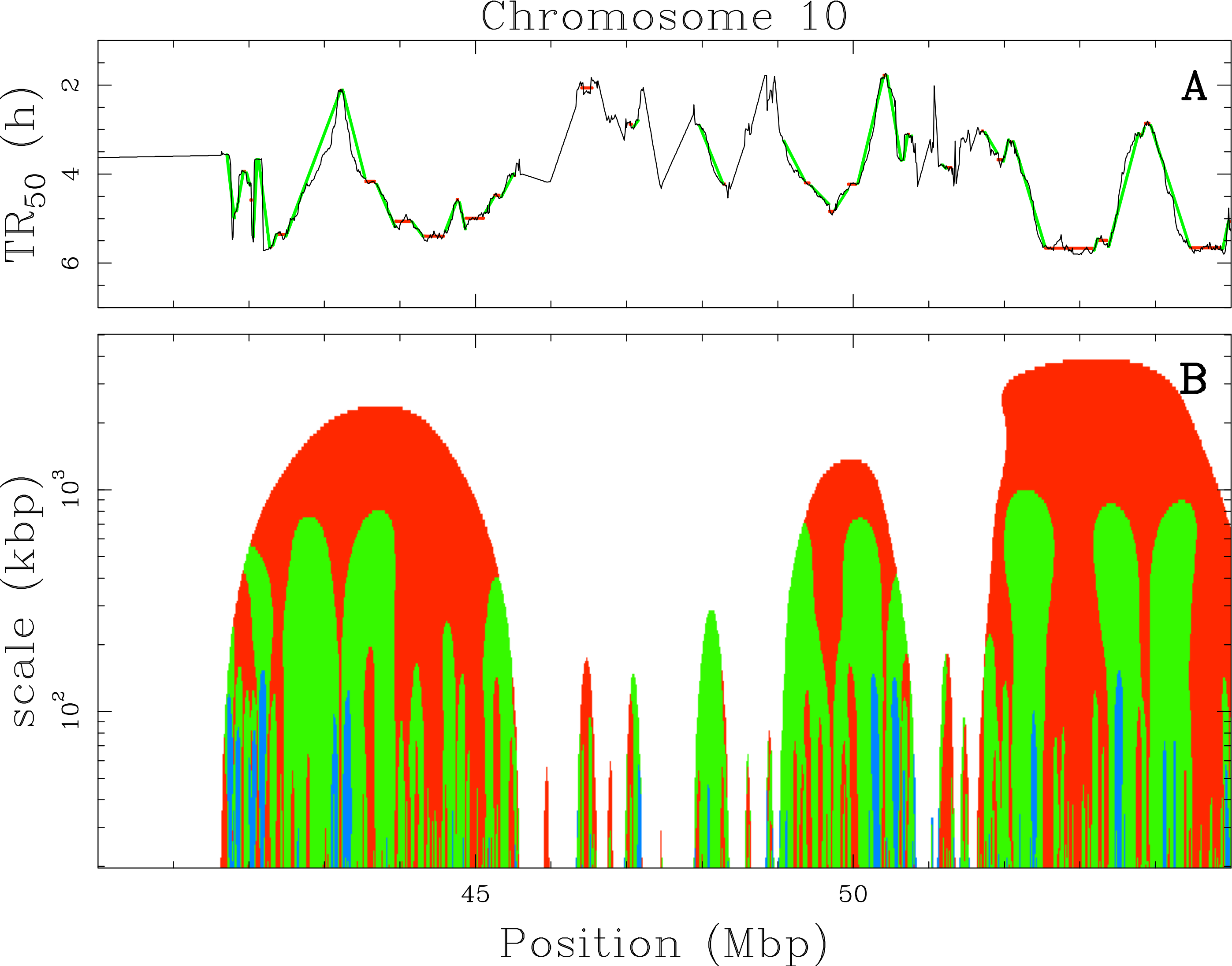
Chromosome 10
TR50 (h)
2
4
6
A
B
scale (kbp)
10^3
10^2
45
50
Position (Mbp)

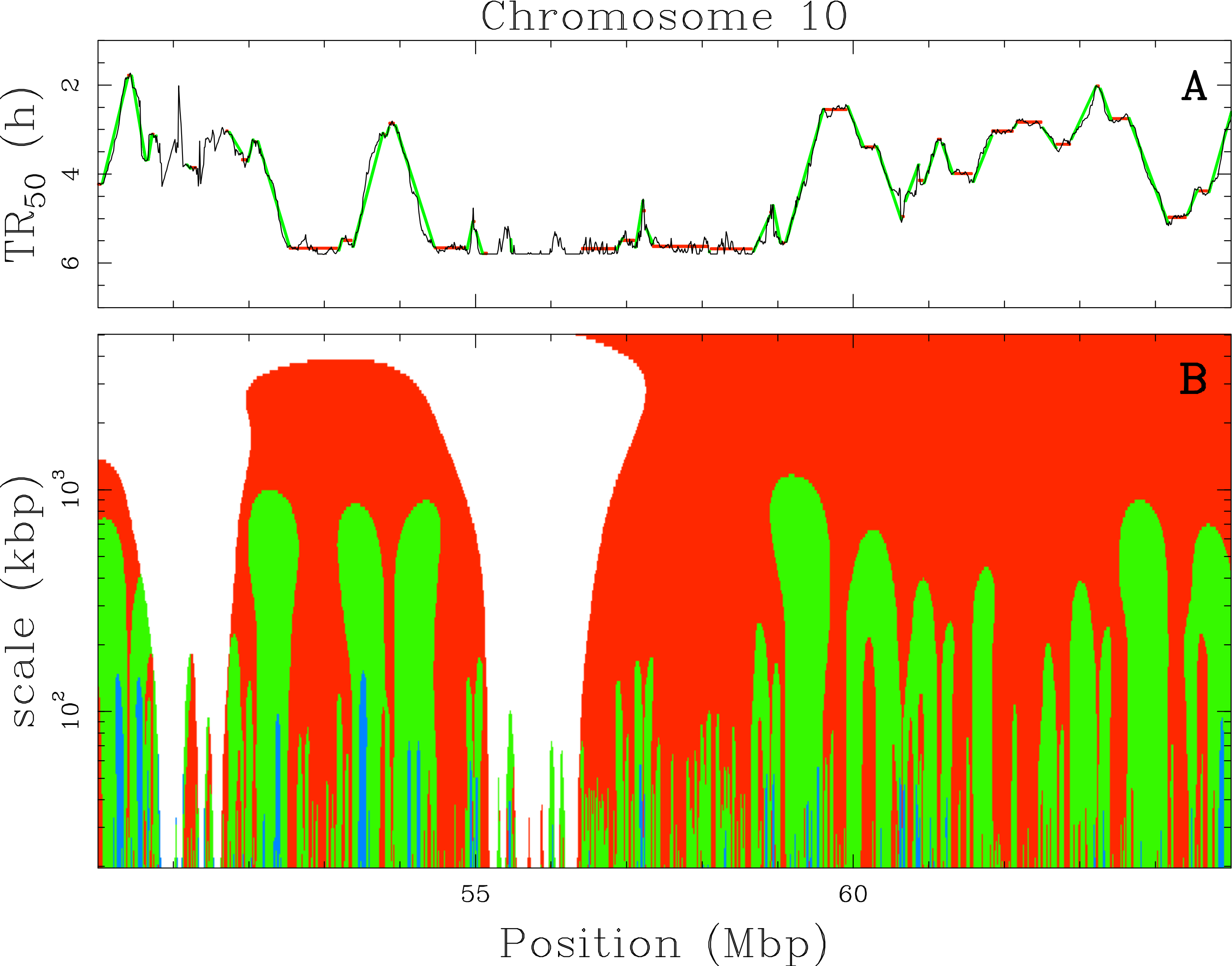

Chromosome 10
A
TR50 (h)
2
4
6
B
scale (kbp)
10^3
10^2
55
60
Position (Mbp)

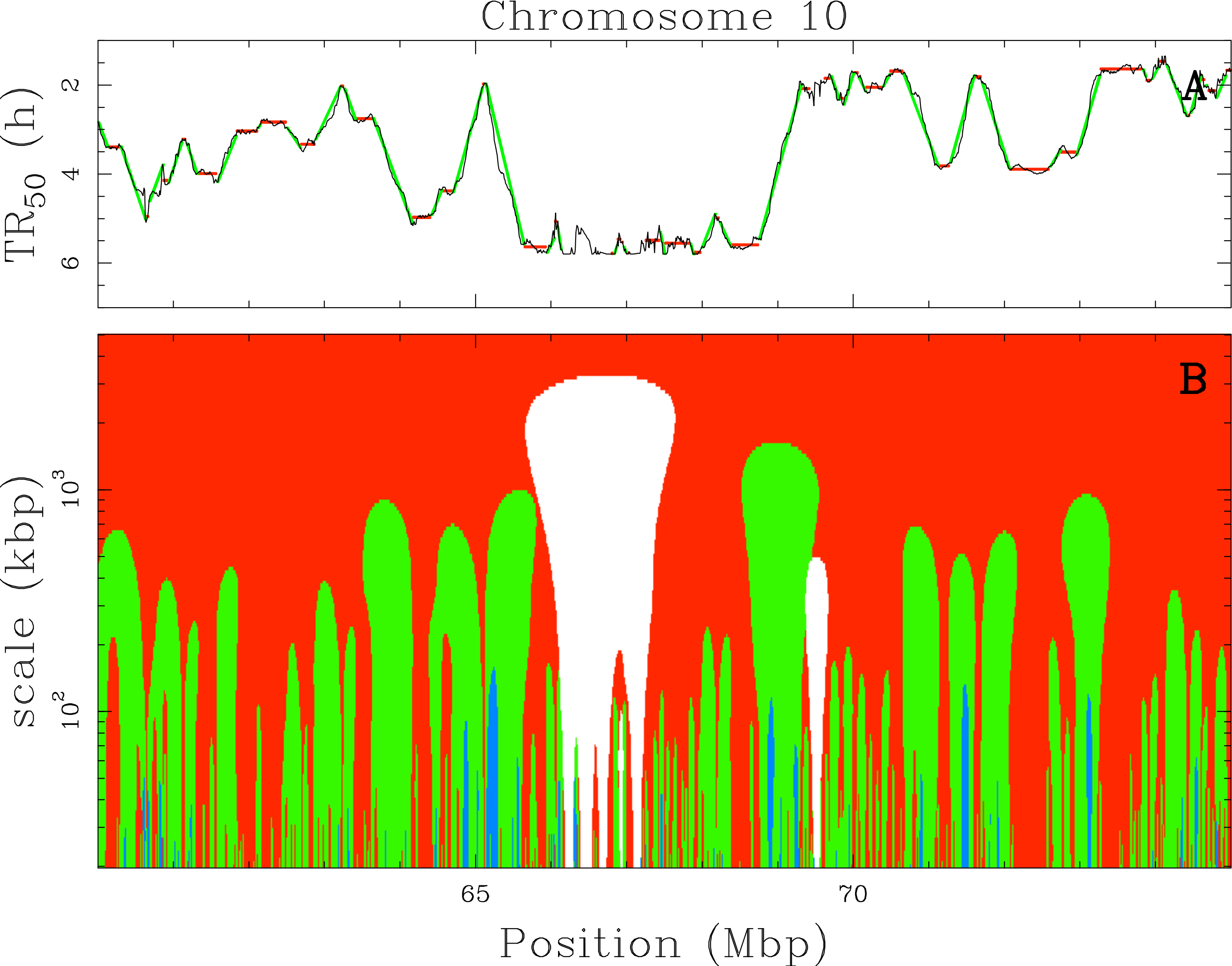

Chromosome 10
TR50 (h)
2
4
6
A
B
scale (kbp)
10^3
10^2
65
70
Position (Mbp)

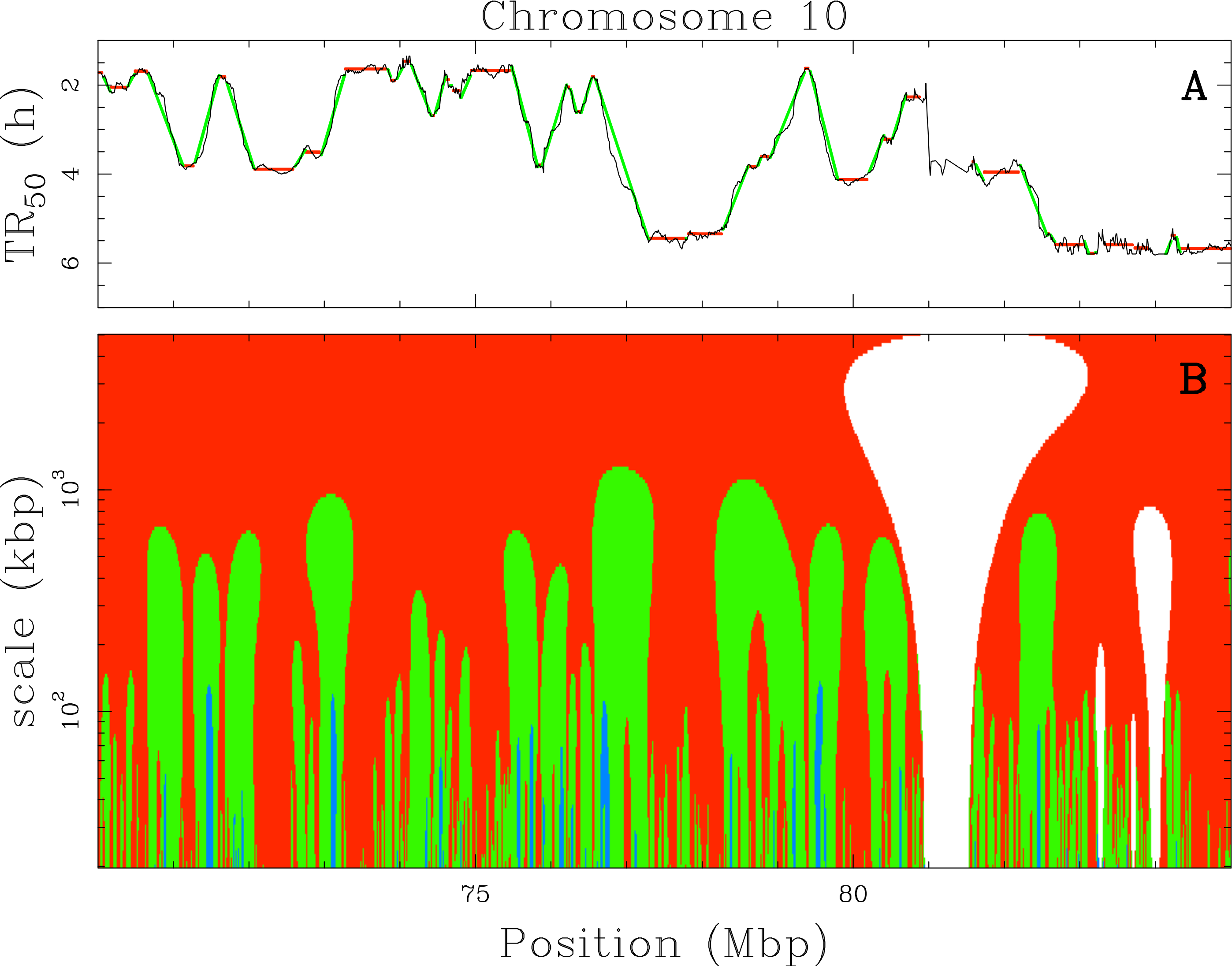
Chromosome 10
A
TR50 (h)
2
4
6
B
scale (kbp)
10^3
10^2
75
80
Position (Mbp)

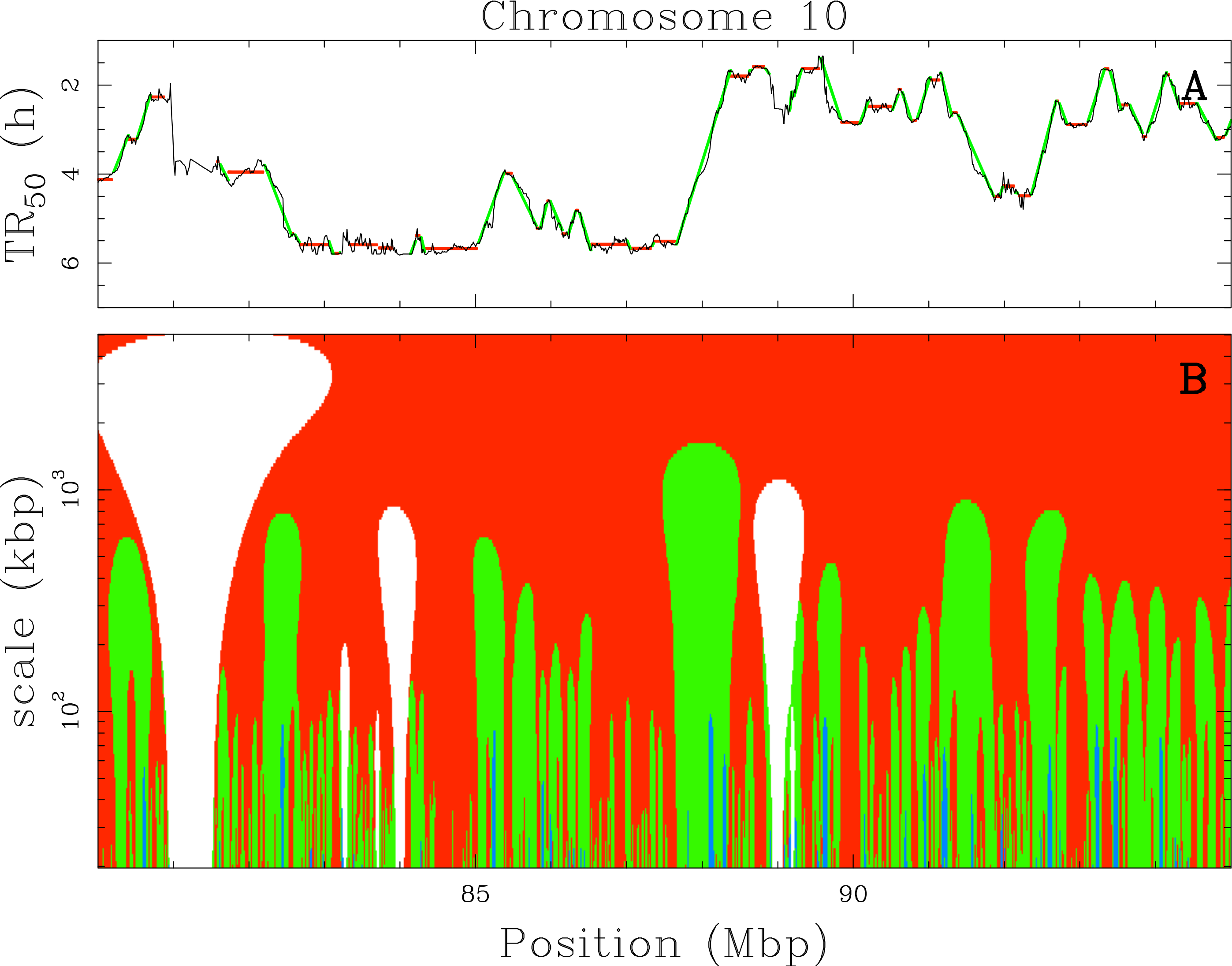
Chromosome 10
TR50 (h)
2
4
6
A
B
scale (kbp)
10^3
10^2
85
90
Position (Mbp)

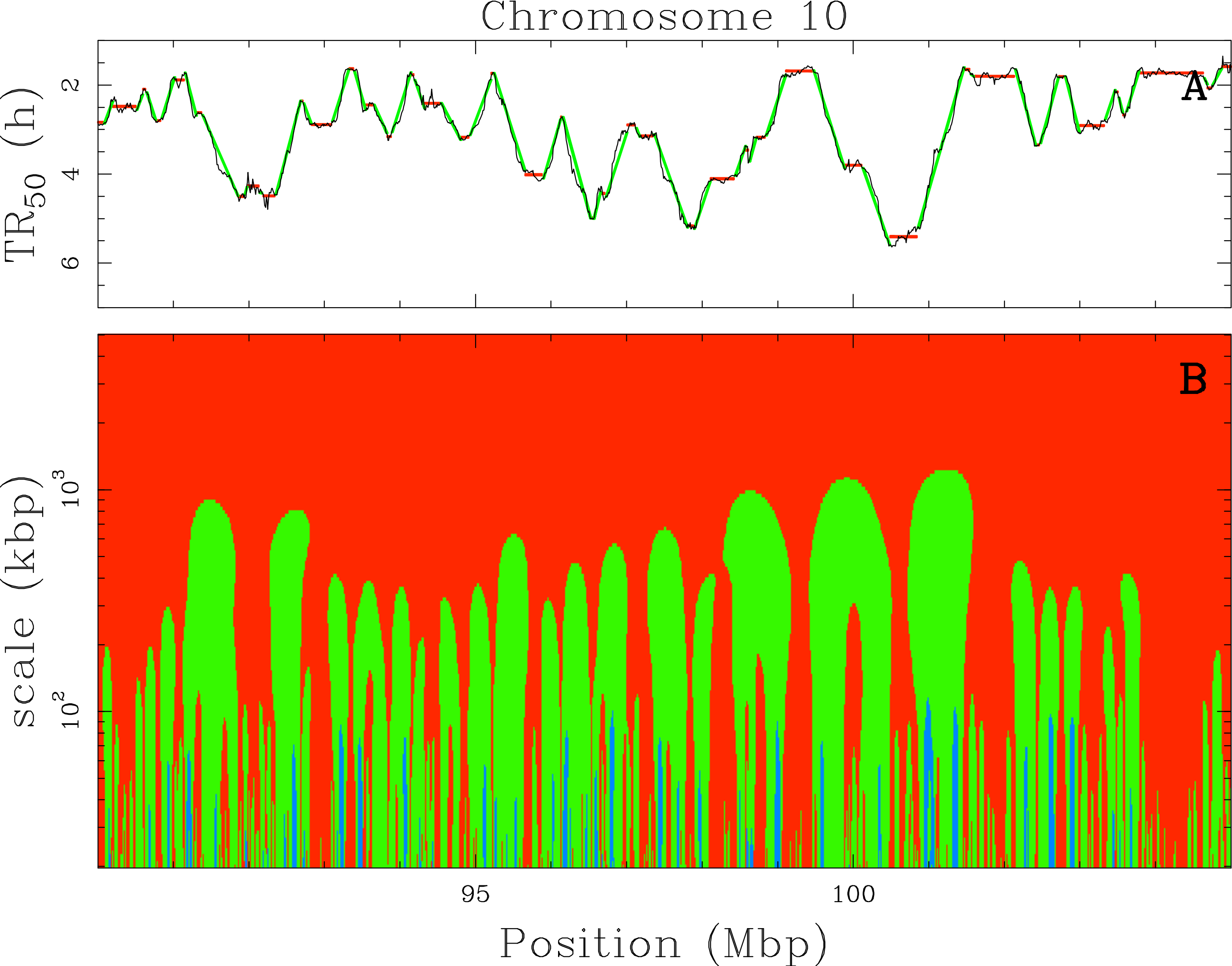

Chromosome 10
TR50 (h)
2
4
6
A
B
scale (kbp)
10^3
10^2
95
100
Position (Mbp)

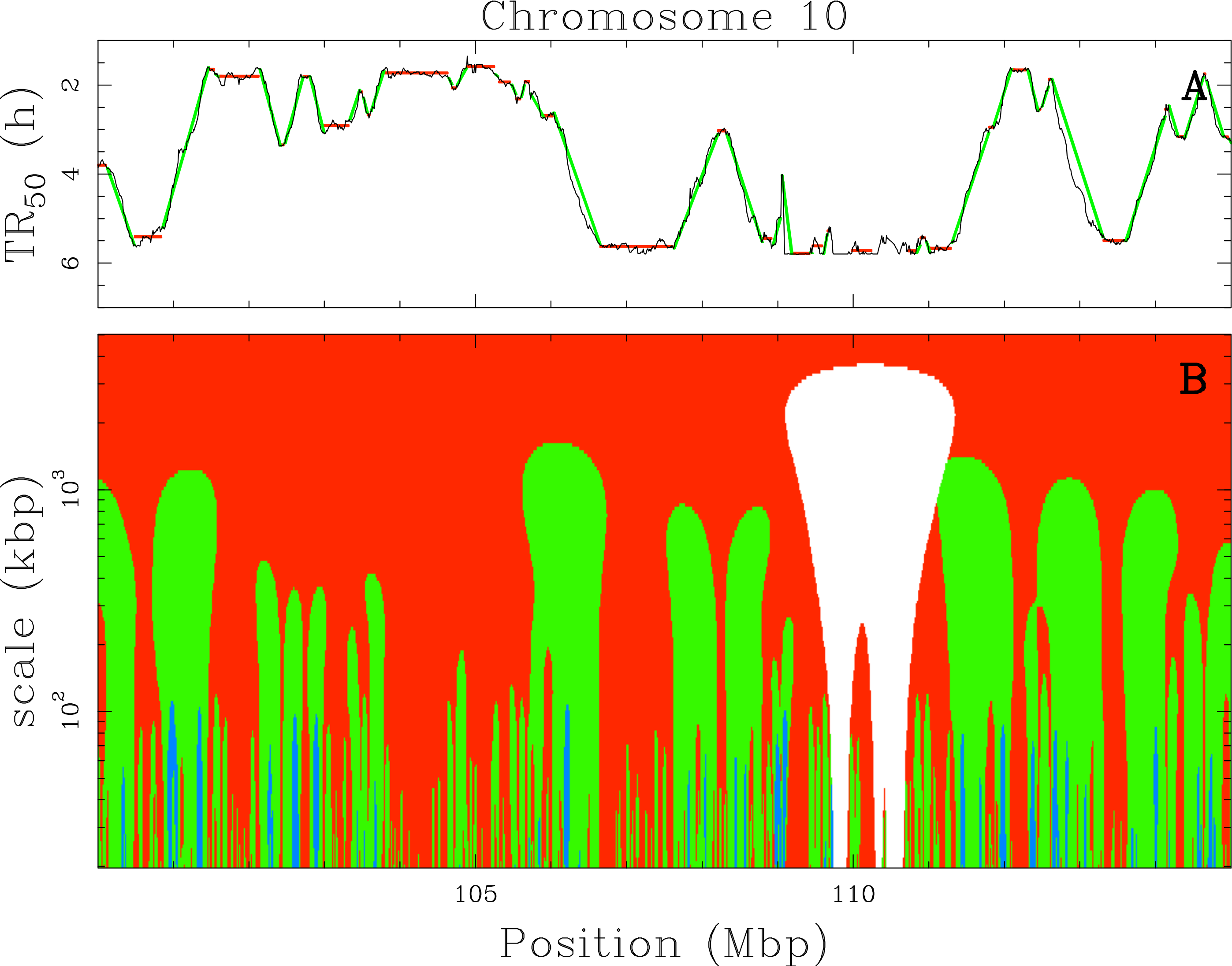
Chromosome 10
A
B
TR50 (h)
2
4
6
scale (kbp)
10^3
10^2
105
110
Position (Mbp)

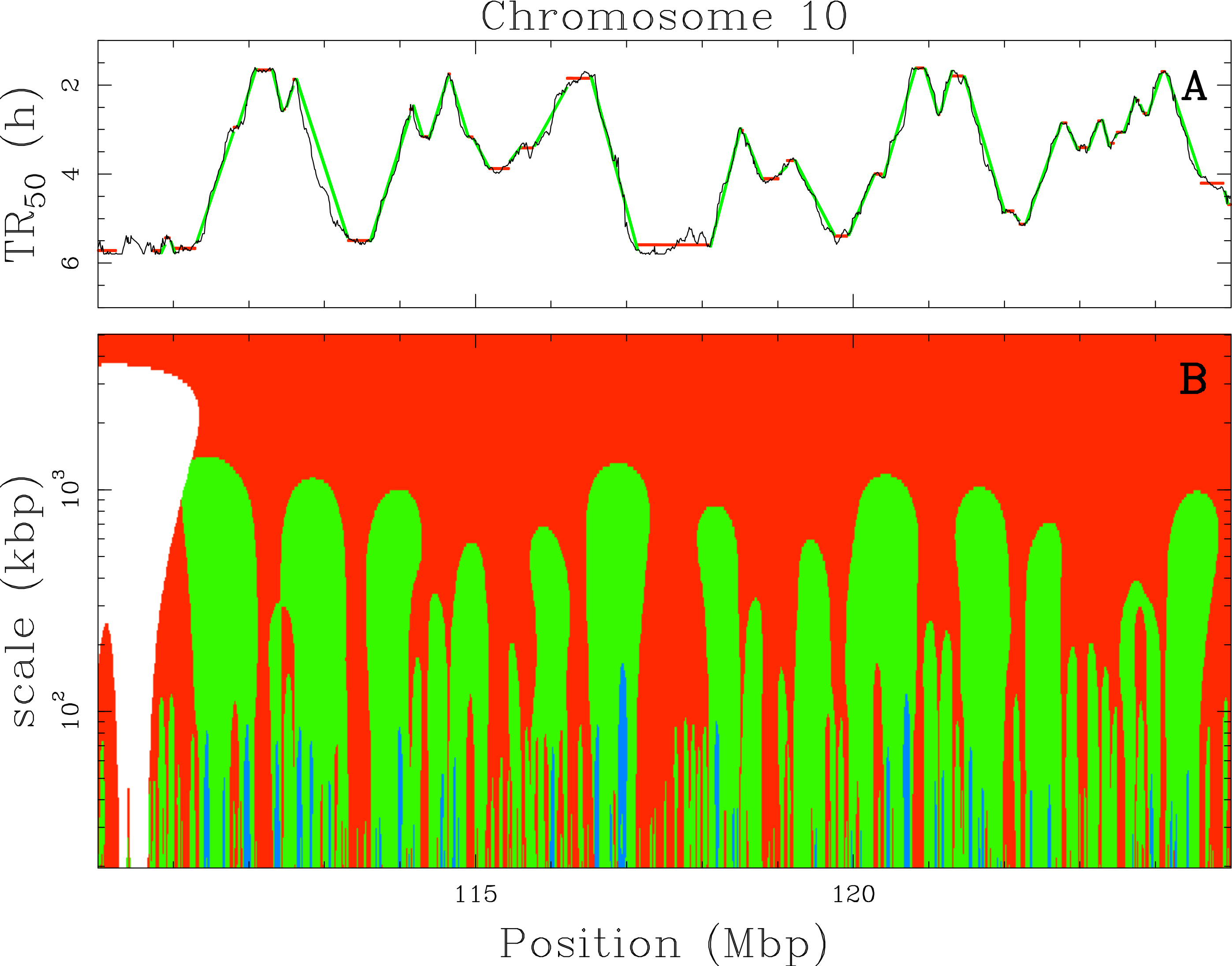

Chromosome 10
TR50 (h)
2
4
6
A
B
scale (kbp)
10^3
10^2
115
120
Position (Mbp)

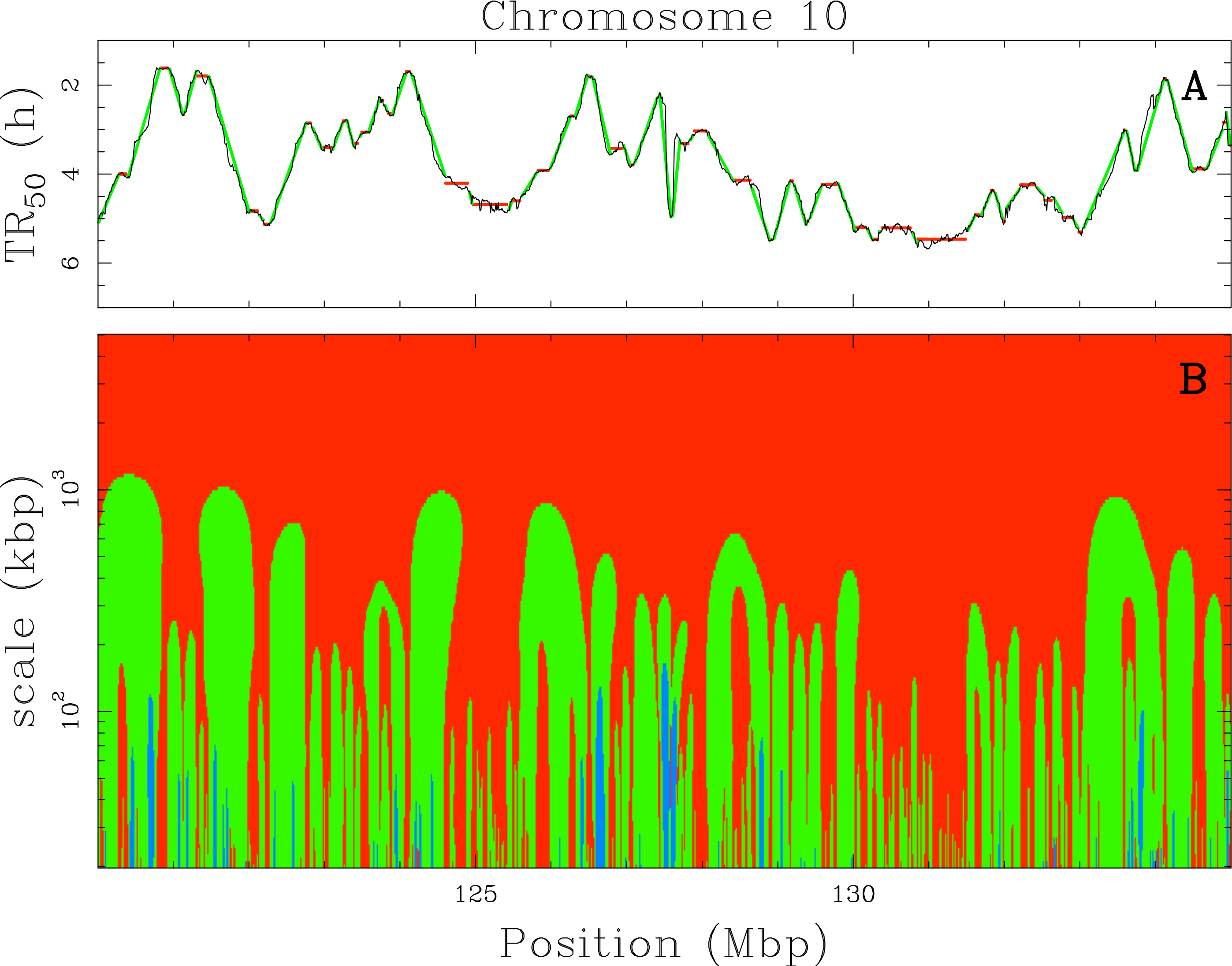

Chromosome 10
A
TR50 (h)
2
4
6
B
scale (kbp)
10^3
10^2
125
130
Position (Mbp)

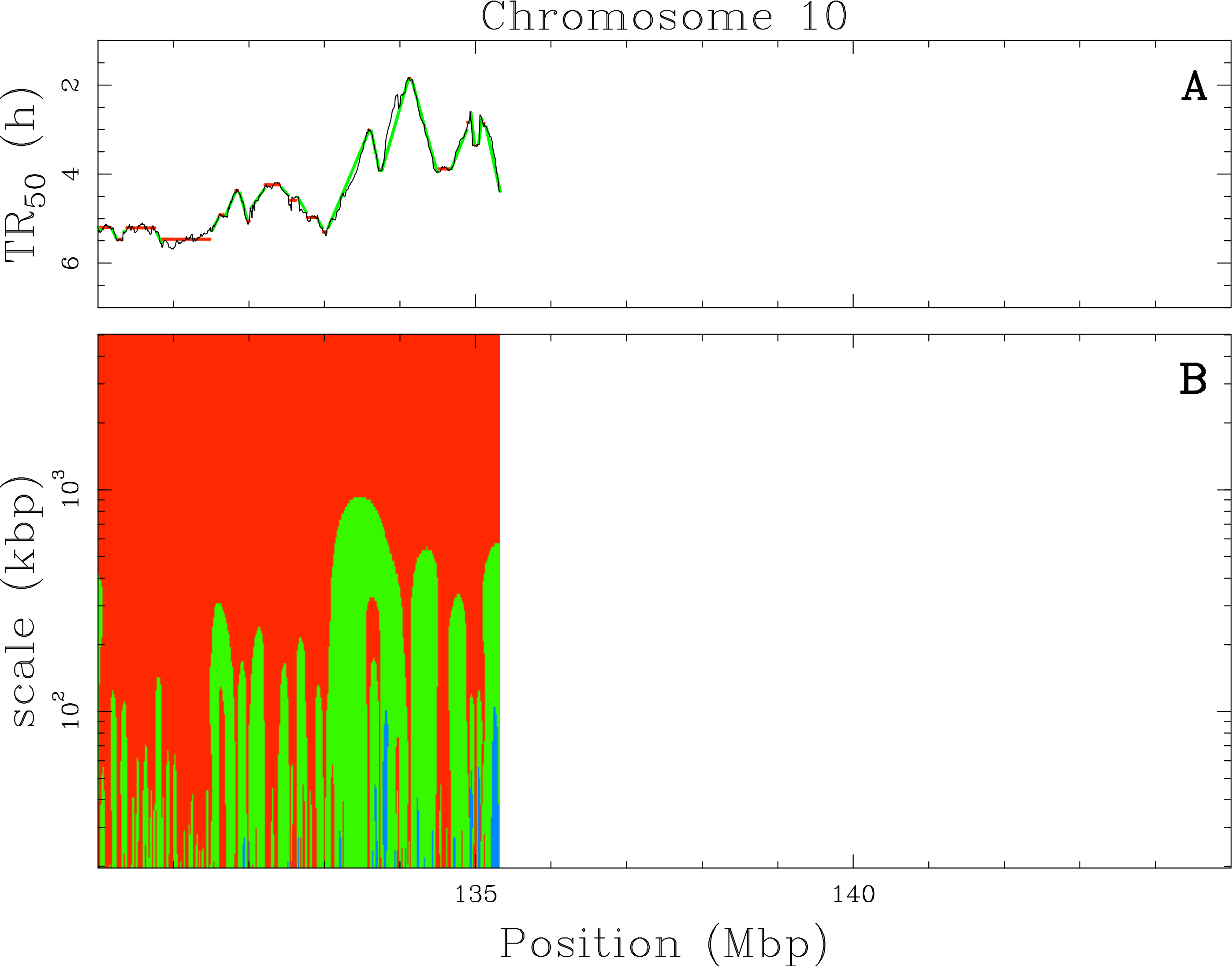
Chromosome 10
A
B
TR_{50} (h)
2
4
6
scale (kbp)
10^3
10^2
135
140
Position (Mbp)

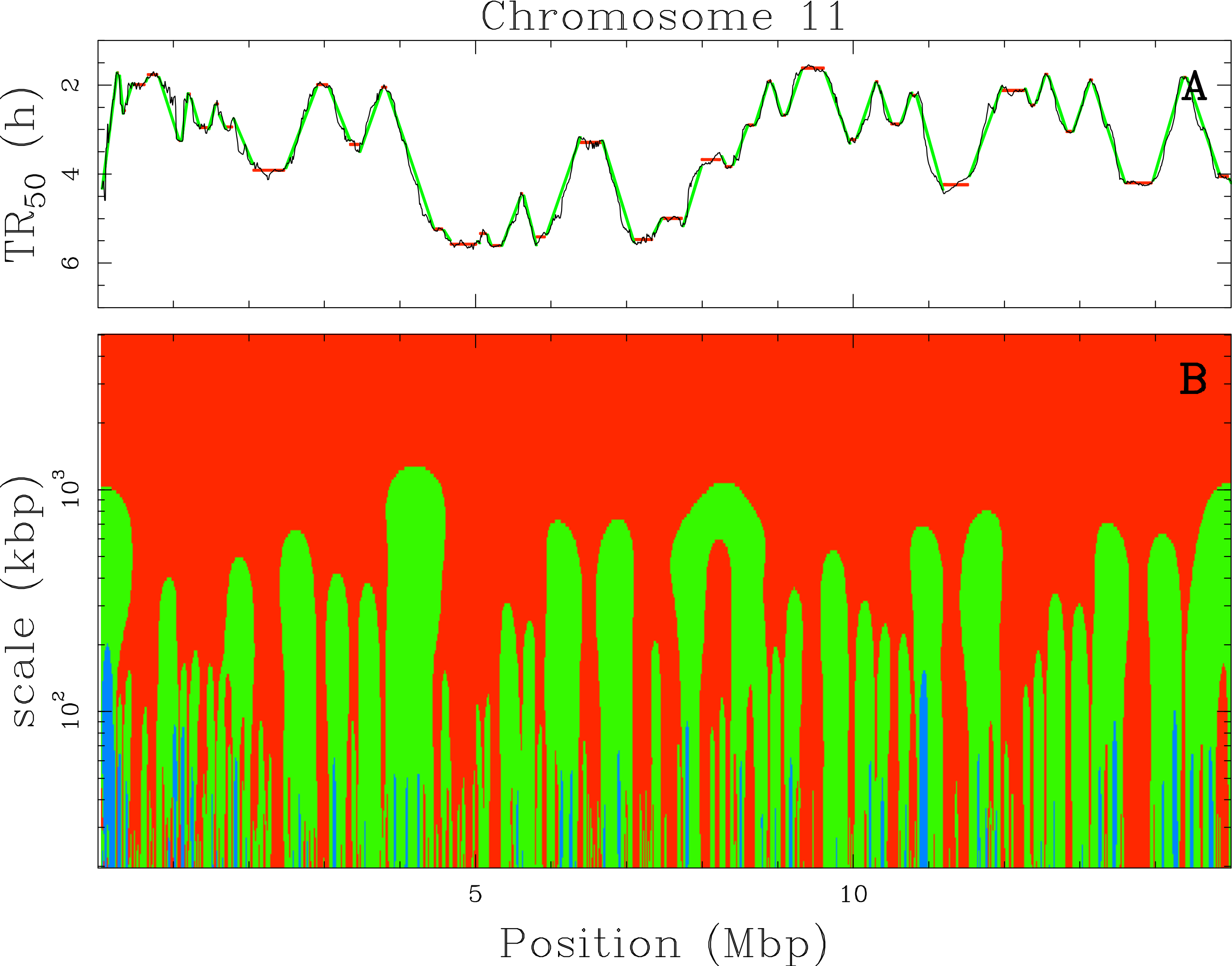
Chromosome 11
A
B
TR50 (h)
2
4
6
scale (kbp)
10^3
10^2
5
10
Position (Mbp)

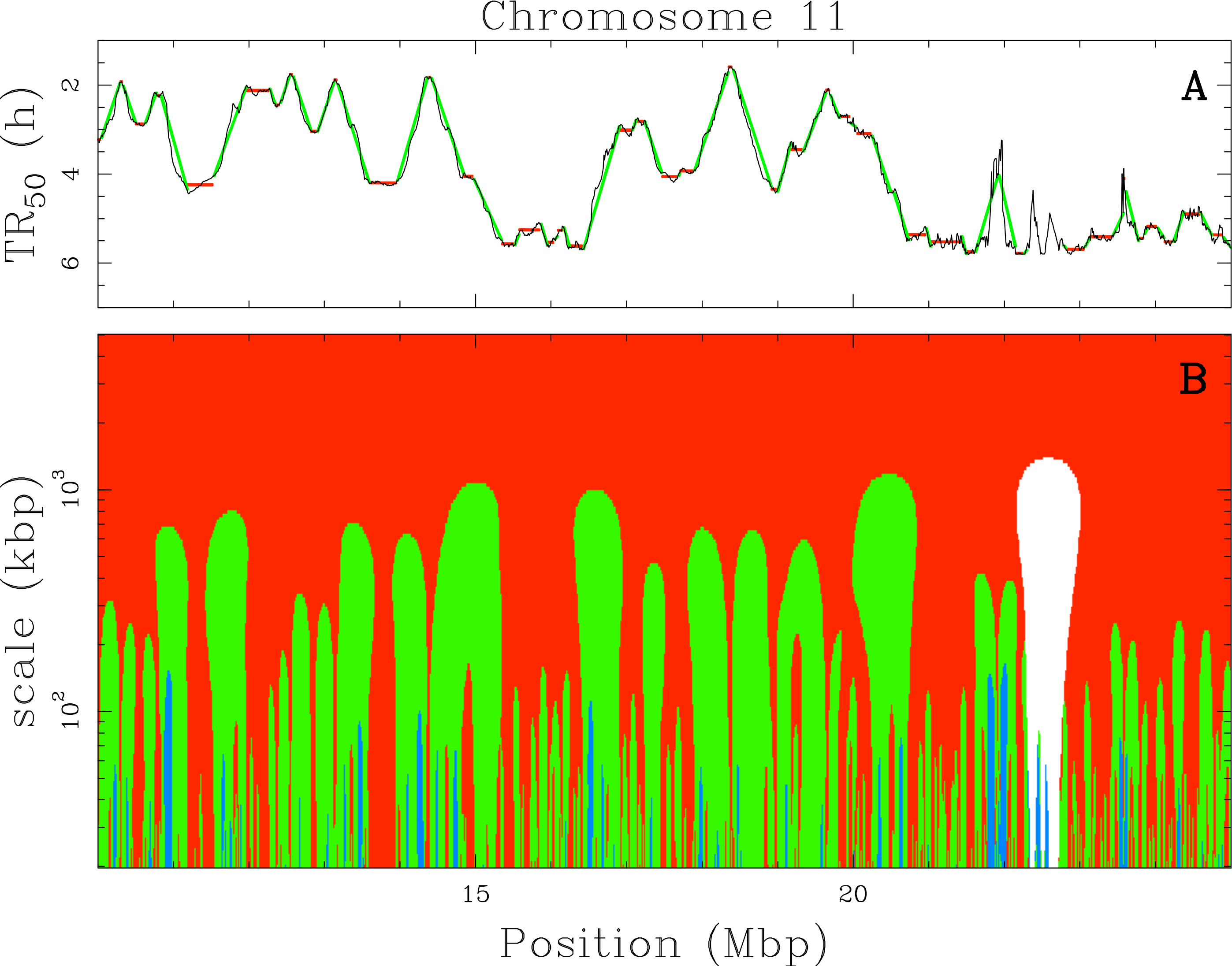

Chromosome 11
A
TR50 (h)
2
4
6
B
scale (kbp)
10^3
10^2
15
20
Position (Mbp)

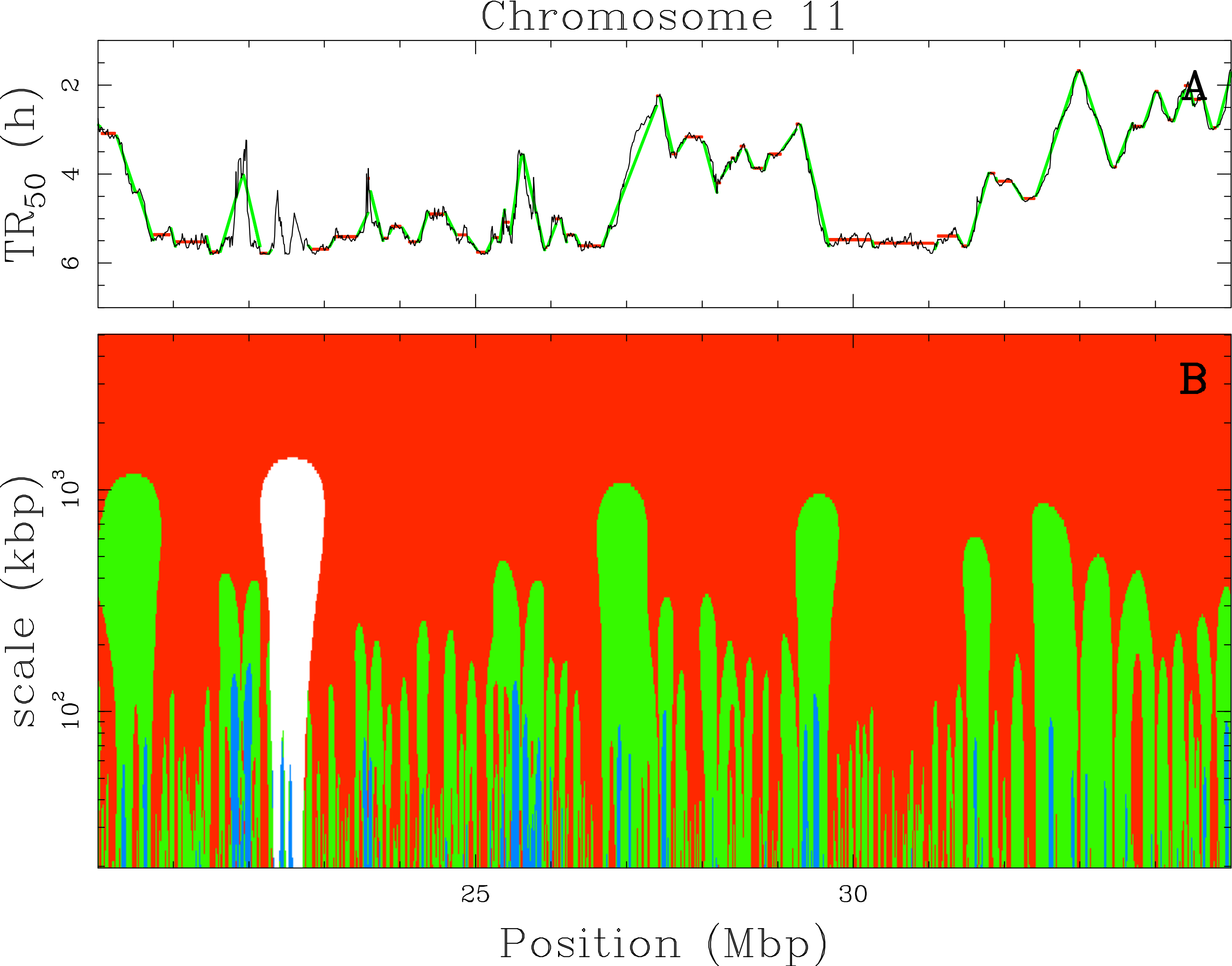

Chromosome 11
A
B
TR50 (h)
2
4
6
scale (kbp)
10^3
10^2
25
30
Position (Mbp)

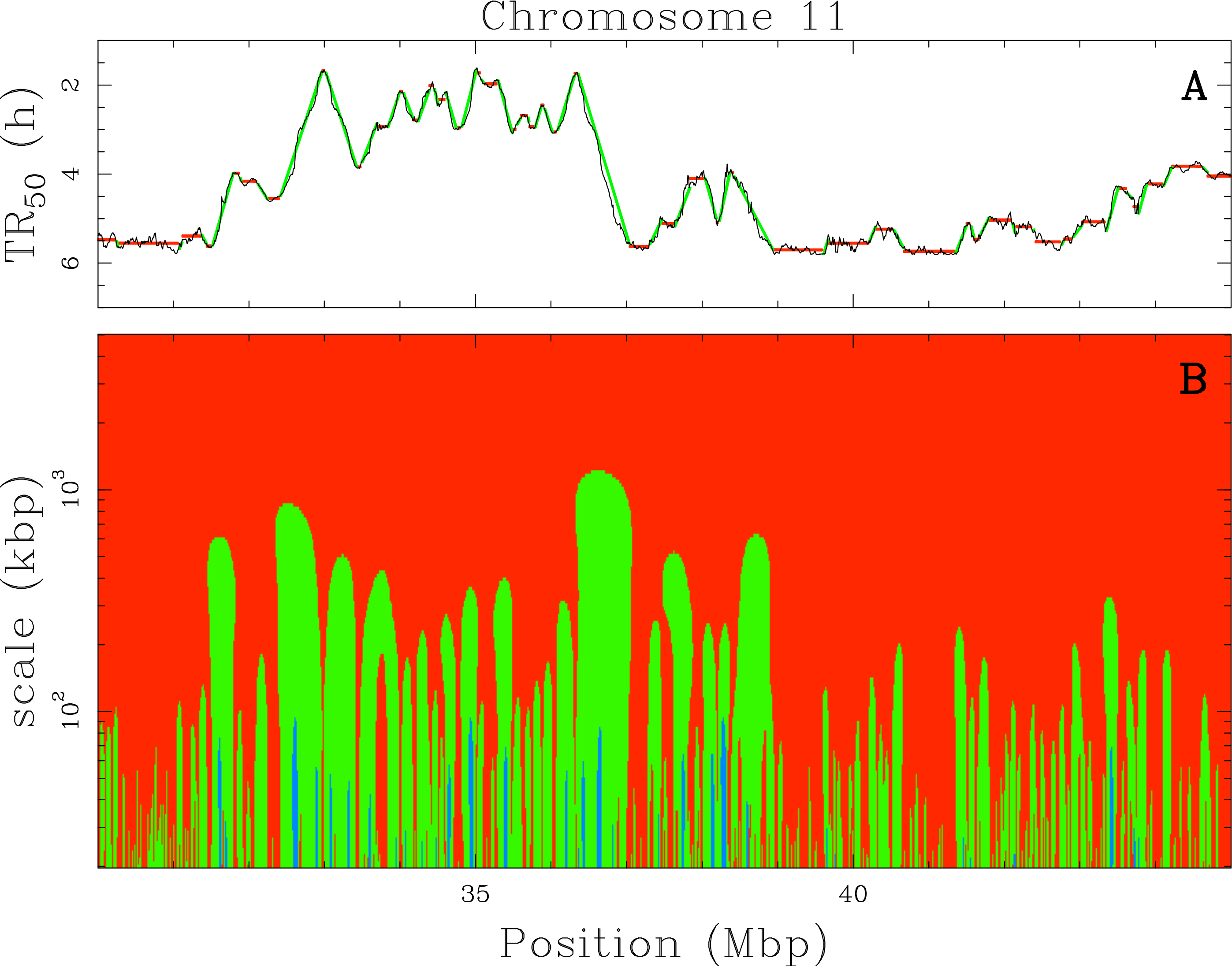

Chromosome 11
A
B
TR50 (h)
2
4
6
scale (kbp)
10^3
10^2
35
40
Position (Mbp)

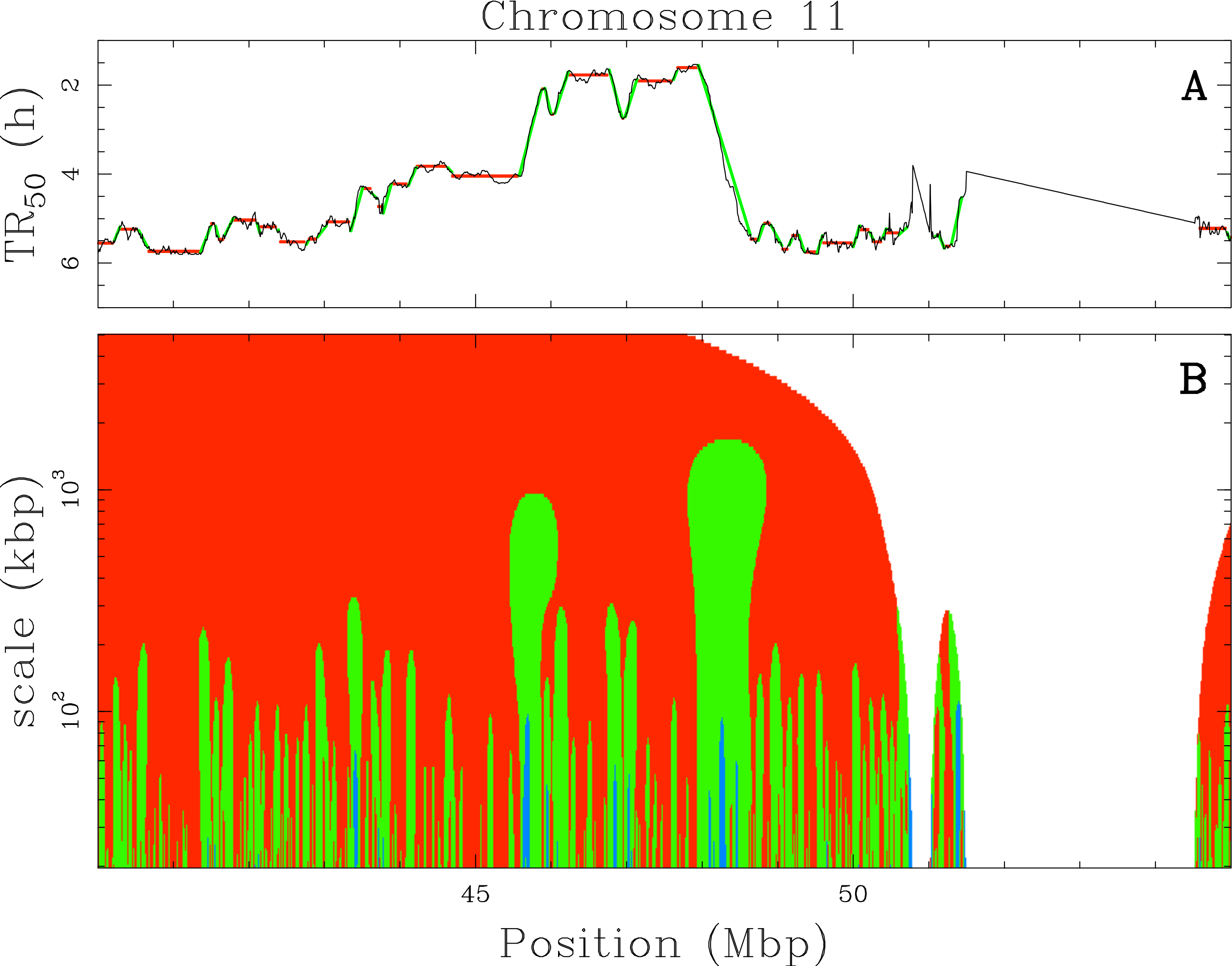

Chromosome 11
A
TR_{50} (h)
2
4
6
B
scale (kbp)
10^3
10^2
45
50
Position (Mbp)

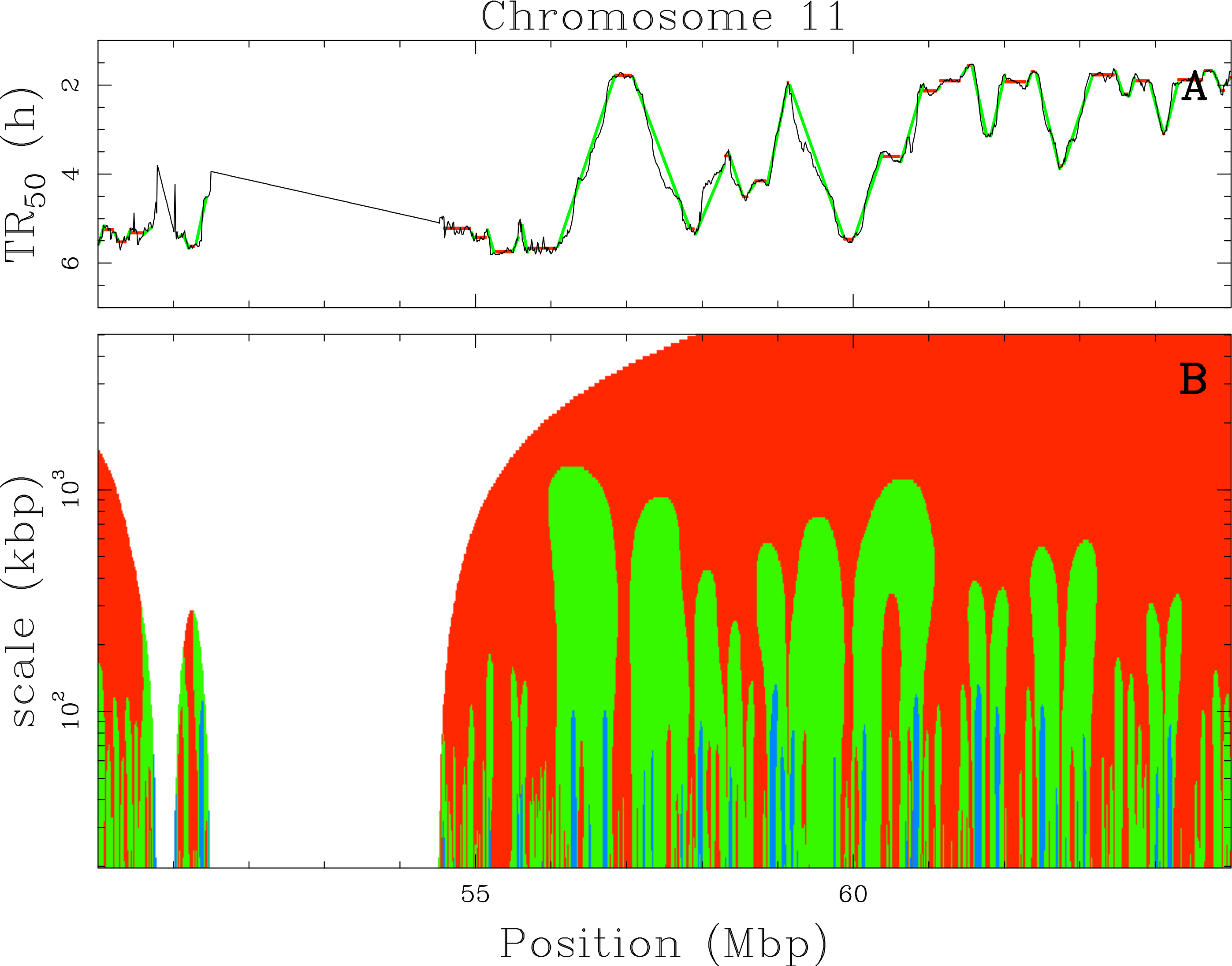

Chromosome 11
A
B
TR_{50} (h)
2
4
6
scale (kbp)
10^3
10^2
55
60
Position (Mbp)

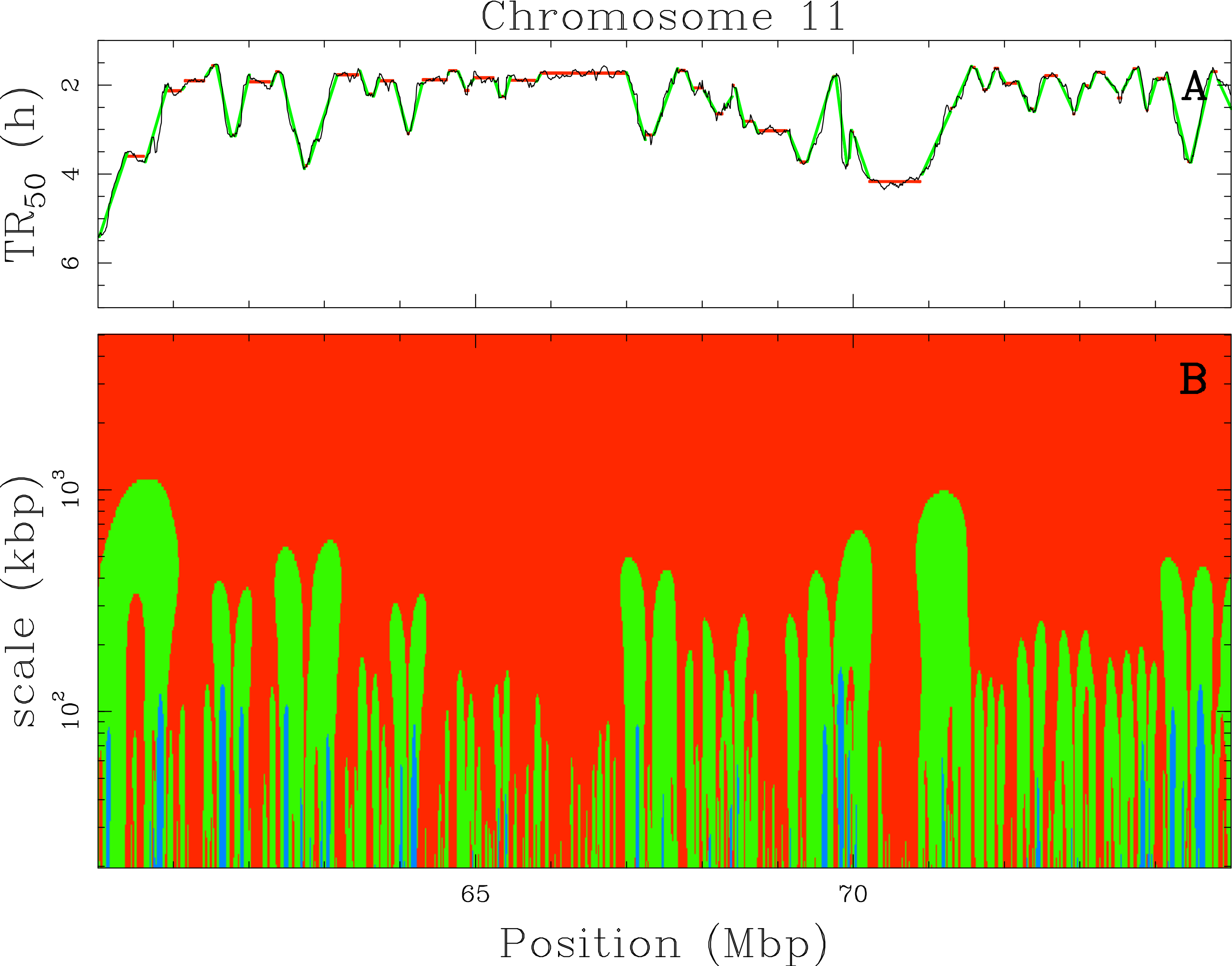

Chromosome 11
TR50 (h)
2
4
6
A
B
scale (kbp)
10^3
10^2
65
70
Position (Mbp)

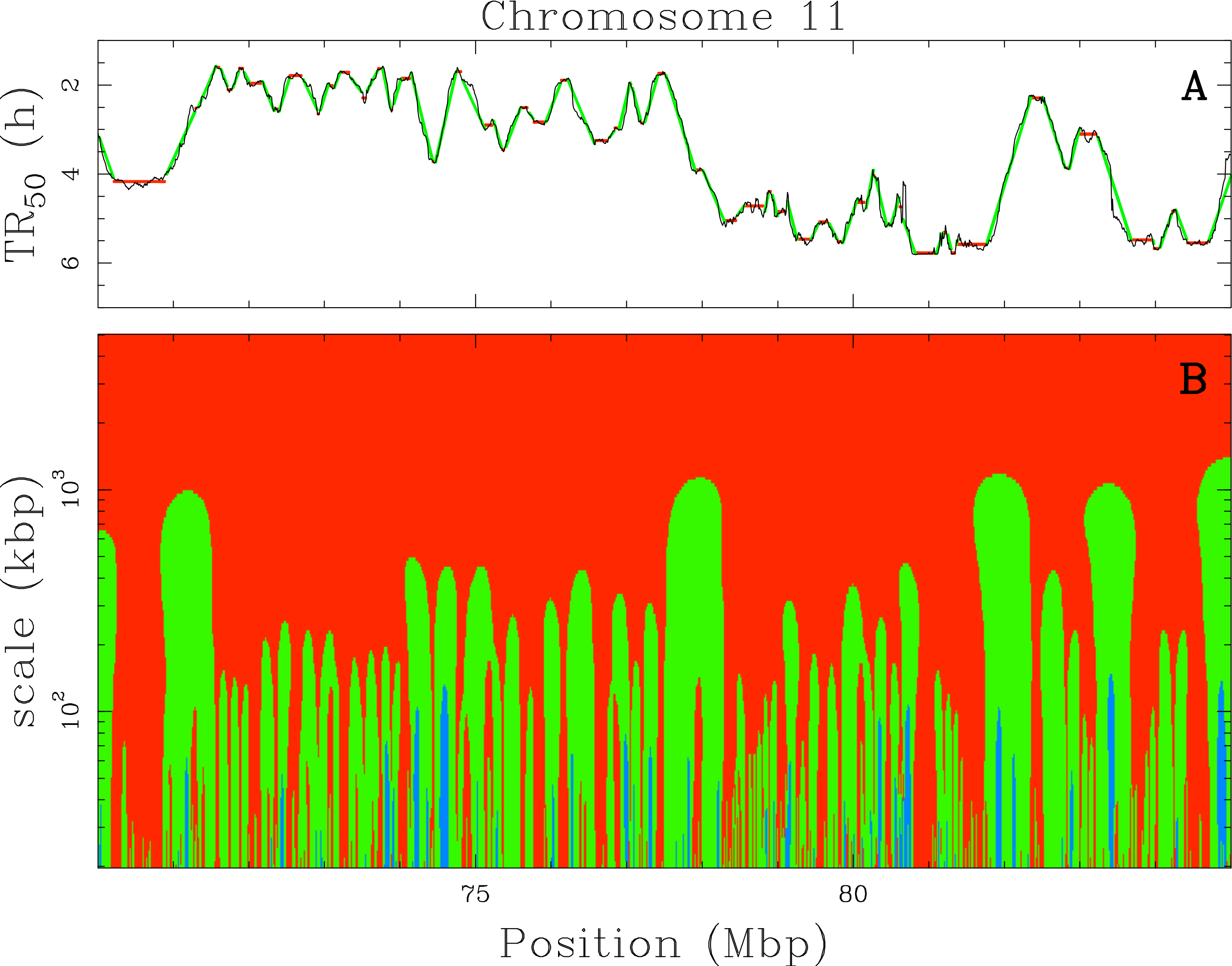

Chromosome 11
A
B
TR$_{50}$ (h)
2
4
6
scale (kbp)
10^3
10^2
75
80
Position (Mbp)

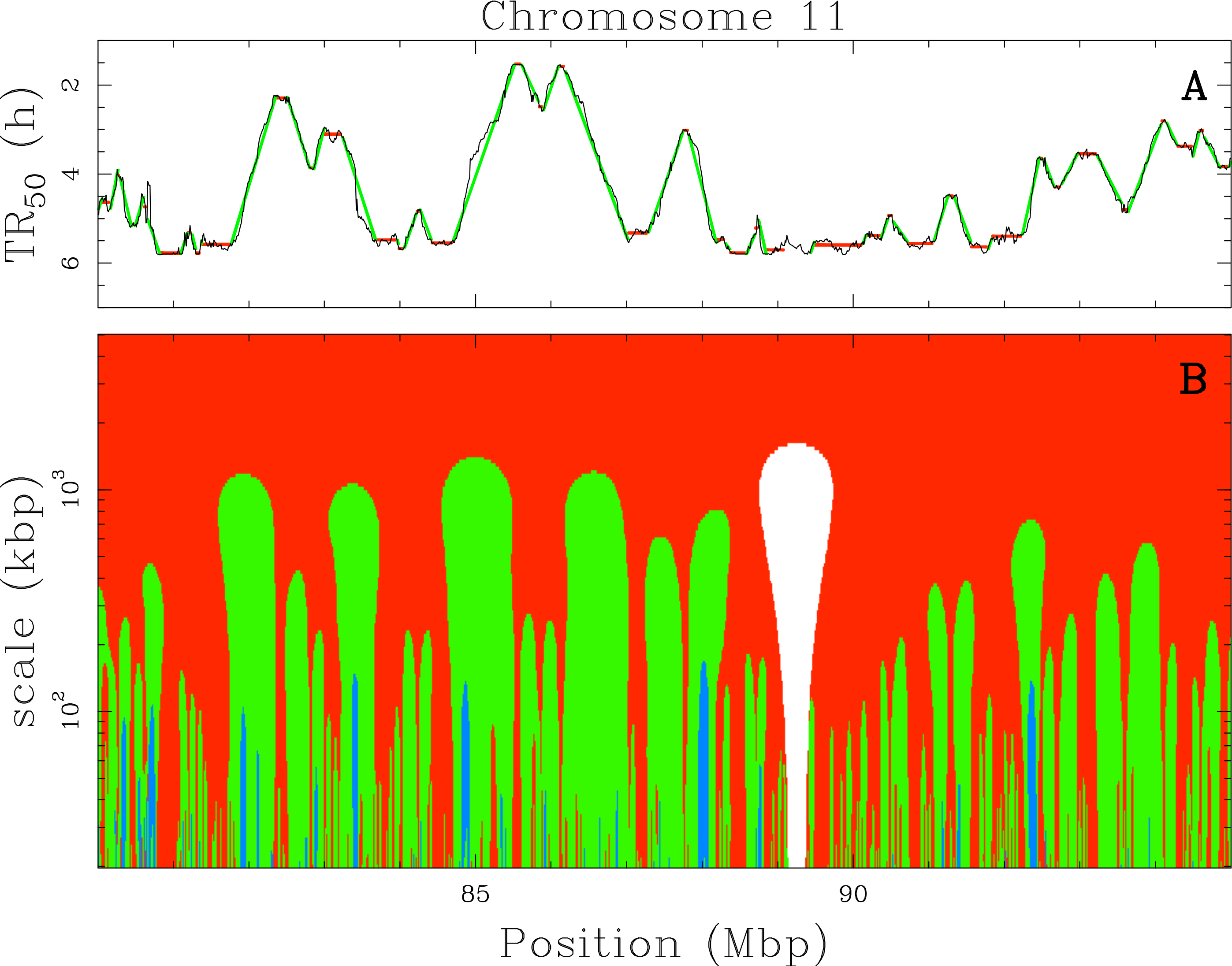

Chromosome 11
TR50 (h)
2
4
6
A
B
scale (kbp)
10^3
10^2
85
90
Position (Mbp)

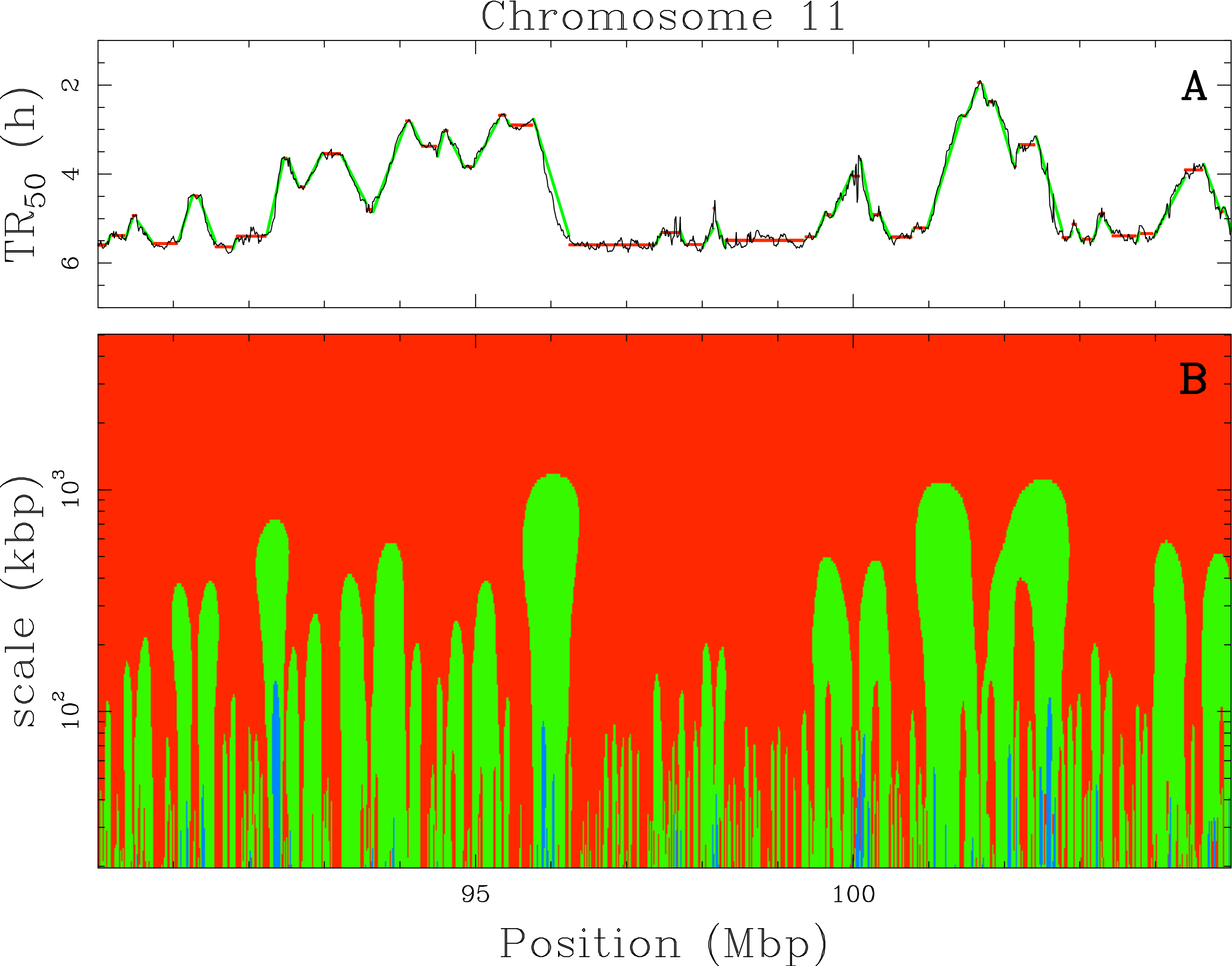

Chromosome 11
A
TR50 (h)
2
4
6
B
scale (kbp)
10^3
10^2
95
100
Position (Mbp)

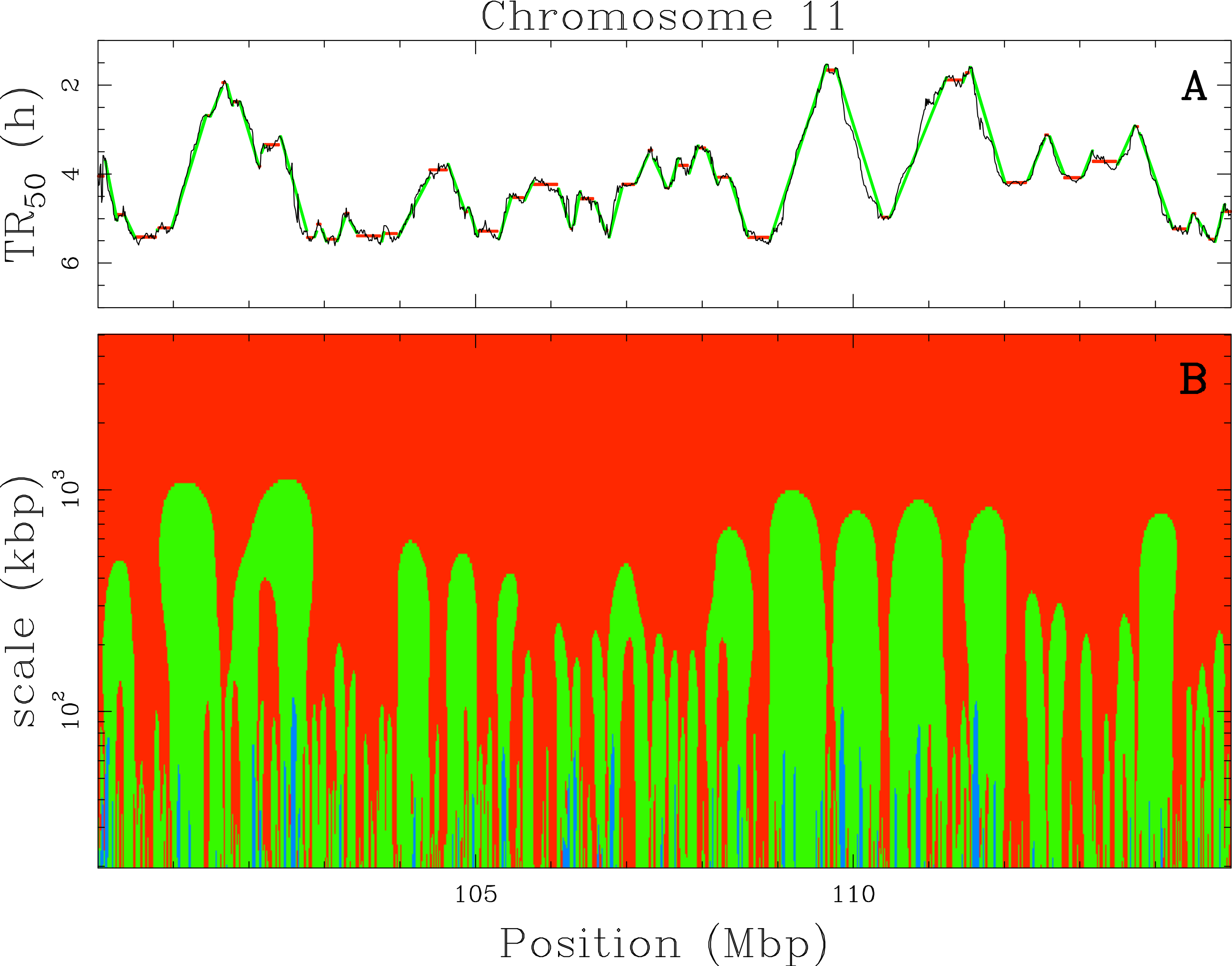

Chromosome 11
A
B
TR50 (h)
2
4
6
scale (kbp)
10^3
10^2
105
110
Position (Mbp)

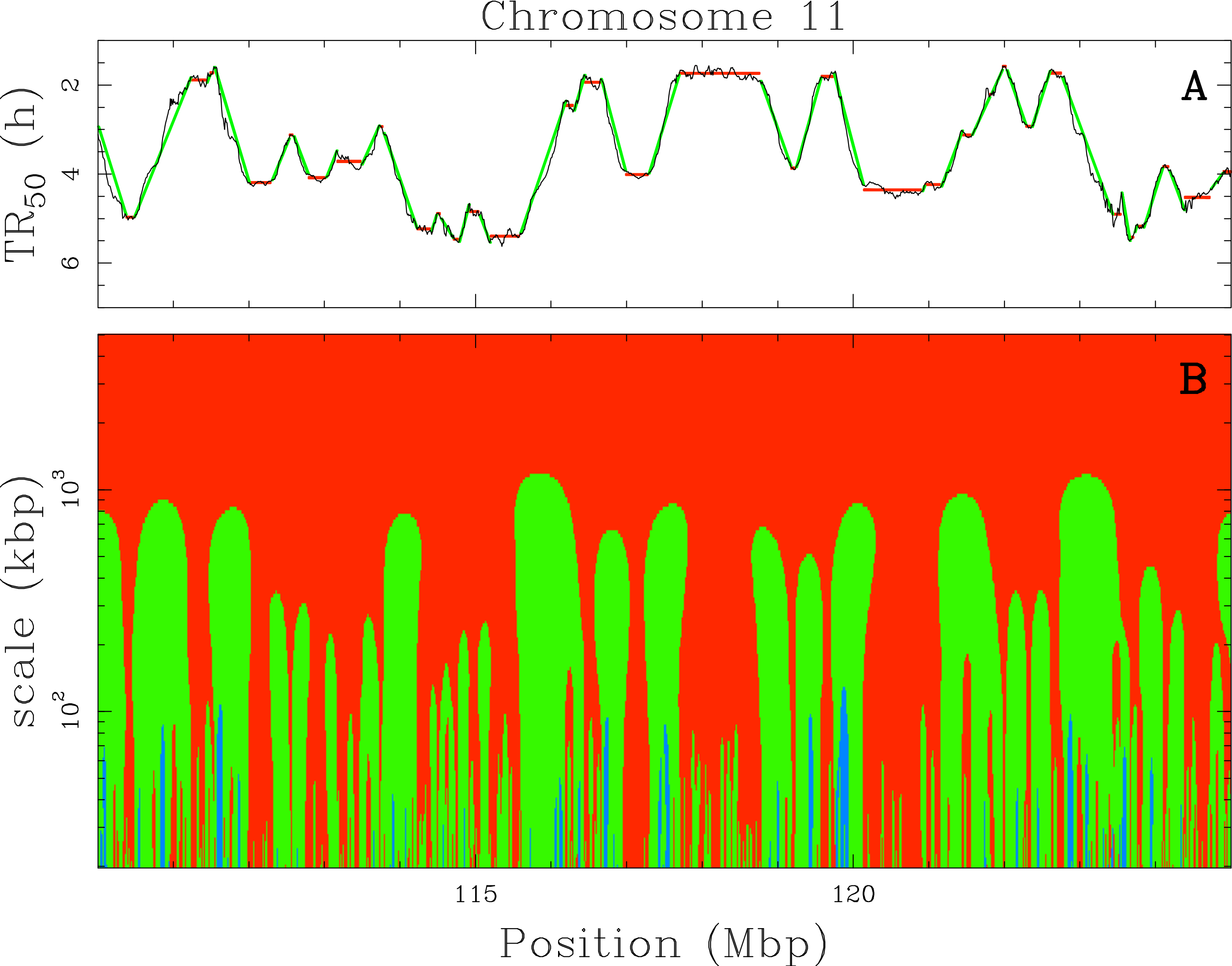

Chromosome 11
TR50 (h)
2
4
6
A
B
scale (kbp)
10^3
10^2
115
120
Position (Mbp)

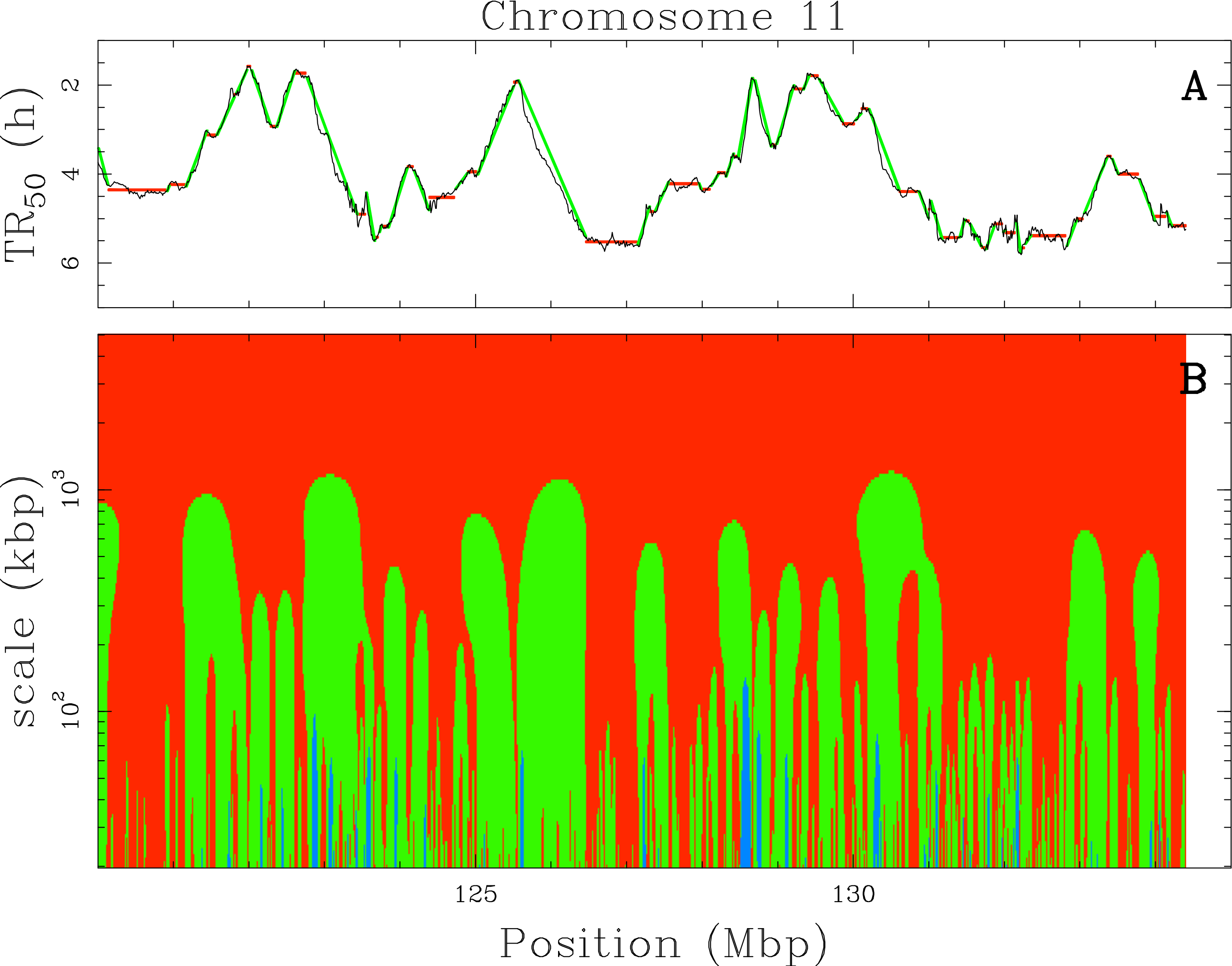

Chromosome 11
A
B
TR_{50} (h)
2
4
6
scale (kbp)
10^3
10^2
125
130
Position (Mbp)

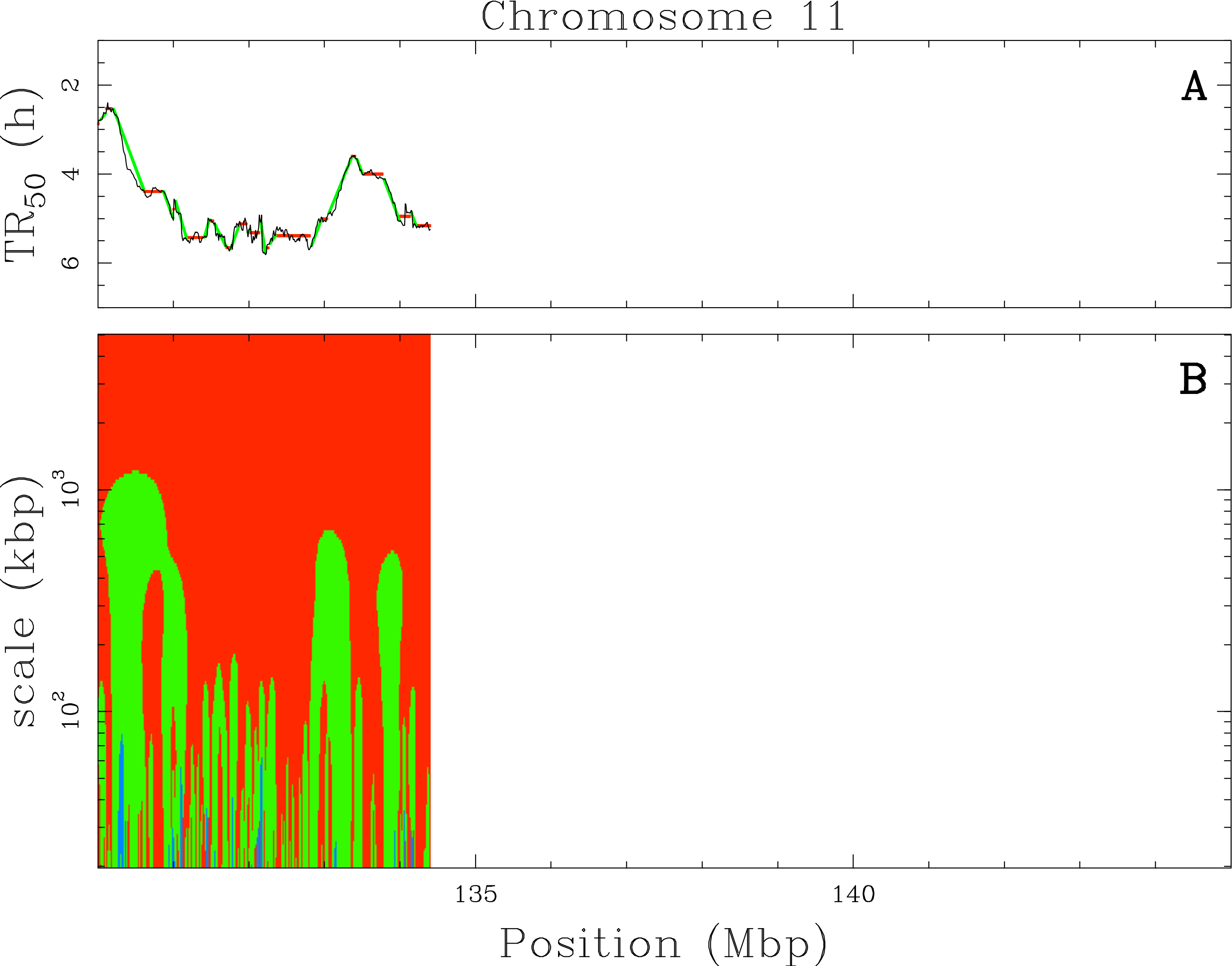

Chromosome 11
A
B
TR50 (h)
2
4
6
scale (kbp)
10^3
10^2
135
140
Position (Mbp)

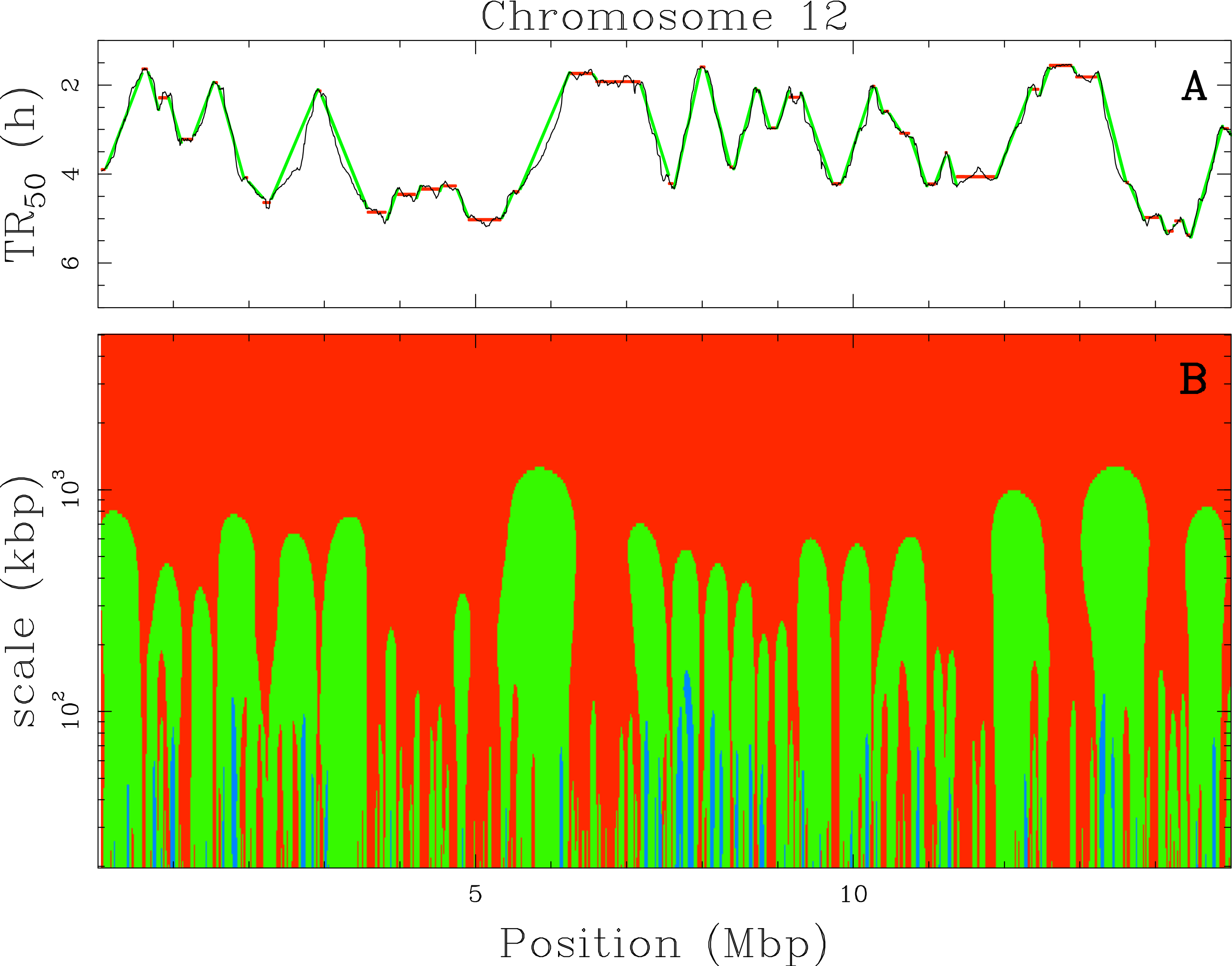

Chromosome 12
A
B
TR_{50} (h)
2
4
6
scale (kbp)
10^3
10^2
5
10
Position (Mbp)

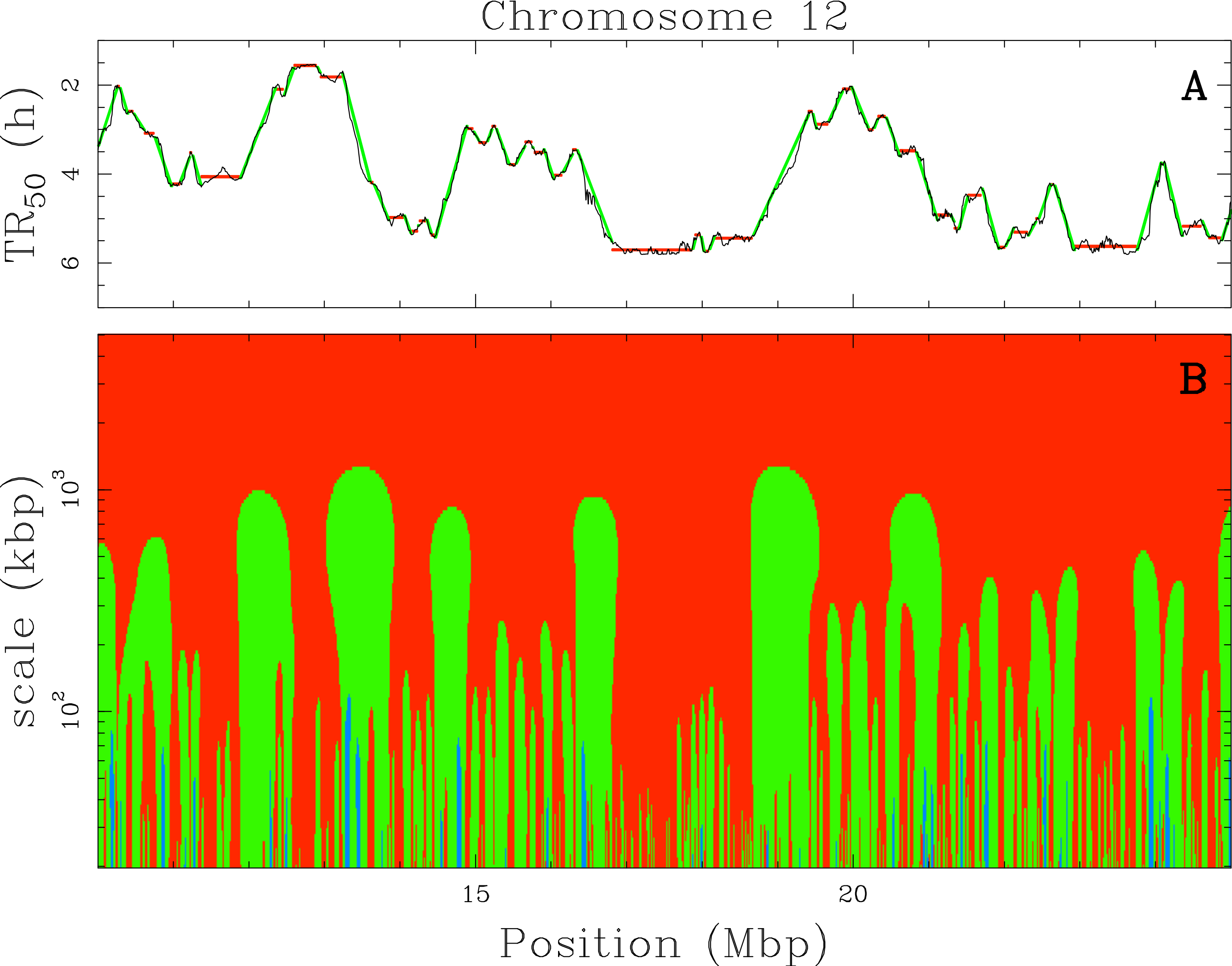

Chromosome 12
A
TR50 (h)
2
4
6
B
scale (kbp)
10^3
10^2
15
20
Position (Mbp)

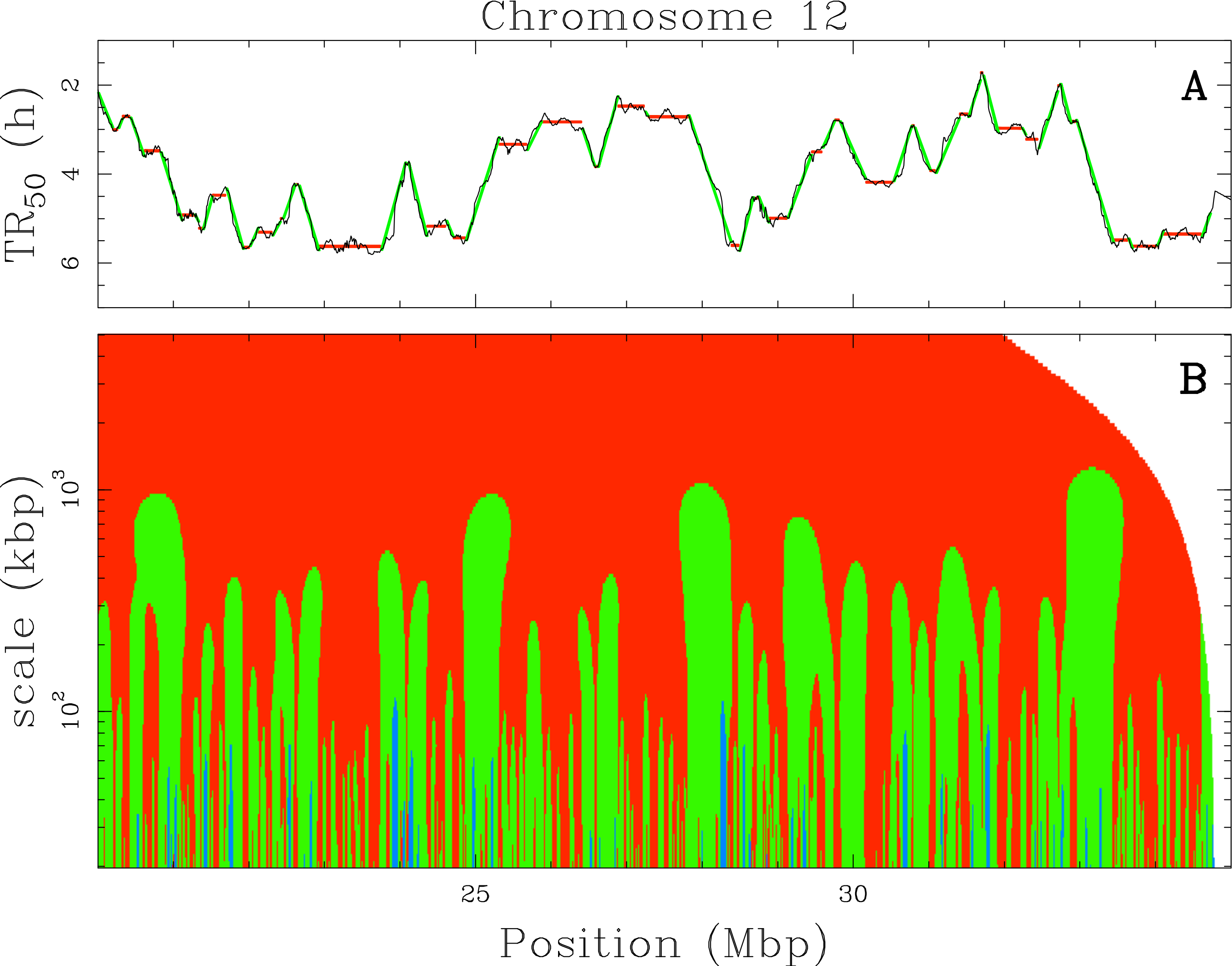

Chromosome 12
TR50 (h)
2
4
6
A
B
scale (kbp)
10^3
10^2
25
30
Position (Mbp)

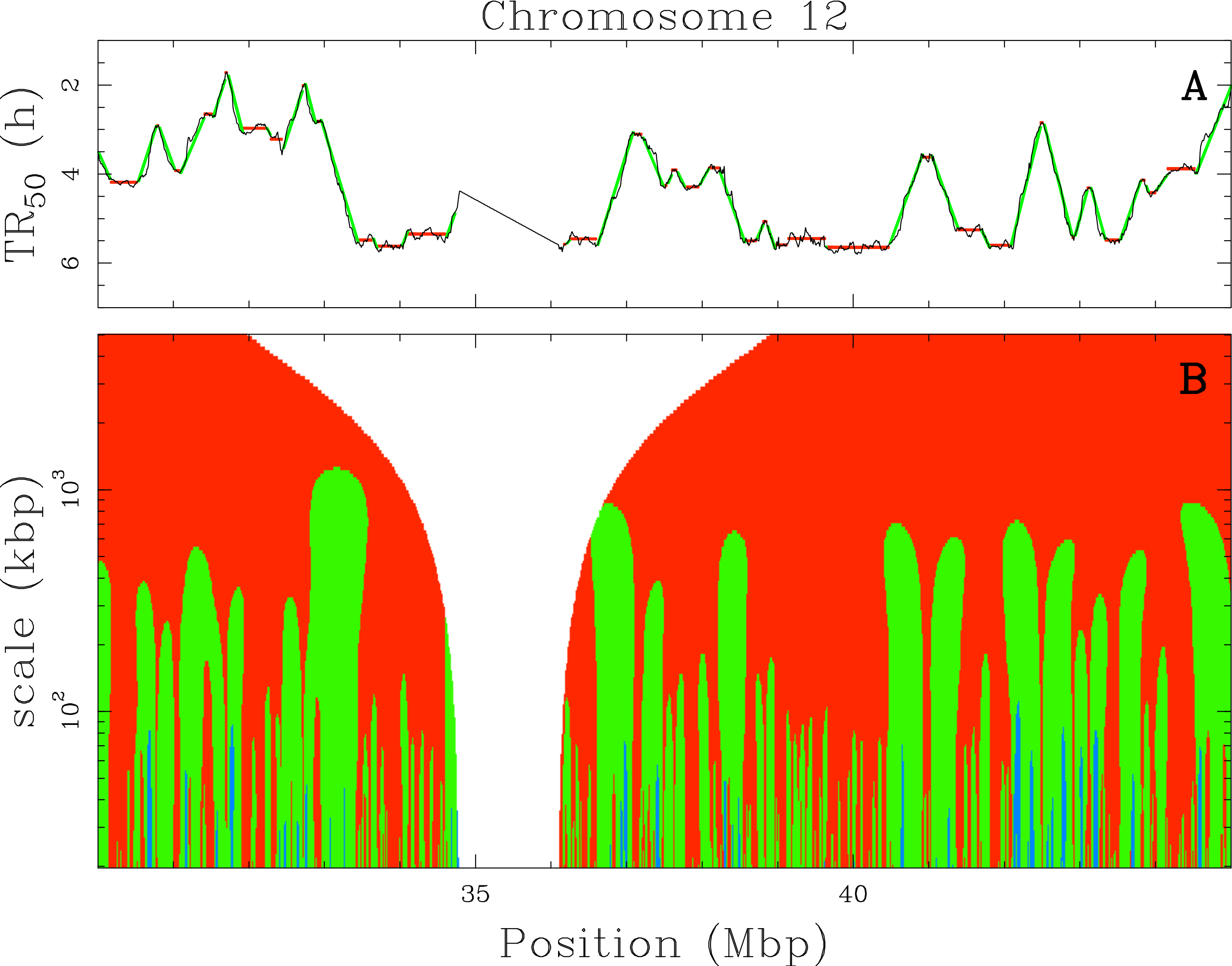
Chromosome 12
A
B
TR50 (h)
2
4
6
scale (kbp)
10^3
10^2
35
40
Position (Mbp)

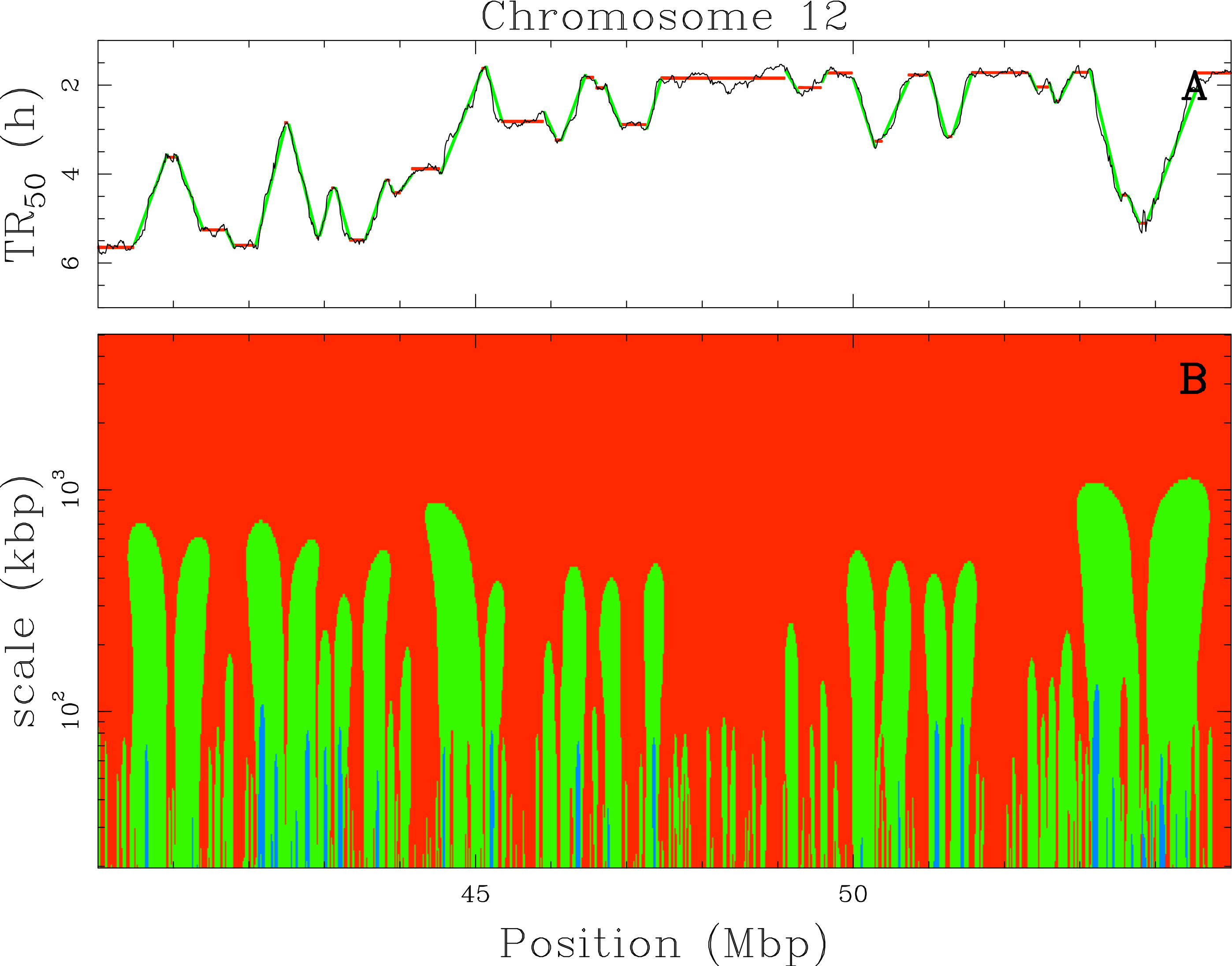

Chromosome 12
TR50 (h)
2
4
6
A
B
scale (kbp)
10^3
10^2
45
50
Position (Mbp)

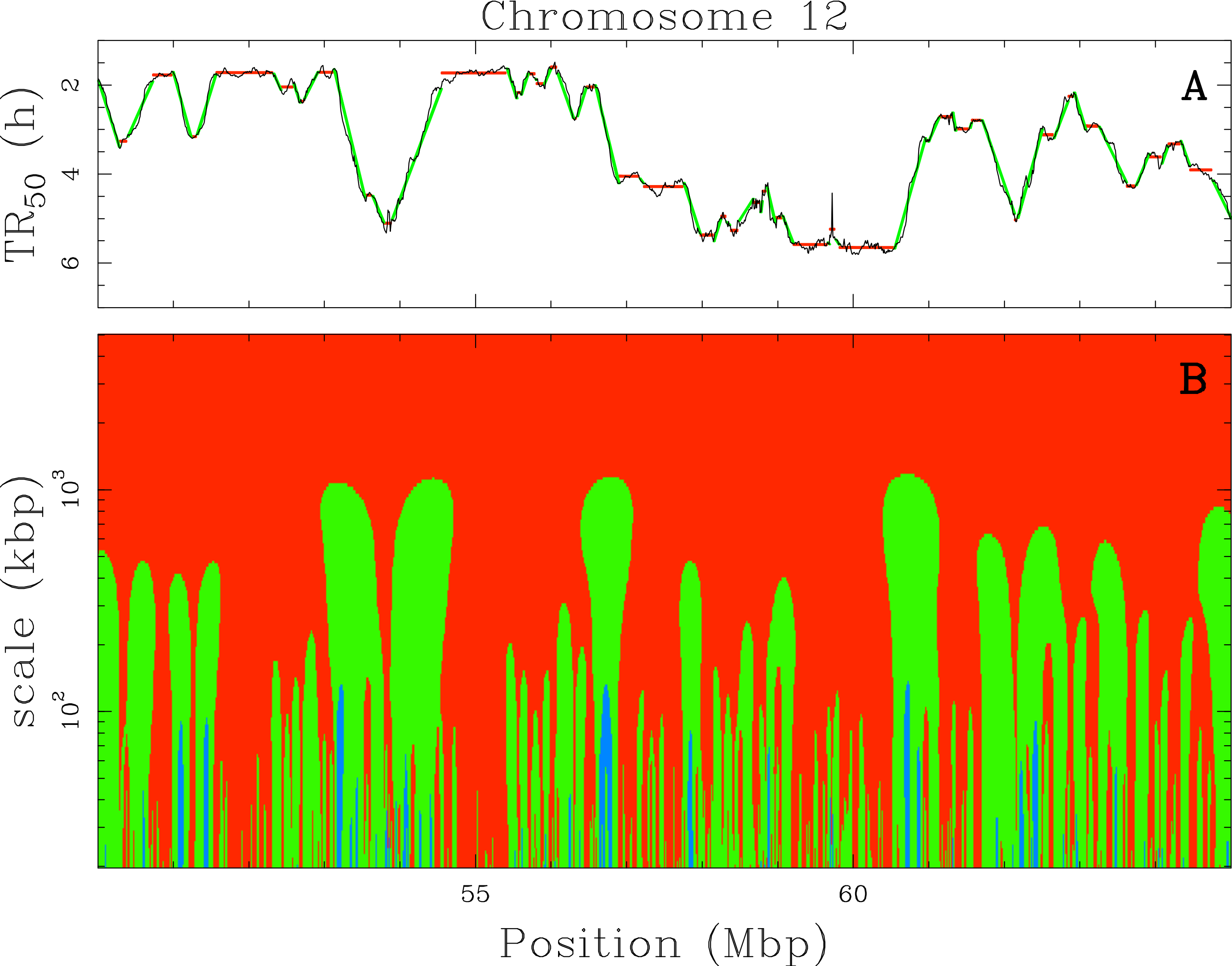

Chromosome 12
A
B
TR50 (h)
2
4
6
scale (kbp)
10^3
10^2
55
60
Position (Mbp)

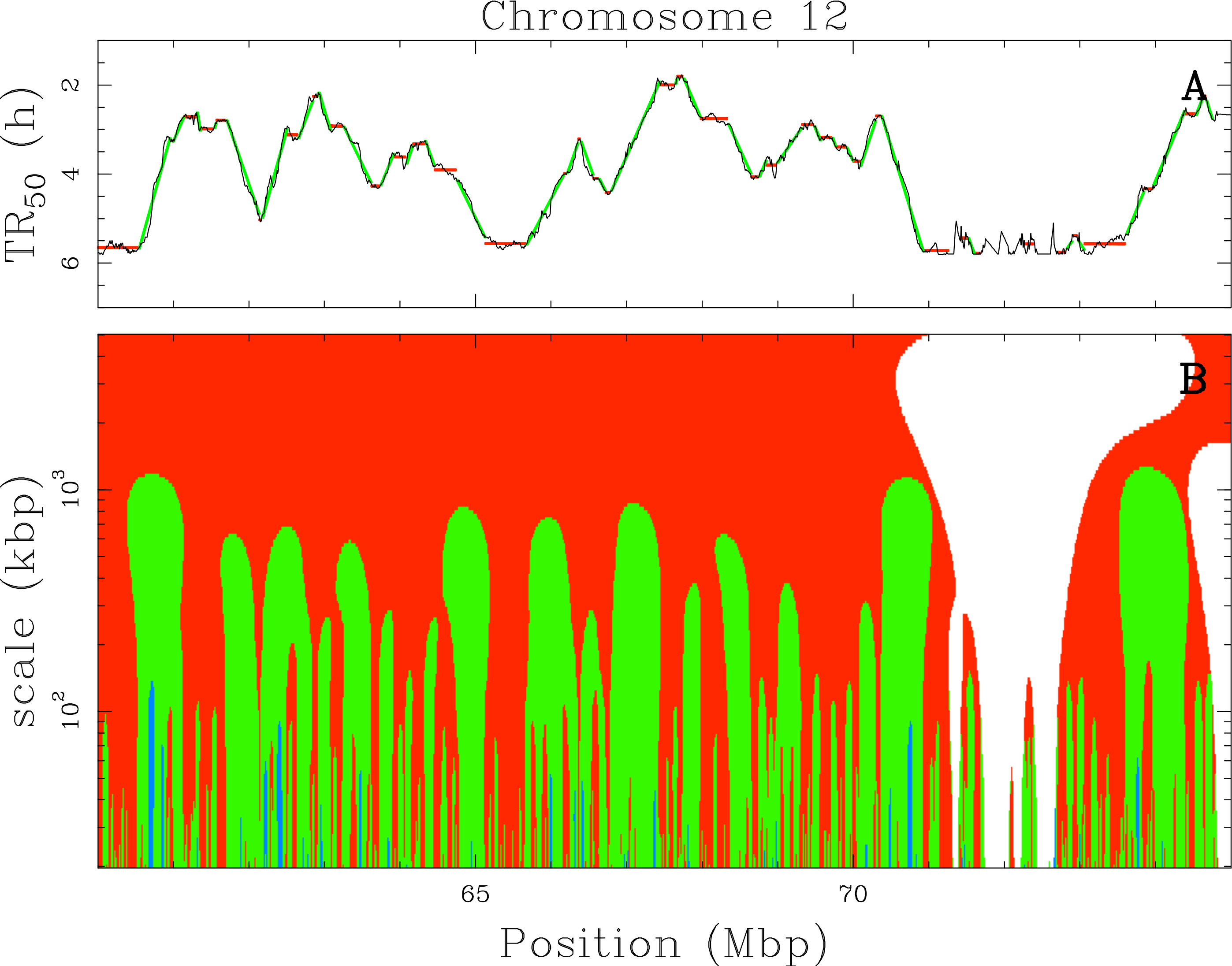

Chromosome 12
A
B
TR50 (h)
2
4
6
scale (kbp)
10^3
10^2
65
70
Position (Mbp)

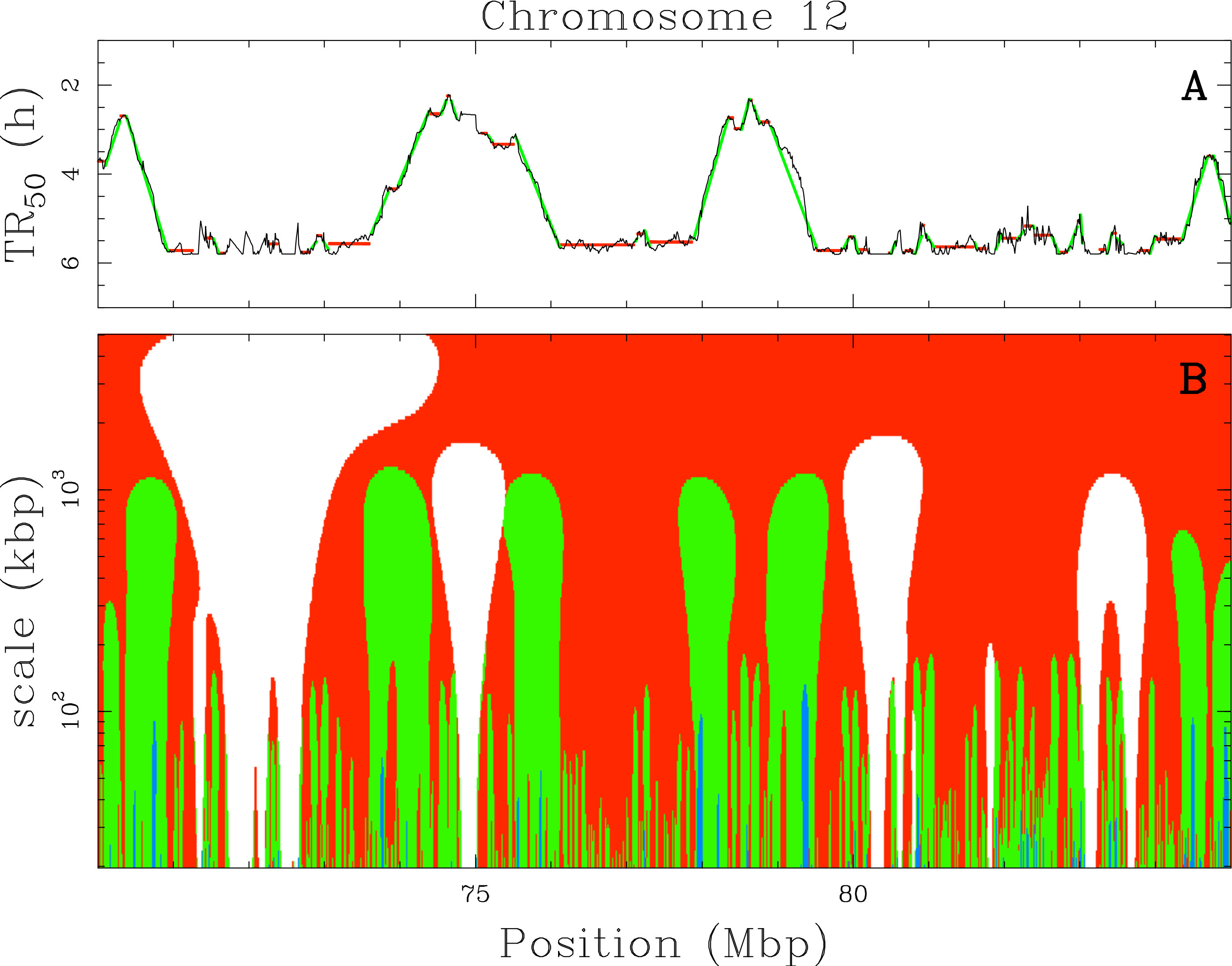
Chromosome 12
A
B
TR$_{50}$ (h)
2
4
6
scale (kbp)
10^3
10^2
75
80
Position (Mbp)

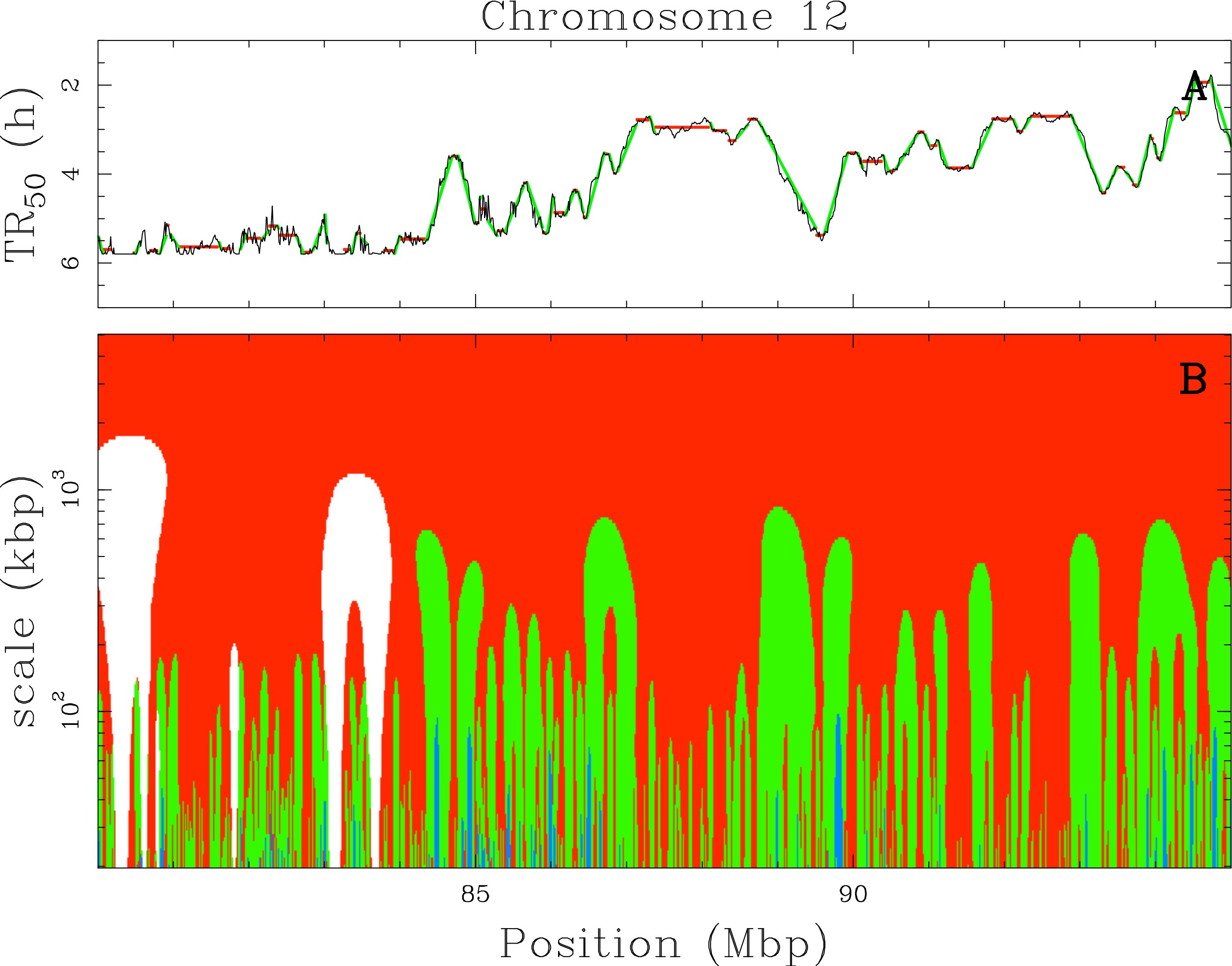

Chromosome 12
A
B
TR50 (h)
2
4
6
scale (kbp)
10^3
10^2
85
90
Position (Mbp)

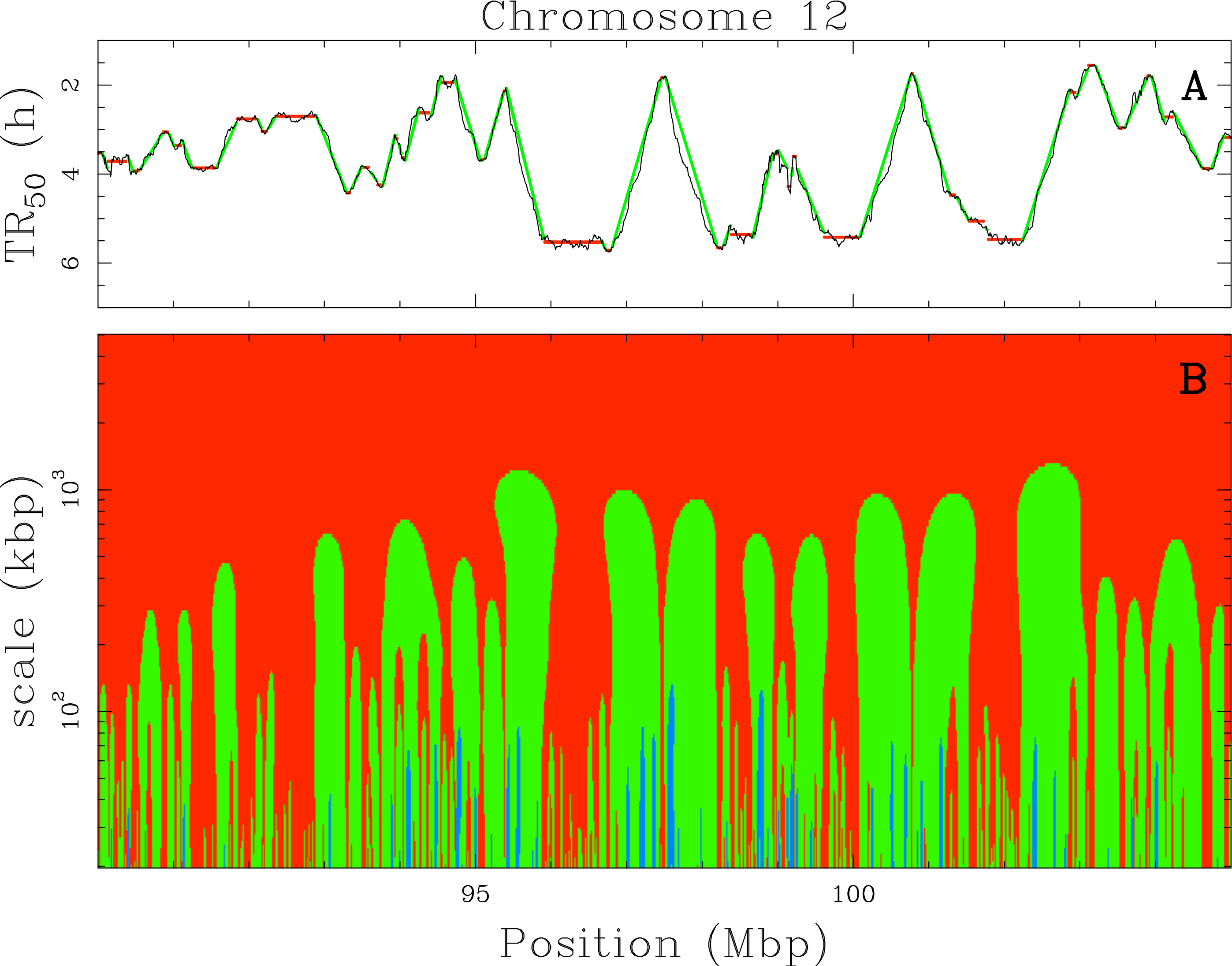

Chromosome 12
A
TR50 (h)
2
4
6
B
scale (kbp)
10^3
10^2
95
100
Position (Mbp)

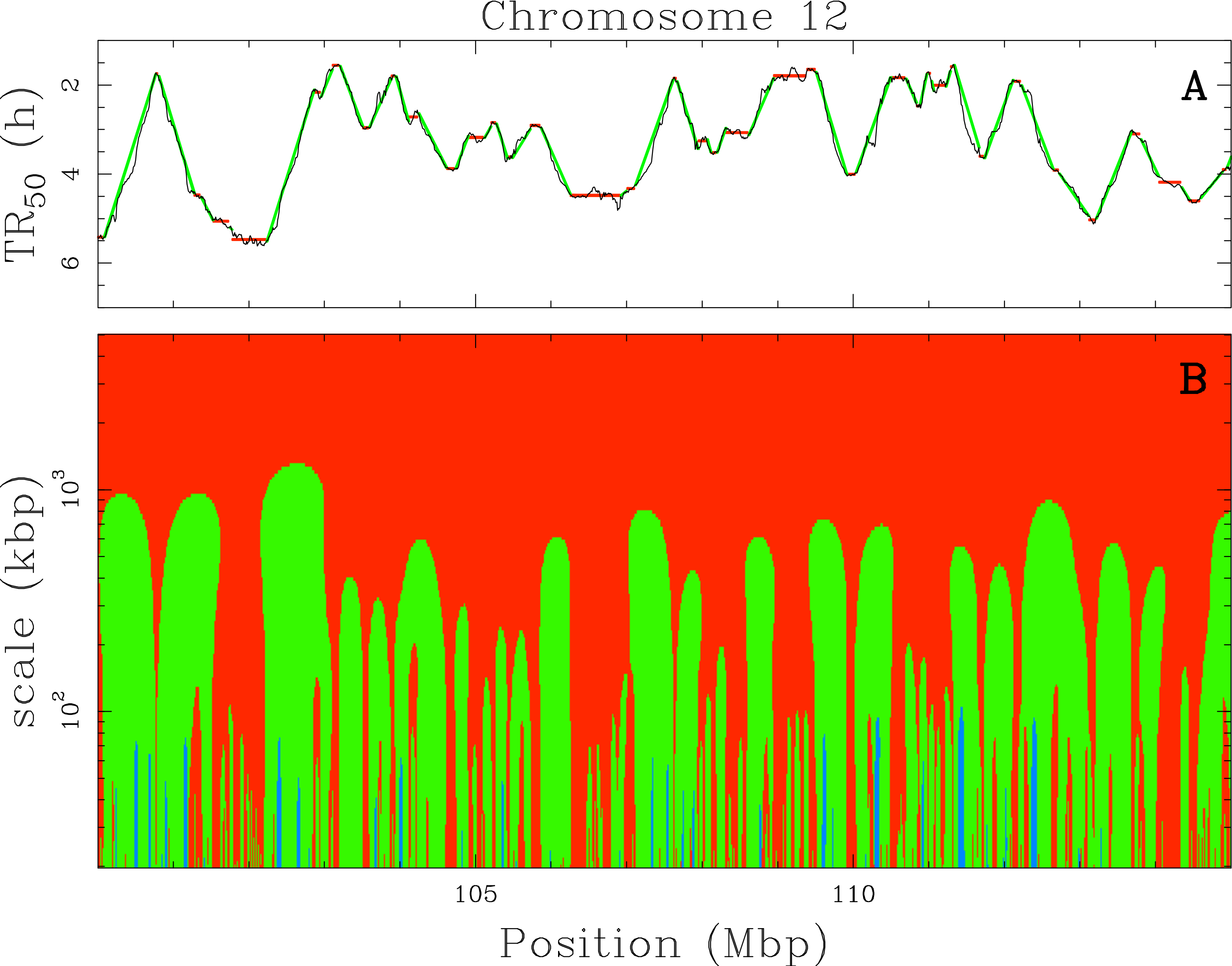

Chromosome 12
A
B
TR50 (h)
2
4
6
scale (kbp)
10^3
10^2
105
110
Position (Mbp)

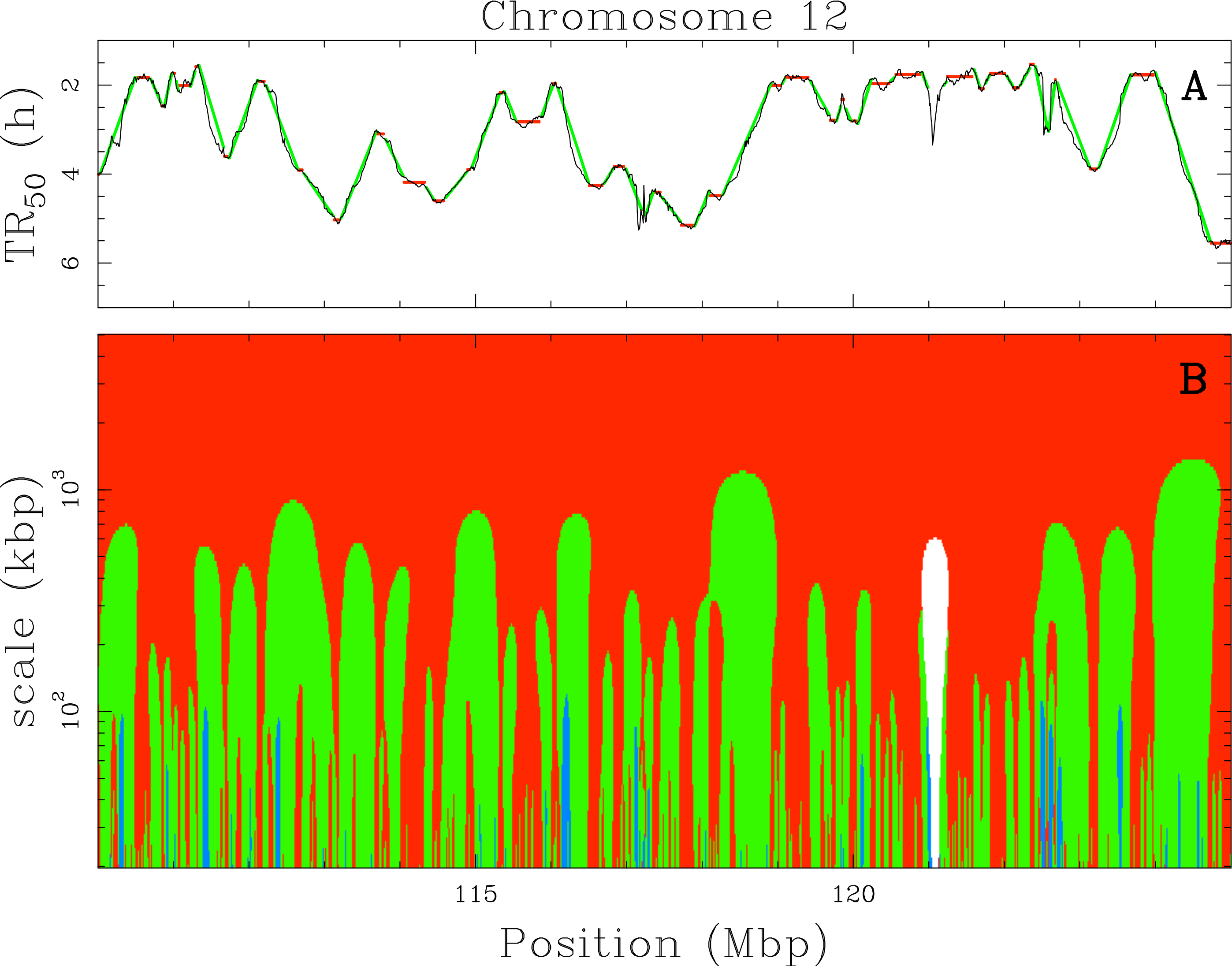
Chromosome 12
A
B
TR50 (h)
2
4
6
scale (kbp)
10^3
10^2
115
120
Position (Mbp)

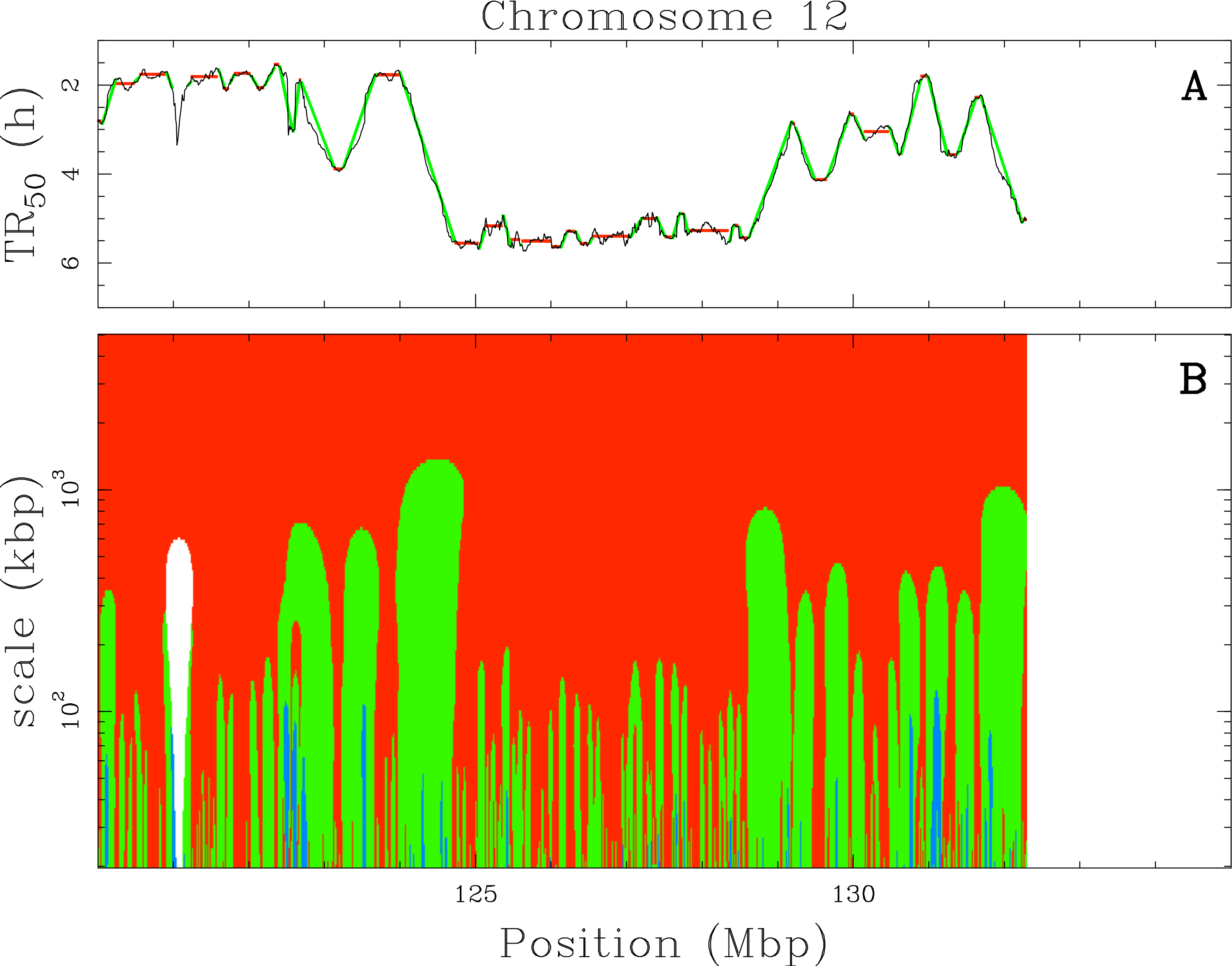

Chromosome 12
A
TR50 (h)
2
4
6
B
scale (kbp)
10^3
10^2
125
130
Position (Mbp)

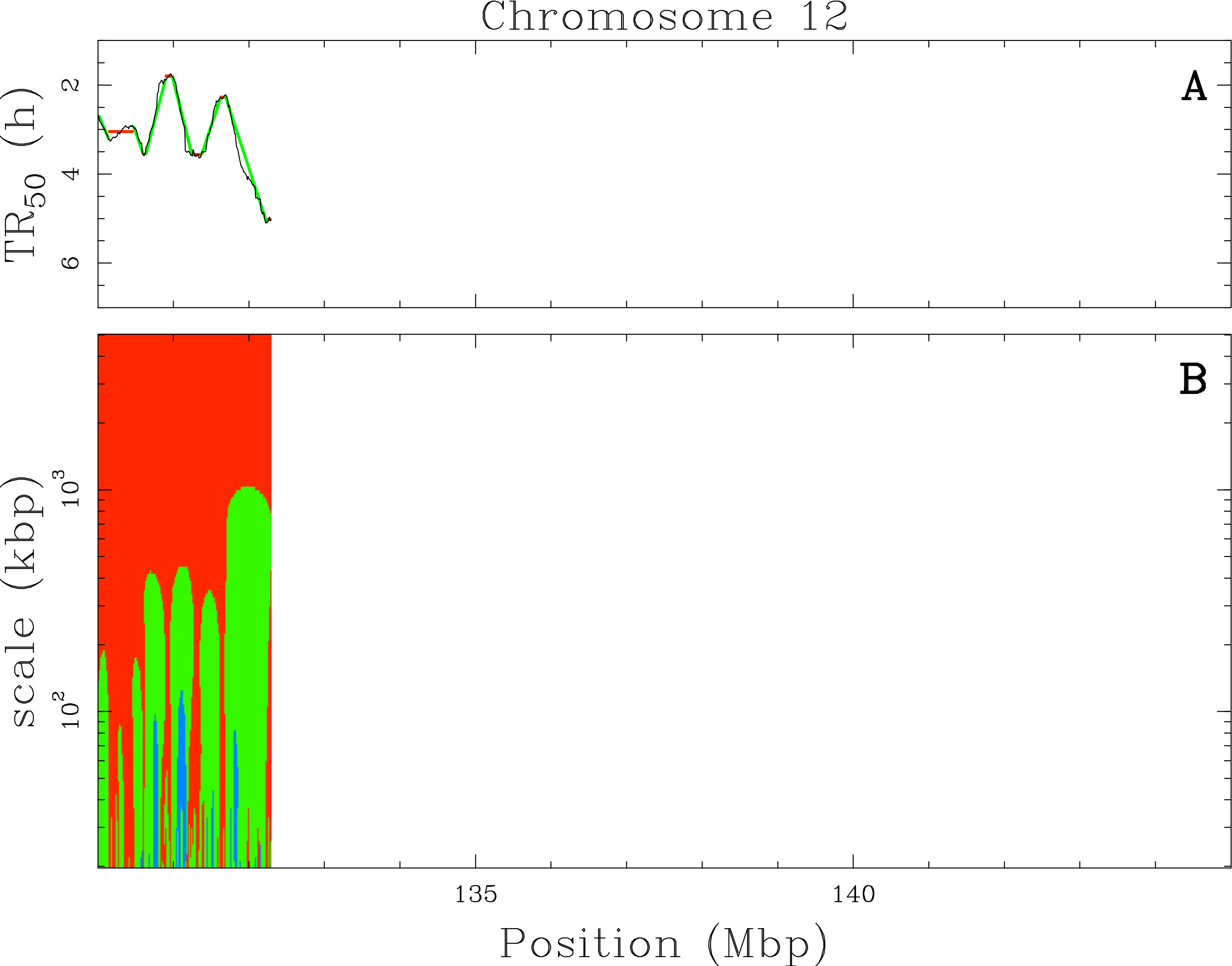

Chromosome 12
A
B
TR50 (h)
2
4
6
scale (kbp)
10^3
10^2
135
140
Position (Mbp)

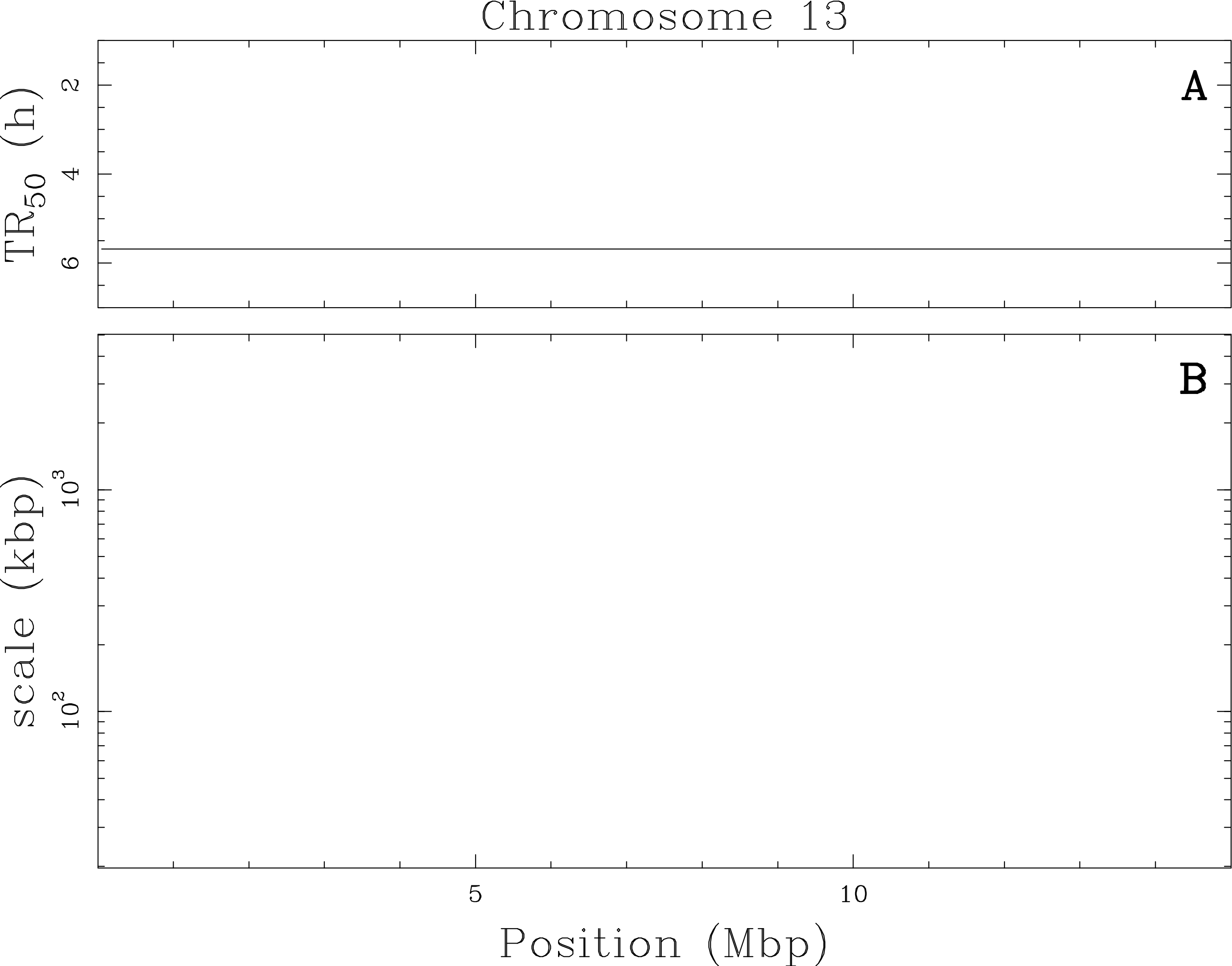

Chromosome 13
A
B
TR_{50} (h)
2
4
6
scale (kbp)
10^3
10^2
5
10
Position (Mbp)

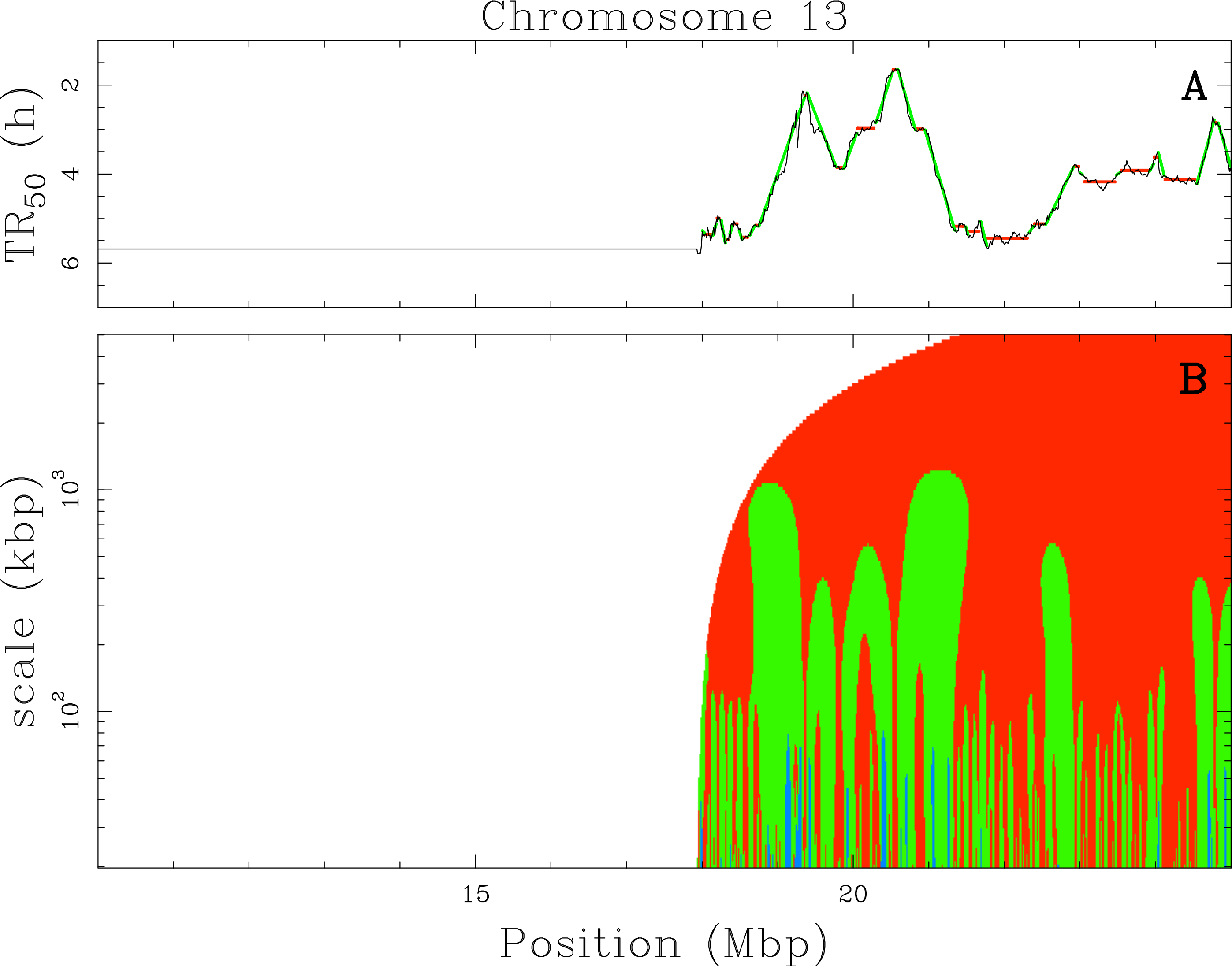

Chromosome 13
A
B
TR50 (h)
2
4
6
scale (kbp)
10^3
10^2
15
20
Position (Mbp)

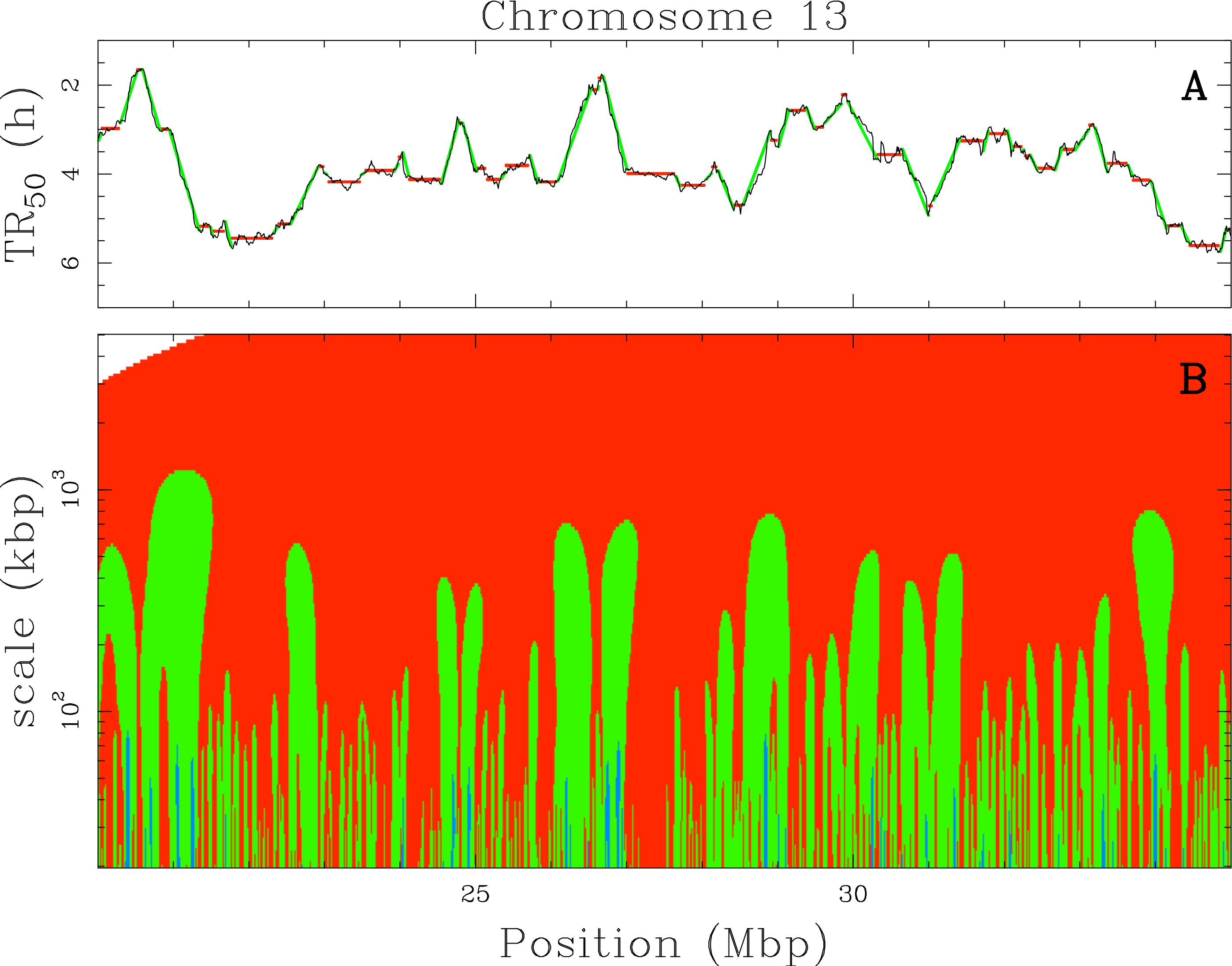

Chromosome 13
A
B
TR_{50} (h)
2
4
6
scale (kbp)
10^3
10^2
25
30
Position (Mbp)

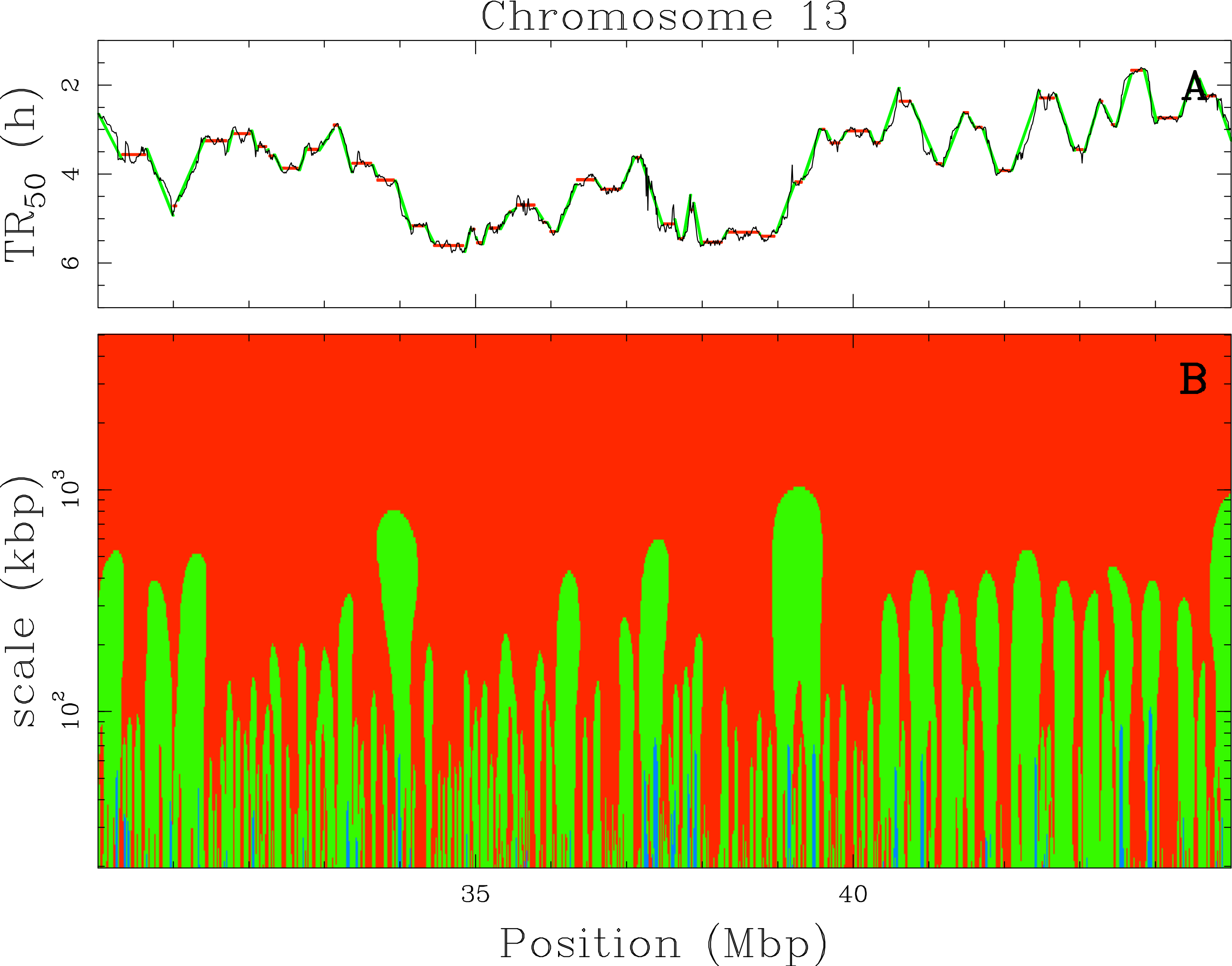

Chromosome 13
TR50 (h)
2
4
6
A
B
scale (kbp)
10^3
10^2
35
40
Position (Mbp)

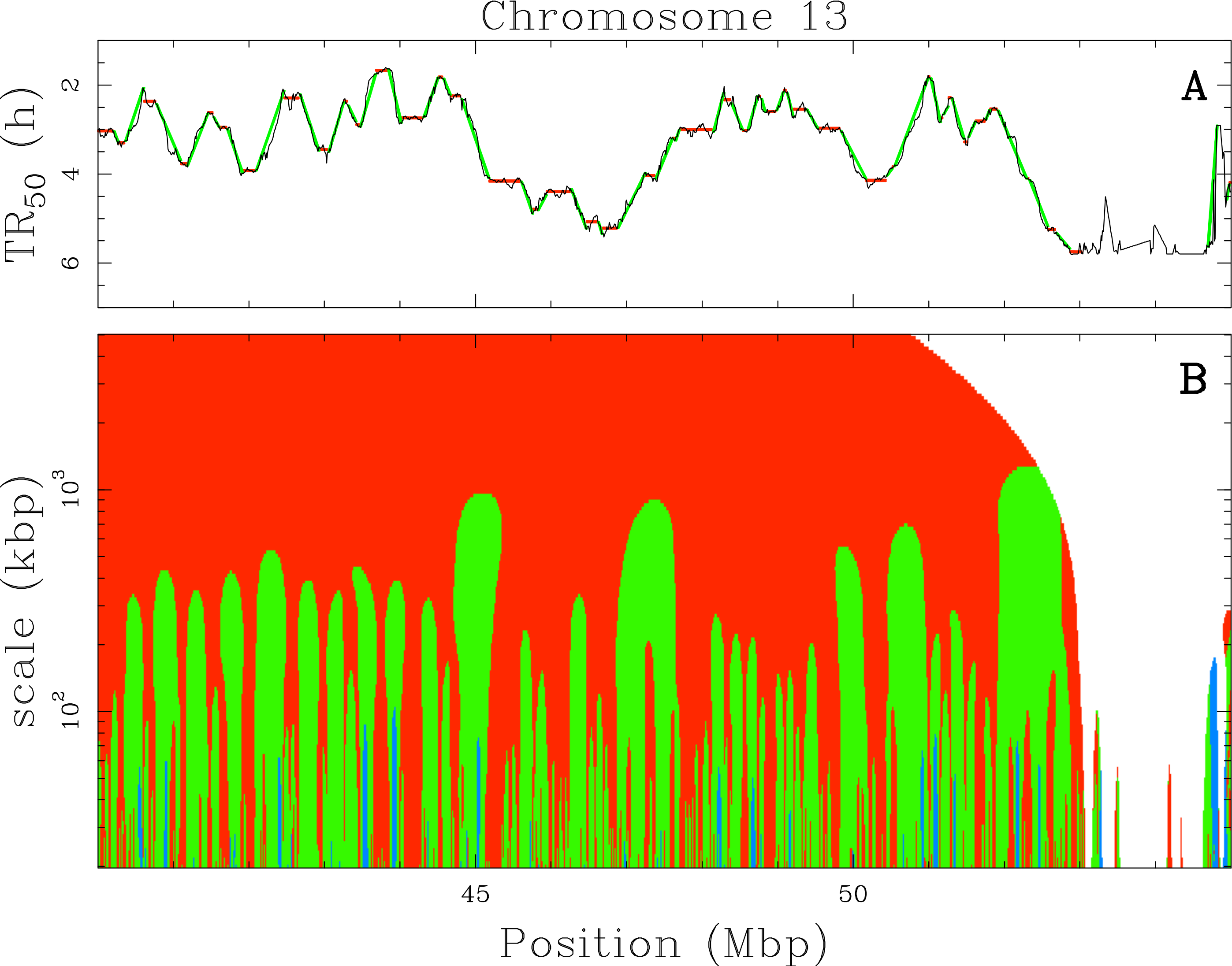

Chromosome 13
A
B
TR50 (h)
2
4
6
scale (kbp)
10^3
10^2
45
50
Position (Mbp)

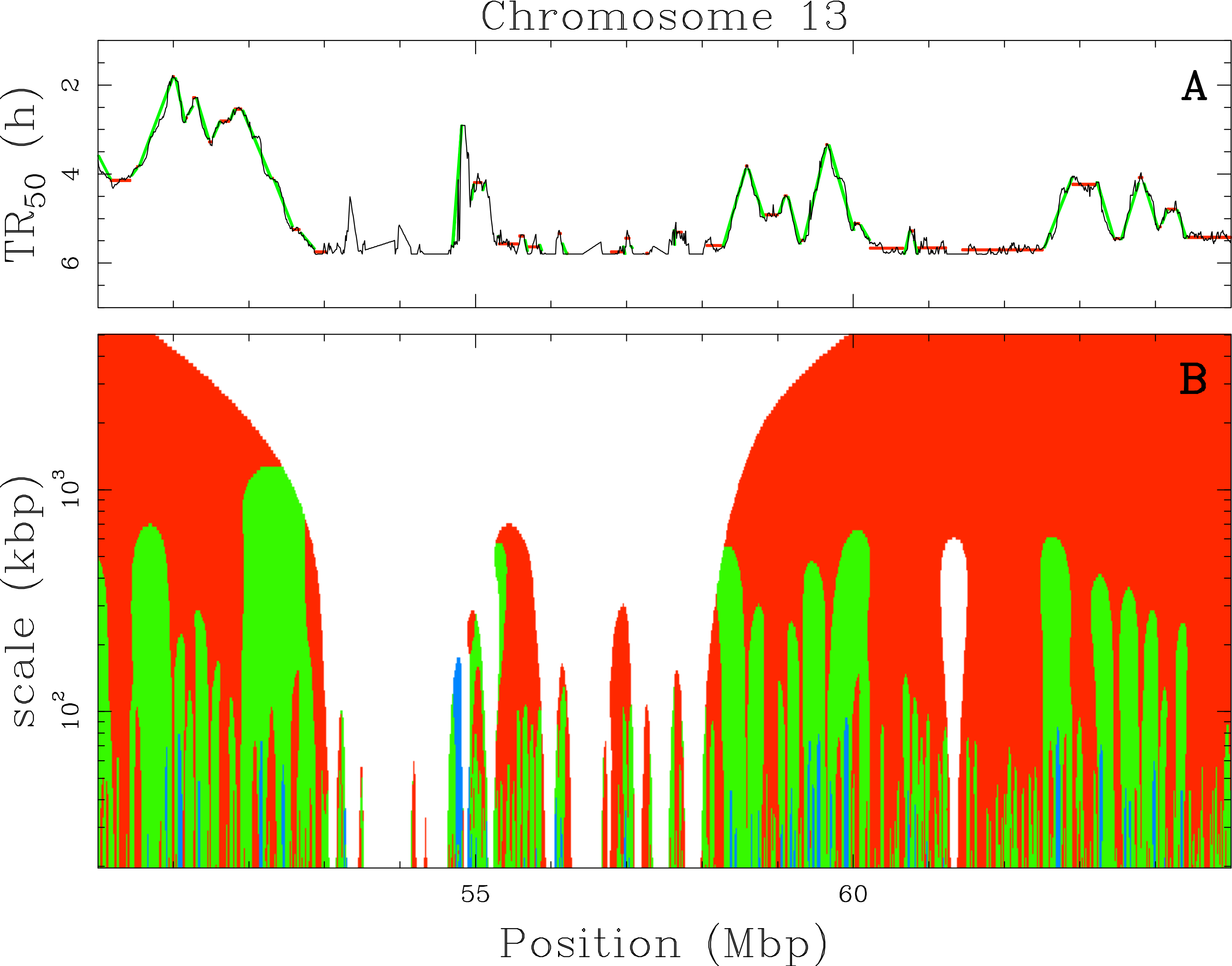

Chromosome 13
A
TR_{50} (h)
2
4
6
B
scale (kbp)
10^3
10^2
55
60
Position (Mbp)

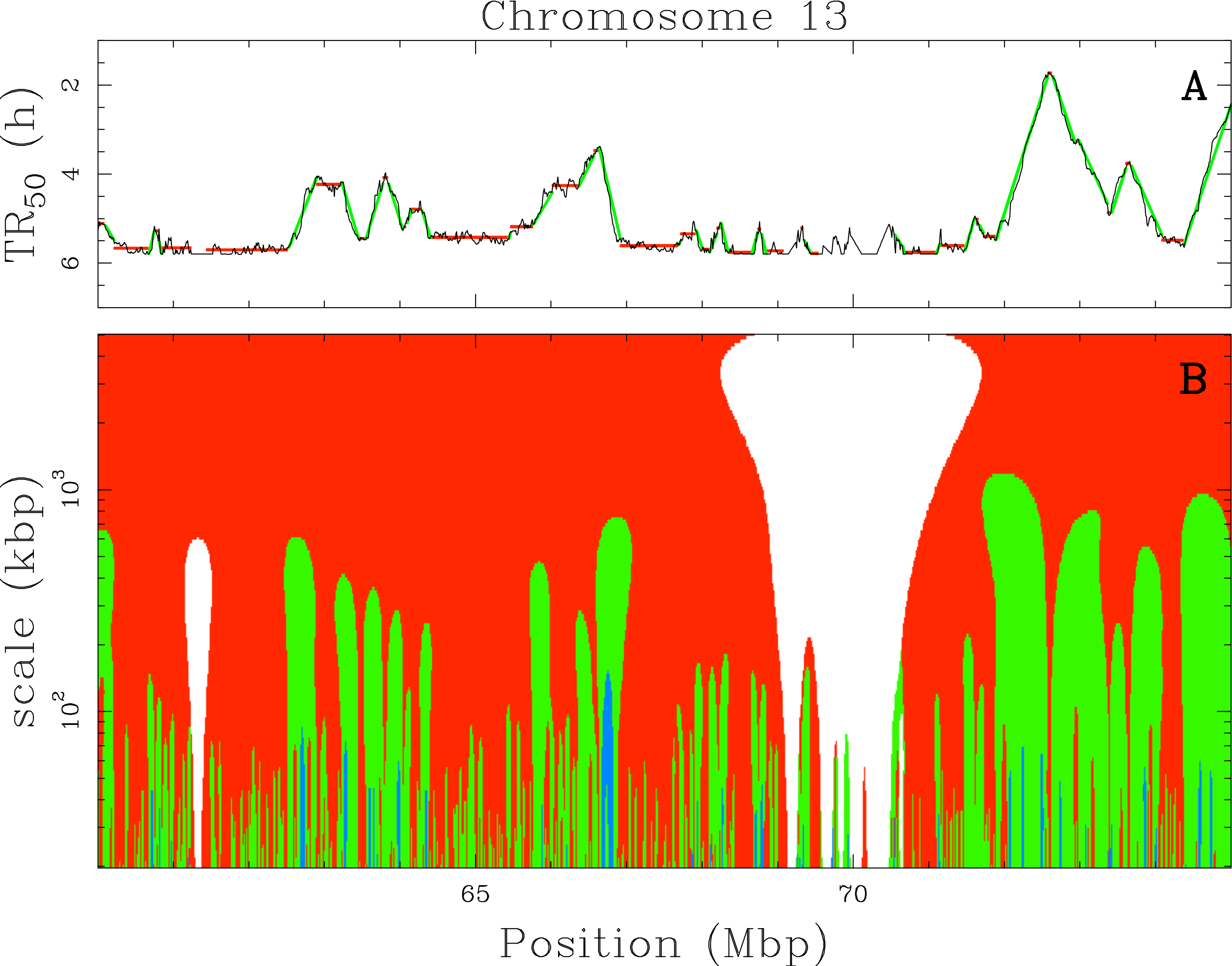
Chromosome 13
A
B
TR50 (h)
2
4
6
scale (kbp)
10^3
10^2
65
70
Position (Mbp)

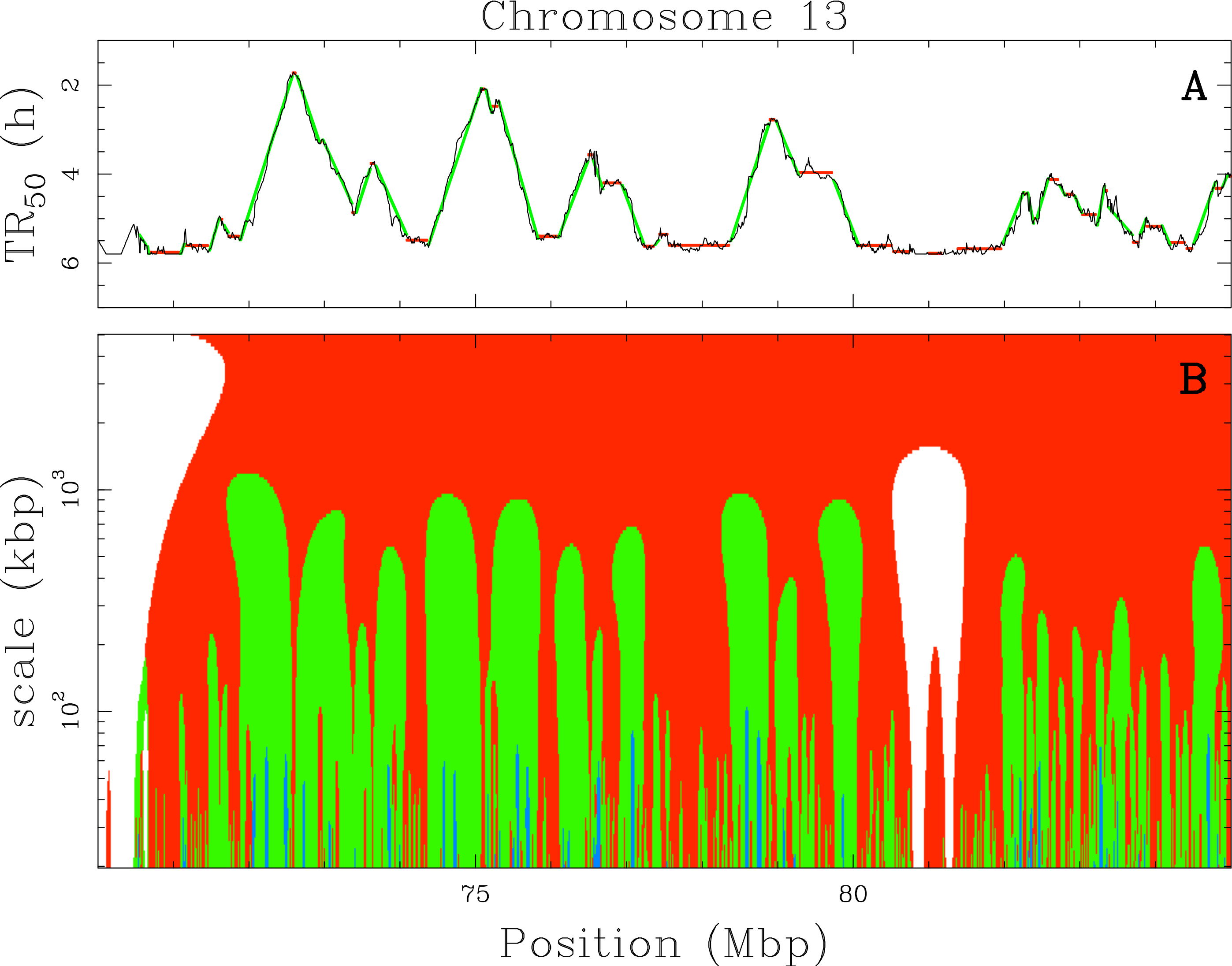

Chromosome 13
A
TR50 (h)
2
4
6
B
scale (kbp)
10^3
10^2
75
80
Position (Mbp)

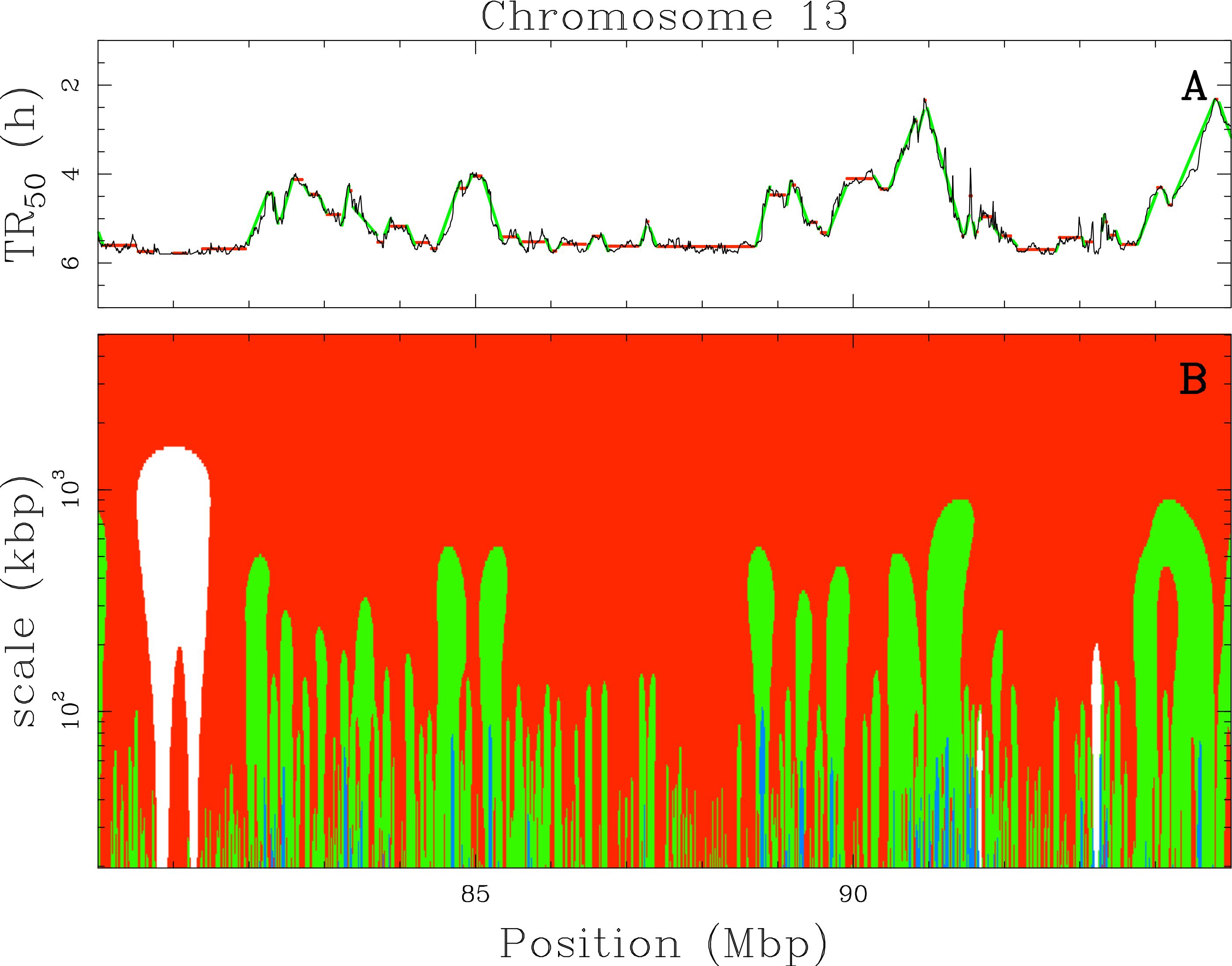

Chromosome 13
A
TR50 (h)
2
4
6
B
scale (kbp)
10^3
10^2
85
90
Position (Mbp)

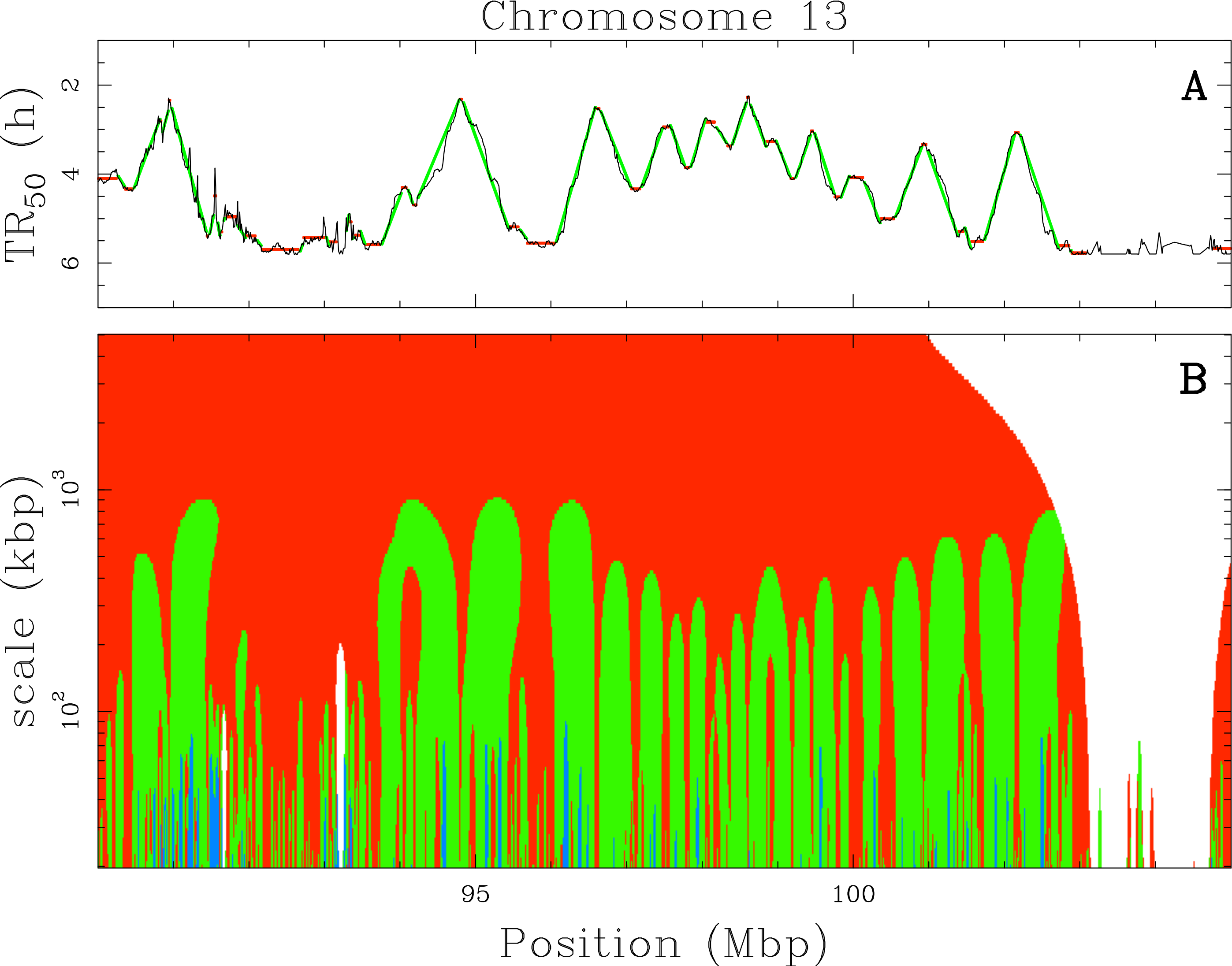

Chromosome 13
TR$_{50}$ (h)
2
4
6
A
B
scale (kbp)
10^3
10^2
95
100
Position (Mbp)

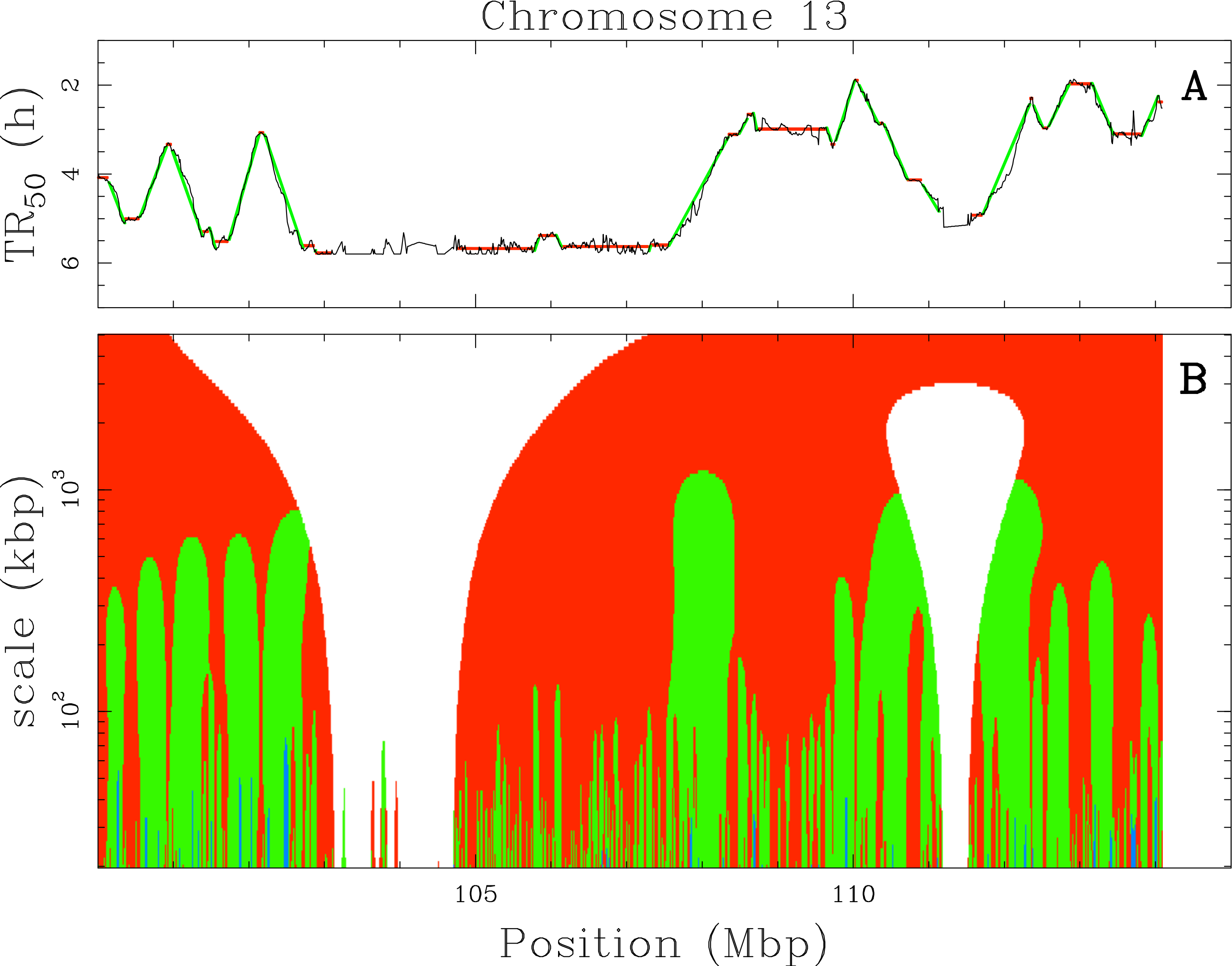

Chromosome 13
A
B
TR50 (h)
2
4
6
scale (kbp)
10^3
10^2
105
110
Position (Mbp)

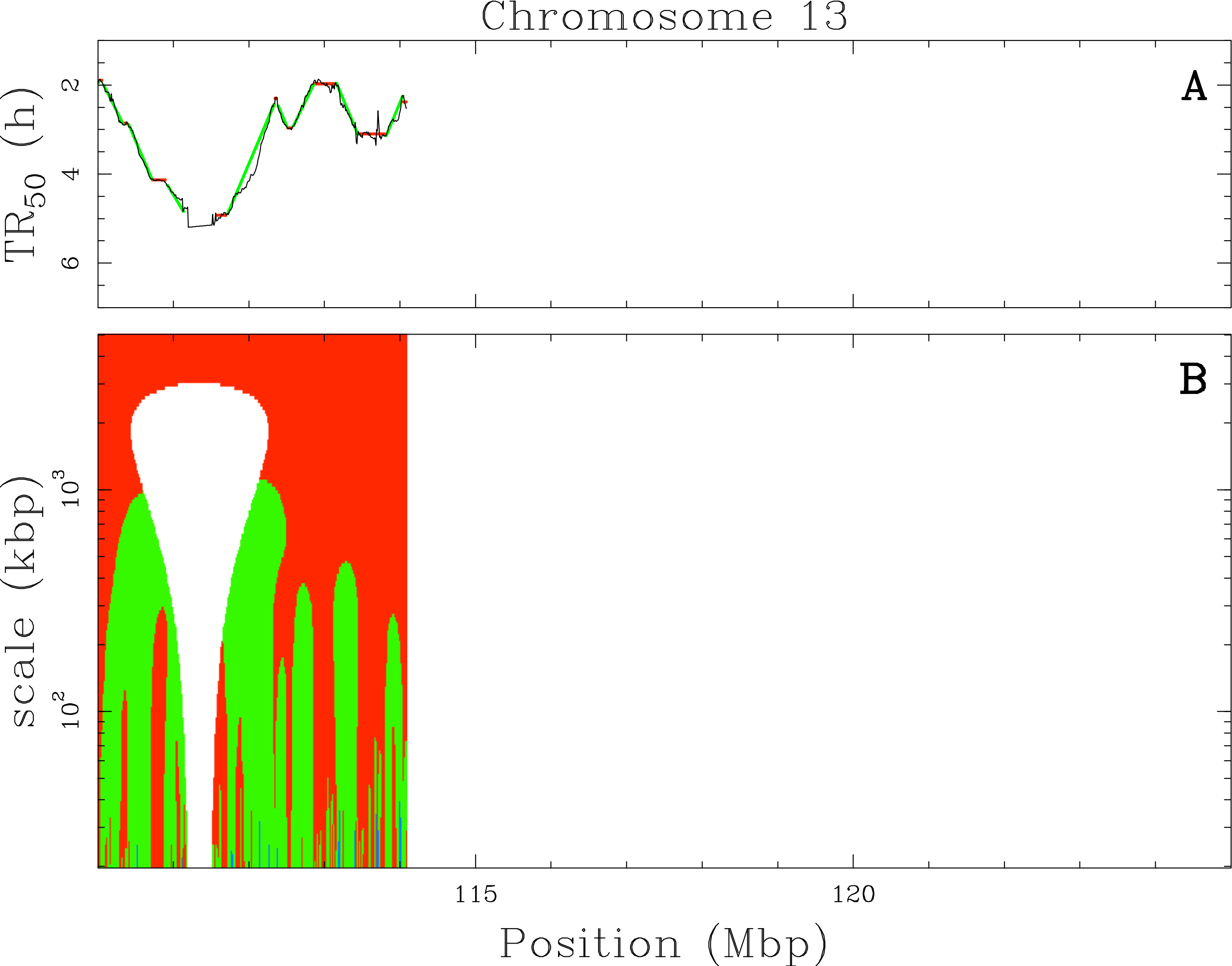
Chromosome 13
A
B
TR50 (h)
2
4
6
scale (kbp)
10^2
10^3
115
120
Position (Mbp)

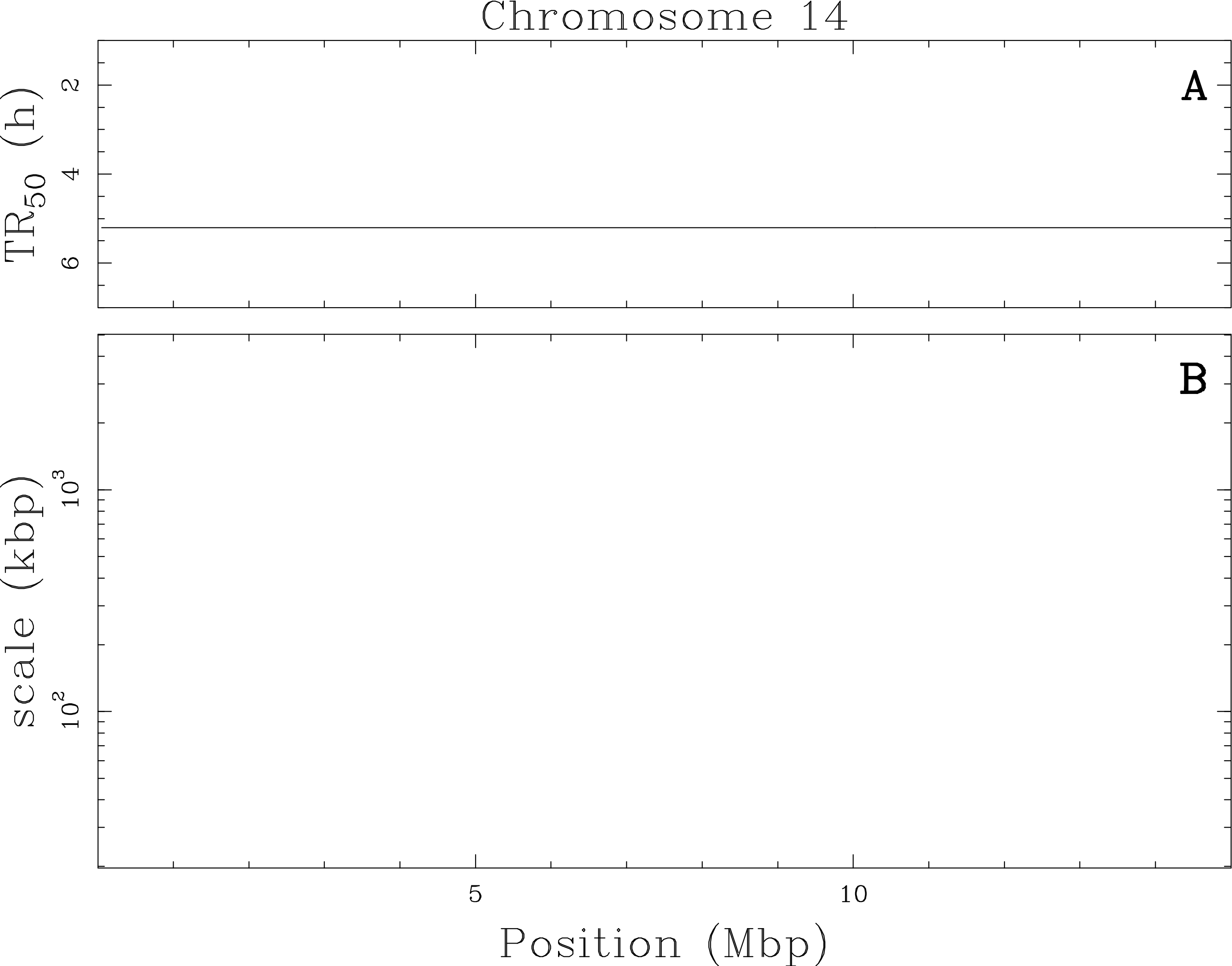
Chromosome 14
A
B
TR50 (h)
2
4
6
scale (kbp)
10^3
10^2
5
10
Position (Mbp)

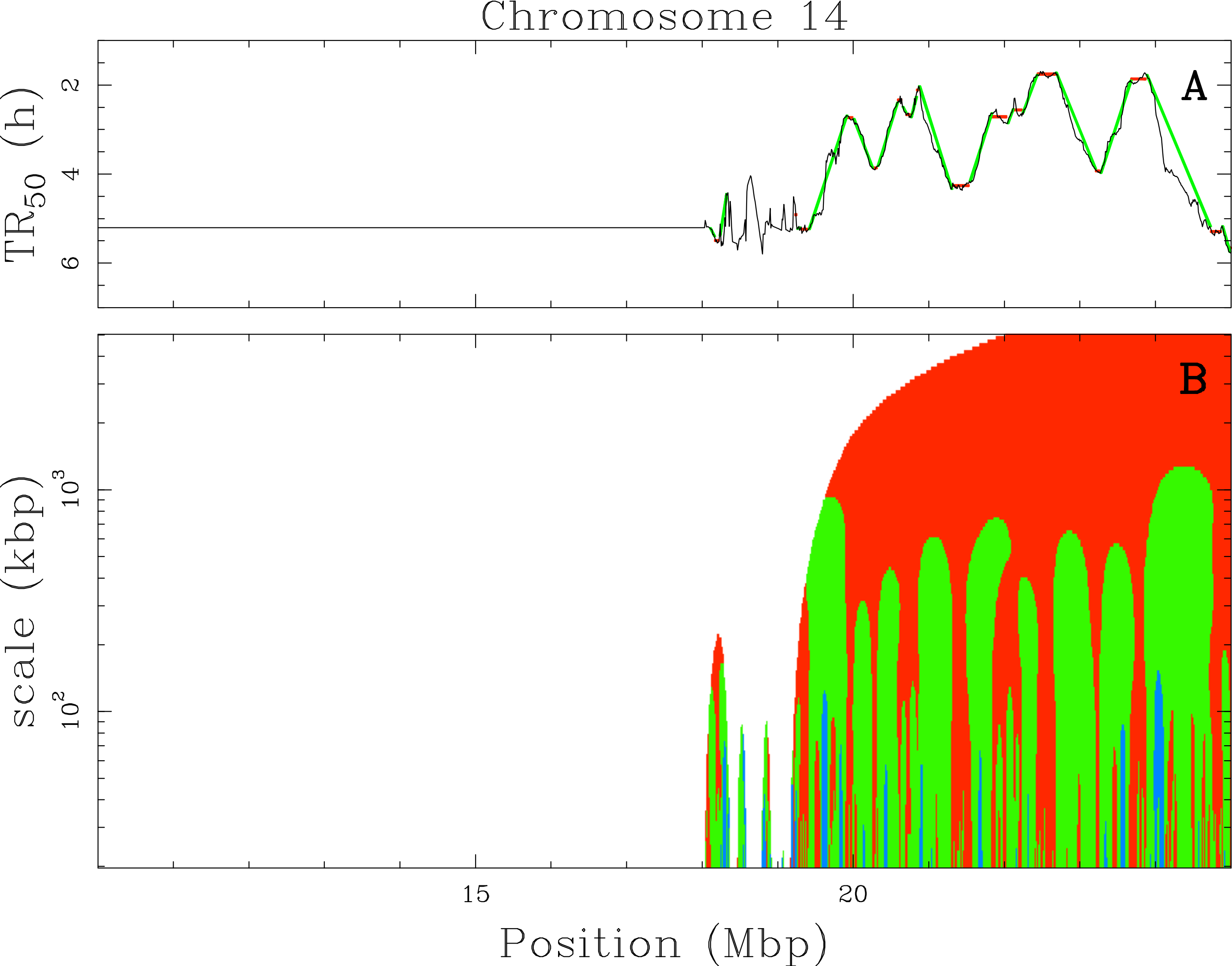

Chromosome 14
A
B
TR50 (h)
2
4
6
scale (kbp)
10^3
10^2
15
20
Position (Mbp)

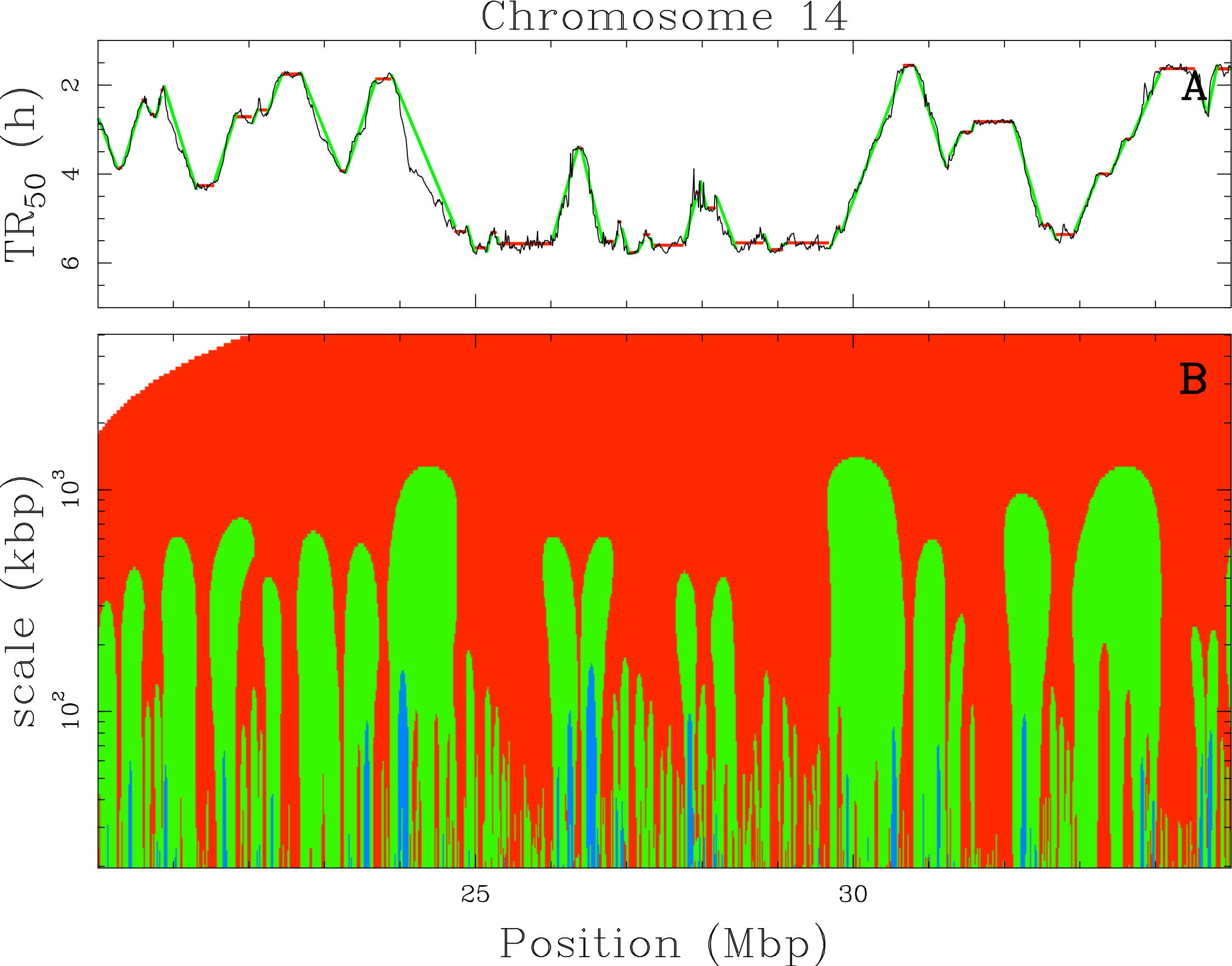

Chromosome 14
TR50 (h)
2
4
6
A
B
scale (kbp)
10^3
10^2
25
30
Position (Mbp)

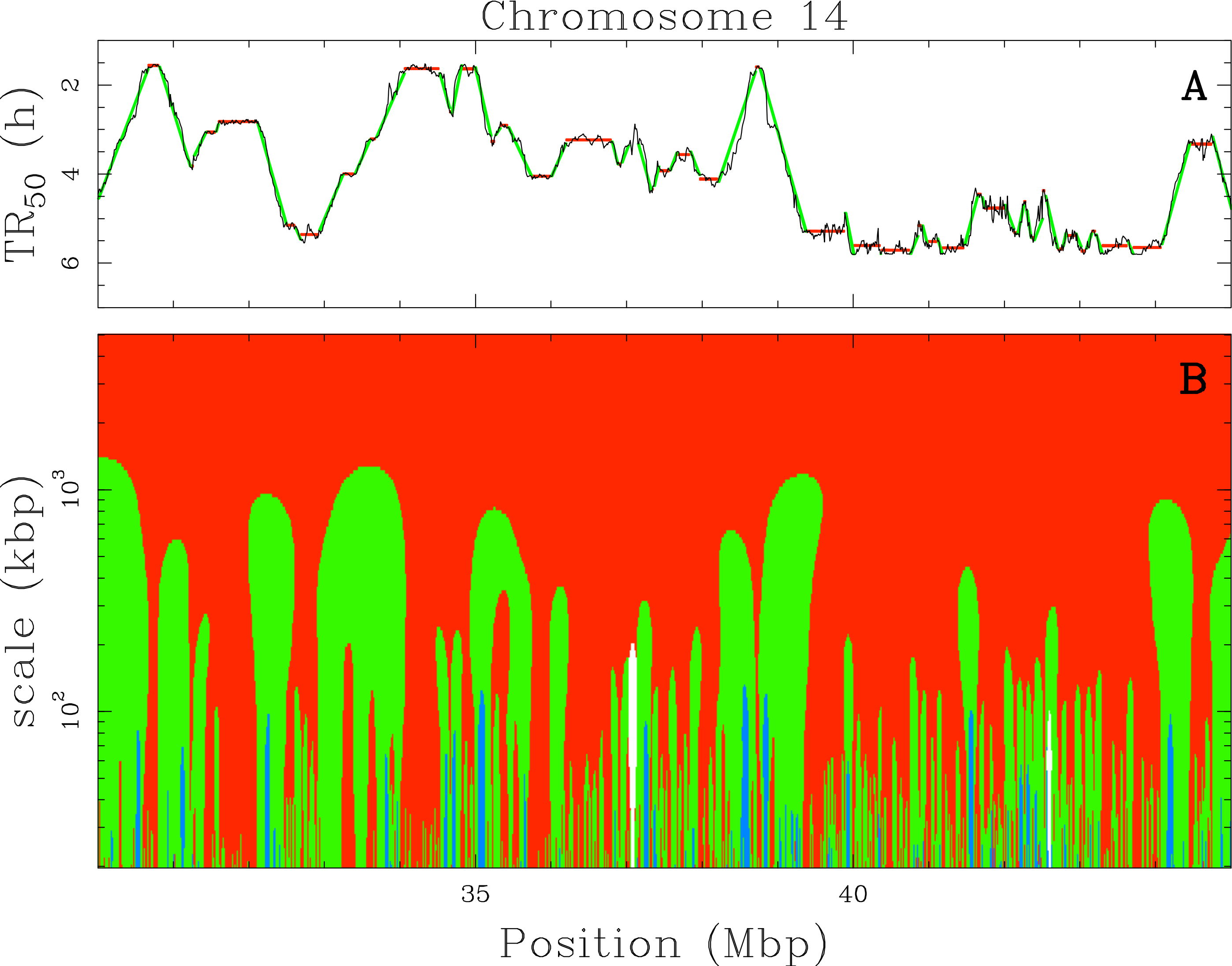

Chromosome 14
A
B
TR50 (h)
2
4
6
scale (kbp)
10^3
10^2
35
40
Position (Mbp)

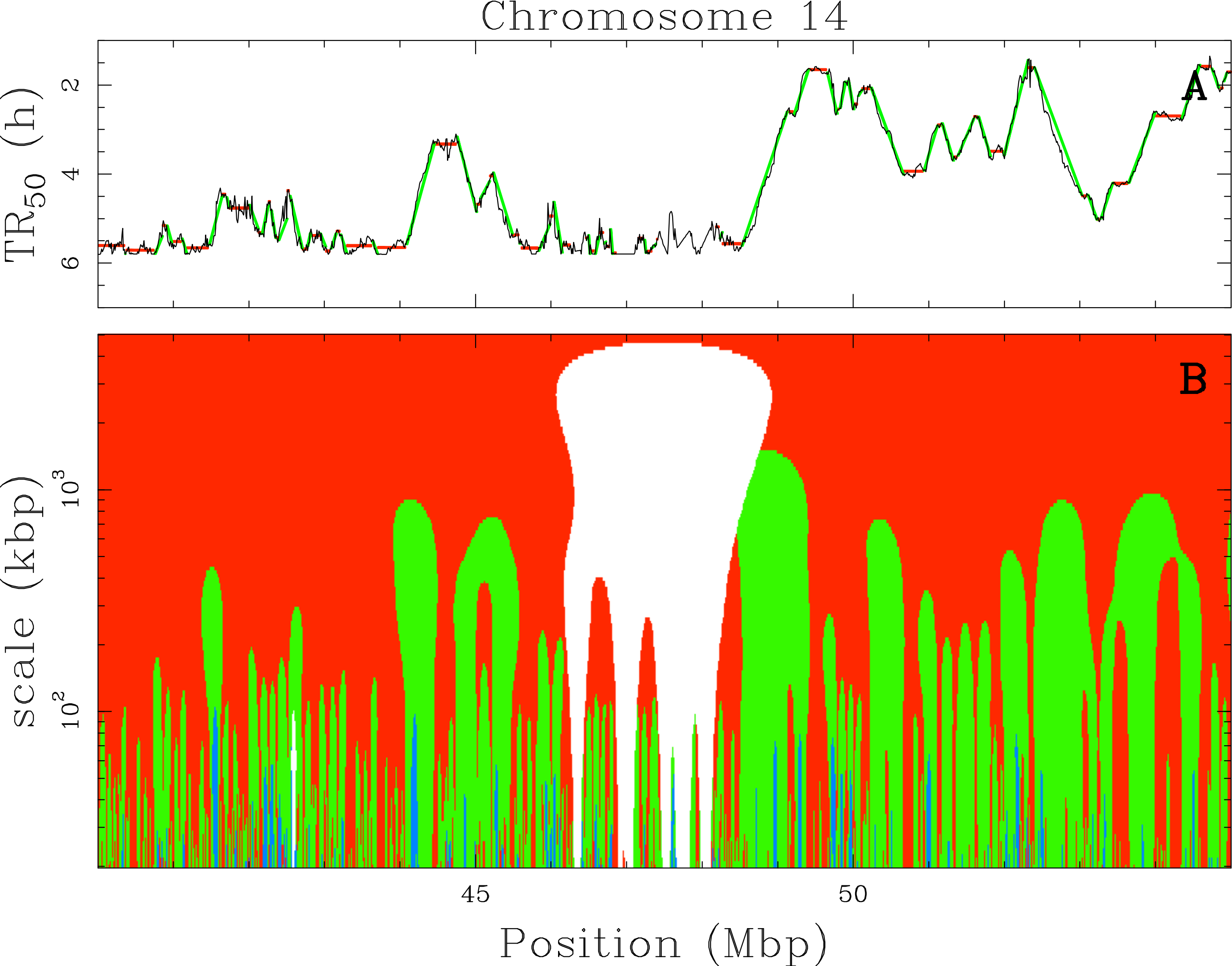

Chromosome 14
TR50 (h)
2
4
6
A
B
scale (kbp)
10^3
10^2
45
50
Position (Mbp)

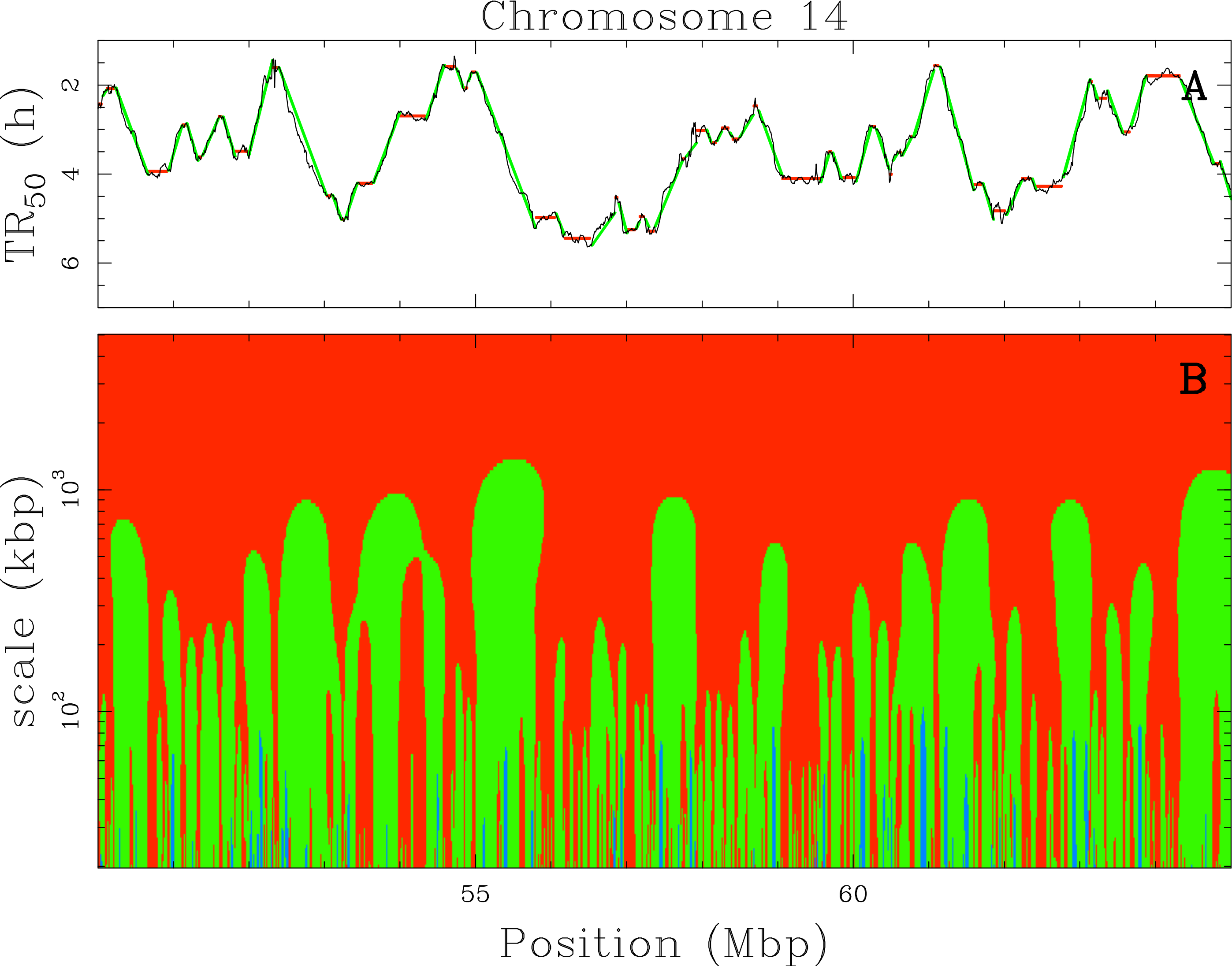

Chromosome 14
A
TR50 (h)
2
4
6
B
scale (kbp)
10^3
10^2
55
60
Position (Mbp)

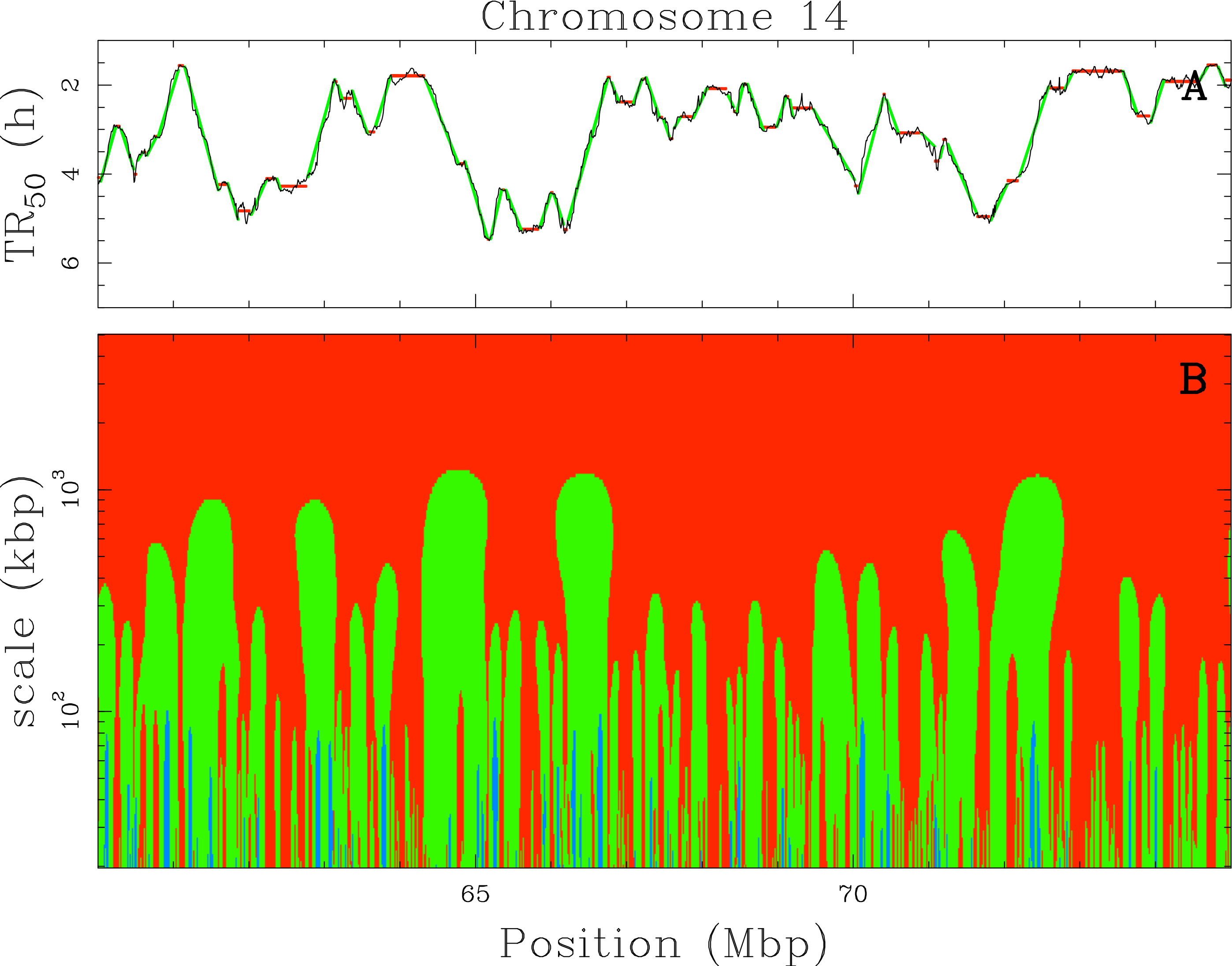

Chromosome 14
TR50 (h)
2
4
6
A
B
scale (kbp)
10^3
10^2
65
70
Position (Mbp)

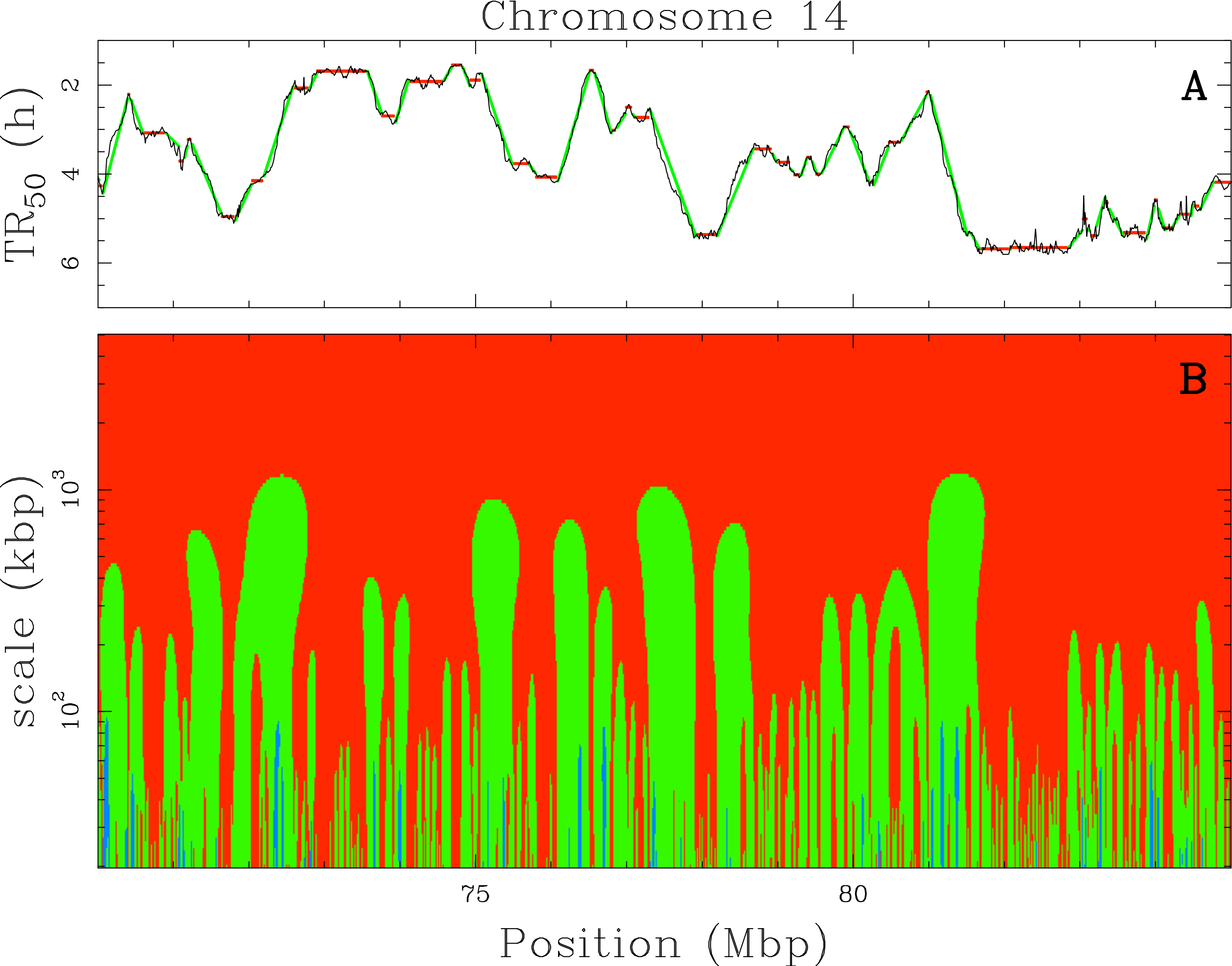

Chromosome 14
A
TR50 (h)
2
4
6
B
scale (kbp)
10^3
10^2
75
80
Position (Mbp)

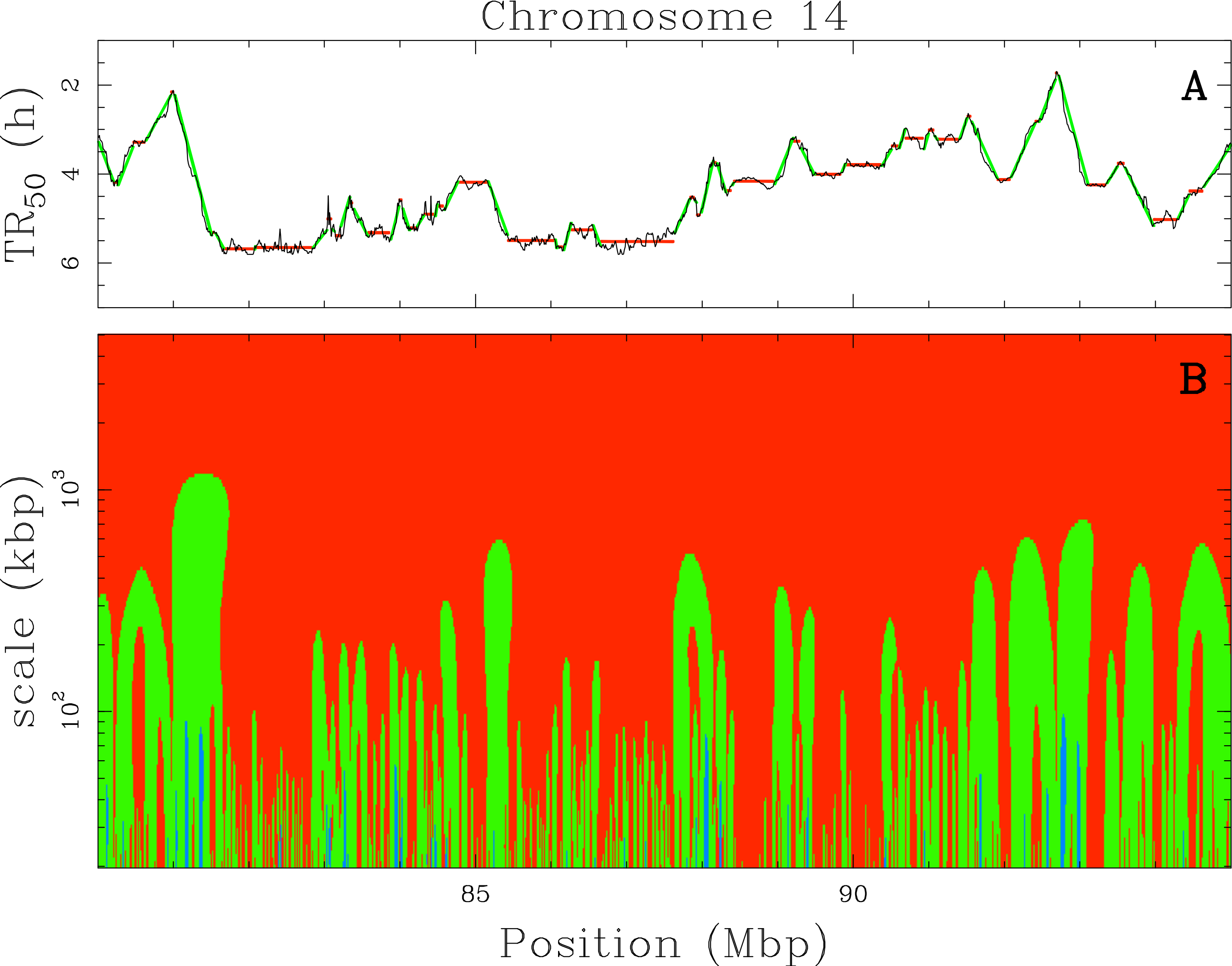
Chromosome 14
A
TR50 (h)
2
4
6
B
scale (kbp)
10^3
10^2
85
90
Position (Mbp)

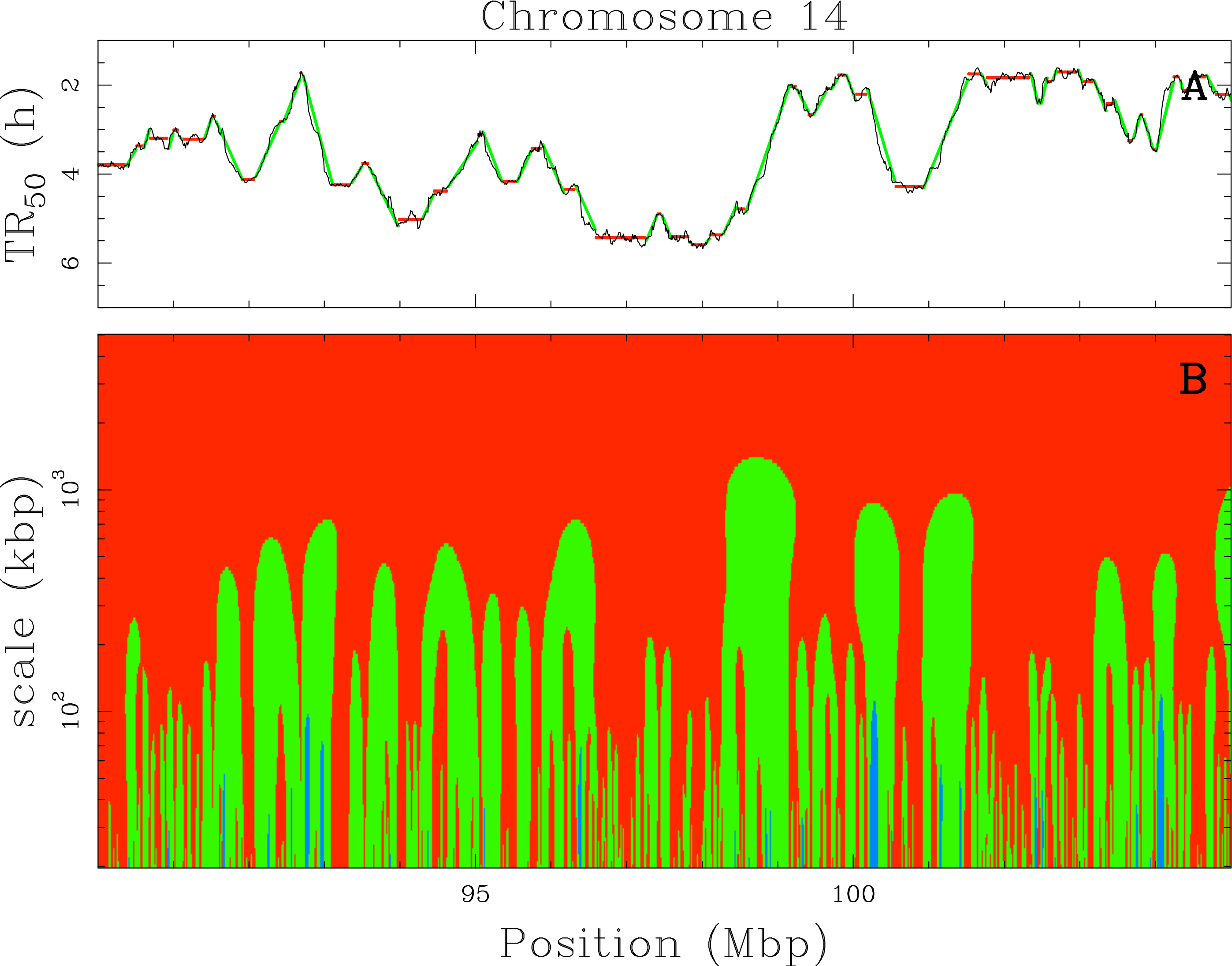
Chromosome 14
TR50 (h)
2
4
6
A
B
scale (kbp)
10^3
10^2
95
100
Position (Mbp)

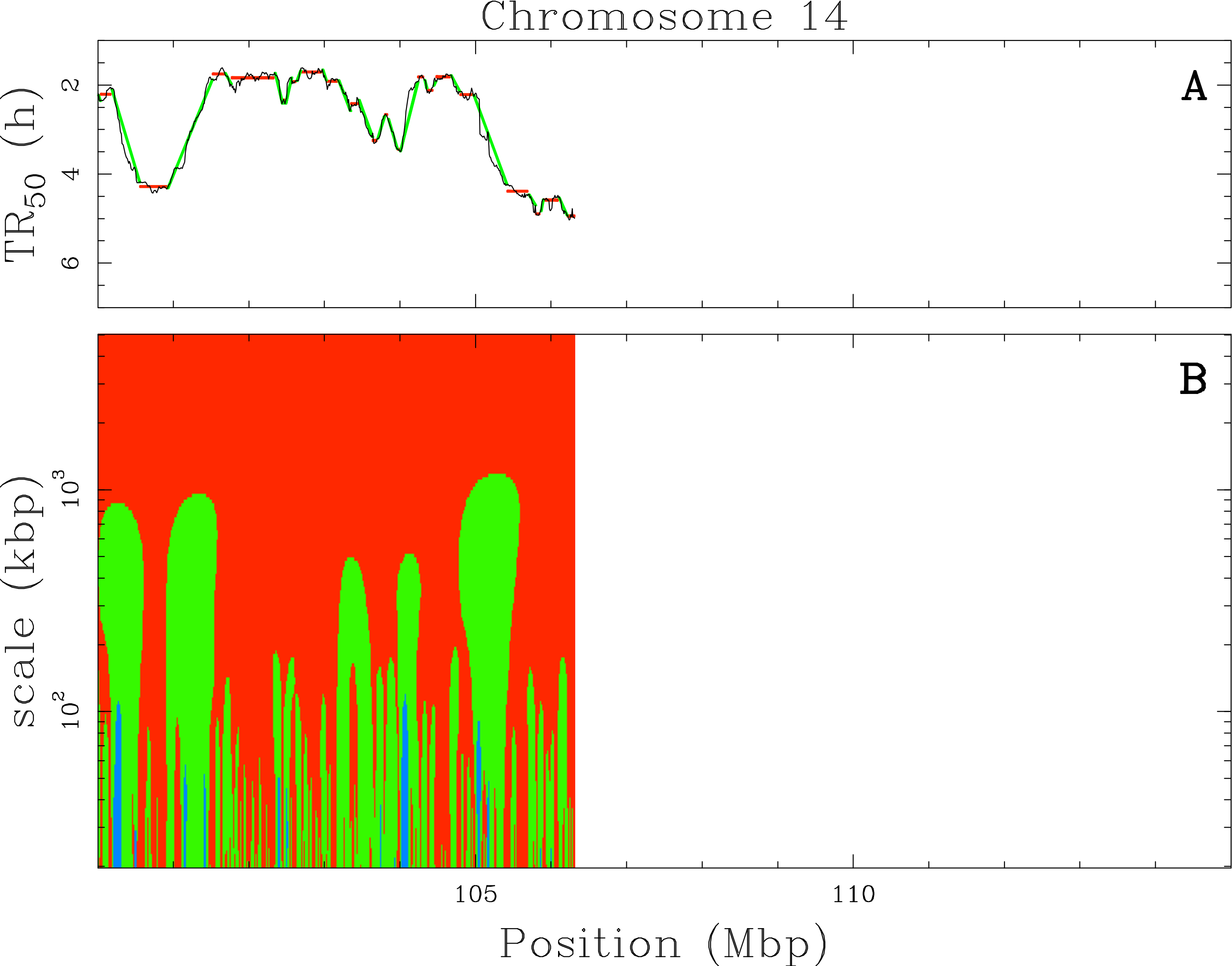

Chromosome 14
TR50 (h)
2
4
6
A
B
scale (kbp)
10^3
10^2
105
110
Position (Mbp)

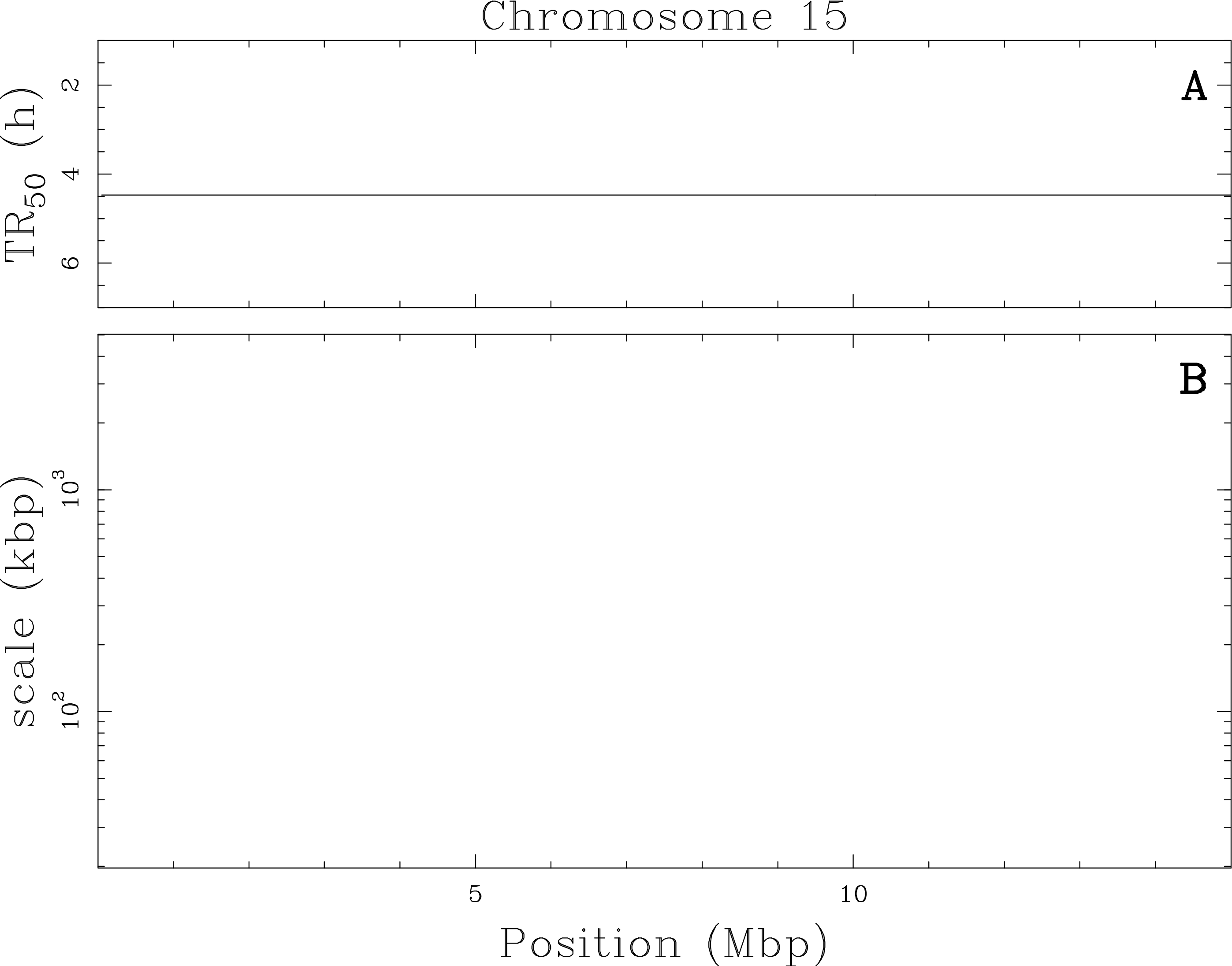
Chromosome 15
A
B
TR50 (h)
2
4
6
scale (kbp)
10^3
10^2
5
10
Position (Mbp)

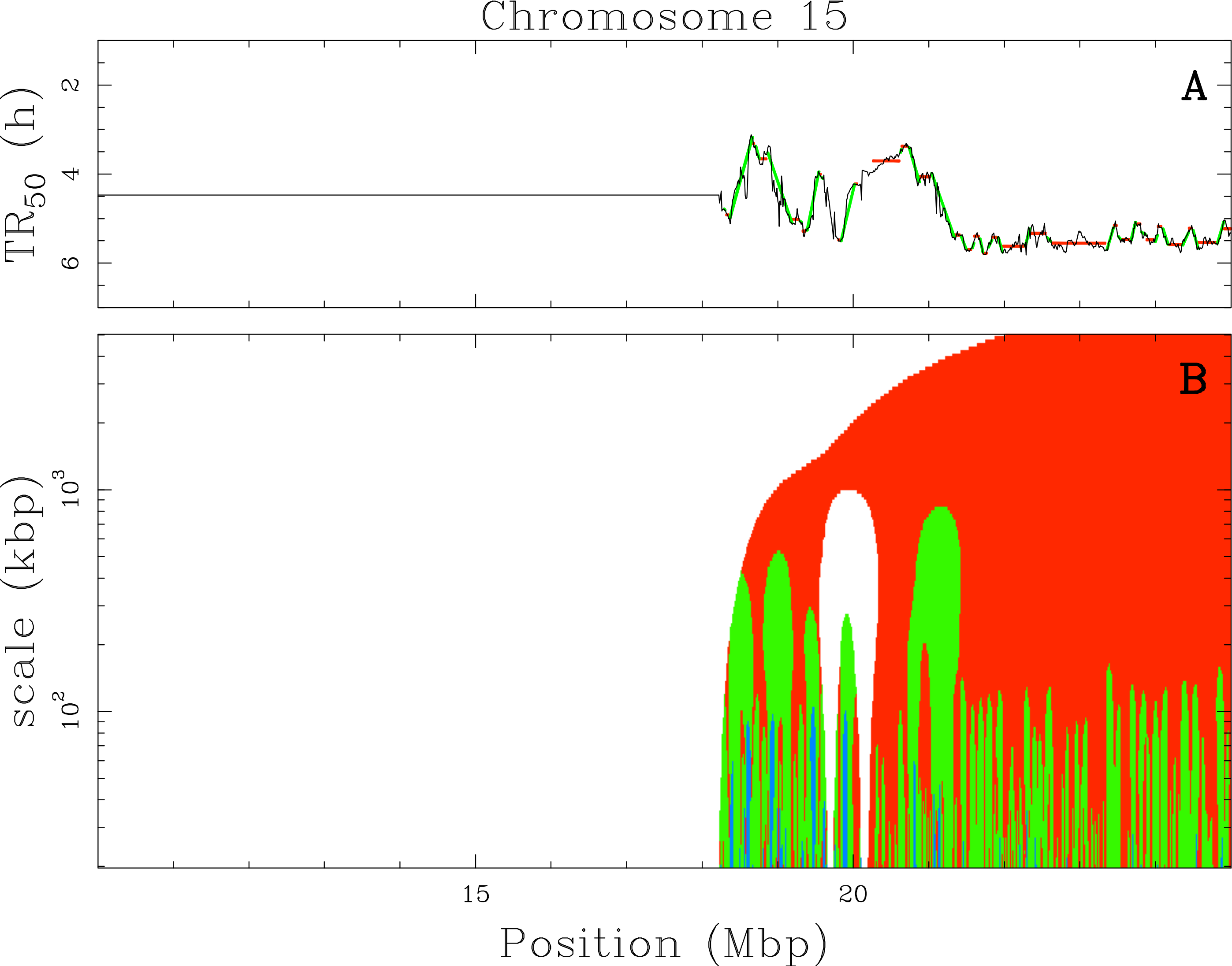
Chromosome 15
A
B
TR50 (h)
2
4
6
scale (kbp)
10^3
10^2
15
20
Position (Mbp)

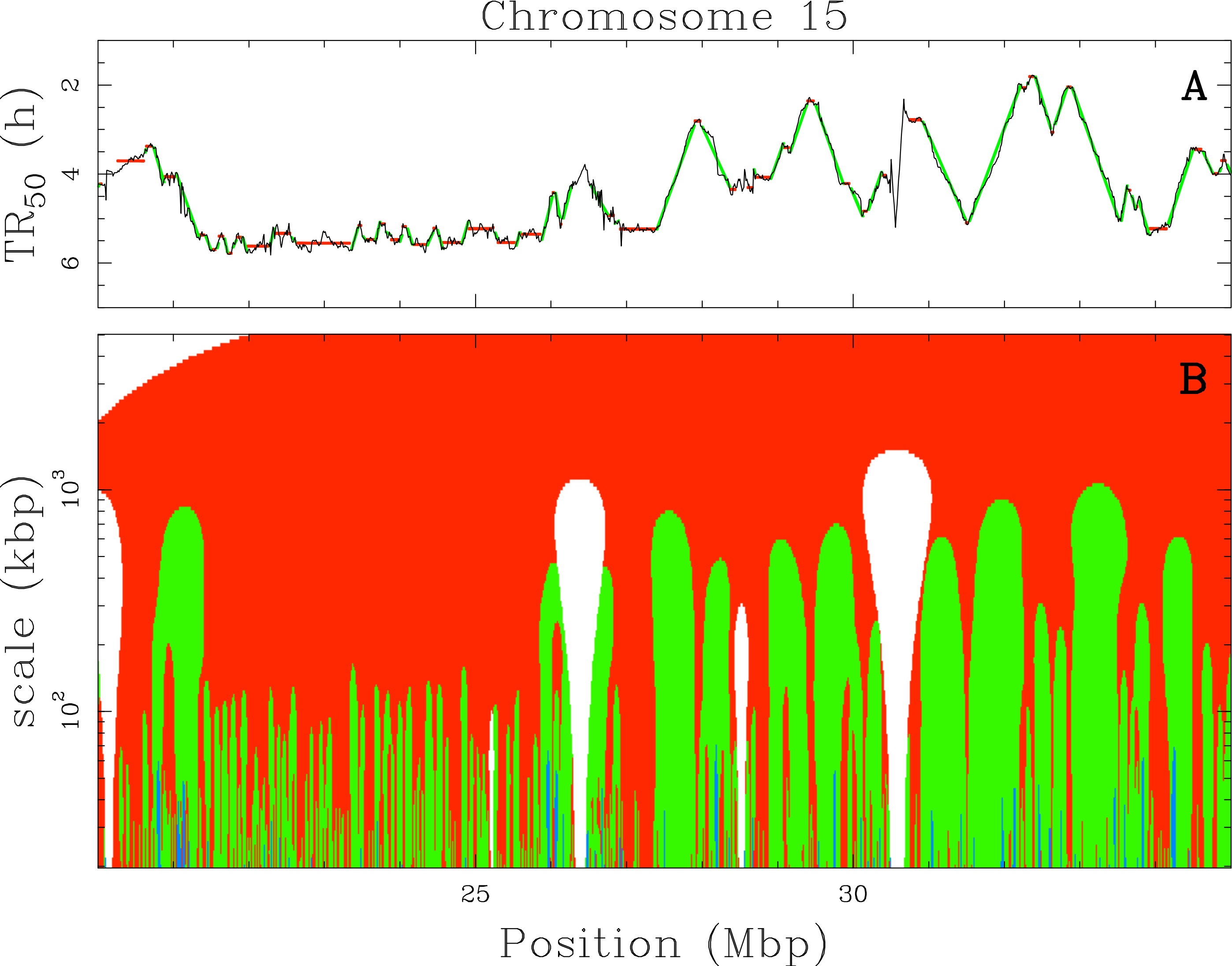
Chromosome 15
A
B
TR50 (h)
2
4
6
scale (kbp)
10^3
10^2
25
30
Position (Mbp)

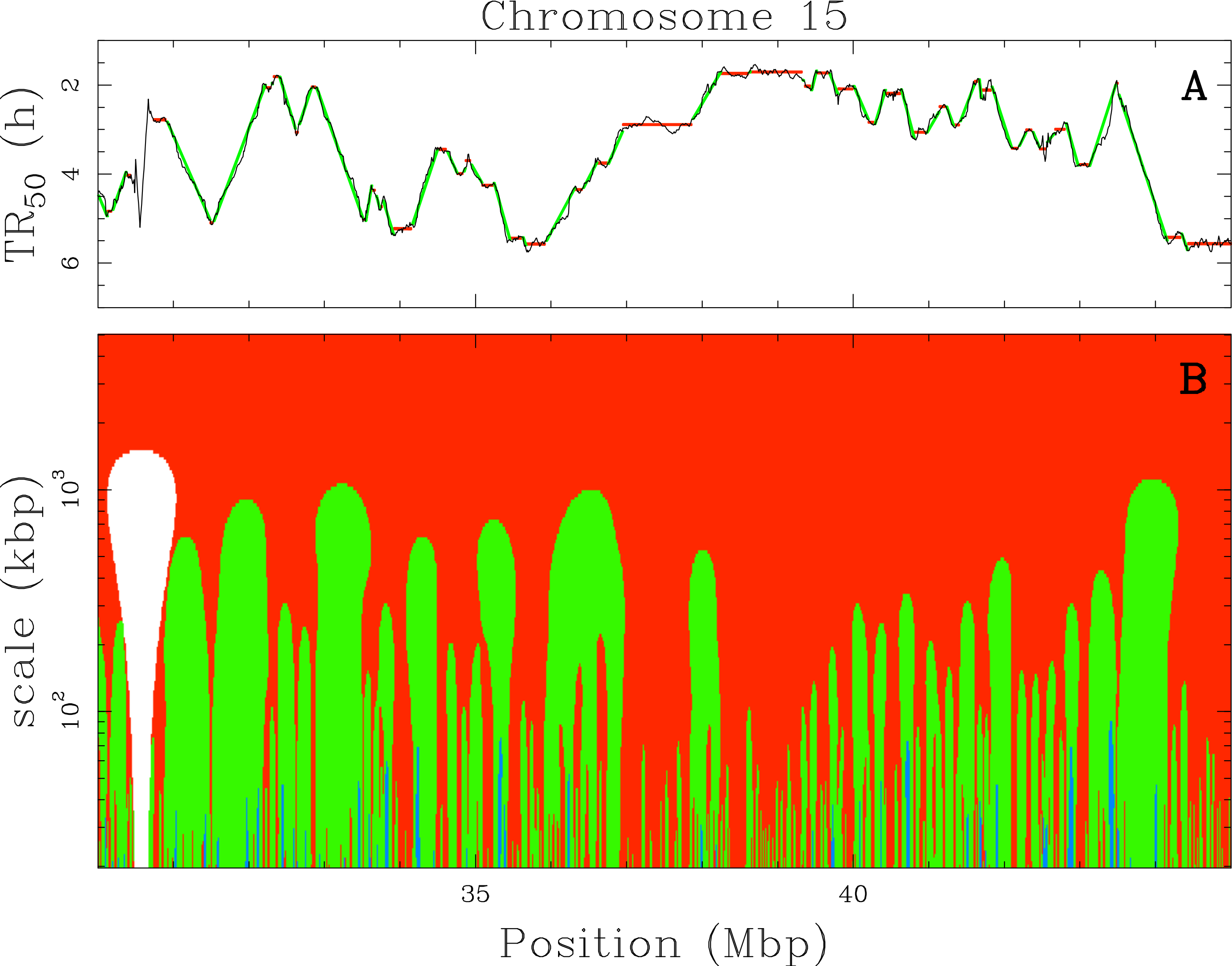

Chromosome 15
TR50 (h)
2
4
6
A
B
scale (kbp)
10^3
10^2
35
40
Position (Mbp)

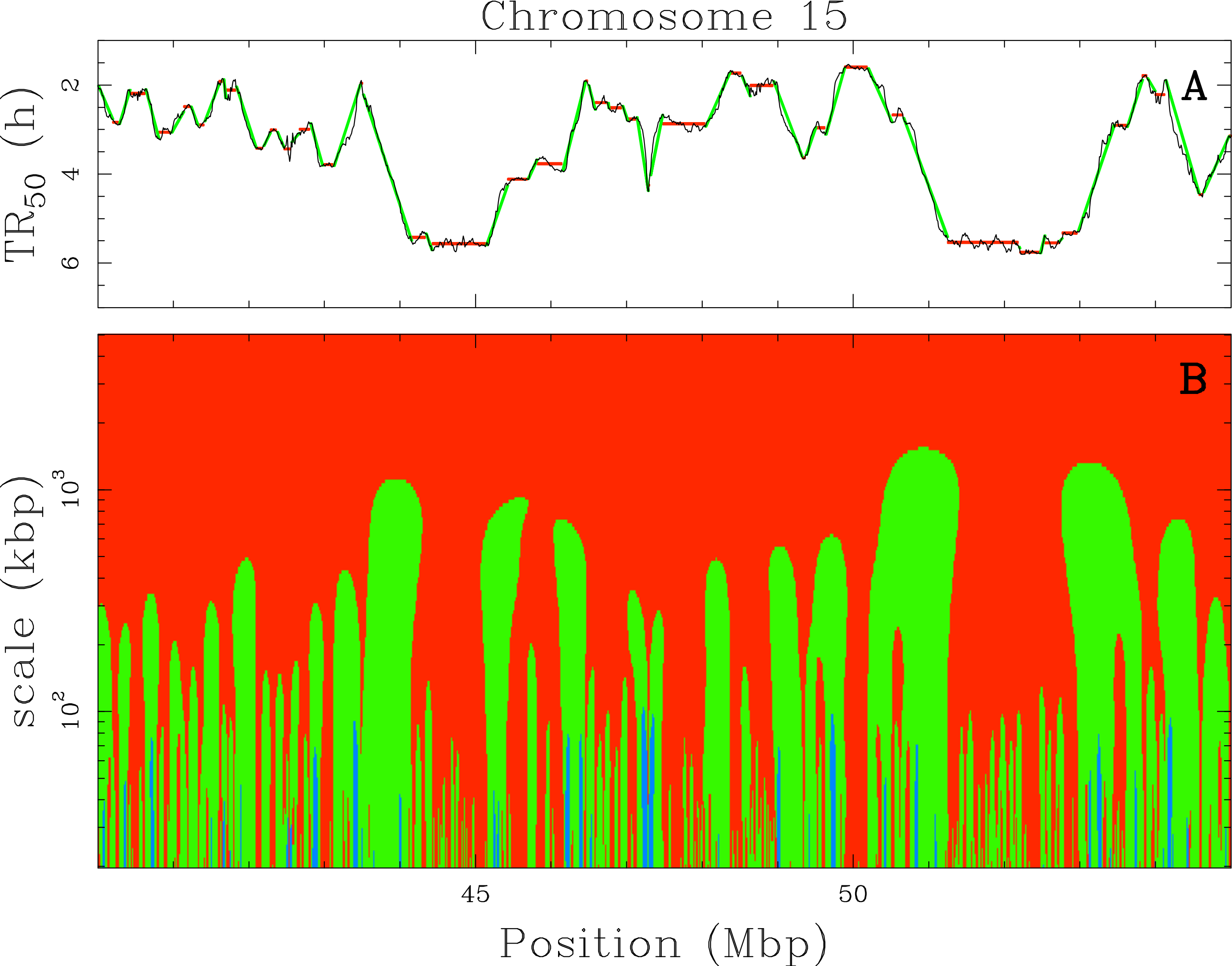

Chromosome 15
A
B
TR50 (h)
2
4
6
scale (kbp)
10^3
10^2
45
50
Position (Mbp)

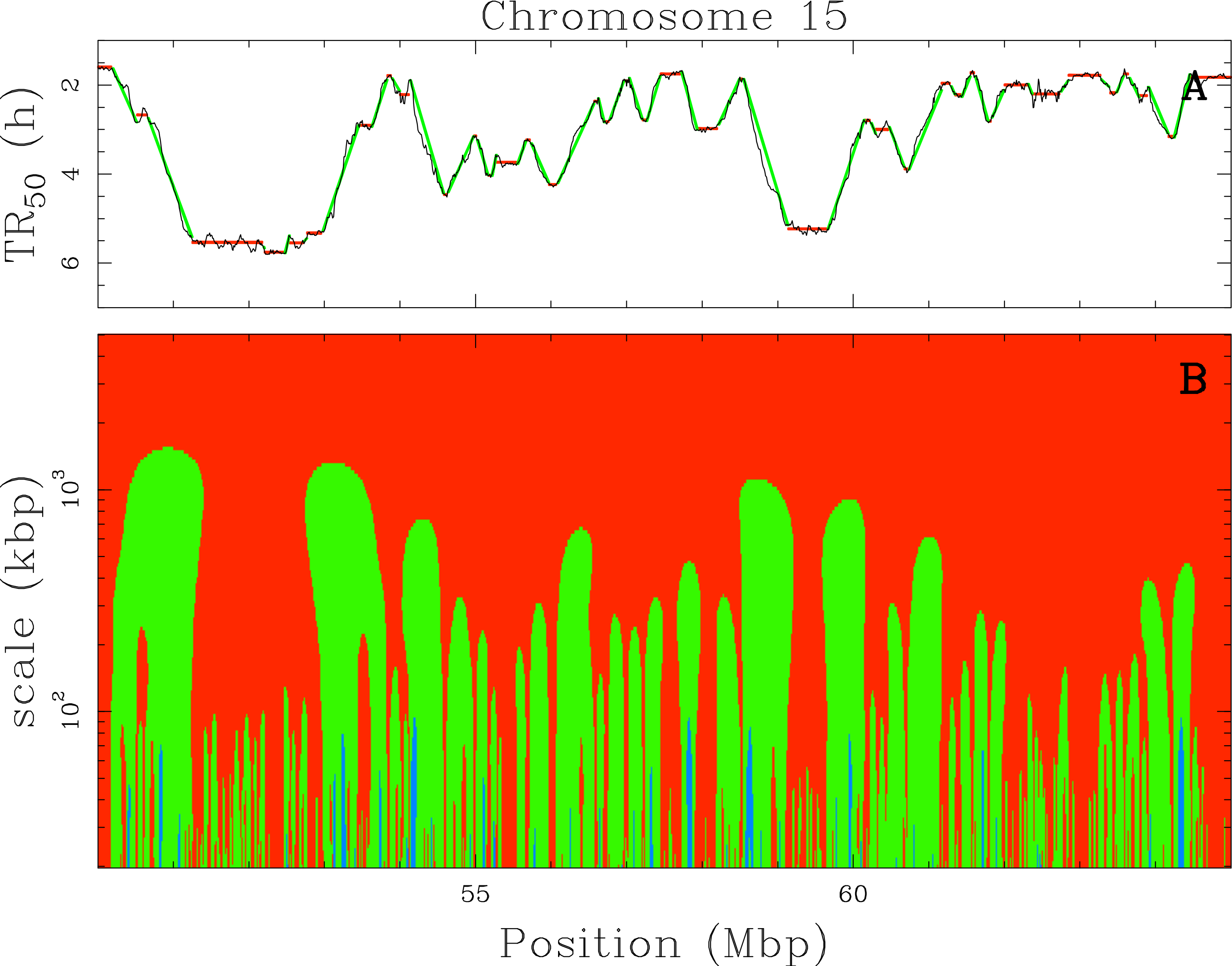

Chromosome 15
A
TR_{50} (h)
2
4
6
B
scale (kbp)
10^3
10^2
55
60
Position (Mbp)

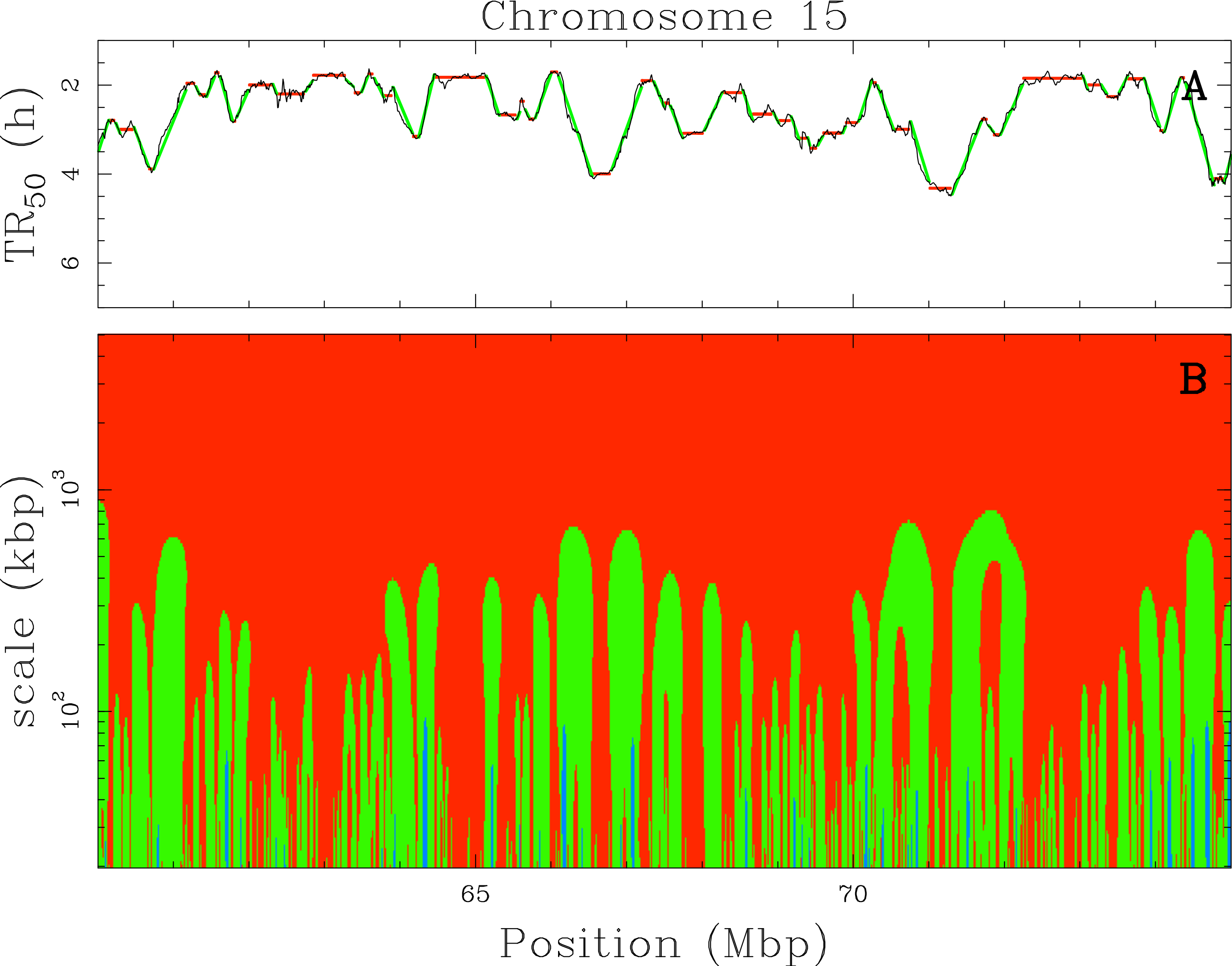

Chromosome 15
A
TR_{50} (h)
2
4
6
B
scale (kbp)
10^3
10^2
65
70
Position (Mbp)

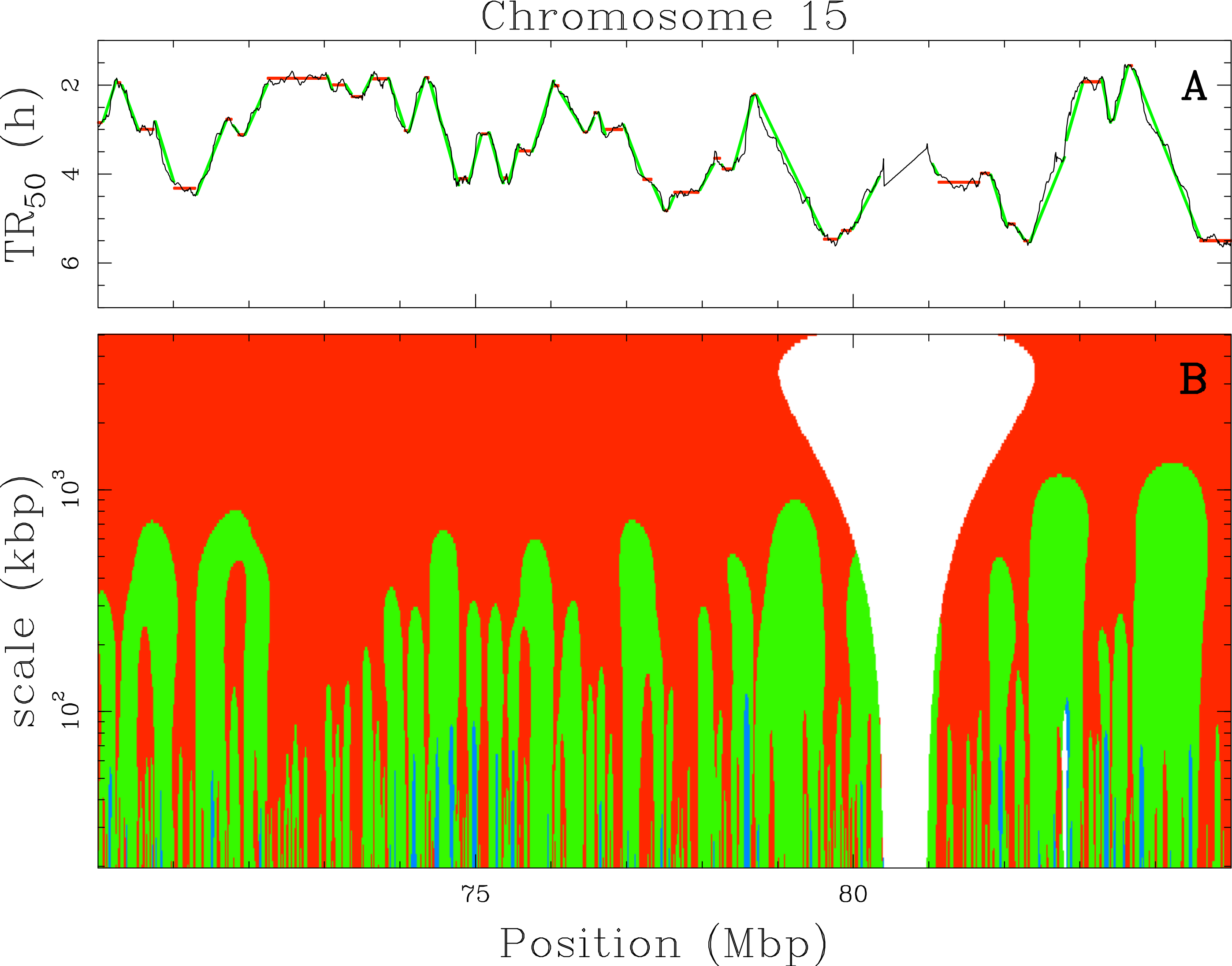
Chromosome 15
A
TR50 (h)
2
4
6
B
scale (kbp)
10^3
10^2
75
80
Position (Mbp)

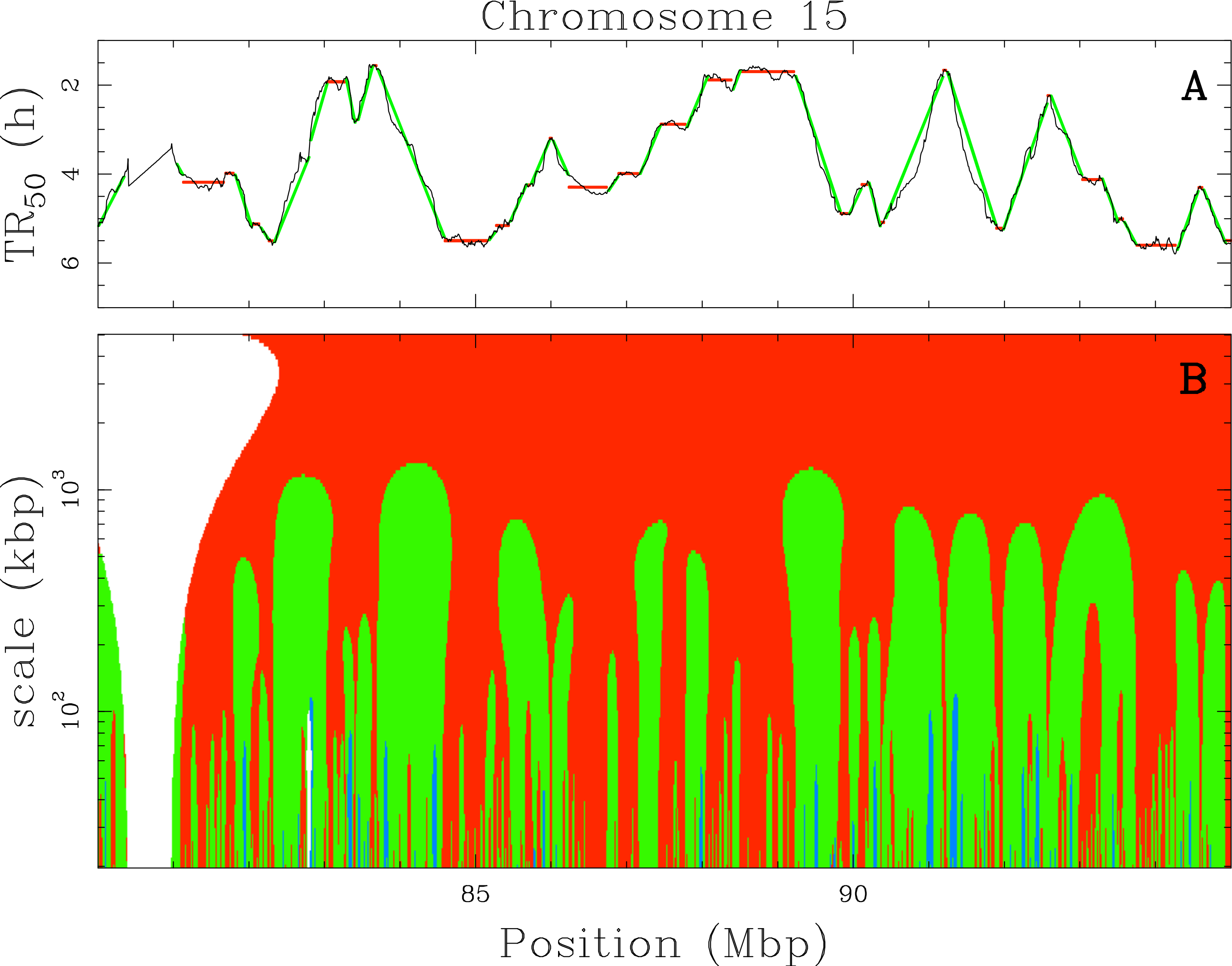

Chromosome 15
TR50 (h)
2
4
6
A
B
scale (kbp)
10^3
10^2
85
90
Position (Mbp)

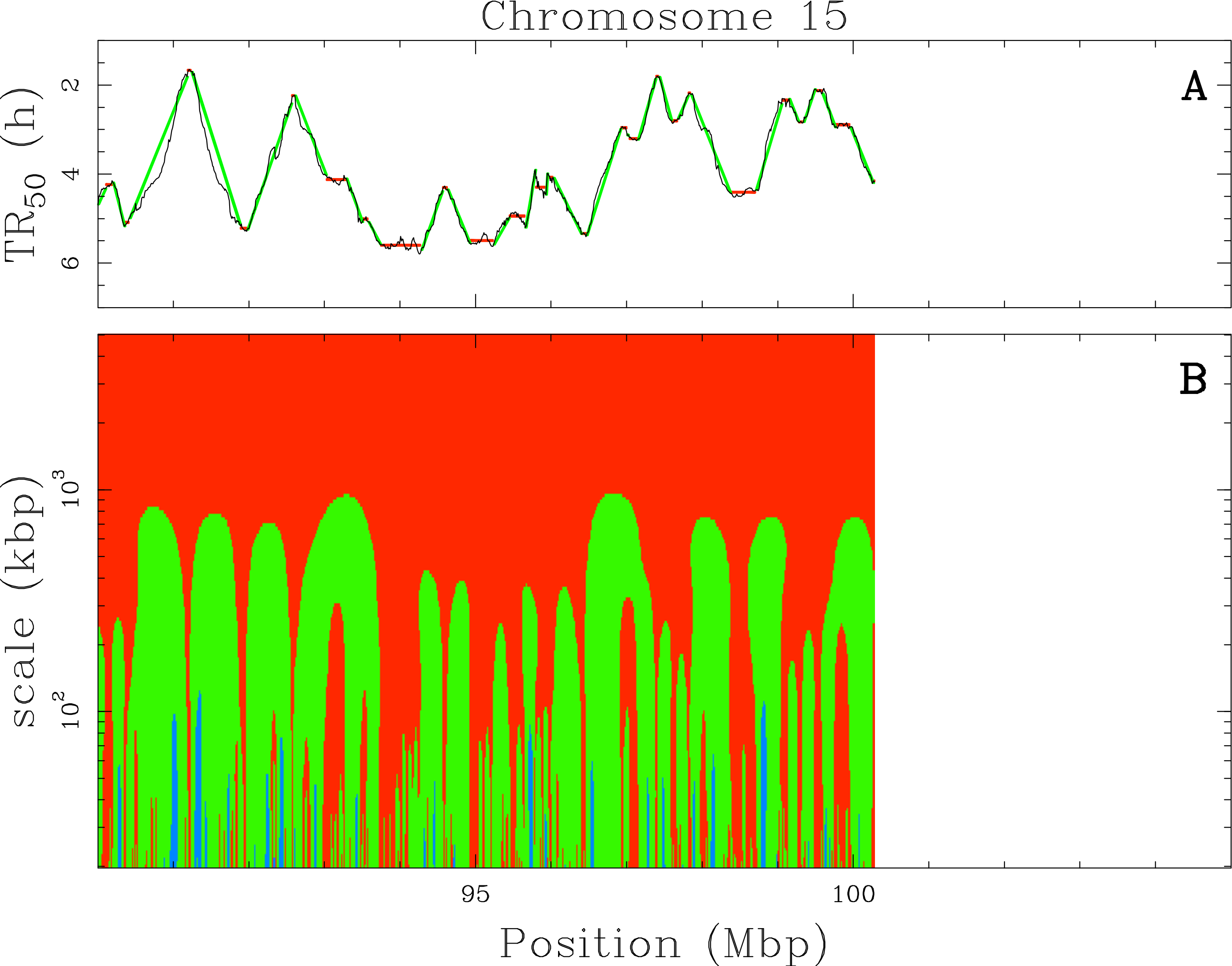

Chromosome 15
TR50 (h)
2
4
6
A
B
scale (kbp)
10^3
10^2
95
100
Position (Mbp)

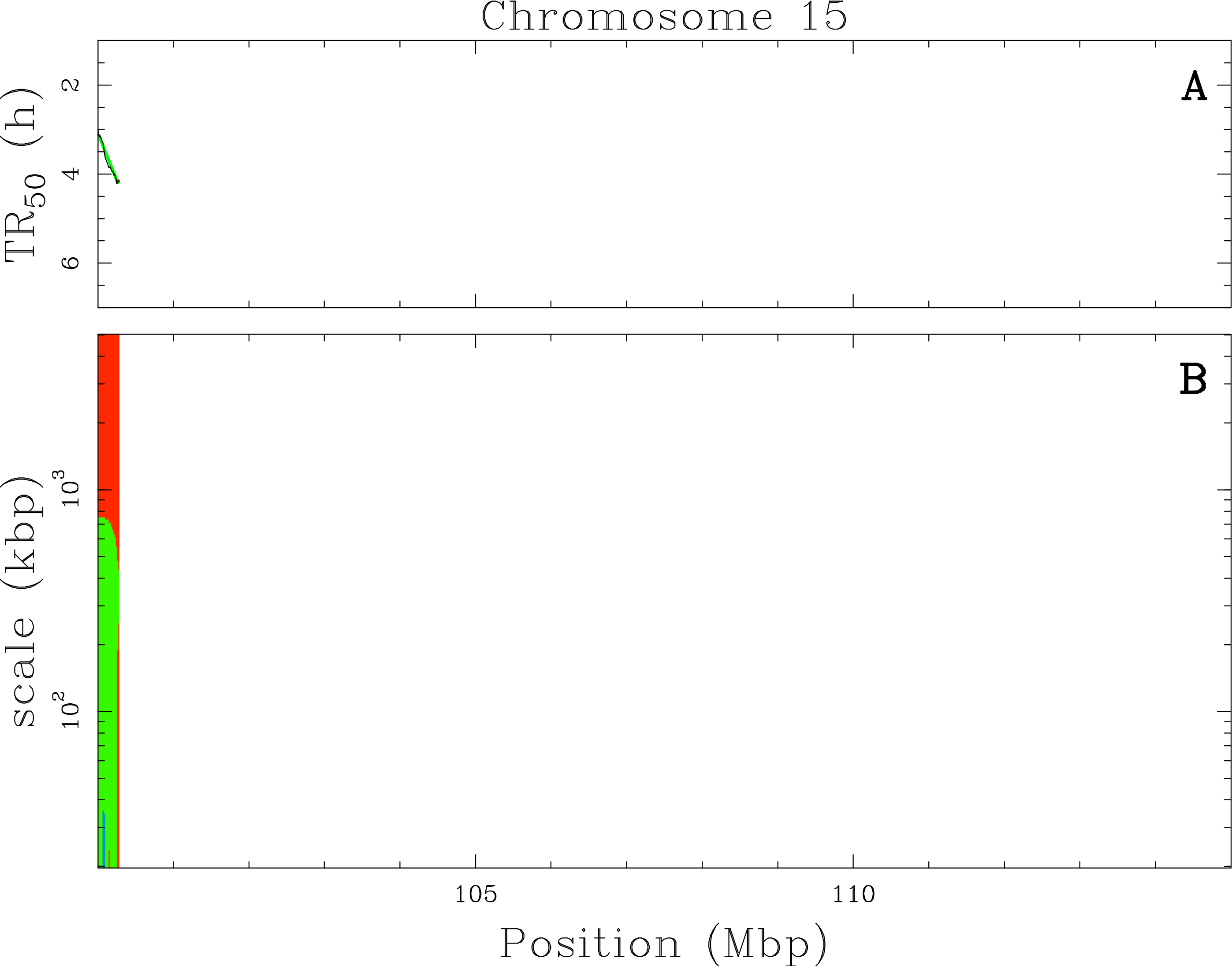
Chromosome 15
A
B
TR50 (h)
2
4
6
scale (kbp)
10^3
10^2
105
110
Position (Mbp)

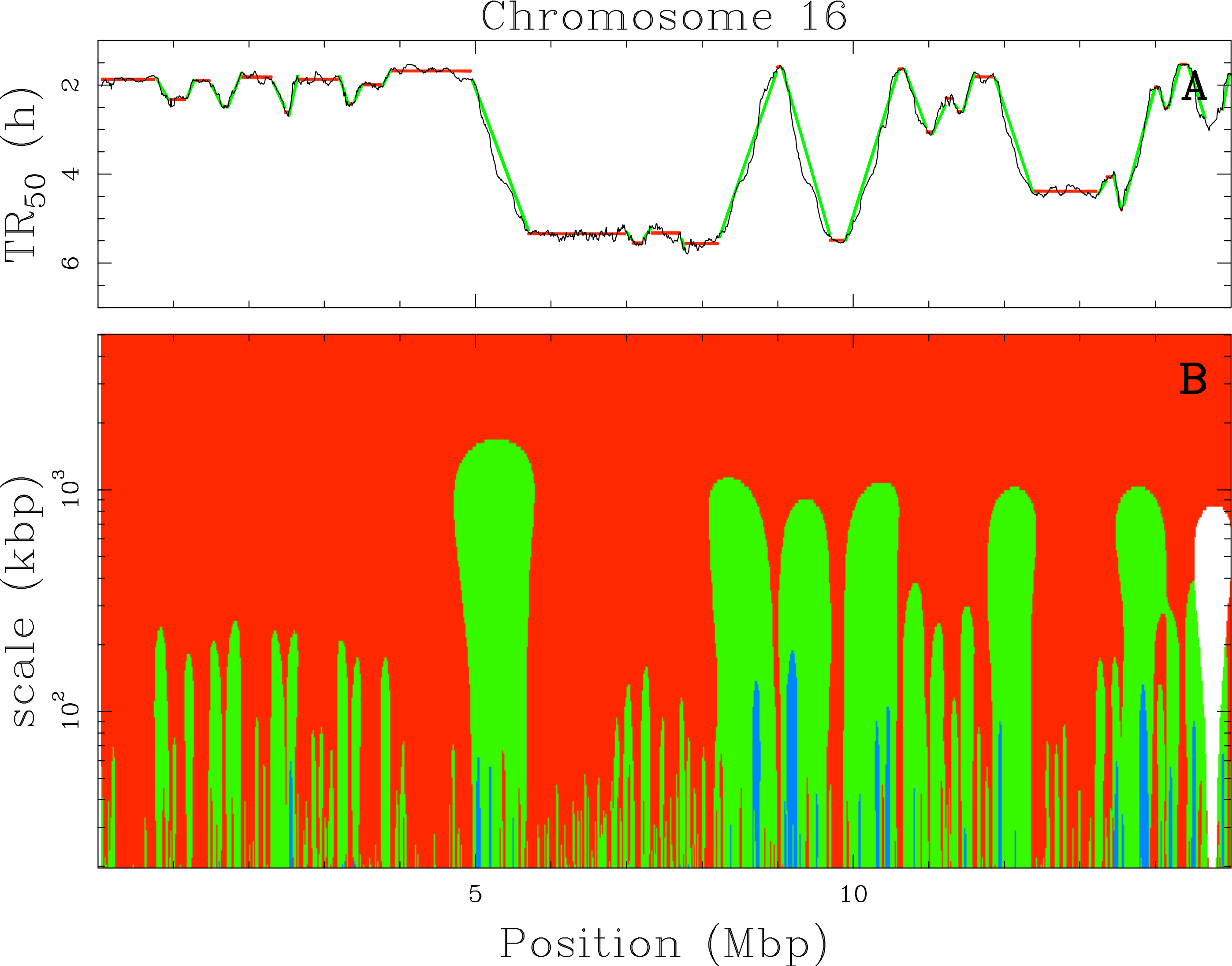

Chromosome 16
TR$_{50}$ (h)
2
4
6
A
B
scale (kbp)
10^3
10^2
5
10
Position (Mbp)

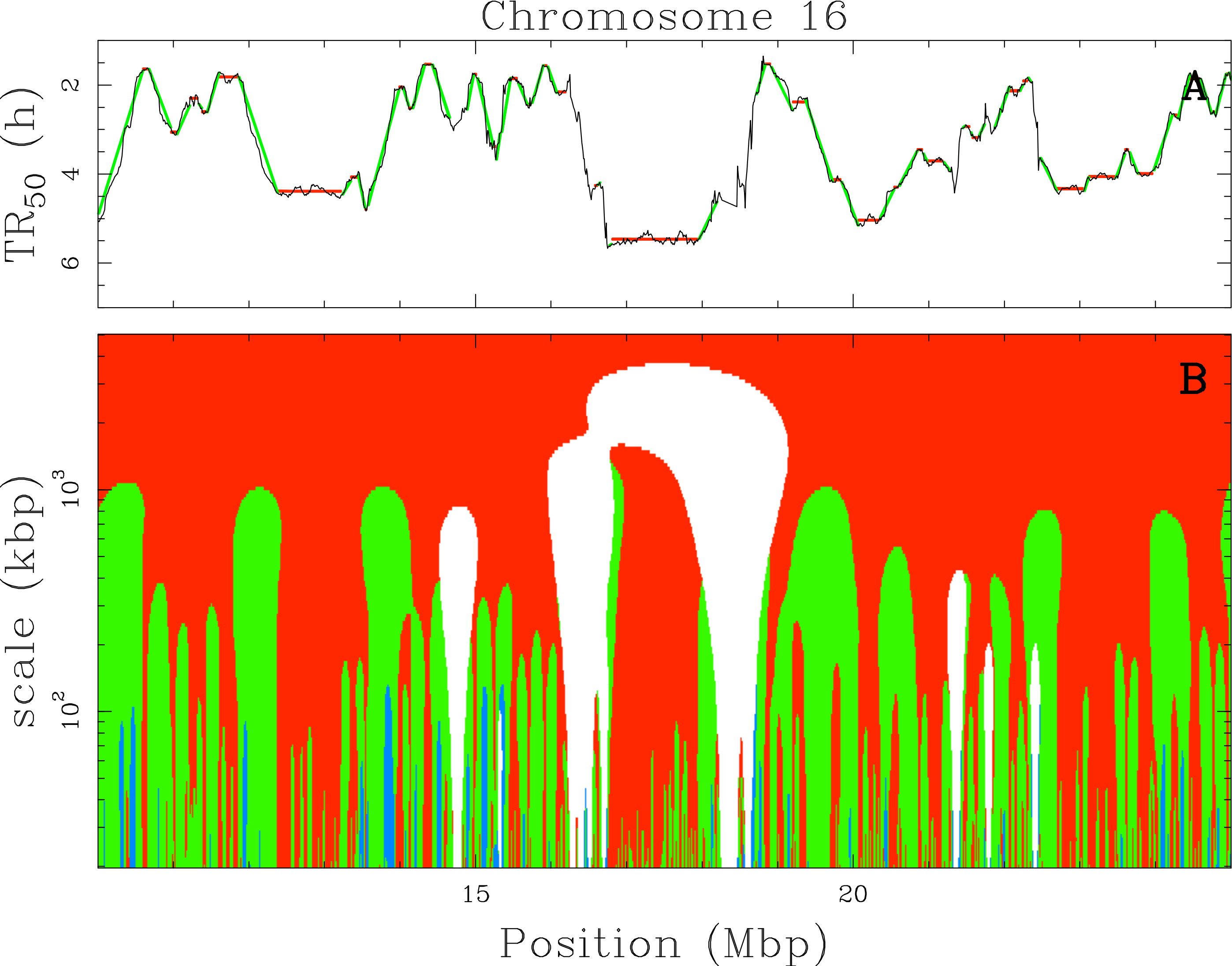

Chromosome 16
A
B
TR50 (h)
2
4
6
scale (kbp)
10^3
10^2
15
20
Position (Mbp)

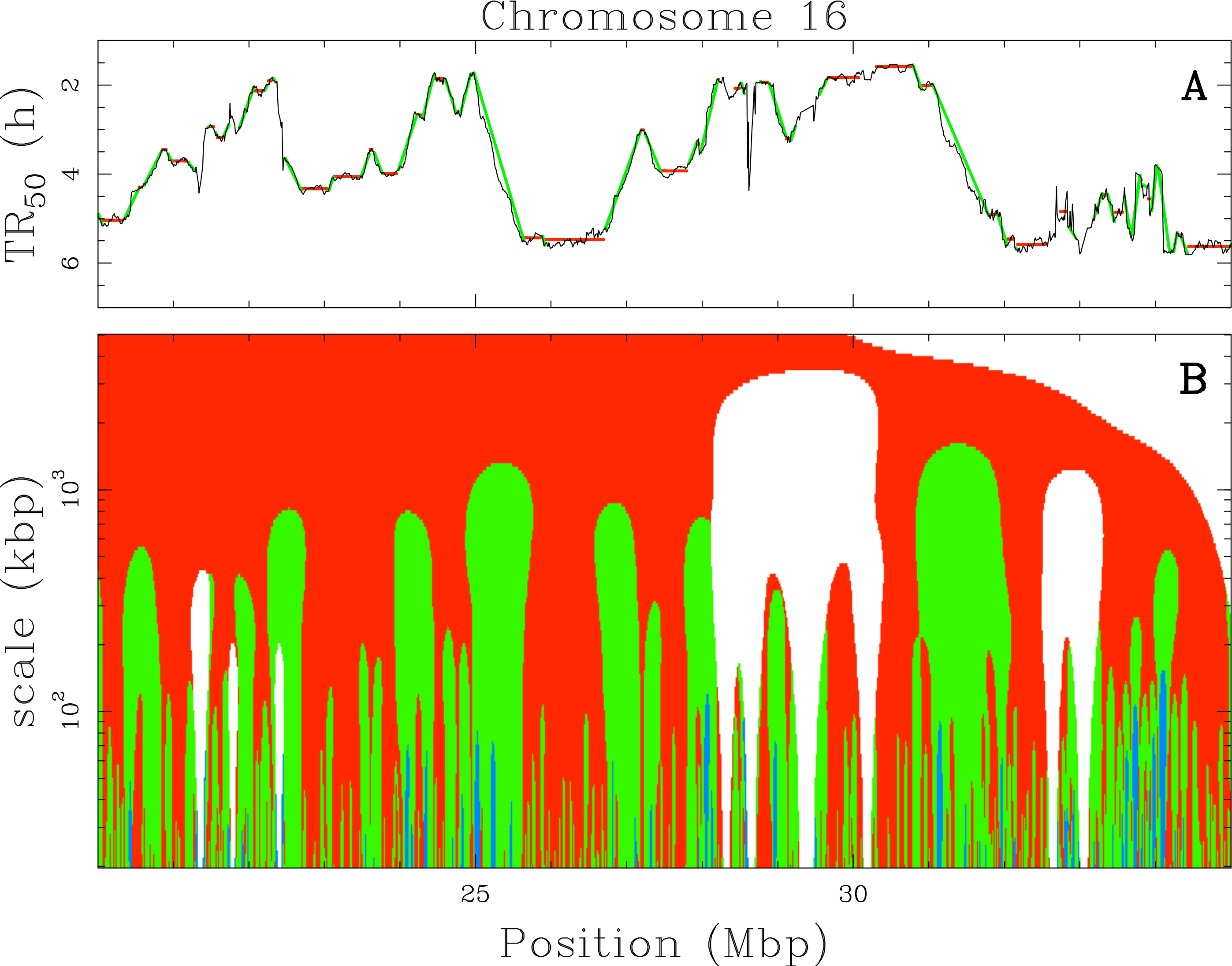
Chromosome 16
A
B
TR$_{50}$ (h)
2
4
6
scale (kbp)
10^3
10^2
25
30
Position (Mbp)

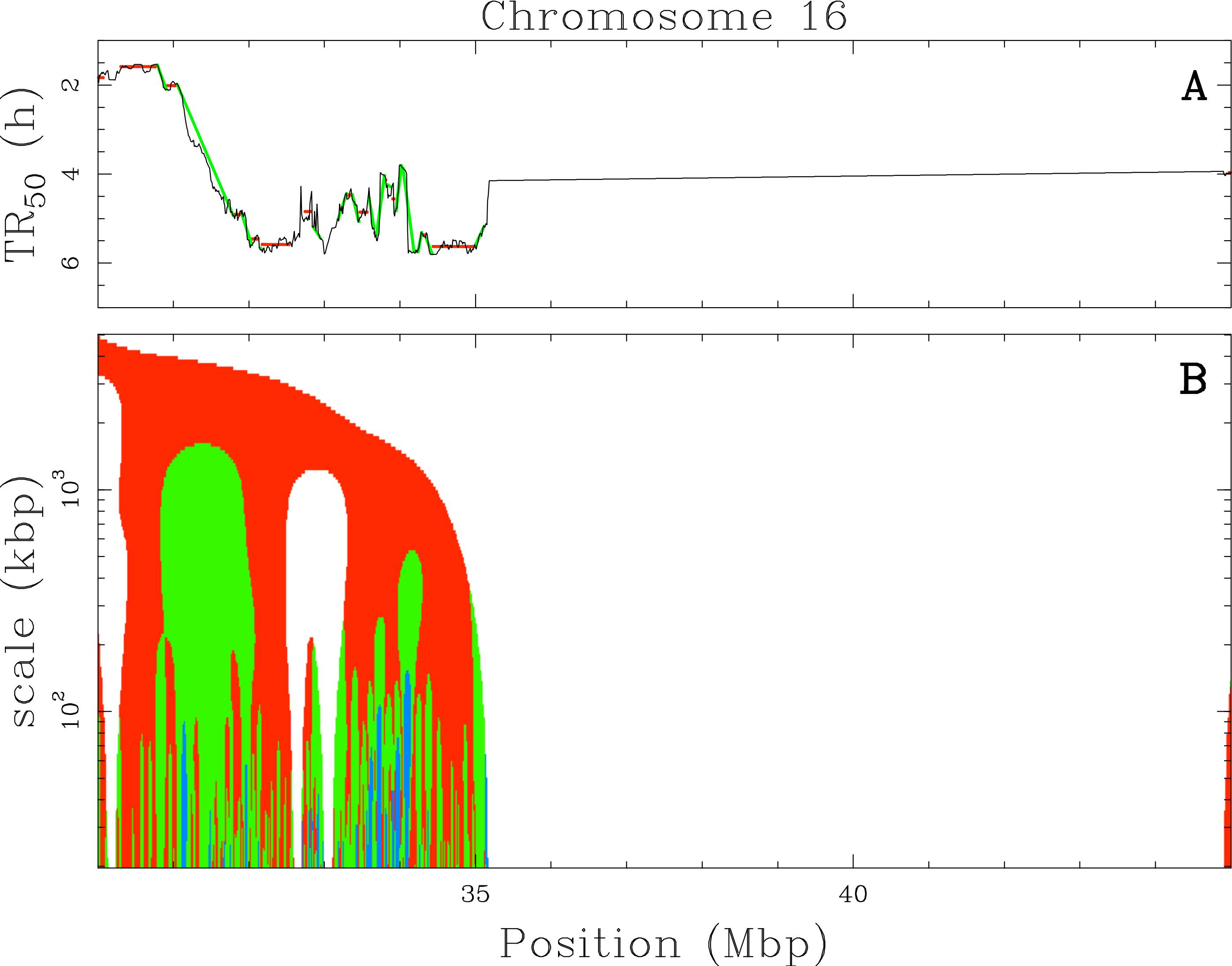

Chromosome 16
TR50 (h)
2
4
6
A
B
scale (kbp)
10^3
10^2
35
40
Position (Mbp)

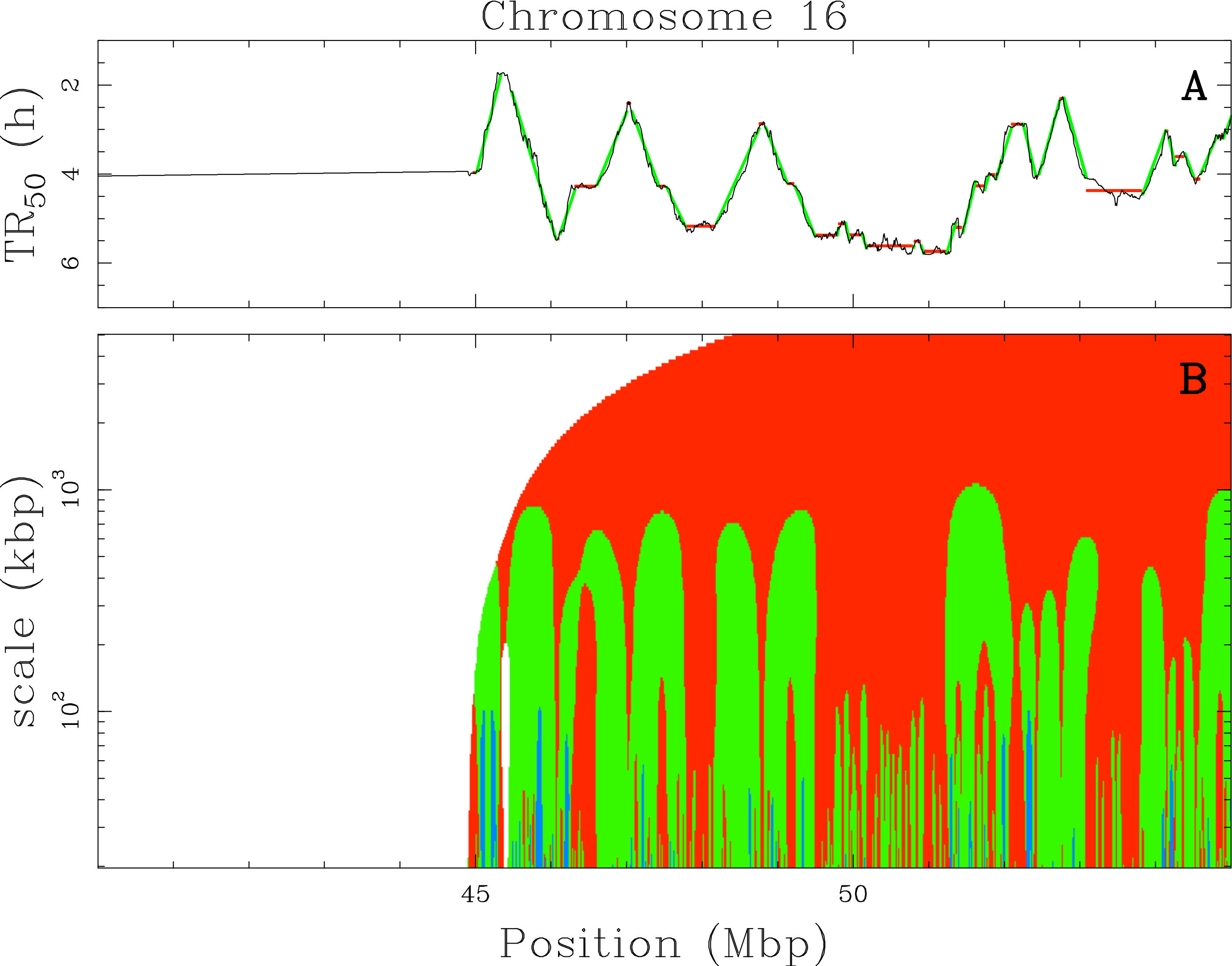

Chromosome 16
A
B
TR50 (h)
2
4
6
scale (kbp)
10^3
10^2
45
50
Position (Mbp)

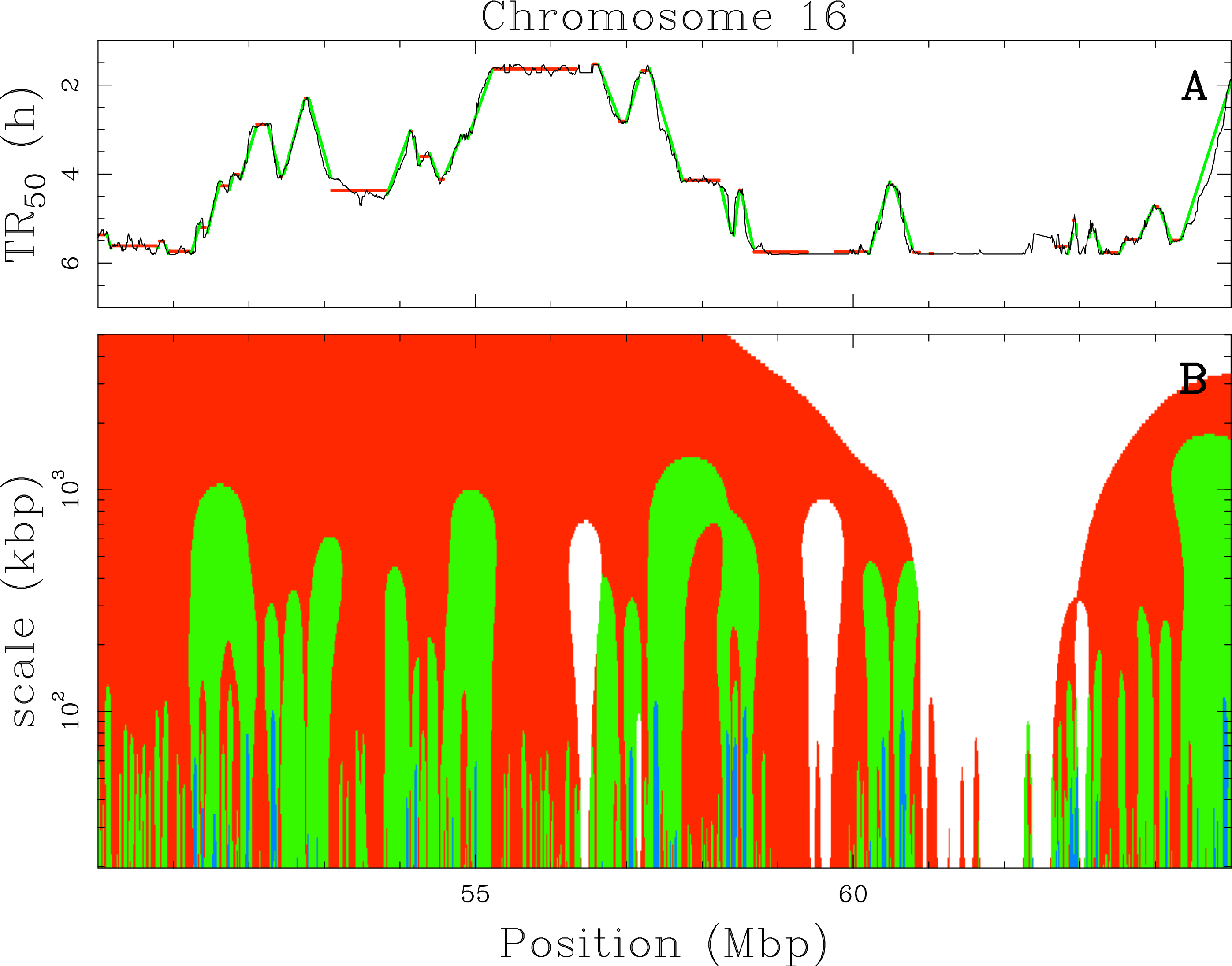

Chromosome 16
A
TR50 (h)
2
4
6
B
scale (kbp)
10^3
10^2
55
60
Position (Mbp)

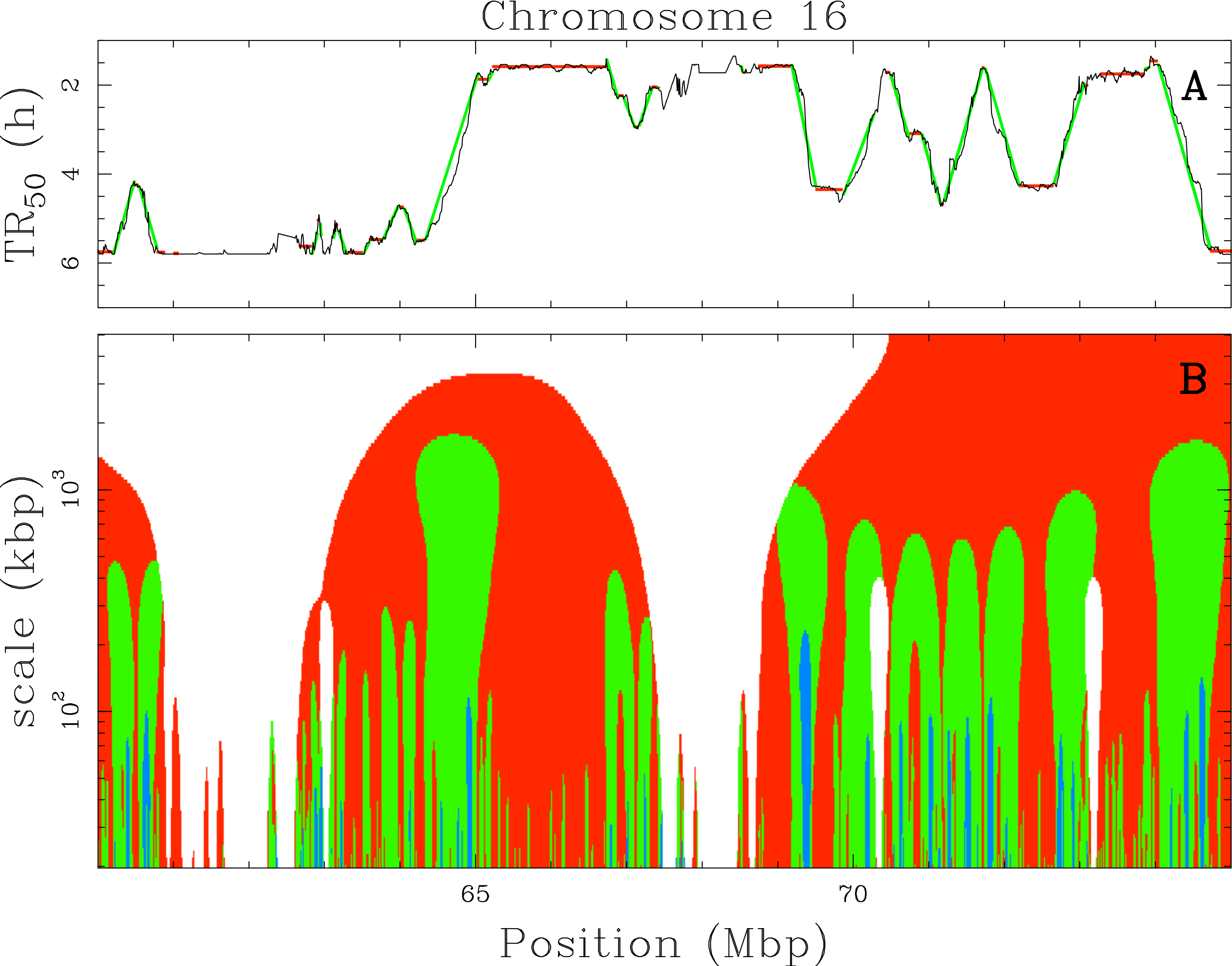

Chromosome 16
TR$_{50}$ (h)
2
4
6
A
B
scale (kbp)
10^3
10^2
65
70
Position (Mbp)

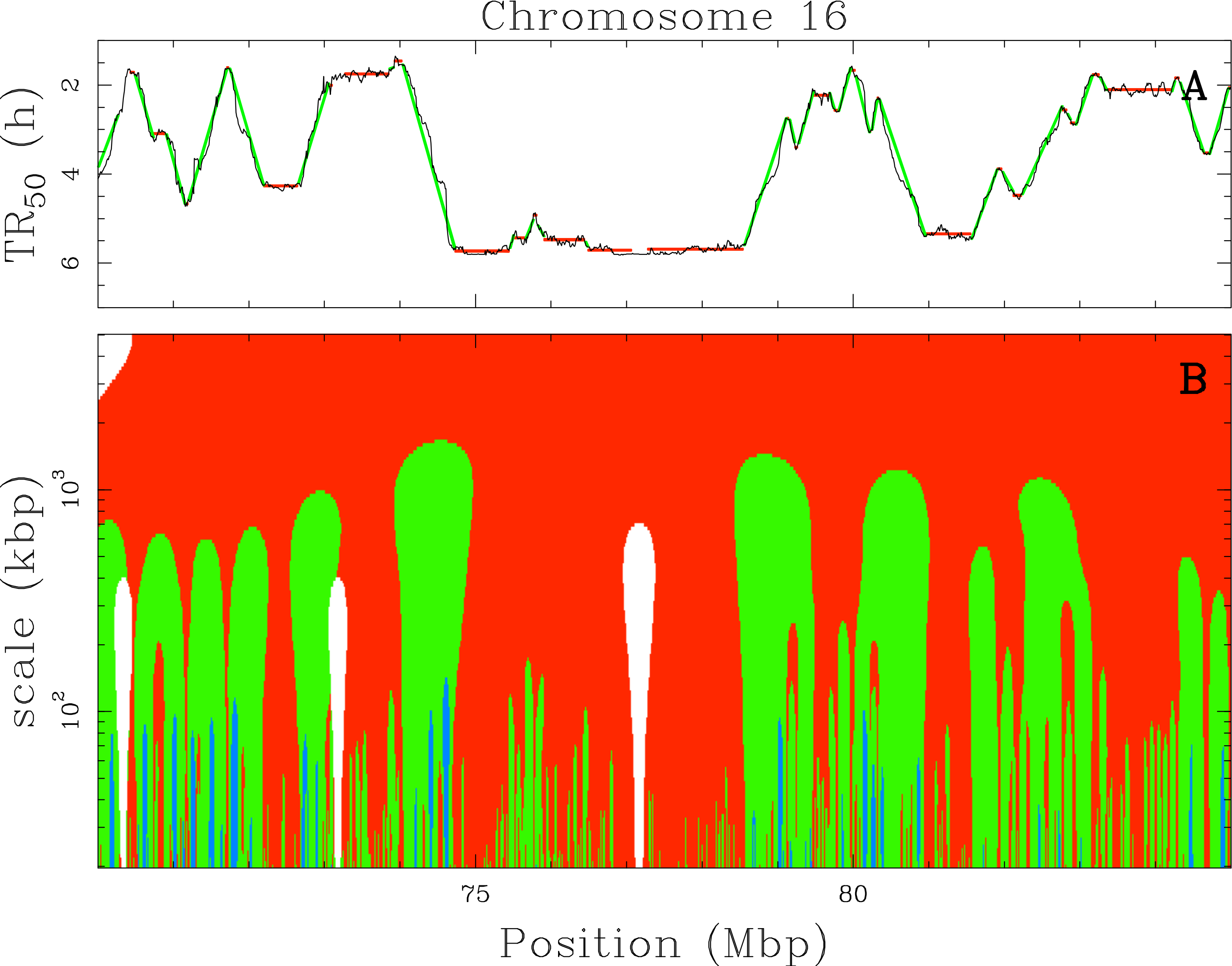
Chromosome 16
TR50 (h)
2
4
6
A
B
scale (kbp)
10^3
10^2
75
80
Position (Mbp)

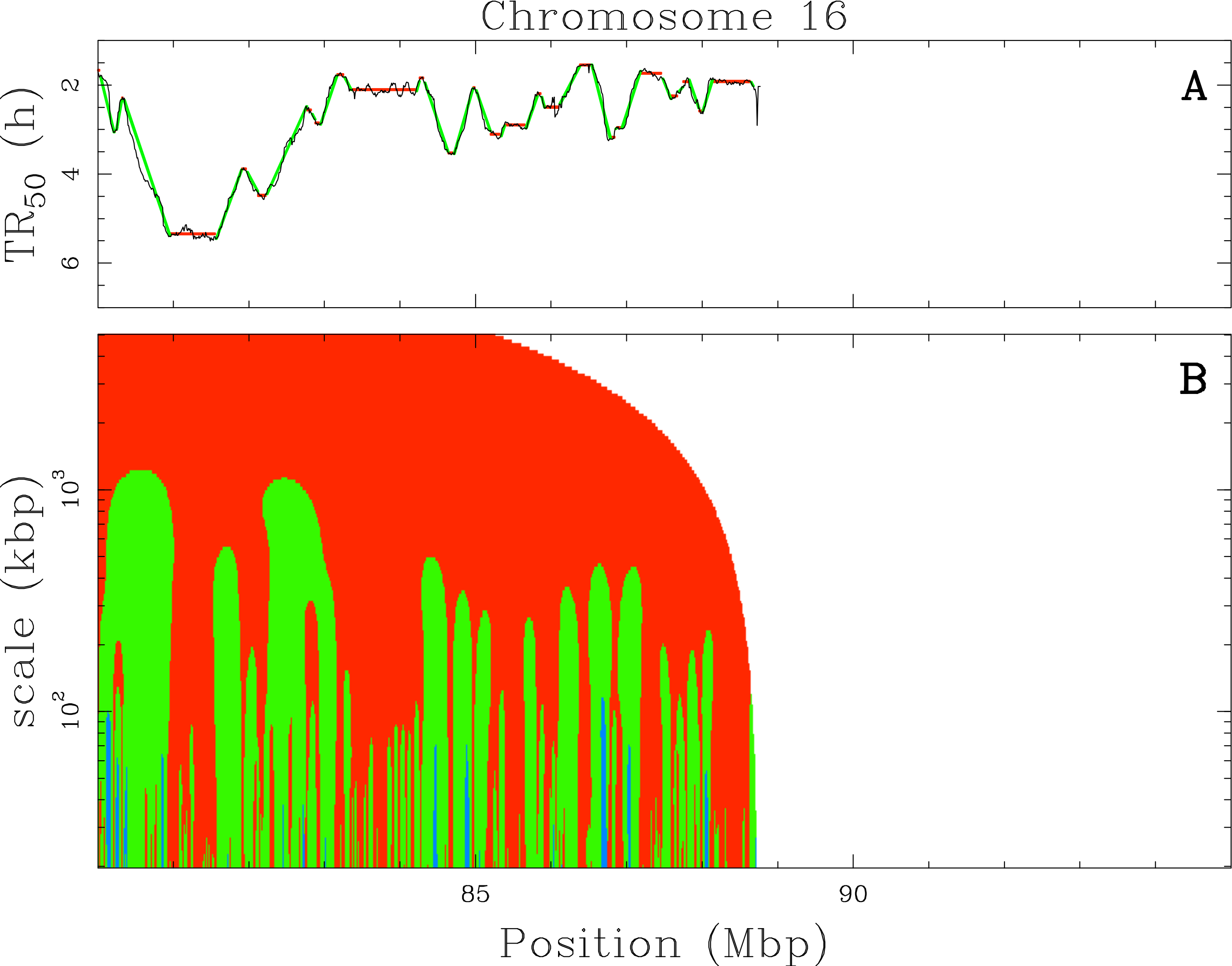
Chromosome 16
TR50 (h)
2
4
6
A
B
scale (kbp)
10^3
10^2
85
90
Position (Mbp)

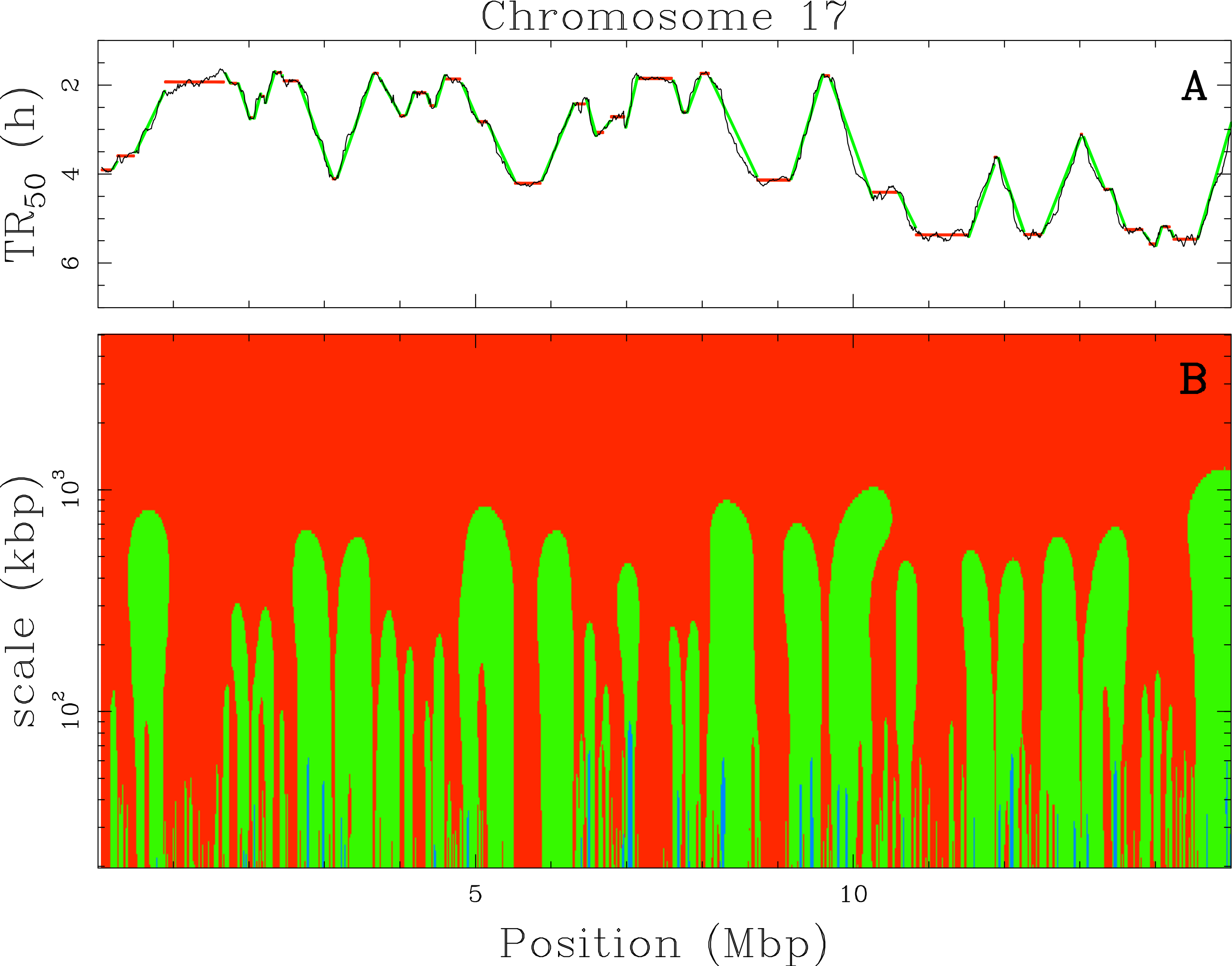

Chromosome 17
A
TR50 (h)
2
4
6
B
scale (kbp)
10^3
10^2
5
10
Position (Mbp)

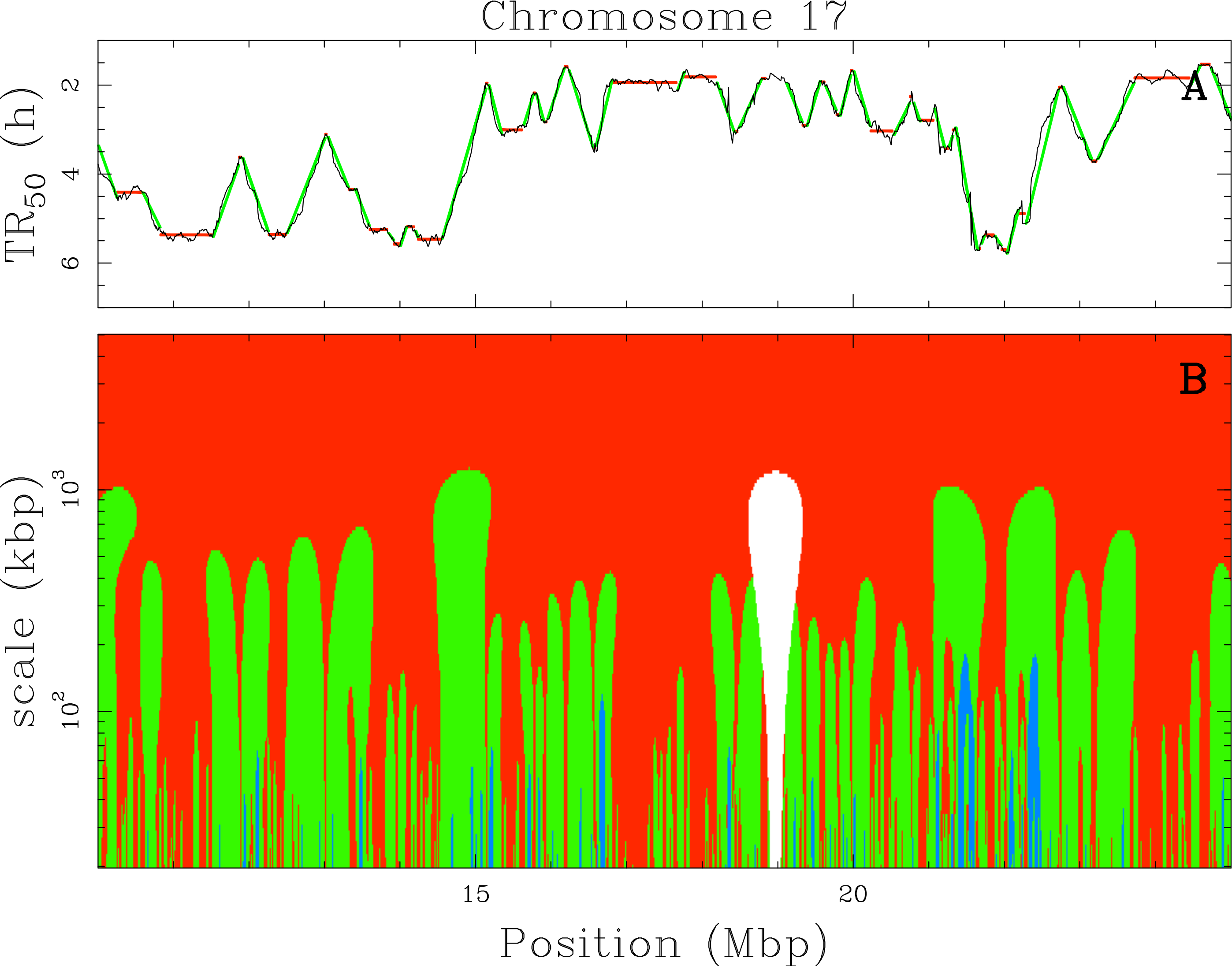

Chromosome 17
A
B
TR_{50} (h)
2
4
6
scale (kbp)
10^3
10^2
15
20
Position (Mbp)

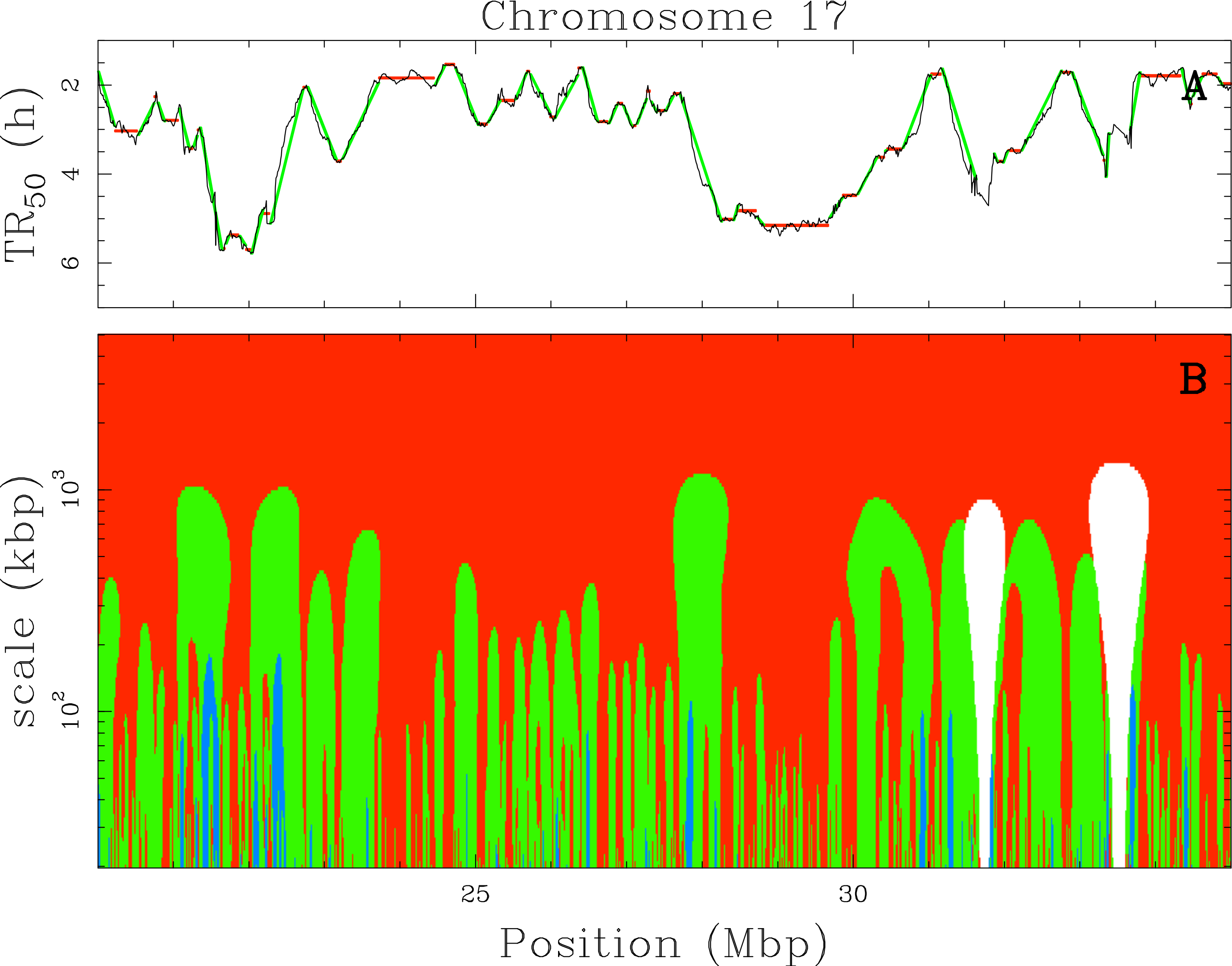

Chromosome 17
TR50 (h)
2
4
6
A
B
scale (kbp)
10^3
10^2
25
30
Position (Mbp)

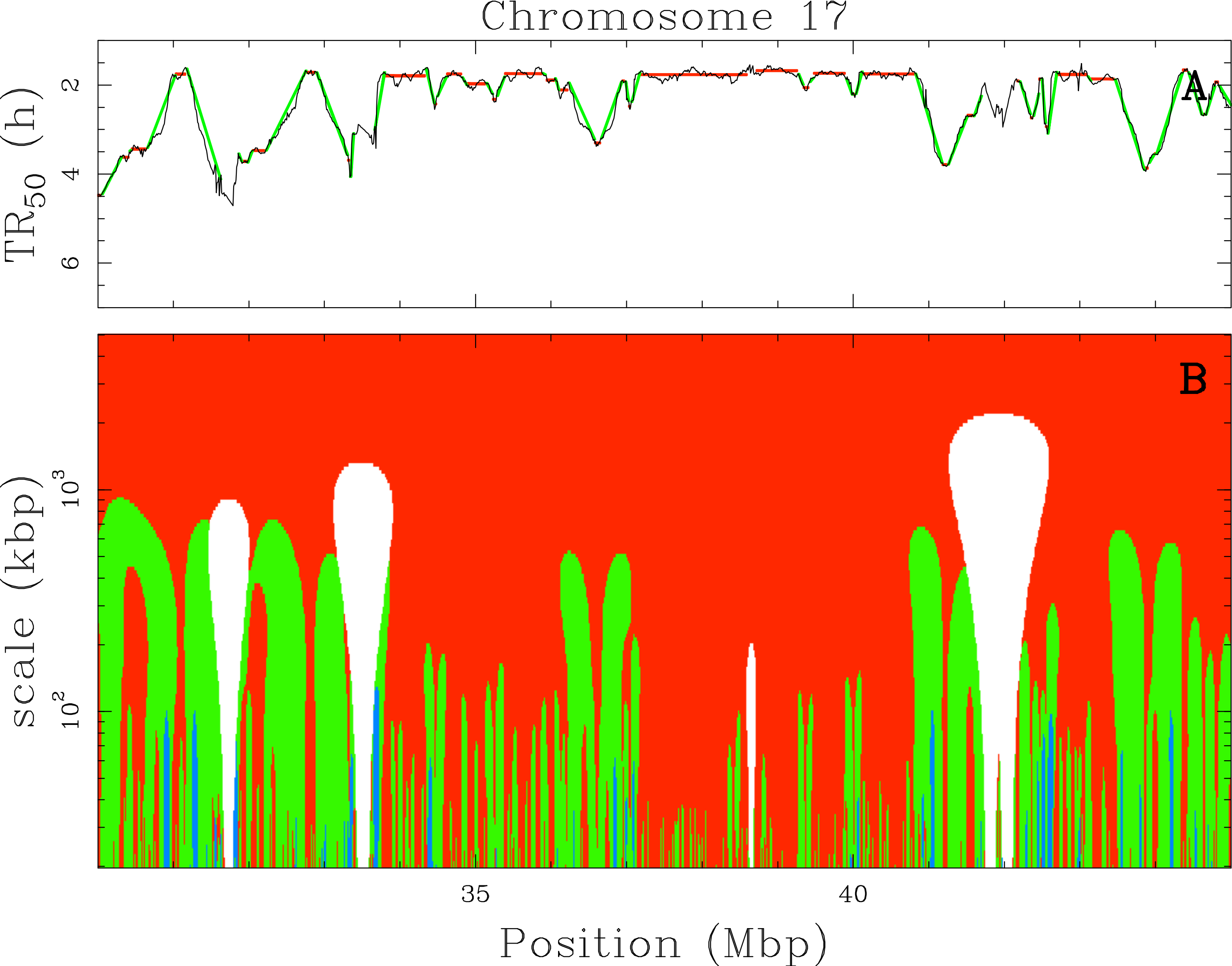
Chromosome 17
TR50 (h)
2
4
6
A
B
scale (kbp)
10^3
10^2
35
40
Position (Mbp)

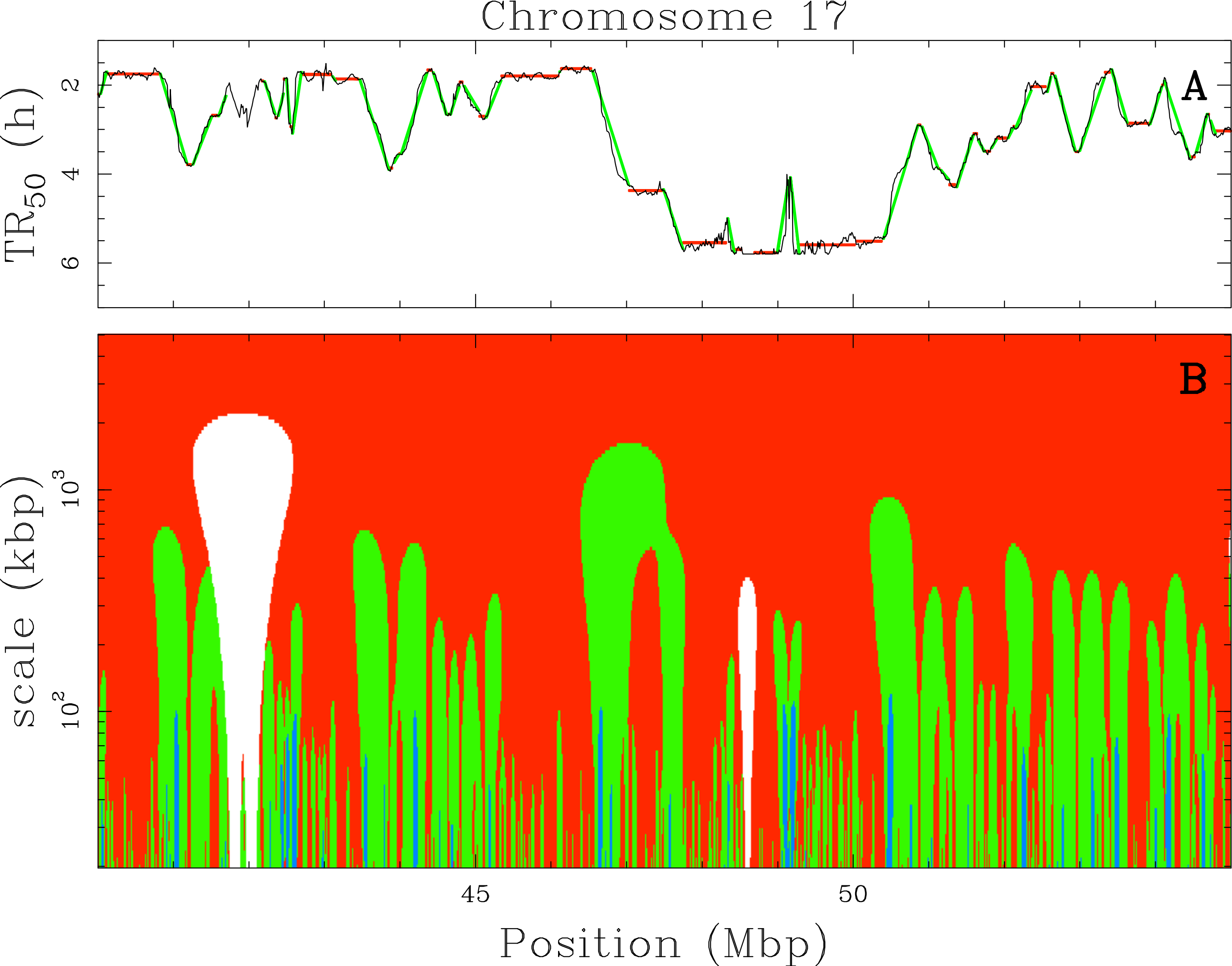

Chromosome 17
A
B
TR50 (h)
2
4
6
scale (kbp)
10^3
10^2
45
50
Position (Mbp)

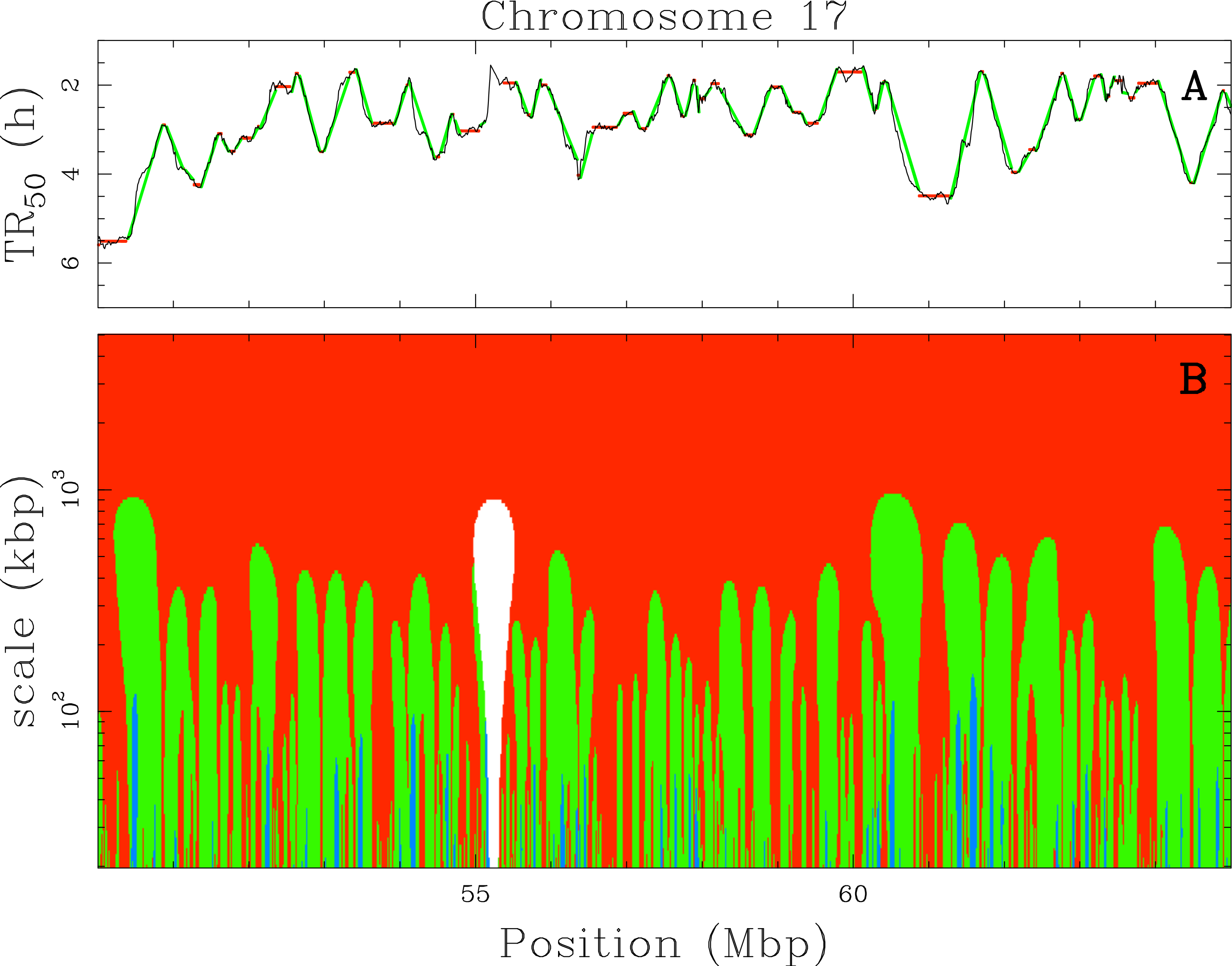
Chromosome 17
TR50 (h)
2
4
6
A
B
scale (kbp)
10^3
10^2
55
60
Position (Mbp)

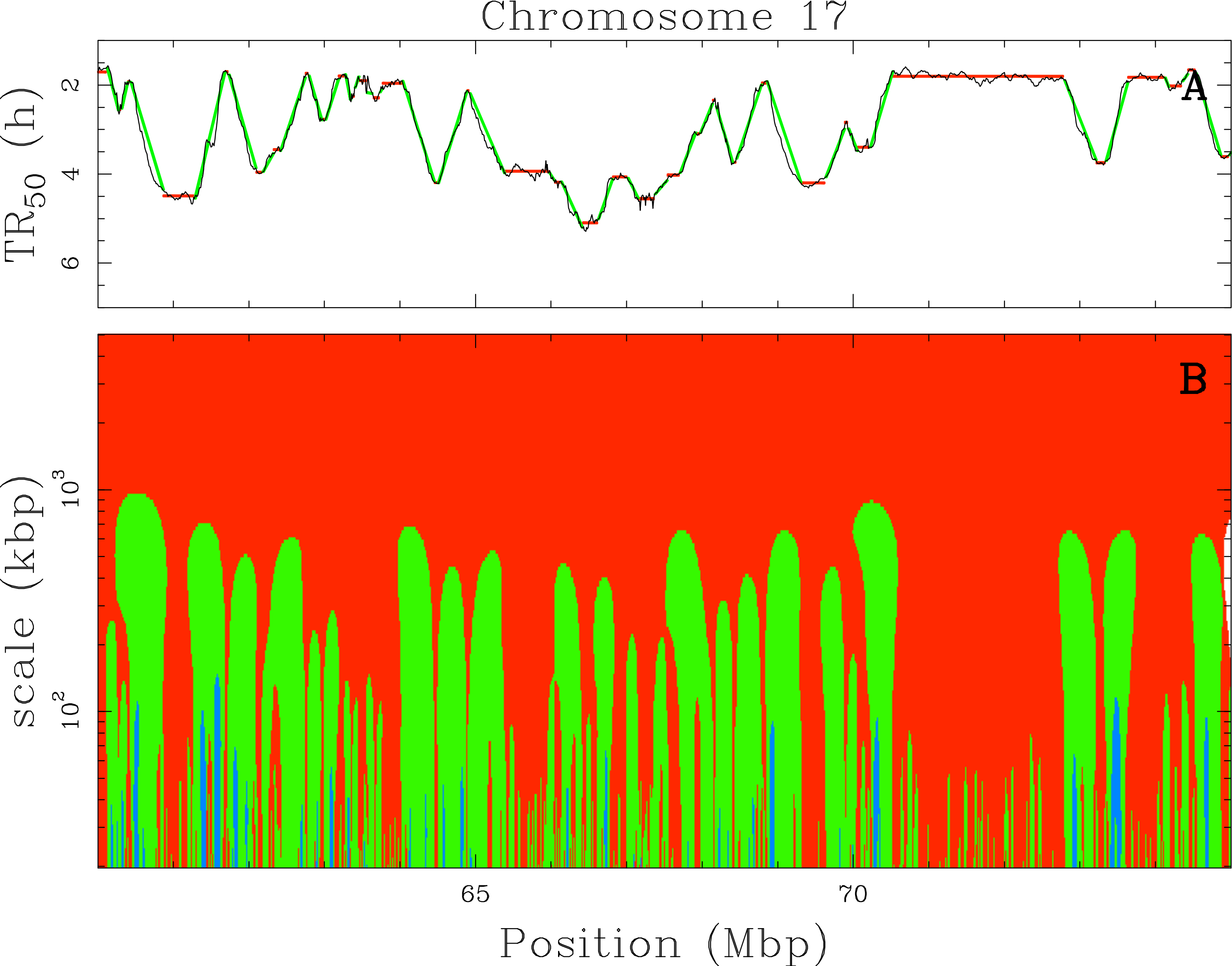

Chromosome 17
TR_{50} (h)
2
4
6
A
B
scale (kbp)
10^3
10^2
65
70
Position (Mbp)

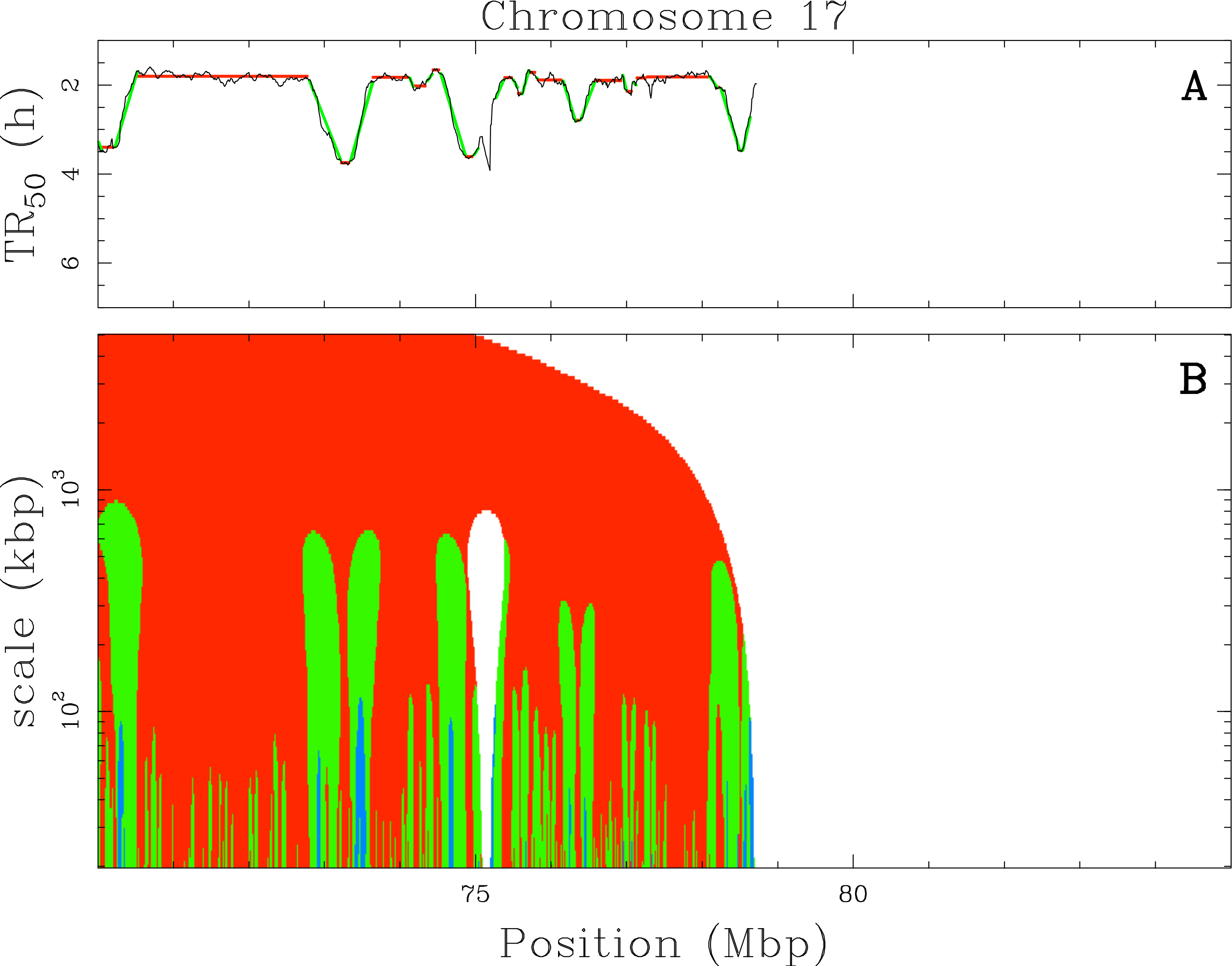

Chromosome 17
TR50 (h)
2
4
6
A
scale (kbp)
10^3
10^2
B
75
80
Position (Mbp)

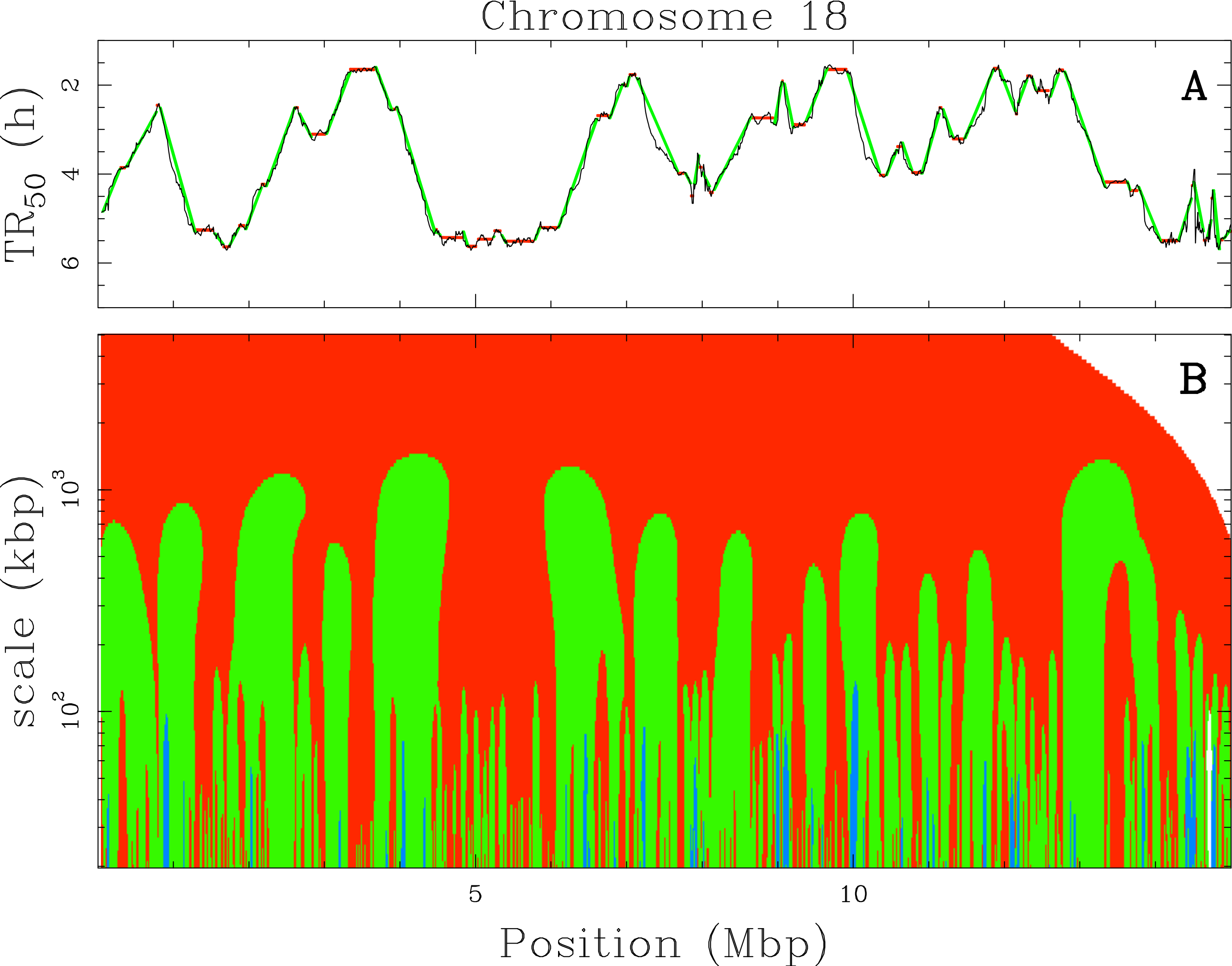

Chromosome 18
A
B
TR50 (h)
2
4
6
scale (kbp)
10^3
10^2
5
10
Position (Mbp)

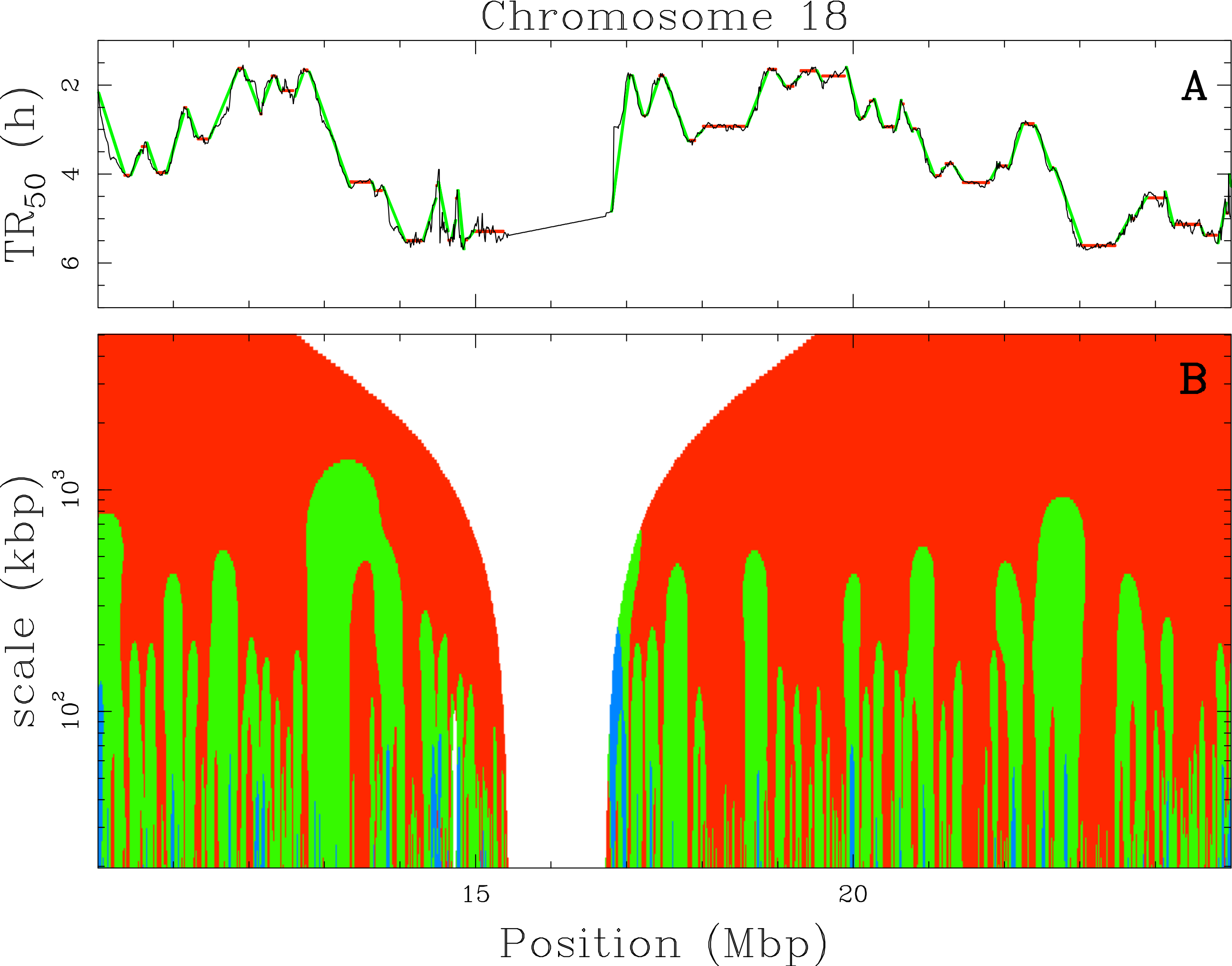

Chromosome 18
A
B
TR50 (h)
2
4
6
scale (kbp)
10^3
10^2
15
20
Position (Mbp)

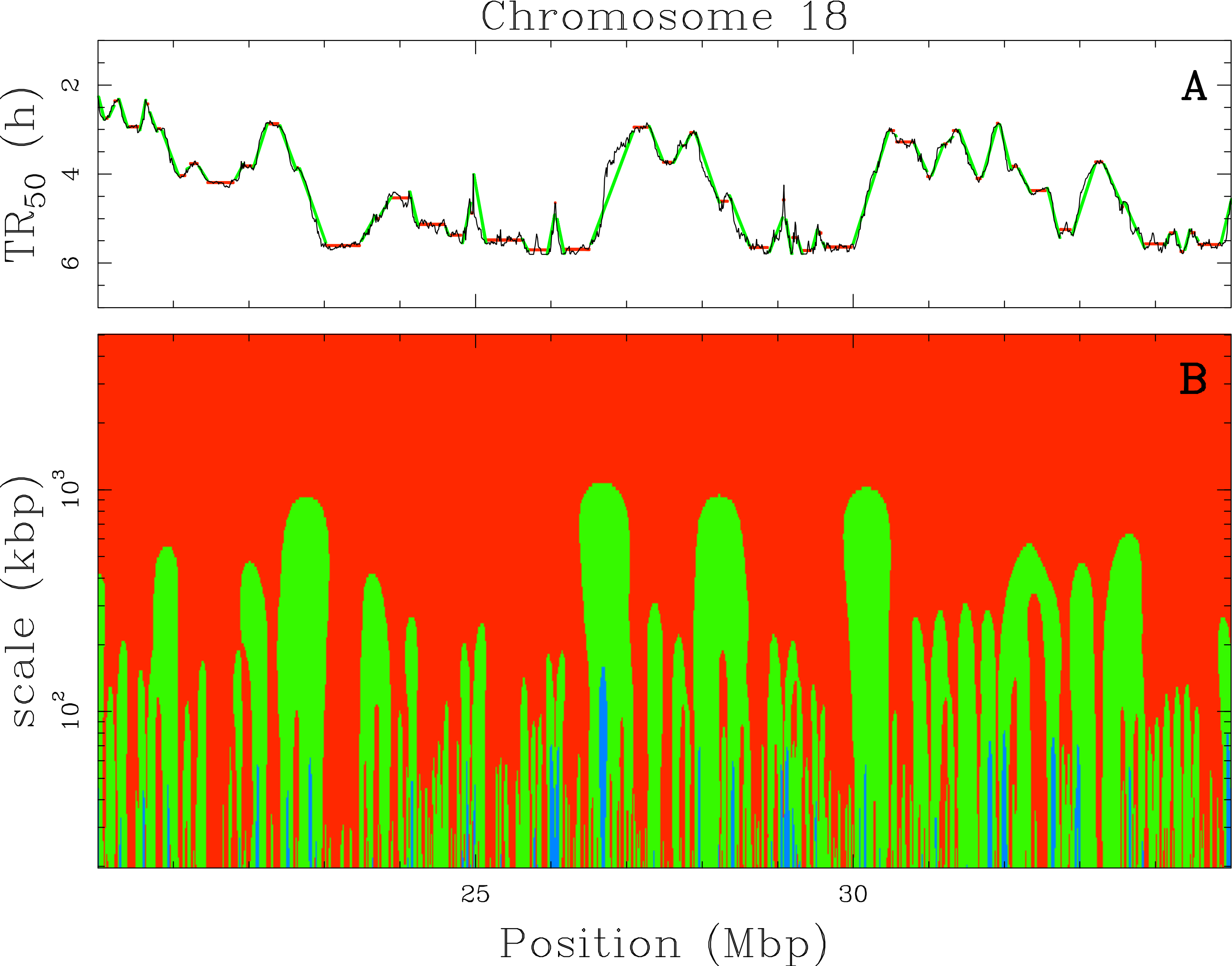

Chromosome 18
A
B
TR_{50} (h)
2
4
6
scale (kbp)
10^3
10^2
25
30
Position (Mbp)

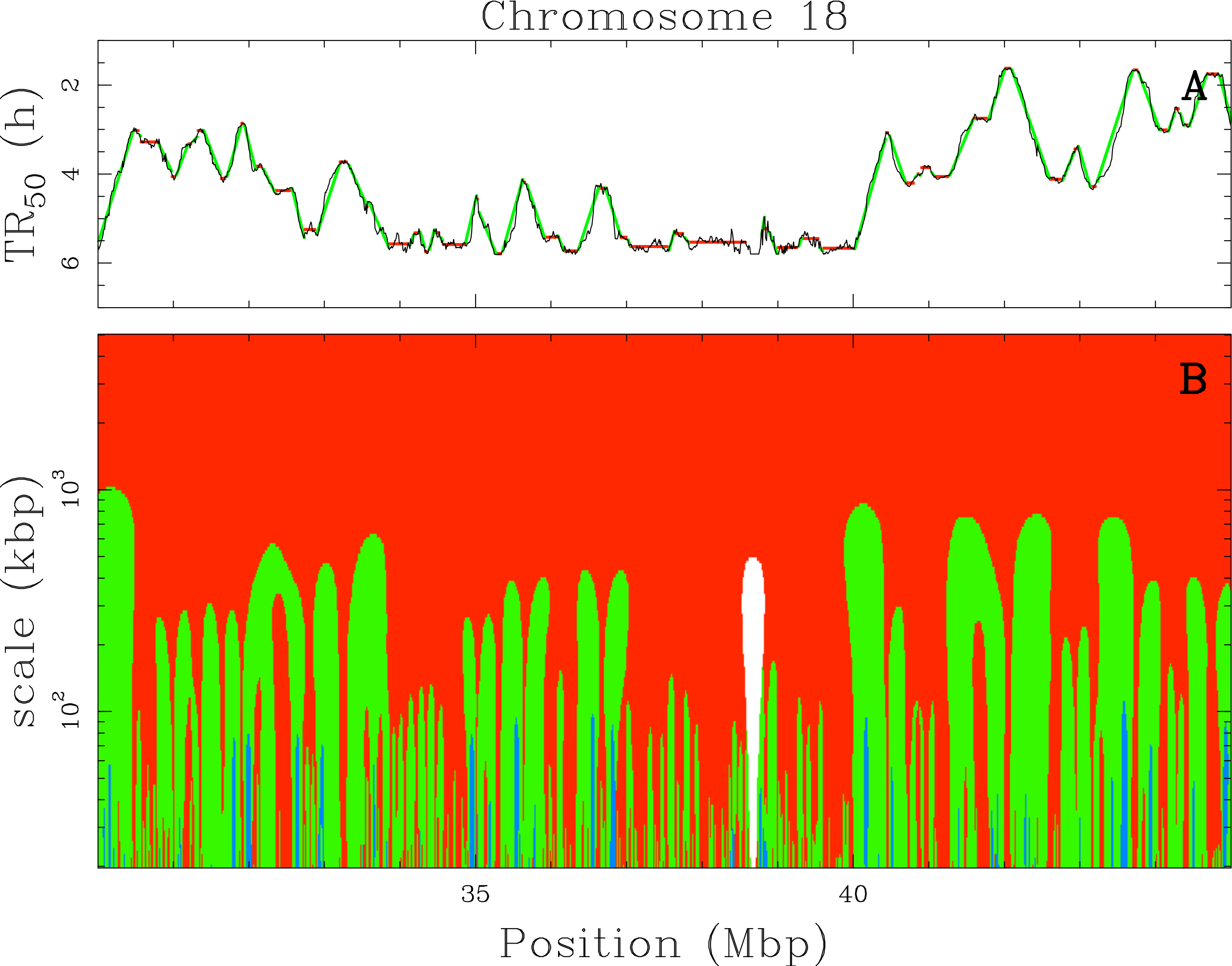
Chromosome 18
A
B
TR50 (h)
2
4
6
scale (kbp)
10^3
10^2
35
40
Position (Mbp)

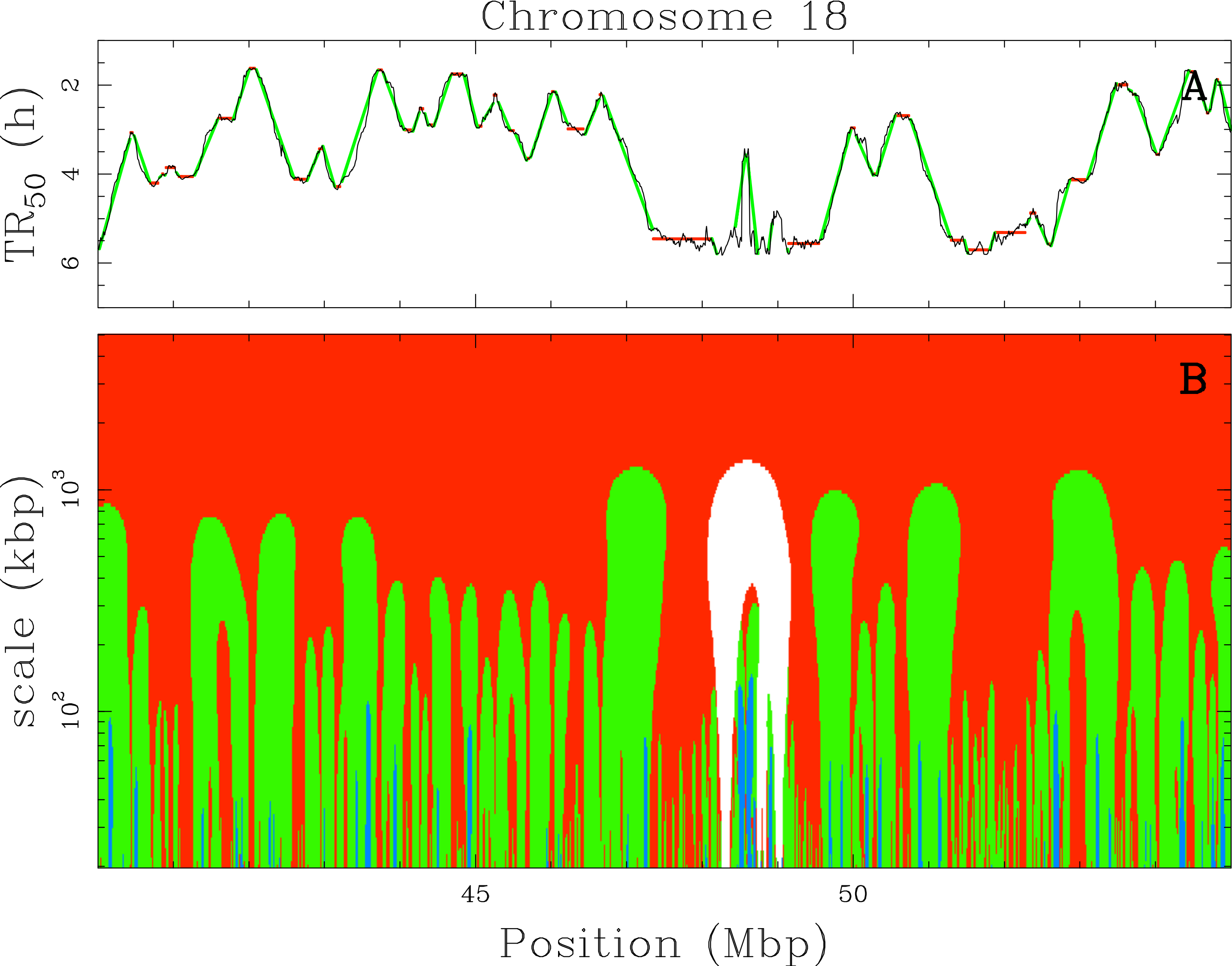
Chromosome 18
A
B
TR50 (h)
2
4
6
scale (kbp)
10^3
10^2
45
50
Position (Mbp)

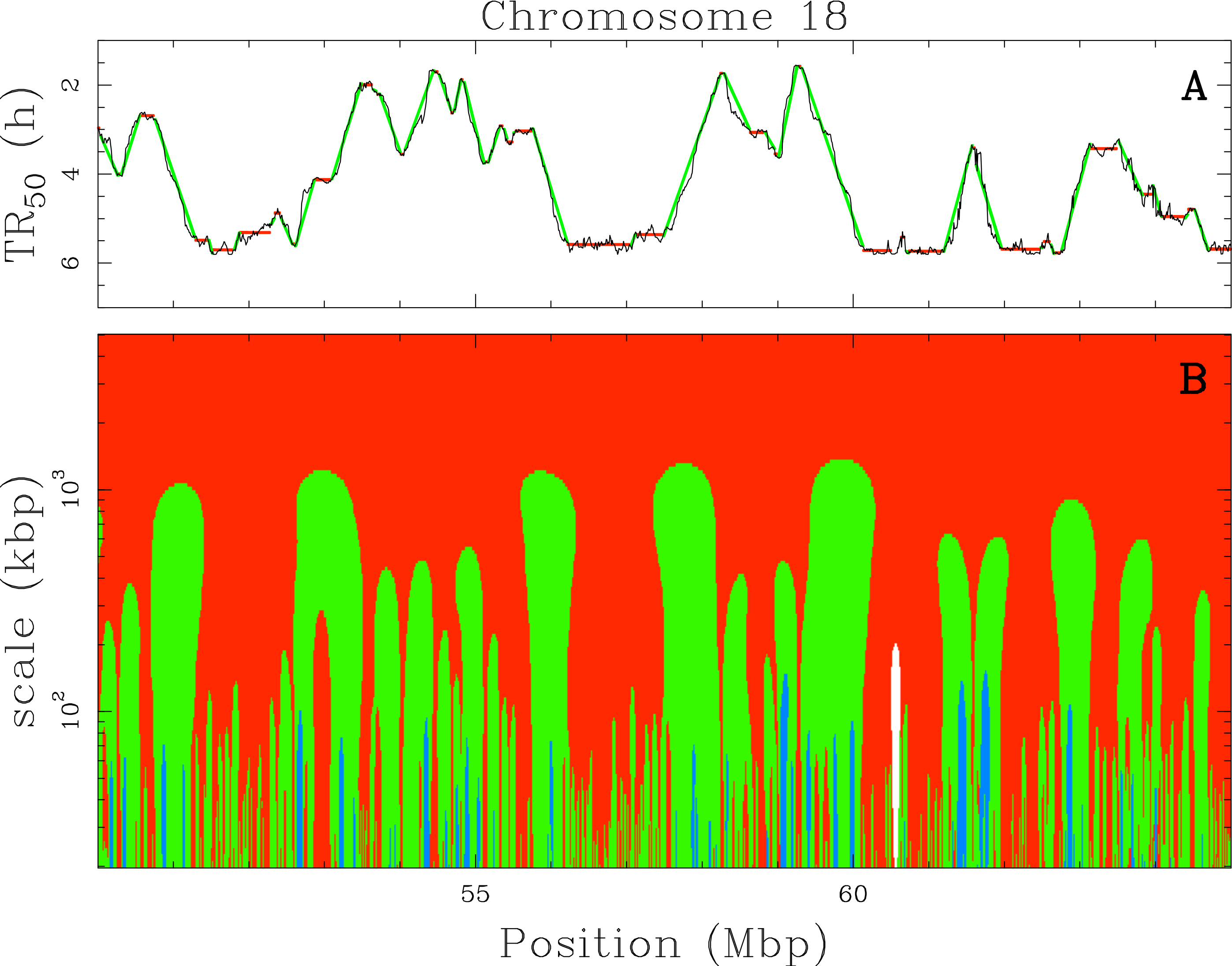
Chromosome 18
A
B
TR50 (h)
2
4
6
scale (kbp)
10^3
10^2
55
60
Position (Mbp)

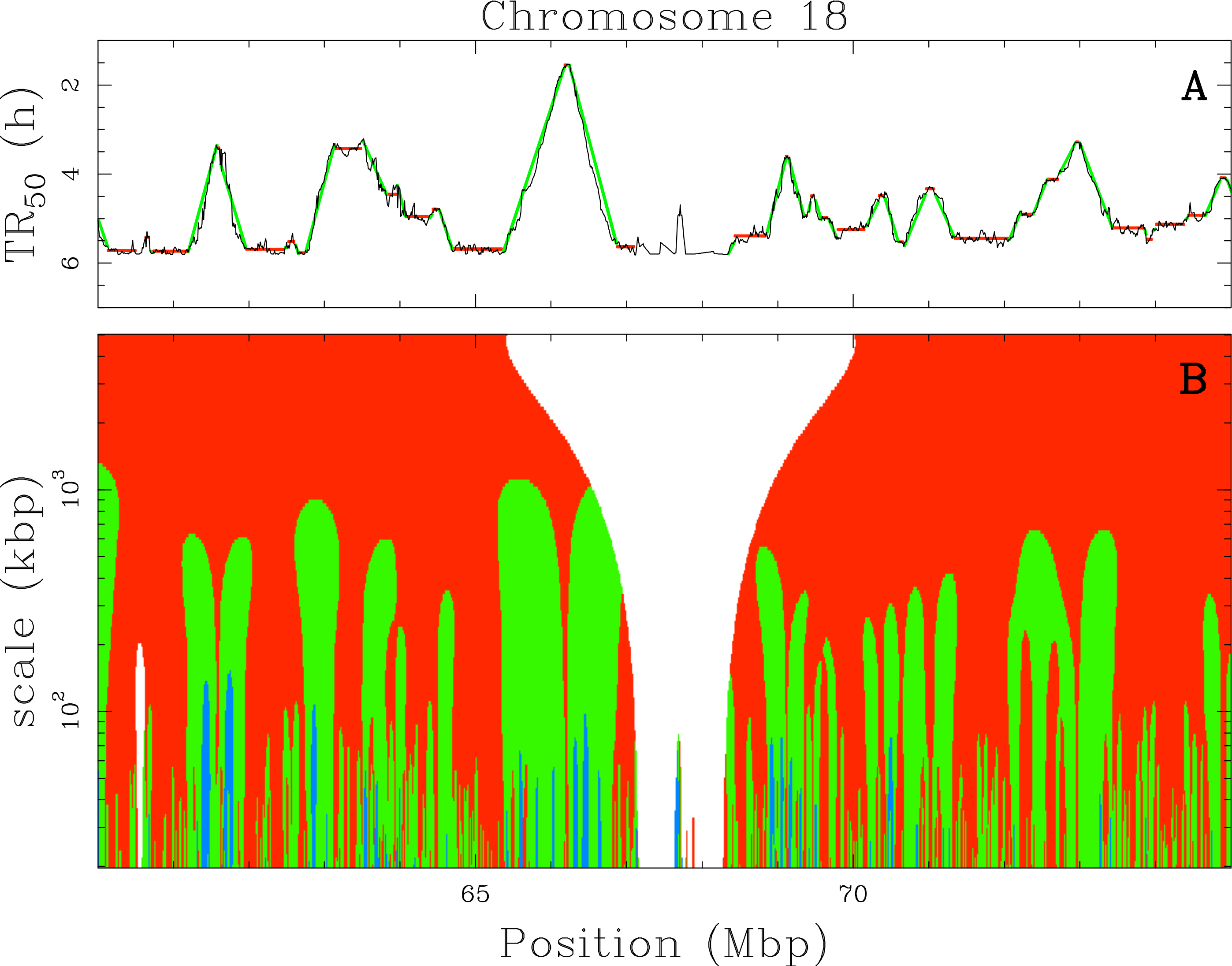

Chromosome 18
A
B
TR_{50} (h)
2
4
6
scale (kbp)
10^3
10^2
65
70
Position (Mbp)

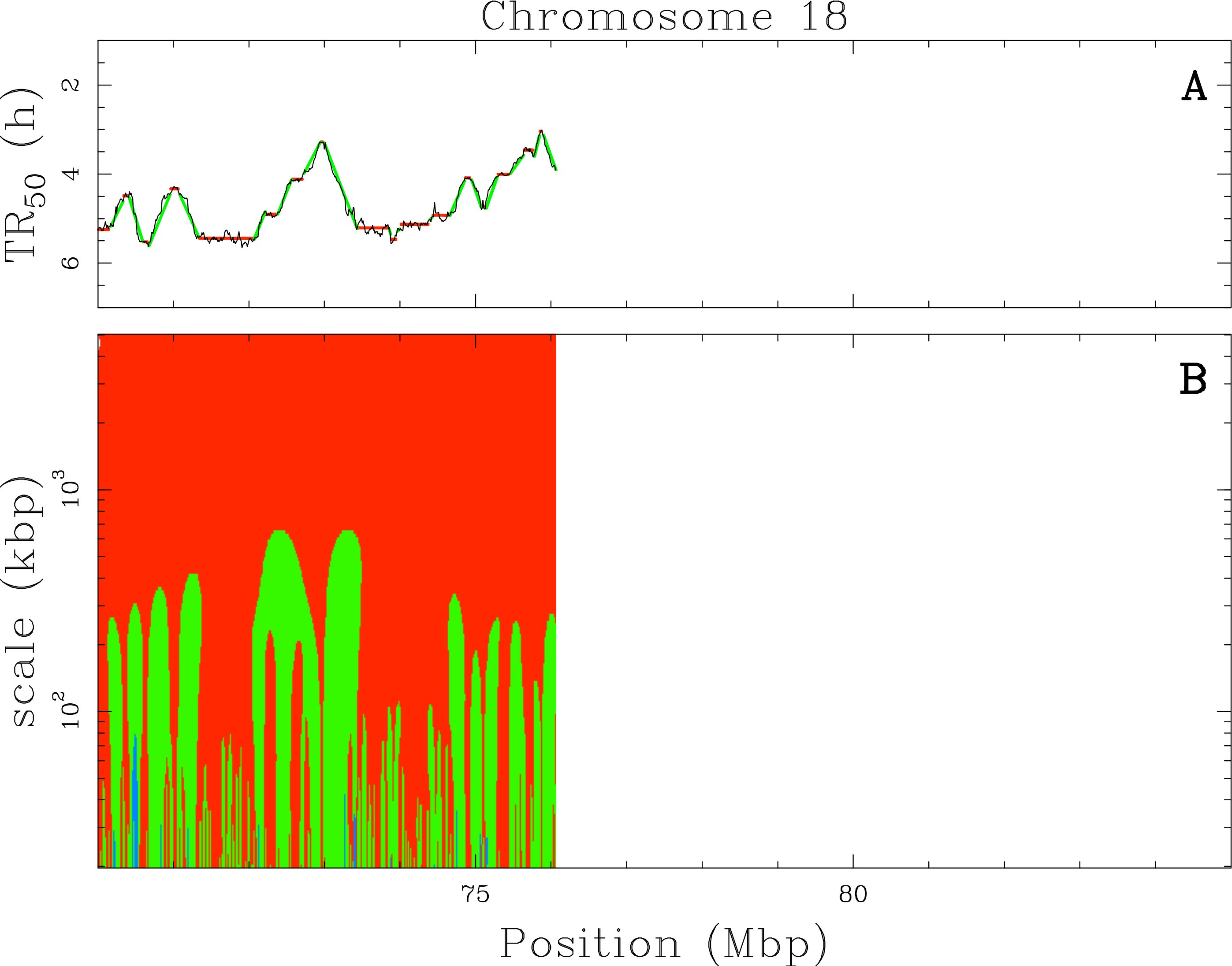
Chromosome 18
A
B
TR50 (h)
2
4
6
scale (kbp)
10^3
10^2
75
80
Position (Mbp)

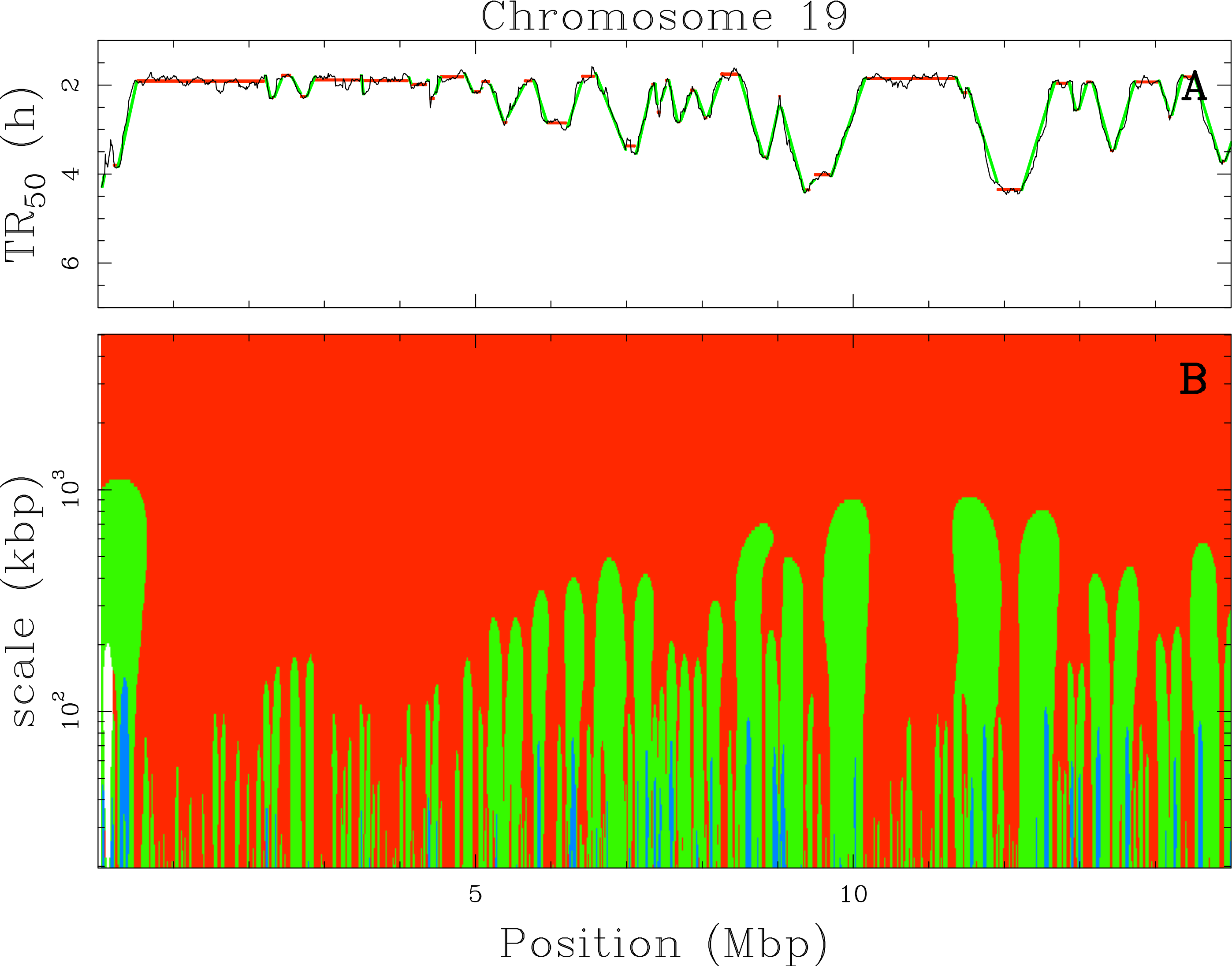

Chromosome 19
A
B
TR_{50} (h)
2
4
6
scale (kbp)
10^3
10^2
5
10
Position (Mbp)

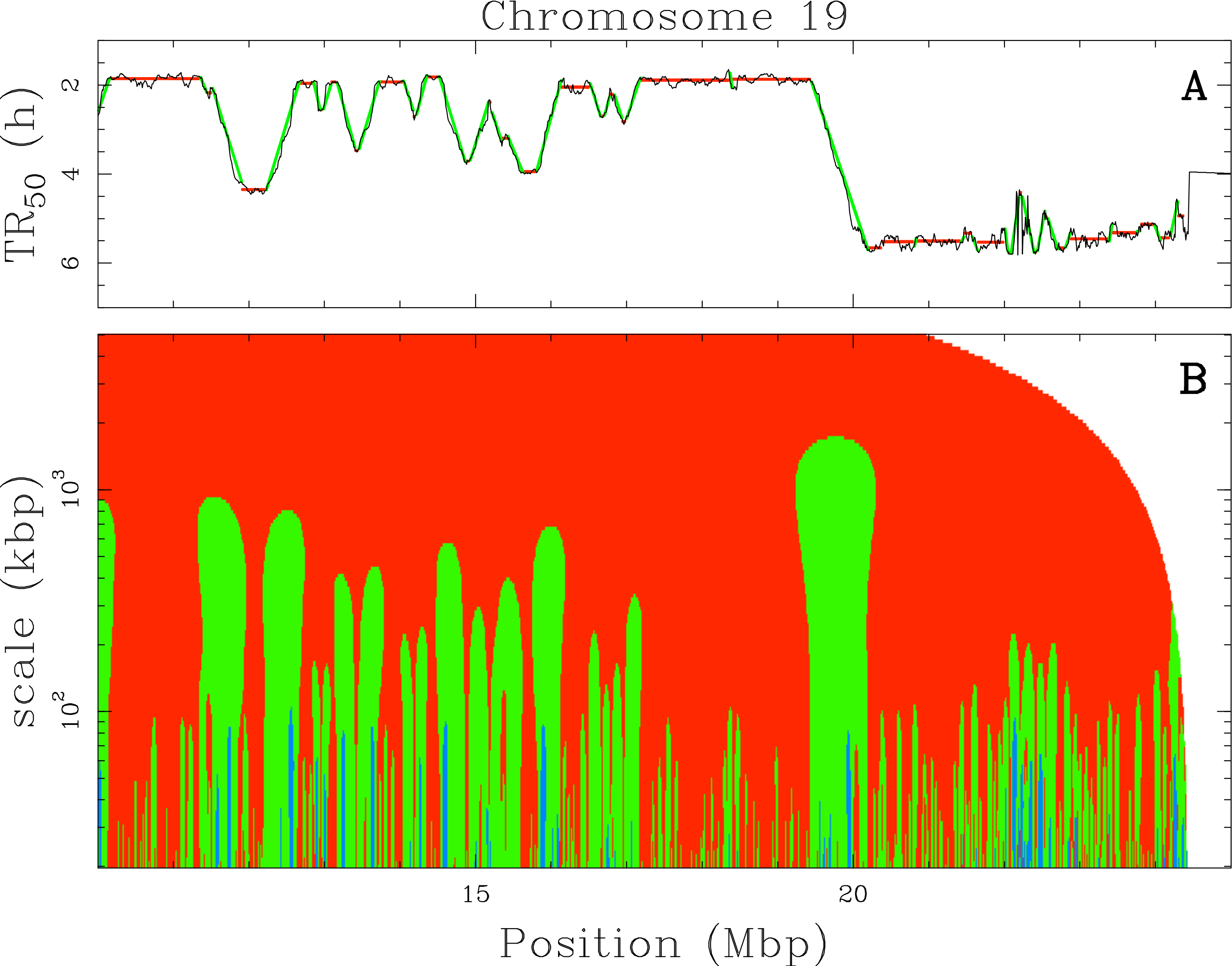

Chromosome 19
A
TR_{50} (h)
2
4
6
B
scale (kbp)
10^3
10^2
15
20
Position (Mbp)

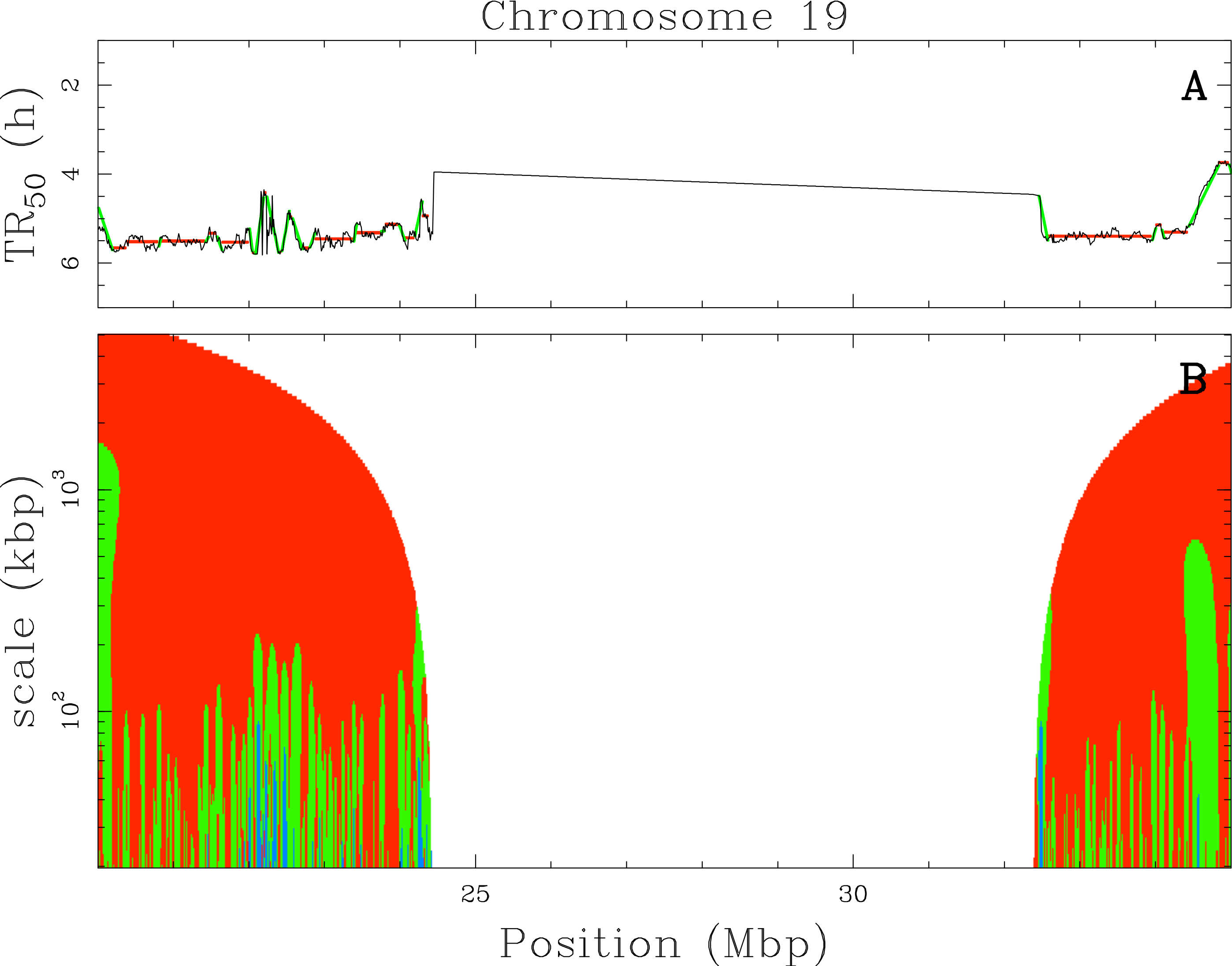

Chromosome 19
A
B
TR50 (h)
2
4
6
scale (kbp)
10^3
10^2
25
30
Position (Mbp)

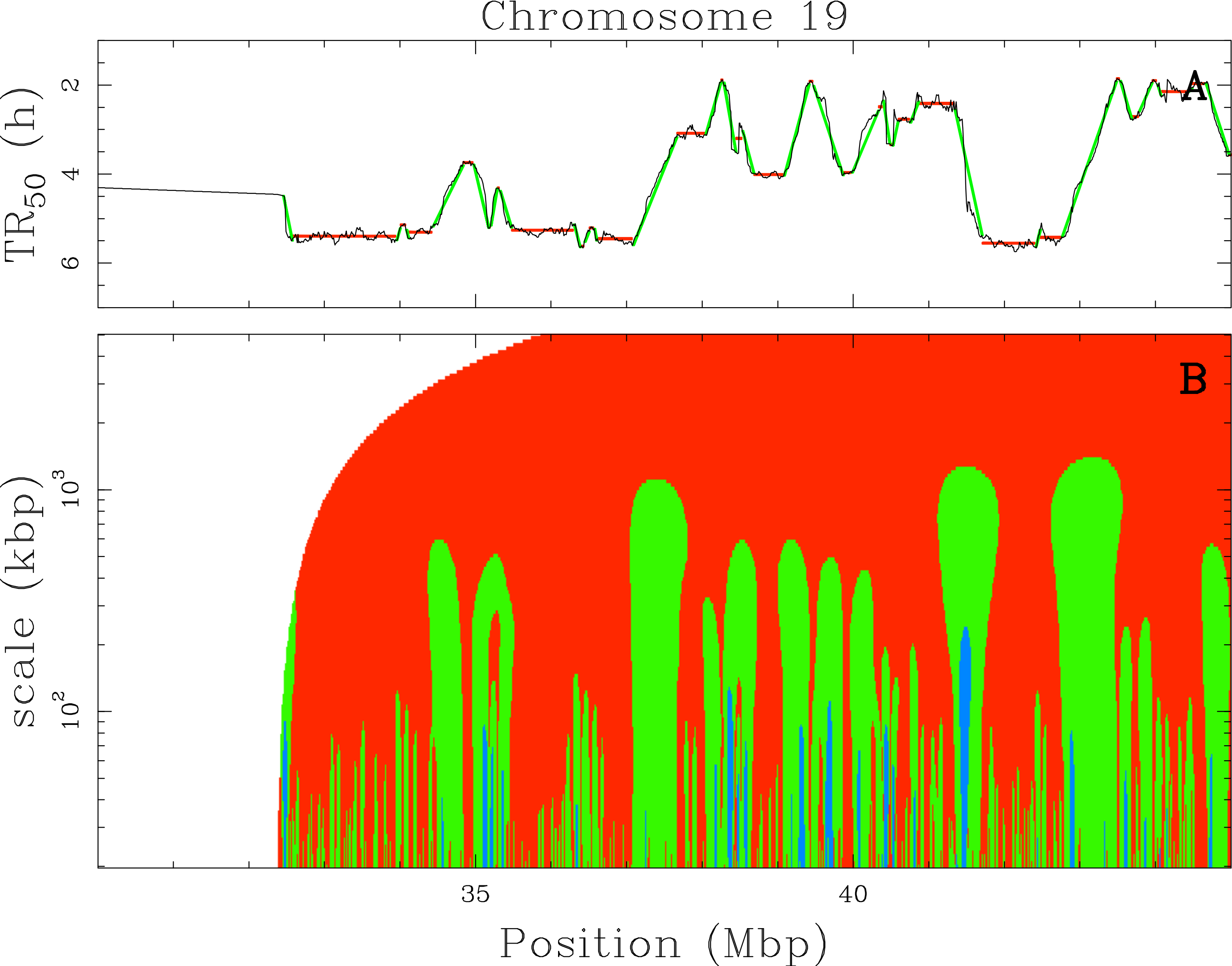

Chromosome 19
A
TR50 (h)
2
4
6
B
scale (kbp)
10^3
10^2
35
40
Position (Mbp)

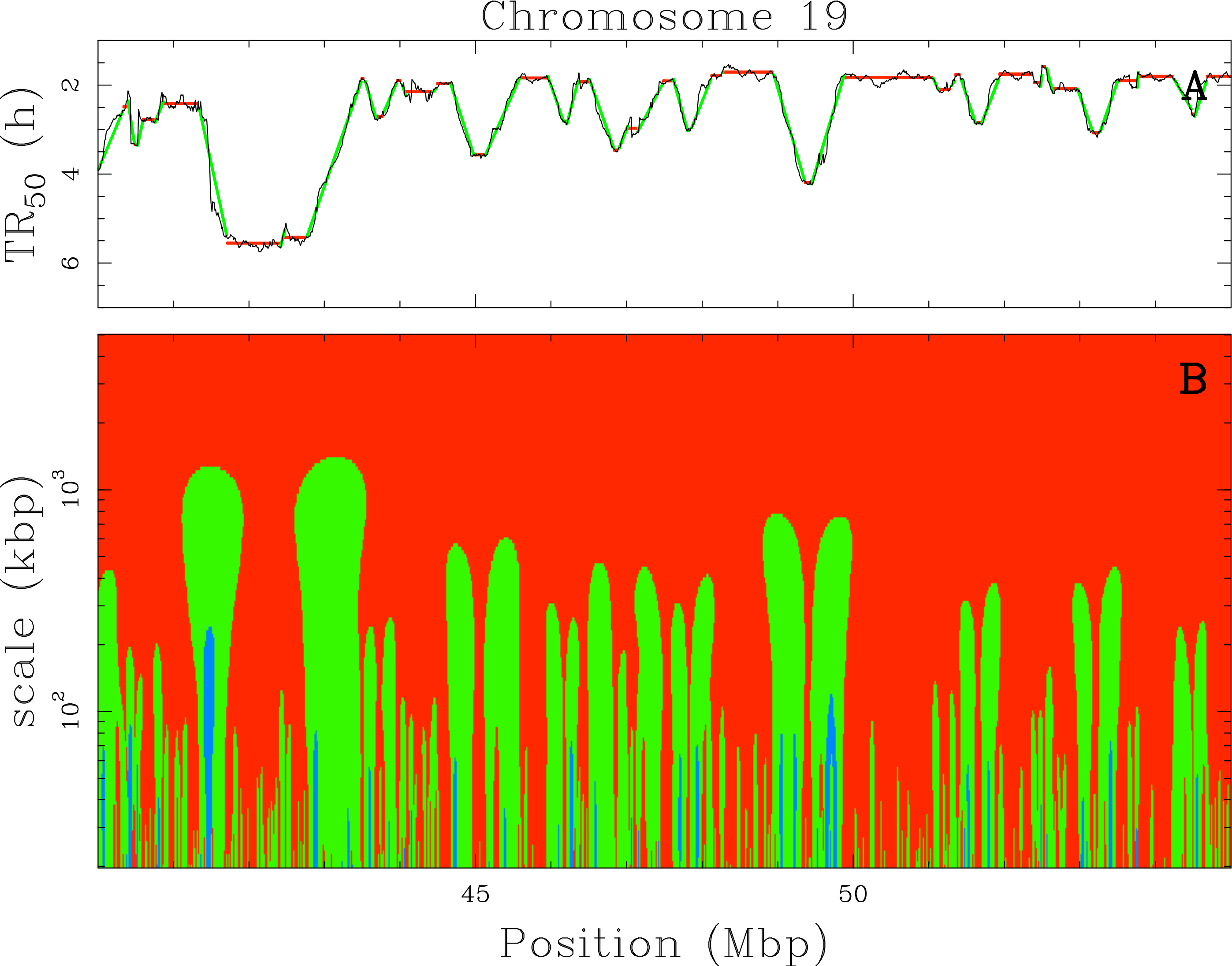

Chromosome 19
A
B
TR50 (h)
2
4
6
scale (kbp)
10^3
10^2
45
50
Position (Mbp)

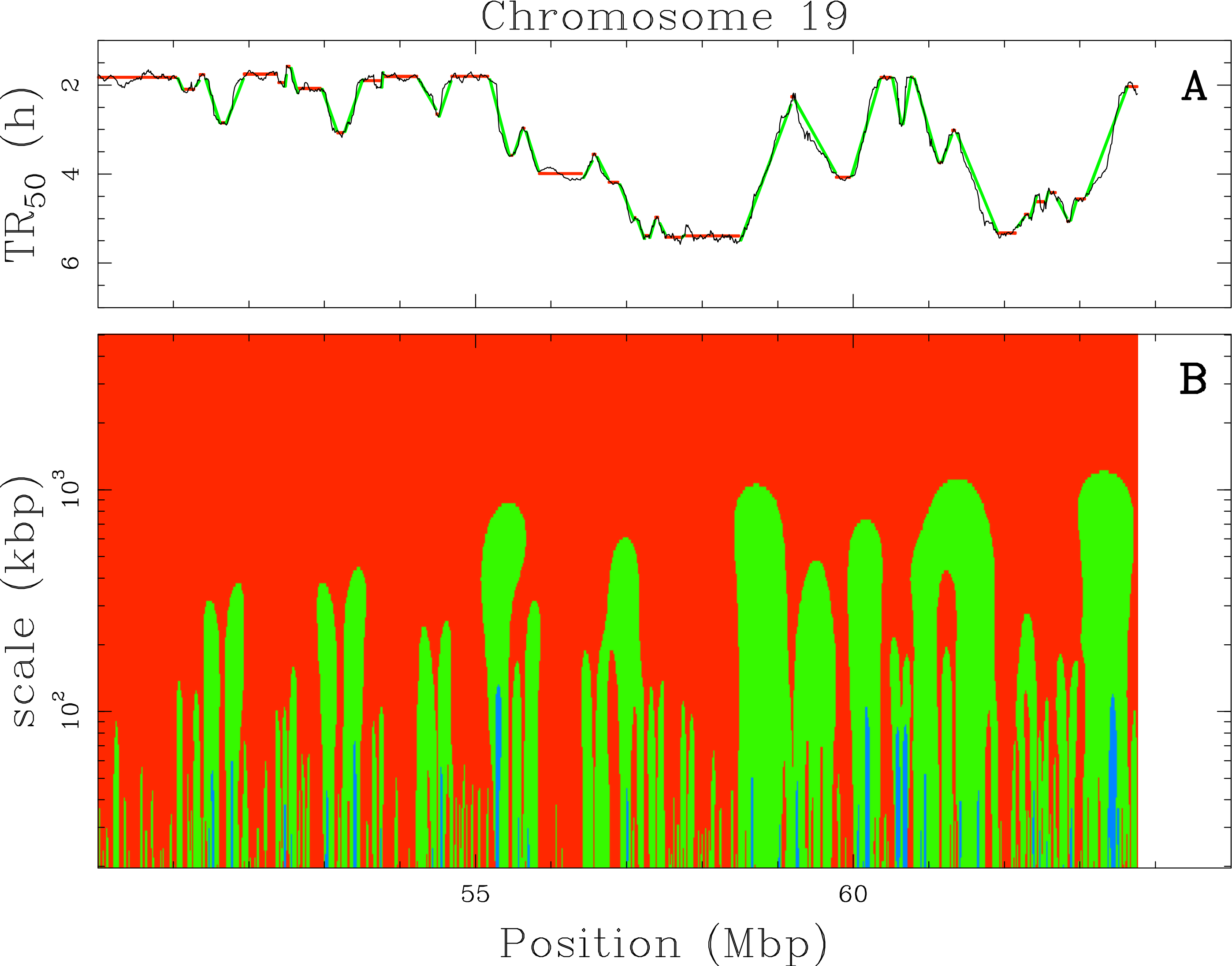
Chromosome 19
A
B
TR$_{50}$ (h)
2
4
6
scale (kbp)
10^3
10^2
55
60
Position (Mbp)

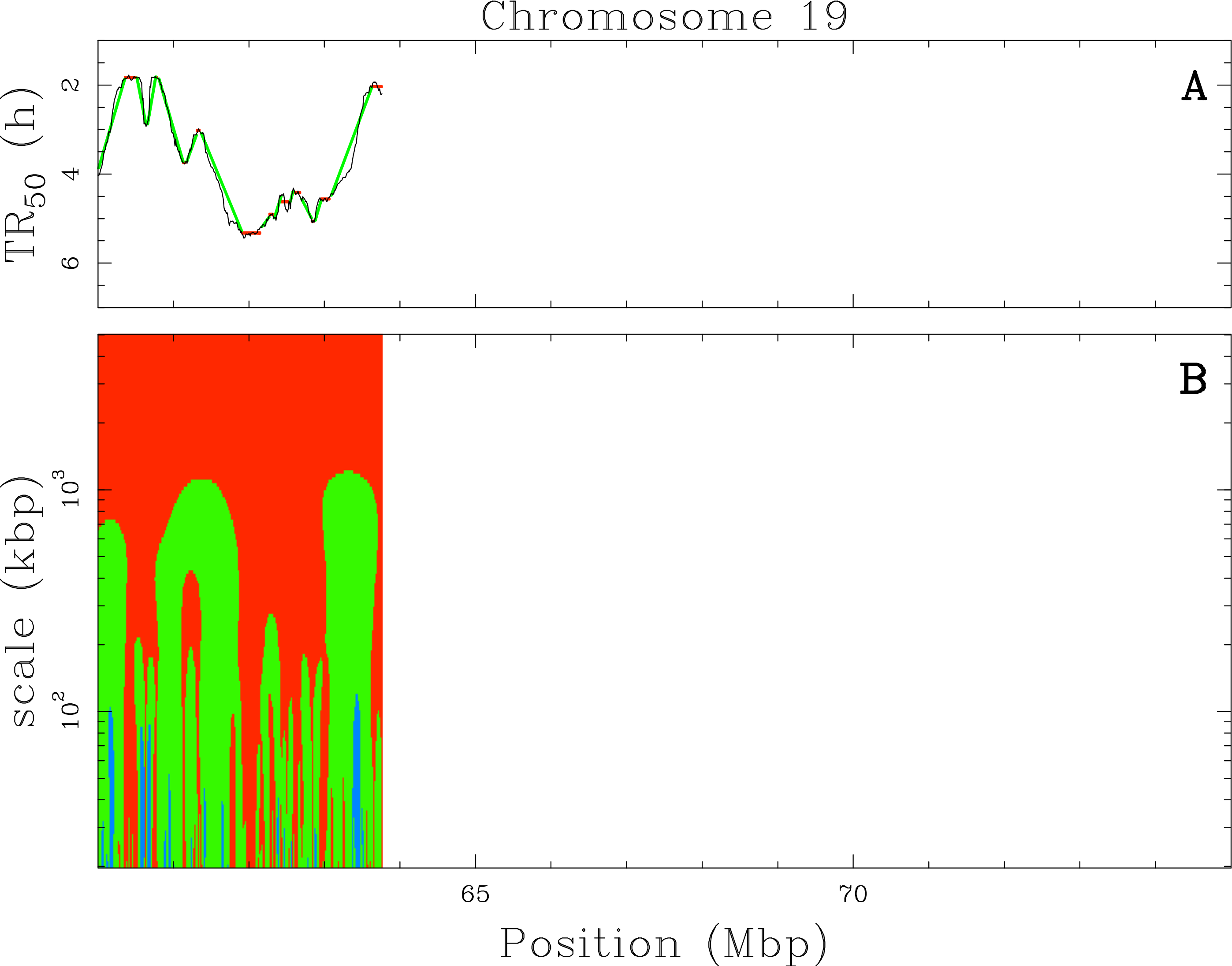

Chromosome 19
A
B
TR50 (h)
2
4
6
scale (kbp)
10^3
10^2
65
70
Position (Mbp)

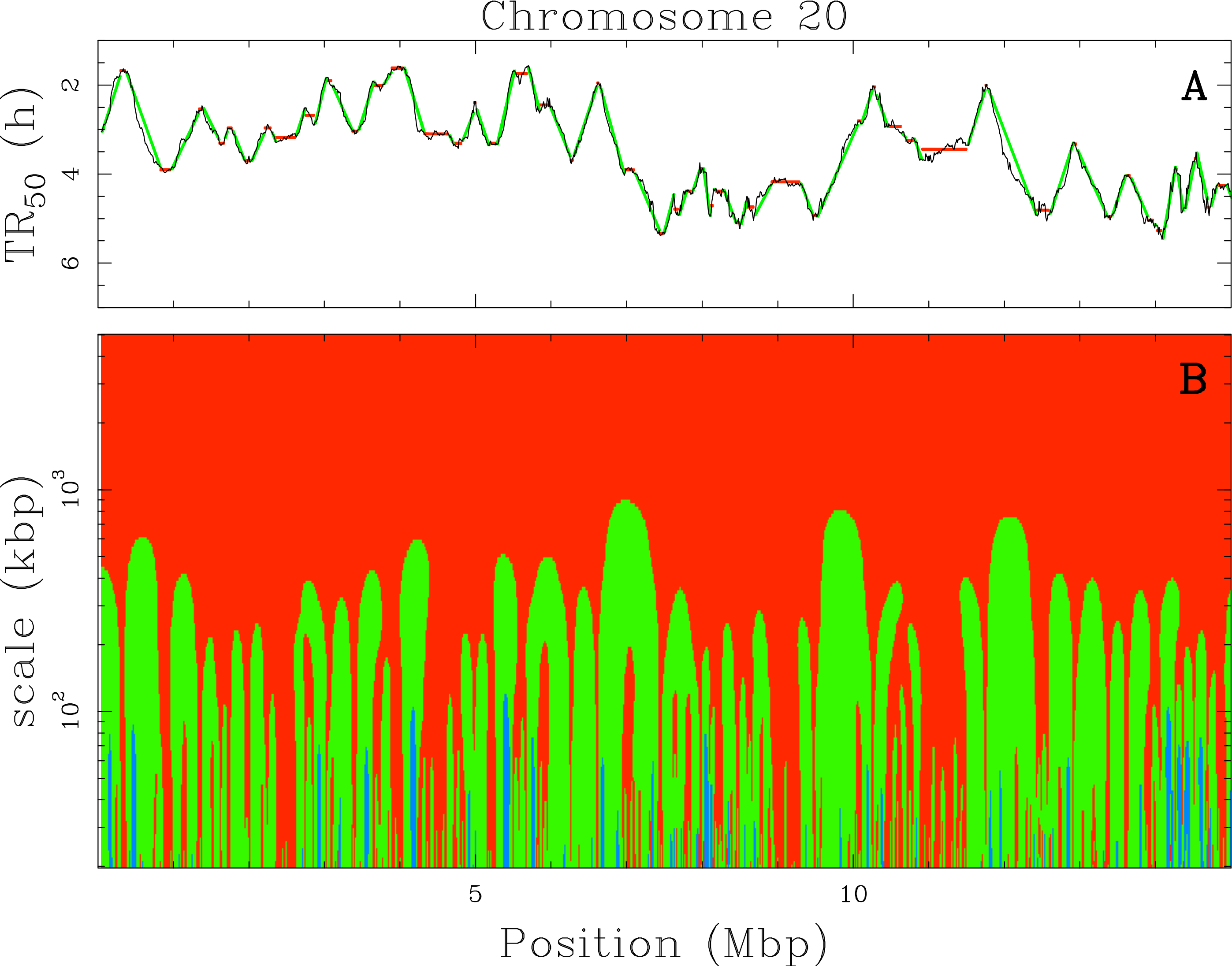

Chromosome 20
A
TR50 (h)
2
4
6
B
scale (kbp)
10^3
10^2
5
10
Position (Mbp)

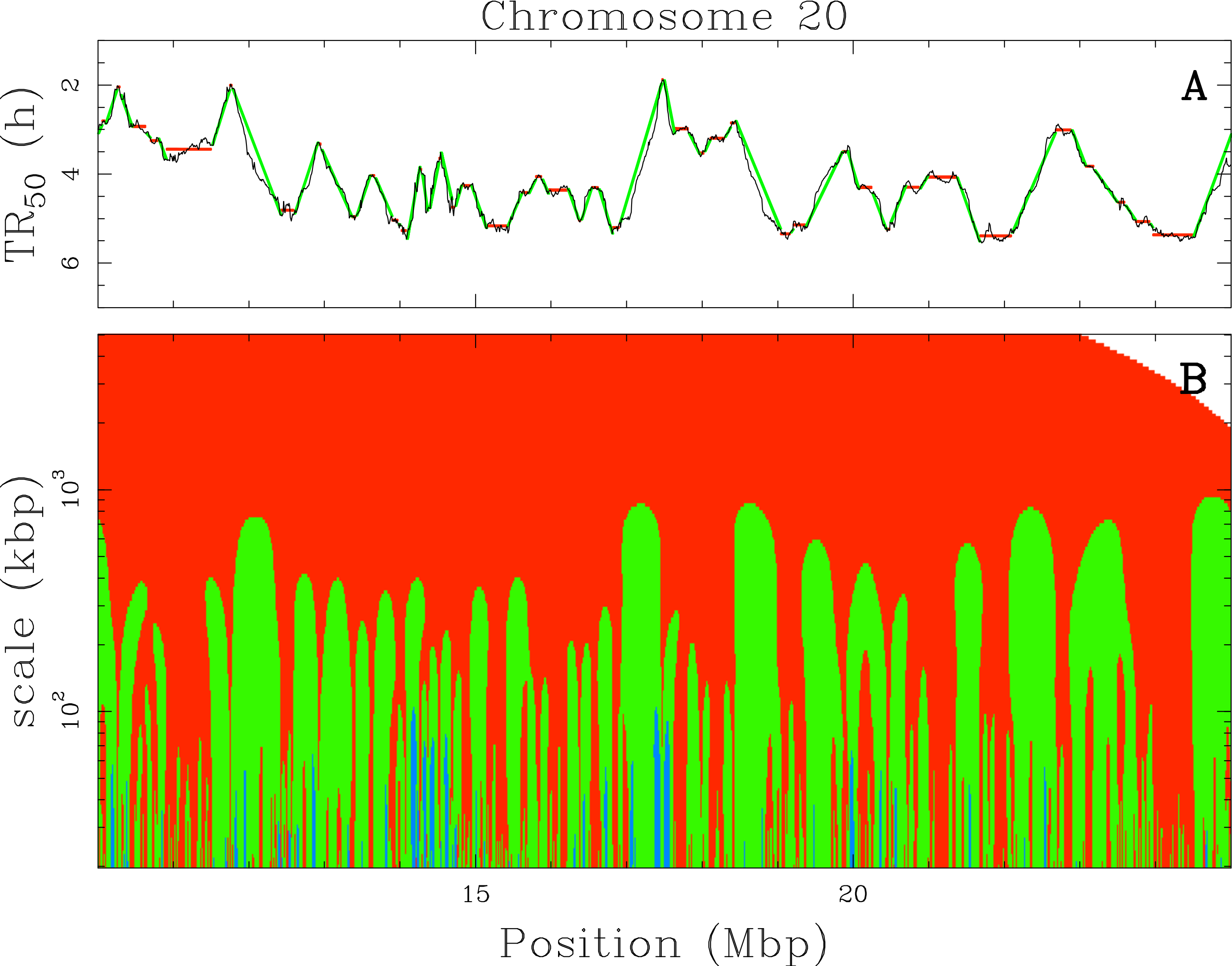

Chromosome 20
A
B
TR50 (h)
2
4
6
scale (kbp)
10^3
10^2
15
20
Position (Mbp)

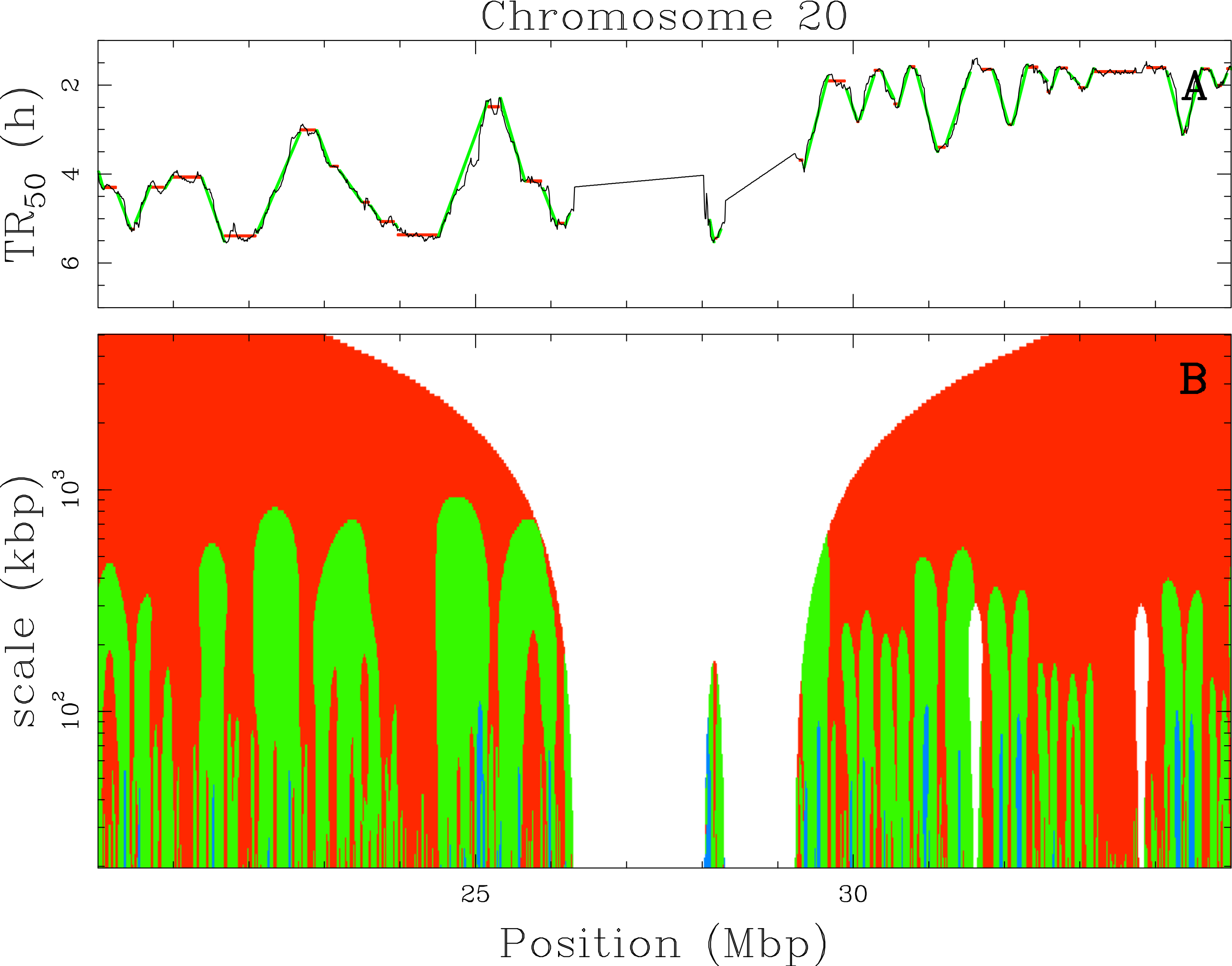

Chromosome 20
TR_{50} (h)
2
4
6
A
B
scale (kbp)
10^3
10^2
25
30
Position (Mbp)

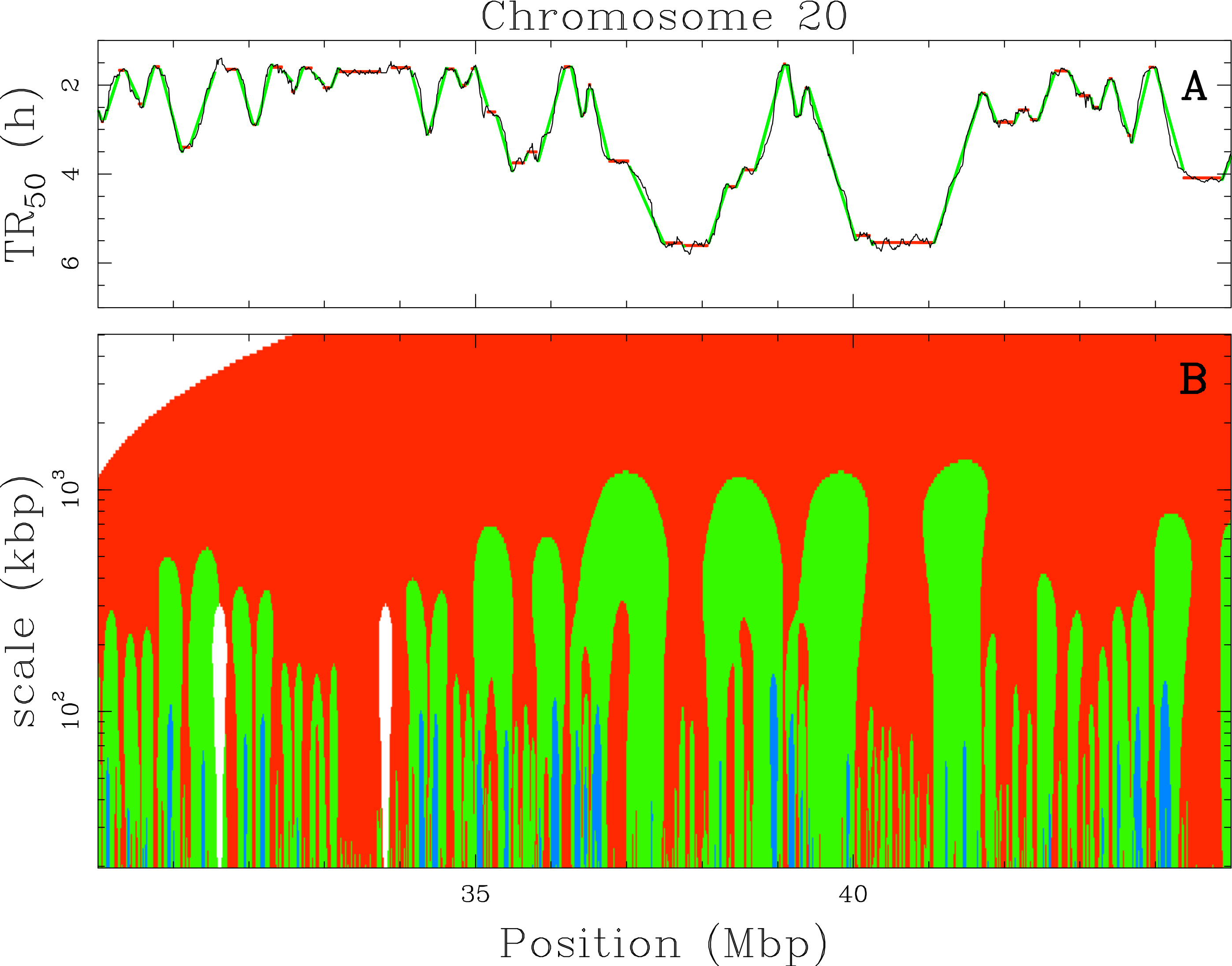

Chromosome 20
TR50 (h)
2
4
6
A
B
scale (kbp)
10^3
10^2
35
40
Position (Mbp)

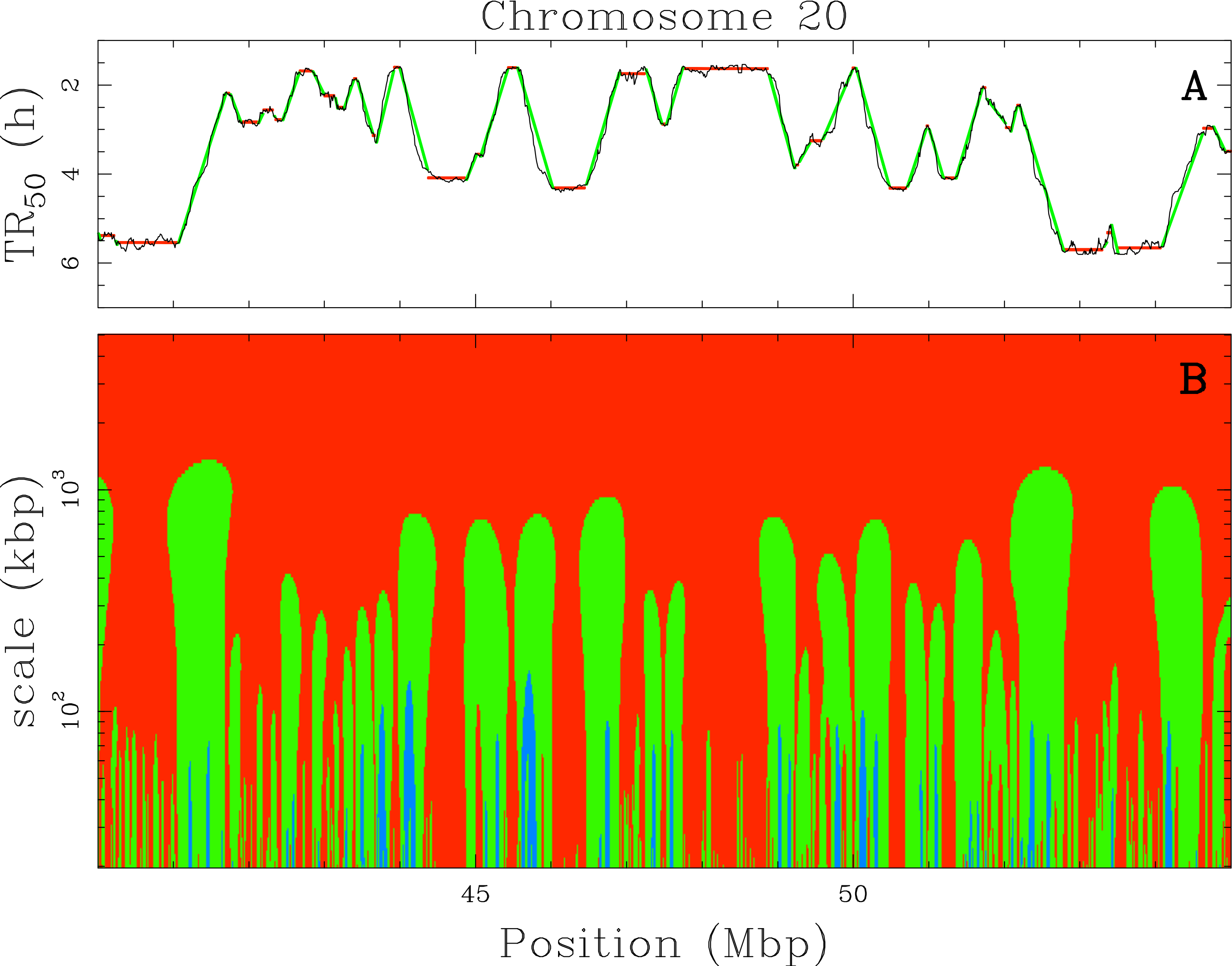

Chromosome 20
TR50 (h)
2
4
6
A
B
scale (kbp)
10^3
10^2
45
50
Position (Mbp)

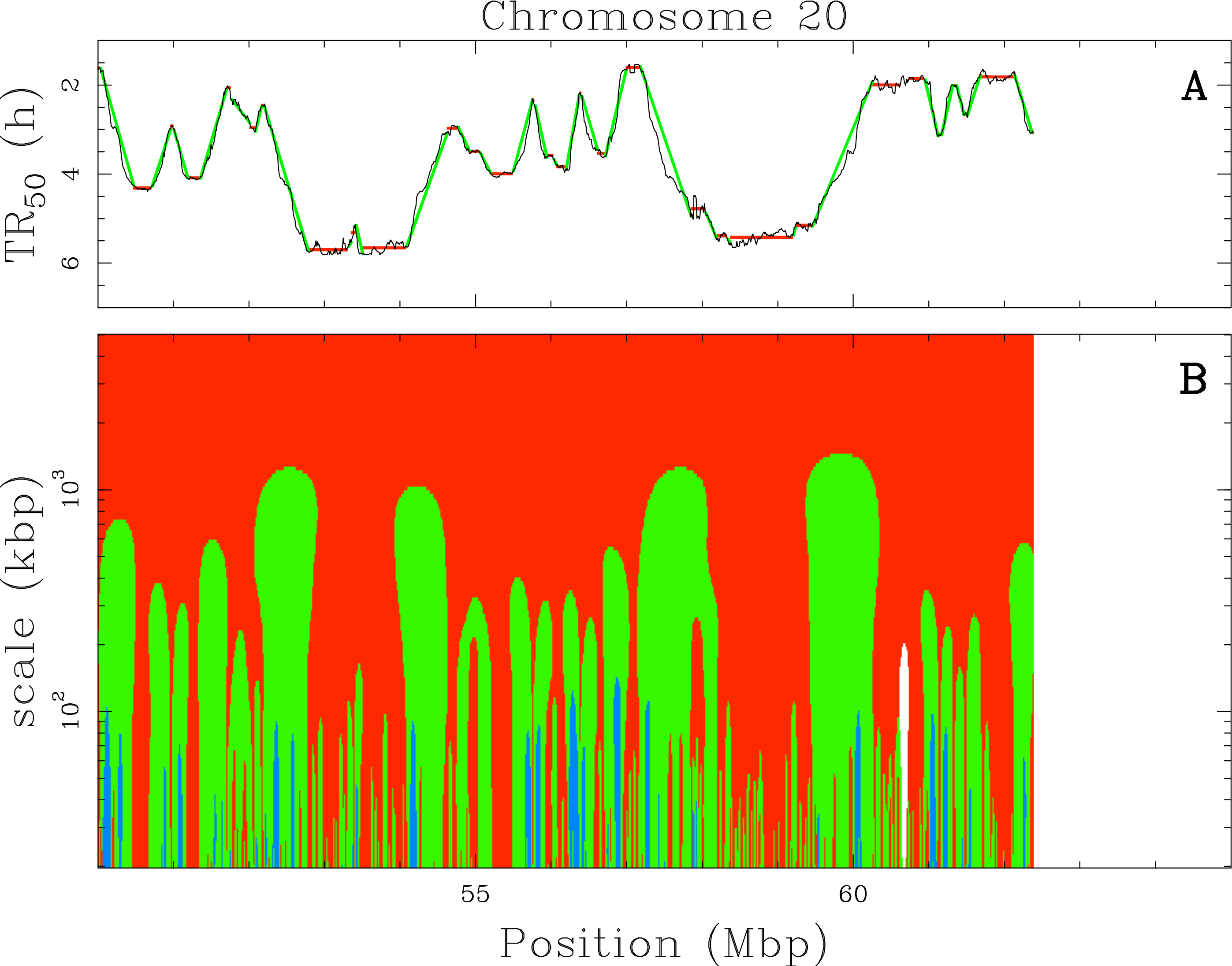

Chromosome 20
A
B
TR50 (h)
2
4
6
scale (kbp)
10^3
10^2
55
60
Position (Mbp)

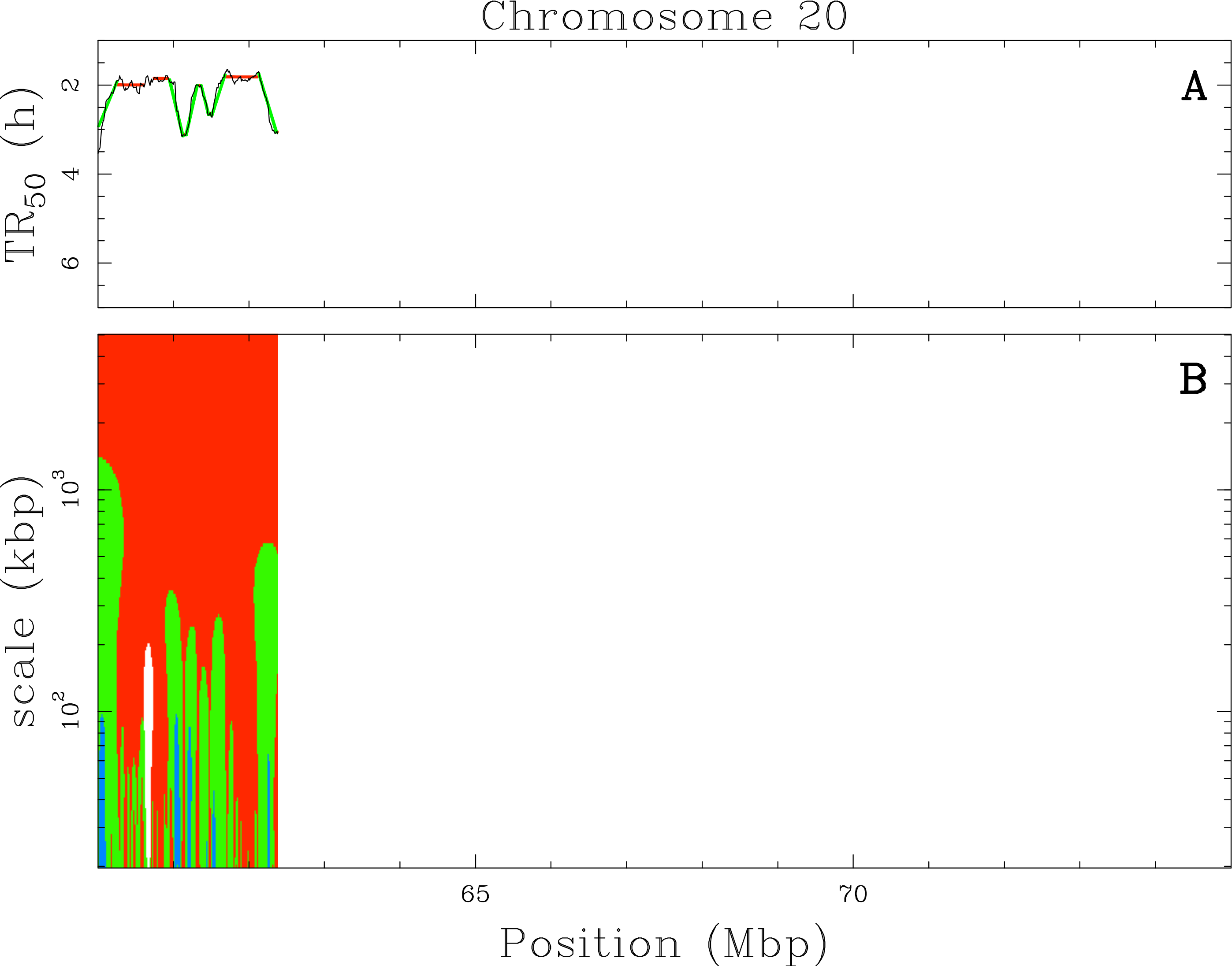

Chromosome 20
TR50 (h)
2
4
6
A
B
scale (kbp)
10^3
10^2
65
70
Position (Mbp)

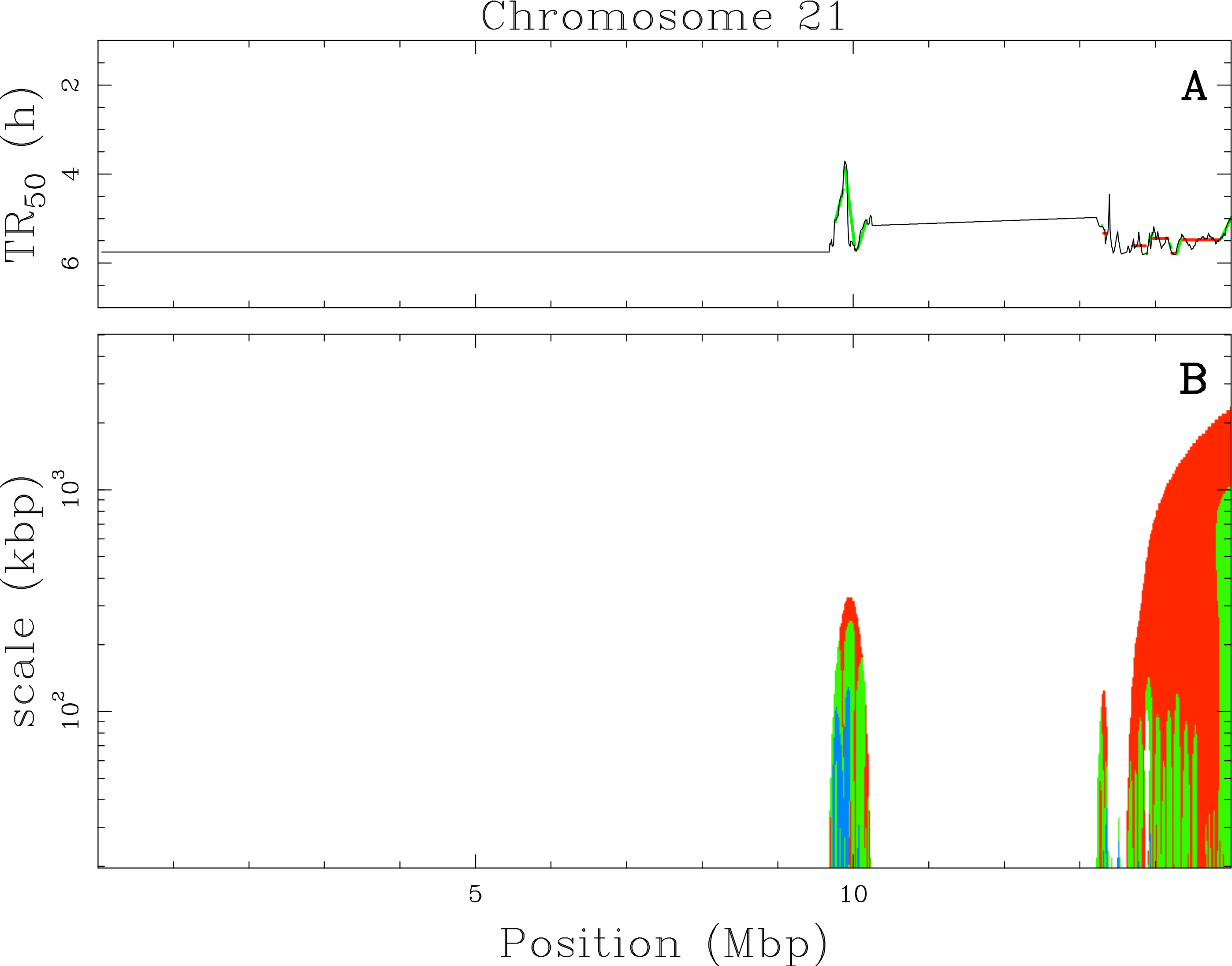

Chromosome 21
A
B
TR50 (h)
2
4
6
scale (kbp)
10^3
10^2
5
10
Position (Mbp)

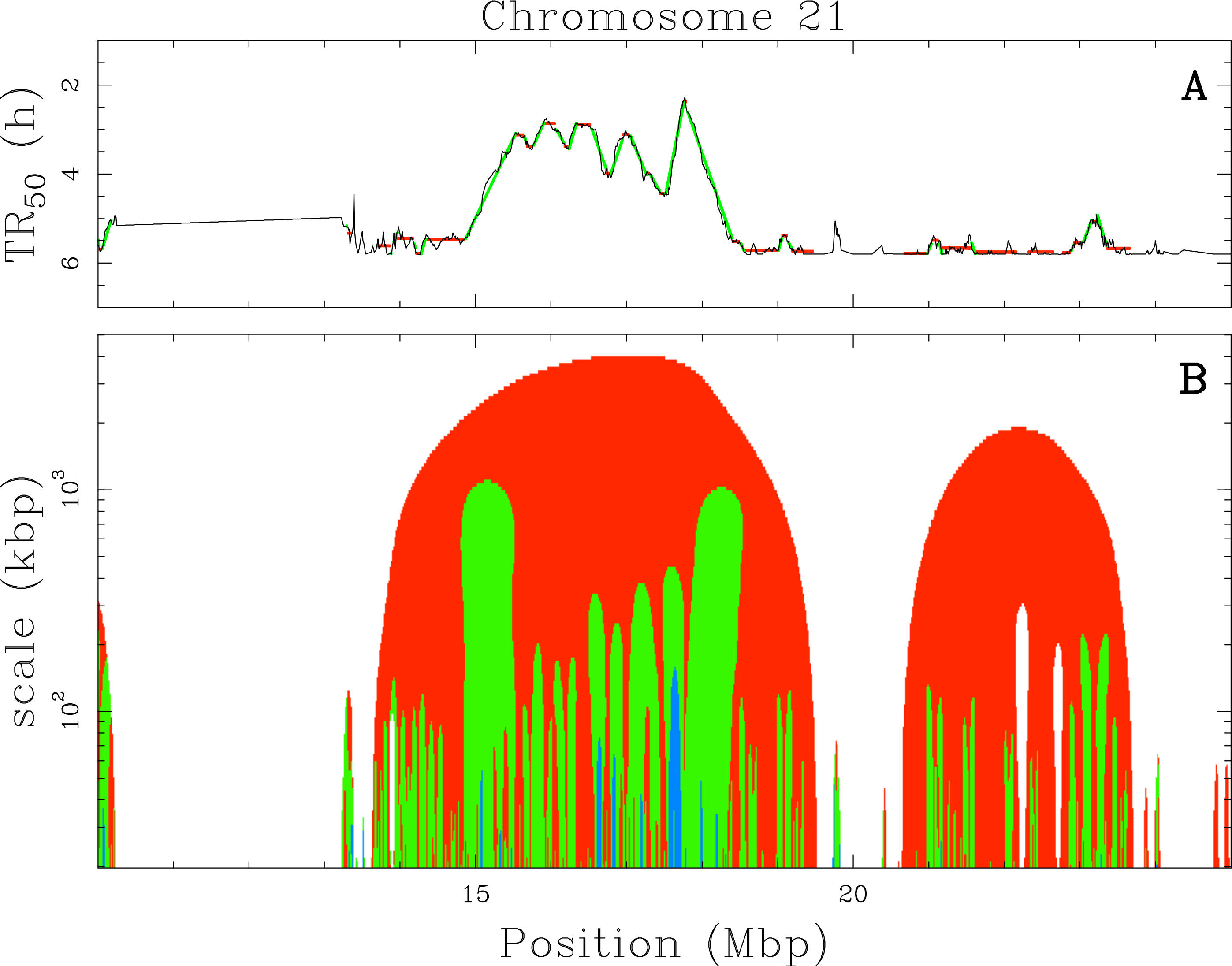

Chromosome 21
A
B
TR50 (h)
2
4
6
scale (kbp)
10^3
10^2
15
20
Position (Mbp)

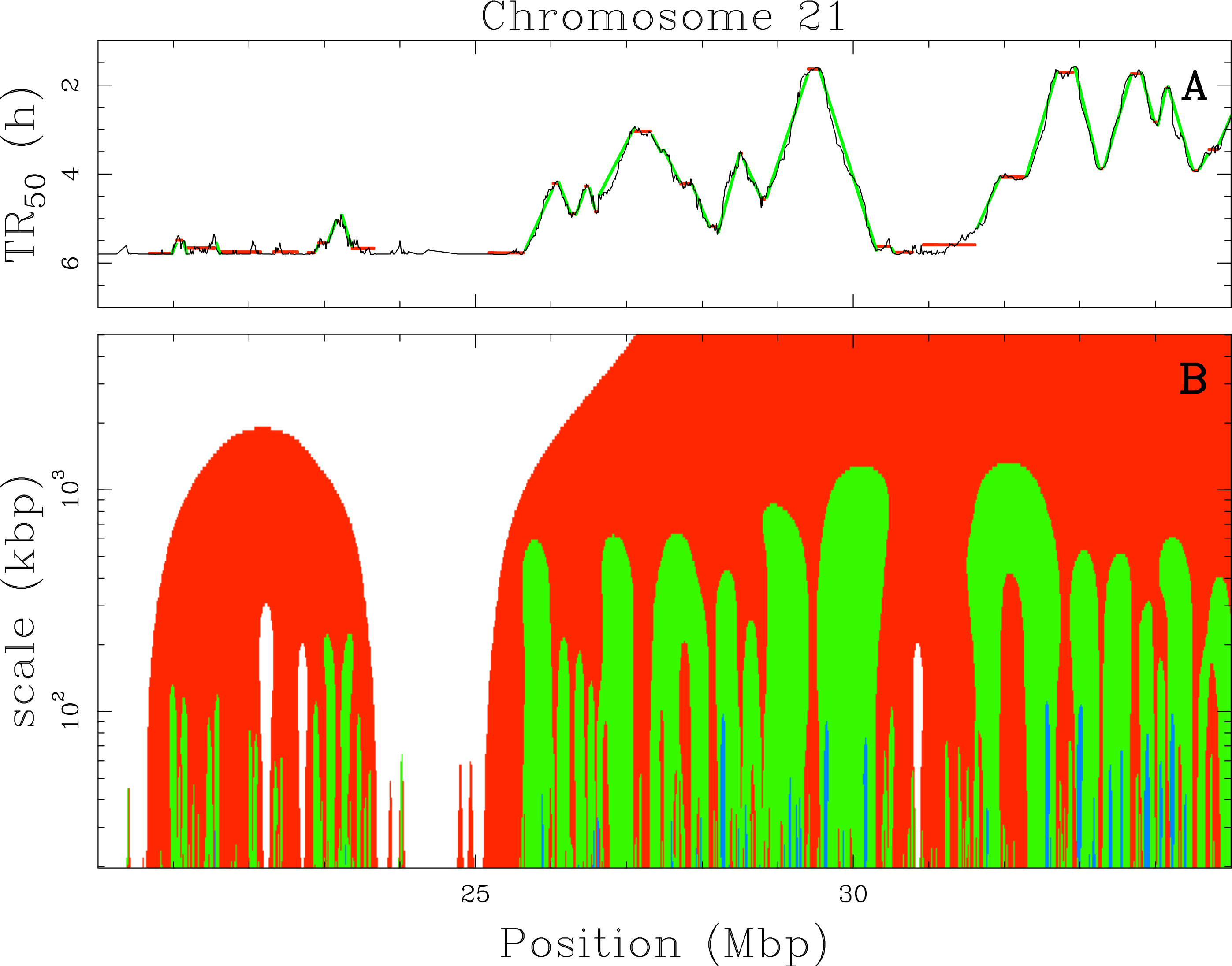
Chromosome 21
A
B
TR_{50} (h)
2
4
6
scale (kbp)
10^3
10^2
25
30
Position (Mbp)

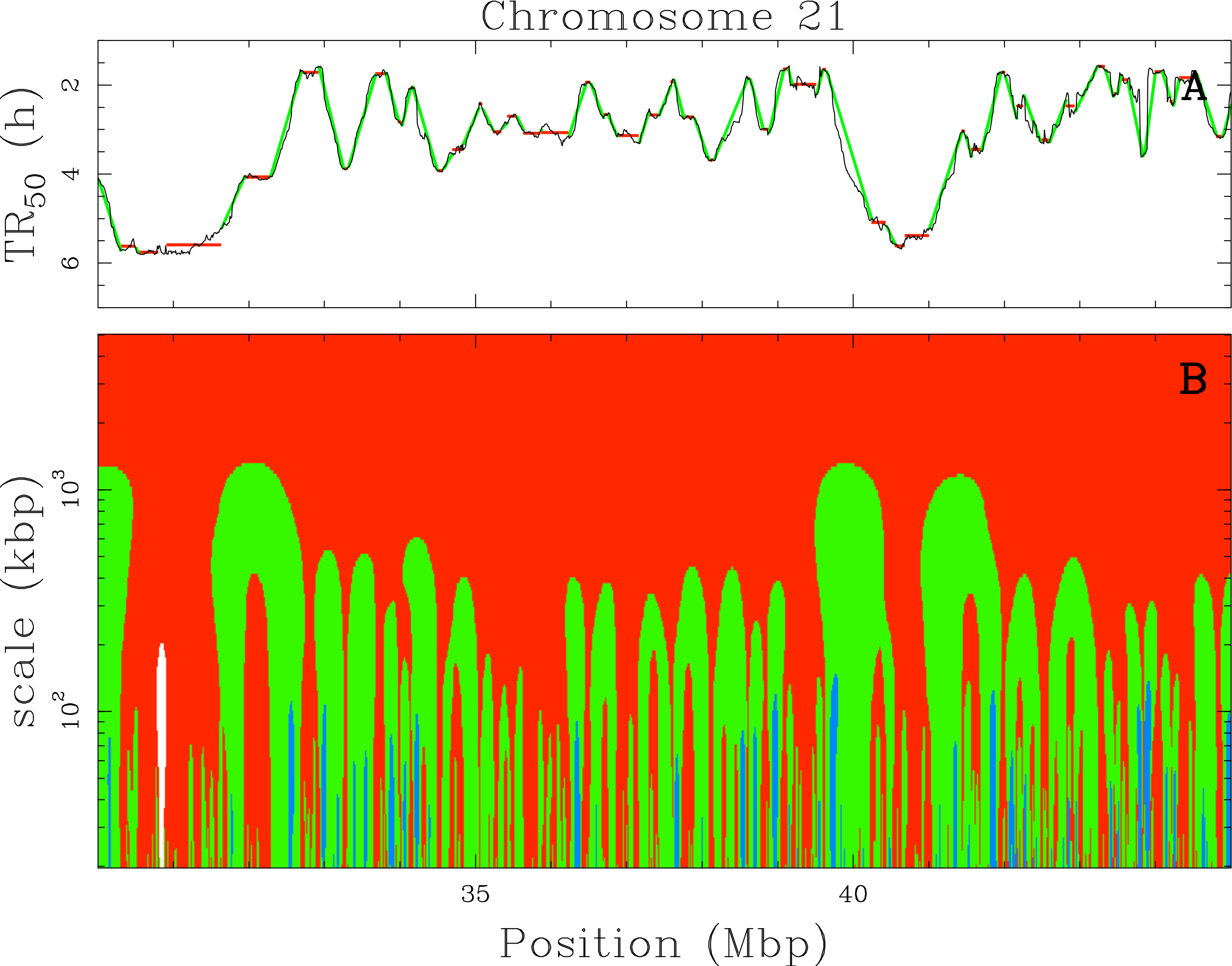

Chromosome 21
A
B
TR50 (h)
2
4
6
scale (kbp)
10^3
10^2
35
40
Position (Mbp)

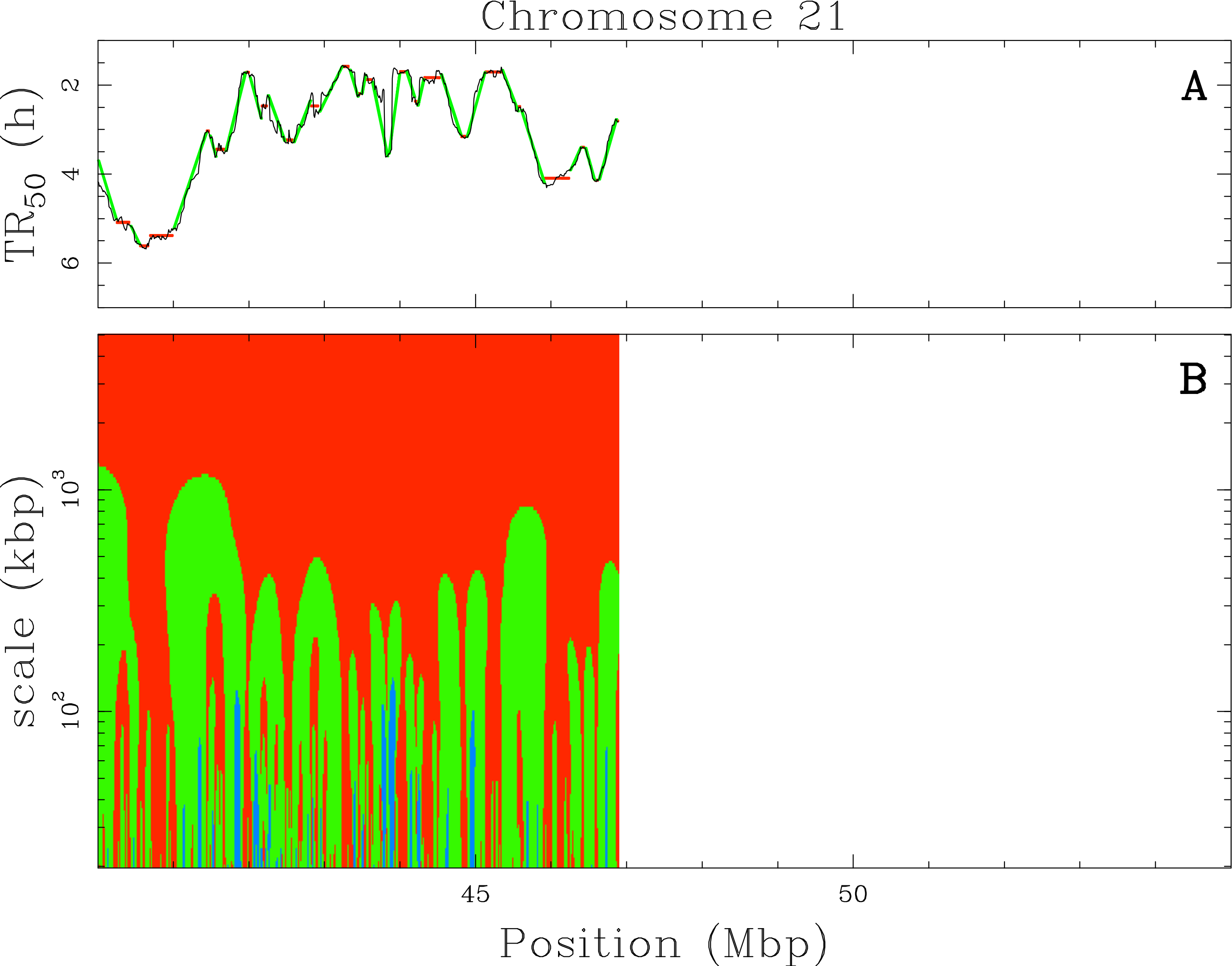

Chromosome 21
A
B
TR50 (h)
2
4
6
scale (kbp)
10^3
10^2
45
50
Position (Mbp)

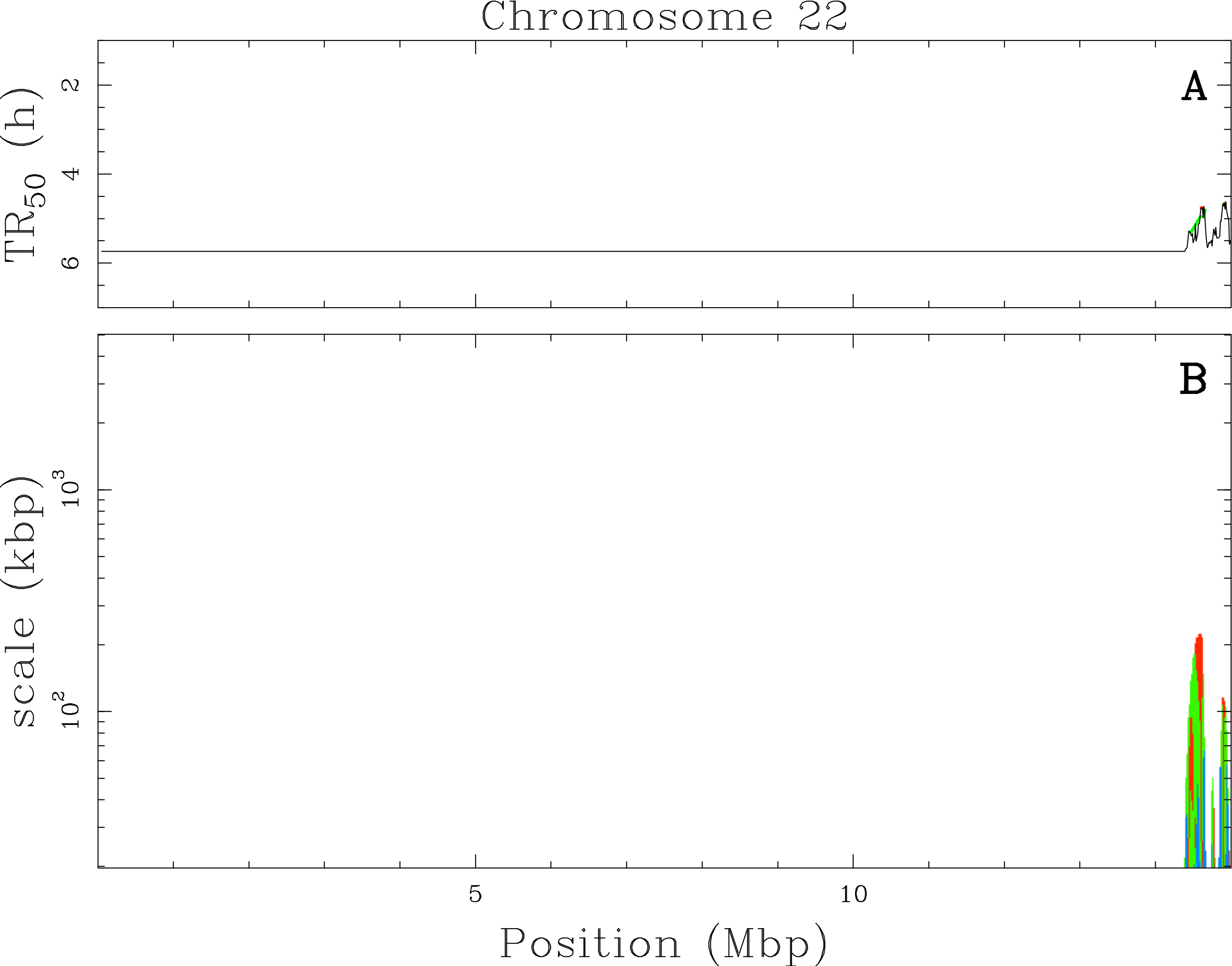

Chromosome 22
A
B
TR50 (h)
2
4
6
scale (kbp)
10^3
10^2
5
10
Position (Mbp)

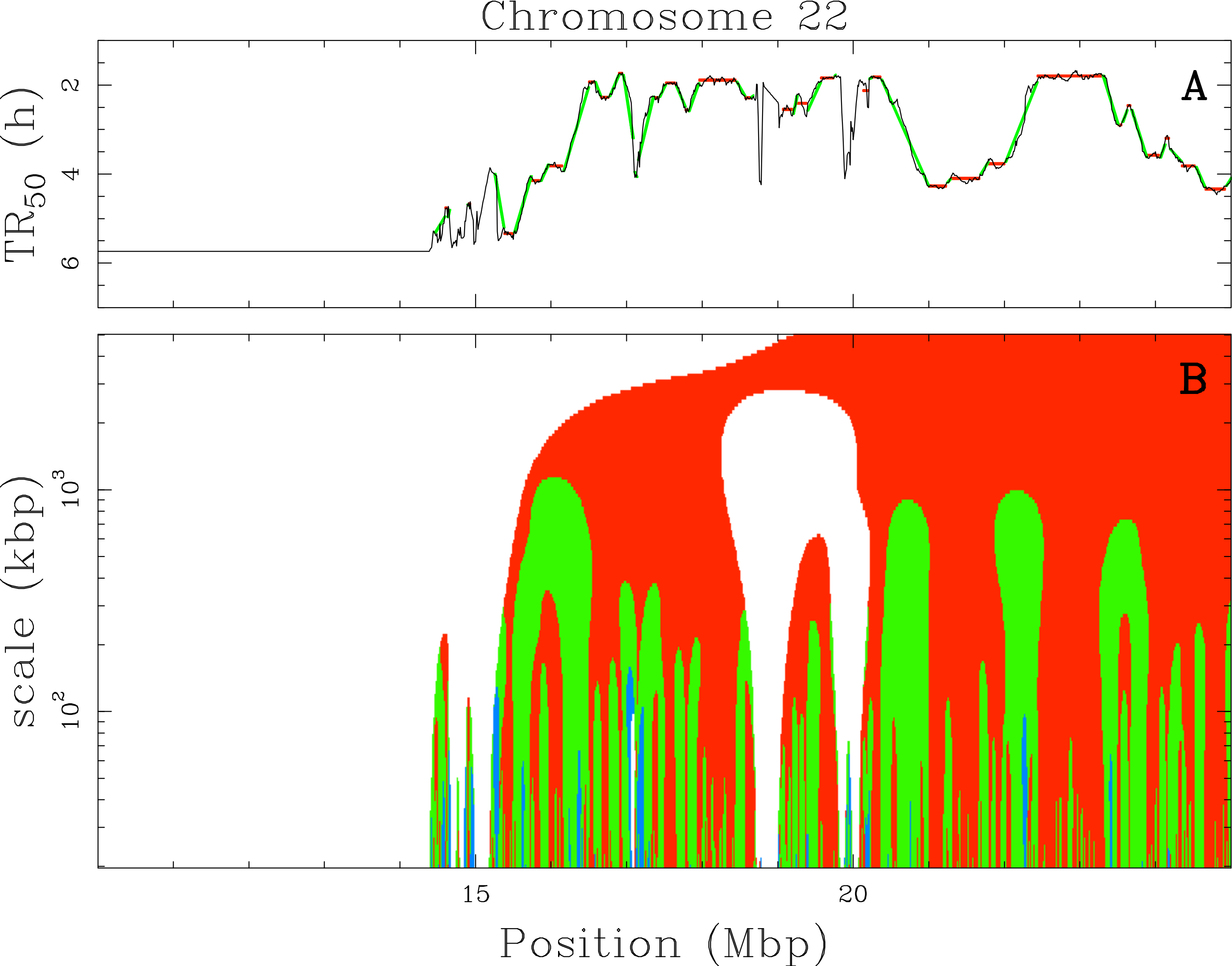

Chromosome 22
A
B
TR_{50} (h)
2
4
6
scale (kbp)
10^3
10^2
15
20
Position (Mbp)

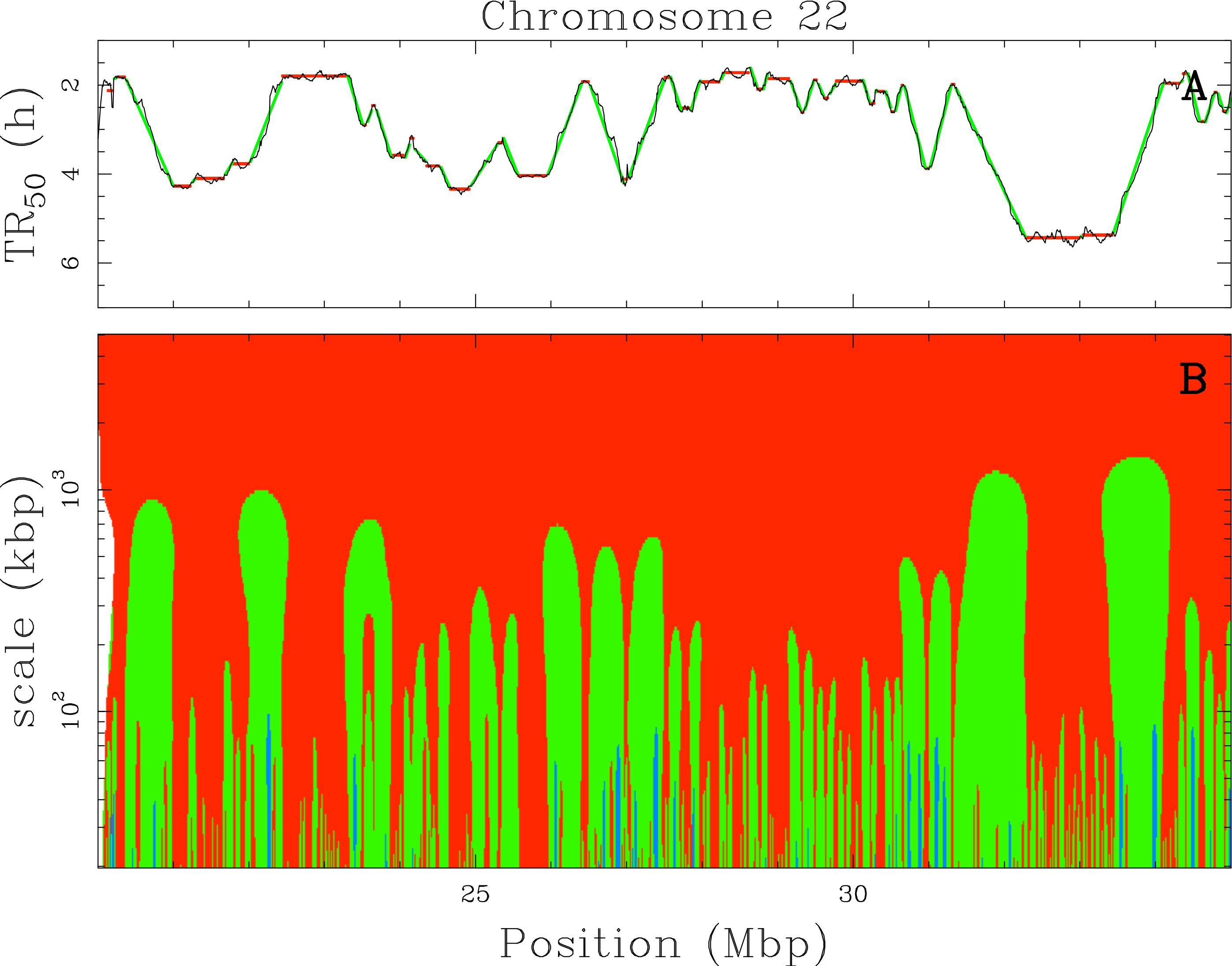

Chromosome 22
A
B
TR_{50} (h)
2
4
6
scale (kbp)
10^3
10^2
25
30
Position (Mbp)

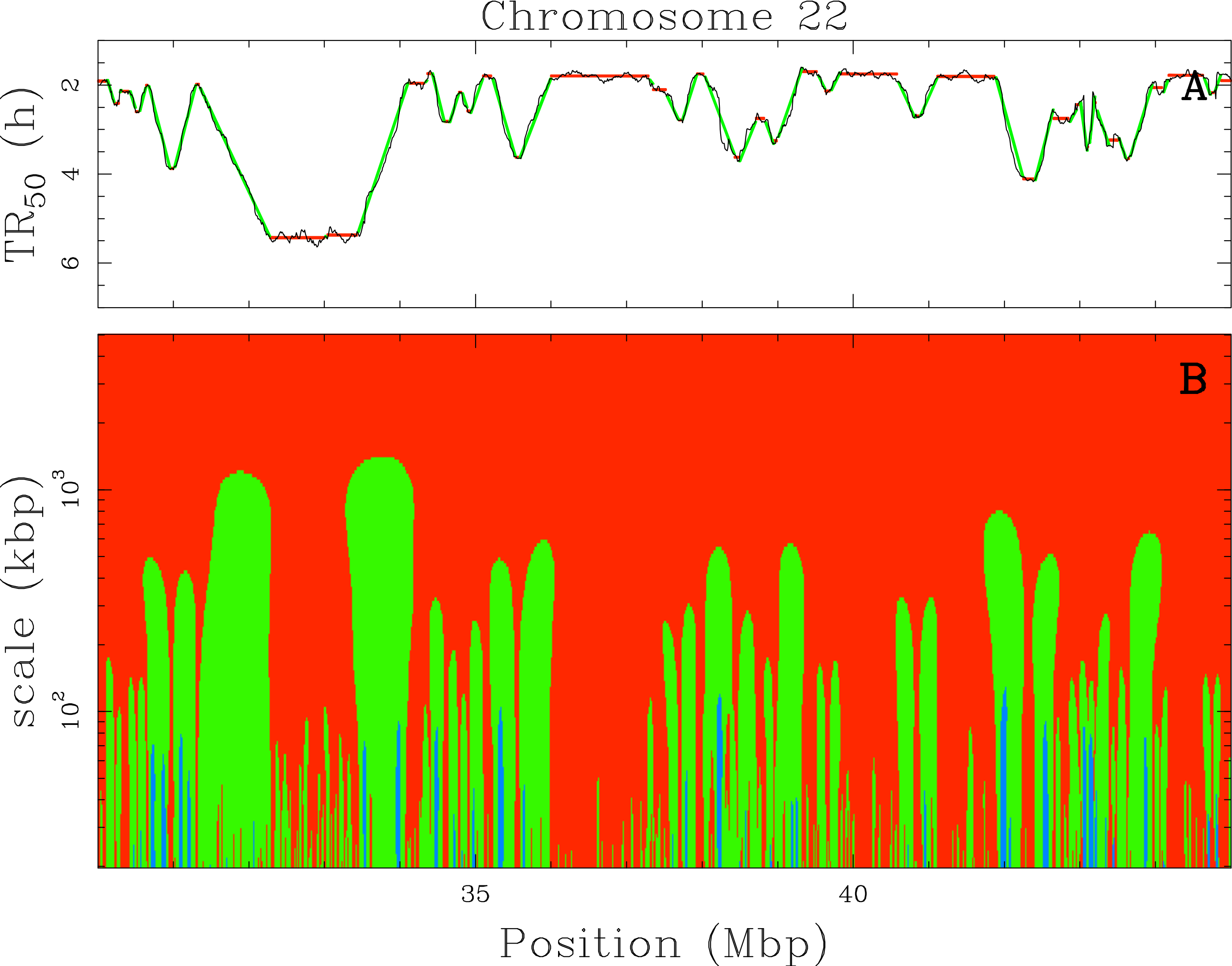

Chromosome 22
TR50 (h)
2
4
6
A
B
scale (kbp)
10^3
10^2
35
40
Position (Mbp)

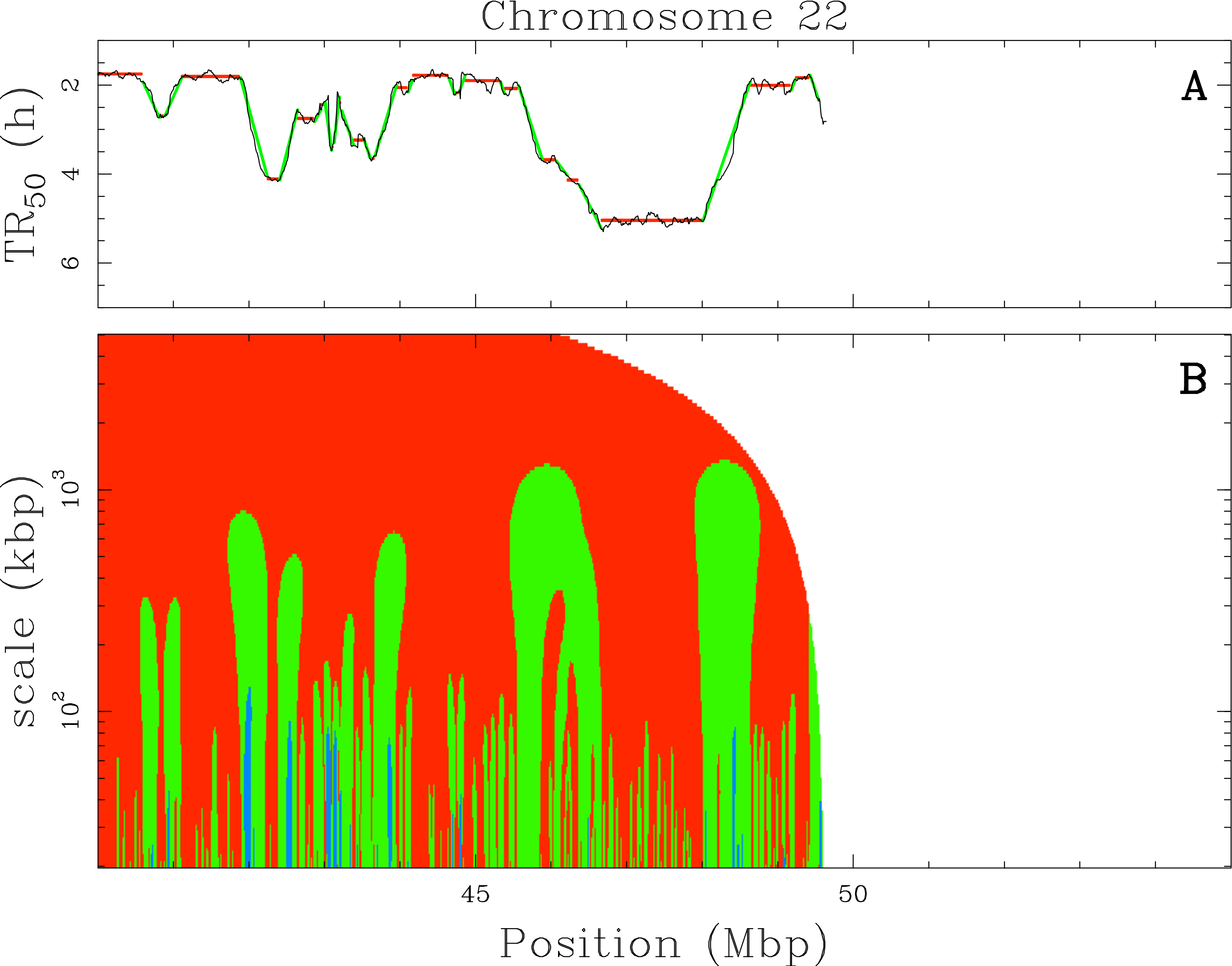

Chromosome 22
TR$_{50}$ (h)
2
4
6
A
B
scale (kbp)
10^3
10^2
45
50
Position (Mbp)

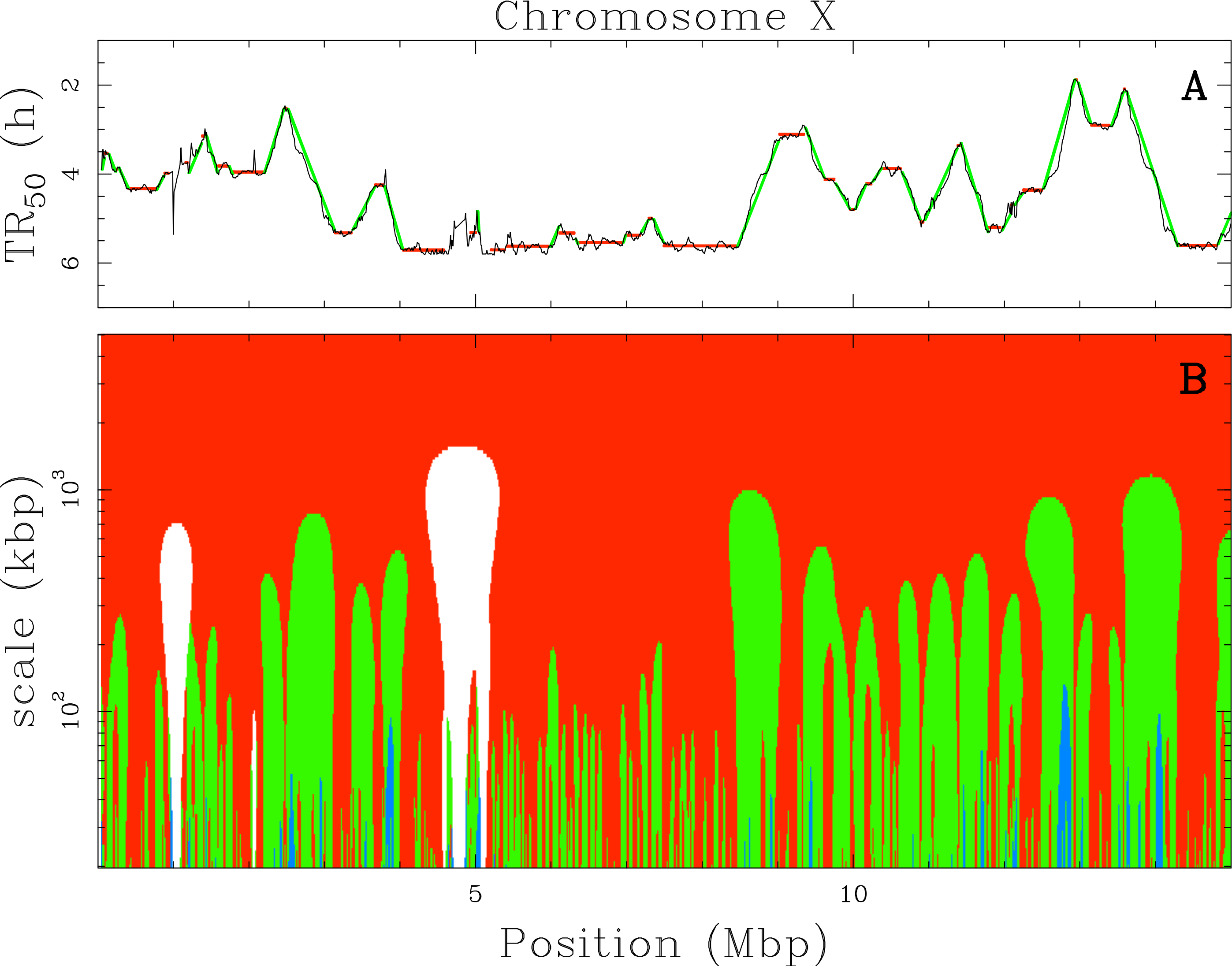

Chromosome X
A
B
TR50 (h)
2
4
6
scale (kbp)
10^3
10^2
5
10
Position (Mbp)

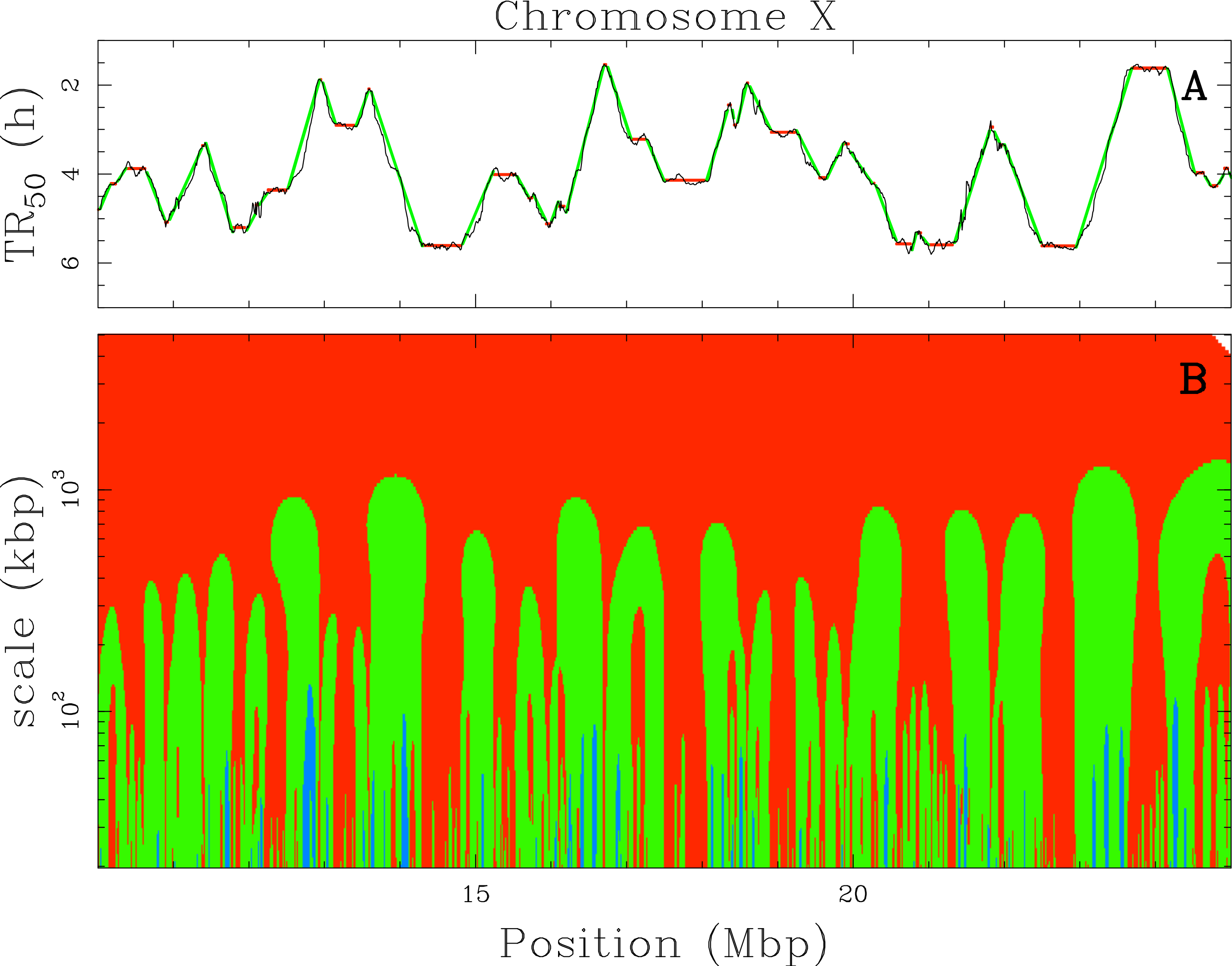

Chromosome X
A
TR50 (h)
2
4
6
B
scale (kbp)
10^3
10^2
15
20
Position (Mbp)

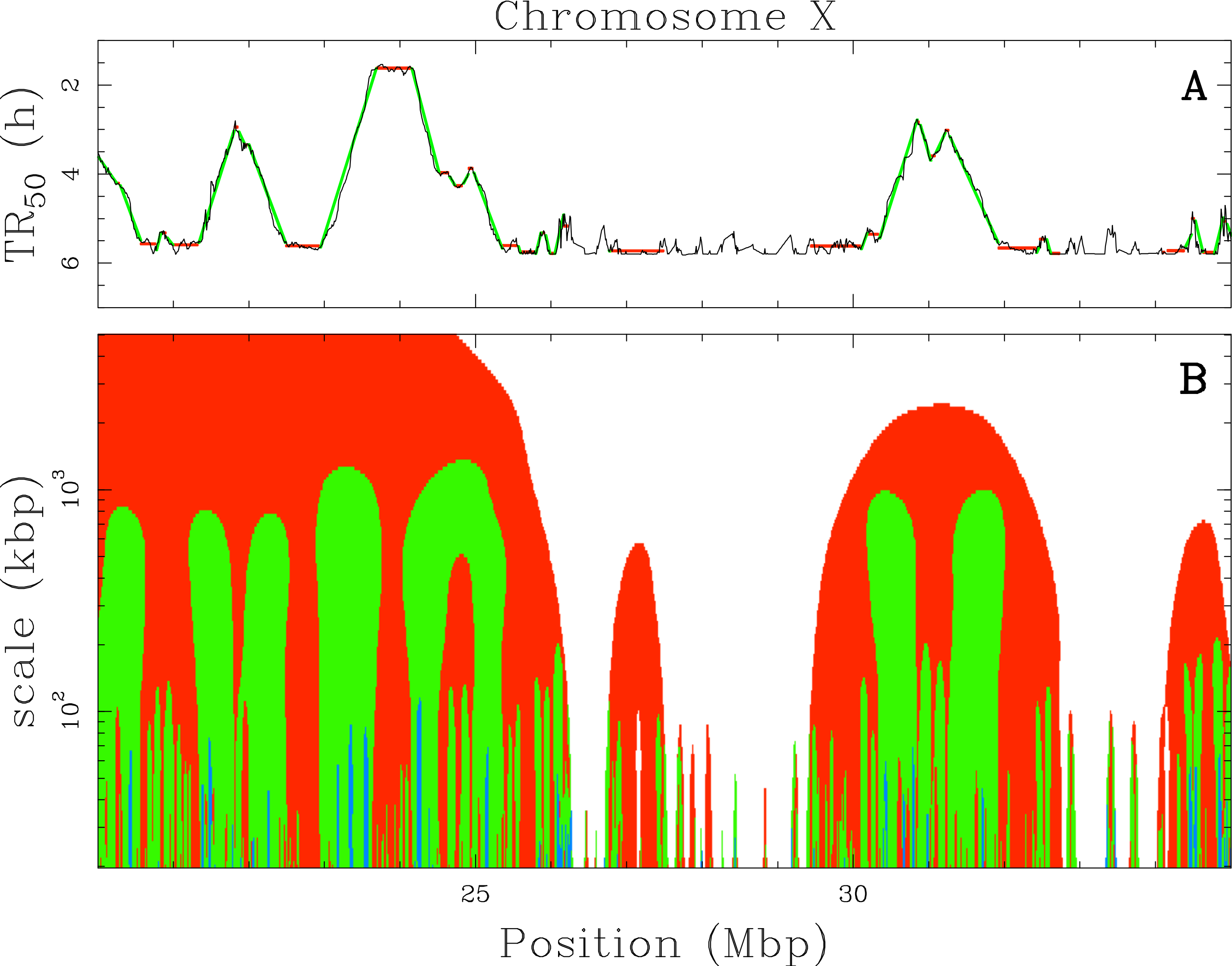
Chromosome X
A
B
TR50 (h)
2
4
6
scale (kbp)
10^3
10^2
25
30
Position (Mbp)

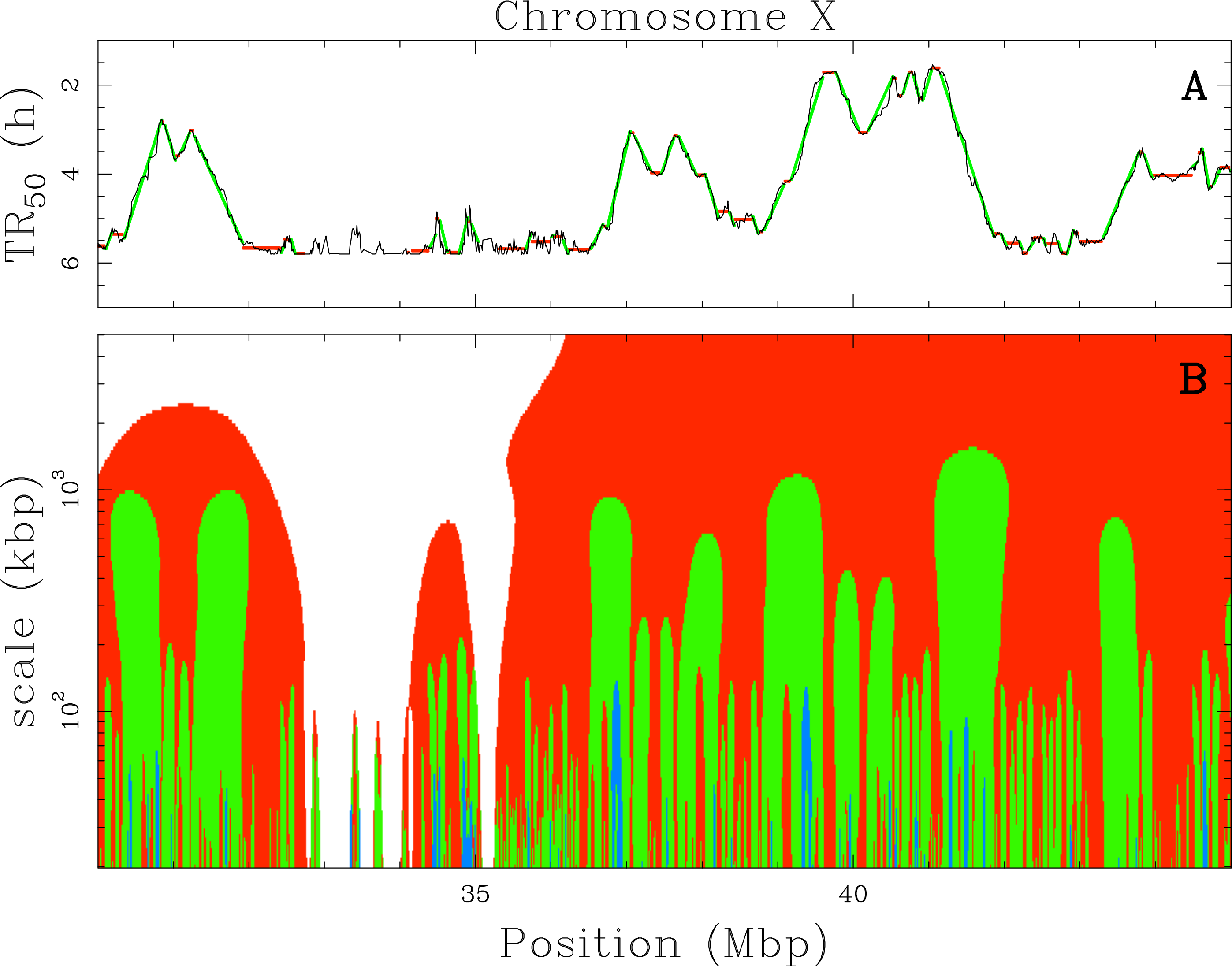
Chromosome X
TR50 (h)
2
4
6
A
B
scale (kbp)
10^3
10^2
35
40
Position (Mbp)

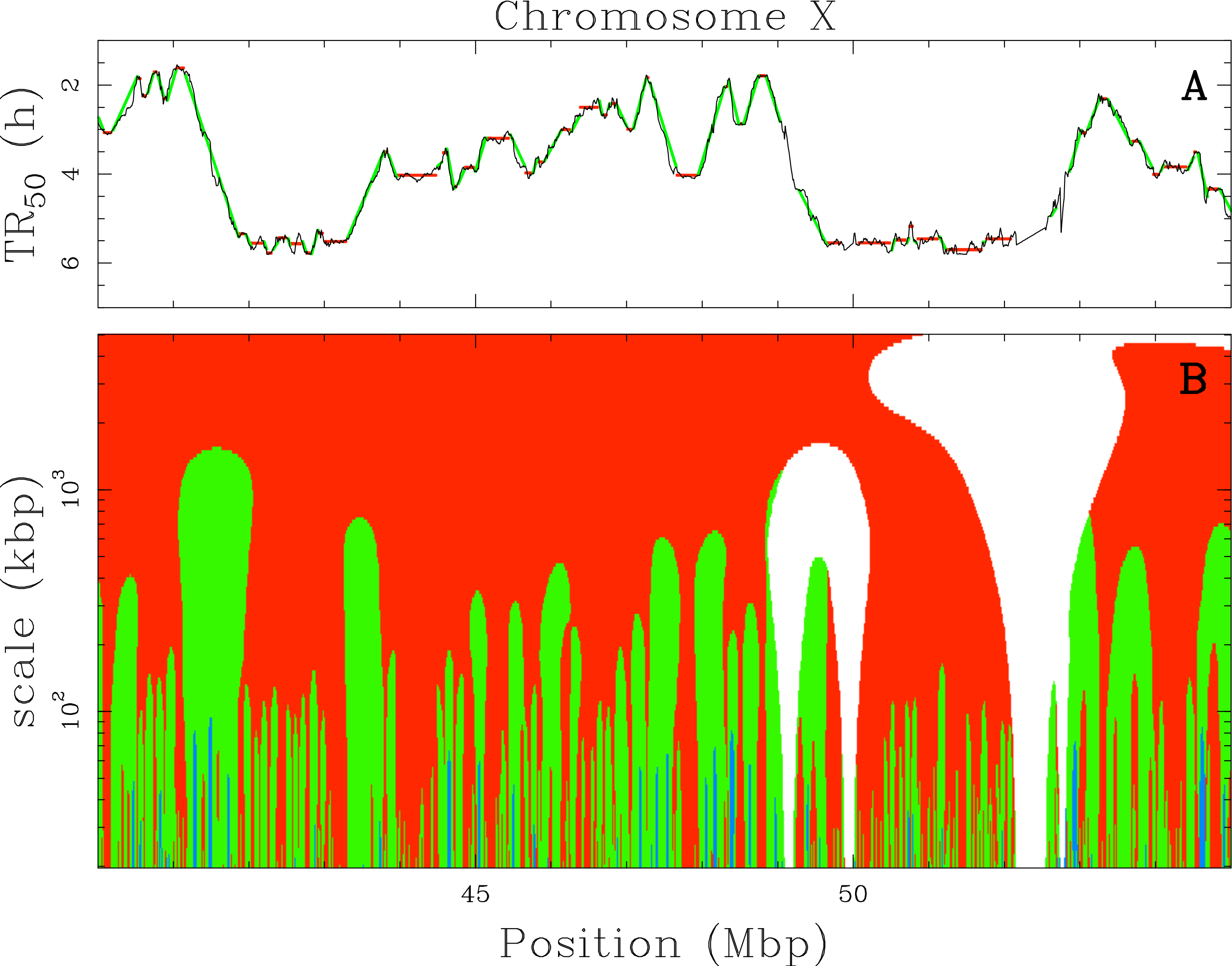

Chromosome X
A
B
TR50 (h)
2
4
6
scale (kbp)
10^3
10^2
45
50
Position (Mbp)

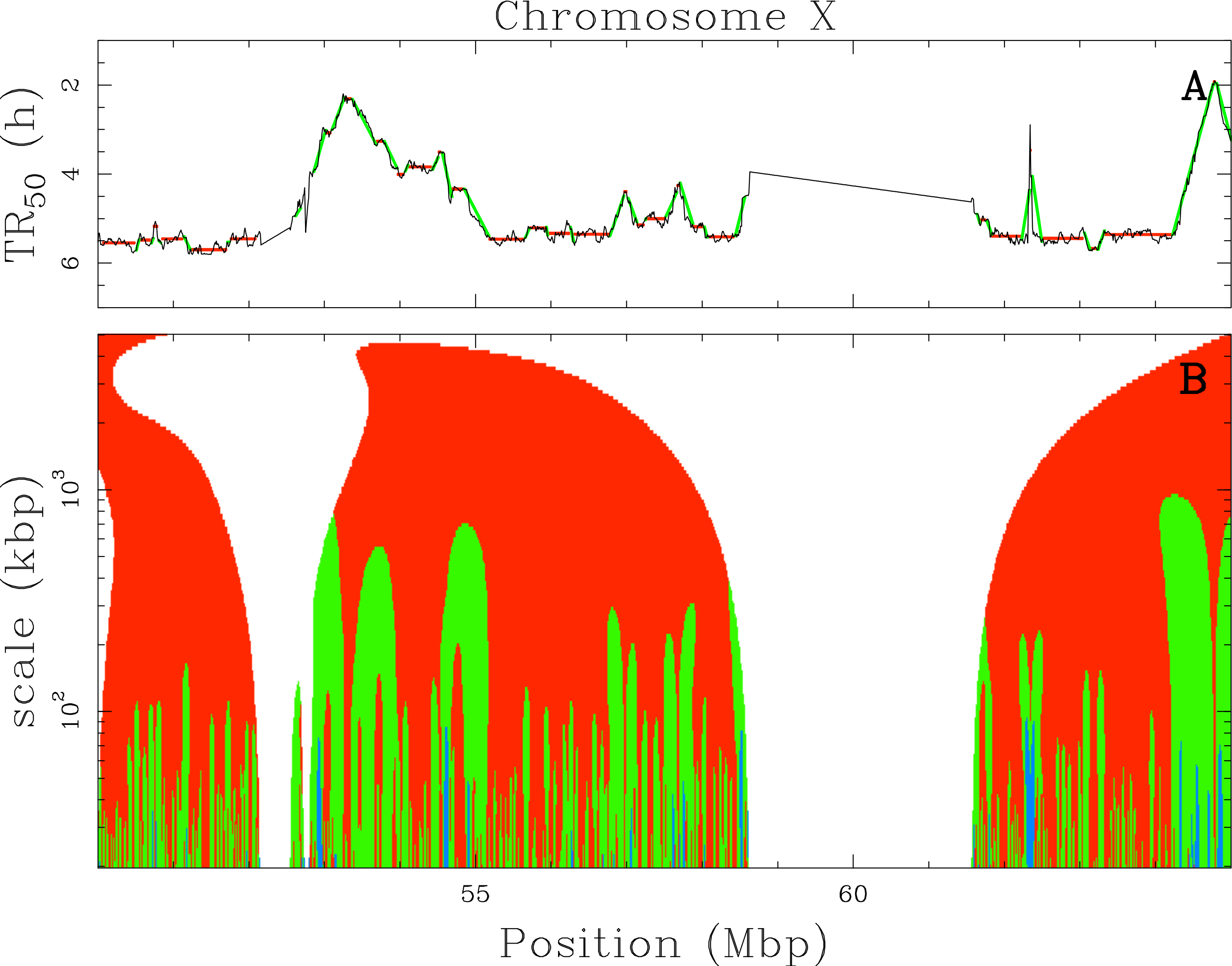

Chromosome X
A
TR50 (h)
2
4
6
B
scale (kbp)
10^3
10^2
55
60
Position (Mbp)

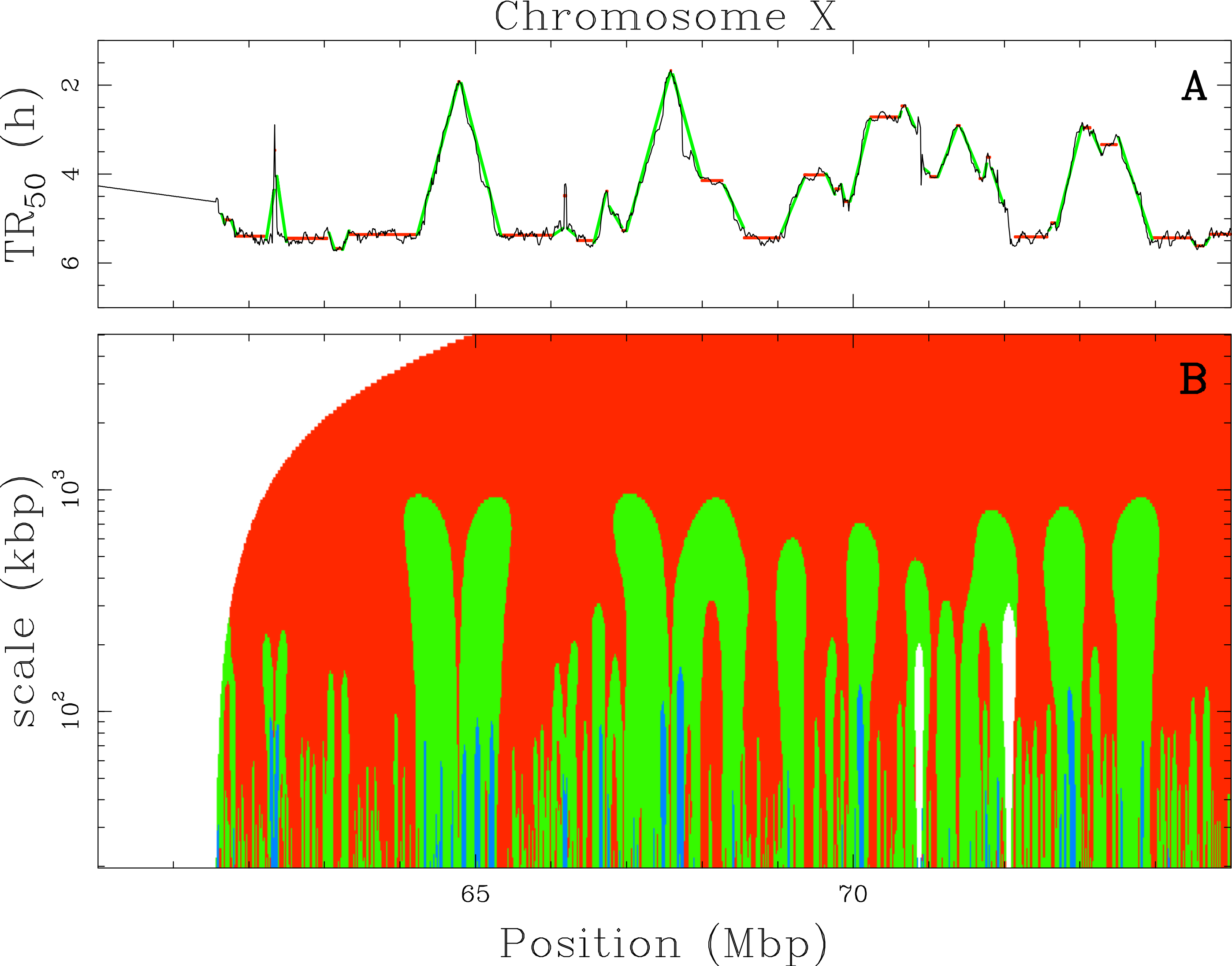

Chromosome X
A
TR50 (h)
2
4
6
B
scale (kbp)
10^3
10^2
65
70
Position (Mbp)

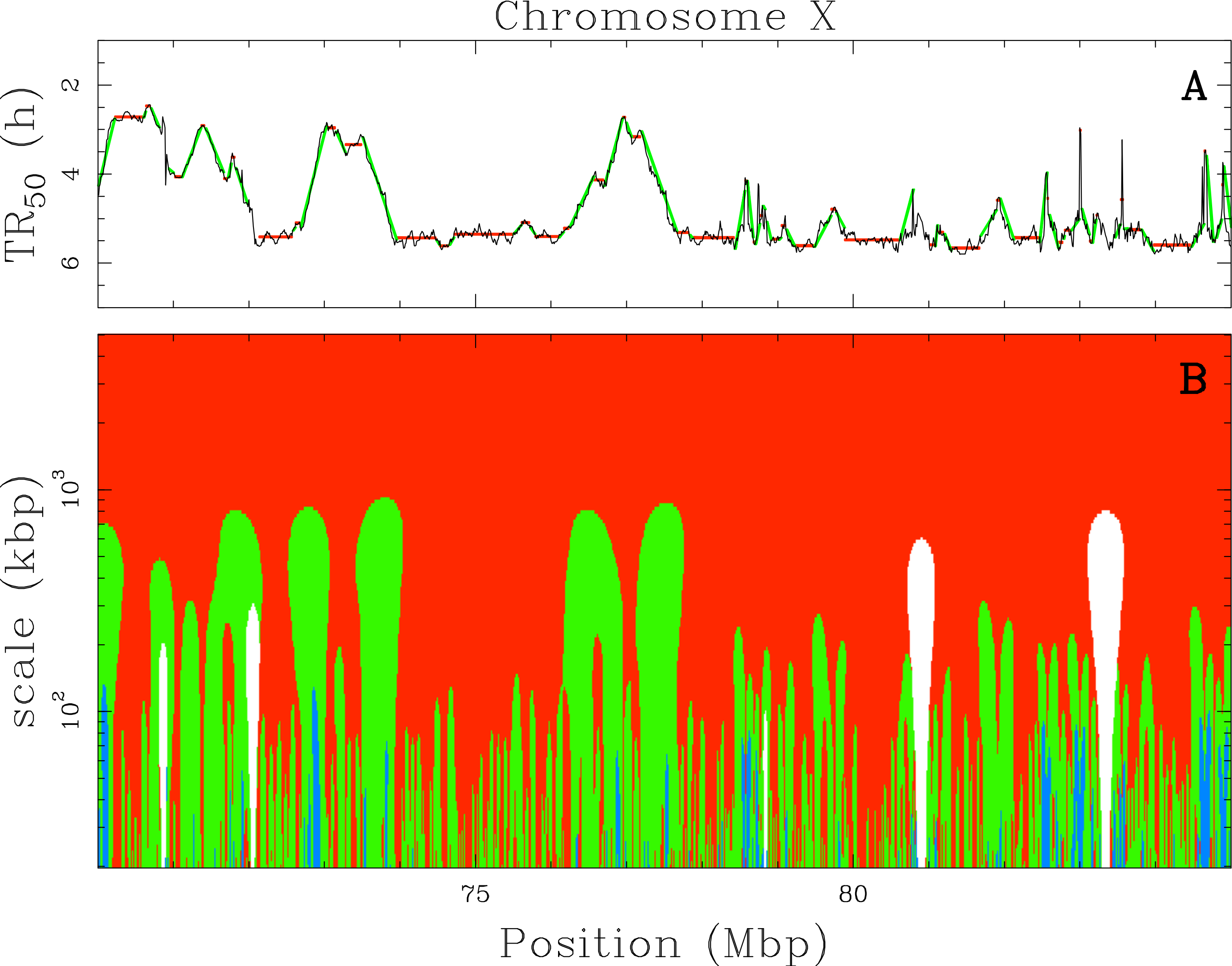

Chromosome X
A
TR50 (h)
2
4
6
B
scale (kbp)
10^3
10^2
75
80
Position (Mbp)

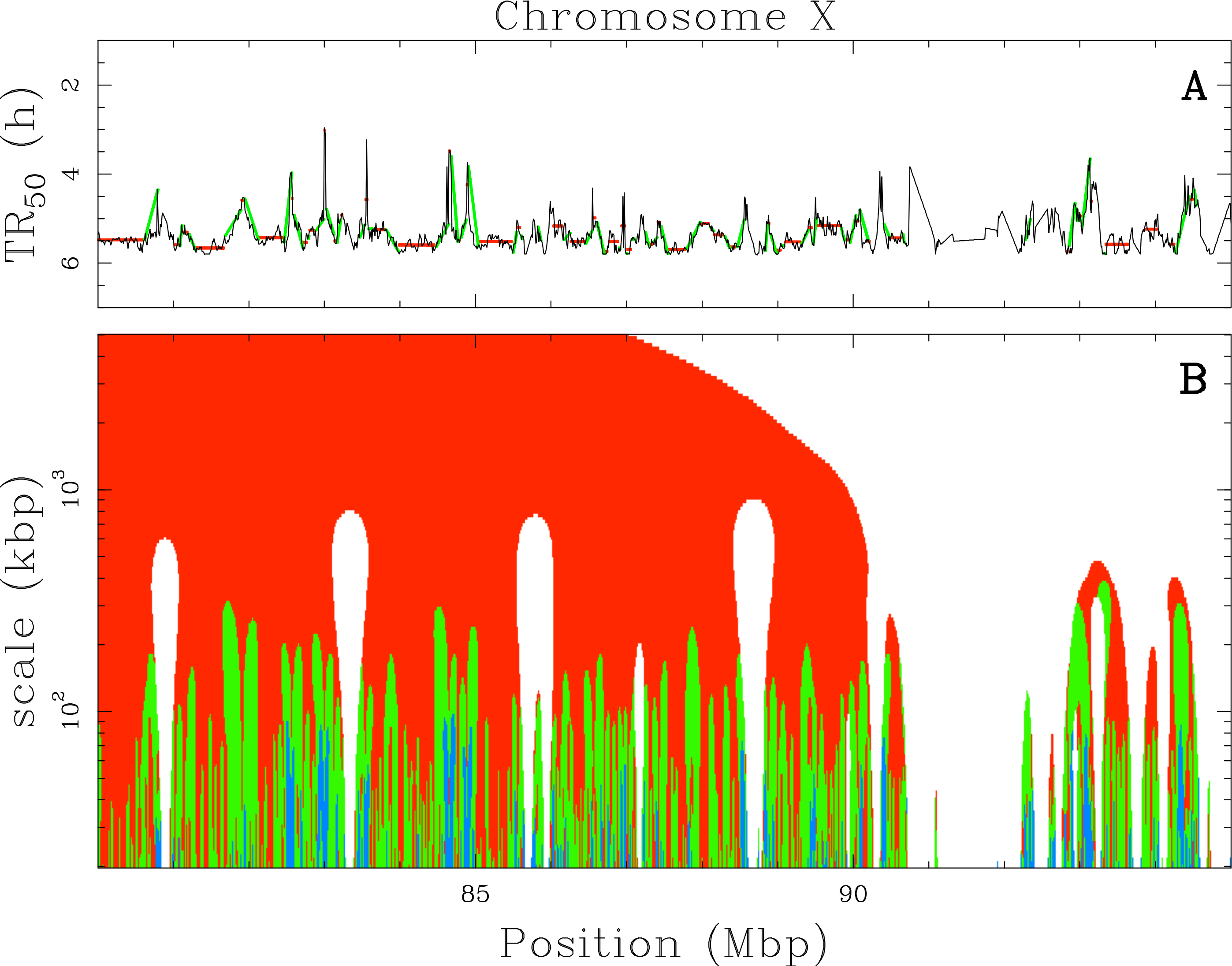
Chromosome X
A
B
TR50 (h)
2
4
6
scale (kbp)
10^3
10^2
85
90
Position (Mbp)

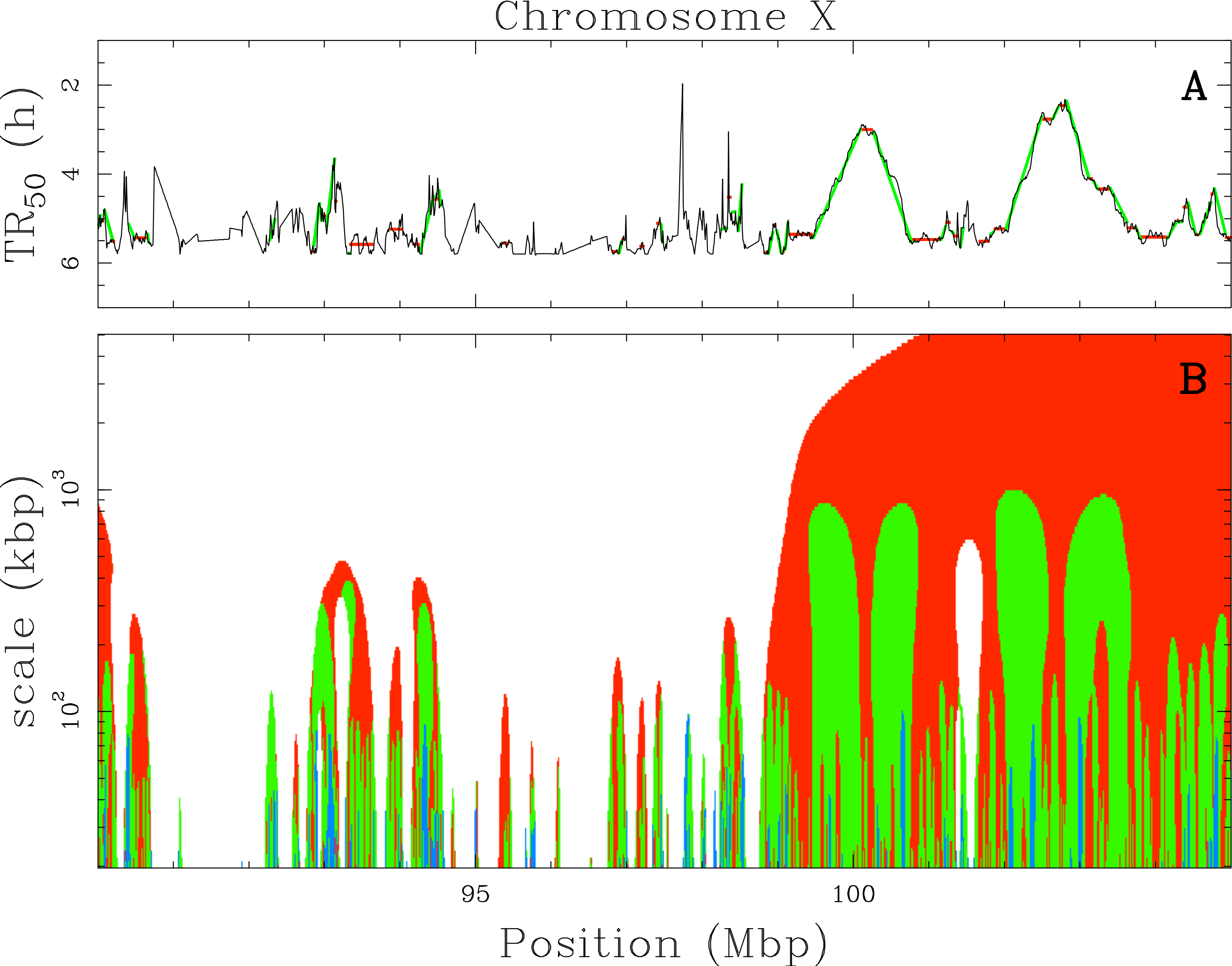

Chromosome X
A
TR50 (h)
2
4
6
B
scale (kbp)
10^3
10^2
95
100
Position (Mbp)

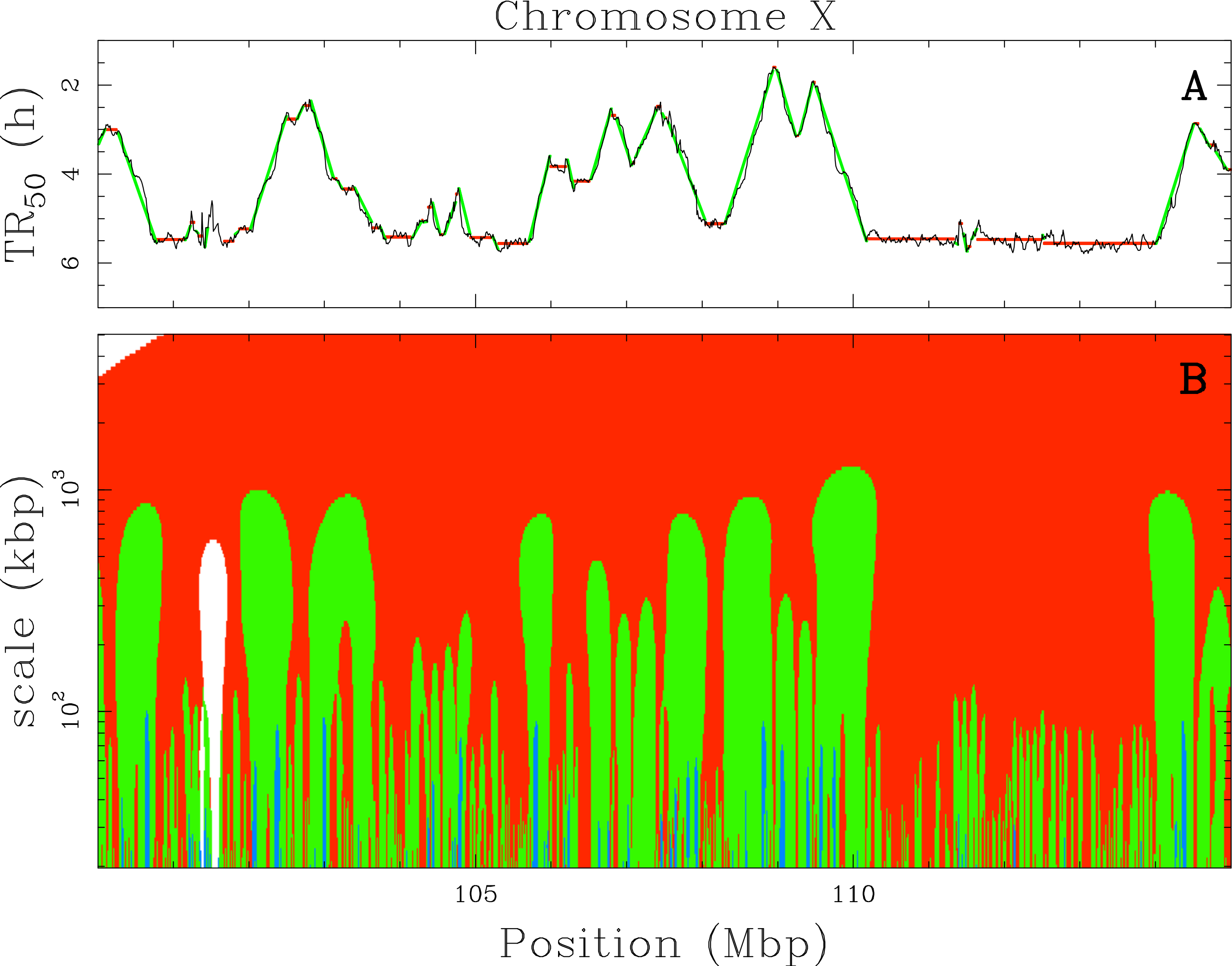
Chromosome X
A
B
TR50 (h)
2
4
6
scale (kbp)
10^3
10^2
105
110
Position (Mbp)

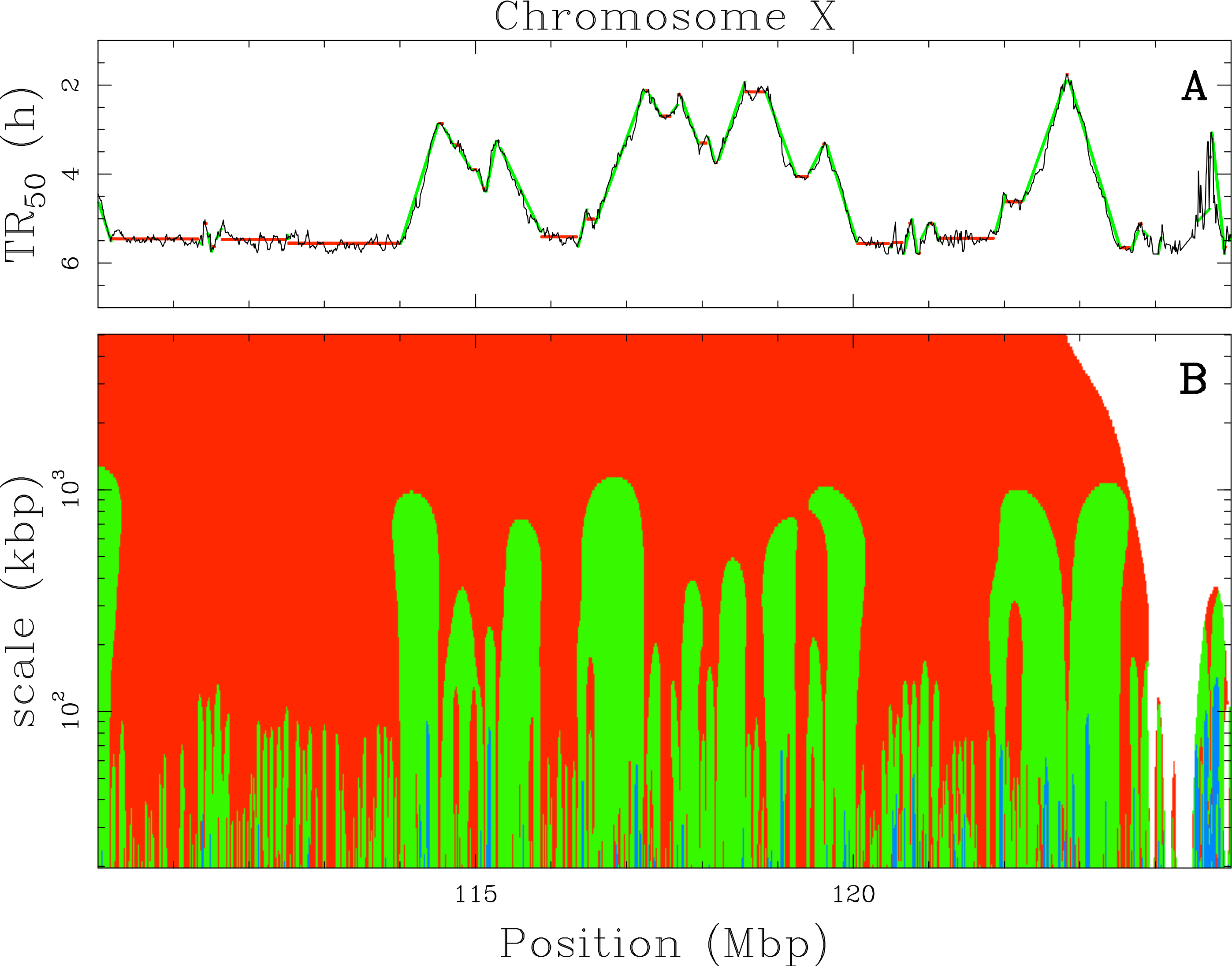

Chromosome X
A
B
TR50 (h)
2
4
6
scale (kbp)
10^3
10^2
115
120
Position (Mbp)

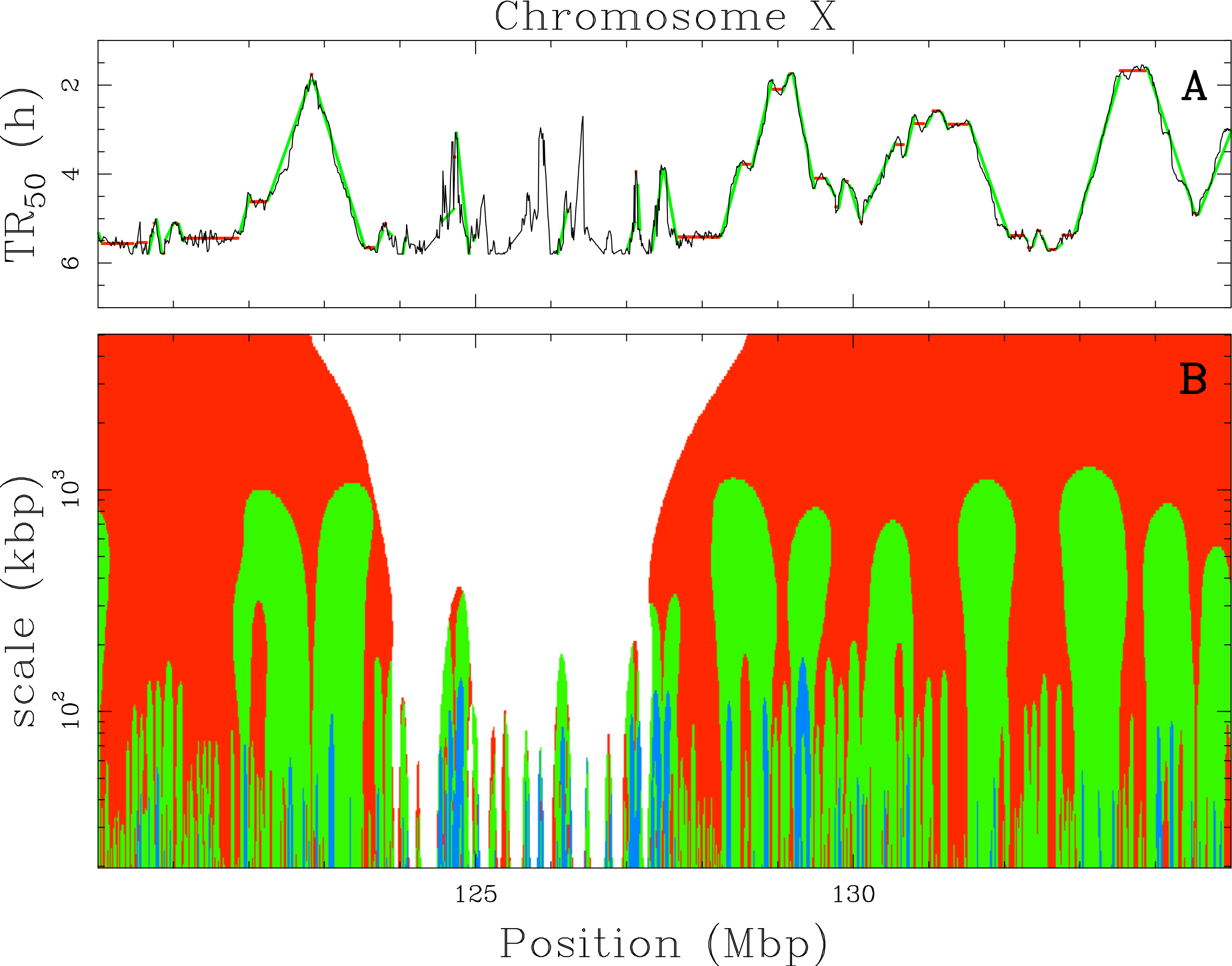

Chromosome X
A
TR_{50} (h)
2
4
6
B
scale (kbp)
10^3
10^2
125
130
Position (Mbp)

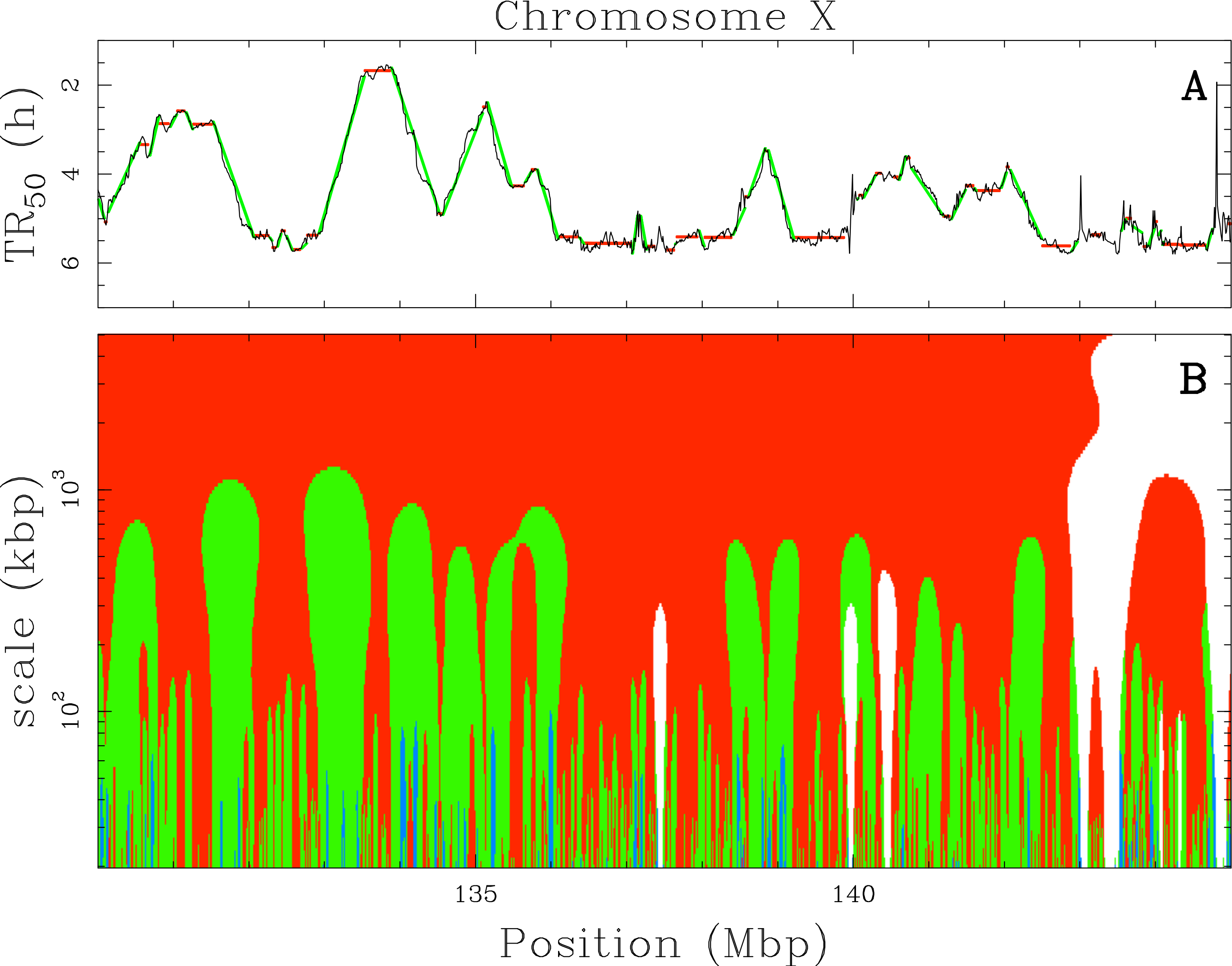

Chromosome X
A
B
TR50 (h)
2
4
6
scale (kbp)
10^3
10^2
135
140
Position (Mbp)

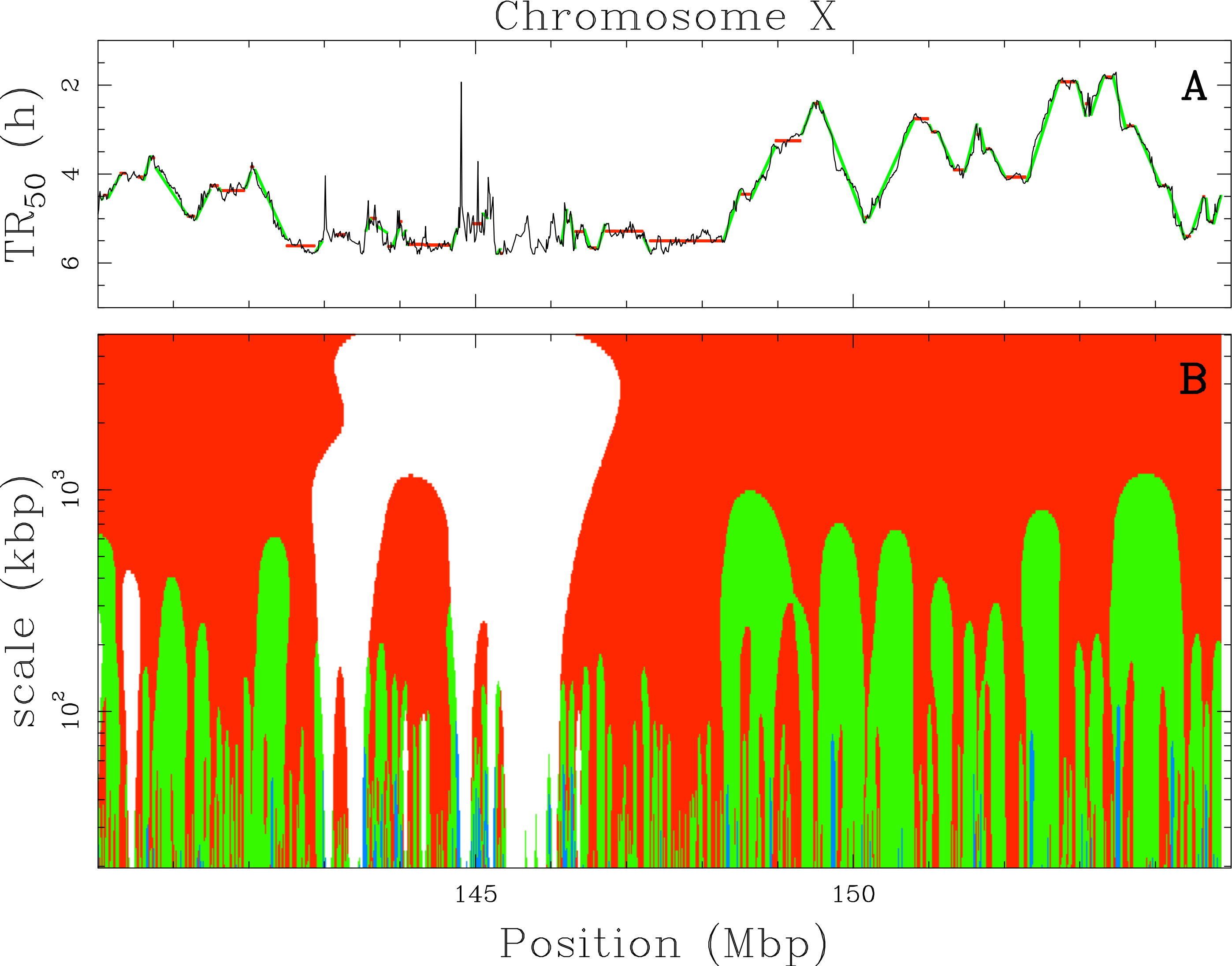

Chromosome X
A
B
TR$_{50}$ (h)
2
4
6
scale (kbp)
10^3
10^2
145
150
Position (Mbp)

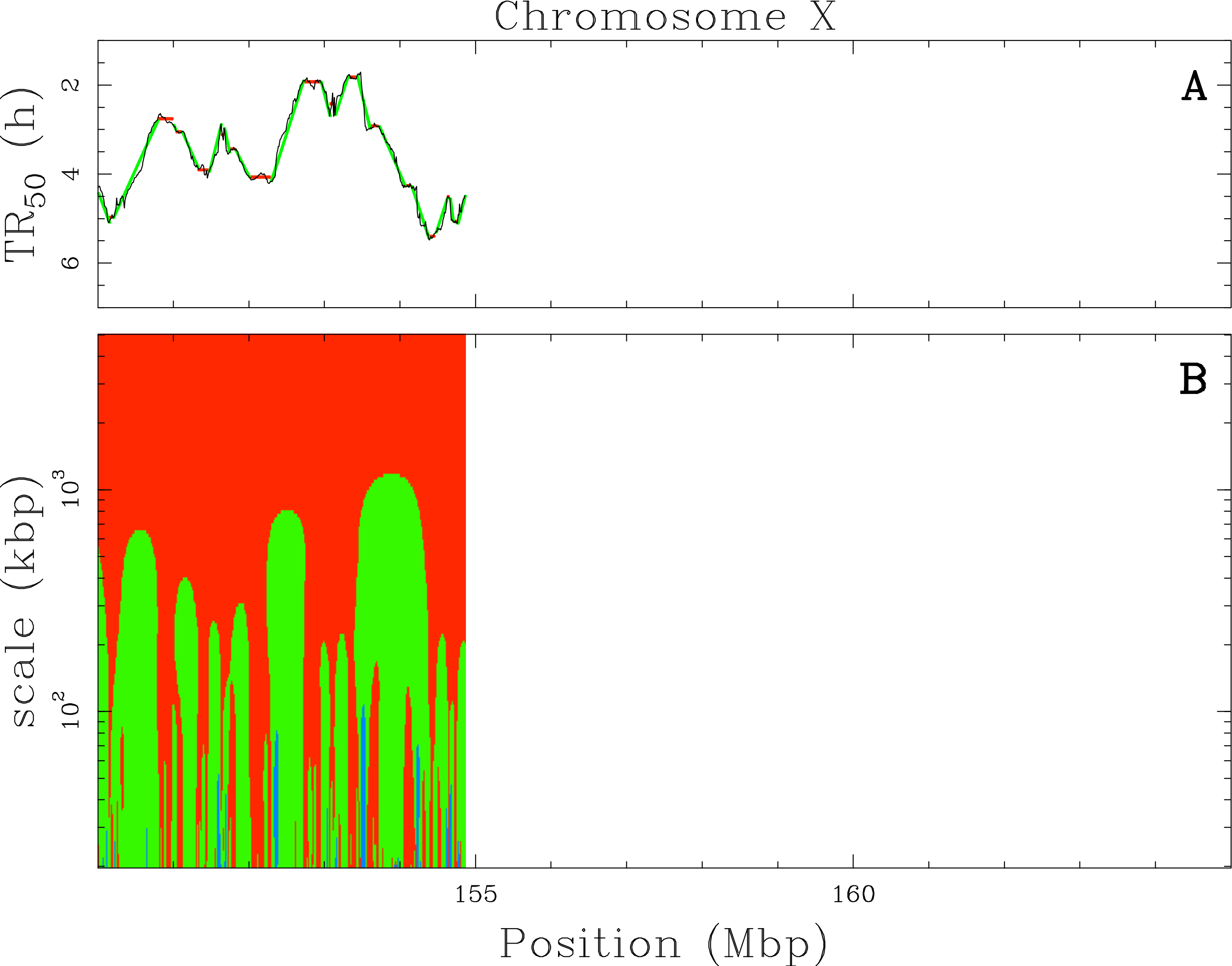

Chromosome X
A
B
TR50 (h)
2
4
6
scale (kbp)
10^3
10^2
155
160
Position (Mbp)
