# Supplementary material for: Evidence for Sequential and Increasing Activation of Replication Origins along Replication Timing Gradients in the Human Genome
Source: PLoS Comput Biol. 2011 Dec 29;7(12):e1002322. doi: 10.1371/journal.pcbi.1002322 (PMC3248390; doi:10.1371/journal.pcbi.1002322)
Supplement: Figure S4 — Complete set of all molecules of the IGH TTR analyzed by DNA combing. The top diagram shows a map of the IGH region, the position of the fosmid probes (red lines) and chromosome coordinates. The bottom panel shows the complete set of molecules schematized in Figure 8D. (PDF) [file pcbi.1002322.s004.pdf]

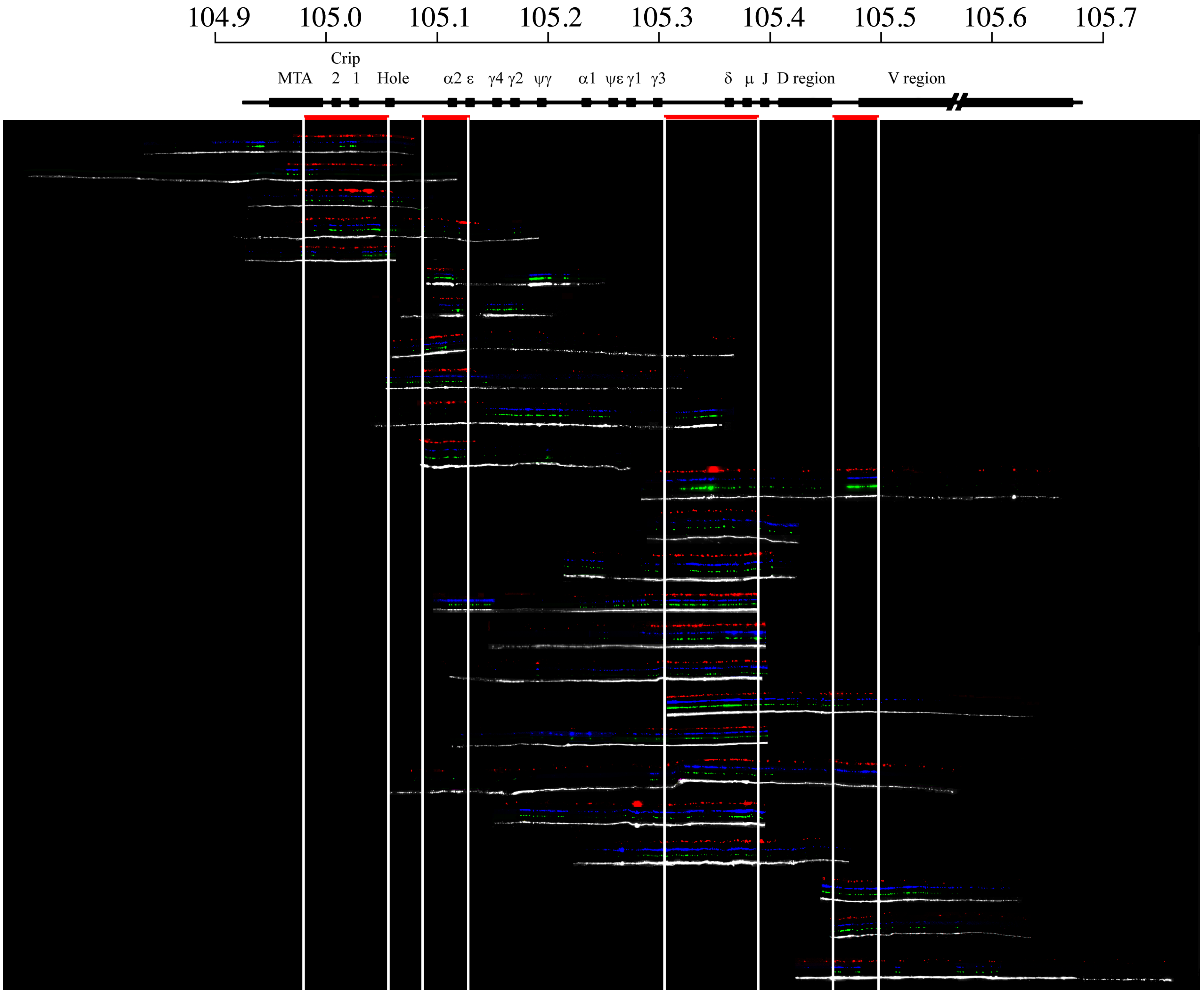
104.9
105.0
105.1
105.2
105.3
105.4
105.5
105.6
105.7
Crip
MTA
2
1
Hole
α2 ε
γ4 γ2
ψγ
α1
ψε γ1
γ3
δ
μ
J
D region
V region
