# Supplementary material for: Evidence for Sequential and Increasing Activation of Replication Origins along Replication Timing Gradients in the Human Genome
Source: PLoS Comput Biol. 2011 Dec 29;7(12):e1002322. doi: 10.1371/journal.pcbi.1002322 (PMC3248390; doi:10.1371/journal.pcbi.1002322)

Replication Timing Vs Encode Origin data, ENr231 (chr1:149424684_149924684)

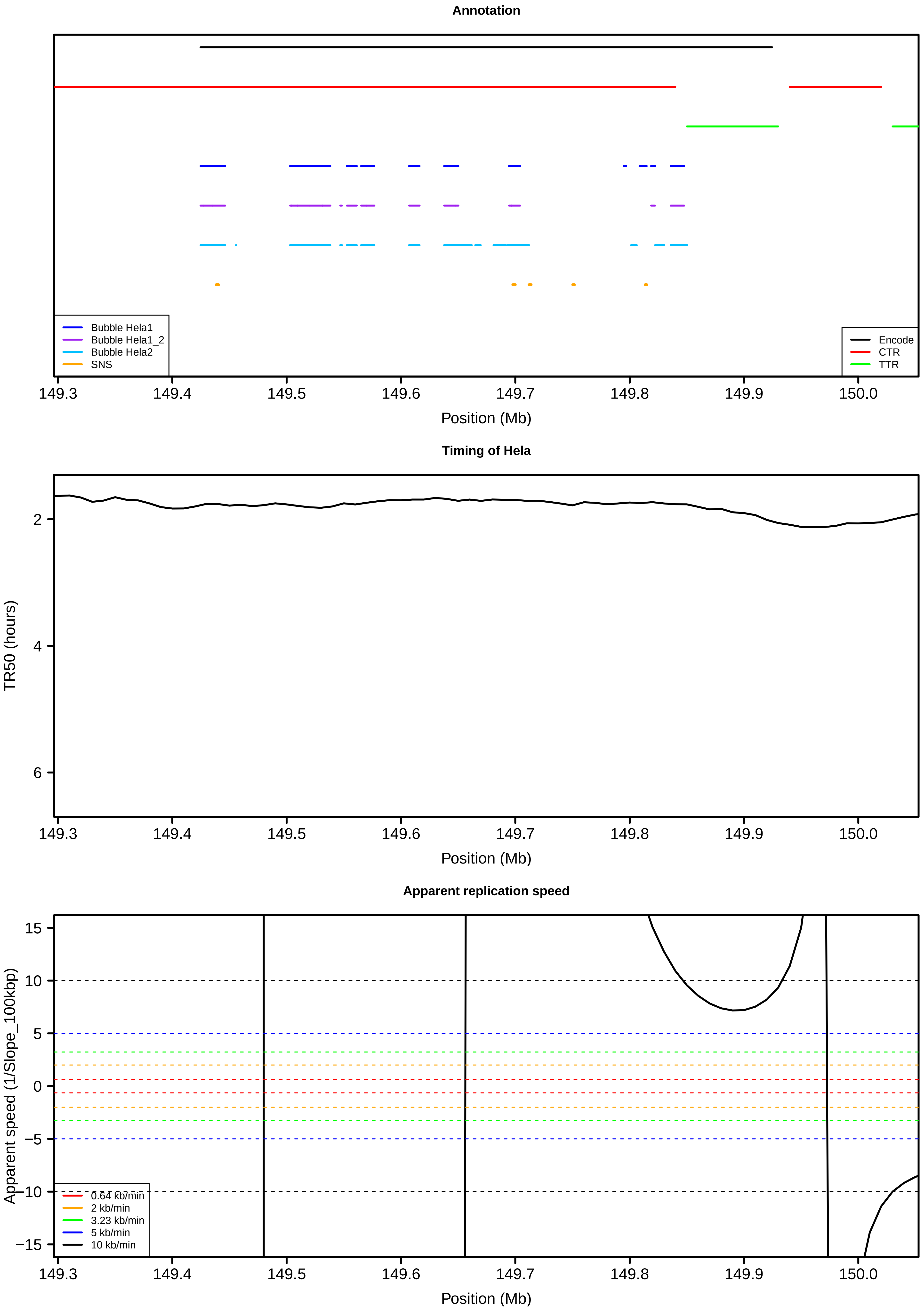

Replication Timing Vs Encode Origin data, ENr112 (chr2:51512208_52012208)

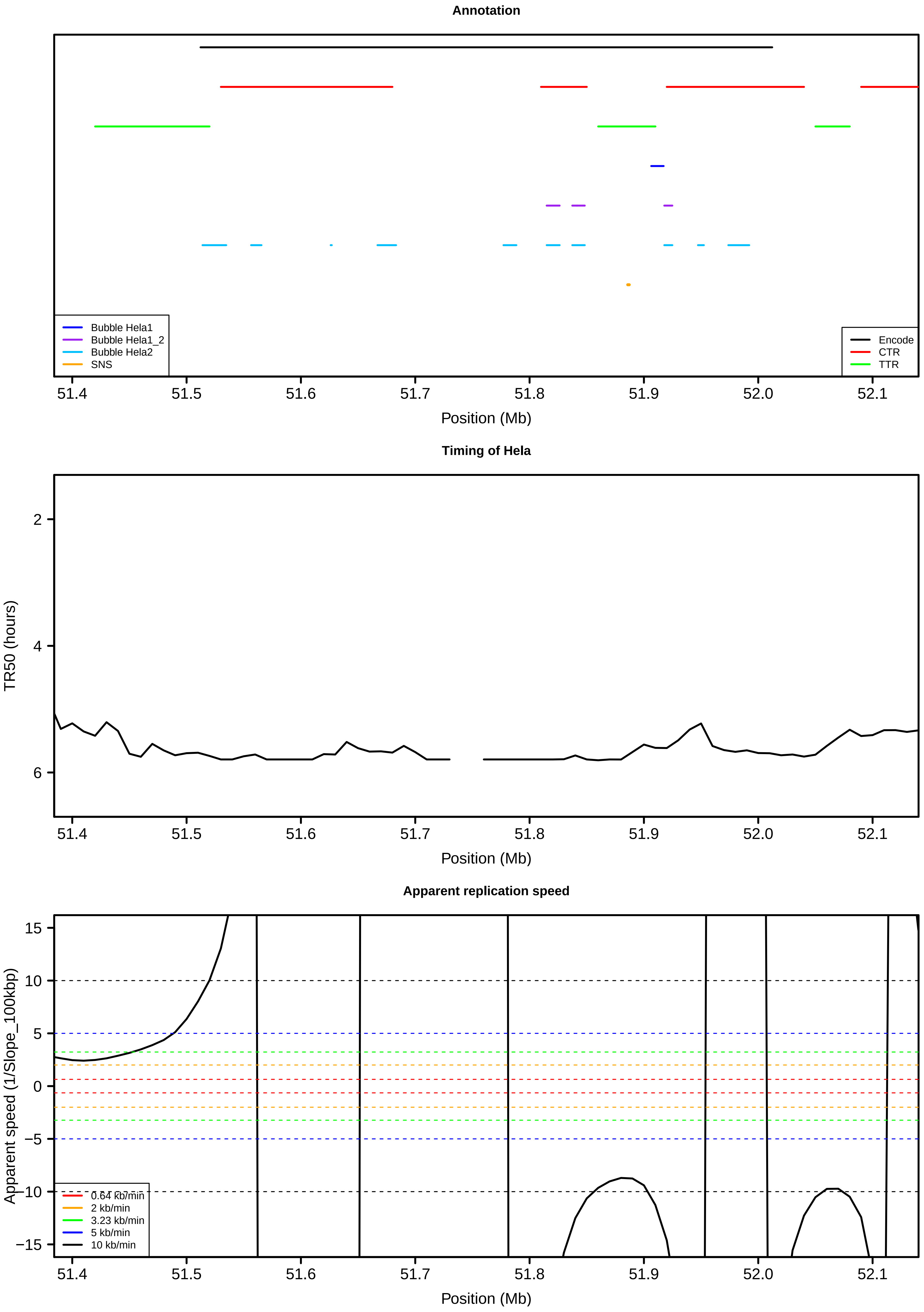

Replication Timing Vs Encode Origin data, ENr121 (chr2:118011043_118511043)

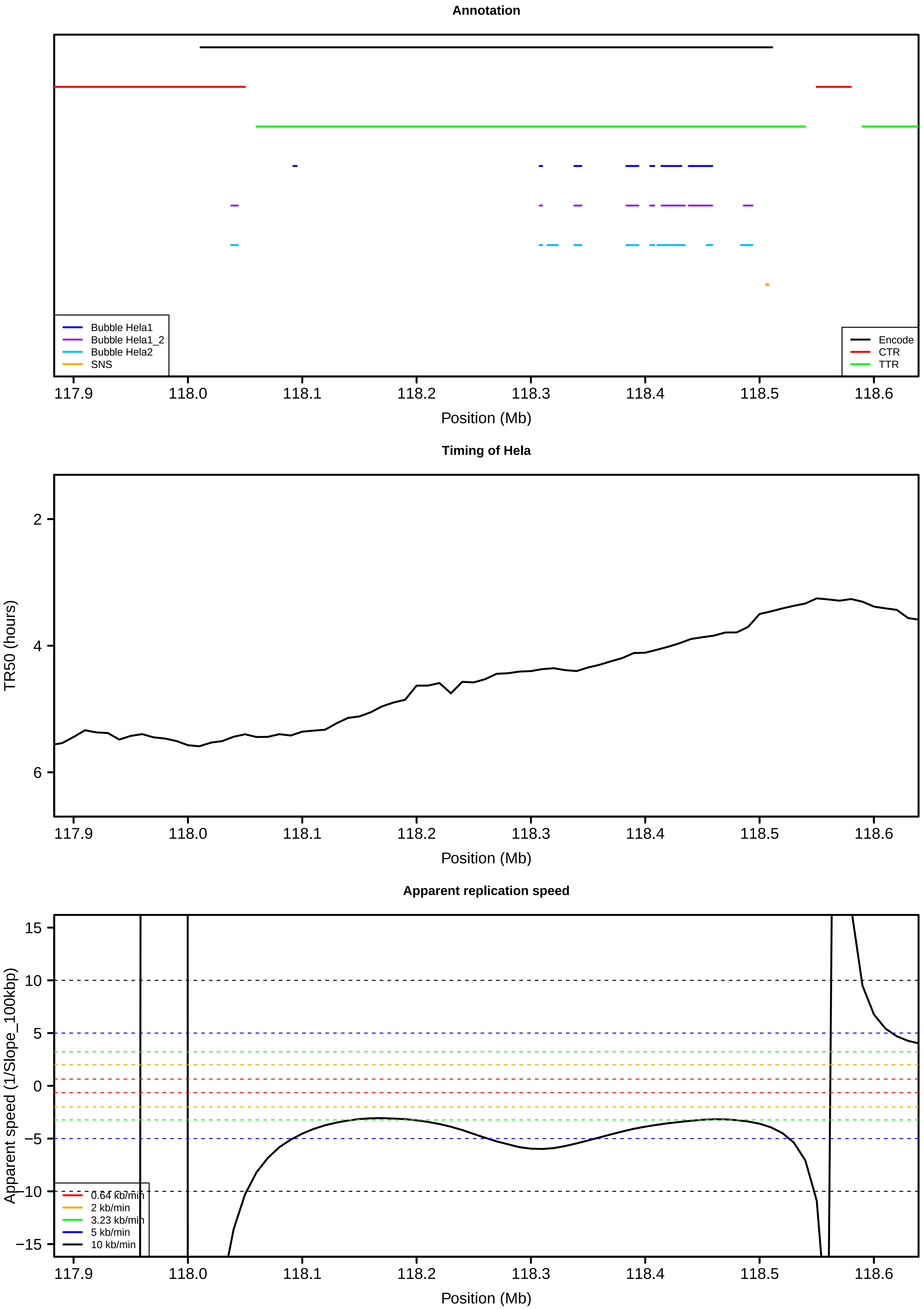

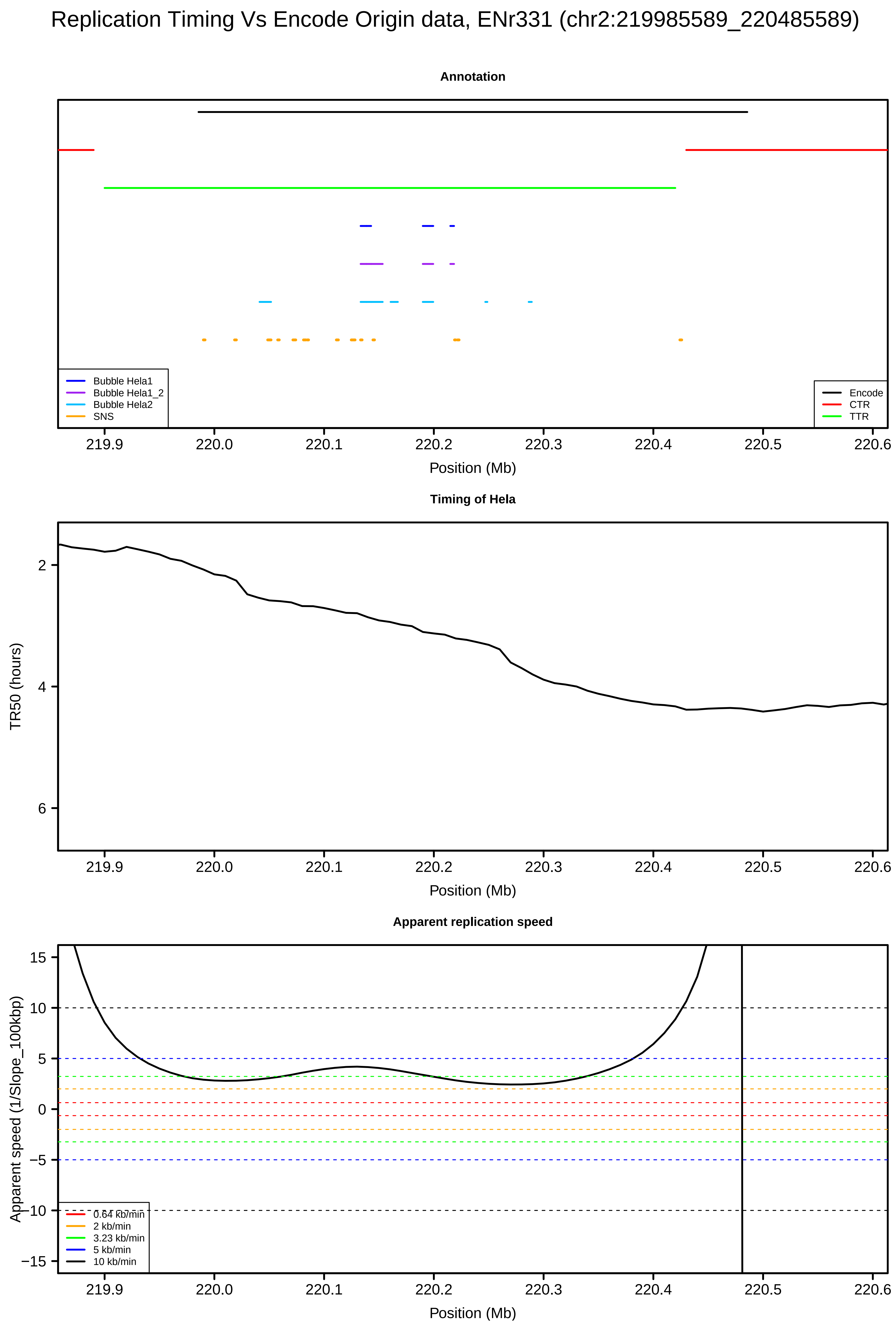
Replication Timing Vs Encode Origin data, ENr331 (chr2:219985589_220485589)
Annotation
Bubble Hela1
Bubble Hela1_2
Bubble Hela2
SNS
Encode
CTR
TTR
219.9
220.0
220.1
220.2
220.3
220.4
220.5
220.6
Position (Mb)
Timing of Hela
TR50 (hours)
2
4
6
Apparent replication speed
Apparent speed (1/Slope_100kbp)
15
10
5
0
−5
−10
−15
0.64 kb/min
2 kb/min
3.23 kb/min
5 kb/min
10 kb/min

Replication Timing Vs Encode Origin data, ENr131 (chr2:234156563_234656627)

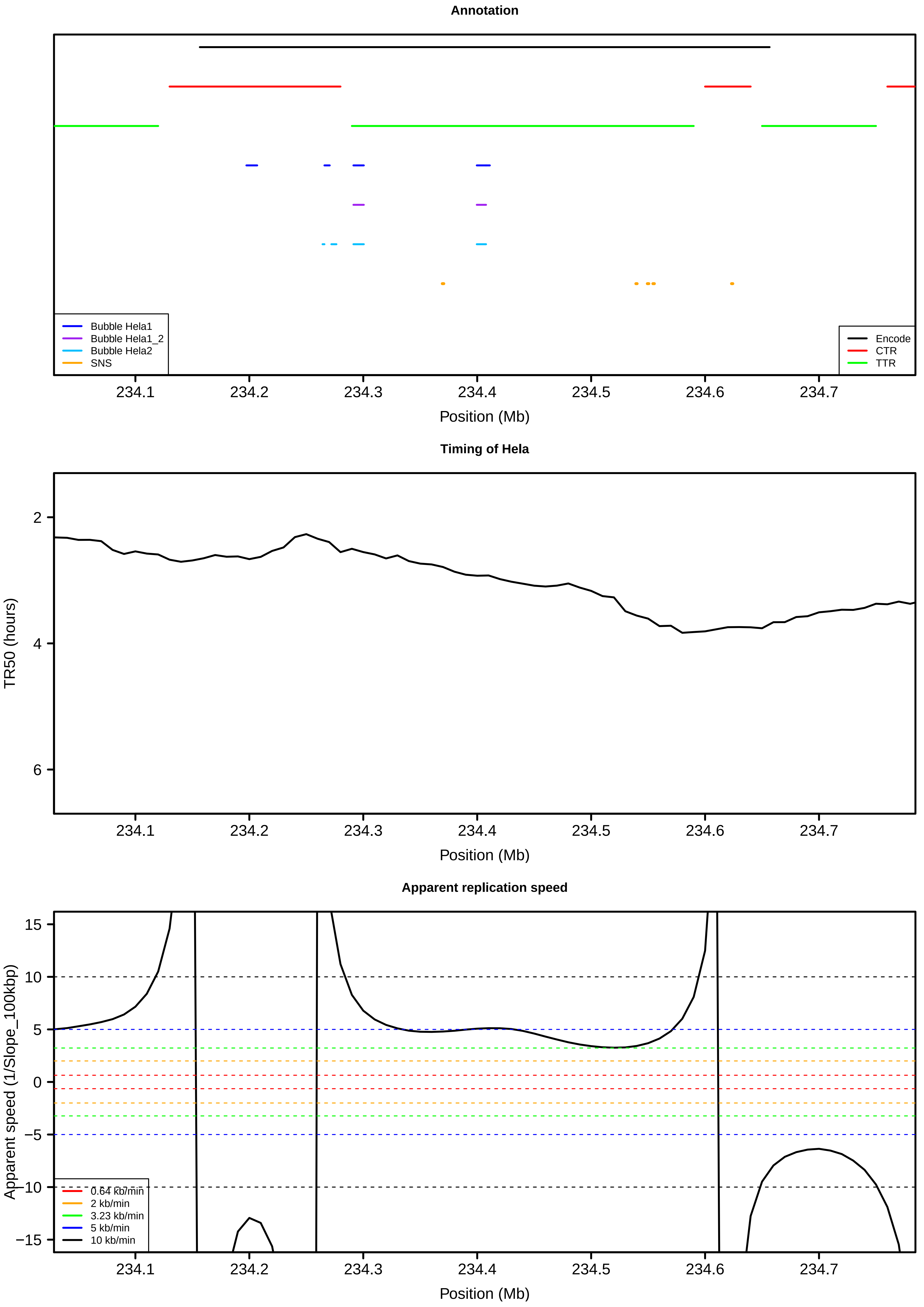

Replication Timing Vs Encode Origin data, ENr113 (chr4:118466103_118966103)

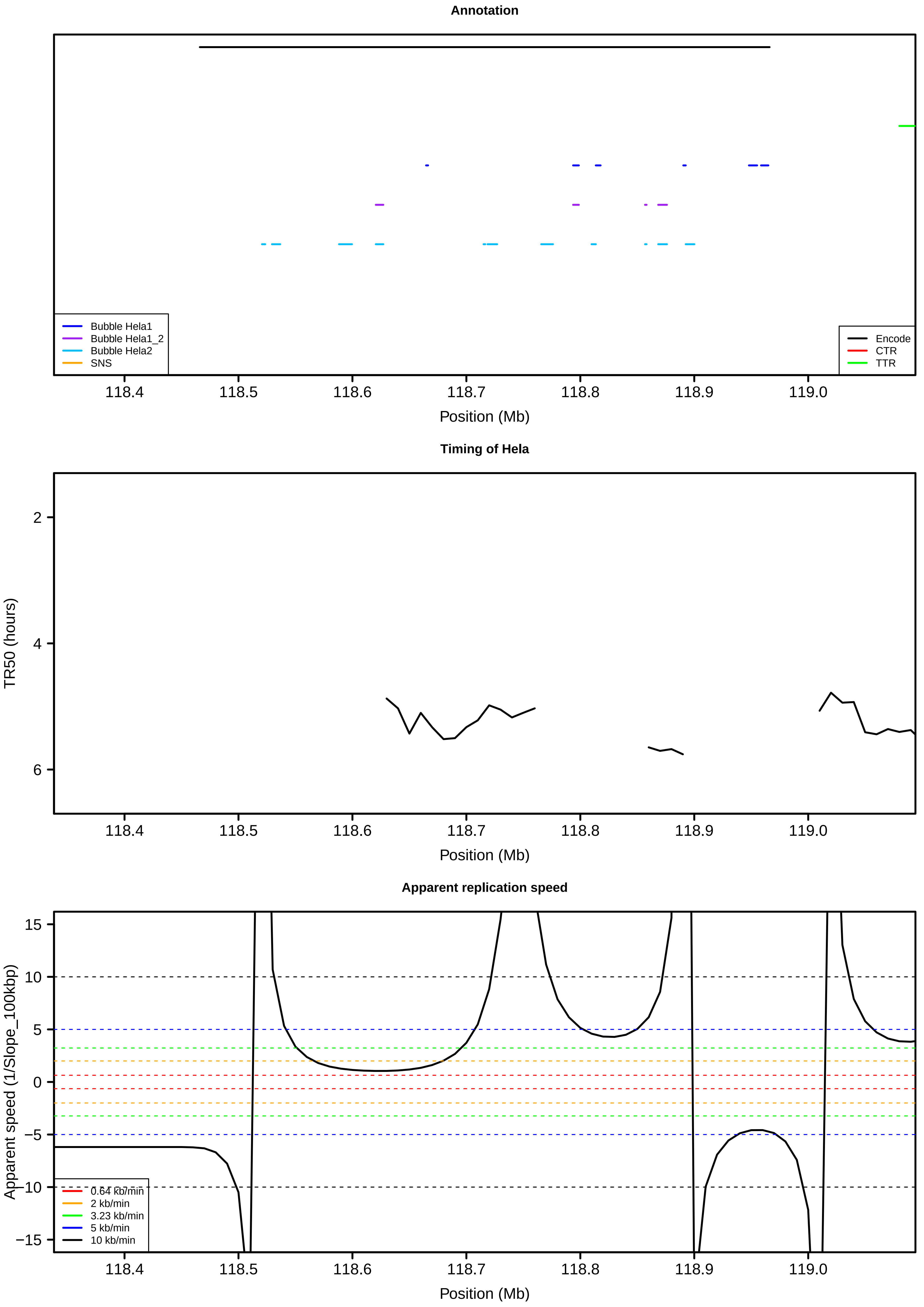

Replication Timing Vs Encode Origin data, ENr221 (chr5:55871006_56371006)

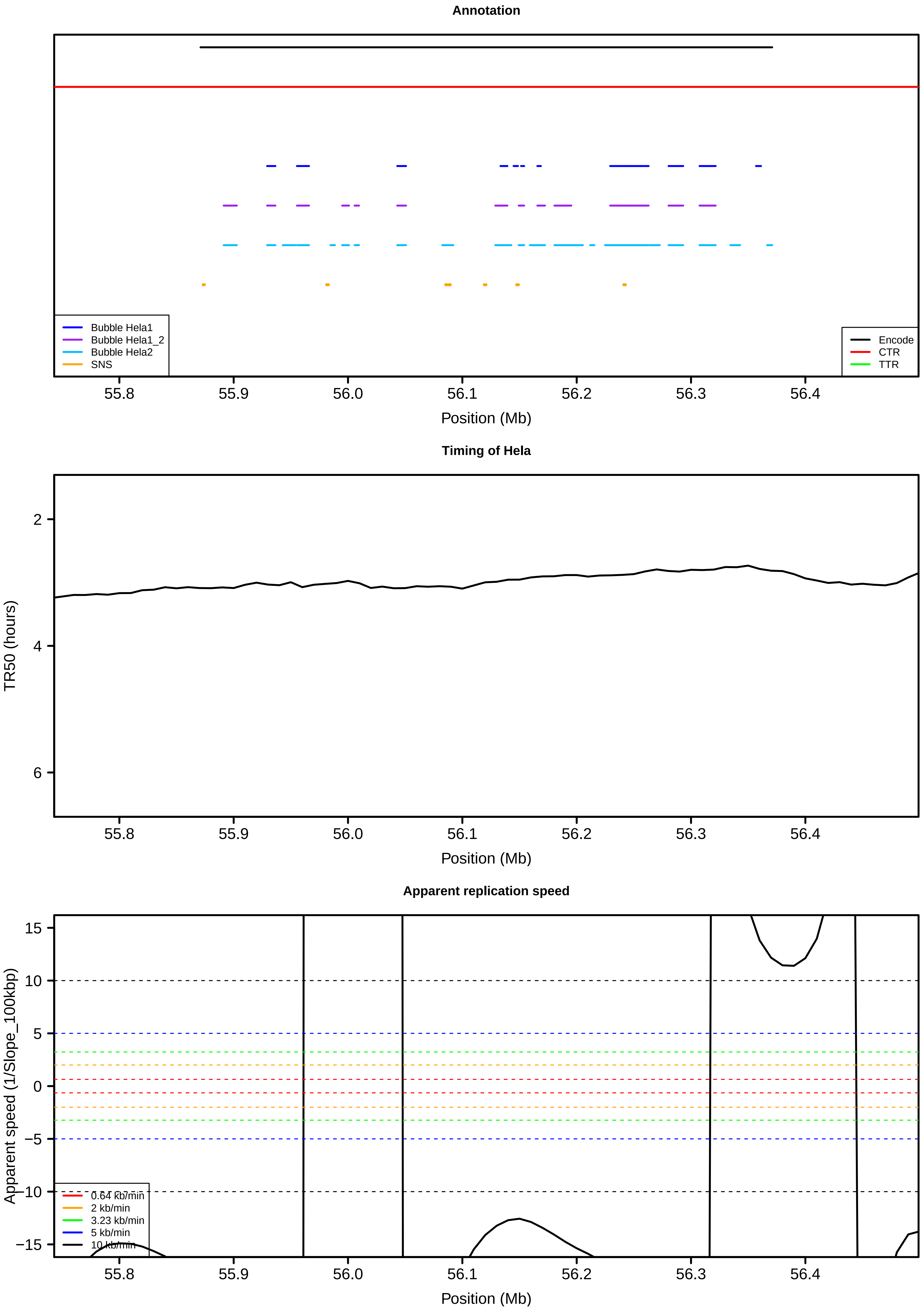

Replication Timing Vs Encode Origin data, ENm002 (chr5:131284313_132284313)

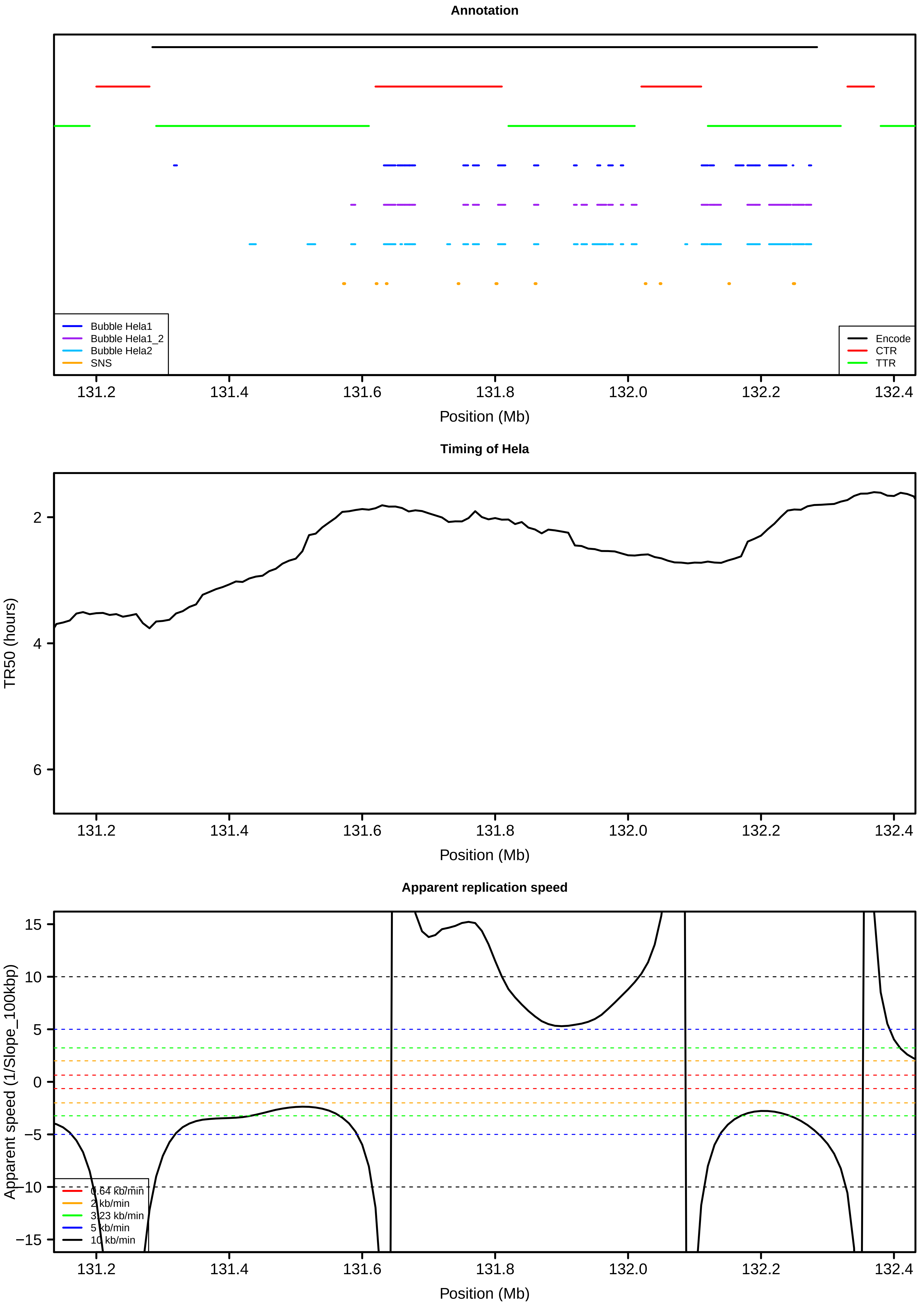

Replication Timing Vs Encode Origin data, ENr212 (chr5:141880150_142380150)

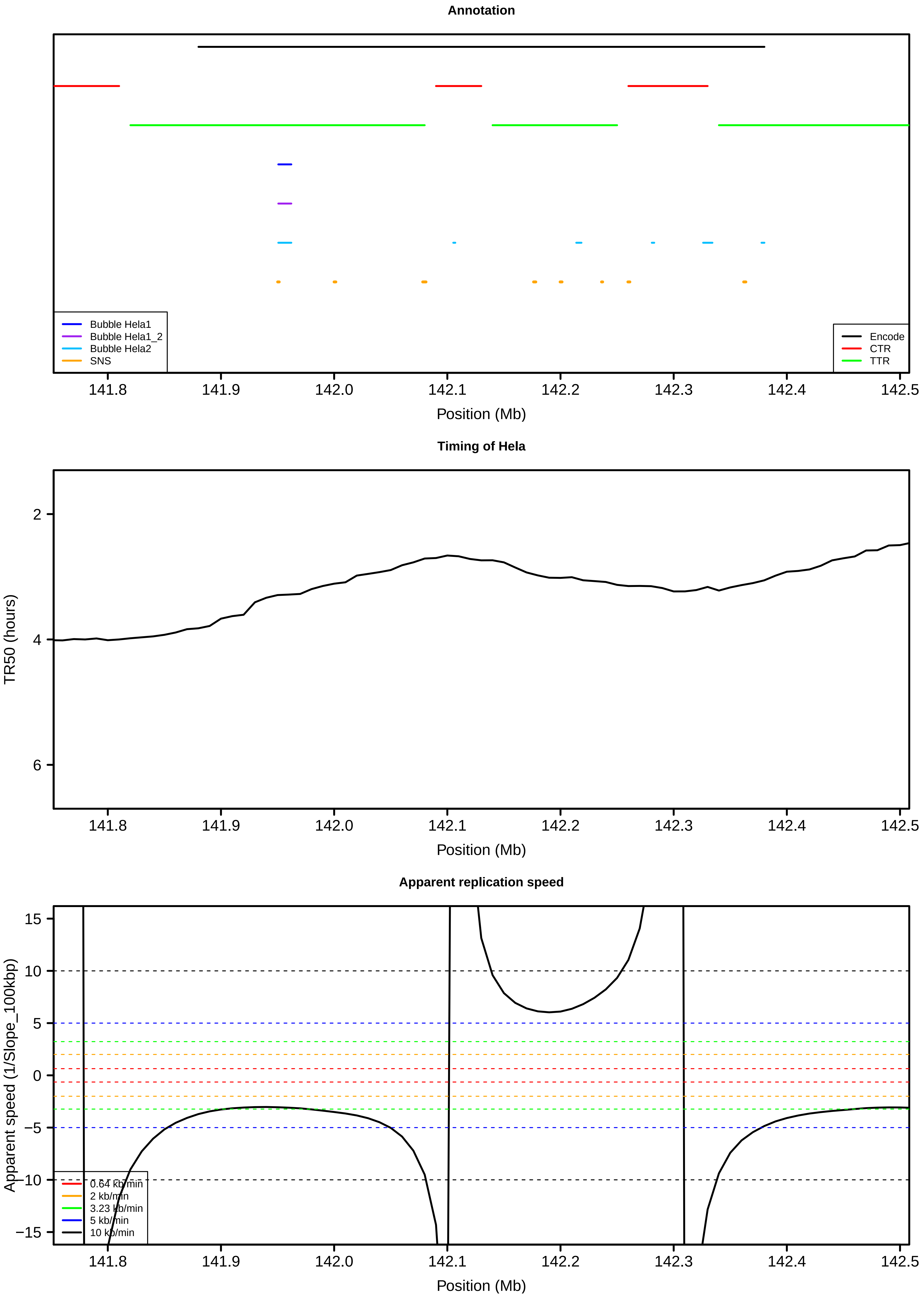

Replication Timing Vs Encode Origin data, ENr334 (chr6:41405894_41905894)

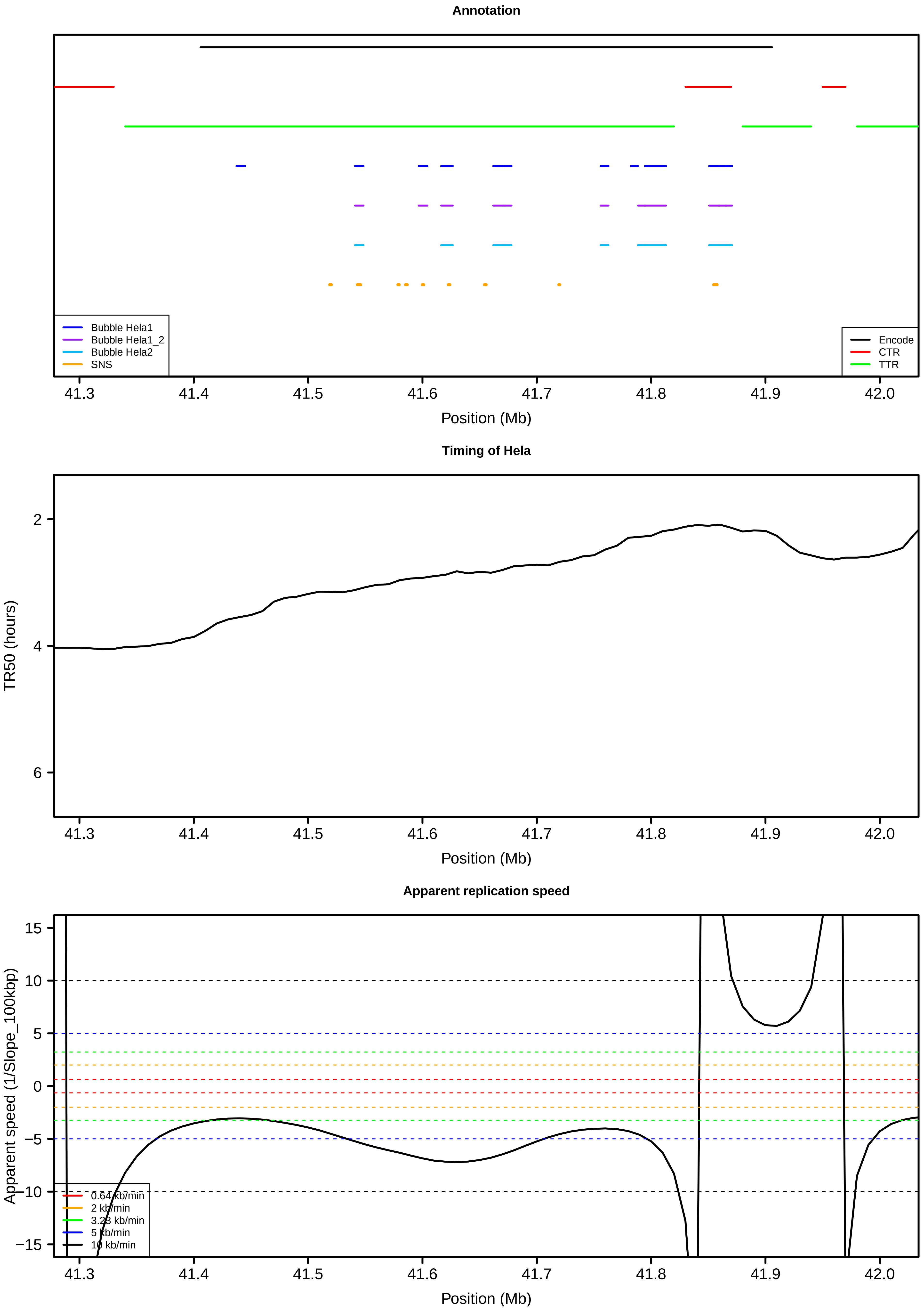

Replication Timing Vs Encode Origin data, ENr223 (chr6:73789952_74289952)

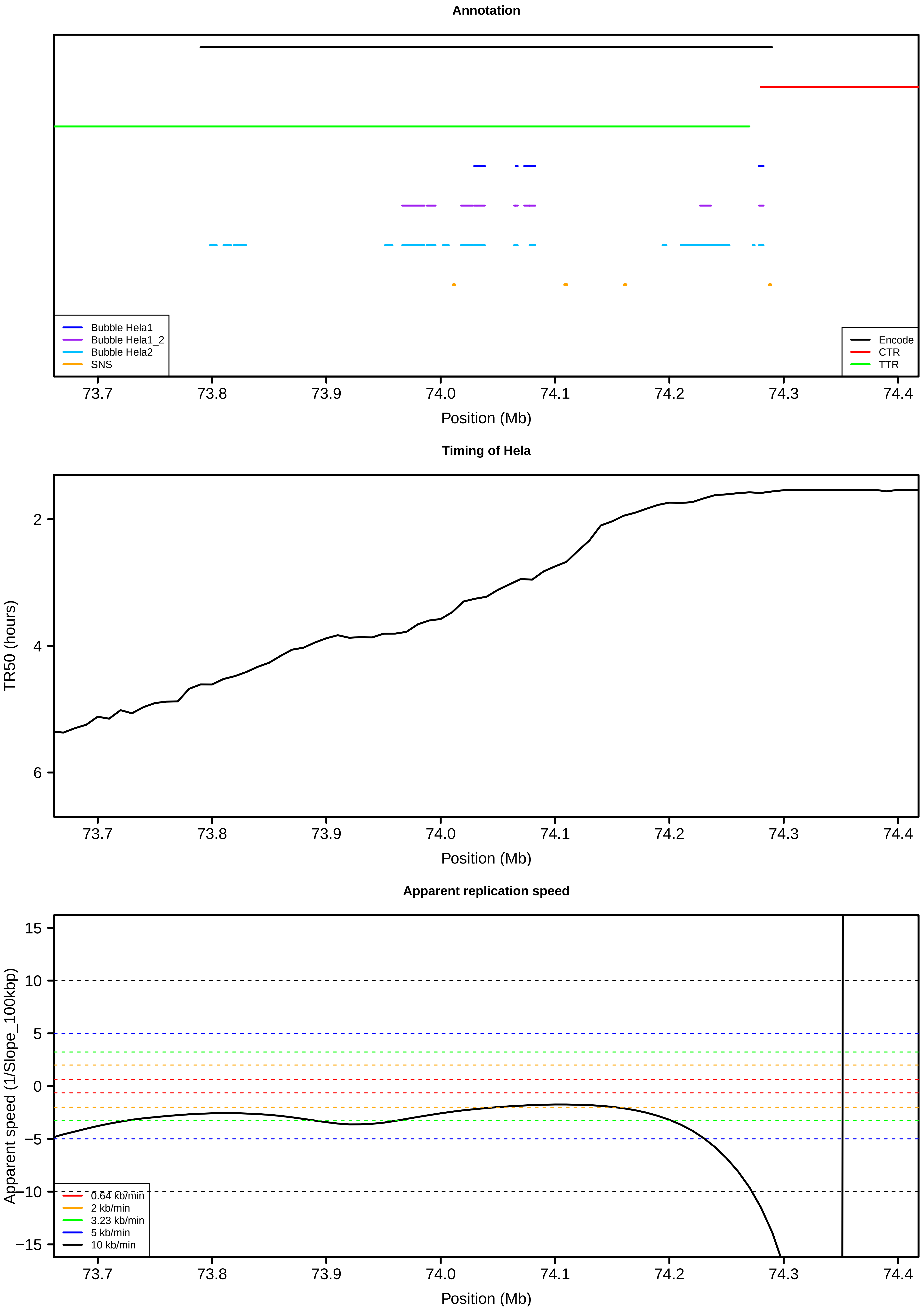

Replication Timing Vs Encode Origin data, ENr323 (chr6:108371396_108871396)

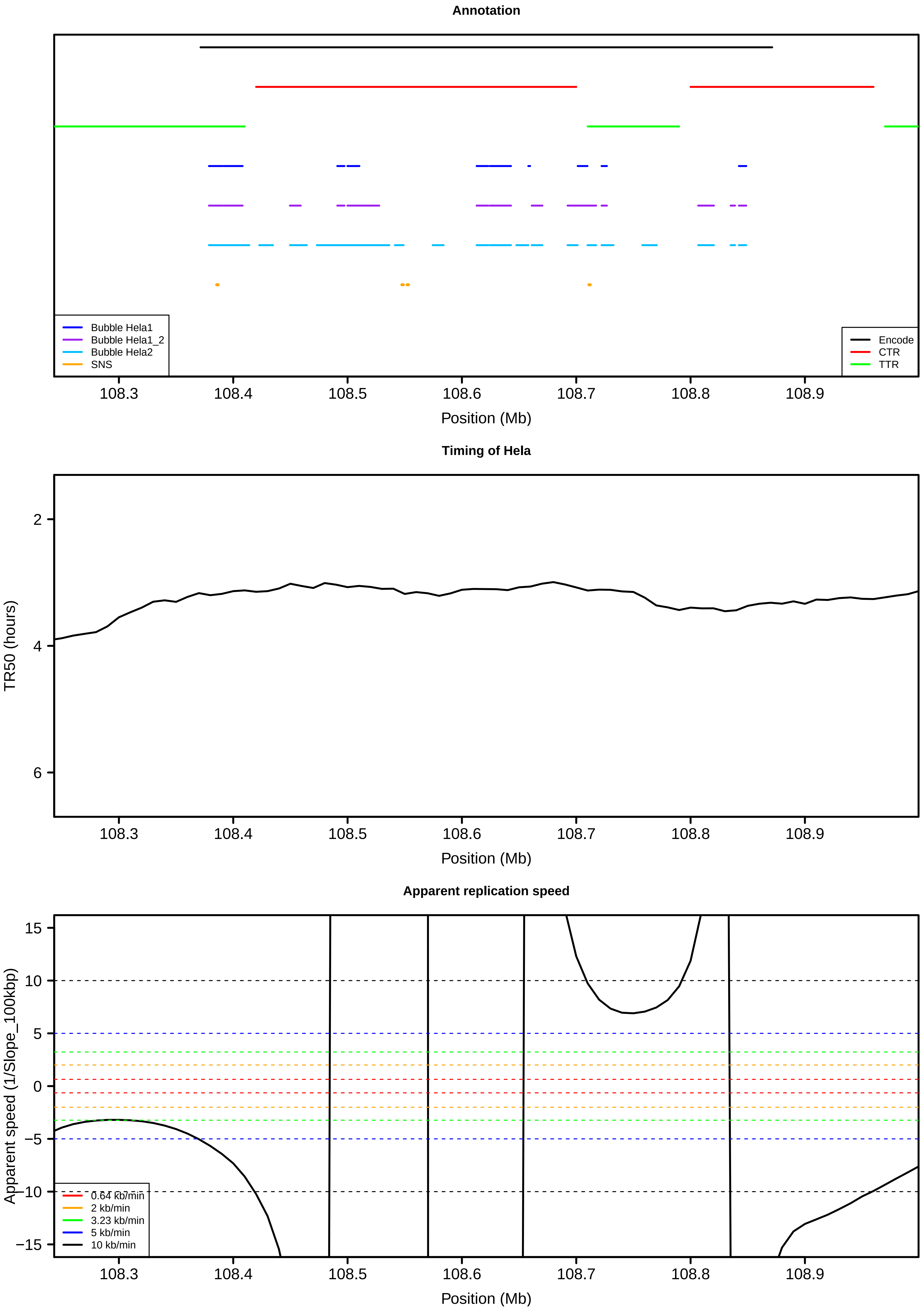

Replication Timing Vs Encode Origin data, ENr222 (chr6:132218539_132718539)

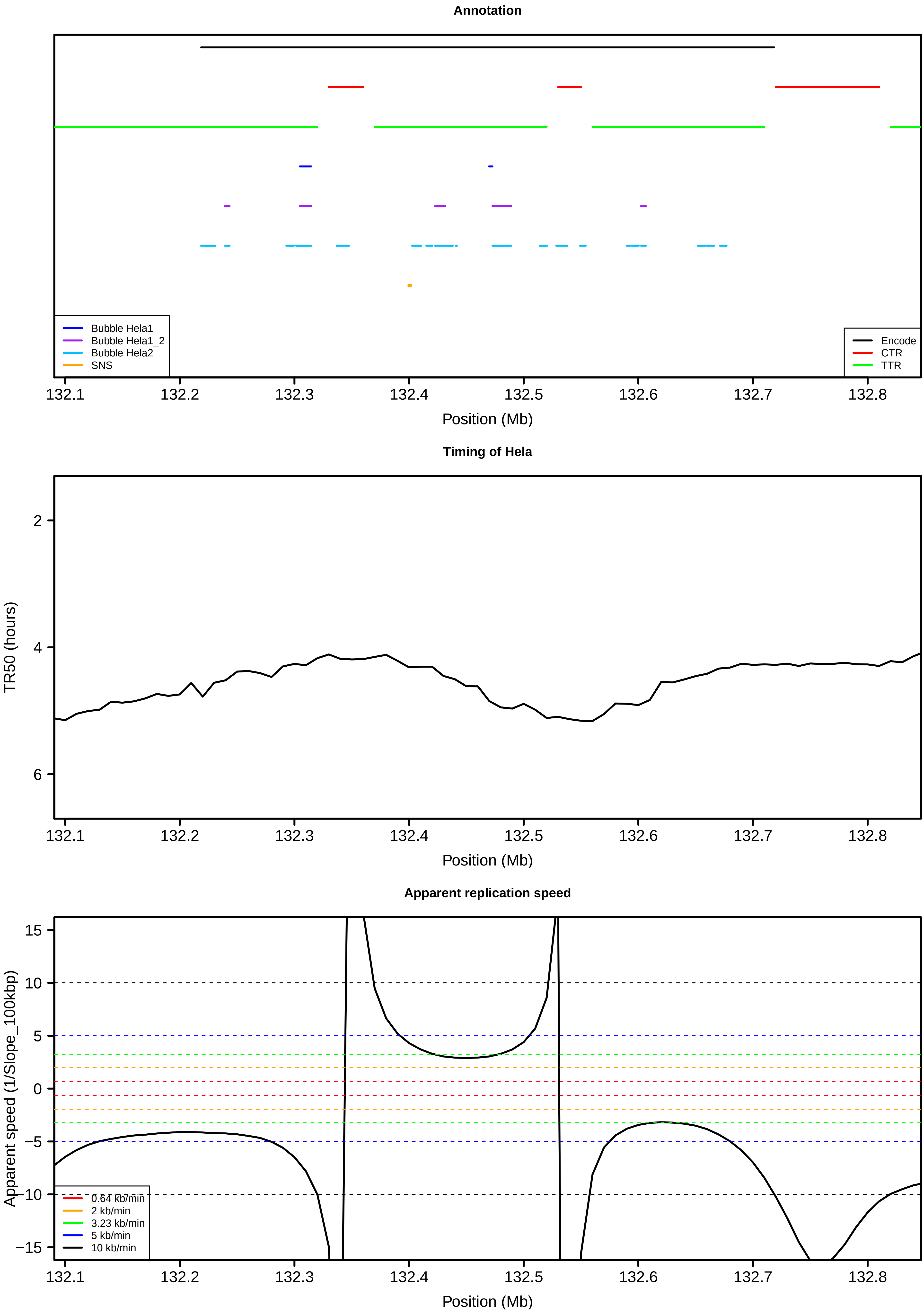

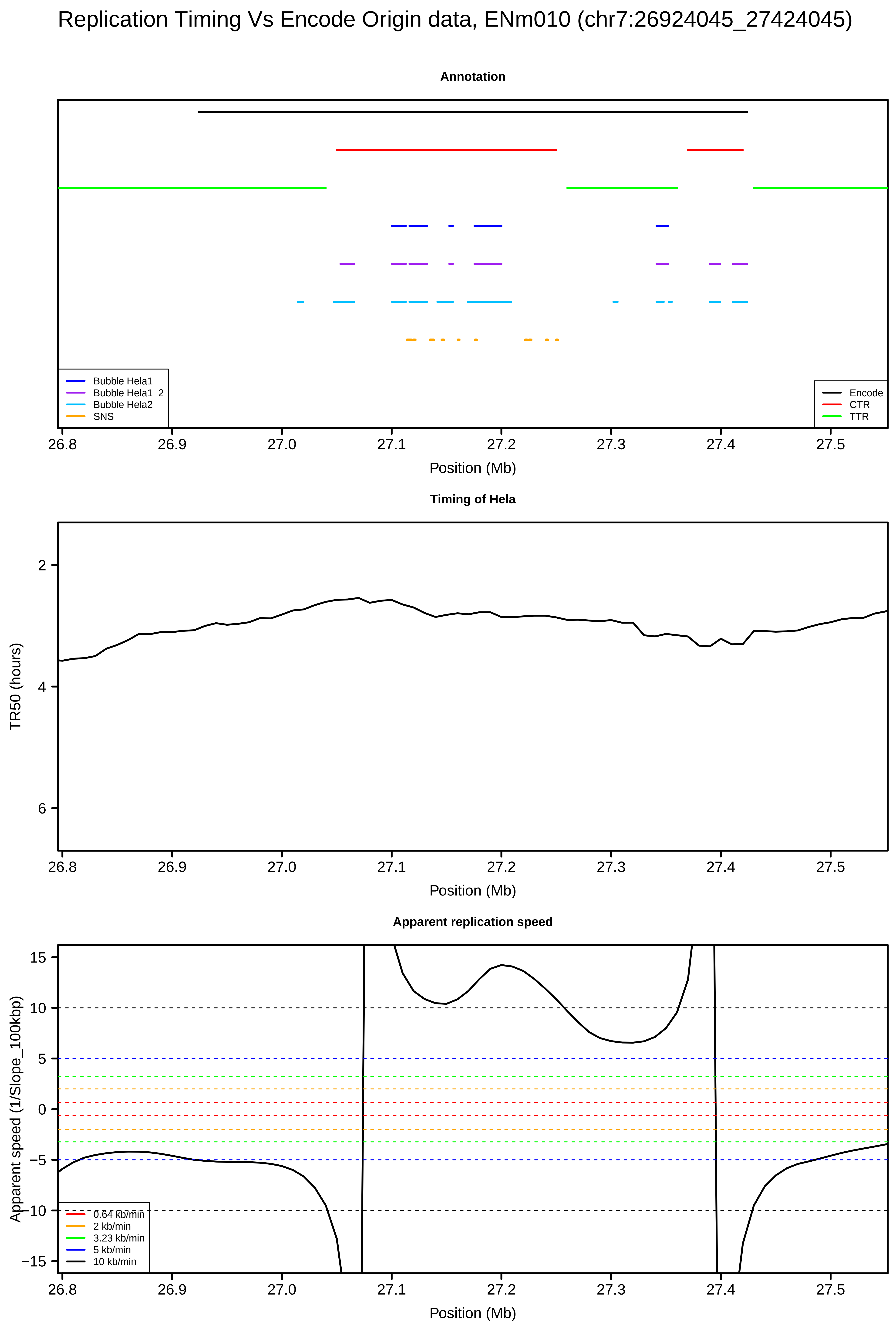

Replication Timing Vs Encode Origin data, ENm010 (chr7:26924045_27424045)
Annotation
Bubble Hela1
Bubble Hela1_2
Bubble Hela2
SNS
Encode
CTR
TTR
26.8
26.9
27.0
27.1
27.2
27.3
27.4
27.5
Position (Mb)
Timing of Hela
TR50 (hours)
2
4
6
Position (Mb)
Apparent replication speed
Apparent speed (1/Slope_100kbp)
15
10
5
0
−5
−10
−15
0.64 kb/min
2 kb/min
3.23 kb/min
5 kb/min
10 kb/min
Position (Mb)

Replication Timing Vs Encode Origin data, ENm013 (chr7:89621624_90736048)

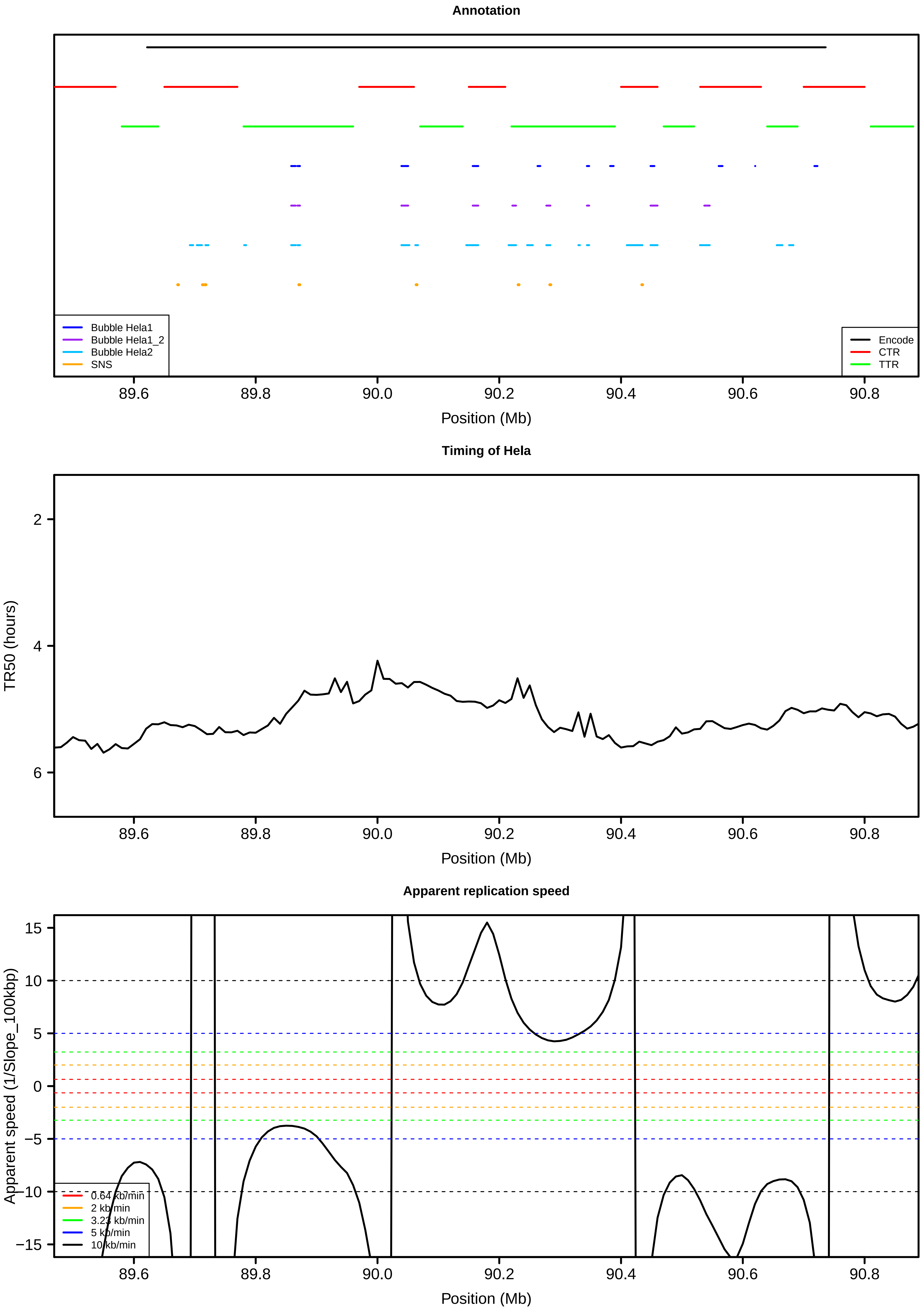

Replication Timing Vs Encode Origin data, ENm012 (chr7:113720368_114720368)

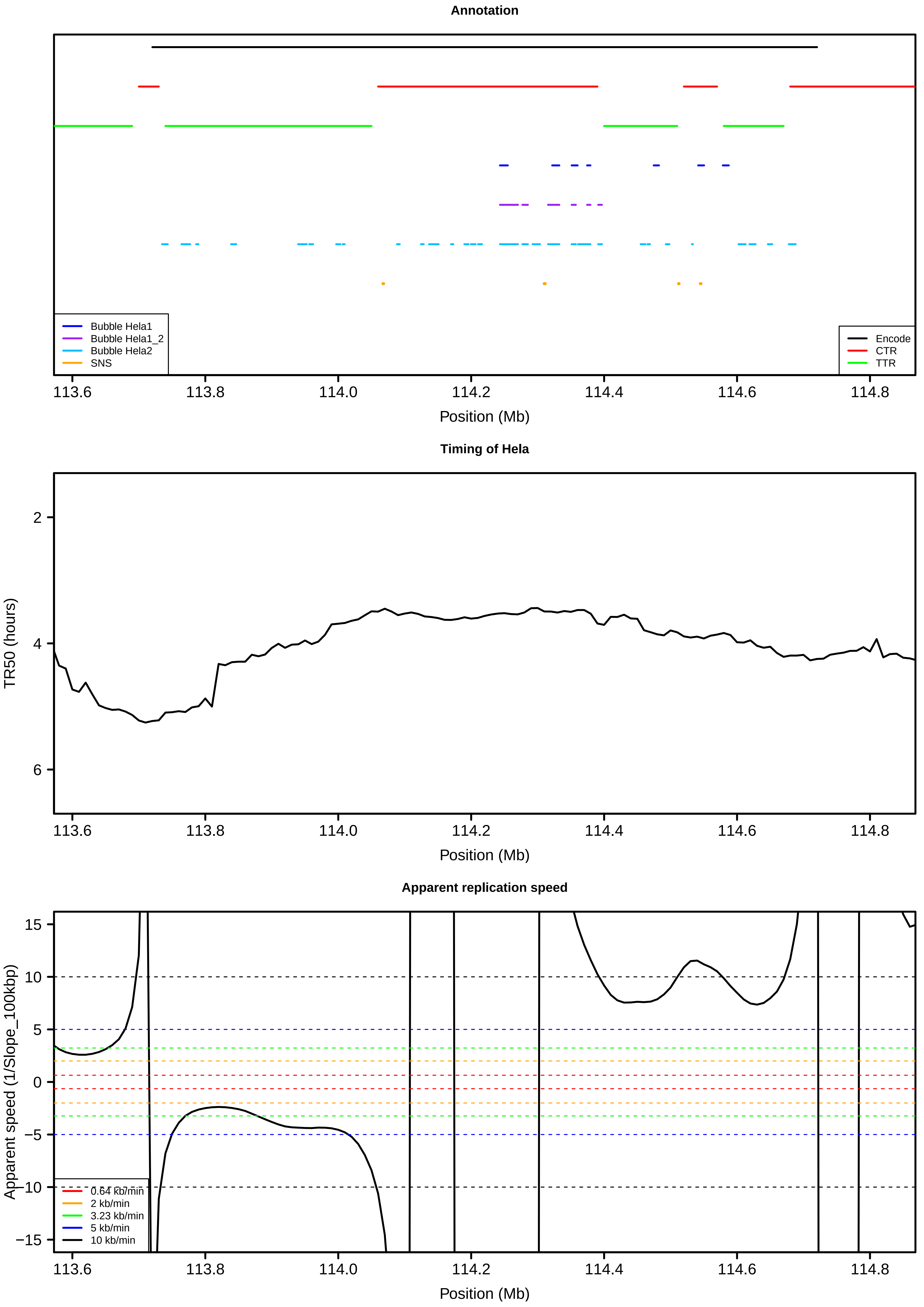

Replication Timing Vs Encode Origin data, ENm001 (chr7:115597756_117475182)

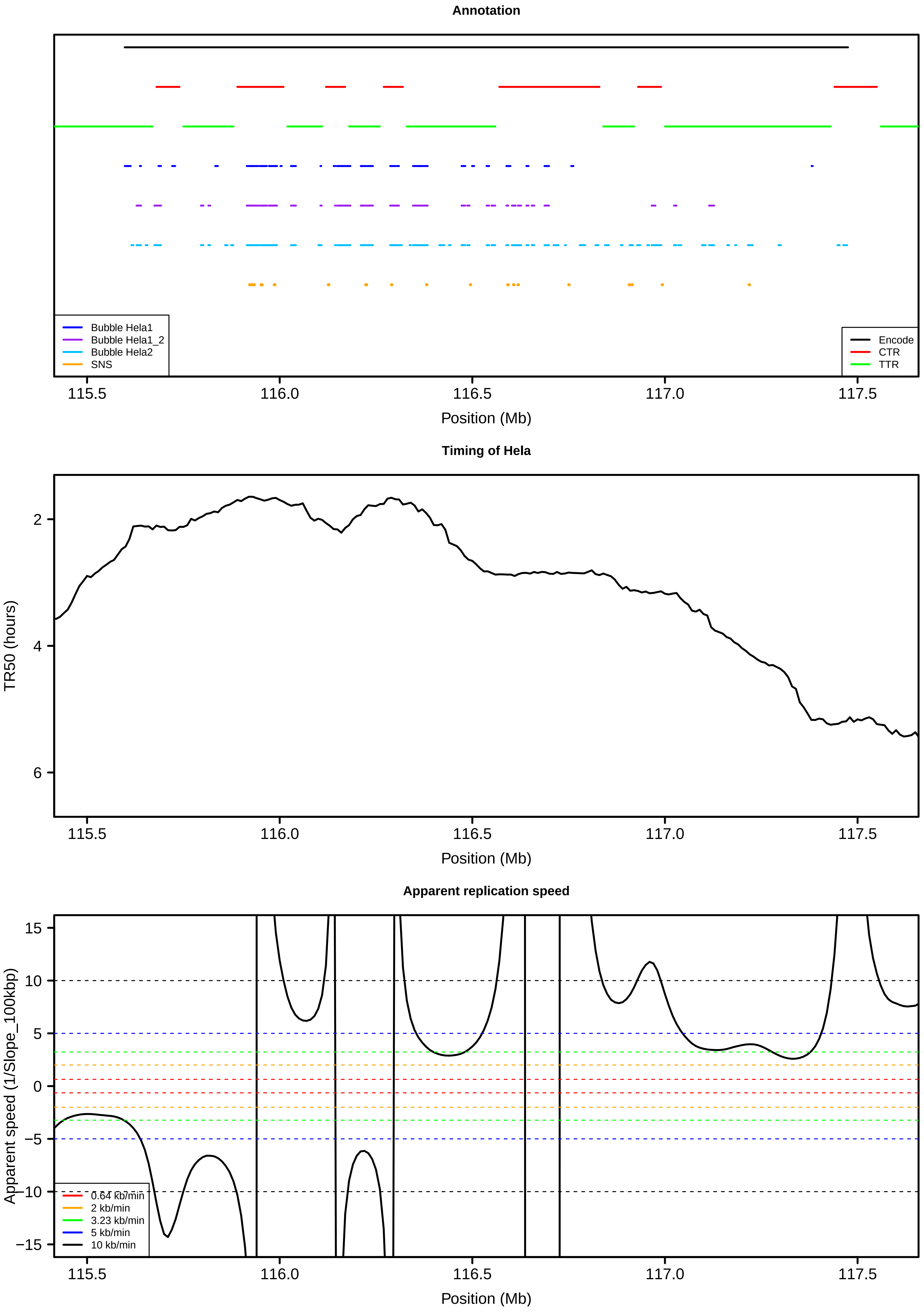

Replication Timing Vs Encode Origin data, ENm014 (chr7:125865891_127029088)

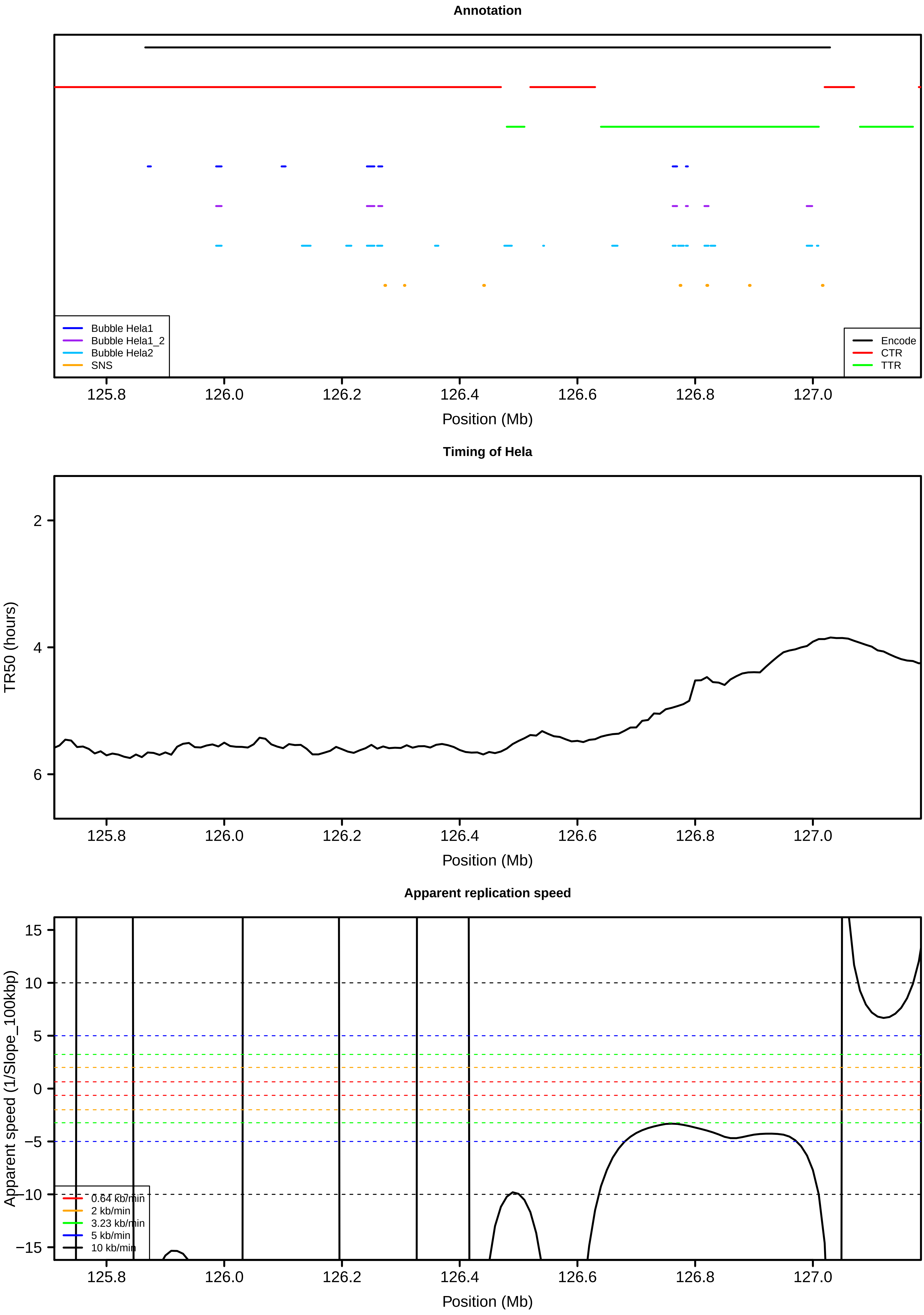

Replication Timing Vs Encode Origin data, ENr321 (chr8:118882220_119382220)

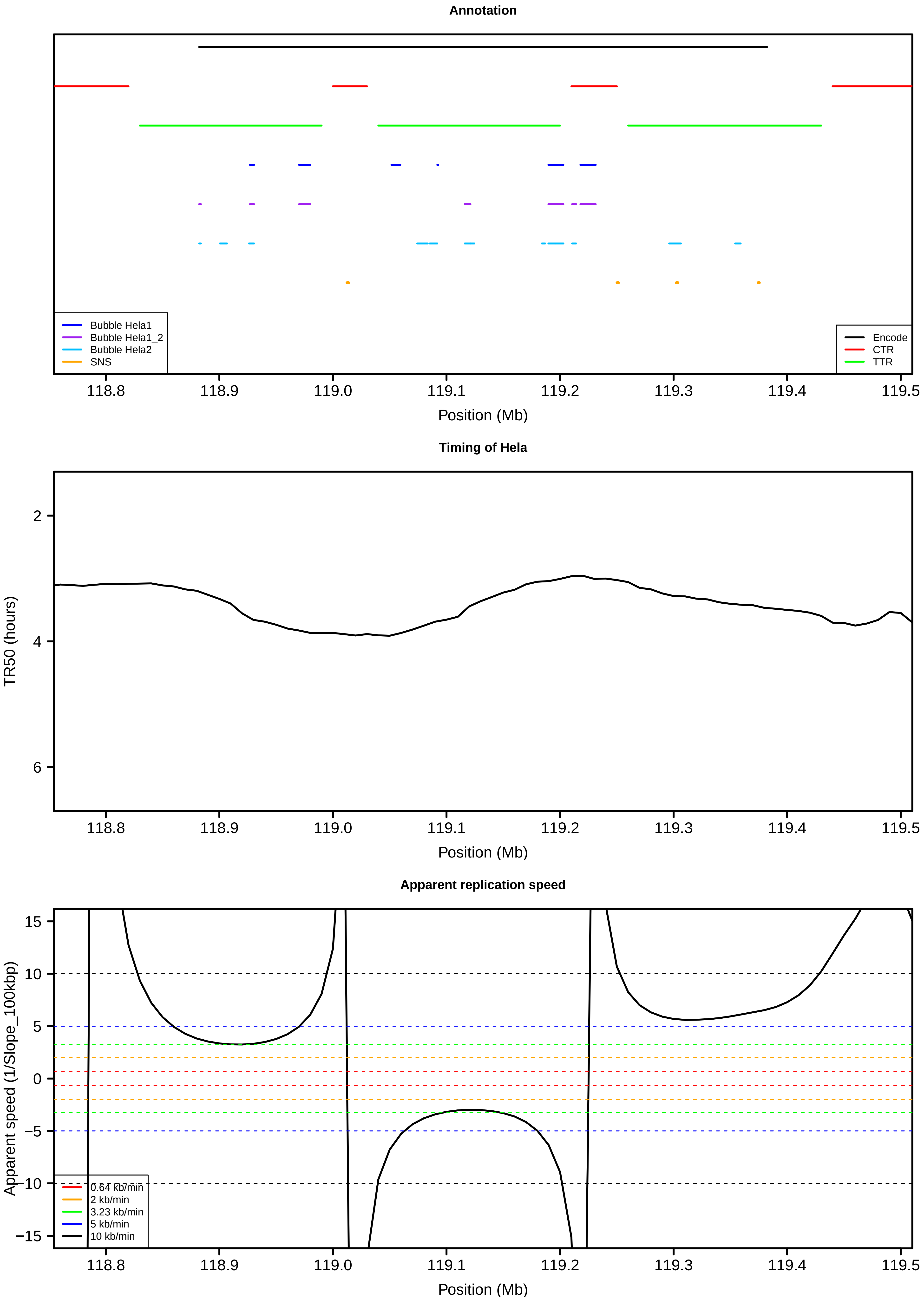

Replication Timing Vs Encode Origin data, ENr232 (chr9:130725122_131225122)

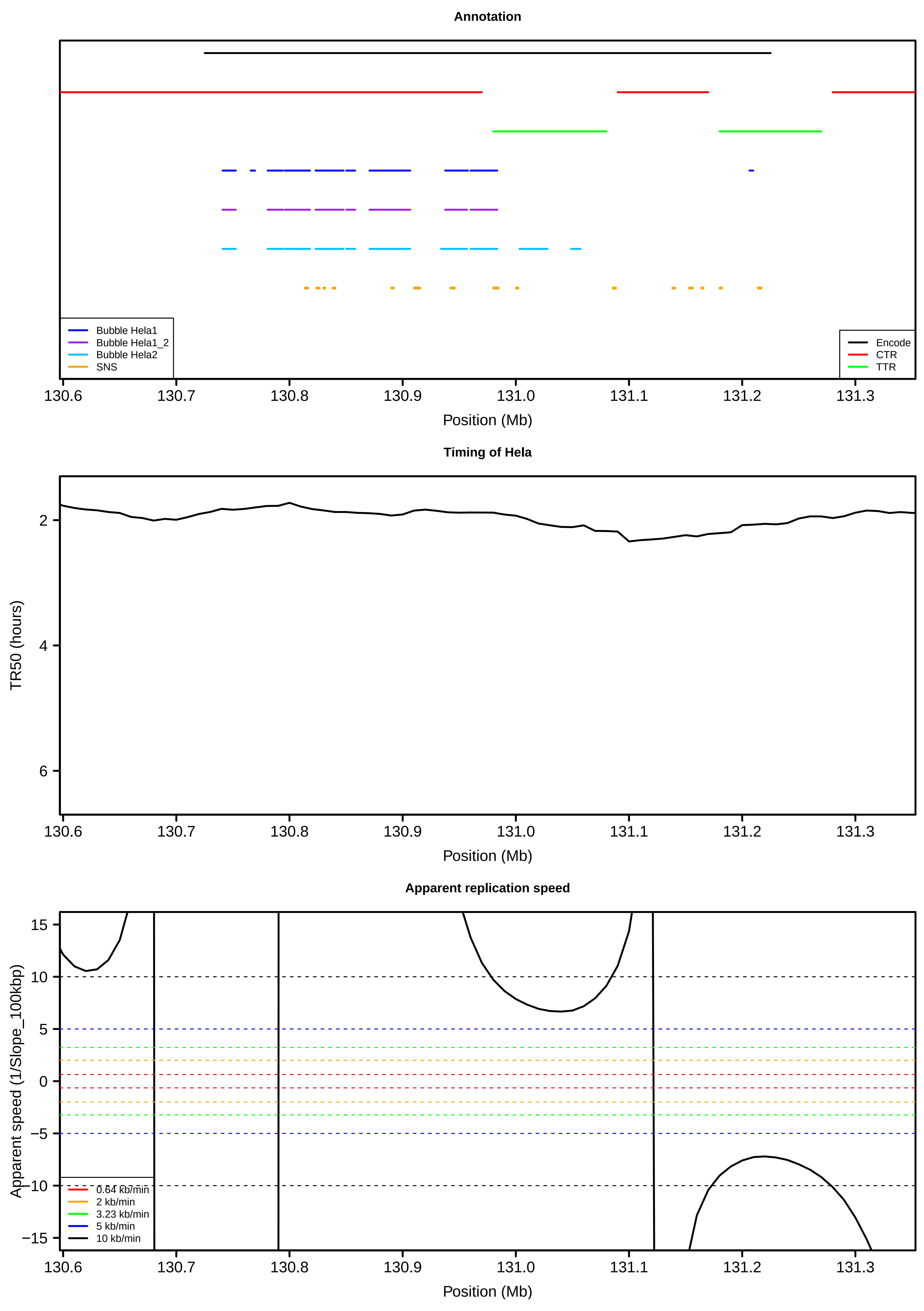

Replication Timing Vs Encode Origin data, ENr114 (chr10:55153818_55653818)

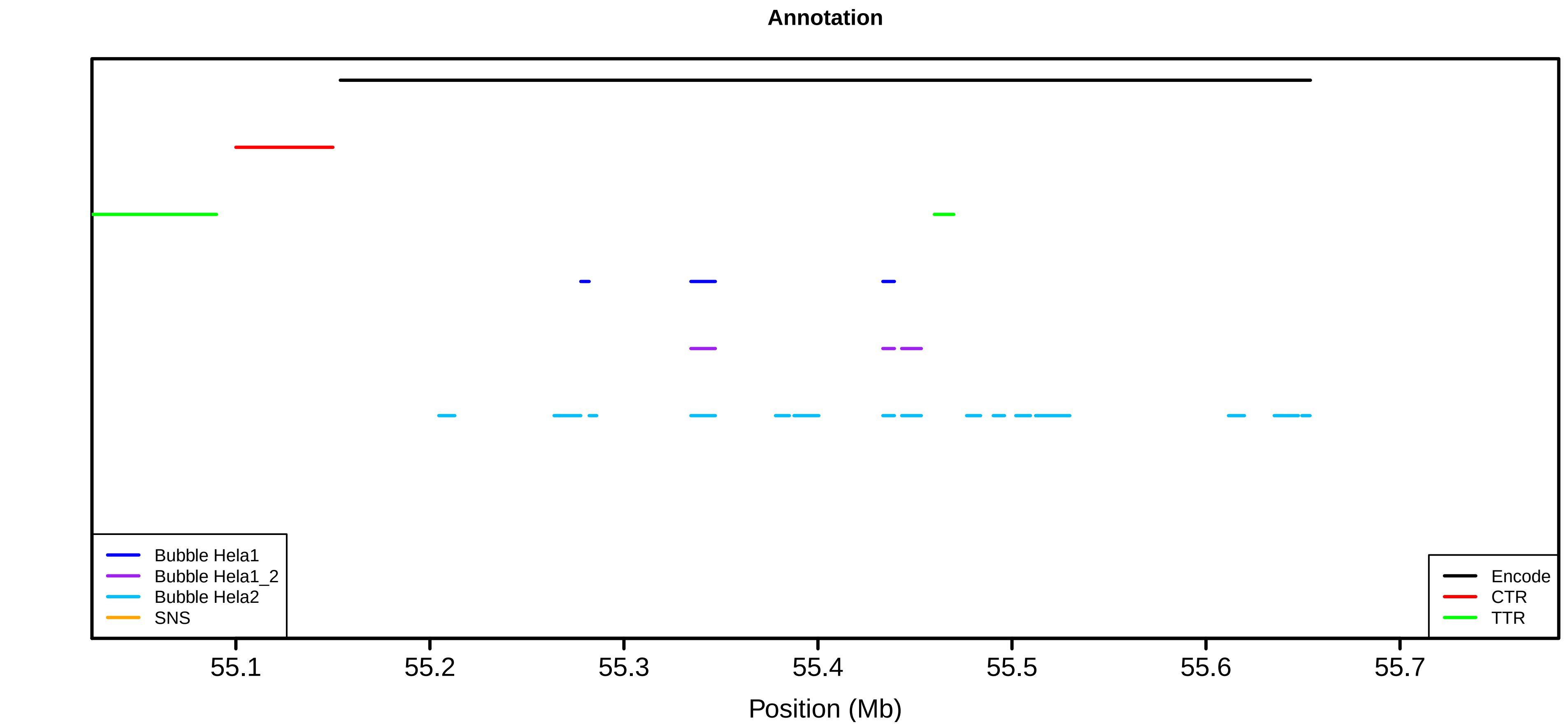

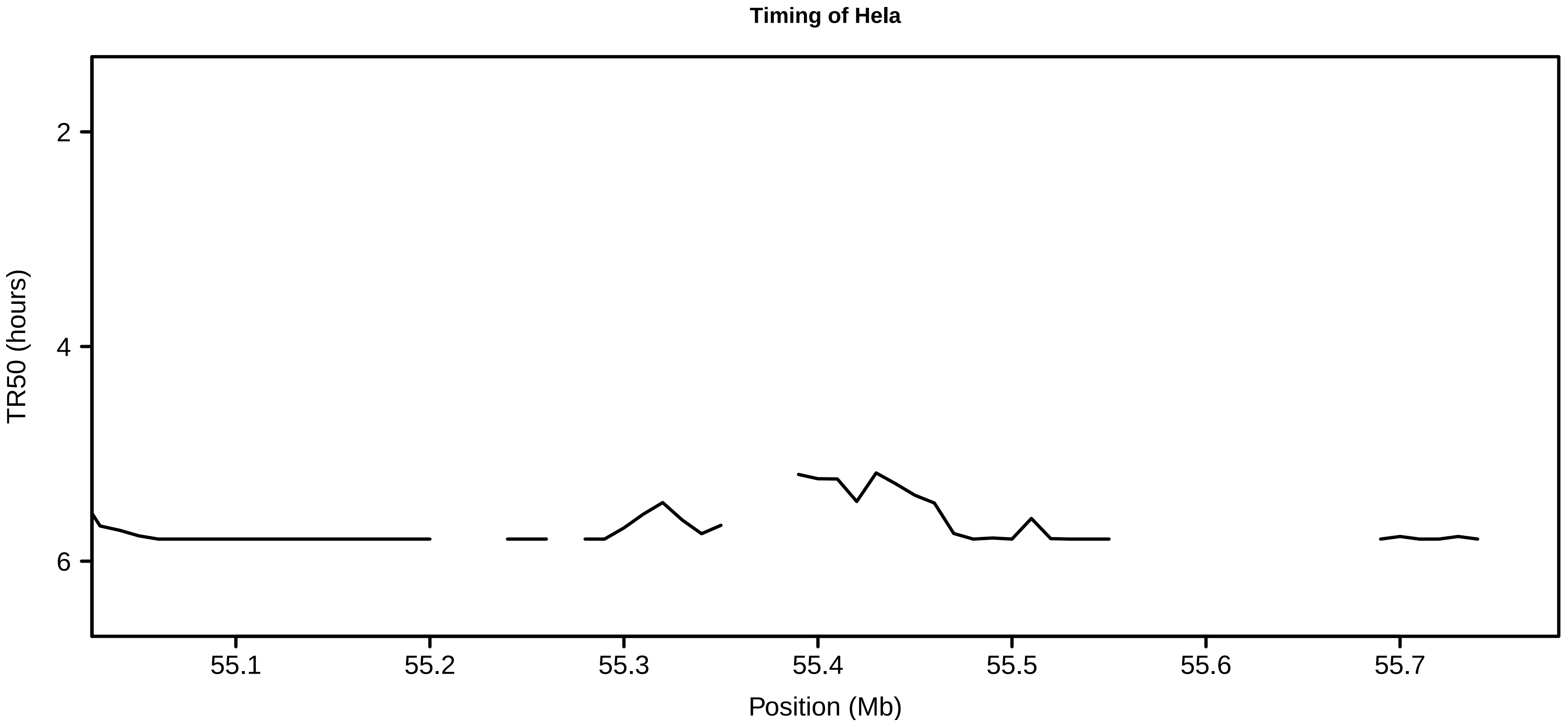

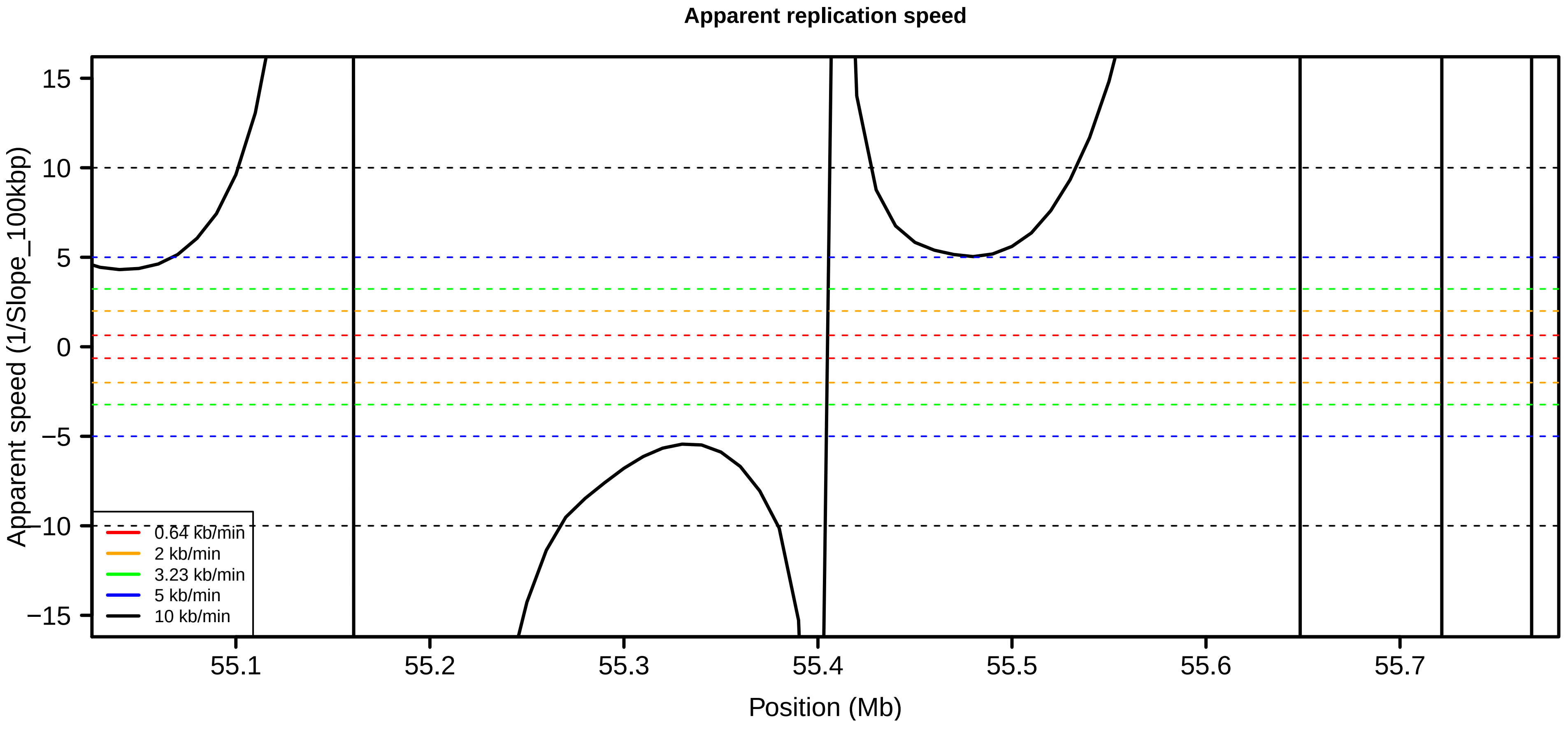

Replication Timing Vs Encode Origin data, ENm011 (chr11:1699991_2306039)

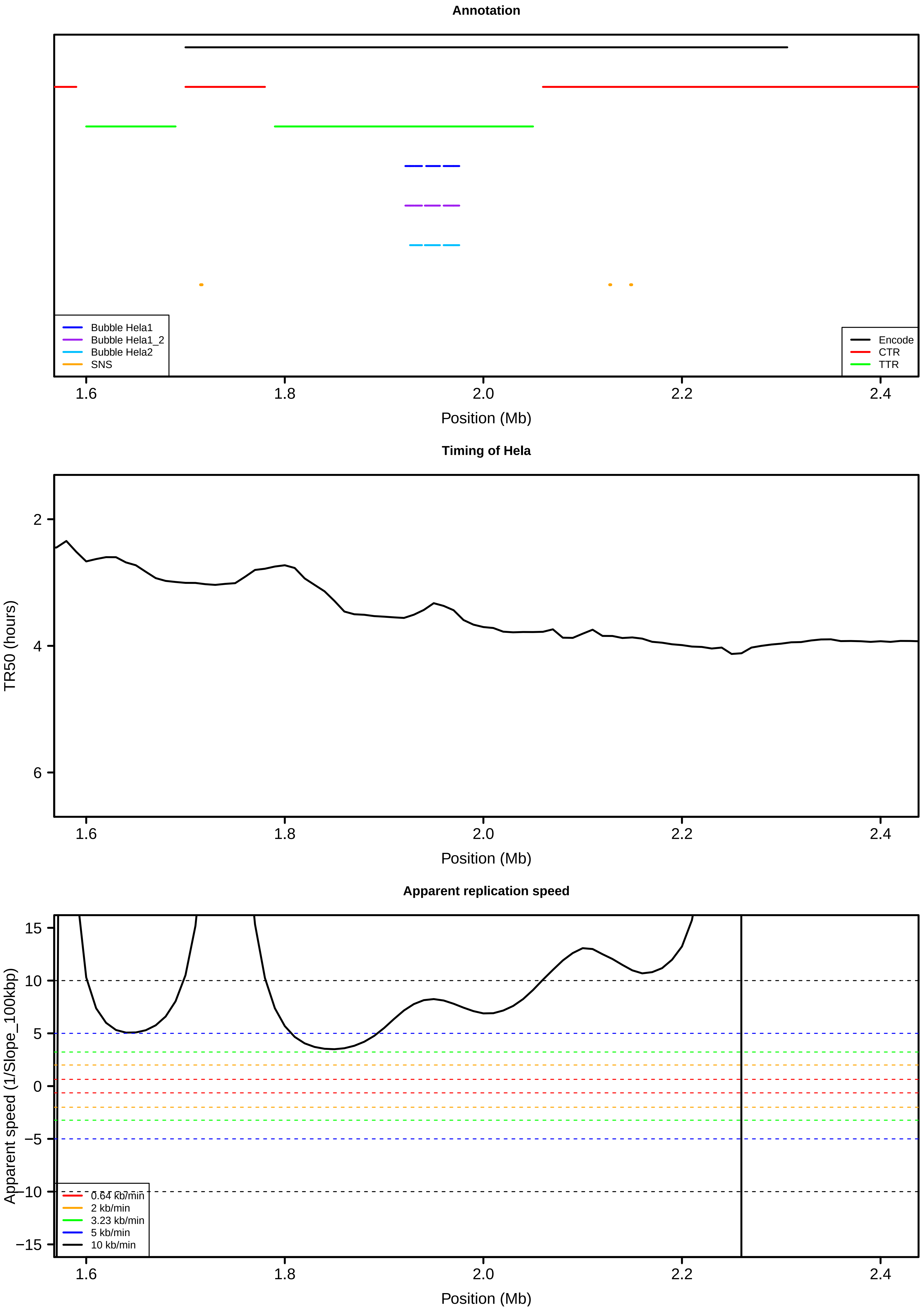

Replication Timing Vs Encode Origin data, ENm009 (chr11:4730995_5732587)

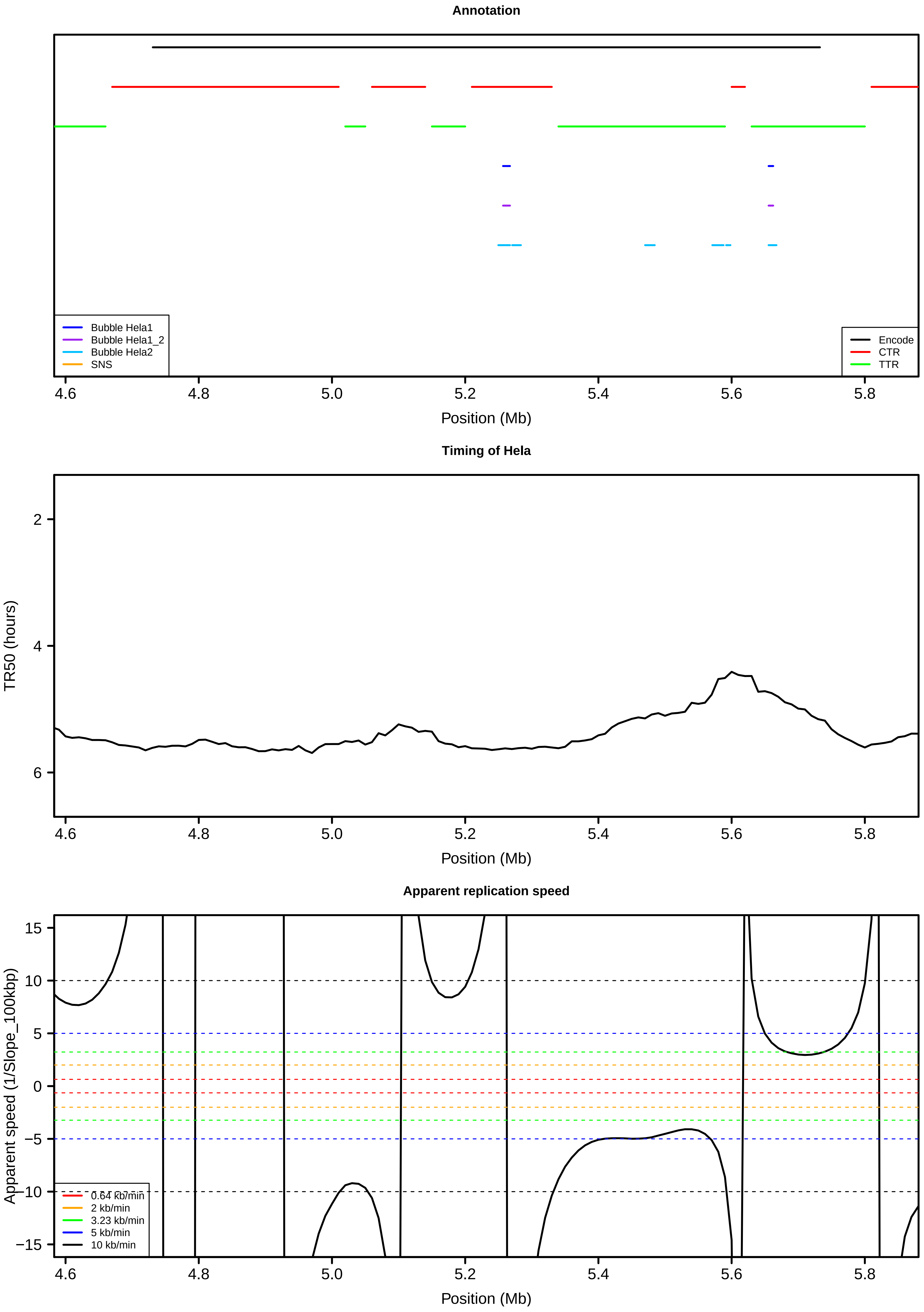

Replication Timing Vs Encode Origin data, ENr332 (chr11:63940888_64440888)

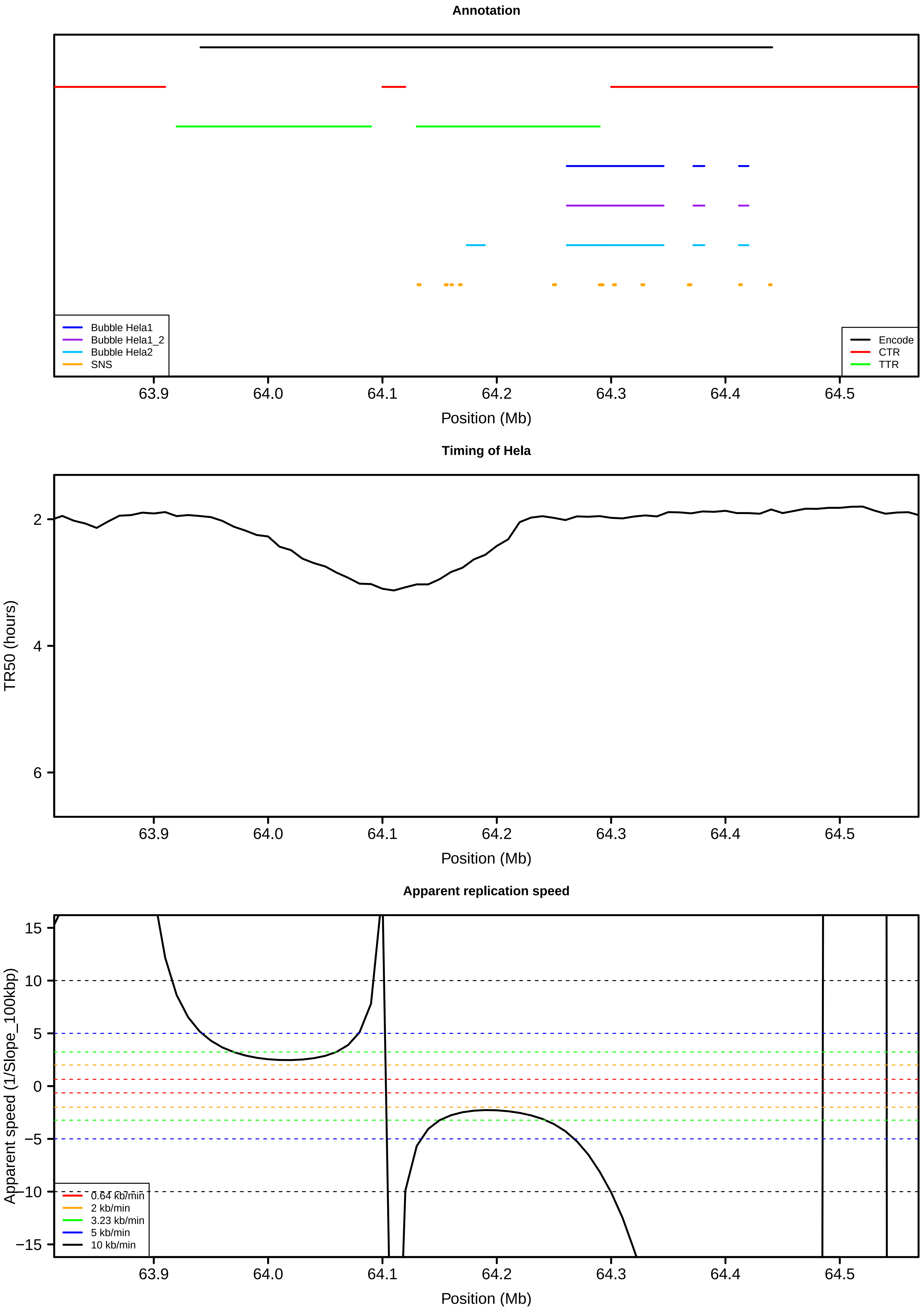

Replication Timing Vs Encode Origin data, ENm003 (chr11:115962315_116462315)

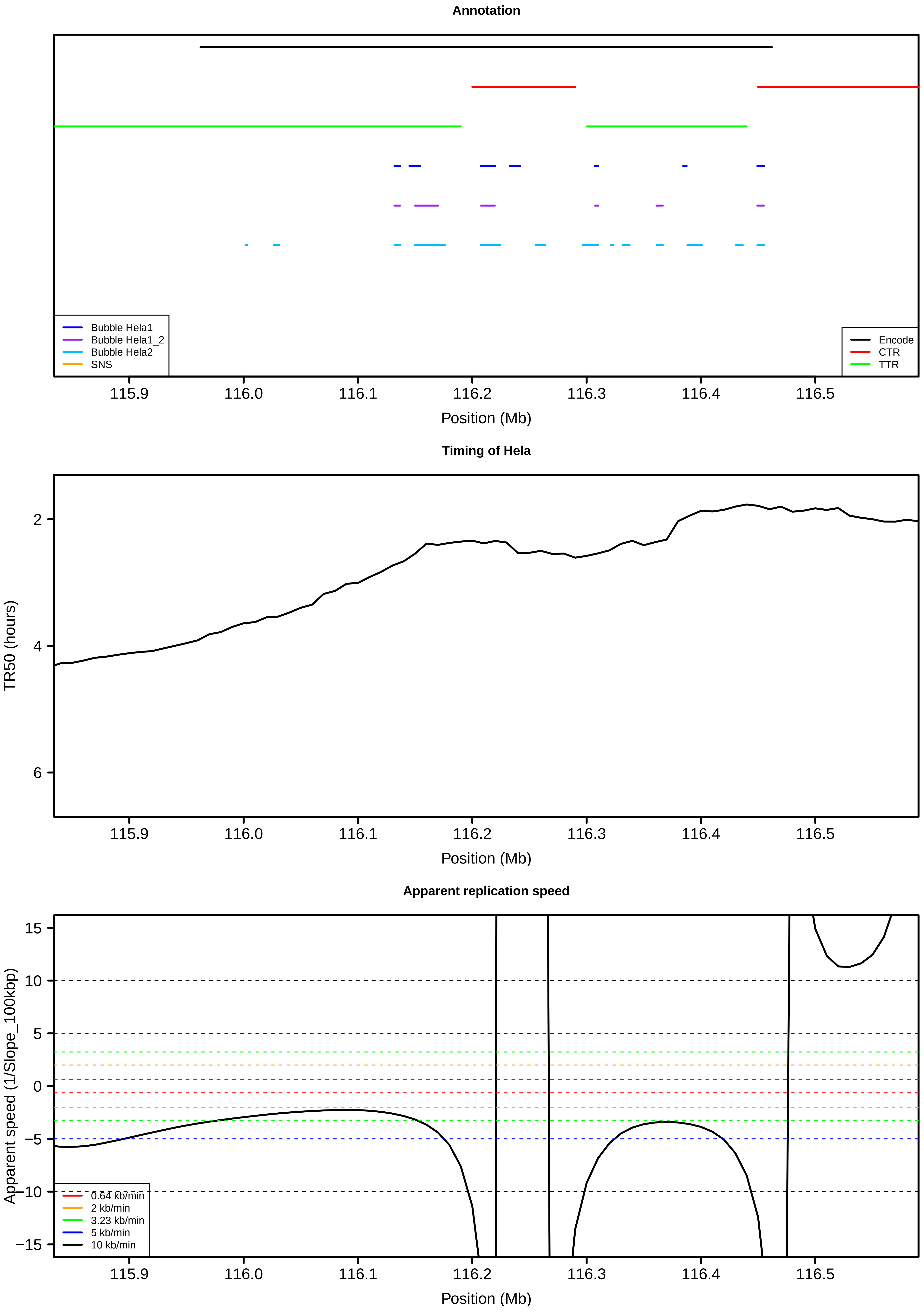

Replication Timing Vs Encode Origin data, ENr312 (chr11:130604797_131104797)

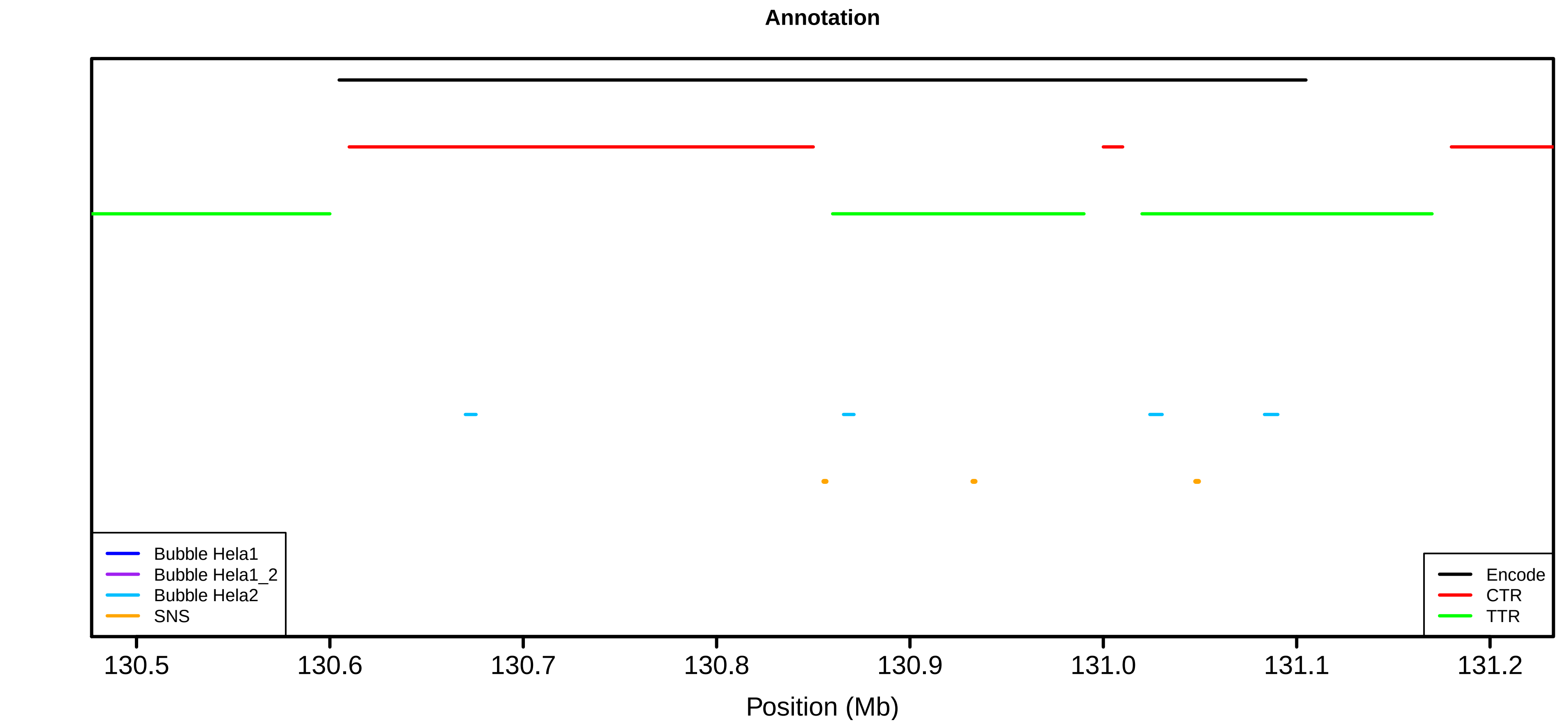

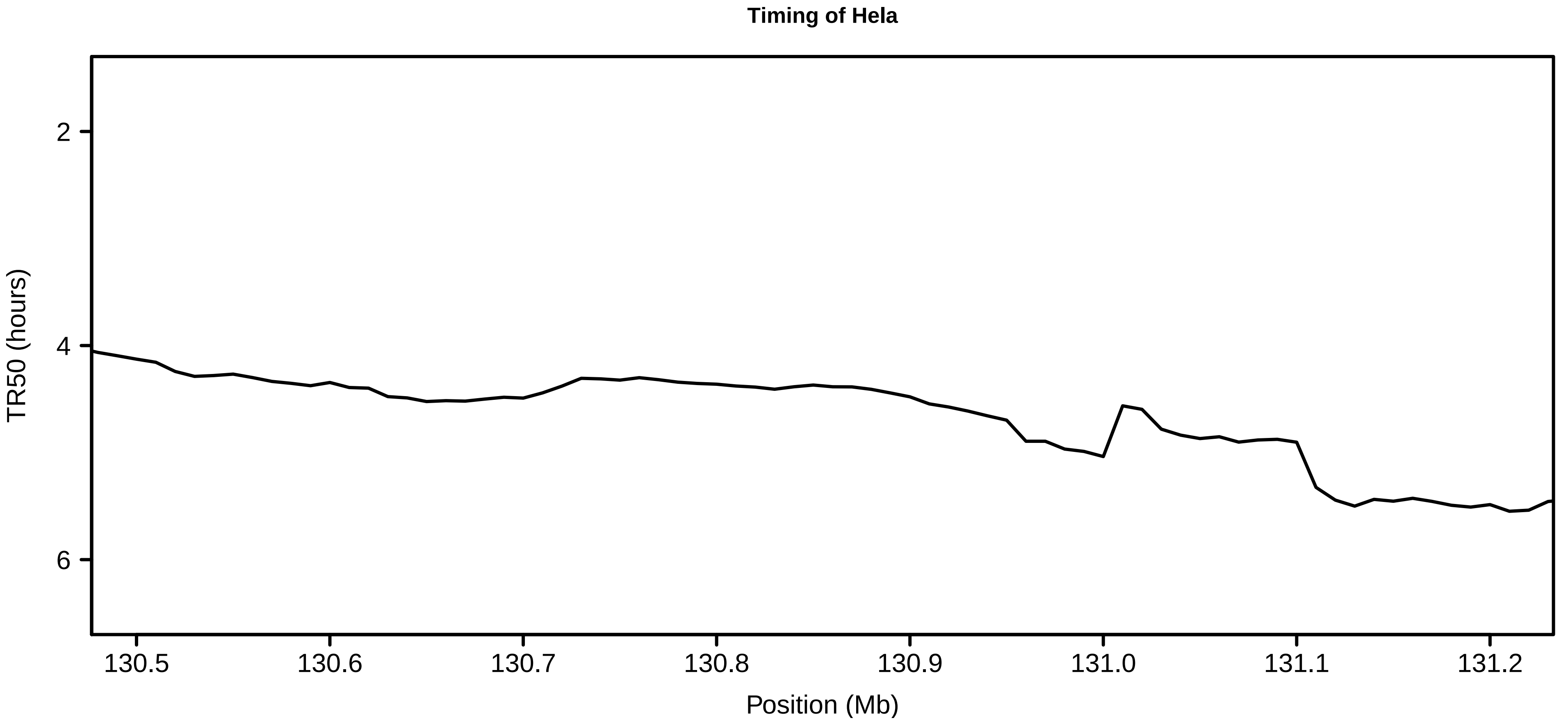

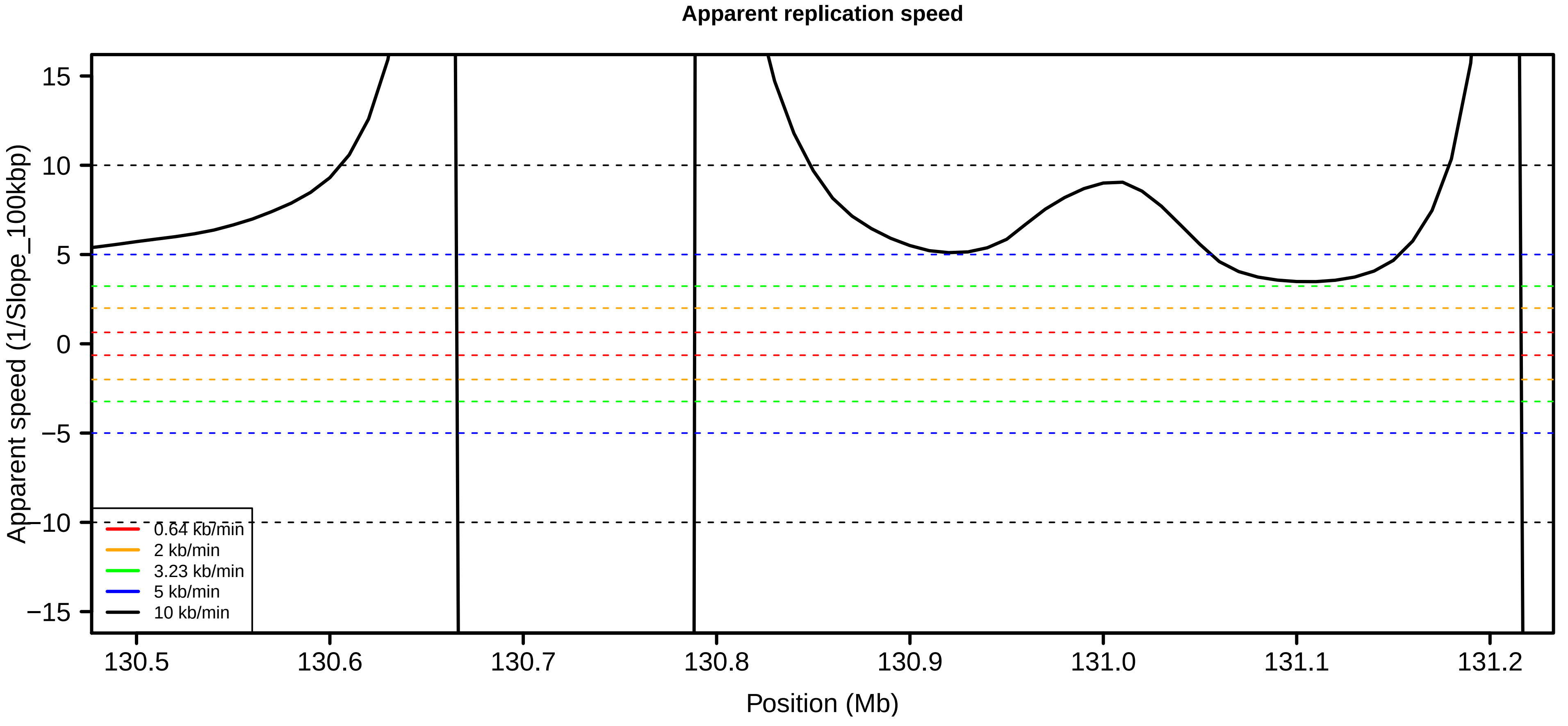

Replication Timing Vs Encode Origin data, ENr123 (chr12:38626476_39126476)

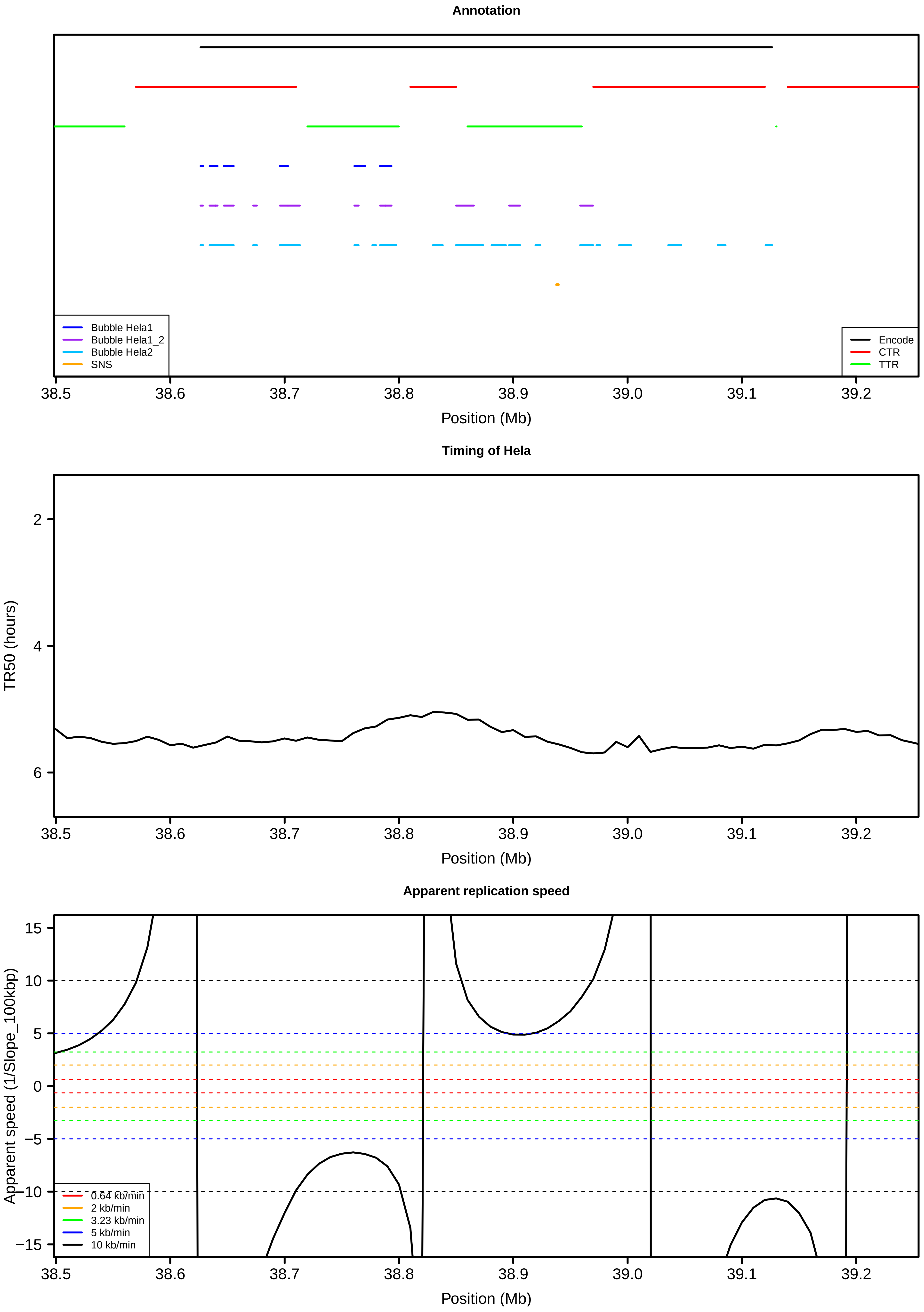

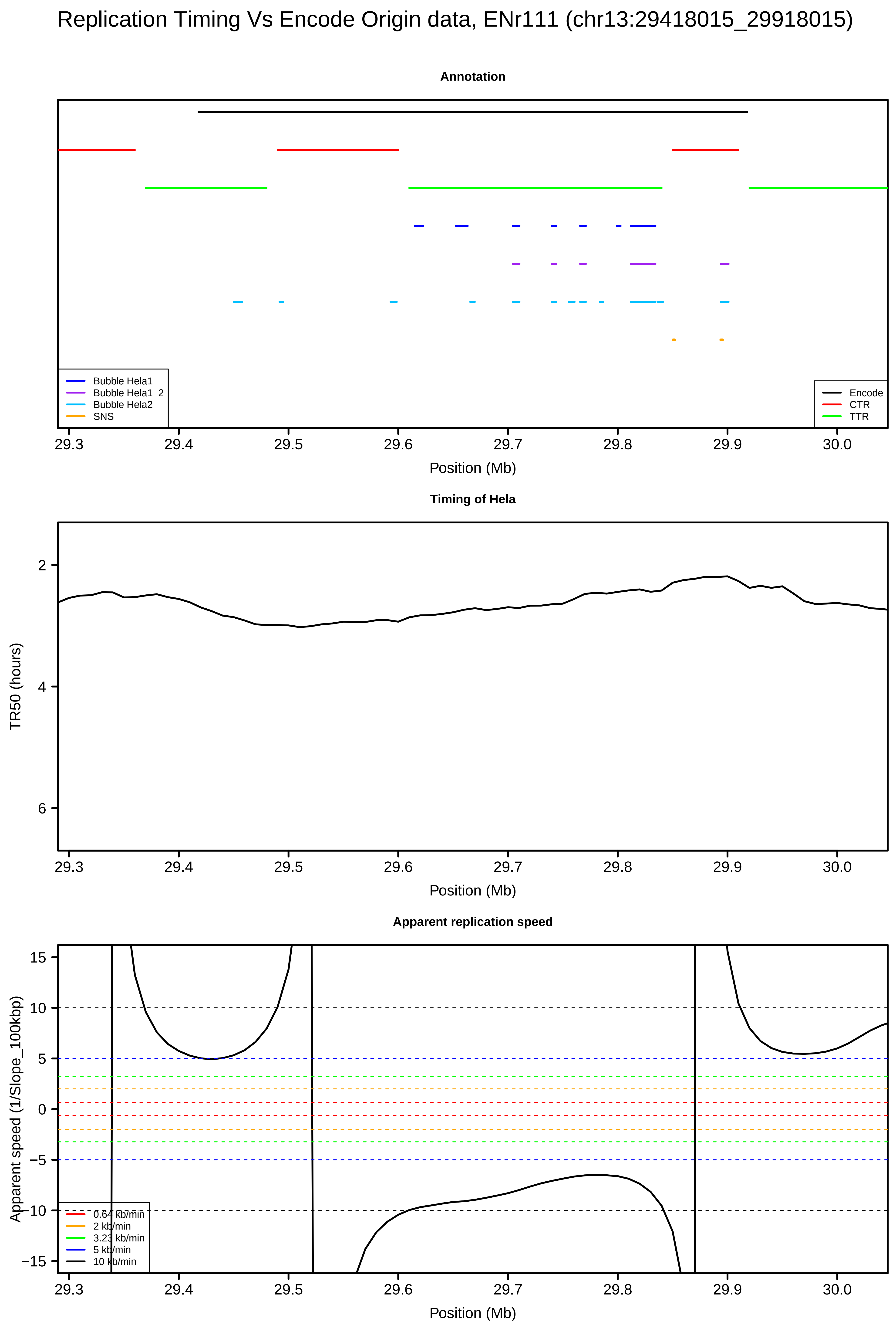

Replication Timing Vs Encode Origin data, ENr111 (chr13:29418015_29918015)
Annotation
Bubble Hela1
Bubble Hela1_2
Bubble Hela2
SNS
Encode
CTR
TTR
29.3
29.4
29.5
29.6
29.7
29.8
29.9
30.0
Position (Mb)
Timing of Hela
TR50 (hours)
2
4
6
29.3
29.4
29.5
29.6
29.7
29.8
29.9
30.0
Position (Mb)
Apparent replication speed
Apparent speed (1/Slope_100kbp)
15
10
5
0
−5
−10
−15
0.64 kb/min
2 kb/min
3.23 kb/min
5 kb/min
10 kb/min
29.3
29.4
29.5
29.6
29.7
29.8
29.9
30.0
Position (Mb)

Replication Timing Vs Encode Origin data, ENr132 (chr13:112338064_112838064)

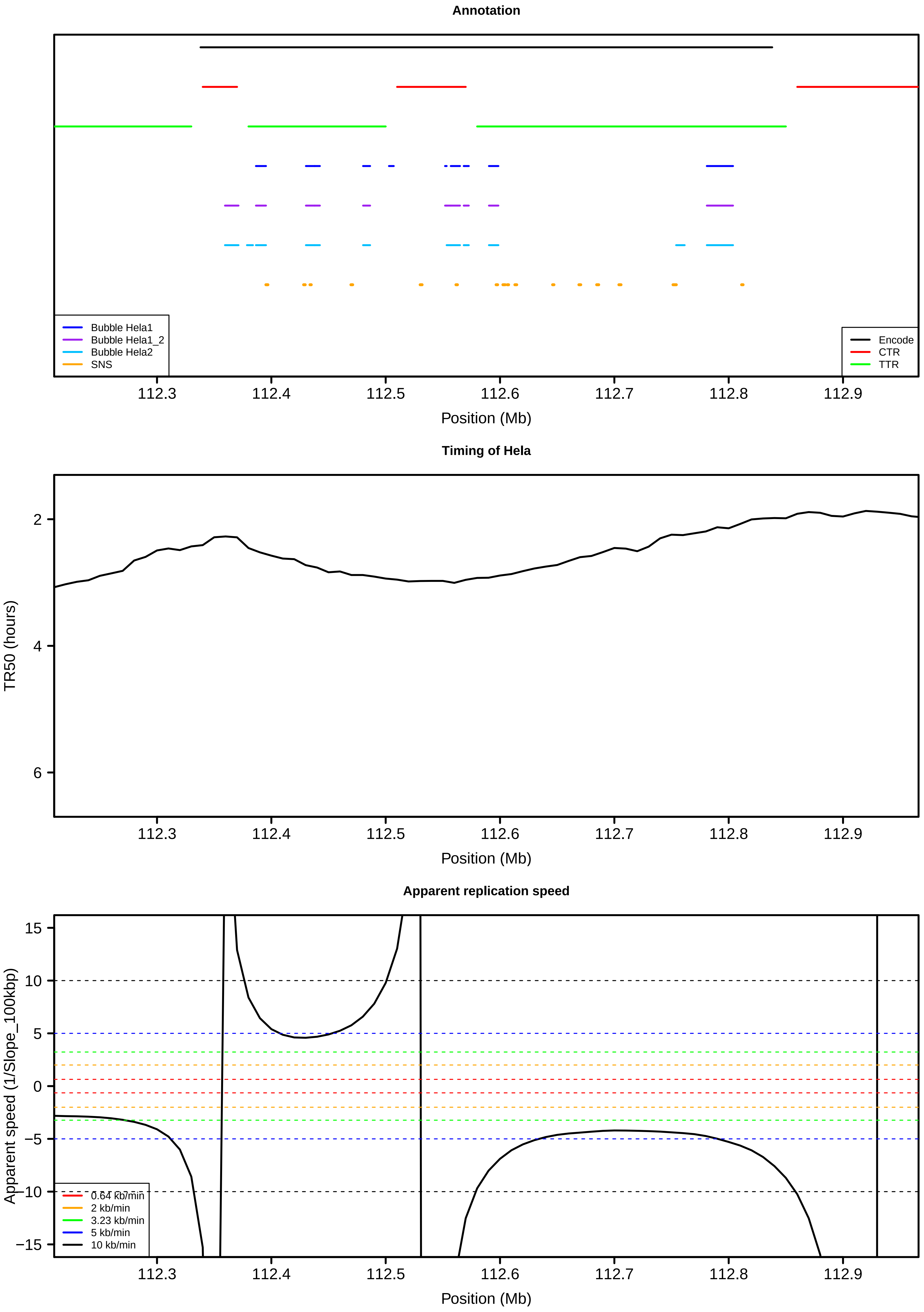

Replication Timing Vs Encode Origin data, ENr311 (chr14:52947075_53447075)

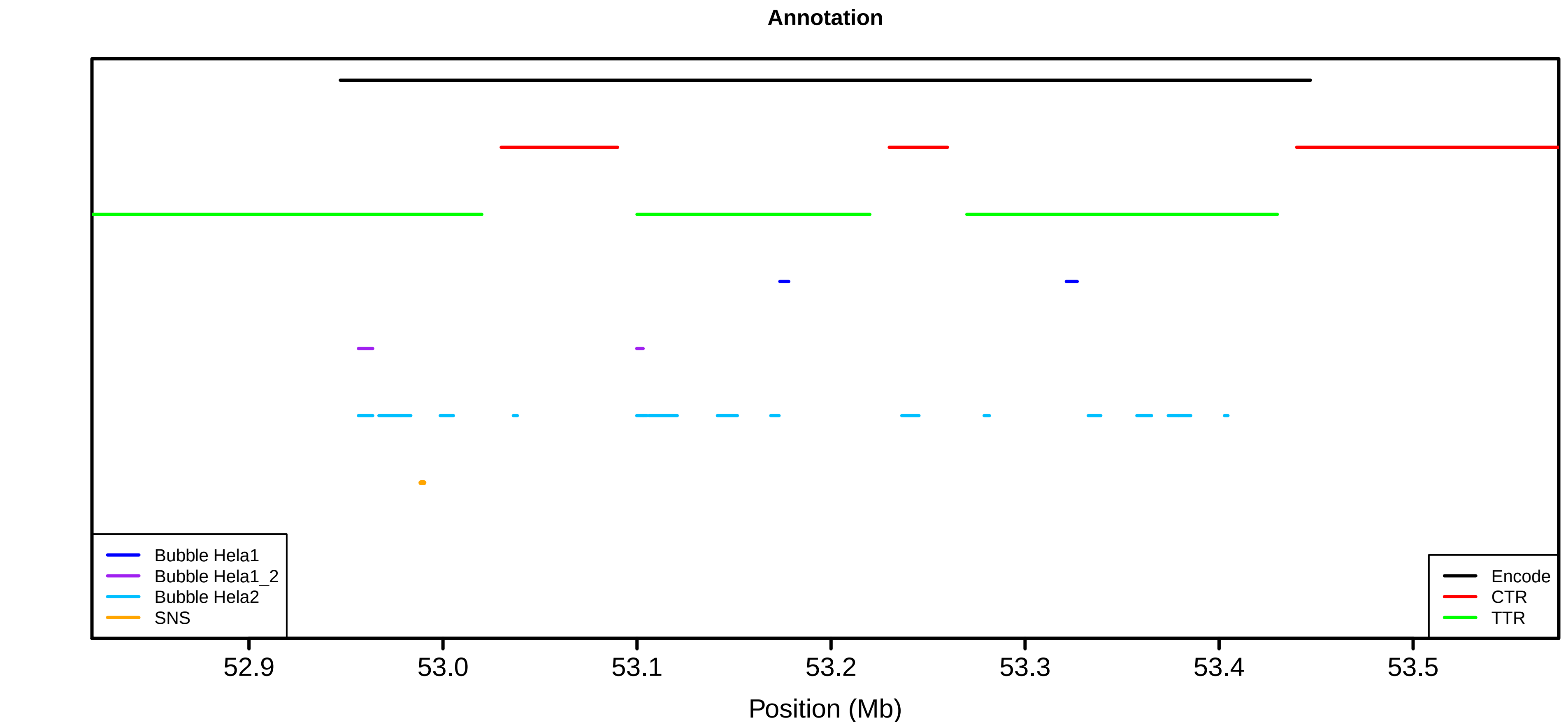

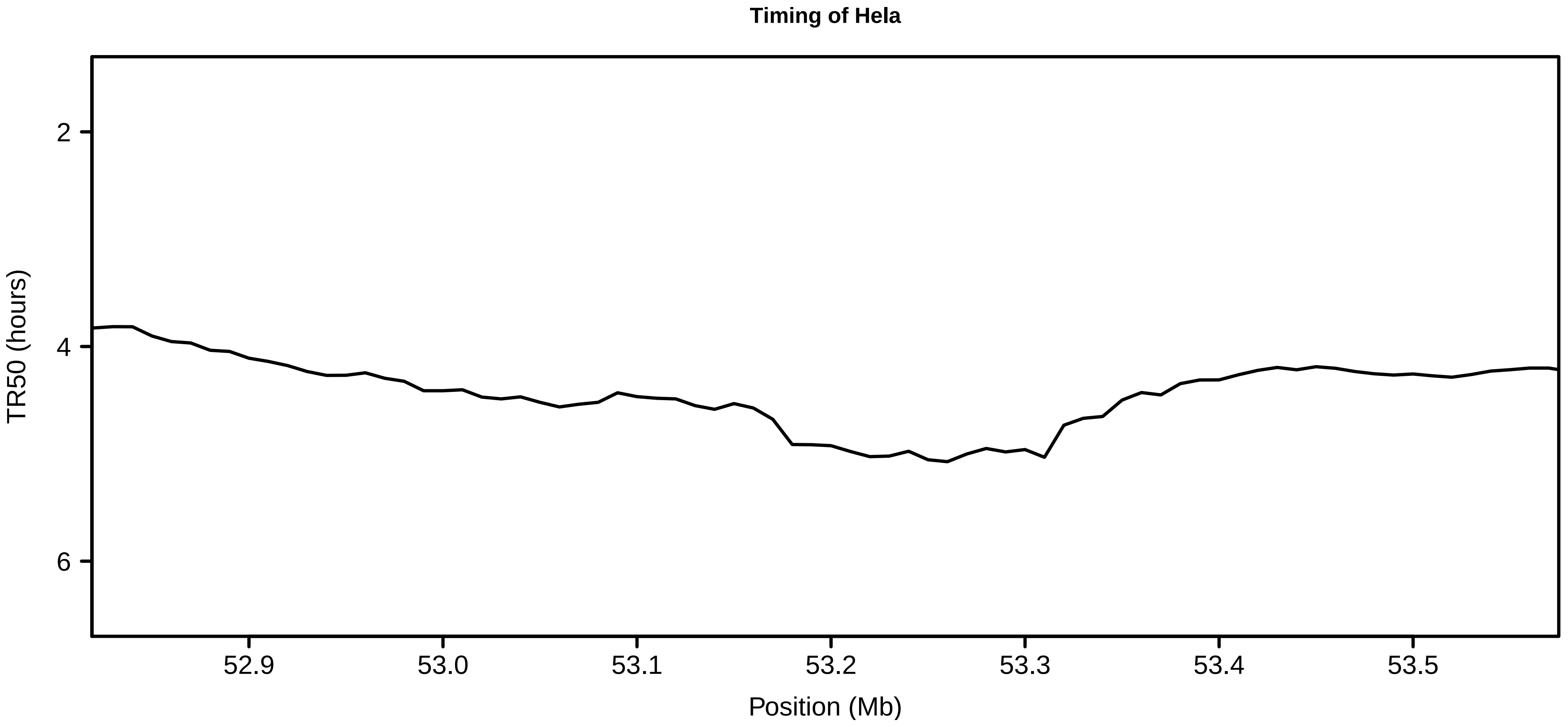

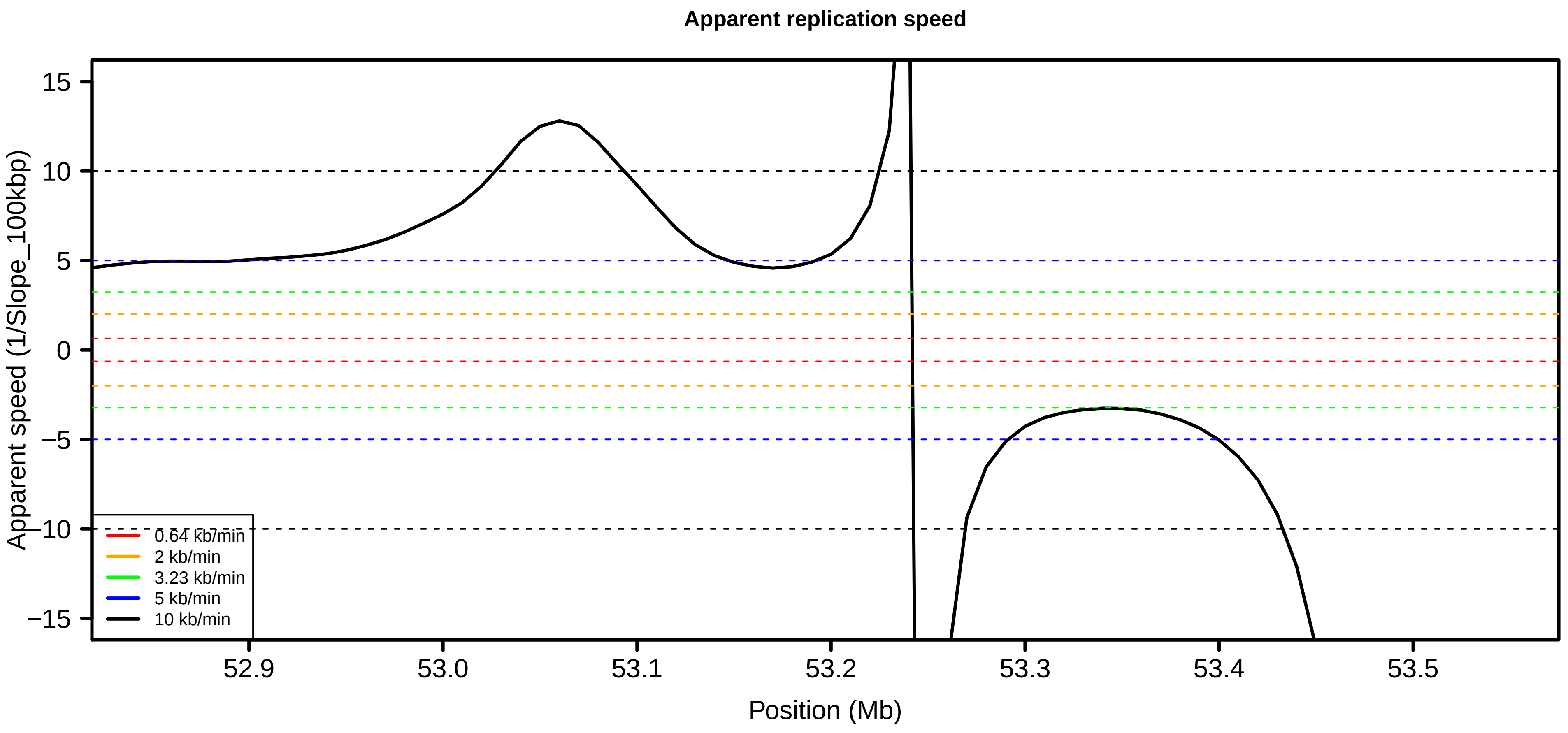

Replication Timing Vs Encode Origin data, ENr322 (chr14:98458223_98958223)

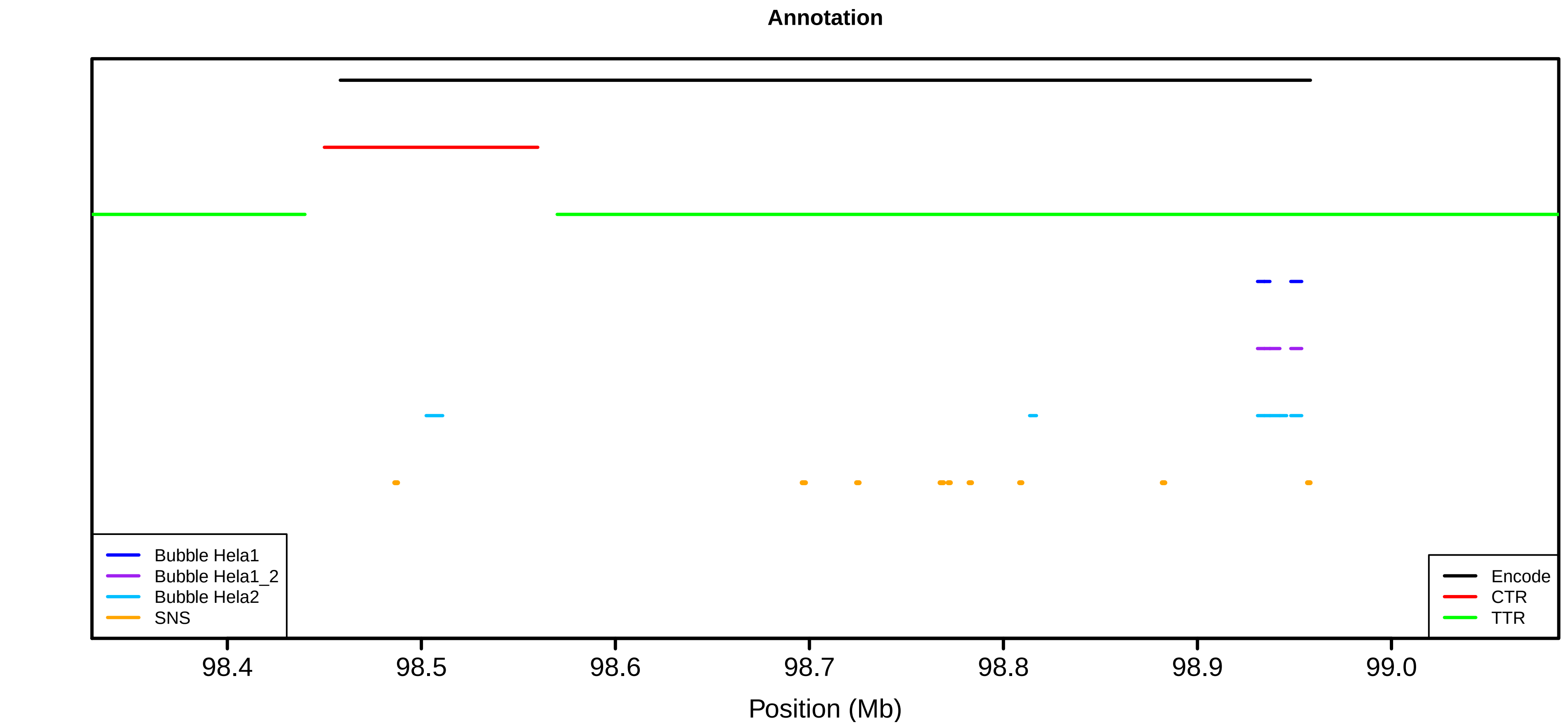

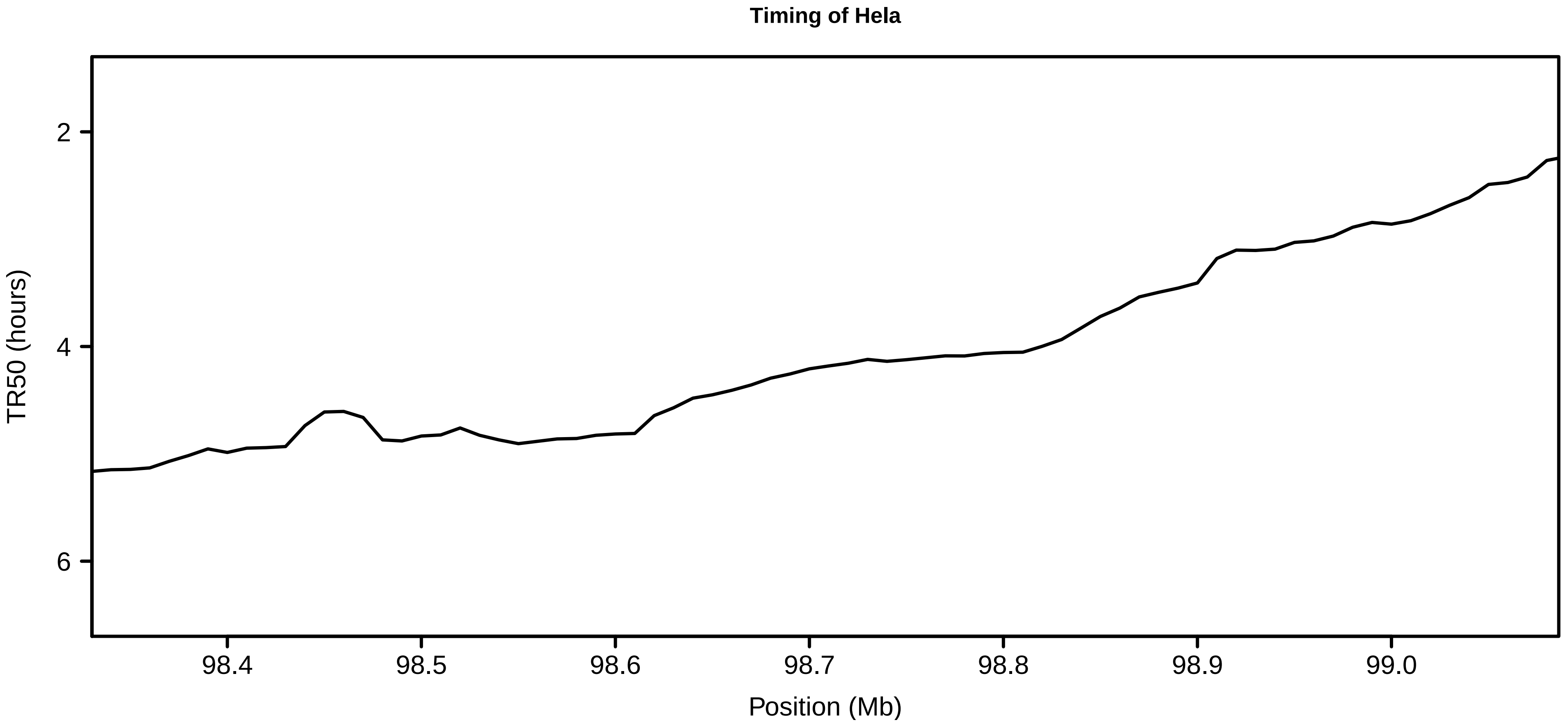

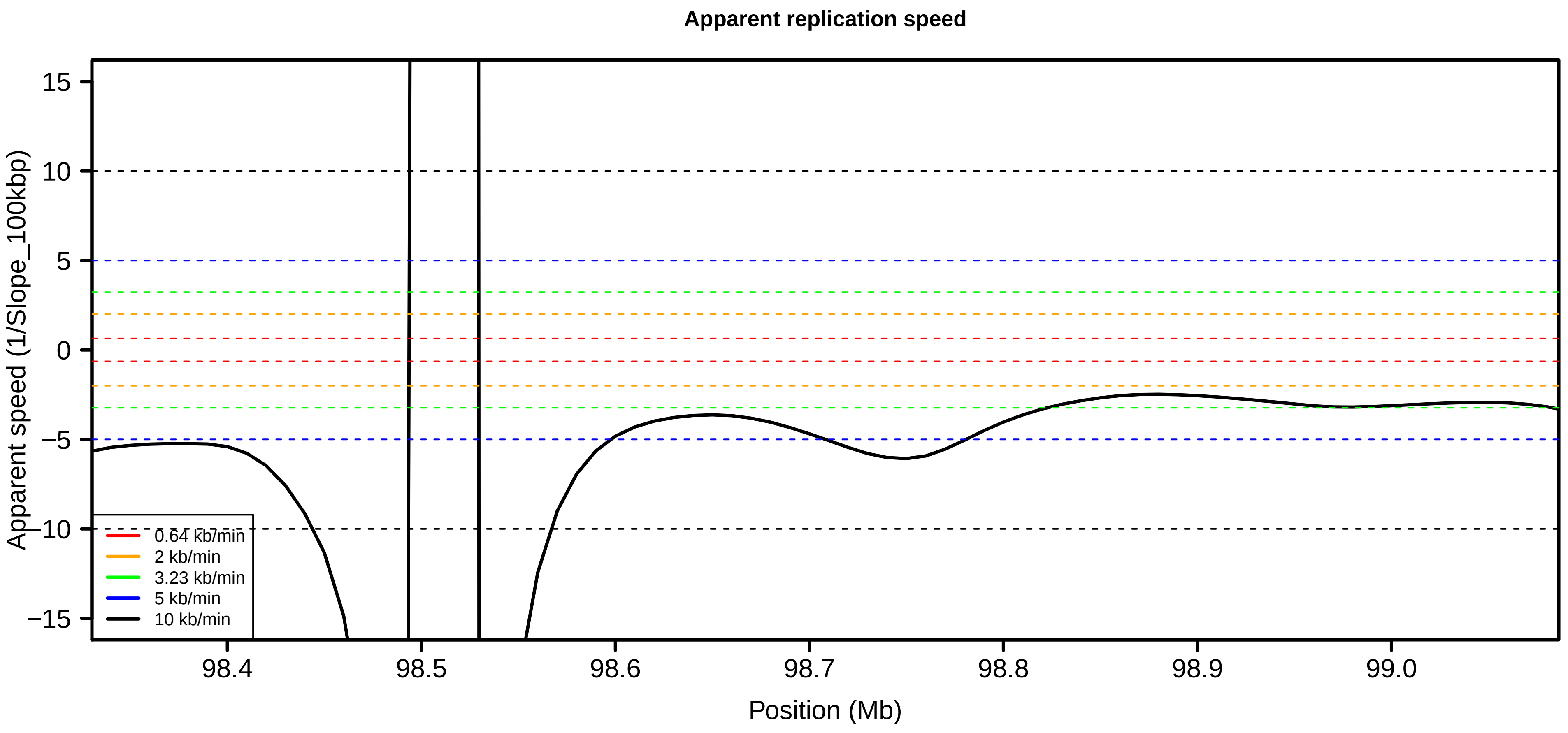

Replication Timing Vs Encode Origin data, ENr233 (chr15:41520088_42020088)

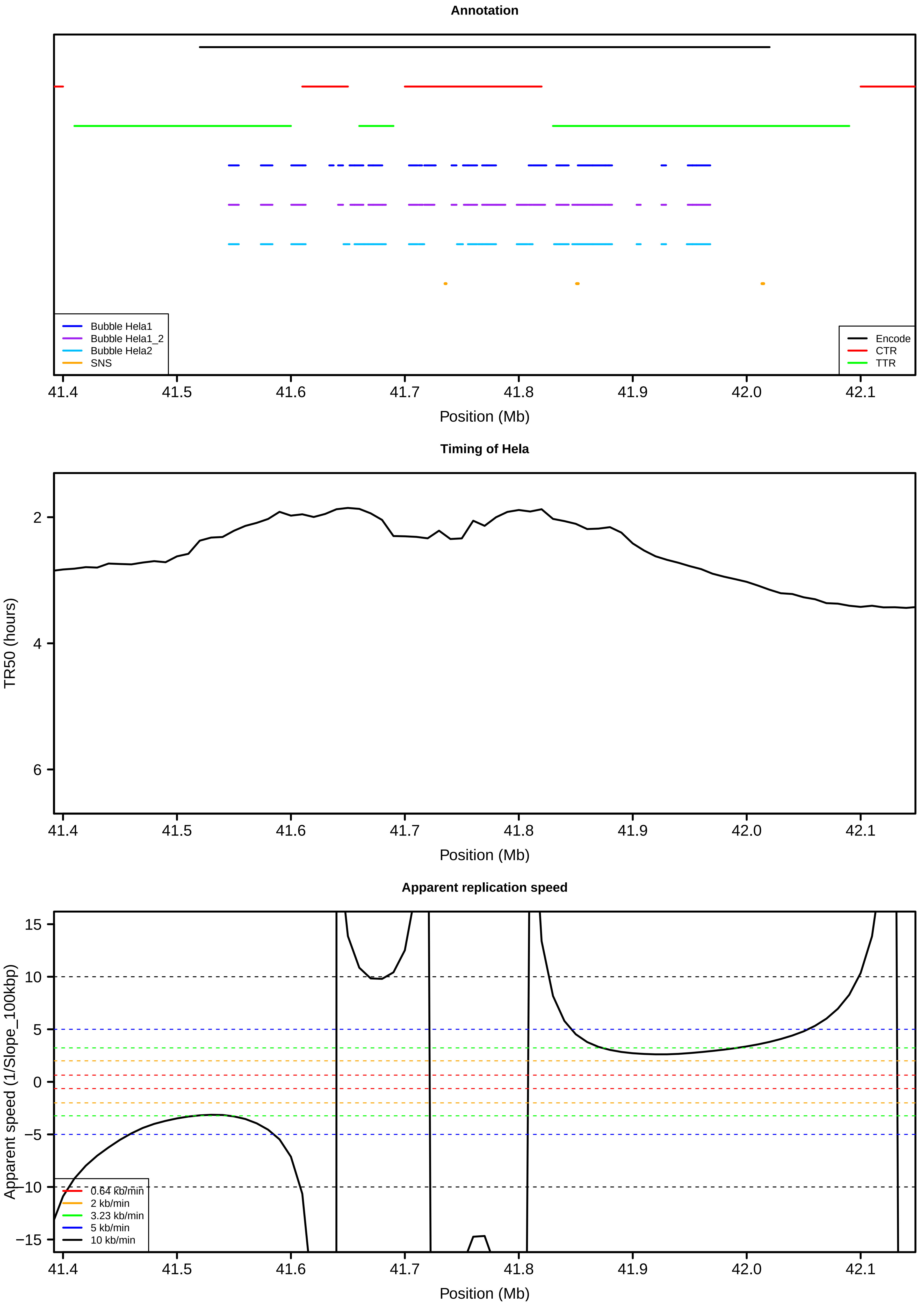

Replication Timing Vs Encode Origin data, ENm008 (chr16:0_500000)

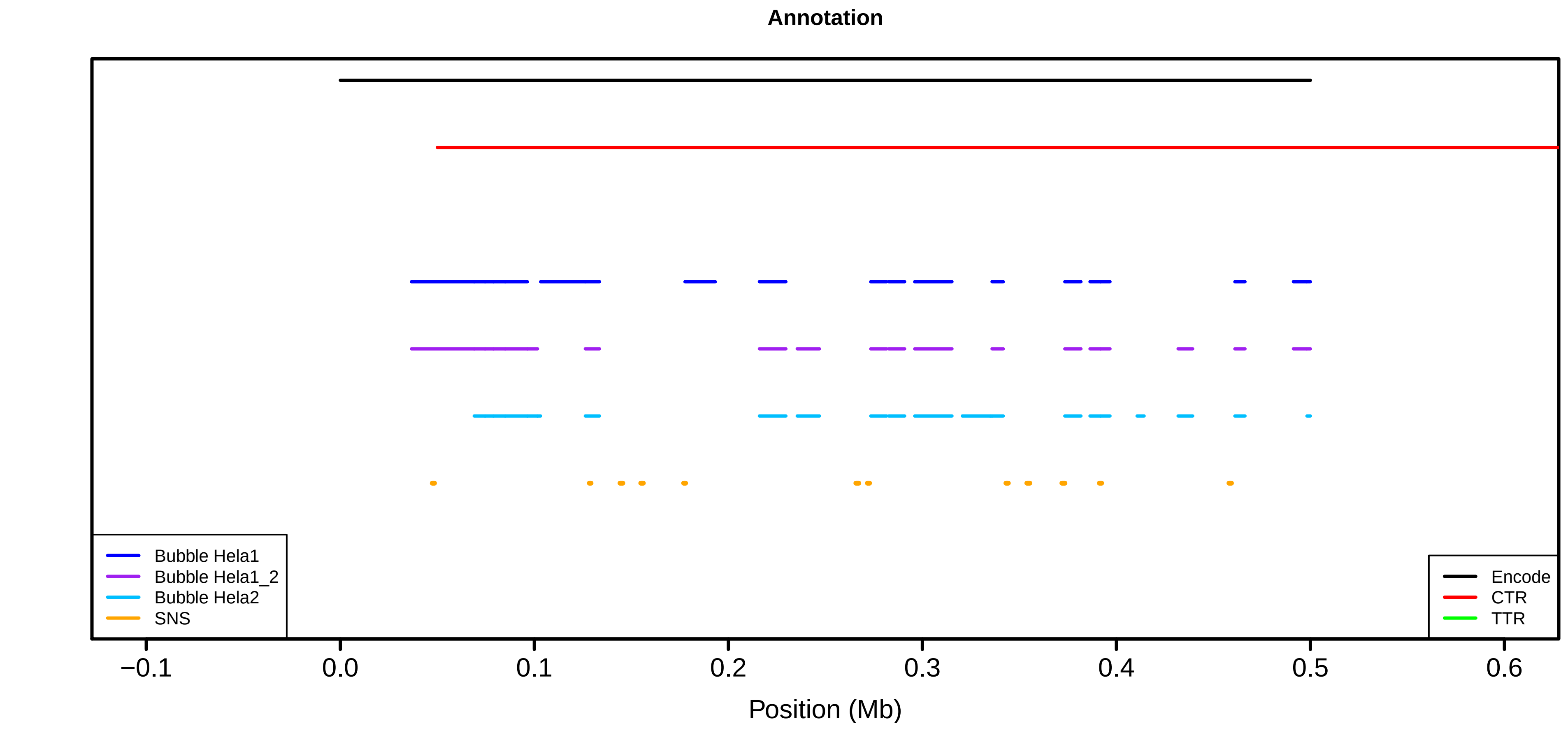

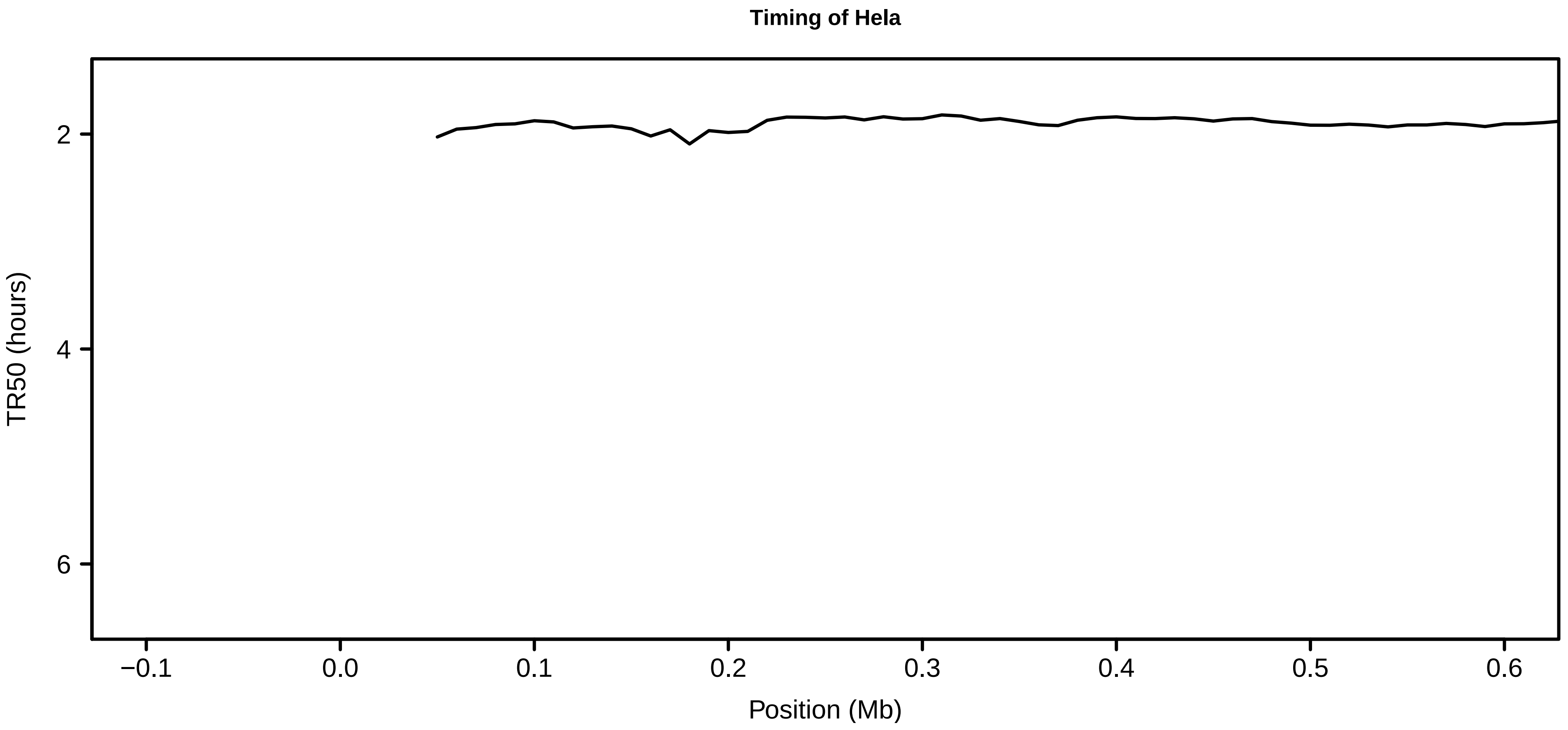

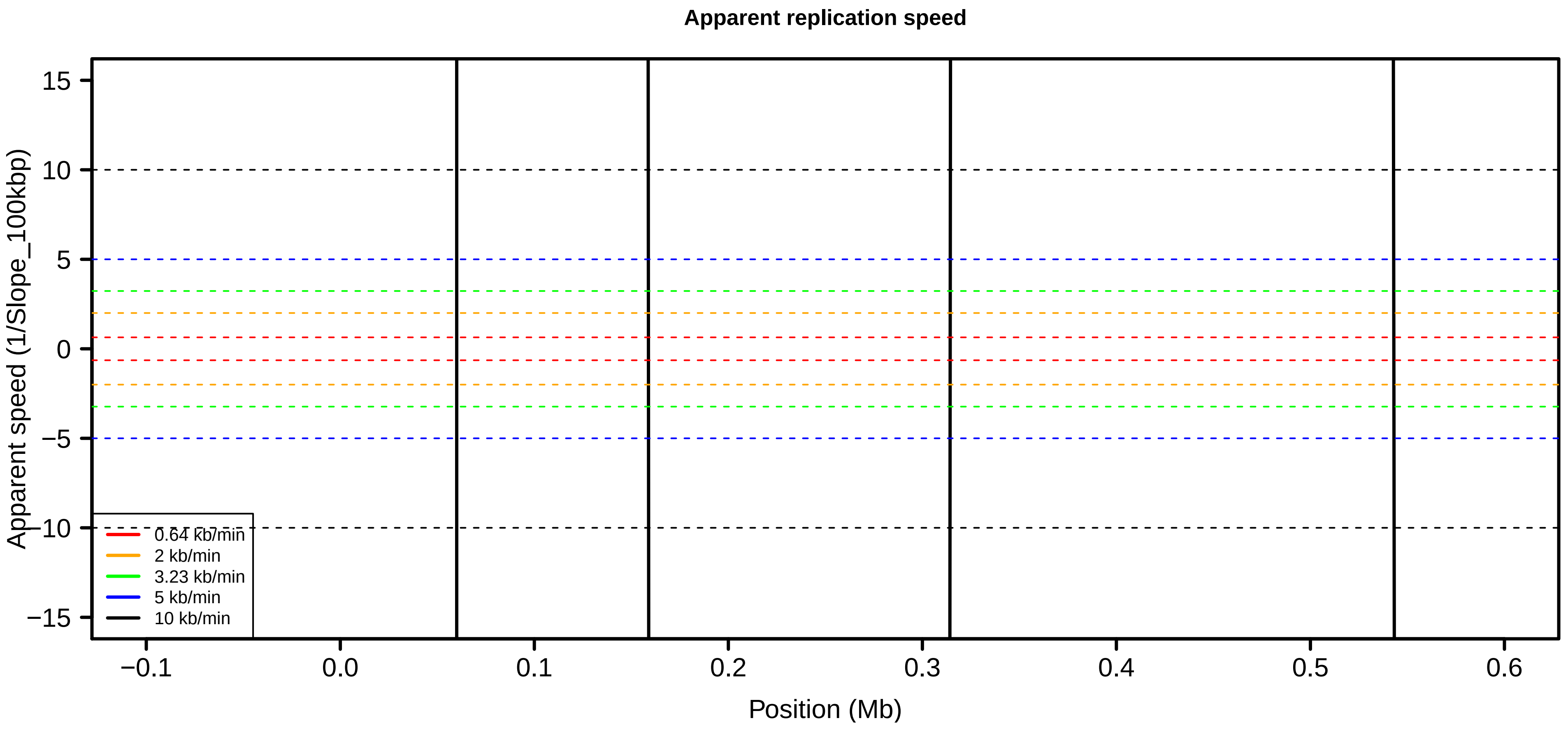

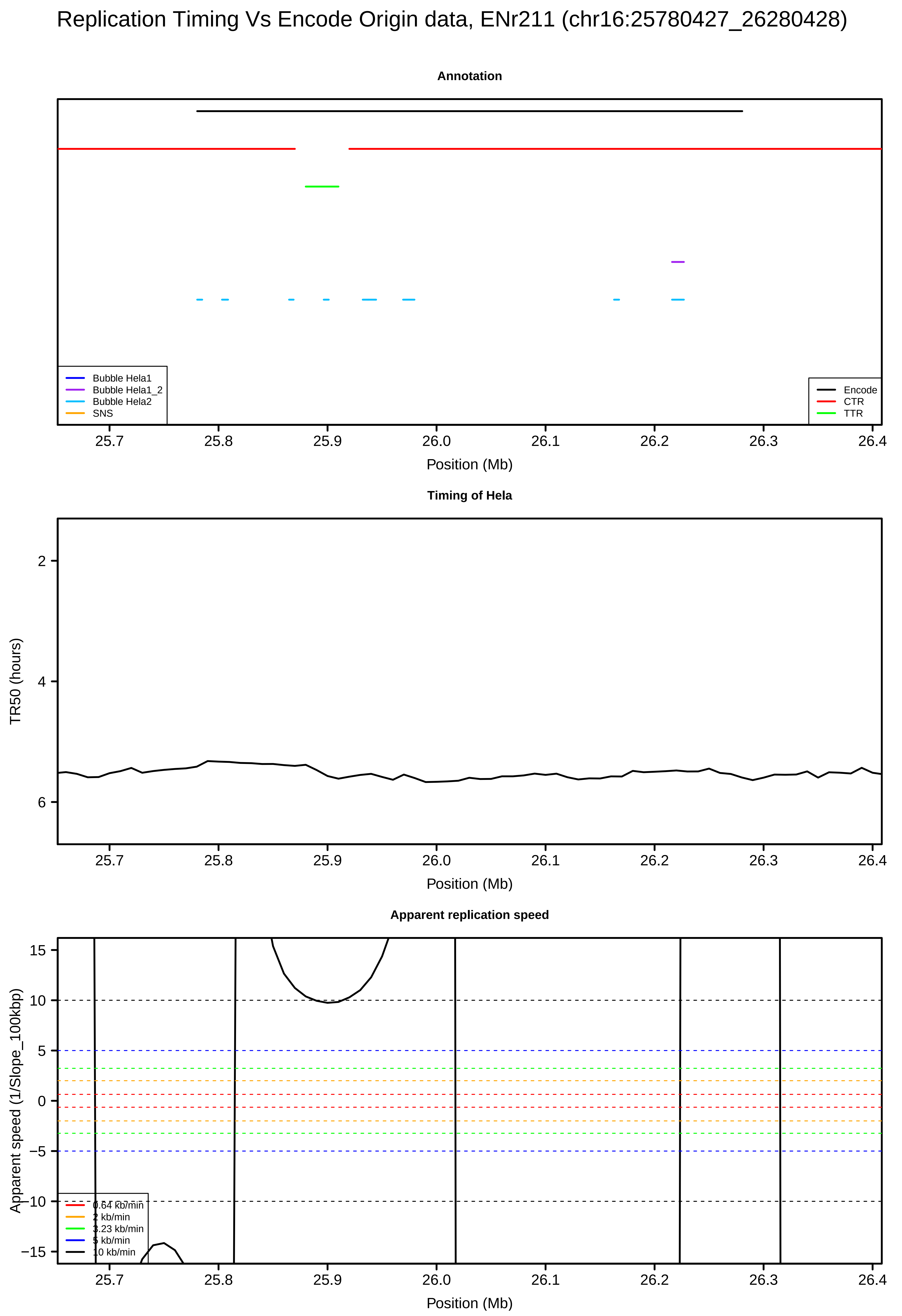
Replication Timing Vs Encode Origin data, ENr211 (chr16:25780427_26280428)
Annotation
Bubble Hela1
Bubble Hela1_2
Bubble Hela2
SNS
Encode
CTR
TTR
25.7
25.8
25.9
26.0
26.1
26.2
26.3
26.4
Position (Mb)
Timing of Hela
TR50 (hours)
2
4
6
25.7
25.8
25.9
26.0
26.1
26.2
26.3
26.4
Position (Mb)
Apparent replication speed
Apparent speed (1/Slope_100kbp)
15
10
5
0
−5
−10
−15
0.64 kb/min
2 kb/min
3.23 kb/min
5 kb/min
10 kb/min
25.7
25.8
25.9
26.0
26.1
26.2
26.3
26.4
Position (Mb)

Replication Timing Vs Encode Origin data, ENr313 (chr16:60833949_61333949)

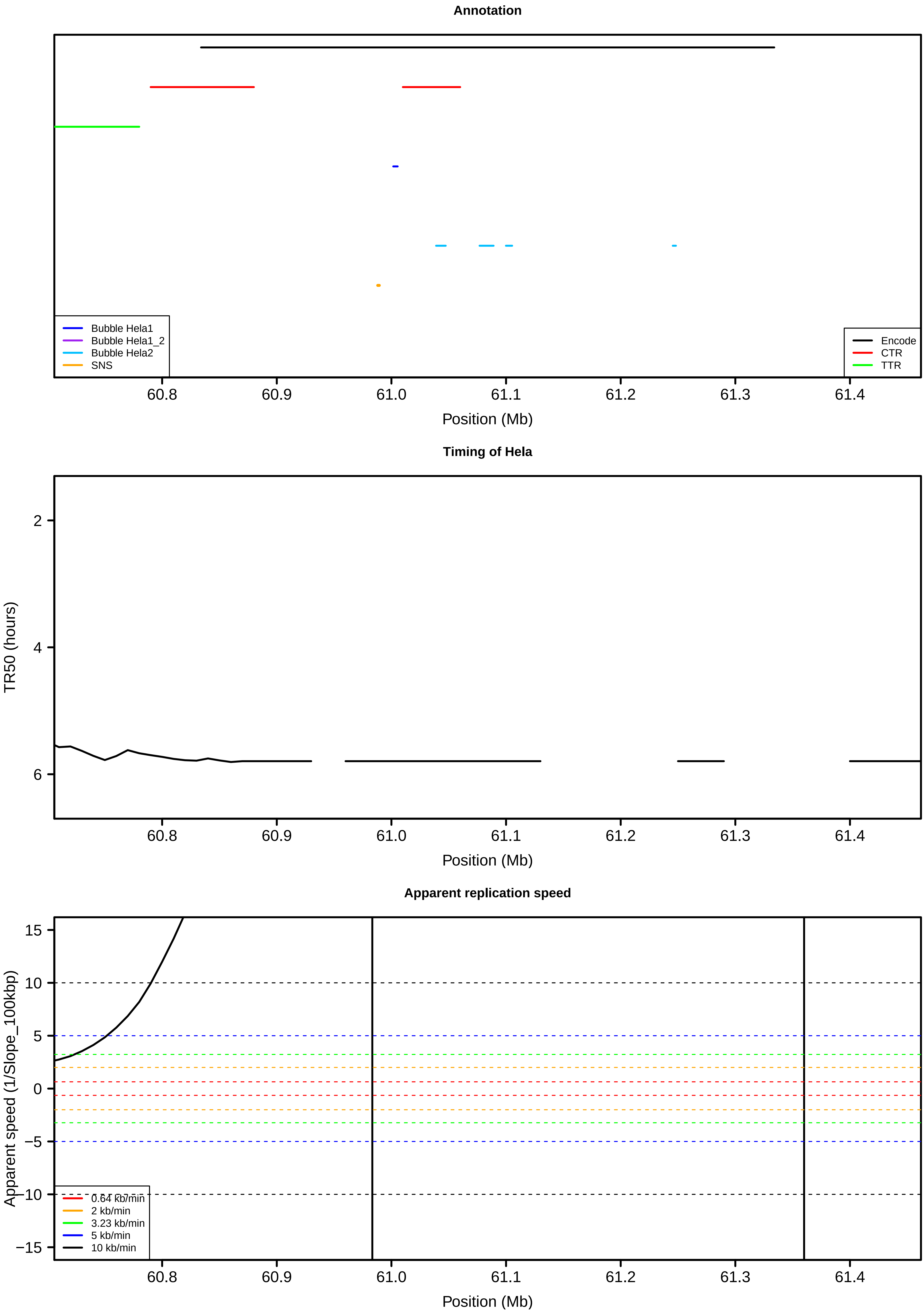

Replication Timing Vs Encode Origin data, ENr213 (chr18:23719231_24219231)

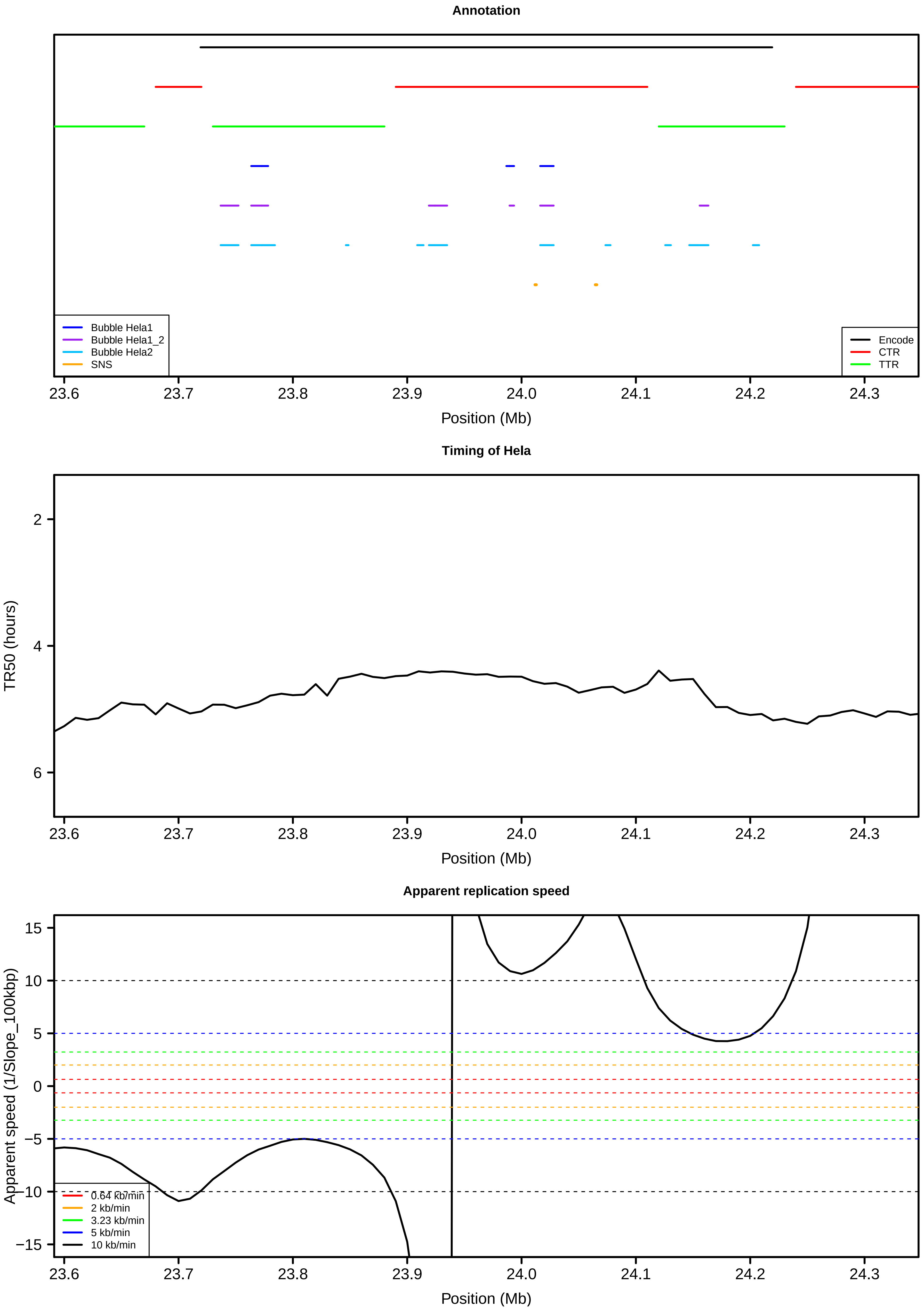

Replication Timing Vs Encode Origin data, ENr122 (chr18:59412300_59912300)

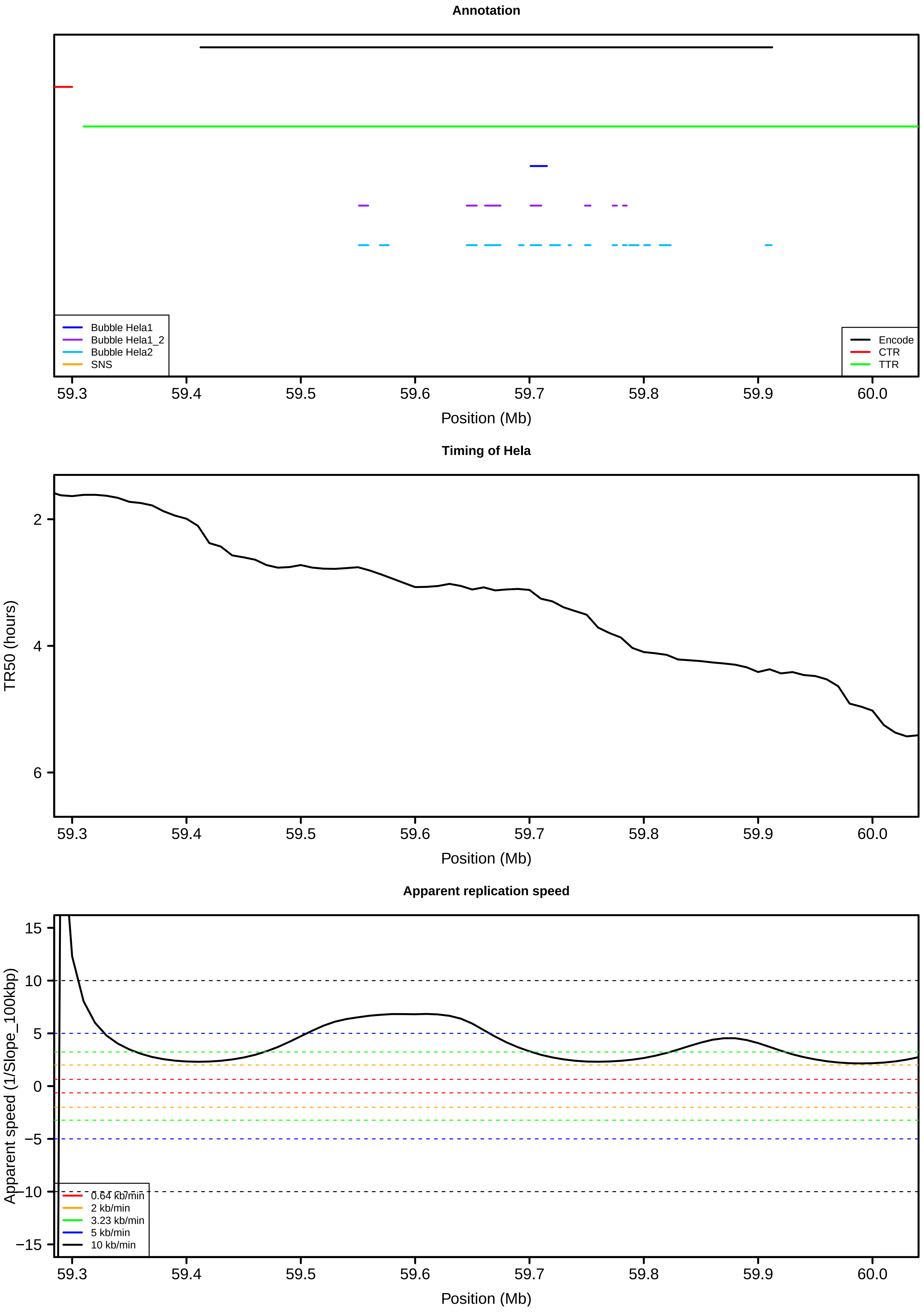

Replication Timing Vs Encode Origin data, ENm007 (chr19:59023584_60024460)

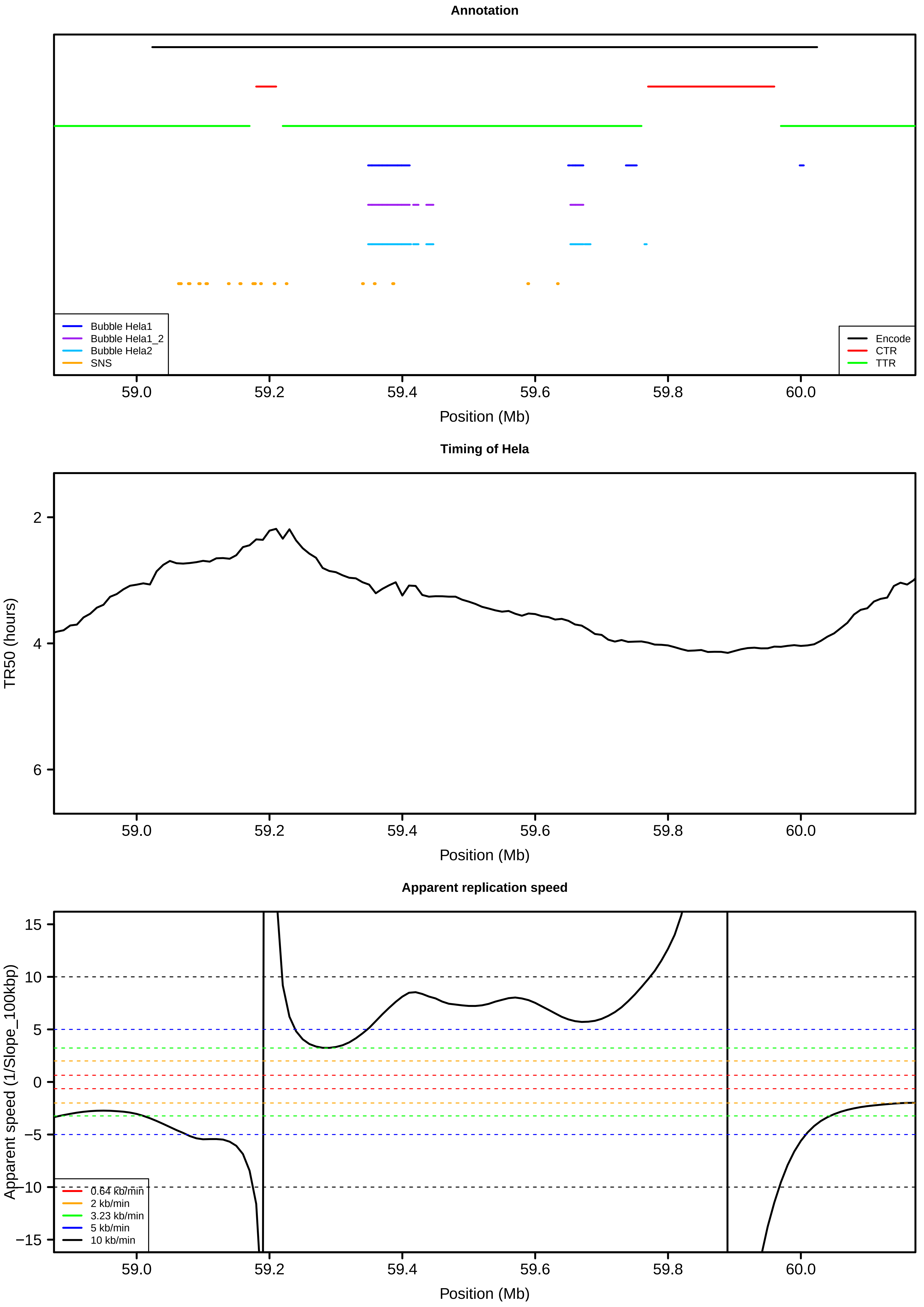

Replication Timing Vs Encode Origin data, ENr333 (chr20:33304928_33804928)

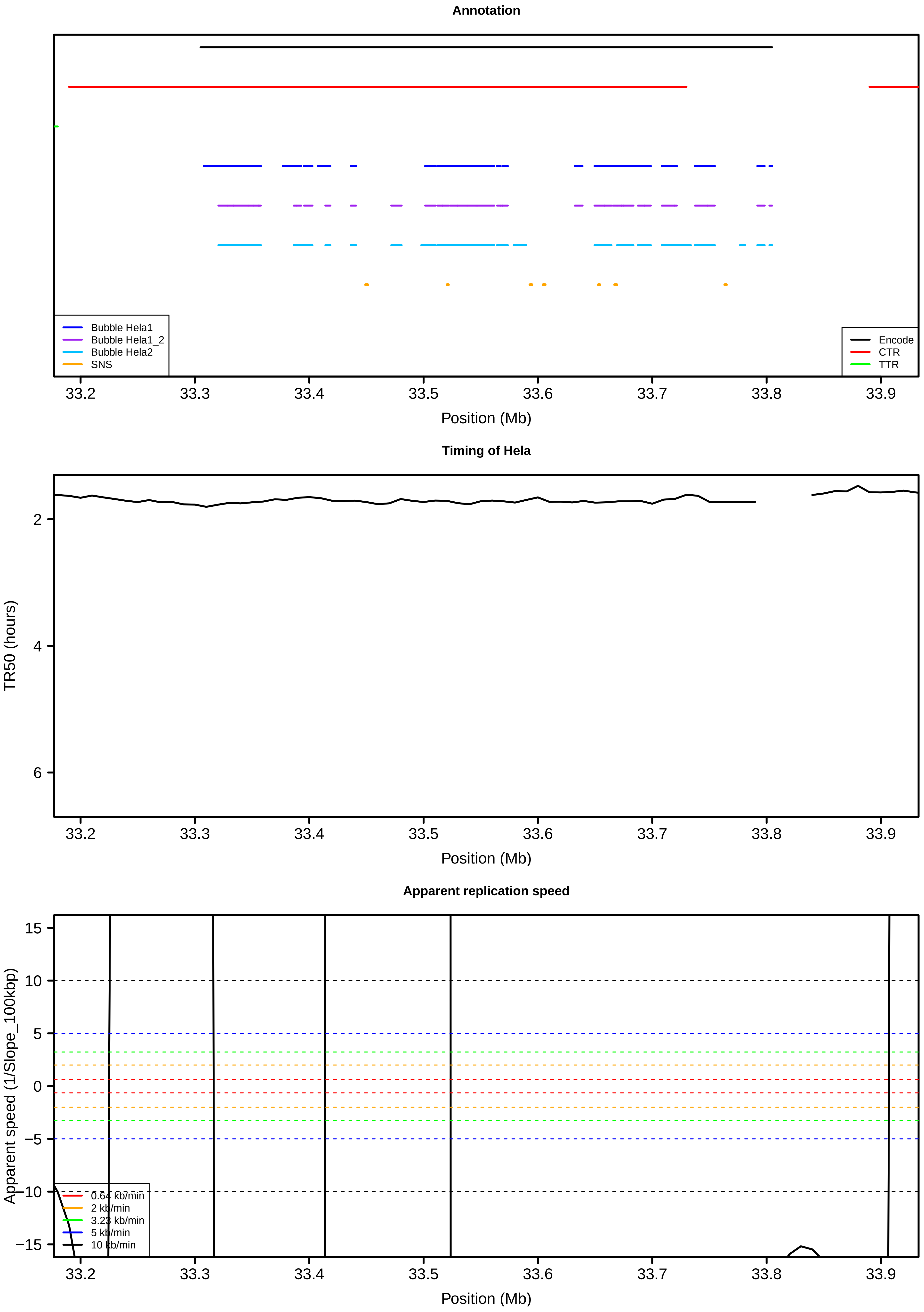

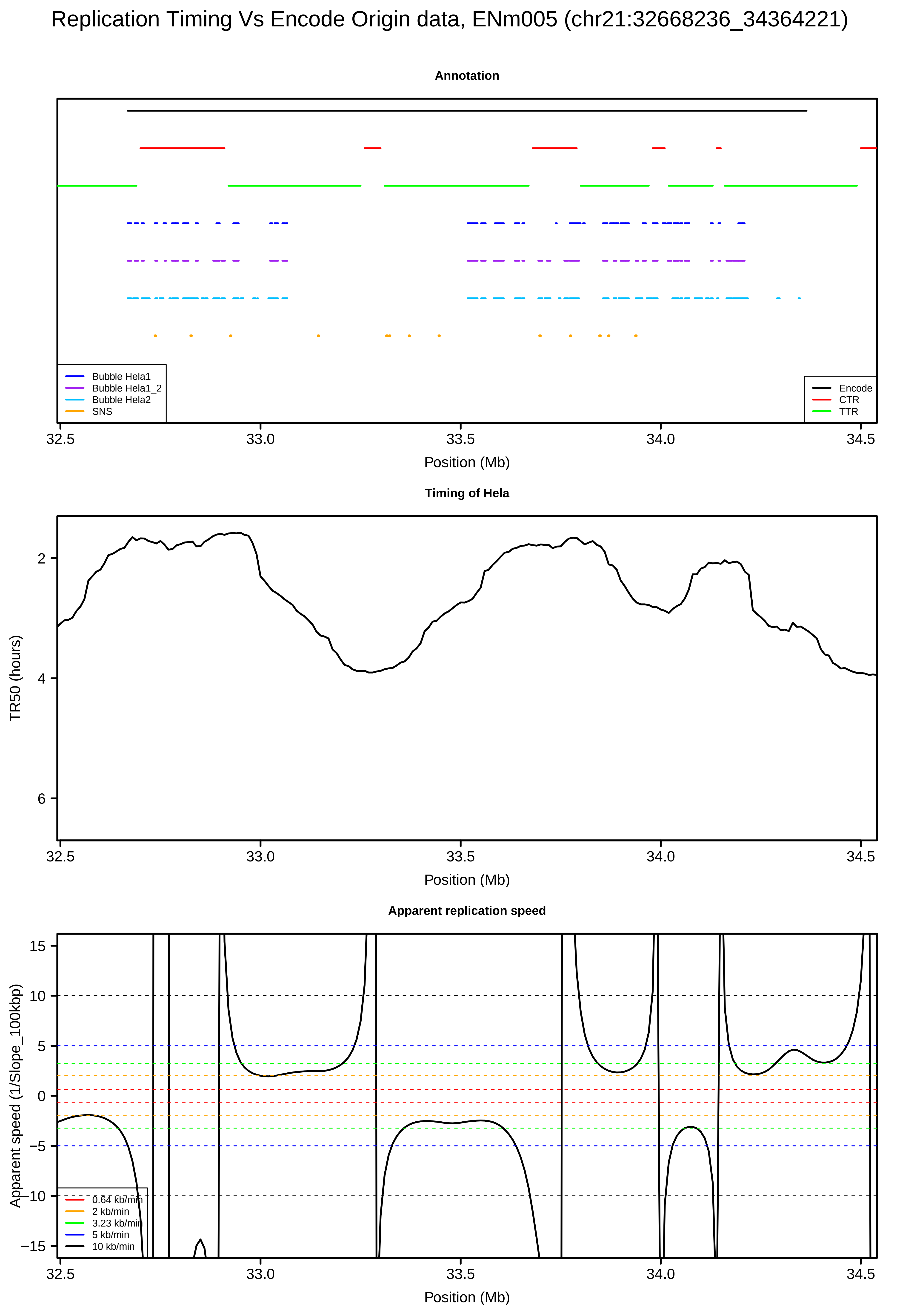
Replication Timing Vs Encode Origin data, ENm005 (chr21:32668236_34364221)
Annotation
Bubble Hela1
Bubble Hela1_2
Bubble Hela2
SNS
Encode
CTR
TTR
32.5
33.0
33.5
34.0
34.5
Position (Mb)
Timing of Hela
TR50 (hours)
2
4
6
32.5
33.0
33.5
34.0
34.5
Position (Mb)
Apparent replication speed
Apparent speed (1/Slope_100kbp)
15
10
5
0
−5
−10
−15
0.64 kb/min
2 kb/min
3.23 kb/min
5 kb/min
10 kb/min
32.5
33.0
33.5
34.0
34.5
Position (Mb)

Replication Timing Vs Encode Origin data, ENr133 (chr21:39244466_39744466)

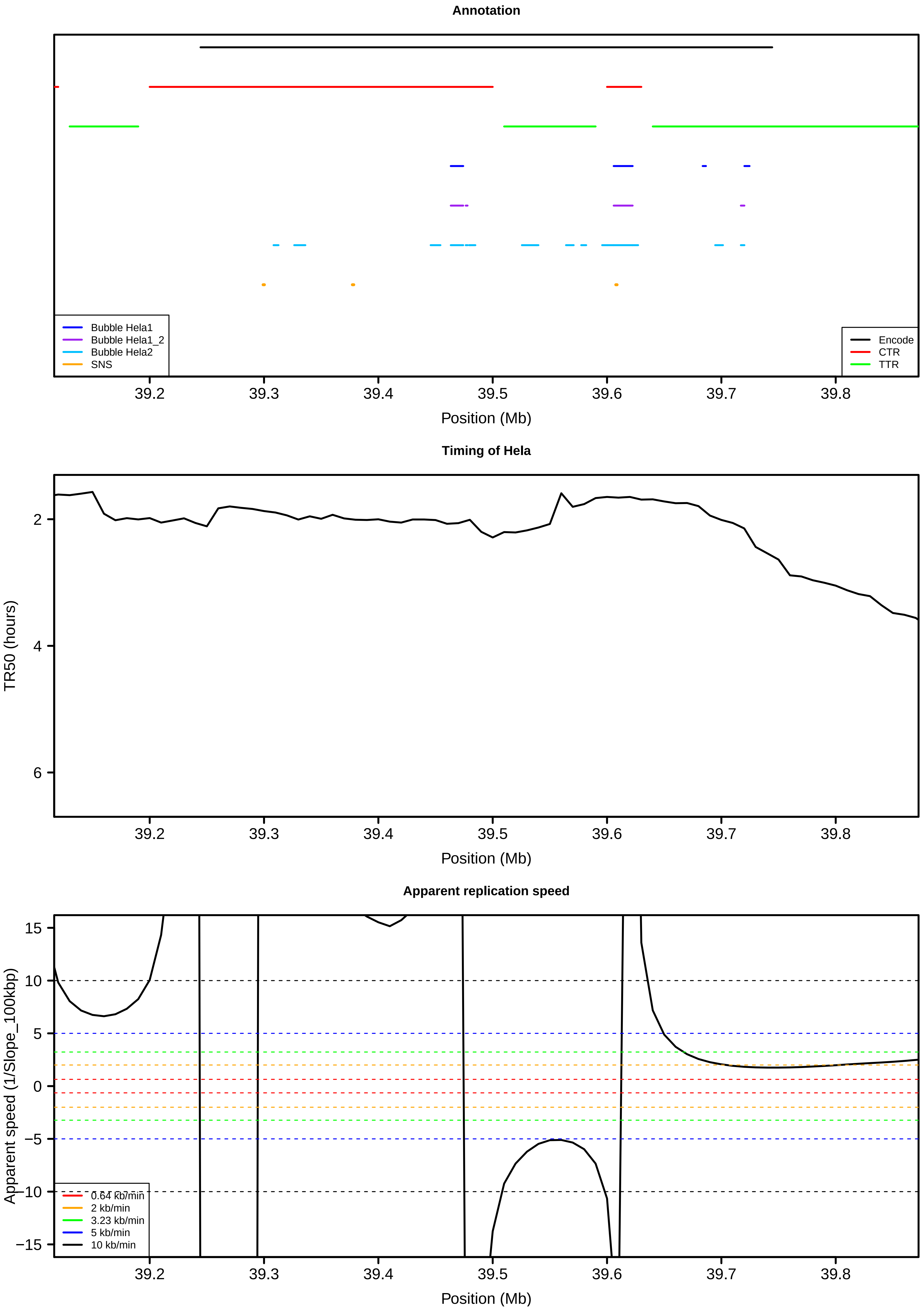

Replication Timing Vs Encode Origin data, ENm004 (chr22:30133953_31833953)

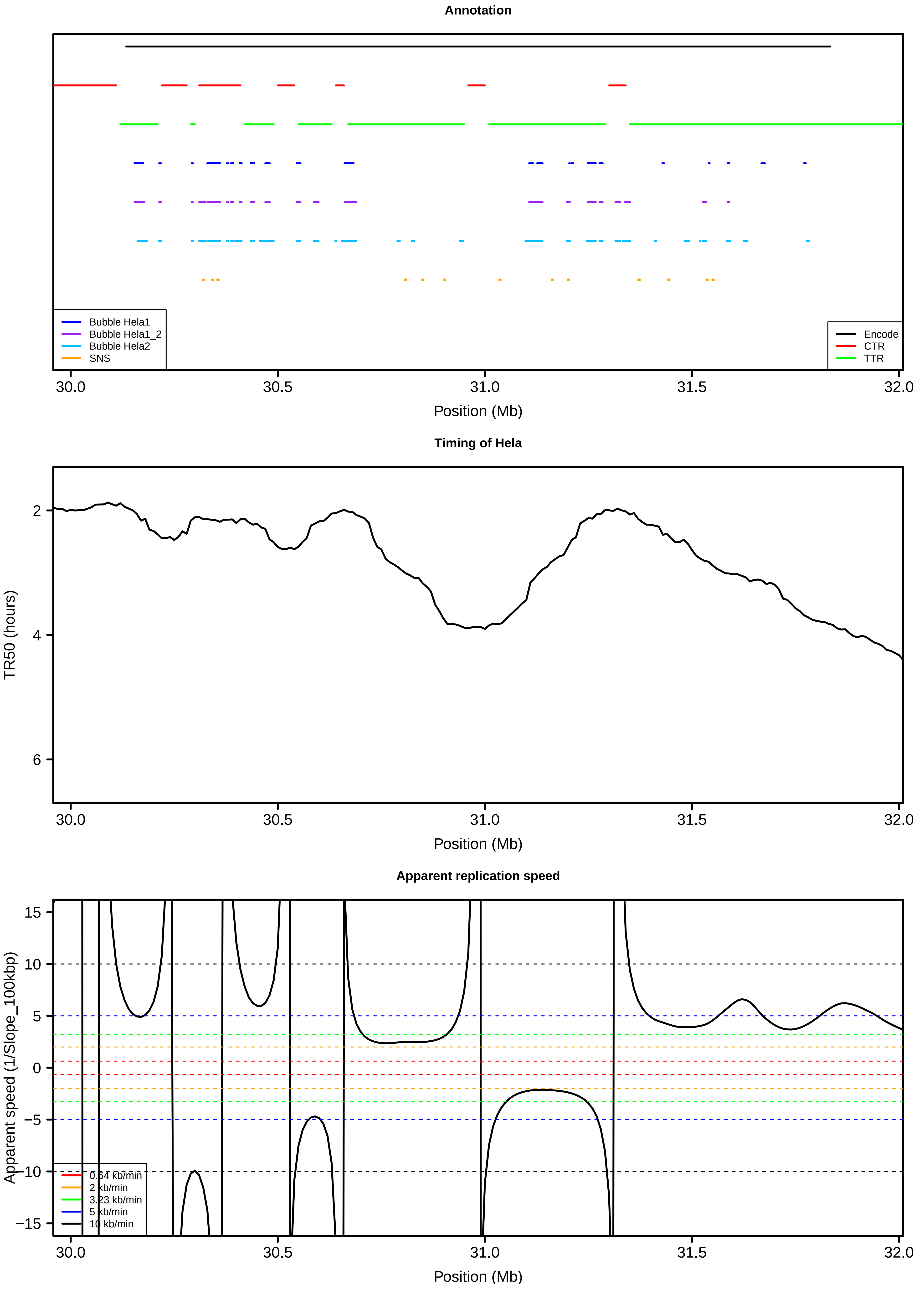

Replication Timing Vs Encode Origin data, ENr324 (chrX:122609995_123109995)

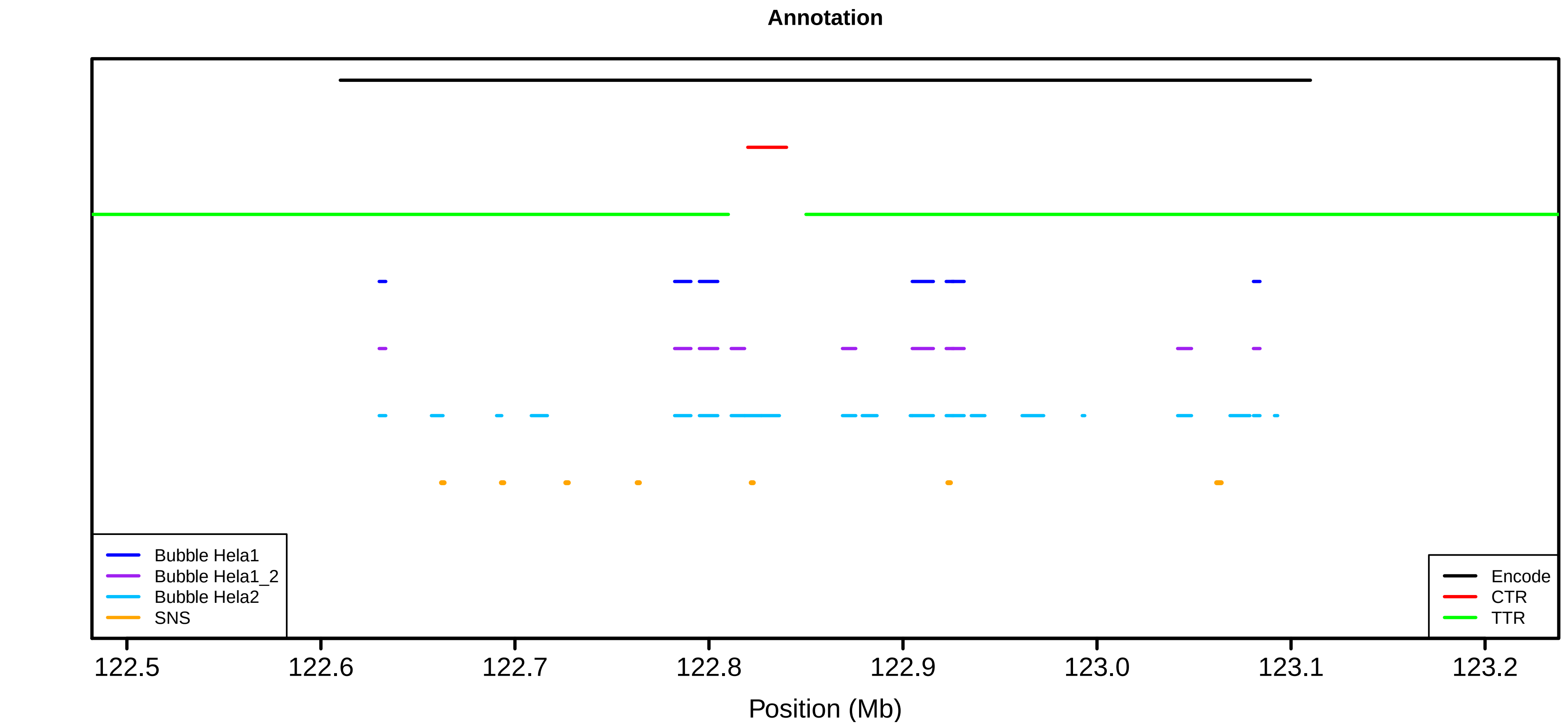

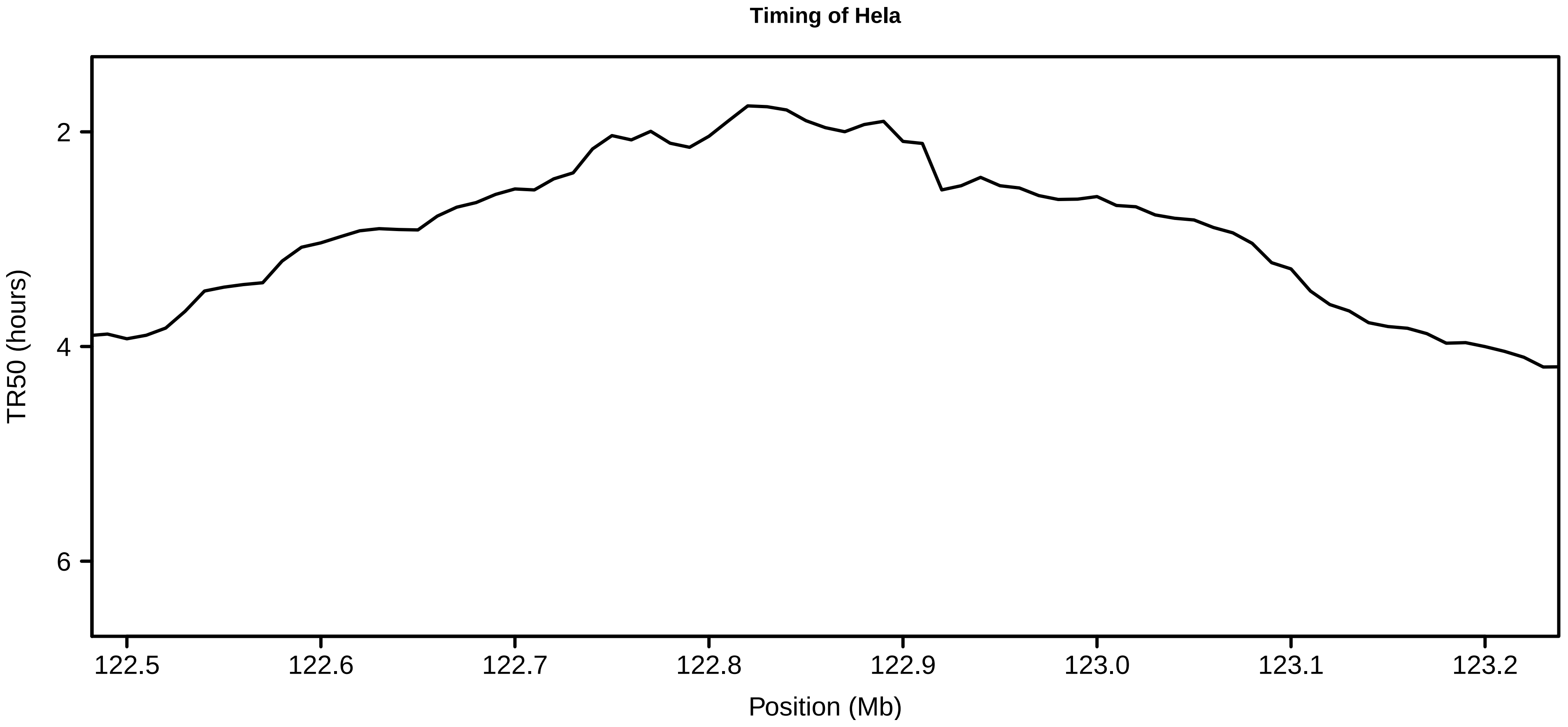

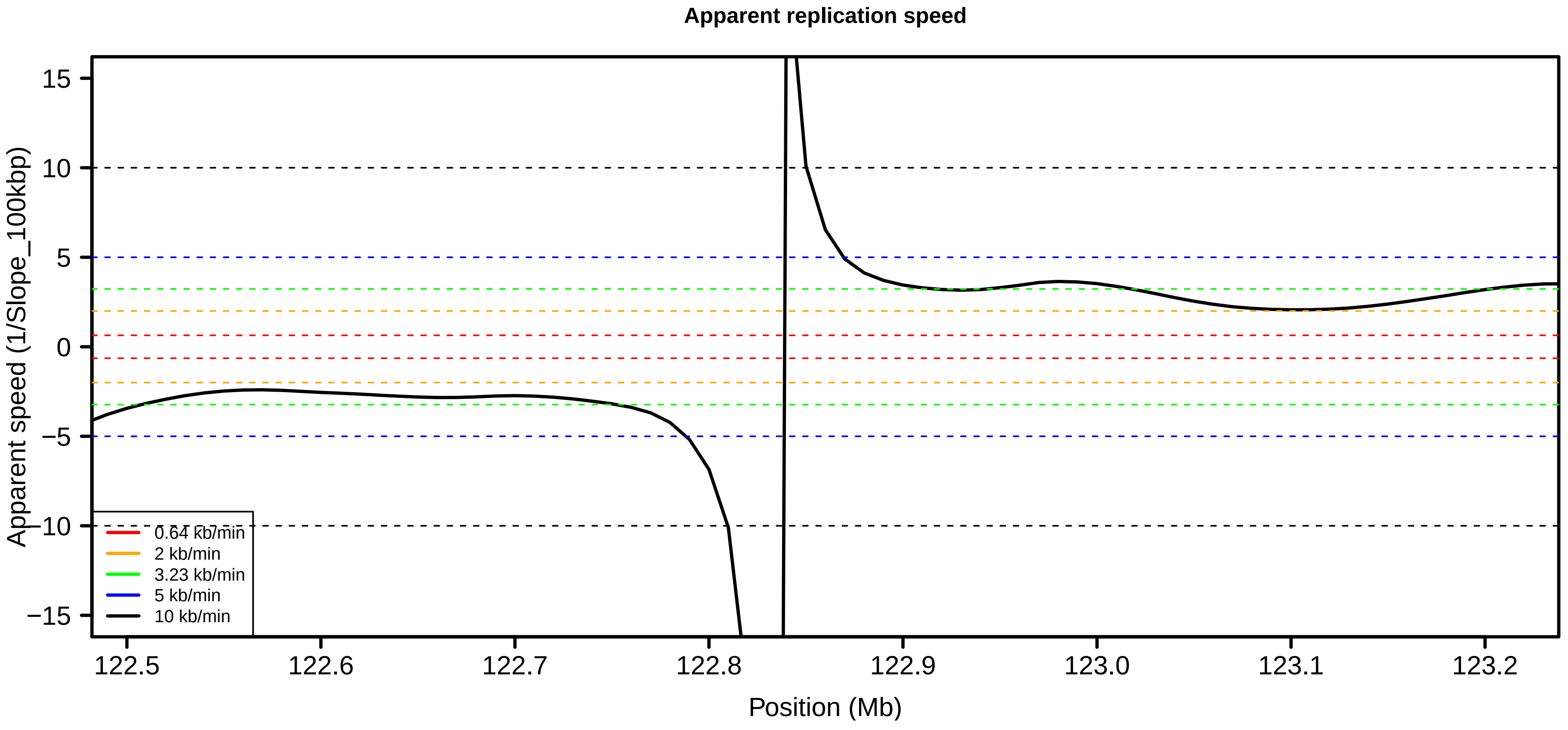

Replication Timing Vs Encode Origin data, ENm006 (chrX:152767491_154063081)

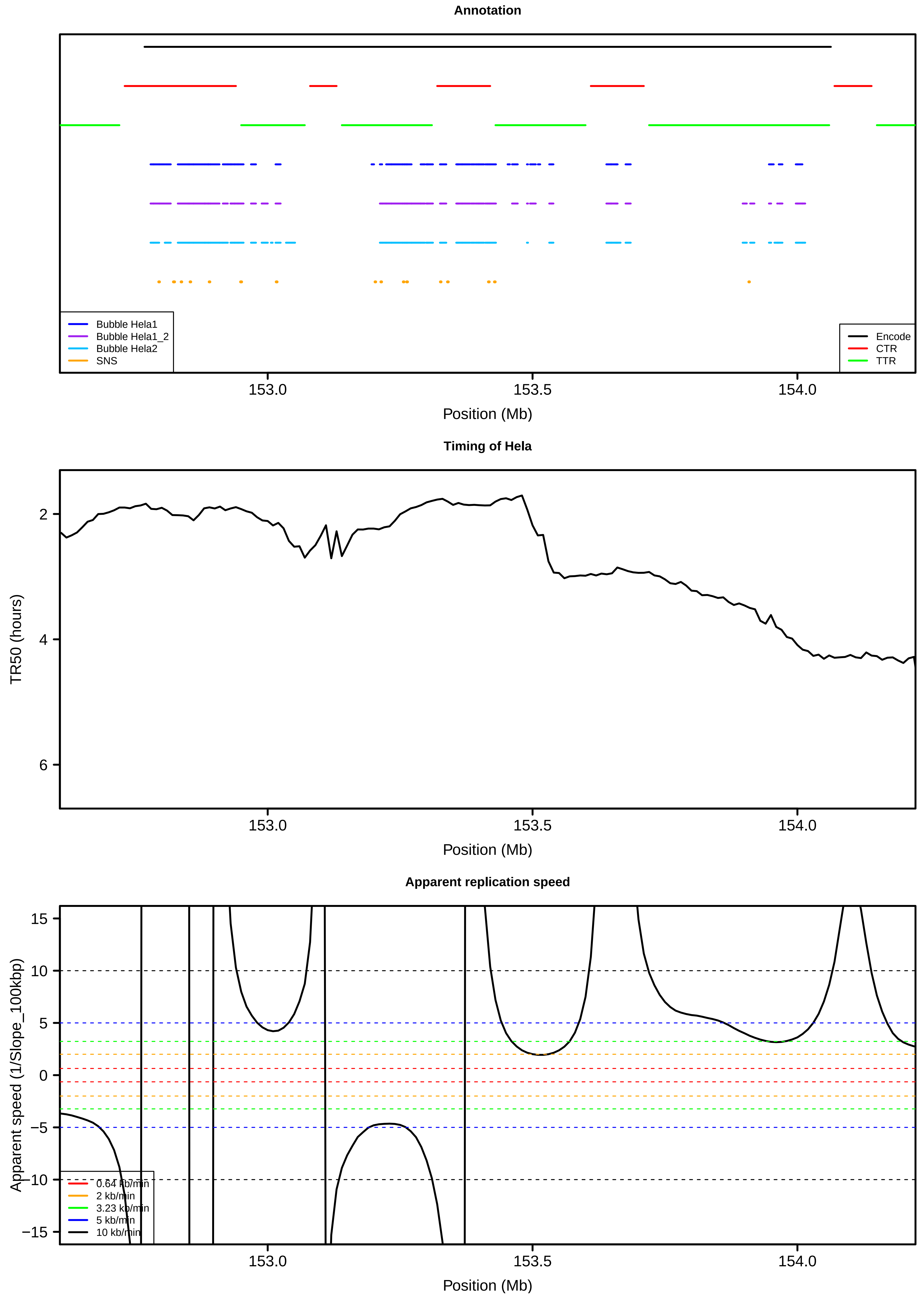

Supplement: Figure S5 — Comparison of replication timing data with replication bubble data in ENCODE regions. Each page shows: (top) the extent of each ENCODE region (dark line), the segmentation into CTRs (blue) and TTRs (red), the mapping of replication bubbles in log-phase HeLa library Rep3 (orange) and Rep4 (purple) and when the two libraries were combined (pale blue); (middle) the replication timing profile of the considered region and its immediate surroundings; (bottom) the signed apparent replication speed at scale 100 kb. (PDF) [file pcbi.1002322.s005.pdf]
